# Cytotoxic trichothecene derivatives from *Trichothecium* sp. DWS815

**DOI:** 10.1007/s13659-026-00613-3

**Published:** 2026-05-03

**Authors:** Peng-Ju Xu, Ai-Lin Liang, Wen-Yu Lu, Hong-Ping Long, Qing-Hui Xiao, Qi-An Chen, Meng-Lan Hu, Ngoc Nhu Thao Nguyen, Shao Liu, Jing Li, Wen-Xuan Wang

**Affiliations:** 1 Xiangya School of Pharmaceutical Sciences, Central South University, Changsha, 410013 People’s Republic of China; 2 Department of Pharmacy, National Clinical Research Center for Geriatric Disease, Xiangya Hospital, Central South University, Changsha, 410008 People’s Republic of China; 3 Center for Medical Research and Innovation, the First Hospital of Hunan University of Chinese Medicine, Changsha, People’s Republic of China

**Keywords:** Trichothecenes, *Trichothecium* sp., Secondary metabolite, Cytotoxic activity

## Abstract

Ten novel trichothecene sesquiterpenoids including two new *seco*-trichothecenes, trichotheciumones A (**1**) and B (**2**), a new trichothecene sesquiterpenoid glycoside, trichothecinoside A (**3**), and seven new trichothecene sesquiterpenoids, trichothecrotocins T−Z (**4**−**10**), together with three new natural products (**11**−**13**) and thirteen known compounds (**14**−**26**), were isolated from the soil fungus *Trichothecium* sp. DWS815. The structures and absolute configurations of the new compounds were elucidated by extensive spectroscopic analyses and quantum chemistry ECD calculations. Given the notable anticancer properties of known trichothecenes, the isolated compounds were evaluated for the cytotoxic activities against three cancer cell lines (HCT116, 4T1, MHCC97H) and one normal cell line (GES-1). Cell cycle analysis revealed new compounds **7** and **8** induced G2/M phase arrest in HCT116 cancer cells, which resulted to cell proliferation inhibition activity.

**Graphical Abstract:**

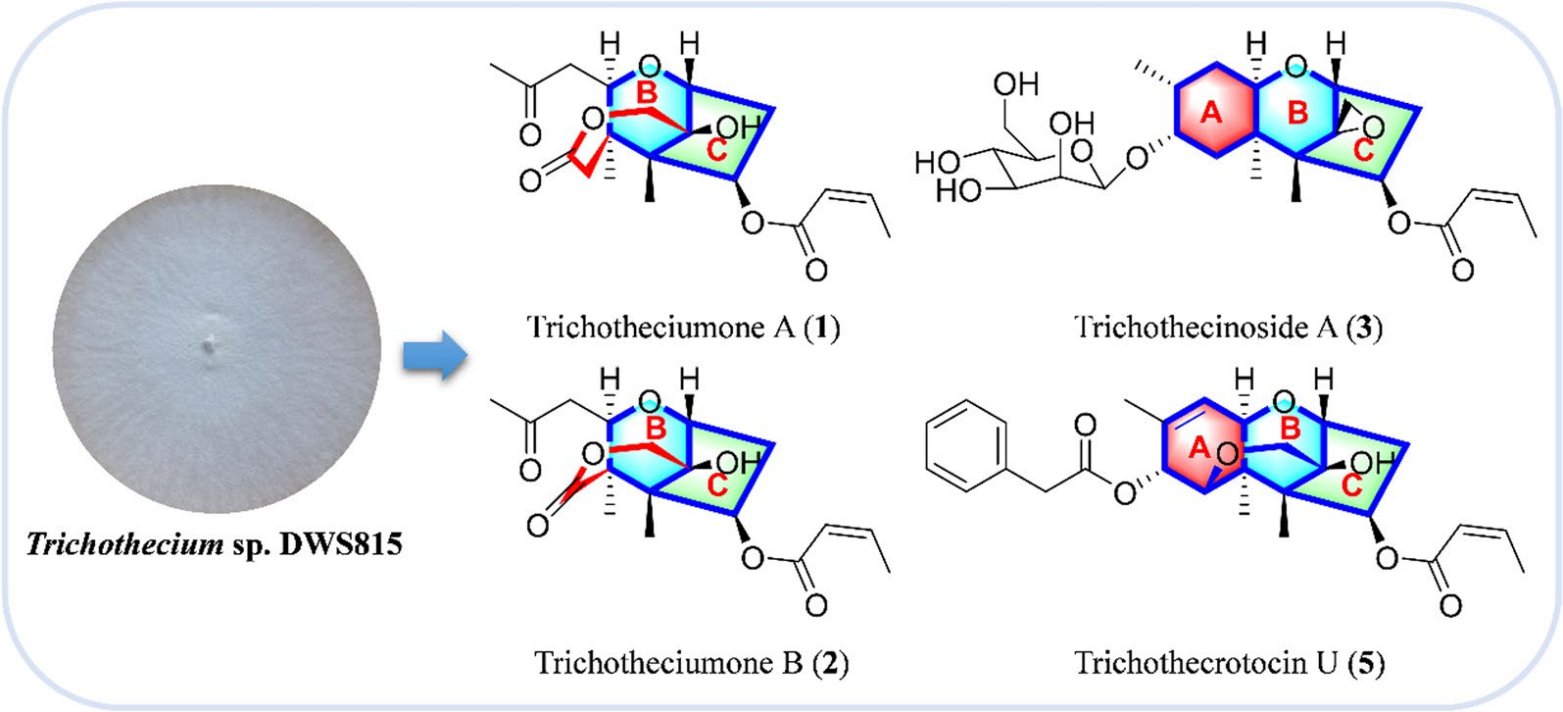

**Supplementary Information:**

The online version contains supplementary material available at 10.1007/s13659-026-00613-3.

## Introduction

Trichothecenes, a family of sesquiterpenoids produced by various fungal genera such as *Aspergillus*, *Cephalosporium*, *Fusarium*, *Myrothecium*, *Stachybotrys*, *Verticimonosporium* and *Trichothecium* [1, 2], are common contaminants of grains [3]. This family is divided into two main groups, simple and macrocyclic trichothecenes [4]. The simple trichothecenes share a core ring structure and exhibit cytotoxicity by inhibiting eukaryotic protein synthesis and disrupting mitochondrial and ribosomal functions [5–8]. The 12,13-epoxytrichothec-9-ene (EPT) is cited as the cause of this toxicity [9], and structural diversity within this family arises from various substitution patterns on the EPT core. Recent years have witnessed the continuous discovery of trichothecenes from the genus of *Trichothecium* [10–13].

**Fig. 1:**
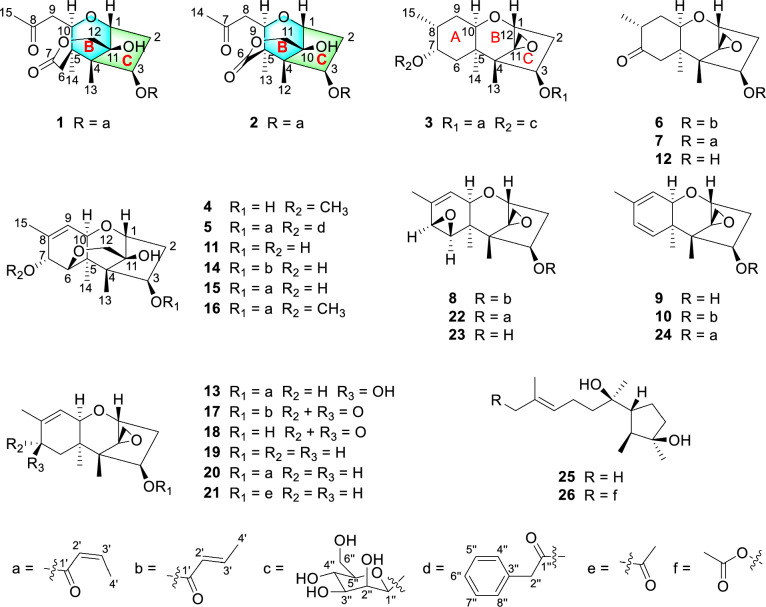
Structures of compounds **1**−**26**

Building upon the long-standing focus on discovering the structurally novel and biologically active compounds from soil fungi [14–17], our previous chemical investigation of *Myrothecium verrucaria* PA 57 led to the discovery of novel macrocyclic trichothecenes exhibiting cytotoxic activities [18]. In our persistent endeavor to mine the novel types of trichothecene natural products with higher cytotoxic activities against cancer cell lines and lower cytotoxic activities toward normal cell lines, the fungal strain *Trichothecium* sp. DWS815 was isolated from a soil sample collected in Daweishan National Forest Park, Hunan Province, China. A comprehensive LC–MS analysis of the fungal fermentation extract revealed a metabolite profile abundant in simple trichothecenes, suggesting the strain's potential to produce novel trichothecene derivatives. Chemical investigation of this strain led to the isolation of ten previously undescribed trichothecene derivatives, trichotheciumones A (**1**) and B (**2**), trichothecinoside A (**3**), and trichothecrotocins T–Z (**4**–**10**), together with three new natural products (**11**–**13**) and thirteen known compounds (**14**–**26**) (Fig. 1). The structures and absolute configurations of all new compounds were established through spectroscopic analysis and quantum chemistry calculations of electronic circular dichroism (ECD). This paper details the purification, structure elucidation, and cytotoxicity of these compounds.

## Results and discussion

Compound **1** was obtained as a white powder. The molecular formula was determined to be C_19_H_26_O_7_ based on HRESIMS at *m/z* 367.1758 [M + H]^+^ (calcd for C_19_H_27_O_7_, 367.1751), corresponding to seven degrees of unsaturation. The ^1^H NMR spectrum (Table 1) of **1** indicated the presence of four methyl groups at *δ*_H_ 0.98 (s, H_3_-14), 1.02 (s, H_3_-13), 2.17 (dd, *J* = 7.2, 1.8 Hz, H_3_-4'), and 2.17 (s, H_3_-15); four methylene groups at *δ*_H_ 2.13/2.53 (ddd, *J* = 15.8, 4.9, 3.4 Hz, H_a_-2; dd, *J* = 15.8, 8.0 Hz, H_b_-2), 2.34/2.89 (d, *J* = 15.1 Hz, H_a_-6; d, *J* = 15.1 Hz, H_b_-6), 2.44/3.10 (dd, *J* = 16.6, 2.3 Hz, H_a_-9; dd, *J* = 16.6, 9.1 Hz, H_b_-9), and 4.21/4.30 (d, *J* = 13.9 Hz, H_a_-12; d, *J* = 13.9 Hz, H_b_-12); five methines including two *cis*-olefinic methines at *δ*_H_ 5.77 (dq, *J* = 11.4, 1.8 Hz, H-2') and 6.45 (dq, *J* = 11.4, 7.2 Hz, H-3') as well as three oxygenated methines at *δ*_H_ 4.01 (d, *J* = 9.1 Hz, H-10), 4.45 (d, *J* = 4.8 Hz, H-1), and 5.63 (dd, *J* = 8.0, 3.4 Hz, H-3). The ^13^C NMR (Table 4) and DEPT spectra confirmed the presence of 19 carbon resonances assignable to four methyl groups (*δ*_C_ 7.4, 15.6, 20.6, 31.5), four sp^3^ methylene groups including one downfield oxygenated carbon (*δ*_C_ 36.5, 39.4, 42.0, 66.8), three sp^3^ oxygenated methine carbons (*δ*_C_ 72.3, 75.3, 81.7), three non-protonated sp^3^ carbons including one oxygenated carbon (*δ*_C_ 43.1, 53.4, 76.5), two sp^2^ olefinic carbons (*δ*_C_ 119.3, 147.9), one ketone carbonyl carbon (*δ*_C_ 206.5), and two ester carbonyl carbons (*δ*_C_ 164.8, 173.6).

**Table 1:** ^1^H NMR Spectroscopic Data for Compounds **1**−**4**

Position	**1**^*a*^	**2**^*a*^	**3**^*a*^	**4**^*a*^
	*δ*_H_, mult. (*J* = Hz)	*δ*_H_, mult. (*J* = Hz)	*δ*_H_, mult. (*J* = Hz)	*δ*_H_, mult. (*J* = Hz)
1	4.45, d (4.8)	4.21, d (5.4)	3.80, overlapped	4.27, d (5.4, 7.7)
2	H_a_ 2.13, ddd (15.8, 4.9, 3.4) H_b_ 2.53, dd (15.8, 8.0)	H_a_ 2.19, overlapped H_b_ 2.66, dd (16.0, 8.1)	H_a_ 1.99, overlapped H_b_ 2.51, d (15.3, 7.7)	H_a_ 2.09, overlapped H_b_ 2.46, dd (15.6, 7.7)
3	5.63, dd (8.0, 3.4)	5.54, dd (8.1, 3.3)	5.53, brs	4.29, m
6	H_a_ 2.34, d (15.1) H_b_ 2.89, d (15.1)	–	H_a_ 1.83, overlapped H_b_ 1.95, overlapped	3.99, brs
7	–	–	3.80, overlapped	3.45, brs
8	–	H_a_ 2.46, dd (17.1, 9.0) H_b_ 2.54, dd (17.1, 1.8)	1.93, overlapped	–
9	H_a_ 2.44, dd (16.6, 2.3) H_b_ 3.10, dd (16.6, 9.1)	4.02, dd (9.0, 1.8)	H_a_ 1.46, d (13.8) H_b_ 1.77, overlapped	5.57, d (5.6)
10	4.01, d (9.1)	–	3.52, overlapped	3.54, d (5.6)
11	–	H_a_ 4.24, d (12.0) H_b_ 4.71, d (12.0)	–	–
12	H_a_ 4.21, d (13.9) H_b_ 4.30, d (13.9)	1.10, s	H_a_ 2.85, d (4.1) H_b_ 3.12, d (4.1)	H_a_ 3.72, d (11.5) H_b_ 3.97, d (11.5)
13	1.02, s	1.20, s	0.67, s	1.20, s
14	0.98, s	2.16, s	1.18, s	0.80, s
15	2.17, s		0.90, d (6.8)	1.84, s
16				3.49, s
2'	5.77, dq (11.4, 1.8)	5.78, dq (11.4, 1.8)	5.81, dd (11.4, 1.8)	
3'	6.45, dq (11.4, 7.2)	6.44, dq (11.4, 7.3)	6.34, d (11.4, 7.3)	
4'	2.17, dd (7.2, 1.8)	2.17, dd (7.3, 1.8)	2.14, d (7.3, 1.8)	
1''			4.48, brs	
2''			3.99, brs	
3''			3.52, overlapped	
4''			3.80, overlapped	
5''			3.21, d (11.0)	
6''			3.86, overlapped	

^*a*^Recorded in CDCl_3_, 600 MHz

**Fig. 2:**
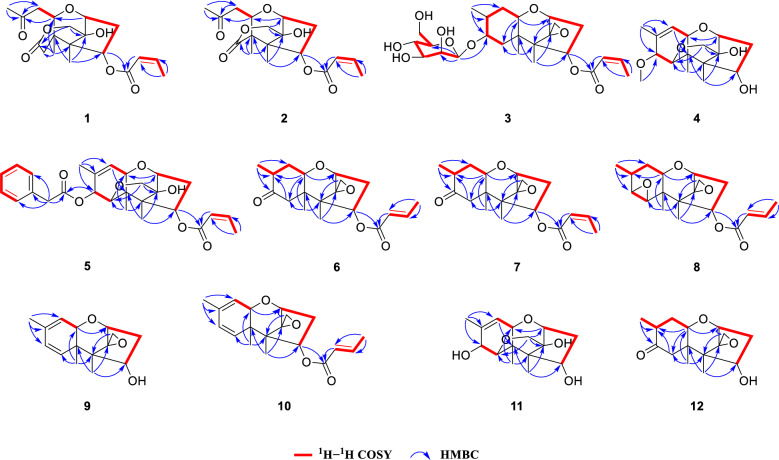
Key HMBC and ^1^H−^1^H COSY correlations of compounds **1**−**12**

Key ^1^H–^1^H COSY correlations (H-1/H-2/H-3, H-10/H-9, and H-2'/H-3'/H-4') established three independent spin–spin coupling systems (Fig. 2). A crotonyl group was connected to C-3 by HMBC correlations from H-3 and H-2' to C-1' as well as from H-4' to C-2' and C-3'. Further detailed 1D and 2D spectra analysis and comparison with a known co-isolated compound, trichothecene analogue (**15**) [19], indicated compound **1** bore a resemblance with the trichothecene central skeleton. The B and C rings in **1** were constructed by HMBC correlations from H-1 to C-10 and from H_3_-13 to C-3, C-5 and C-11. An acetonyl group was attached to C-10 in the B ring based on the HMBC correlations from H-10, H-9 and H_3_-15 to the carbonyl carbon C-8 (*δ*_C_ 206.5) and from H_3_-15 to C-9 and ^1^H–^1^H COSY correlation between H-10 and H-9. Moreover, HMBC correlations from H_2_-12 to C-11 and C-7, from H_3_-14 to C-4, C-10 and C-6, together with a long-range (^4^*J*_CH_) correlation from H_3_-14 to C-7 confirmed a lactone bridge between C-5 and C-11 across the B ring. The aforementioned planar structure assignment confirmed that compound **1** possessed a 7,8-*seco*-trichothecene core, representing the first reported isolation of this specific scaffold. NOESY correlations of H-3/H-10/H_3_-14, as well as H-1/H_a_-12 and H_3_-13/H_b_-12, indicated that H-3, H-10, and H_3_-14 were *α*-oriented, while H-1, H_3_-13, and OH-11 resided on the opposite face. This relative configuration of **1** aligned with the biosynthetic origin of trichothecene sesquiterpenoids. The absolute configuration was verified based on the agreement between the experimental and calculated ECD spectra. Finally, the absolute configuration was determined to be 1*R*,3*R*,4*S*,5*R*,10*R*,11*S* (Fig. 4). Thus, **1** was established to be trichotheciumone A, a 7,8-*seco*-trichothecene.

**Fig. 3:**
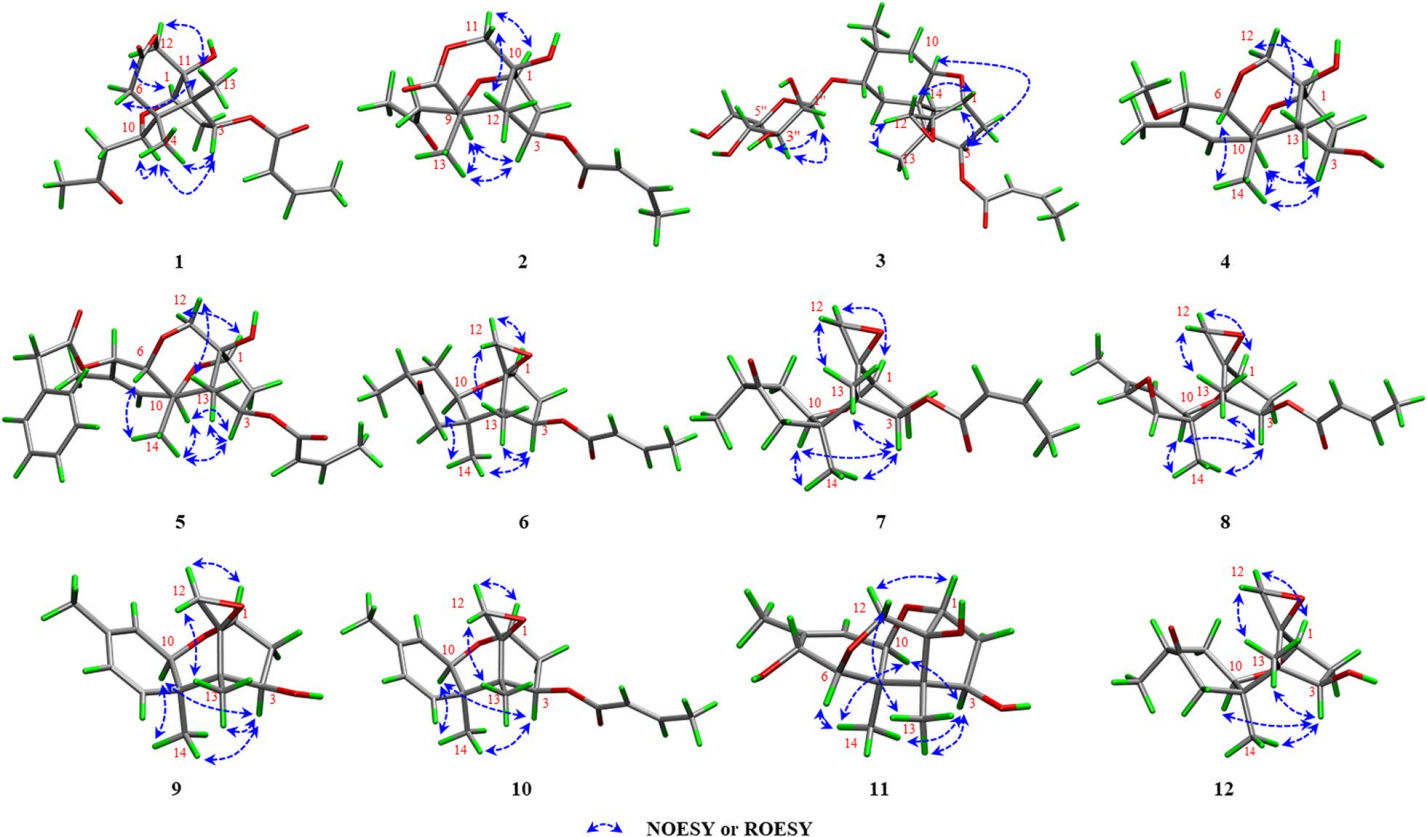
Key NOESY or ROESY (blue arrows) correlations of compounds** 1**−**12**

**Fig. 4:**
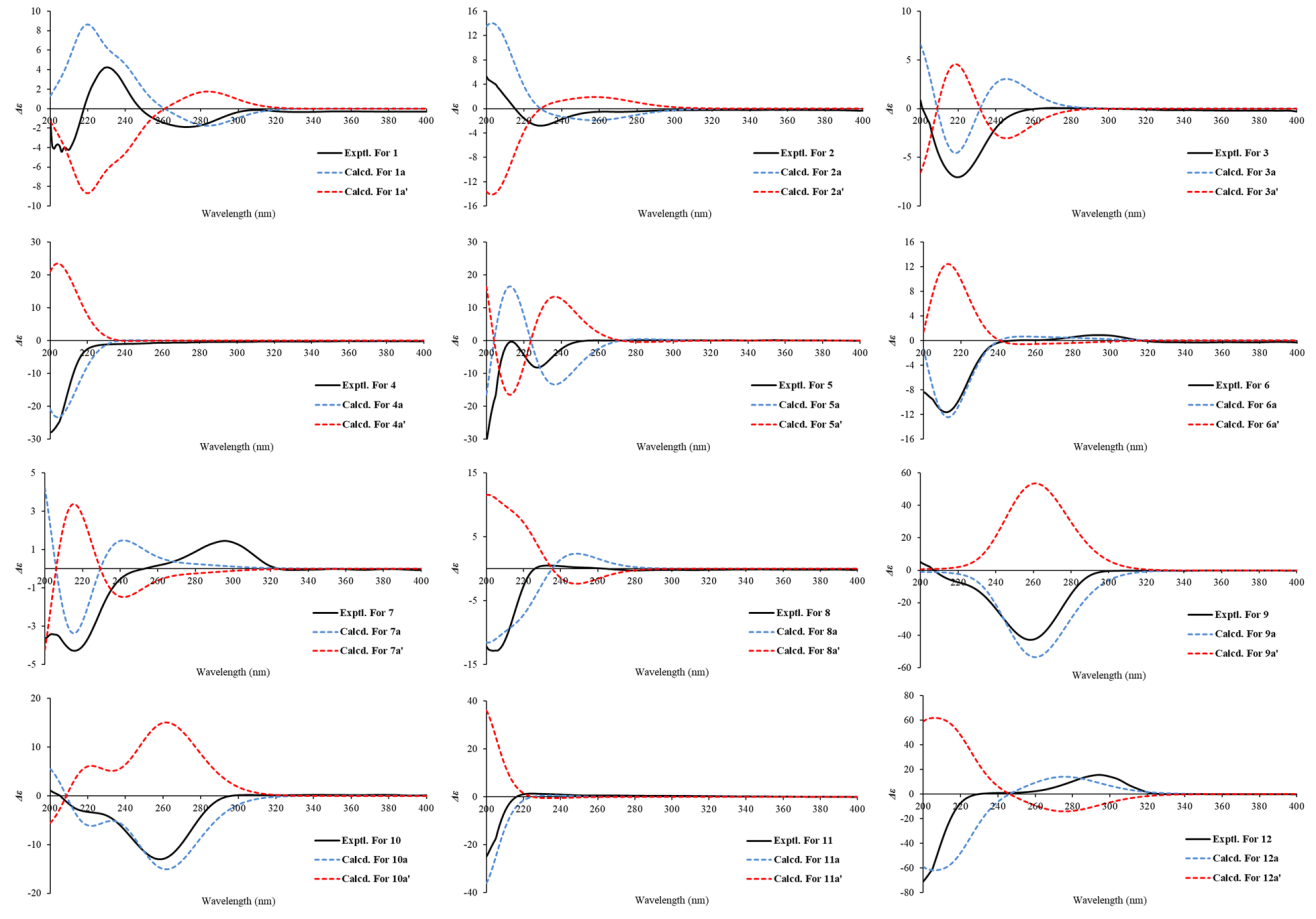
Calculated and experimental ECD spectra of compounds** 1**−**12**

Compound **2** was obtained as a white powder with the molecular formula of C_18_H_24_O_7_, determined by HRESIMS at *m/z* 353.1599 [M + H]^+^ (calcd for C_18_H_25_O_7_, 353.1595) and corresponding to seven degrees of unsaturation. The NMR spectrum for **2** (Tables 1 and 4) showed similarities to those of **1**. The key structural difference was the absence of a methylene unit in **2** at the bridged lactone moiety, suggesting it to be a previously undescribed 7,8-*seco*-7-*nor*-trichothecene. The B and C rings, along with the crotonyl group and the acetonyl group were assigned by comparison of the key NMR signals (^1^H–^1^H COSY, HSQC, and HMBC correlations) with those of **1**, as depicted in Fig. 2. The bridged lactone moiety was assigned between C-5 and C-10 across the B ring according to the critical HMBC correlations from H-9, H-11, and H_3_-13 to C-6 and from H_2_-11 to C-10 and C-6. Moreover, NOESY correlations of H_3_-12/H-3/H-9/H_3_-13, and H_3_-12/H_a_-11, and H-1/H_b_-11 supported the relative configuration shown in Fig. 3. The relative configuration of **2** was consistent with the biosynthetic origin of trichothecene sesquiterpenoids. By comparing the experimental and calculated ECD spectra (Fig. 4), the absolute configuration of **2** was defined as 1*R*,3*R*,4*S*,5*R*,9*R*,10*S*. Consequently, **2** was assigned as depicted and named trichotheciumone B, a 7,8-*seco*-7-*nor*-trichothecene.

Compound **3** was obtained as a white powder, displaying an exact mass of *m/z* 499.2519 [M + H]^+^ (calcd for C_25_H_39_O_10_, 499.2538) in HRESIMS, corresponding to a molecular formula of C_25_H_38_O_10_ with eight degrees of unsaturation. Analysis of the NMR data in Tables 1 and 4 indicated that **3** was deemed as a derivative of the trichothecenes. The ^13^C NMR spectrum displayed six characteristic signals indicative of a glycosyl unit (*δ*_C_ 61.8, 67.5, 70.9, 74.1, 75.6, 102.1). The mannose unit was further evidenced by comparison with the literature data (*δ*_C_ 61.2, 66.6, 71.1, 74.1, 75.9, 100.1) and *δ*_H_ 3.18 (brs), 3.53 (brs), 3.86 (m), 3.87 (brs), 3.97 (brs), and 4.44 (d, *J* = 0.7 Hz) in CDCl_3_ [20]. The mannosidic linkage of 1''-O-7 was revealed by a key HMBC correlation from H-1'' to C-7. The *β*-configuration of the mannose unit was confirmed by the following evidences. Two broad singlets of the anomeric proton H-1'' and H-2'', as well as the critical and obvious NOESY correlations of H-1'' with H-3'' and H-5'', provided compelling evidence for axial situation of anomeric proton H-1'', H-3'', and H-5'' and equatorial situation of H-2''. Based on the NOESY correlations of H_3_-13/H-3/H-6/H_3_-14 and analysis in light of biosynthetic origin, the relative configuration of chiral centers C-1, C-3, C-4, C-5, C-10, and C-11 was established. Meanwhile, the overall relative configuration of **3** was determined by interpretation of key NOESY correlations and GIAO ^13^C NMR calculations with the STS protocol performed for eight isomers (**3a**–3**h**) (Table S4). The absolute configuration of **3**, including the D-mannose moiety, was determined by ECD calculations using the time-dependent density functional theory (TDDFT) method. As shown in Fig. 4, the calculated ECD curve of **3a** (1*R*,3*R*,4*S*,5*R*,7*S*,8*R*,10*R*,11*S*-*β*-D-mannopyranoside) matched well with the experimental ECD curve. Therefore, the absolute configuration of **3** was assigned as 1*R*,3*R*,4*S*,5*R*,7*S*,8*R*,10*R*,11*S*-*β*-D-mannopyranoside, and it was named trichothecinoside A.

Compound **4** was obtained as a white powder, having the molecular formula C_16_H_24_O_5_ based on the protonated ion peak at *m/z* 297.1700 [M + H]^+^ (calcd for C_16_H_25_O_5_, 297.1697), corresponding to five degrees of unsaturation. Analysis of 1D data of **4** (Tables 1 and 4) revealed close resemblance to the co-isolated new natural product isocrotocol B (**11**) [21], indicating a shared trichothecene backbone. A key structural distinction was the presence of an oxygenated methyl group at C-7 in **4**. Further analysis of the 2D data confirmed that the remaining structure of **4** were the same as that of **11**. The absolute configuration was defined to be 1*R*,3*R*,4*S*,5*R*,6*R*,7*R*,10*R*,11*S* by comparison of the experimental ECD spectrum with calculated one (Fig. 4). Therefore, **4** was established as depicted and named trichothecrotocin T.

Compound **5** was obtained as a white powder, exhibiting the molecular formula C_27_H_32_O_7_, based on the protonated molecule peak at *m/z* 469.2211 [M + H]^+^ (calcd for C _27_H_33_O_7_, 469.2221), implying twelve degrees of unsaturation. Analysis of the 1D data (Tables 2 and 4) indicated a structure similar to that of co-isolated compound trichothecrotocin O (**16**), differing by the replacement of a methoxy group in **16** with a phenylacetoxy moiety at the C-7 position in **5**. The proposed structure was supported by ^1^H–^1^H COSY correlations for H-4''/H-5''/H-6''/H-7''/H-8'', as well as HMBC correlations from H-7 to C-1'' and from H_2_-2'' to C-1'', C-3'', C-4'', and C-8''. Key NOESY correlations of H_3_-13/H-3/H-10/H_3_-14 revealed the same relative configuration as **16**, although the stereochemistry at C-7 remained unassigned. To resolve this, GIAO ^13^C NMR calculations with the STS protocol were conducted for two diastereomers **5a** and **5b** (Table S9). The calculated data for **5a** matched the experimental data, thereby establishing the relative configuration. Furthermore, the agreement between experimental ECD spectrum and the calculated spectrum for **5** confirmed absolute configuration as 1*R*,3*R*,4*S*,5*R*,6*R*,7*R*,10*R*,11*S* (Fig. 4). Therefore, the structure of **5** was validated and assigned the name trichothecrotocin U.

**Table 2:** ^1^H NMR Spectroscopic Data for Compounds **5**−**8**

Position	**5**^*a*^	**6**^*a*^	**7**^*a*^	**8**^*b*^
	*δ*_H_, mult. (*J* = Hz)	*δ*_H_, mult. (*J* = Hz)	*δ*_H_, mult. (*J* = Hz)	*δ*_H_, mult. (*J* = Hz)
1	4.25, d (5.1)	3.95, d (5.3)	3.95, d (5.3)	3.90, d (5.4)
2	H_a_ 2.15, overlapped H_b_ 2.42, dd (15.6, 8.2)	H_a_ 2.07, ddd (15.5, 5.3, 3.6) H_b_ 2.59, dd (15.5, 7.7)	H_a_ 2.10, ddd (15.5, 5.3, 3.9) H_b_ 2.59, dd, (15.5, 7.9)	H_a_ 2.08, ddd (15.5, 5.4, 3.9) H_b_ 2.53, dd (15.5, 7.9)
3	5.51, dd (8.2, 3.9)	5.49, dd (7.7, 3.6)	5.51, dd (7.9, 3.9)	5.57, dd (7.9, 3.8)
6	3.67, overlapped	H_a_ 2.10, dd (12.5, 1.7) H_b_ 2.85, d (12.5)	H_a_ 2.09, overlapped H_b_ 2.11, overlapped	3.37, dd (4.2, 3.0)
7	5.21, s	–	–	3.16, dd (4.2, 1.9)
8	–	2.76, m	2.76, dp (13.1, 6.5)	–
9	5.70, d (5.5)	H_a_ 1.73, ddd (14.7, 13.2, 3.3) H_b_ 2.15, ddd (14.7, 6.4, 2.6)	H_a_ 1.73, m H_b_ 2.15, dt (6.6, 1.9)	5.72, dd (6.5, 1.9)
10	3.68, overlapped	3.60, brs	3.61, dd (6.6, 3.0)	3.81, dd (6.5, 3.0)
12	H_a_ 3.63, overlapped H_b_ 4.00, d (11.8)	H_a_ 2.83, d (4.1) H_b_ 3.19, d (4.1)	H_a_ 2.83, d (3.5) H_b_ 3.18, d (3.5)	H_a_ 3.00, d (3.5) H_b_ 3.21, d (3.5)
13	0.93, s	0.66, s	0.67, s	0.94, s
14	0.67, s	0.96, s	0.97, s	0.95, s
15	1.67, s	0.99, d (6.5)	0.99, d (1.7)	2.00, s
2'	5.80, dd (11.5, 1.9)	5.87, dq (15.5, 1.7)	5.82, dq (11.5, 1.8)	5.90, dq (15.6, 1.9)
3'	6.40, dq (11.5, 7.2)	7.00, dq (15.5, 6.9)	6.37, dq (11.5, 7.3)	7.02, dq (15.6, 6.8)
4'	2.17, dd (7.2, 1.9)	1.88, dd (6.9, 1.7)	2.15, dd (7.3, 1.8)	1.89, dd (6.8, 1.9)
2''	3.63, overlapped			
4''	7.25, overlapped			
5''	7.31, t (7.6)			
6''	7.25, overlapped			
7''	7.31, t (7.6)			
8''	7.25, overlapped			

^*a*^Recorded in CDCl_3_, 600 MHz

^*b*^Recorded in CDCl_3_, 500 MHz

Compounds **6** and **7** were each obtained as a white powder, presenting the same molecular formula C_19_H_26_O_5_, based on their HRESIMS data at *m/z* 335.1851 [M + H]^+^ (calcd for C_19_H_27_O_5_, 335.1853) and 335.1863 [M + H]^+^ (calcd for C_19_H_27_O_5_, 335.1853), respectively, corresponding to seven degrees of unsaturation. Analysis of 1D data for **6** and **7** (Tables 2 and 4) indicated close similarity to those of a new co-isolated natural product dihydrotrichothecolone (**12**), differing from **12** by the presence of the crotonyl moiety. The connectivity of this moiety was confirmed by the ^1^H–^1^H COSY data of H-2'/H-3'/H-4' and HMBC correlations from H-3 and H-2' to C-1' and from H-4' to C-2' and C-3'. The relative configuration of **6** was deemed as being identical to that of **12** through key NOESY correlations as depicted in Fig. 2. The *α*-orientation of the C-15 was assigned by comparing experimental data with GIAO ^13^C NMR calculations (STS protocol) performed for two diastereomers **6a** and **6b** (Table S11). A key distinction between **6** and **7** is the geometry of crotonyl group, which was ascertained as *Z* in **7**, assigned based on the coupling constant (*J* = 11.4 Hz) between H-2' and H-3'. The relative stereochemistry of **7** was elucidated using the same procedure as for **6**. Finally, the absolute configurations of both** 6** and **7** were defined to be 1*R*,3*R*,4*S*,5*R*,8*R*,10*R*,11*S* by comparing their experimental and calculated ECD spectra (Fig. 4). Consequently, compounds **6** and **7** were named trichothecrotocin V and trichothecrotocin W, respectively.

Compound **8** was obtained as a white powder, possessing the molecular formula C_19_H_24_O_5_ based on the proton adduct at *m/z* 333.1708 [M + H]^+^ (calcd for C_19_H_25_O_5_, 333.1697) in its HRESIMS, indicating eight degrees of unsaturation. The 1D (Tables 2 and 4) and 2D data disclosed that the structure of **8** was structurally similar to that of a co-isolated known compound crotocin (**22**), differing only in the *E* geometry of the crotonyl group. The geometric distinction between **8** and **22** paralleled that observed between **6** and **7**, as supported by the large coupling constant (*J* = 15.6 Hz) between H-2' and H-3' in **8**. NOESY correlations of H_3_-13/H-3/H-10/H_3_-14/H-6 suggested their co-facial orientation, which facilitated the determination of the relative configuration. The calculated ECD curve of 1*R*,3*R*,4*S*,5*R*,6*R*,7*S*,10*R*,11*S* was in good agreement with the experimental data (Fig. 4). Thus, **8** was assigned as depicted and named trichothecrotocin X.

Compounds **9** and **10** were each obtained as a white powder. Compound **9** possessed a molecular formula of C_15_H_20_O_3_ based on the proton adduct in its HRESIMS at *m/z* 249.1482 [M + H]^+^ (calcd for C_15_H_21_O_3_, 249.1485), corresponding to six degrees of unsaturation. The molecular formula for **10** was determined to be C_19_H_24_O_5_ by HRESIMS based on the sodium adduct at *m/z* 339.1563 [M + H]^+^ (calcd for C_19_H_24_O_5_Na, 339.1567), corresponding to eight degrees of unsaturation. Analysis of 1D data for both compounds **9** and **10** (Tables 3 and 4) exhibited close similarity to those of a co-isolated known compound 7-dehydro-8-dehydroxytrichothecinol B (**24**). For compound **9**, the key structural distinction was the absence of a crotonyl group at C-3. Comprehensive analysis of 2D data confirmed that the remaining planar structure and the relative configuration of **9** were identical to those of **24**. The calculated ECD curve for isomer **9a** (1*R*,3*R*,4*S*,5*R*,10*R*,11*S*) showed excellent agreement with the experimental data (Fig. 4). For compound **10**, the key structural distinction was only in the *E* geometry of the crotonyl group, supported by HMBC correlations from H-3 to C-1' and from H-4' to C-2' and C-3' as well as the large coupling constant (*J* = 15.5 Hz) between C-2′ and C-3′, confirming crotonyl group with *trans*-olefinic double bond. NOESY correlations observed for **10** were similar to those of **9**, suggesting an identical relative configuration. Furthermore, the experimental ECD spectrum of **10** (Fig. 4) closely matched that of **9** and was consistent with the calculated spectrum for isomer **10a** (1*R*,3*R*,4*S*,5*R*,10*R*,11*S*). Therefore, **9** and **10** were named trichothecrotocin Y and trichothecrotocin Z, respectively.

**Table 3:** ^1^H NMR Spectroscopic Data for Compounds **9**−**13**

Position	**9**^*a*^	**10**^*b*^	**11**^*c*^	**12**^*d*^	**13**^*c*^
	*δ*_H_, mult. (*J* = Hz)	*δ*_H_, mult. (*J* = Hz)	*δ*_H_, mult. (*J* = Hz)	*δ*_H_, mult. (*J* = Hz)	*δ*_H_, mult. (*J* = Hz)
1	3.76, d (5.4)	3.57, overlapped	4.27, d (5.4)	3.83, d (5.1)	3.85, d (5.3)
2	H_a_ 1.93, ddd (15.7,5.5, 3.2) H_b_ 2.65, dd (15.7, 7.5)	H_a_ 1.84, overlapped H_b_ 2.50, overlapped	H_a_ 2.10, ddd (15.7, 5.4, 3.1) H_b_ 2.47, dd (15.6, 7.7)	H_a_ 1.91, ddd (15.2, 5.1, 3.6) H_b_ 2.52, dd (15.2, 7.6)	H_a_ 2.04, ddd (15.5, 5.3, 3.7) H_b_ 2.54, dd (15.5, 7.9)
3	4.30, dd (7.5, 3.2)	5.50, dd (7.9, 3.8)	4.30, d (7.7)	4.31, dd (7.6, 3.6)	5.59, dd (7.9, 3.7)
6	5.85, brs	5.95, dd (9.8, 1.5)	3.93, brs	H_a_ 2.03, dd (12.6, 1.6) H_b_ 2.95, d (12.6)	1.88, m
7	5.85, brs	5.78, dd (9.8, 1.9)	4.03, brs	–	4.07, t (8.3)
8	–	–	–	2.77, m	–
9	5.54, d (6.1)	5.47, d (6.2)	5.62, d (5.8)	H_a_ 1.70, ddd (14.6, 13.2, 3.3) H_b_ 2.09, ddd (14.6, 6.4, 2.6)	5.49, m
10	3.63, d (6.1)	3.74, dd (6.2, 1.9)	3.56, d (5.8)	3.57, brs	3.67, d (5.5)
11	–	–	–	–	–
12	H_a_ 2.85, d (3.7) H_b_ 2.98 d (3.7)	H_a_ 2.83, d (4.0) H_a_ 2.92, d (4.0)	H_a_ 3.71, d (11.5) H_b_ 3.97, d (11.5)	H_a_ 2.80, d (4.0) H_b_ 3.09, d (4.0)	H_a_ 2.85, d (4.0) H_b_ 3.12, d (4.0)
13	0.93 s	0.73, s	1.20, s	0.71, s	0.74, s
14	0.91, s	0.88, s	0.87, s	0.86, s	1.00, s
15	1.83, s	1.75, d (1.5)	1.88, s	0.93, d (6.5)	1.83, s
2′		5.90, dd (15.5, 1.8)			5.83, dd (11.4, 1.8)
3′		6.91, dq (15.5, 6.9)			6.36, dq (11.4, 7.3)
4′		1.87, dd (6.9, 1.8)			2.15, dd (7.3, 1.8)

^*a*^Recorded in CDCl_3_,500 MHz

^*b*^Recorded in dimethyl sulfoxide-*d*_6_, 600 MHz

^*c*^Recorded in CDCl_3_, 600 MHz

^*d*^Recorded in methanol-*d*_4_, 600 MHz

**Table 4:** ^13^C NMR Spectroscopic Data for Compounds **1**−**13**

Position	**1**^*a*^	**2**^*a*^	**3**^*a*^	**4**^*a*^	**5**^*a*^	**6**^*a*^	**7**^*a*^	**8**^*b*^	**9**^*b*^	**10**^*c*^	**11**^*a*^	**12**^*d*^	**13**^*a*^
	*δ*_C_, type	*δ*_C_, type	*δ*_C_, type	*δ*_C_, type	*δ*_C_, type	*δ*_C_, type	*δ*_C_, type	*δ*_C_, type	*δ*_C_, type	*δ*_C_, type	*δ*_C_, type	*δ*_C_, type	*δ*_C_, type
1	81.7 CH	82.6 CH	79.2 CH	82.5 CH	82.4 CH	79.7 CH	79.8 CH	79.2 CH	77.7 CH	76.8 CH	82.6 CH	81.2 CH	79.3 CH
2	36.5 CH_2_	36.4 CH_2_	37.0 CH_2_	40.1 CH_2_	37.1 CH_2_	37.2 CH_2_	37.4 CH_2_	36.6 CH_2_	40.2 CH_2_	36.3 CH_2_	40.1 CH_2_	37.9 CH_2_	36.8 CH_2_
3	75.3 CH	74.5 CH	74.0 CH	74.0 CH	74.3 CH	73.9 CH	73.8 CH	74.5 CH	73.8 CH	74.2 CH	74.0 CH	73.6 CH	73.8 CH
4	53.4 C	50.0 C	49.5 C	49.6 C	49.7 C	49.3 C	49.3 C	50.7 C	47.8 C	47.4 C	49.6 C	50.4 C	49.1 C
5	43.1 C	50.8 C	40.5 C	38.3 C	38.5 C	48.2 C	48.3 C	41.7 C	43.7 C	43.8 C	38.3 C	50.0 C	42.4 C
6	39.4 CH_2_	171.1 C	34.4 CH_2_	72.3 CH	74.1 CH	45.1 CH_2_	45.2 CH_2_	59.2 CH	133.0 CH	133.1 CH	77.0 CH	46.2 CH_2_	35.1 CH_2_
7	173.6 C	205.5 C	80.7 CH	81.8 CH	72.3 CH	212.1 C	212.2 C	47.4 CH	126.2 CH	125.2 CH	72.4 CH	215.3 C	69.0 CH
8	206.5 C	44.2 CH_2_	29.4 CH	136.3 C	133.6 C	39.4 CH	39.5 CH	137.3 C	135.4 C	133.8 C	136.5 CH	40.4 CH	142.3 C
9	42.0 CH_2_	71.3 CH	30.1 CH_2_	121.1 CH	123.5 CH	36.6 CH_2_	36.8 CH_2_	123.0 CH	117.3 CH	117.5 CH	121.5 CH	37.9 CH_2_	121.1 CH
10	72.3 CH	75.8 C	72.4 CH	69.4 CH	69.3 CH	71.5 CH	71.7 CH	70.1 CH	69.9 CH	69.1 CH	69.3 CH	72.6 CH	70.4 C
11	76.5 C	70.7 CH_2_	65.7 C	75.1 C	74.5 C	65.6 C	65.8 C	65.8 C	66.6 C	66.5 C	75.1 C	66.8 C	65.4 C
12	66.8 CH_2_	10.6 CH_3_	48.4 CH_2_	66.5 CH_2_	66.5 CH_2_	47.9 CH_2_	48.1 CH_2_	48.3 CH_2_	46.6 CH_2_	45.8 CH_2_	66.5 CH_2_	48.1 CH_2_	47.8 CH_2_
13	7.4 CH_3_	15.1 CH_3_	5.8 CH_3_	6.9 CH_3_	6.4 CH_3_	5.7 CH_3_	5.9 CH_3_	6.7 CH_3_	6.3 CH_3_	5.6 CH_3_	6.8 CH_3_	6.3 CH_3_	6.0 CH_3_
14	20.6 CH_3_	31.2 CH_3_	20.5 CH_3_	15.0 CH_3_	15.0 CH_3_	18.8 CH_3_	18.9 CH_3_	16.3 CH_3_	16.5 CH_3_	16.3 CH_3_	15.5 CH_3_	18.9 CH_3_	16.9 CH_3_
15	31.5 CH_3_		17.7 CH_3_	20.5 CH_3_	20.2 CH_3_	13.7 CH_3_	13.8 CH_3_	22.0 CH_3_	21.2 CH_3_	20.7 CH_3_	20.5 CH_3_	14.1 CH_3_	18.8 CH_3_
16				58.9 CH_3_									
1′	164.8 C	165.1 C	166.4 C		165.7 C	166.2 C	166.4 C	166.3 C		165.3 C			166.4 C
2′	119.3 CH	119.5 CH	120.7 CH		120.1 CH	122.5 CH	120.6 CH	122.5 CH		122.2 CH			120.5 CH
3′	147.9 CH	147.6 CH	145.5 CH		146.6 CH	145.5 CH	146.1 CH	145.5 CH		145.6 CH			145.9 CH
4′	15.6 CH_3_	15.6 CH_3_	15.5 CH_3_		15.5 CH_3_	18.1 CH_3_	15.6 CH_3_	18.1 CH_3_		17.7 CH_3_			15.5 CH_3_
1″			102.1 CH		170.6 C								
2″			70.9 CH		41.7 CH_2_								
3″			74.1 CH		133.6 C								
4″			67.5 CH		129.2 CH								
5″			75.6 CH		128.7 CH								
6″			61.8 CH_2_		123.5 CH								
7″					128.7 CH								
8″					129.2 CH								

^*a*^Recorded in CDCl_3_, 150 MHz

^*b*^Recorded in CDCl_3_,125 MHz

^*c*^Recorded in dimethyl sulfoxide-*d*_6_, 150 MHz

^*d*^Recorded in methanol-*d*_4_, 150 MHz

Compounds **11**, **12**, and *epi*-trichothecinol B (**13**) were isolated from this fungus as new natural products for the first time. The absolute configuration of compound **11** was first determined and the ECD spectra of **13** was provided in Figure S143. The known compounds **14**–**26** were characterized via analysis of their HRESIMS and 1D data and comparison with literature data. They were identified as trichothecrotocin N (**14**) [12], trichothecene analogue (**15**) [19], trichothecrotocin O (**16**) [12], trichothecrotocin Q (**17**) [12], trichothecolone (**18**) [22], trichodermol (**19**) [23], 8-deoxy-trichothecin (**20**) [22], trichodermin (**21**) [24], crotocin (**22**) [21], crotocol (**23**) [21], 7-dehydro-8-dehydroxytrichothecinol B (**24**) [25], cyclonerodiol (**25**) [26], and cyclonerodiol C (**26**) [27].

**Scheme 1:**
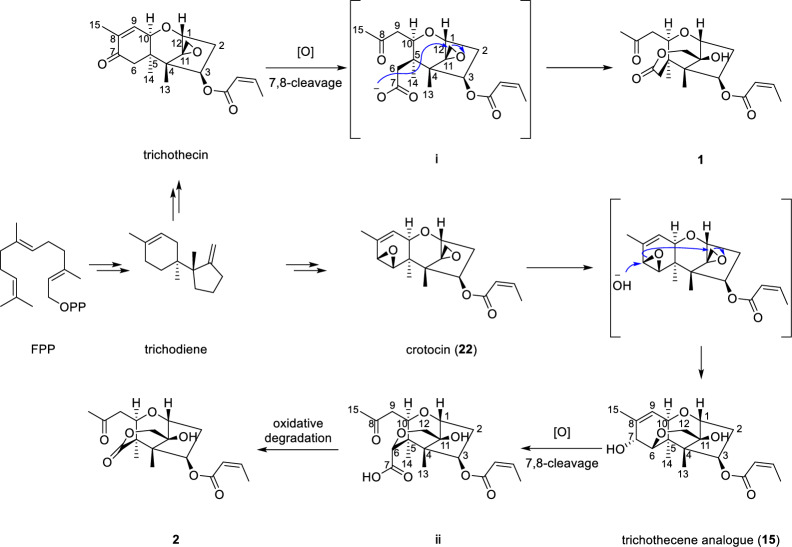
Proposed biogenetic pathway for compounds **1** and **2**

Trichotheciumone A (**1**) and B (**2**) possess unusual sesquiterpenoid skeletons based on the trichothecene core. Although structurally distinct, compounds **1** and **2** are likely biosynthesized from the same precursor, trichodiene, a key intermediate in trichothecene biosynthesis [4, 28]. A putative biosynthetic pathway for **1** and **2** is proposed in Scheme 1. The pathway suggests that cleavage of the C-7–C-8 bond in trichothecin, followed by oxidation, would yield the crucial intermediate **ⅰ**, which belongs to the rare 7,8-*seco*-trichothecene class. A subsequent nucleophilic attack of the carboxylate anion on the C-12 epoxide would form compound **1**, representing the first reported trichothecene-type sesquiterpenoid with an opened A-ring. Alternatively, crotocin (**22**) could be converted to intermediate **15** via nucleophilic ring-opening of the 6,7-epoxide by a hydroxide ion. Further cleavage of the C-7–C-8 bond in **15**, followed by oxidation, would lead to intermediate **ⅱ**. The oxidative decarboxylation of **ⅱ** would then yield compound **2**, which is the first reported example of a rare A-ring-*seco*-*nor*-trichothecene-type sesquiterpenoid.

**Fig. 5:**
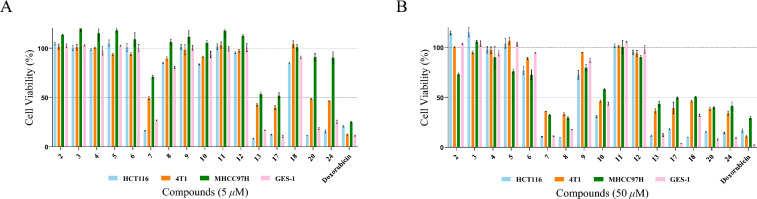
Antiproliferative activity against four cell lines. **A**, **B** Cytotoxicity screening of isolated compounds with concentrations 5 μM and 50 μM against four cell lines (HCT116, 4T1, MHCC97H, and GES-1) determined by CCK-8 assay (doxorubicin as positive control)

**Fig. 6:**
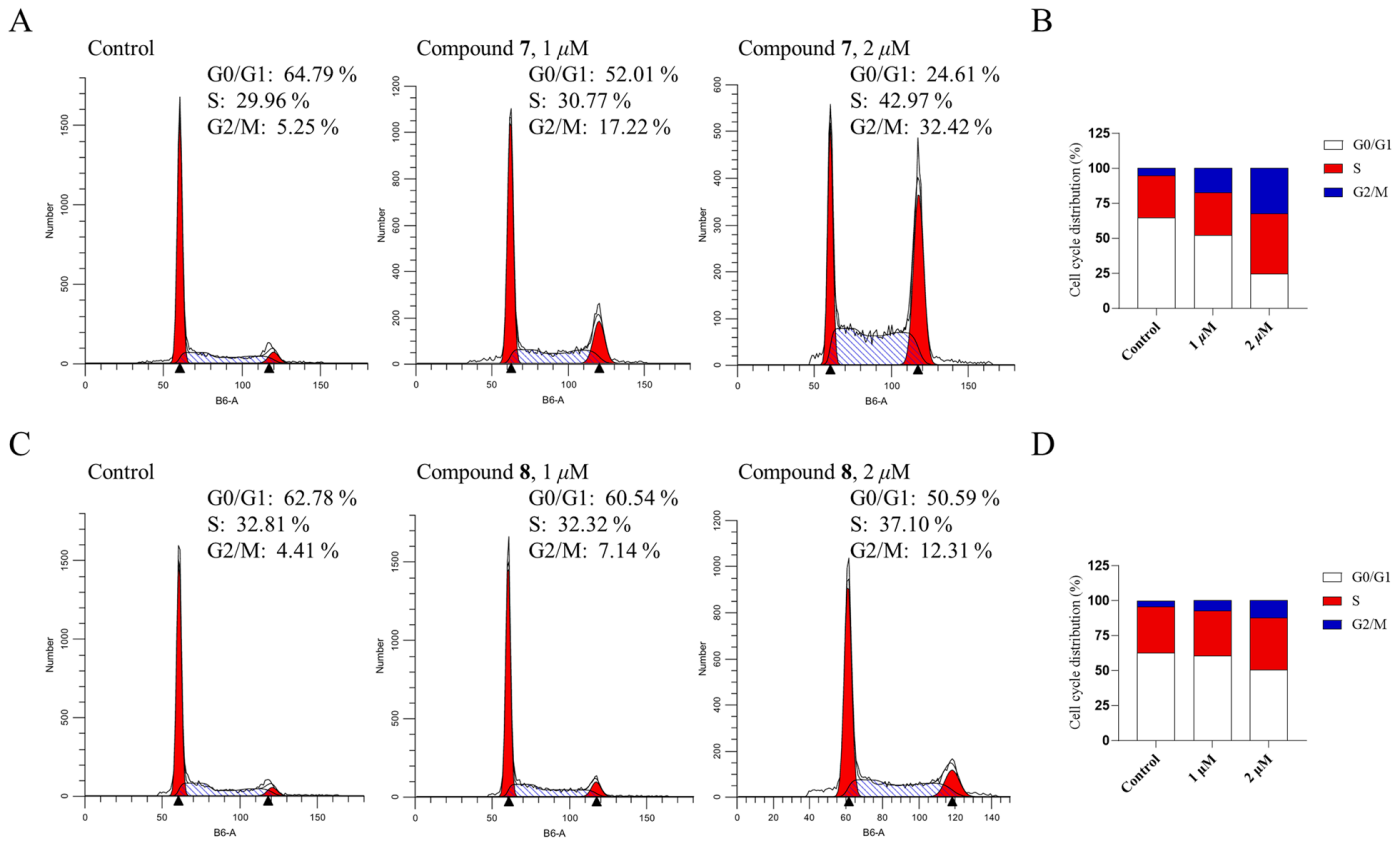
Cell cycle analysis of HCT116 cells after treatment with compounds **7** and **8**. **A**, **C** Histograms of cell cycle distribution after treatment with 0, 1 and 2 μM of compounds **7** and **8** for 24 h, respectively. **B**, **D** Percentage of HCT116 cell in G0/G1, S and G2/M phases after treatment with compounds **7** and **8**, respectively. Data are presented as mean ± SD (n = 3)

The cytotoxic activities of the isolated compounds were evaluated against human colon carcinoma HCT116 cell line, mouse breast cancer 4T1 cell line, and human liver cancer MHCC97H cell line, with one strain of human gastric epithelial GES-1 cells serving as a normal cell lines control, using the CCK-8 method. Seven compounds (**7**, **8**, **10**, **13**, **17**, **18**, **20**, **24**) exhibited significant anti-proliferative activity (Fig. 5A, B). Among them, compound **17** was the most potent compound with IC_50_ of 0.35 μM, 0.52 μM, 0.57 μM against HCT116, 4T1, and MHCC97H cells respectively (Table 5). Cell cycle assays revealed that new compounds **7** and **8** arrested HCT116 cells at the G2/M phase. Treatment with 0, 1, and 2 μM of compound **7** increased the G2/M population compared with the control (control: 5.25%, 1 μM: 17.22%, 2 μM: 32.42%) and decreased G0/G1 population (control: 64.79%, 1 μM: 52.01%, 2 μM: 24.61%) (Fig. 6A, B). Treatment with 0, 1, and 2 μM of compounds **8** increased the G2/M population compared with the control (control: 4.41%, 1 μM: 7.14%, 2 μM: 12.31%) and decreased G0/G1 population (control: 62.78%, 1 μM: 60.54%, 2 μM: 50.59%) (Fig. 6C, D). However, the test compounds exhibited no observable selectivity in inhibiting proliferation between these three cancer cell lines and GES-1 cell line. Structure–activity relationship (SAR) analysis indicated that the configuration of the C-3 crotonyl group influences toxicity. Specifically, changing the crotonyl group at C-3 from the *Z*-configuration to the *E*-configuration results in decreased toxicity, a *Z*-configuration (as in compounds **7** and **24**) conferred greater potency than an *E*-configuration (as in compounds **6** and **10**).

**Table 5:** Cytotoxic activities of compounds **7**, **8**, **10**, **13**, **17**, **18**, **20**, and **24**^*a*^

Compound ^*b*^	HCT116	4T1	MHCC97H
**7**	1.89 ± 0.04	2.75 ± 0.05	3.09 ± 0.08
**8**	6.39 ± 0.74	7.85 ± 0.26	7.63 ± 0.59
**13**	1.55 ± 0.07	1.35 ± 0.16	2.30 ± 0.09
**17**	0.35 ± 0.01	0.52 ± 0.19	0.57 ± 0.04
**18**	13.53 ± 0.97	17.26 ± 1.52	> 40
**20**	1.90 ± 0.05	2.16 ± 0.11	3.99 ± 0.06
**24**	2.21 ± 0.05	3.04 ± 0.11	4.36 ± 0.27
Doxorubicin	0.19 ± 0.01	1.13 ± 0.21	0.77 ± 0.05

^*a*^IC_50_, *μ*M, mean ± SD, n = 3

^*b*^IC_50_ values of compound **10** were all higher than 40 μM

## Experimental section

### General experimental procedures

Optical rotations were measured on a Rudolph Research Analytical Autopol IV automatic polarimeter. UV spectra were recorded using a Cary 300 spectrometer (Agilent Technologies, Santa Clara, CA, USA). IR spectra were recorded on a Shimadzu Fourier Transform Infrared Spectrometer using KBr pellets. Circular Dichroism (CD) spectra were obtained on a Chirascan™-plus Circular Dichroism spectrometer. High-resolution electrospray ionization mass spectrometry (HRESIMS) spectra were acquired with an Agilent 6500 series Q-TOF mass spectrometer (Agilent Technologies, Singapore). NMR spectra were recorded on Bruker Avance III HD 600 MHz and 500 MHz spectrometer with tetramethylsilane (TMS) serving as the internal standard. Column chromatography (CC) was performed using macro-porous adsorbent resin D101 (Tianjin Haoju Resin Technology Co., Ltd., Tianjin, China), silica gel (200–300 mesh, Qingdao Marine Chemical, Qingdao, China), and Sephadex LH-20 (GE Healthcare, Uppsala, Sweden). MCI GEL™ (Mitsubishi Chemical Corporation, Japan). Semipreparative ZORBAX SB-C18 (Agilent, 5 μm, 9.4 mm × 150 mm), Supersil ODS-B (Elite, 10 μm, 20.0 mm × 250 mm), Supersil ODS2 (Elite, 10 μm, 20.0 mm × 250 mm), Ultimate XB-CN (Welch, 5 μm, 10.0 mm × 250 mm) and preparative SinoPark C18 (Elite, 10 μm, 20.0 mm × 250 mm) were analyzed by an EClassical P3500 prep-HPLC system and a DAD detector (EClassical P3500, Dalian Elite Analytical Instruments Co., Ltd., Dalian, China). The progress of the experiments was monitored by pre-coated silica gel GF254 plates (Qingdao Marine Chemical). Spots were visualized under UV light (254 or 365 nm) or by spraying with 10% H_2_SO_4_ in EtOH followed by heating. All other chemicals used in this study were of analytical grade.

### Fungal material

The fungal strain *Trichothecium* sp. DWS815 was isolated from a forest soil sample collected from Daweishan National Forest Park, Hunan Province. The strain was identified as *Trichothecium* sp. (GenBank Accession No. ON386019.1) based on their ITS sequences and the phylogenetic analysis (Tables S24 and S25). A voucher specimen of this strain has been deposited at the Xiangya School of Pharmaceutical Sciences, Central South University, Changsha, China.

### Fermentation, extraction and isolation

This fungus was initially cultivated on Sabouraud Dextrose Agar (SDA) medium (containing 20 g/L dextrose, 10 g/L peptone, 1 g/L yeast extract powder, 15 g/L agar and water) for 8 days at 25 °C. Subsequently, the mycelia on agar plugs were cut and transferred to the sterile rice fermentation medium. The medium was prepared with 100 g of rice and 80 mL of water in 500 mL Erlenmeyer flasks. For large-scale fermentation, a total of 14 kg of rice was distributed into 139 flasks and inoculated with this fungus. The fermentation proceeded at 25 °C in the dark for 21 days.

The rice was extracted with ethyl acetate at room temperature 10 times, and the solvent was evaporated under vacuum to yield an extract (170 g). The extract was absorbed onto 500 g macro-porous adsorbent resin D101 and eluted stepwise with water, 80% aqueous MeOH, and 100% MeOH. The 80% aqueous MeOH fraction was collected and condensed to yield 26 g residue. The 80% aqueous MeOH fraction was then fractionated using silica gel CC eluted with a step gradient of Petroleum ether–EtOAc (v/v, 100:0, 20:1, 15:1, 10:1, 5:1, 2:1, 1:1) and EtOAc–MeOH (v/v, 100:0, 10:1, 5:1, 1:1, 0:100) to provide nine fractions (A∼I).

The Fr.A was subjected to MCI to give five subfractions (Fr.A1∼Fr.A5). Fr.A2 was separated using semipreparative Supersil ODS-B (CH_3_CN: H_2_O, 55:45) to afford compound **24** (*t*_R_ = 20.9 min, 22.5 mg) and Fr.A2-1. Fr.A2-1 was fractionated using Supersil ODS-B (MeOH: H_2_O, 57:43) to afford compounds **7** (*t*_R_ = 36.9 min, 3.5 mg) and **21** (*t*_R_ = 40.1 min, 1.9 mg). Fr.A4 was separated by semipreparative ZORBAX SB-C18 (CH_3_CN: H_2_O, 49:51) to obtain compound **20** (*t*_R_ = 23.9 min, 50.2 mg). Fr.B was subjected to Sephadex LH-20 (MeOH) to give five subfractions (Fr.B1∼Fr.B5). Fr.B3 was isolated on the preparative SinoPark C18 (MeOH: H_2_O, 62:38) to obtain four fractions (Fr.B3-1∼Fr.B3-4). Fr.B3-1 was purified using semipreparative Supersil ODS2 (CH_3_CN: H_2_O, 28:72) to afford compounds **1** (*t*_R_ = 21.1 min, 0.3 mg) and **2** (*t*_R_ = 29.2 min, 0.6 mg). Fr.B3-4 was purified using semipreparative Supersil ODS-B (MeOH: H_2_O, 53:47) to yield compound **17** (*t*_R_ = 47.0 min, 24.5 mg). Fr.C was applied to semipreparative ZORBAX SB-C18 with a step gradient of CH_3_CN-H_2_O (v/v, from 20:100 to 50:50 in 35 min, flow speed: 3 mL/min) to afford compounds **22** (*t*_R_ = 19.8 min, 1.6 g), **16** (*t*_R_ = 17.7 min, 4.9 mg), **8** (*t*_R_ = 19.4 min, 6.8 mg), and **5** (*t*_R_ = 32.8 min, 6.58 mg). Fr.D was subjected to Sephadex LH-20 (MeOH) and then was further fractionated using semipreparative Ultimate XB-CN (CH_3_CN: H_2_O, 19:81) to give **19** (*t*_R_ = 14.6 min, 1.1 mg), **18** (*t*_R_ = 9.5 min, 1.9 mg), **9** (*t*_R_ = 12.3 min, 1.5 mg), **13** (*t*_R_ = 28.9 min, 8.4 mg), **26** (*t*_R_ = 20.0 min, 3.5 mg), **25** (*t*_R_ = 24.9 min, 9.9 mg), and **12** (*t*_R_ = 17.0 min, 2.3 mg). Fr.E was subjected to Sephadex LH-20 (MeOH) and then was purified using semipreparative Supersil ODS-B (MeOH: H_2_O, 26:74) to afford **14** (*t*_R_ = 15.9 min, 1.4 mg), **15** (*t*_R_ = 12.2 min, 41.9 mg), **4** (*t*_R_ = 10.7 min, 3.09 mg), **23** (*t*_R_ = 23.5 min, 3.3 mg), and **11** (*t*_R_ = 8.7 min, 1.6 mg). Fr.F was subjected to Sephadex LH-20 (MeOH) and then was purified using semipreparative ZORBAX SB-C18 with a step gradient of CH_3_CN–H_2_O (v/v, from 5:95 to 21:79 in 100 min, flow speed: 3 mL/min) to yield **3** (*t*_R_ = 81.6 min, 1.3 mg). Fr.I was also chromatographed on Sephadex LH-20 (MeOH) and purified using semipreparative Ultimate XB-CN with a step gradient of CH_3_CN–H_2_O (v/v, from 25:75 to 45:55 in 40 min, flow speed: 3 mL/min) to give compounds **6** (*t*_R_ = 29.8 min, 0.5 mg) and **10** (*t*_R_ = 35.1 min, 1.9 mg).

*Trichotheciumone A (****1****)*. White powder; $[\alpha]^{25}_{\text D}$+ 37.5 (*c* 0.04, MeOH); UV (MeOH) *λ*_max_ (log *ε*): 210 (3.48) nm; IR (KBr) *ν*_max_: 3442, 2979, 2950, 2923, 1714, 1647, 1294, 1178, 1115, 1028, 817 cm^−1^; CD (*c* 1.19 mM, MeOH): 205 nm (Δ*ε* –4.84), 227 nm (Δ*ε* + 4.38), 269 nm (Δ*ε* –1.91); for ^13^C NMR (CDCl_3_, 150 MHz) and ^1^H NMR (CDCl_3_, 600 MHz) data, see Tables 1 and 4; HRESIMS (positive) *m/z* 367.1758 [M + H]^+^ (calcd for C_19_H_27_O_7_, 367.1751, Δ + 1.9064 ppm).

*Trichotheciumone B (****2****)*. White powder; $[\alpha]^{25}_{\text D}$ + 264.86 (*c* 0.037, MeOH); UV (MeOH) *λ*_max_ (log *ε*): 210 (2.88) nm; IR (KBr) *ν*_max_: 3434, 2924, 2855, 2788, 1718, 1578, 1435, 1384, 1180 cm^−1^; CD (*c* 1.05 mM, MeOH): 200 nm (Δ*ε* + 5.29), 226 nm (Δ*ε* –2.83), 270 nm (Δ*ε* –0.51); for ^13^C NMR (CDCl_3_, 150 MHz) and ^1^H NMR (CDCl_3_, 600 MHz) data, see Tables 1 and 4; HRESIMS (positive) *m/z* 353.1599 [M + H]^+^ (calcd for C_18_H_25_O_7_, 353.1595, Δ + 1.1326 ppm).

*Trichothecinoside A (****3****)*. White powder; $[\alpha]^{25}_{\text D}$ –2.0 (*c* 0.05, MeOH); UV (MeOH) *λ*_max_ (log *ε*): 200 (3.17) nm; IR (KBr) *ν*_max_: 3420, 2962, 2935, 2876, 1715, 1649, 1383, 1291, 1184, 1083, 1027, 959 cm^−1^; CD (*c* 1.00 mM, MeOH): 217 nm (Δ*ε* –7.13); for ^13^C NMR (CDCl_3_, 150 MHz) and ^1^H NMR (CDCl_3_, 600 MHz) data, see Tables 1 and 4; HRESIMS (positive) *m/z* 499.2519 [M + H]^+^ (calcd for C_25_H_39_O_10_, 499.2538 Δ –3.8057 ppm).

*Trichothecrotocin T (****4****)*. White powder; $[\alpha]^{25}_{\text D}$ –12.5 (*c* 0.037, MeOH); UV (MeOH) *λ*_max_ (log *ε*): 200 (2.82) nm; IR (KBr) *ν*_max_: 3459, 3371, 2981, 2932, 2859, 1628, 1457, 1135, 1125, 1098, 1051 cm^−1^; CD (*c* 1.35 mM, MeOH): 200 nm (Δ*ε* –28.13); for ^13^C NMR (CDCl_3_, 150 MHz) and ^1^H NMR (CDCl_3_, 600 MHz) data, see Tables 1 and 4; HRESIMS (positive) *m/z* 297.1700 [M + H]^+^ (calcd for C_16_H_25_O_5_, 297.1697, Δ + 1.0009 ppm).

*Trichothecrotocin U (****5****)*. White powder; $[\alpha]^{25}_{\text D}$ –10.9 (*c* 0.064, MeOH); UV (MeOH) *λ*_max_ (log *ε*): 200 (3.21) nm; IR (KBr) *ν*_max_: 3434, 2978, 2946, 2922, 2859, 1737, 1714, 1647, 1436, 1248, 1179, 1133, 1049 cm^−1^; CD (*c* 1.37 mM, MeOH): 200 nm (Δ*ε* –30.89), 210 nm (Δ*ε* + 0.19), 225 nm (Δ*ε* –8.52); for ^13^C NMR (CDCl_3_, 150 MHz) and ^1^H NMR (CDCl_3_, 600 MHz) data, see Tables 2 and 4; HRESIMS (positive) *m/z* 469.2211 [M + H]^+^ (calcd for C _27_H_33_O_7_, 469.2221, Δ –2.1312 ppm).

*Trichothecrotocin V (****6****)*. White powder; $[\alpha]^{25}_{\text D}$+ 12.5 (*c* 0.008, MeOH); UV (MeOH)* λ*_max_ (log *ε*): 210 (2.94) nm; IR (KBr) *ν*_max_: 2971, 2928, 2855, 1717, 1576, 1445, 1384, 1183, 1081, 971 cm^−1^; CD (*c* 1.50 mM, MeOH): 208 nm (Δ*ε* –11.61), 291 nm (Δ*ε* + 0.92); for ^13^C NMR (CDCl_3_, 150 MHz) and ^1^H NMR (CDCl_3_, 600 MHz) data, see Tables 2 and 4; HRESIMS (positive) *m/z* 335.1851 [M + H]^+^ (calcd for C_19_H_27_O_5_, 335.1853, Δ –0.5967 ppm).

*Trichothecrotocin W (****7****)*. white powder; $[\alpha]^{25}_{\text D}$ –6.98 (*c* 0.043, MeOH); UV (MeOH) *λ*_max_ (log *ε*): 210 (3.08) nm; IR (KBr) *ν*_max_: 2959, 2928, 1716, 1647, 1466, 1366, 1287, 1183, 1081, 966, 816 cm^−1^; CD (*c* 1.29 mM, MeOH): 213 nm (Δ*ε* –4.32), 293 nm (Δ*ε* + 1.46); for ^13^C NMR (CDCl_3_, 150 MHz) and ^1^H NMR (CDCl_3_, 600 MHz) data, see Tables 2 and 4; HRESIMS (positive) *m/z* 335.1863 [M + H]^+^ (calcd for C_19_H_27_O_5_, 335.1853, Δ + 2.9834 ppm).

*Trichothecrotocin X (****8****)*. White powder; $[\alpha]^{25}_{\text D}$ –16.67 (*c* 0.024, MeOH); UV (MeOH) *λ*_max_ (log *ε*): 208 (3.29) nm; IR (KBr) *ν*_max_: 2965, 2925, 2857, 1716, 1602, 1384, 1185, 1083, 969 cm^−1^; CD (*c* 0.72 mM, MeOH): 203 nm (Δ*ε* –13.10), 230 nm (Δ*ε* + 0.50); for ^13^C NMR (CDCl_3_, 125 MHz) and ^1^H NMR (CDCl_3_, 500 MHz) data, see Tables 2 and 4; HRESIMS (positive) *m/z* 333.1708 [M + H]^+^ (calcd for C_19_H_25_O_5_, 333.1697, Δ + 3.3016 ppm).

*Trichothecrotocin Y (****9****)*. White powder; $[\alpha]^{25}_{\text D}$ –51.35 (*c* 0.037, MeOH); UV (MeOH) *λ*_max_ (log *ε*): 200 (2.60) nm; IR (KBr) *ν*_max_: 3452, 2965, 2923, 2852, 1664, 1623, 1593, 1447, 1422, 1281, 1185, 1074, 955 cm^−1^; CD (*c* 1.49 mM, MeOH): 256 nm (Δ*ε* –43.19); for ^13^C NMR (CDCl_3_, 125 MHz) and ^1^H NMR (CDCl_3_, 500 MHz) data, see Tables 3 and 4; HRESIMS (positive) *m/z* 249.1482 [M + H]^+^ (calcd for C_15_H_21_O_3_, 249.1485, Δ –1.2041 ppm).

*Trichothecrotocin Z (****10****)*. White powder; $[\alpha]^{25}_{\text D}$ –63.16 (*c* 0.019, MeOH); UV (MeOH) *λ*_max_ (log *ε*): 210 (3.15) nm; IR (KBr) *ν*_max_: 2917, 2852, 1732, 1717, 1606, 1590, 1384, 1189, 1101, 1082, 1064, 1028, 957 cm^−1^; CD (*c* 0.60 mM, MeOH): 226 nm (Δ*ε* –13.12); for ^13^C NMR (dimethyl sulfoxide-*d*_6_, 150 MHz) and ^1^H NMR (dimethyl sulfoxide-*d*_6_, 600 MHz) data, see Tables 3 and 4; HRESIMS (positive) *m/z* 339.1563 [M + H]^+^ (calcd for C_19_H_24_O_5_Na, 339.1567, Δ –1.1793 ppm).

*Isocrotocol B (****11****)*. White powder; $[\alpha]^{25}_{\text D}$ –18.0 (*c* 0.05, MeOH); UV (MeOH) *λ*_max_ (log *ε*): 200 (2.71) nm; IR (KBr) *ν*_max_: 3507, 3435, 3332, 2978, 2939, 2876, 1637, 1452, 1321, 1135, 1100, 1055, 973, 898, 817 cm^−1^; CD (*c* 1.77 mM, MeOH): 221 nm (Δ*ε* + 1.42); for ^13^C NMR (CDCl_3_, 150 MHz) and ^1^H NMR (CDCl_3_, 600 MHz) data, see Tables 3 and 4; HRESIMS (positive) *m/z* 283.1542 [M + H]^+^ (calcd for C_15_H_23_O_4_, 283.1540, Δ + 0.7063 ppm).

*Trichothecolone (****12****)*. White powder; $[\alpha]^{25}_{\text D}$ –5.22 (*c* 0.23, MeOH); UV (MeOH) *λ*_max_ (log *ε*): 200 (2.02) nm; IR (KBr) *ν*_max_: 3502, 2982, 2966, 2931, 1715, 1458, 1280, 1081, 957 cm^−1^; CD (*c* 8.64 mM, MeOH): 200 nm (Δ*ε* –71.20), 292 nm (Δ*ε* + 15.78); for ^13^C NMR (methanol-*d*_4_, 150 MHz) and ^1^H NMR (methanol-*d*_4_, 600 MHz) data, see Tables 3 and 4; HRESIMS (positive) *m/z* 267.1585 [M + H]^+^ (calcd for C_15_H_23_O_4_, 267.1591 Δ –2.2458 ppm).

*Epi-trichothecinol B (****13****)*. White powder; $[\alpha]^{25}_{\text D}$+ 87.5 (*c* 0.04, MeOH); UV (MeOH) *λ*_max_ (log *ε*): 200 (2.71) nm; IR (KBr) *ν*_max_: 3468, 2985, 2962, 2938, 1712, 1643, 1434, 1376, 1313, 1245, 1175, 1078, 1034, 996, 972, 962 cm^−1^; CD (*c* 1.77 mM, MeOH): 221 nm (Δ*ε* + 1.42); for ^13^C NMR (CDCl_3_, 150 MHz) and ^1^H NMR (CDCl_3_, 600 MHz) data, see Tables 3 and 4; HRESIMS (positive) *m/z* 335.1856 [M + H]^+^ (calcd for C_19_H_27_O_5_, 335.1853, Δ + 0.8950 ppm).

### Quantum chemical calculations

Conformer–rotamer ensemble sampling tool (CREST) was used to search the conformational space of compounds at the GFN0 level of theory in the gas phase [29, 30], followed by optimization at the GFN2-xTB level to avoid the errors caused by poor initial geometry [31], with a 4 kcal/mol energy window to remove high-energy conformers. Optimization and frequency calculation of each conformer were performed at the r^2^-SCAN-3c level of theory in the conductor-like polarizable continuum model (CPCM) to ensure they were true local minima on the potential energy surface [32, 33]. Under STS protocol [34], DFT GIAO ^13^C NMR calculation was calculated at the ωB97x-D/6-31G* (IEFPCM, chloroform) level or ωB97x-D/6-31G* (IEFPCM, methanol) level of theory. Time-dependent density-functional theory (TDDFT) ECD calculations were performed at the B3LYP/TZVP (IEFPCM, methanol) level of theory with the consideration of solvent effects. The calculated shielding tensors and ECD curves of conformers were Boltzmann averaged based on Gibbs free energy. In the TDDFT-ECD calculations, 40 excited states were calculated for each conformer. The ECD curves of all conformers were Boltzmann averaged based on Gibbs free energy calculated at the r^2^SCAN-3c level of theory and generated by Multiwfn [35, 36]. All DFT calculations were performed by Gaussian 16 or ORCA 6.0.0 software packages [37–39]. The DFT optimized geometry data, relative energies, and conformational population of all calculated structures were provided in the supplemental material. The 3D molecular structures were rendered using VMD (1.9.3) [40]. Details are provided in the Supplementary Information.

### Cell lines and culture conditions

The cells of GES-1, HCT116, 4T1 and MHCC97H were cultivated in DMEM medium enriched with 10% fetal bovine serum and 1% penicillin–streptomycin. Cultures were maintained at 37 °C in a humidified environment with 5% CO_2_.

### Cell viability analysis

Cell viability was determined with CCK-8 method [18]. All cell lines were plated into 96-well plates at a concentration of 10,000 cells per well and incubated for an additional 24 h. Then, the cells were treated with concentrations 5 μM and 50 μM of the test compounds and positive control (doxorubicin) for a duration of 48 h. In addition to the experimental groups, blank controls (wells with CCK-8 solution but no cells) and negative controls (wells with vehicle) were included. After treatment, 10 *μ*L of CCK-8 reagent was added to each well and incubated for 1 h. The optical density at 450 nm was then measured on a microplate reader (Feyond-A300, allsheng, Hangzhou, China). The IC_50_ value represents the half of maximal inhibitory concentration. The cells of HCT116, 4T1, and MHCC97H were treated with a concentration gradient of selected compounds (**7**, **8**, **10**, **13**, **17**, **18**, **20**, and **24)** to obtain IC_50_ values, using either the range of (0.04 μM–10 μM) or (0.5 μM–50 μM).

### Cell cycle assays

The cell cycle distribution was appraised, using the Propidium Iodide (PI) Flow Cytometry Kit, followed by the flow Cytometry analysis [12]. Briefly, HCT116 cells were planted on 6-well plates and incubated overnight. Next, the cells were treated with **7** and **8** at concentrations of 0, 1 and 2 μM, and incubated for 24 h in triplicate manner. Cells were harvested and washed with cold phosphate buffered saline (PBS), and fixed overnight with ice-cold 70% ethanol. Then, the ethanol was removed, and the cells were rinsed with PBS and stained with the DNA fluorochrome PI at room temperature in the dark for 20 min. CYTEK™ NL-3000 flow cytometer was then used to test samples.

## Conclusion

In summary, ten previously undescribed trichothecenes, three new natural products and thirteen known compounds were identified by chemical investigation of the EtOAc extract from rice cultures of the fungus *Trichothecium* sp. DWS815. Notably, trichotheciumones A (**1**) and B (**2**) are novel trichothecene skeleton characterized by the cleavage of the A ring found in classical trichothecenes and the formation of a lactone bridge across the B ring. Trichothecinoside A (**3**), trichothecrotocin V (**6**), and trichothecrotocin W (**7**) are unusual natural trichothecene characterized by a hydrogenated EPT moiety. The isolated compounds were evaluated for cytotoxic activity against three human cancer cell lines (HCT116, 4T1, MHCC97H) and one normal cell line (GES-1). Among them, compound **17** was the most potent compound with IC_50_ of 0.35 μM, 0.52 μM, 0.57 μM against HCT116, 4T1, and MHCC97H cells respectively and new compounds **7** and **8** were found to induce G2/M phase cell cycle arrest in HCT116 colorectal cancer cells, thereby inhibiting cell proliferation. This work not only expands the structural diversity of trichothecene derivatives but also provides a foundation for future studies on structure–activity relationships and mode of action.

## Supplementary Information

Supplementary Material 1: The Supplementary Information includes the 1D (^1^H, ^13^C and DEPT-135/90 NMR), 2D NMR (^1^H−^1^H COSY, HSQC, HMBC, NOESY, ROESY) and HRESIMS spectra, quantum chemical calculation data, and ITS sequence data.

## Data Availability

All data generated or analyzed during this study are included within the article and its supplementary information file.
