# Supplementary material for: Cytotoxic trichothecene derivatives from Trichothecium sp. DWS815
Source: Nat Prod Bioprospect. 2026 May 3;16(1):59. doi: 10.1007/s13659-026-00613-3 (PMC13135585; doi:10.1007/s13659-026-00613-3)
Supplement: Supplementary file 1 — Supplementary Material 1: The Supplementary Information includes the 1D (1H, 13C and DEPT-135/90 NMR), 2D NMR (1H−1H COSY, HSQC, HMBC, NOESY, ROESY) and HRESIMS spectra, quantum chemical calculation data, and ITS sequence data. [file 13659_2026_613_MOESM1_ESM.pdf]

Supporting Information

Cytotoxic Trichothecene Derivatives from *Trichothecium* sp. DWS815

Peng-Ju Xu[a], Ai-Lin Liang[a], Wen-Yu Lu[a], Hong-Ping Long[c], Qing-Hui Xiao[b], Qi-An Chen[a], Meng-Lan Hu[a], Ngoc Nhu Thao Nguyen[a], Shao Liu[b], Jing Li[a,b,]*, Wen-Xuan Wang[a,]*

[a] *Xiangya School of Pharmaceutical Sciences, Central South University, Changsha 410013, People's Republic of China*

[b] *Department of Pharmacy, National Clinical Research Center for Geriatric Disease, Xiangya Hospital, Central South University, Changsha 410008, People's Republic of China*

[c] *Center for Medical Research and Innovation, the First Hospital of Hunan University of Chinese Medicine, Changsha, People's Republic of China*

Corresponding Authors:

*E-mail: lijingliyun@csu.edu.cn (Jing Li)

*E-mail: wangwenxuan@csu.edu.cn (Wen-Xuan Wang)

Contents

Figure S1. ^{1}H NMR spectrum of **1** (600 MHz, chloroform-*d*) 7
Figure S2. ^{13}C NMR spectrum of **1** (150 MHz, chloroform-*d*) 7
Figure S3. DEPT-135 spectrum of **1** (150 MHz, chloroform-*d*) 8
Figure S4. DEPT-90 spectrum of **1** (150 MHz, chloroform-*d*) 8
Figure S5. ^{1}H–^{1}H COSY spectrum of **1** 9
Figure S6. HSQC spectrum of **1** 9
Figure S7. HMBC spectrum of **1** 10
Figure S8. NOESY spectrum of **1** 10
Figure S9. HRESIMS spectrum of **1** 11
Figure S10. UV spectrum of **1** 11
Figure S11. IR spectrum of **1** 12
Figure S12. ^{1}H NMR spectrum of **2** (600 MHz, chloroform-*d*) 12
Figure S13. ^{13}C NMR spectrum of **2** (150 MHz, chloroform-*d*) 13
Figure S14. DEPT-135 spectrum of **2** (150 MHz, chloroform-*d*) 13
Figure S15. DEPT-90 spectrum of **2** (150 MHz, chloroform-*d*) 14
Figure S16. ^{1}H–^{1}H COSY spectrum of **2** 14
Figure S17. HSQC spectrum of **2** 15
Figure S18. HMBC spectrum of **2** 15
Figure S19. NOESY spectrum of **2** 16
Figure S20. HRESIMS spectrum of **2** 16
Figure S21. UV spectrum of **2** 17
Figure S22. IR spectrum of **2** 17
Figure S23. Agilent HPLC analysis of **1** and **2** 18
Figure S24. ^{1}H NMR spectrum of **3** (600 MHz, chloroform-*d*) 18
Figure S25. ^{13}C NMR spectrum of **3** (150 MHz, chloroform-*d*) 19
Figure S26. DEPT-135 spectrum of **3** (150 MHz, chloroform-*d*) 19
Figure S27. DEPT-90 spectrum of **3** (150 MHz, chloroform-*d*) 20
Figure S28. ^{1}H–^{1}H COSY spectrum of **3** 20
Figure S29. HSQC spectrum of **3** 21
Figure S30. HMBC spectrum of **3** 21
Figure S31. NOESY spectrum of **3** 22
Figure S32. HRESIMS spectrum of **3** 22
Figure S33. UV spectrum of **3** 23
Figure S34. IR spectrum of **3** 23
Figure S35. ^{1}H NMR spectrum of **4** (600 MHz, chloroform-*d*) 24
Figure S36. ^{13}C NMR spectrum of **4** (150 MHz, chloroform-*d*) 24
Figure S37. DEPT-135 spectrum of **4** (150 MHz, chloroform-*d*) 25
Figure S38. DEPT-90 spectrum of **4** (150 MHz, chloroform-*d*) 25

Figure S39. ^{1}H–^{1}H COSY spectrum of **4** 26
Figure S40. HSQC spectrum of **4** 26
Figure S41. HMBC spectrum of **4** 27
Figure S42. NOESY spectrum of **4** 27
Figure S43. HRESIMS spectrum of **4** 28
Figure S44. UV spectrum of **4** 28
Figure S45. IR spectrum of **4** 29
Figure S46. ^{1}H NMR spectrum of **5** (600 MHz, chloroform-*d*) 29
Figure S47. ^{13}C NMR spectrum of **5** (150 MHz, chloroform-*d*) 30
Figure S48. DEPT-135 spectrum of **5** (150 MHz, chloroform-*d*) 30
Figure S49. DEPT-90 spectrum of **5** (150 MHz, chloroform-*d*) 31
Figure S50. ^{1}H–^{1}H COSY spectrum of **5** 31
Figure S51. HSQC spectrum of **5** 32
Figure S52. HMBC spectrum of **5** 32
Figure S53. NOESY spectrum of **5** 33
Figure S54. HRESIMS spectrum of **5** 33
Figure S55. UV spectrum of **5** 34
Figure S56. IR spectrum of **5** 34
Figure S57. ^{1}H NMR spectrum of **6** (600 MHz, chloroform-*d*) 35
Figure S58. ^{13}C NMR spectrum of **6** (150 MHz, chloroform-*d*) 35
Figure S59. DEPT-135 spectrum of **6** (150 MHz, chloroform-*d*) 36
Figure S60. DEPT-90 spectrum of **6** (150 MHz, chloroform-*d*) 36
Figure S61. ^{1}H–^{1}H COSY spectrum of **6** 37
Figure S62. HSQC spectrum of **6** 37
Figure S63. HMBC spectrum of **6** 38
Figure S64. NOESY spectrum of **6** 38
Figure S65. HRESIMS spectrum of **6** 39
Figure S66. UV spectrum of **6** 39
Figure S67. IR spectrum of **6** 40
Figure S68. ^{1}H NMR spectrum of **7** (600 MHz, chloroform-*d*) 40
Figure S69. ^{13}C NMR spectrum of **7** (150 MHz, chloroform-*d*) 41
Figure S70. DEPT-135 spectrum of **7** (150 MHz, chloroform-*d*) 41
Figure S71. DEPT-90 spectrum of **7** (150 MHz, chloroform-*d*) 42
Figure S72. ^{1}H–^{1}H COSY spectrum of **7** 42
Figure S73. HSQC spectrum of **7** 43
Figure S74. HMBC spectrum of **7** 43
Figure S75. ROESY spectrum of **7** 44
Figure S76. HRESIMS spectrum of **7** 44
Figure S77. UV spectrum of **7** 45

Figure S78. IR spectrum of **7** 45
Figure S79. ^{1}H NMR spectrum of **8** (500 MHz, chloroform-*d*) 46
Figure S80. ^{13}C NMR spectrum of **8** (125 MHz, chloroform-*d*) 46
Figure S81. DEPT-135 spectrum of **8** (125 MHz, chloroform-*d*) 47
Figure S82. DEPT-90 spectrum of **8** (125 MHz, chloroform-*d*) 47
Figure S83. ^{1}H–^{1}H COSY spectrum of **8**.. 48
Figure S84. HSQC spectrum of **8**.. 48
Figure S85. HMBC spectrum of **8**.. 49
Figure S86. NOESY spectrum of **8** 49
Figure S87. HRESIMS spectrum of **8** 50
Figure S88. UV spectrum of **8**.. 50
Figure S89. IR spectrum of **8** 51
Figure S90. Elite HPLC comparative analysis of **8** and **22** 51
Figure S91. ^{1}H NMR spectrum of **9** (500 MHz, chloroform-*d*) 52
Figure S92. ^{13}C NMR spectrum of **9** (125 MHz, chloroform-*d*) 52
Figure S93. DEPT-135 spectrum of **9** (125 MHz, chloroform-*d*) 53
Figure S94. DEPT-90 spectrum of **9** (125 MHz, chloroform-*d*) 53
Figure S95. ^{1}H–^{1}H COSY spectrum of **9** (125 MHz, chloroform-*d*) 54
Figure S96. HSQC spectrum of **9**.. 54
Figure S97. HMBC spectrum of **9**.. 55
Figure S98. NOESY spectrum of **9** 55
Figure S99. HRESIMS spectrum of **9** 56
Figure S100. UV spectrum of **9**.. 56
Figure S101. IR spectrum of **9** 57
Figure S102. ^{1}H NMR spectrum of **10** (600 MHz, dimethyl sulfoxide-d_6).. 57
Figure S103. ^{13}C NMR spectrum of **10** (150 MHz, dimethyl sulfoxide-d_6).. 58
Figure S104. DEPT-135 spectrum of **10** (150 MHz, dimethyl sulfoxide-d_6).. 58
Figure S105. DEPT-90 spectrum of **10** (150 MHz, dimethyl sulfoxide-d_6).. 59
Figure S106. ^{1}H–^{1}H COSY spectrum of **10** 59
Figure S107. HSQC spectrum of **10**.. 60
Figure S108. HMBC spectrum of **10**.. 60
Figure S109. NOESY spectrum of **10** 61
Figure S110. HRESIMS spectrum of **10** 61
Figure S111. UV spectrum of **10**.. 62
Figure S112. IR spectrum of **10** 62
Figure S113. ^{1}H NMR spectrum of **11** (600 MHz, chloroform-*d*) 63
Figure S114. ^{13}C NMR spectrum of **11** (150 MHz, chloroform-*d*) 63
Figure S115. DEPT-135 spectrum of **11** (150 MHz, chloroform-*d*) 64
Figure S116. DEPT-90 spectrum of **11** (150 MHz, chloroform-*d*) 64

Figure S117. $^{1}H-^{1}H$ COSY spectrum of **11** 65
Figure S118. HSQC spectrum of **11** 65
Figure S119. HMBC spectrum of **11** 66
Figure S120. NOESY spectrum of **11** 66
Figure S121. HRESIMS spectrum of **11** 67
Figure S122. UV spectrum of **11** 67
Figure S123. IR spectrum of **11** 68
Figure S124. ^{1}H NMR spectrum of **12** (600 MHz, methanol-d_4) 68
Figure S125. ^{13}C NMR spectrum of **12** (150 MHz, methanol-d_4) 69
Figure S126. DEPT-135 spectrum of **12** (150 MHz, methanol-d_4) 69
Figure S127. DEPT-90 spectrum of **12** (150 MHz, methanol-d_4) 70
Figure S128. $^{1}H-^{1}H$ COSY spectrum of **12** 70
Figure S129. HSQC spectrum of **12** 71
Figure S130. HMBC spectrum of **12** 71
Figure S131. NOESY spectrum of **12** 72
Figure S132. HRESIMS spectrum of **12** 72
Figure S133. UV spectrum of **12** 73
Figure S134. IR spectrum of **12** 73
Figure S135. ^{1}H NMR spectrum of **13** (600 MHz, chloroform-*d*) 74
Figure S136. ^{13}C NMR spectrum of **13** (150 MHz, chloroform-*d*) 74
Figure S137. DEPT-135 spectrum of **13** (150 MHz, chloroform-*d*) 75
Figure S138. DEPT-90 spectrum of **13** (150 MHz, chloroform-*d*) 75
Figure S139. HSQC spectrum of **13** 76
Figure S140. HRESIMS spectrum of **13** 76
Figure S141. UV spectrum of **13** 77
Figure S142. IR spectrum of **13** 77
Figure S143. Calculated and experimental ECD spectra of **13**. 78
Table S1. Geometry data of conformers of structure **1a** 79
Table S2. Geometry data of conformers of structure **2a** 83
Table S3. Geometry data of conformers of structure **3a**, **3b**, **3c**, **3d**, **3e**, **3f**, **3g** and **3h** 85
Table S4. Experimental (Exptl.) of **3** and the calculated ^{13}C chemical shift values of possible isomers (**3a**, **3b**, **3c**, **3d**, **3e**, **3f**, **3g** and **3h**) with GIAO ^{13}C NMR calculations with STS protocol 127
Table S5. Geometry data of conformers of structure **3a** for ECD calculation. 129
Table S6. Geometry data of conformers of structure **4a** and 4b 138
Table S7. Experimental (Exptl.) of **4** and the calculated ^{13}C chemical shift values of possible isomers (**4a** and **4b**) with GIAO ^{13}C NMR calculations with STS protocol. 142
Table S8. Geometry data of conformers of structure **5a** and **5b** 143

Table S9. Experimental (Exptl.) of **5** and the calculated ^{13}C chemical shift values of possible isomers (**5a** and **5b**) with GIAO ^{13}C NMR calculations with STS protocol. 160
Table S10. Geometry data of conformers of structure **6a** and **6b** 161
Table S11. Experimental (Exptl.) of **6** and the calculated ^{13}C chemical shift values of possible isomers (**6a** and **6b**) with GIAO ^{13}C NMR calculations with STS protocol. 165
Table S12. Geometry data of conformers of structure **7a** and **7b** 166
Table S13. Experimental (Exptl.) of **7** and the calculated ^{13}C chemical shift values of possible isomers (**7a** and **7b**) with GIAO ^{13}C NMR calculations with STS protocol. 170
Table S14. Geometry data of conformers of structure **8a** 171
Table S15. Geometry data of conformers of structure **9a** 172
Table S16. Geometry data of conformers of structure **10a** 173
Table S17. Geometry data of conformers of structure **11a** and **11b** 175
Table S18. Experimental (Exptl.) of **11** and the calculated ^{13}C chemical shift values of possible isomers (**11a** and **11b**) with GIAO ^{13}C NMR calculations with STS protocol. 181
Table S19. Geometry data of conformers of structure **12a** and **12b** 182
Table S20. Experimental (Exptl.) of **12** and the calculated ^{13}C chemical shift values of possible isomers (**12a** and **12b**) with GIAO ^{13}C NMR calculations with STS protocol. 185
Table S21. Geometry data of conformers of structure **13a** and **13b** 186
Table S22. Experimental (Exptl.) of **13** and the calculated ^{13}C chemical shift values of possible isomers (**13a** and **13b**) with GIAO ^{13}C NMR calculations with STS protocol. 192
Table S23. Geometry data of conformers of structure **13a** for ECD calculation. 194
Table S24. ITS sequence of *Trichothecium* sp. DWS815 198
Table S25. Maximum likelihood phylogenetic tree was constructed using ITS sequences. Bootstrap support values of maximum likelihood above 50% are shown at the nodes......... 199
Table S26. The pairwise genetic distance matrix of ITS sequences based on the Kimura 2-parameter model (complete deletion).. 199

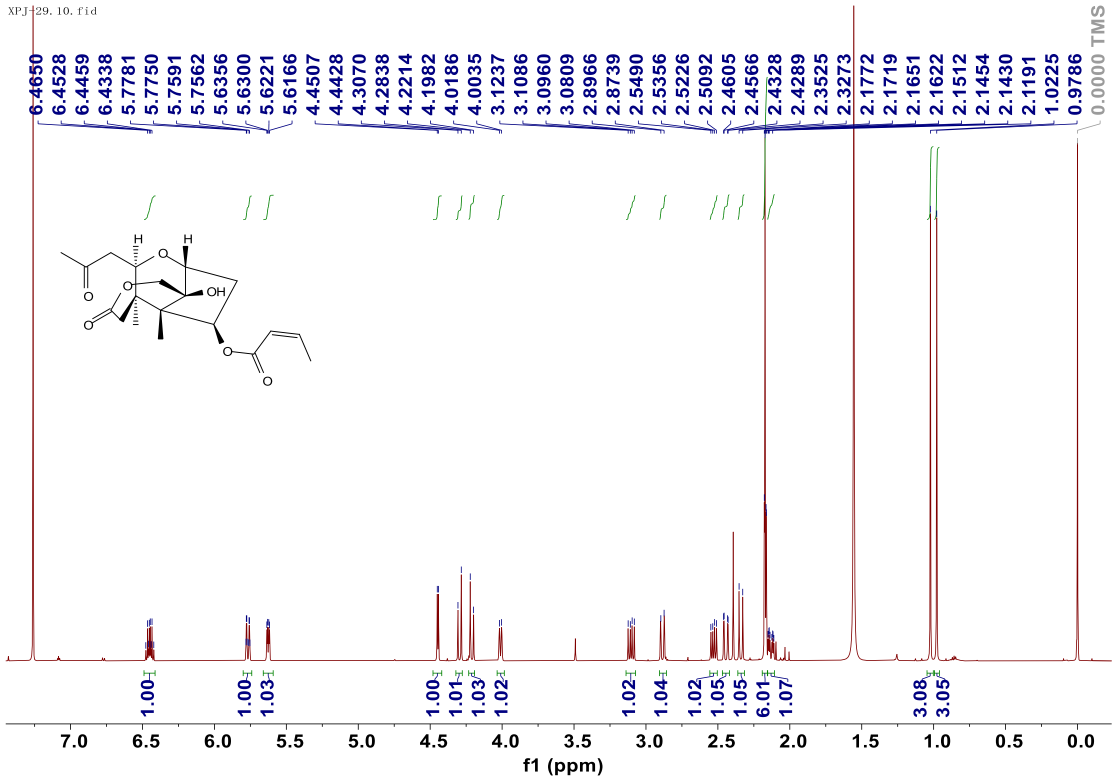

Figure S1. ^{1}H NMR spectrum of **1** (600 MHz, chloroform-*d*)

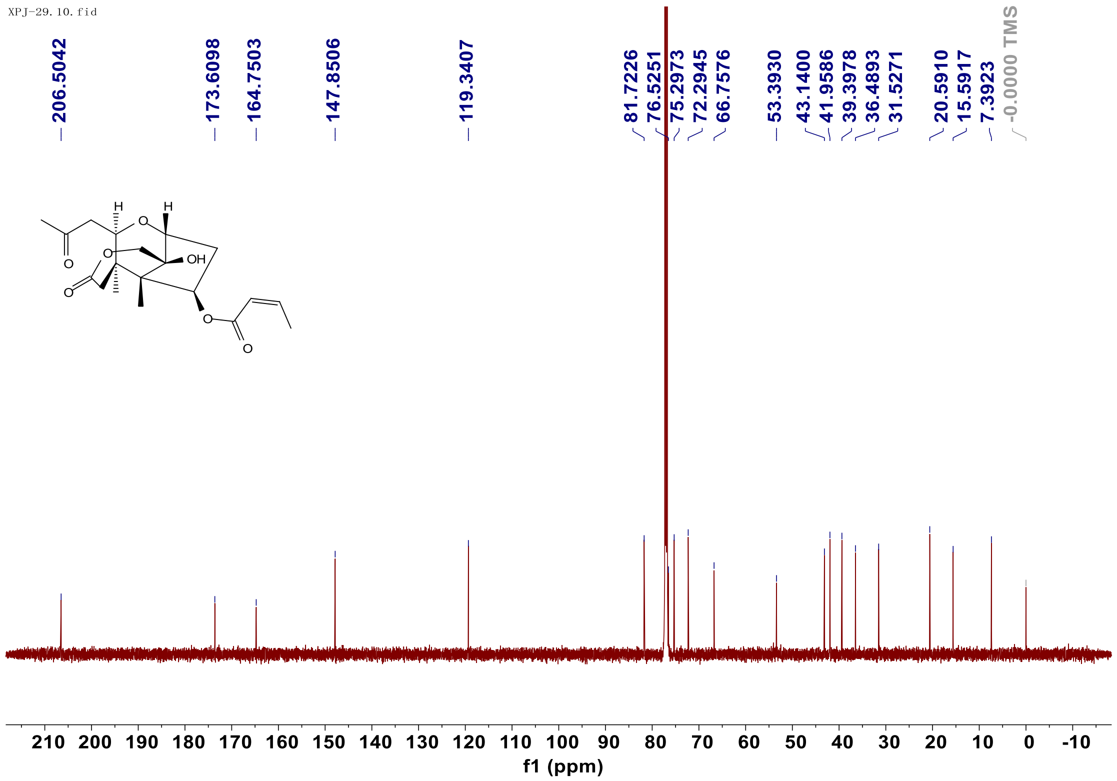

Figure S2. ^{13}C NMR spectrum of **1** (150 MHz, chloroform-*d*)

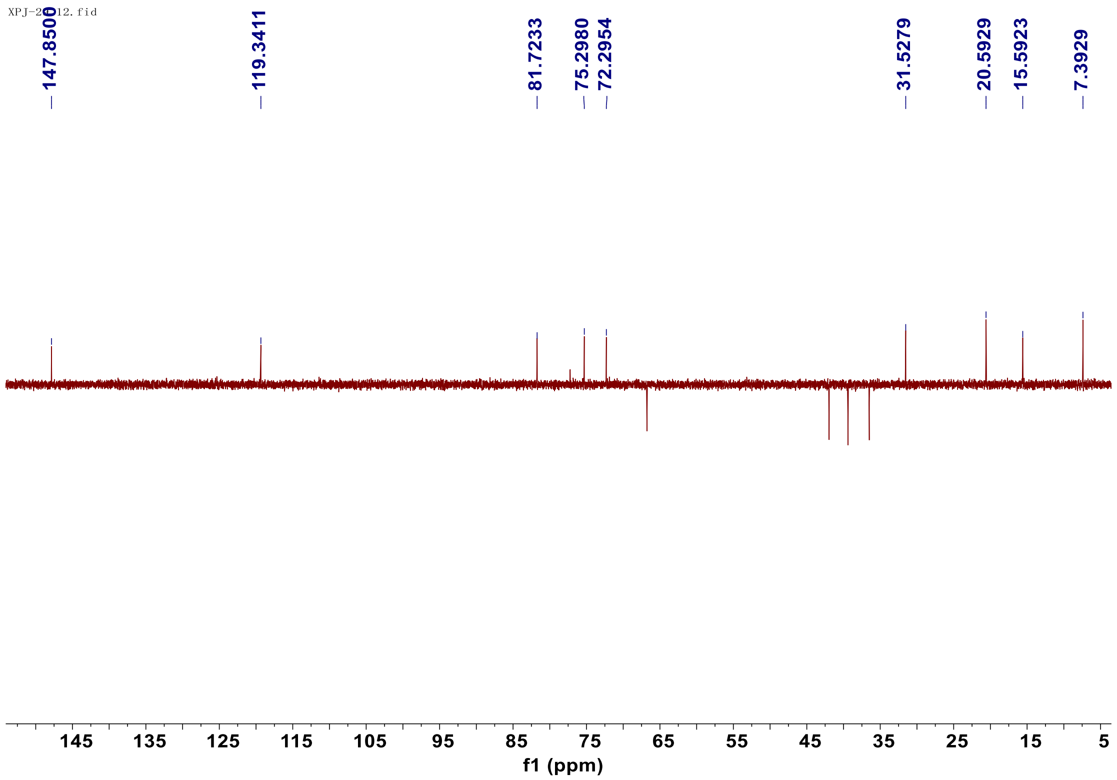

Figure S3. DEPT-135 spectrum of **1** (150 MHz, chloroform-*d*)

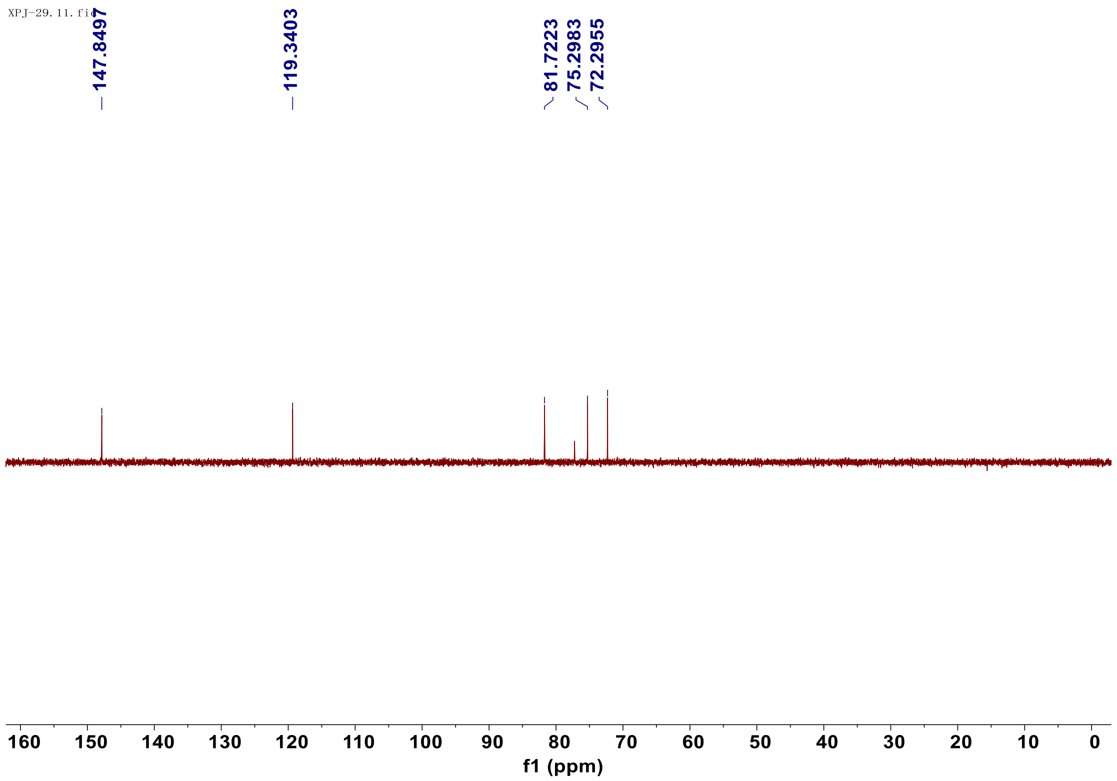

Figure S4. DEPT-90 spectrum of **1** (150 MHz, chloroform-*d*)

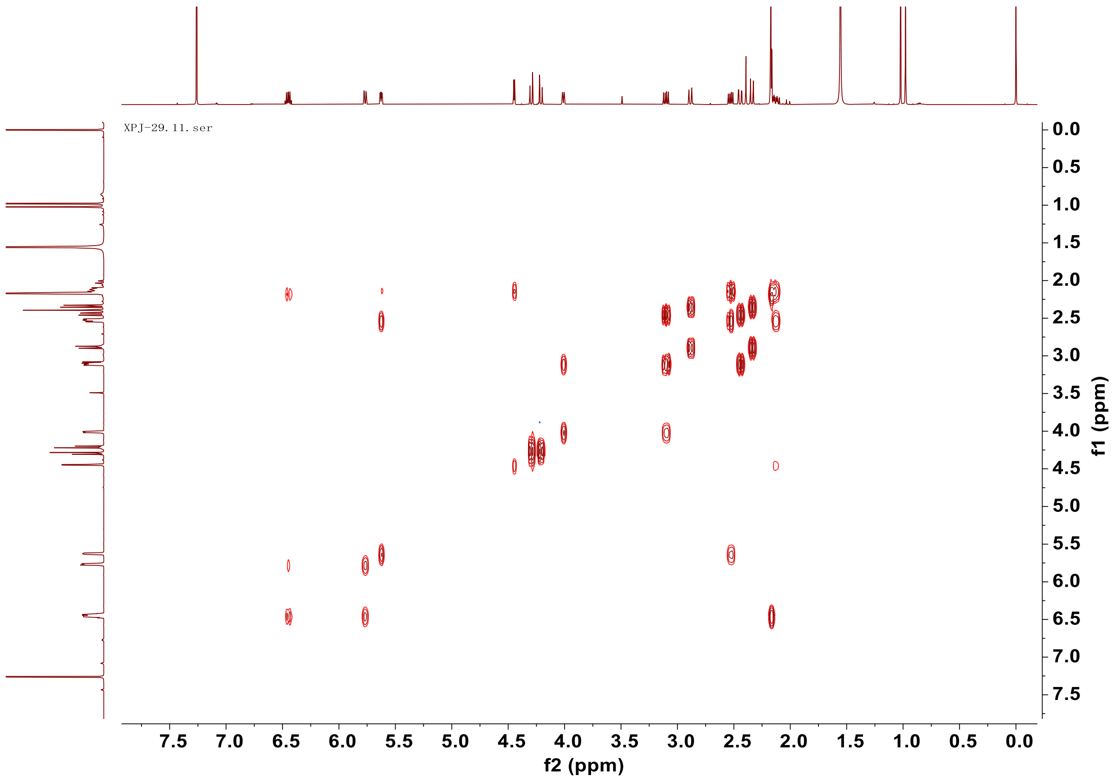

Figure S5. $^{1}H-^{1}H$ COSY spectrum of **1**

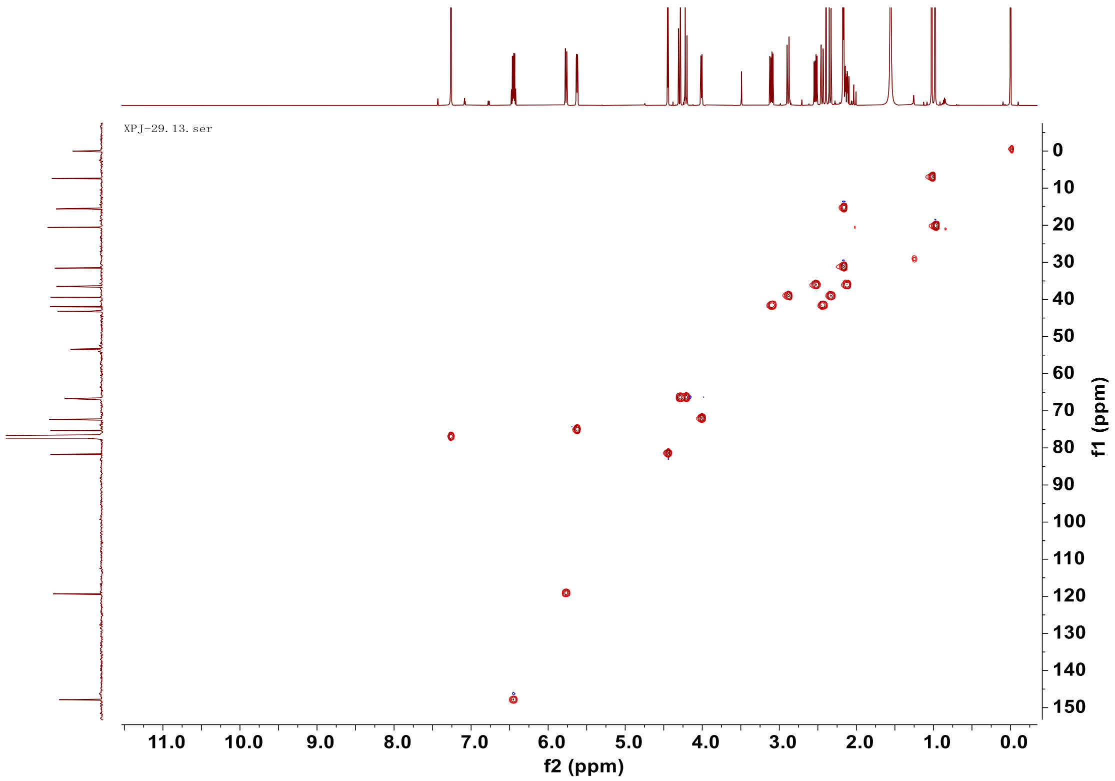

Figure S6. HSQC spectrum of **1**

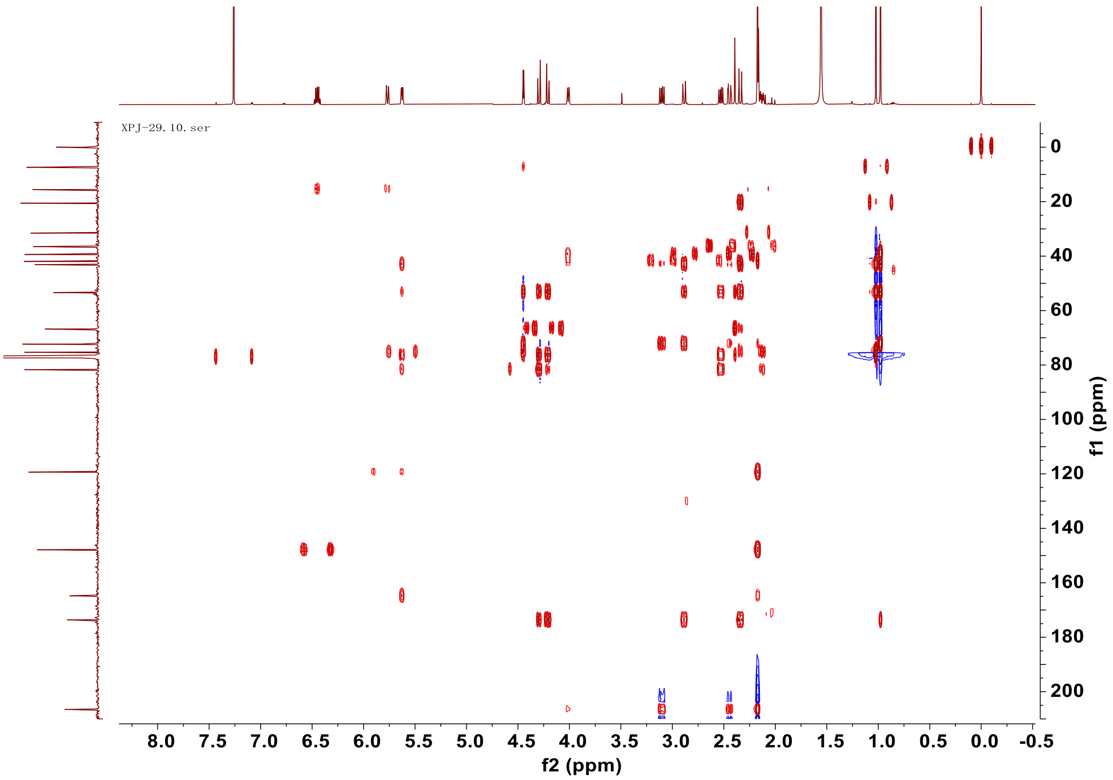

Figure S7. HMBC spectrum of **1**

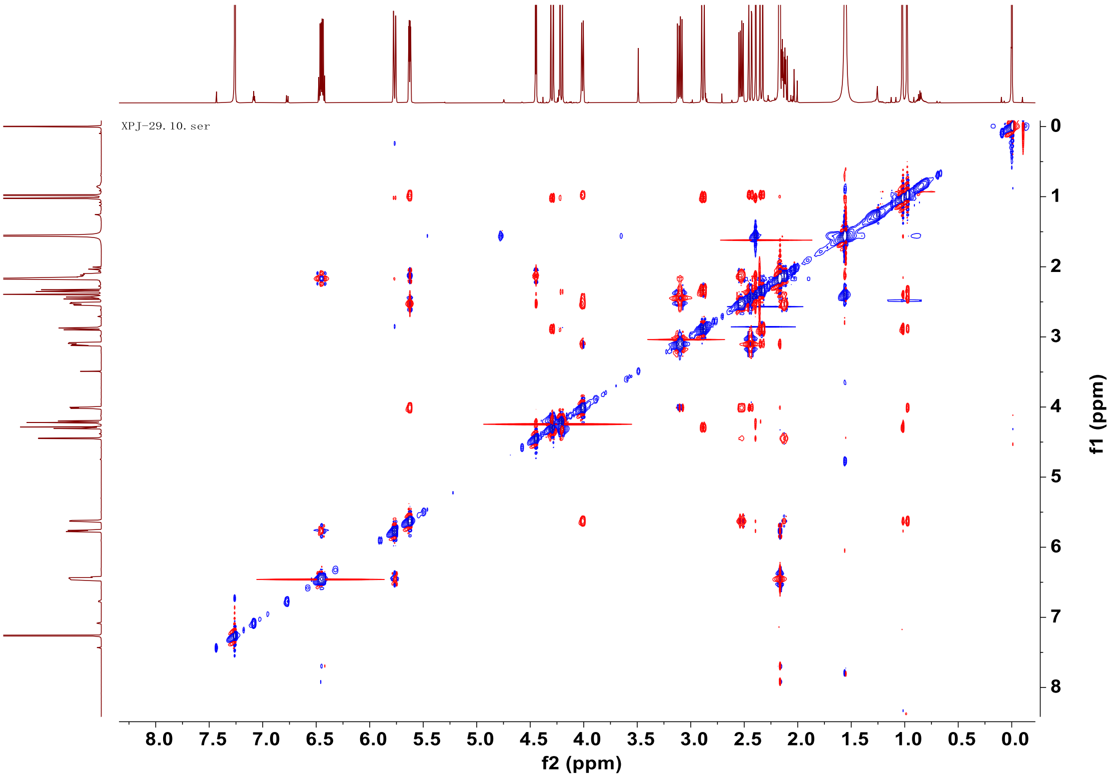

Figure S8. NOESY spectrum of **1**

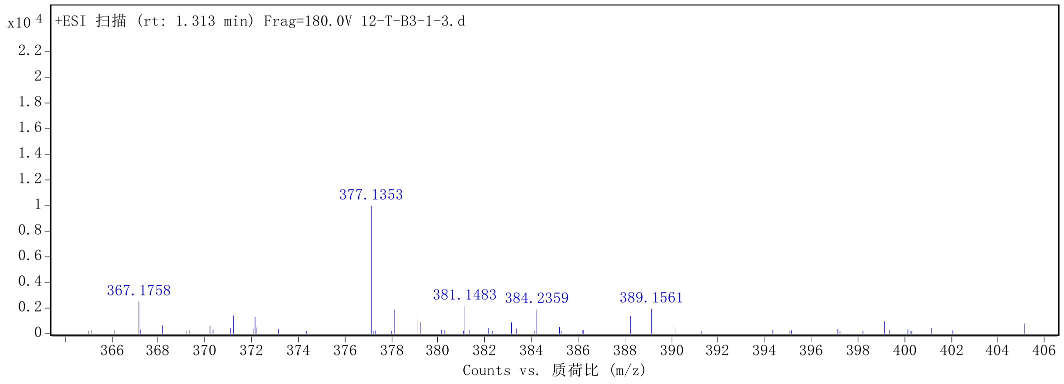

Figure S9. HRESIMS spectrum of **1**

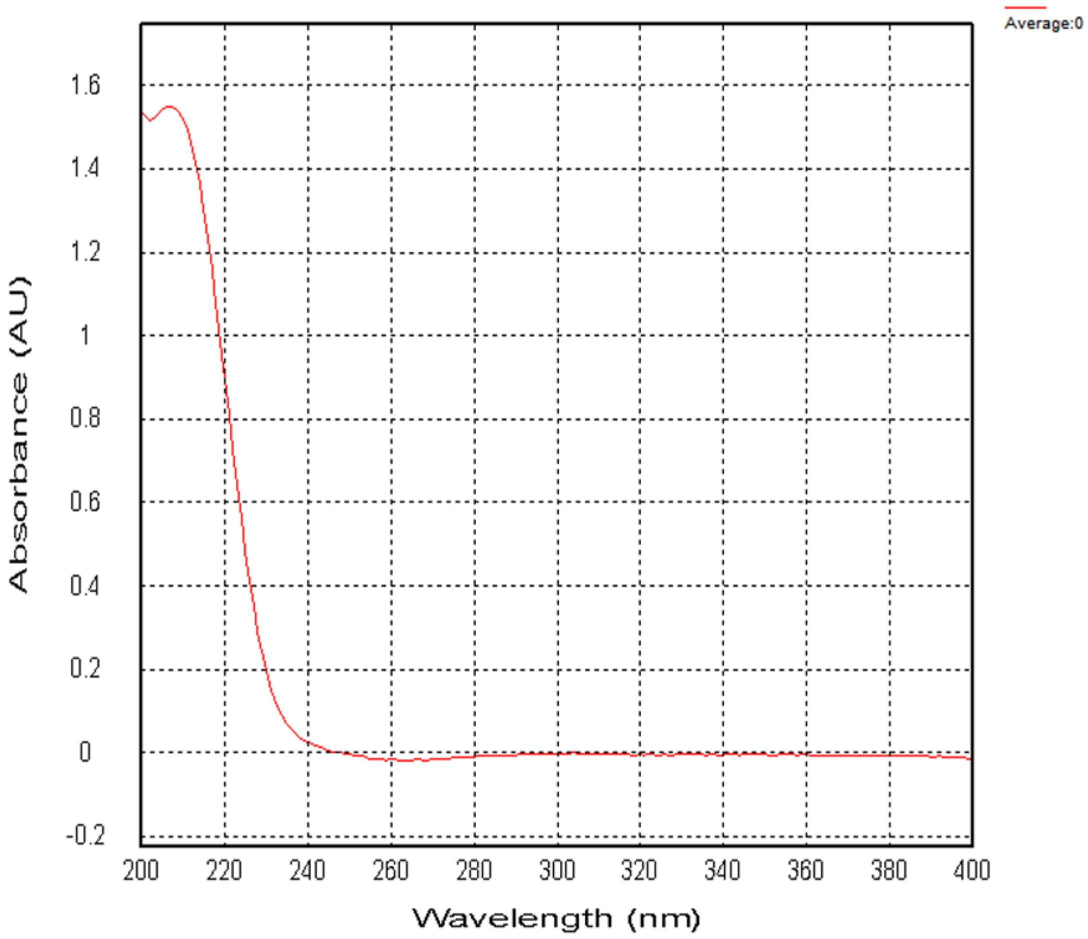

Figure S10. UV spectrum of **1**

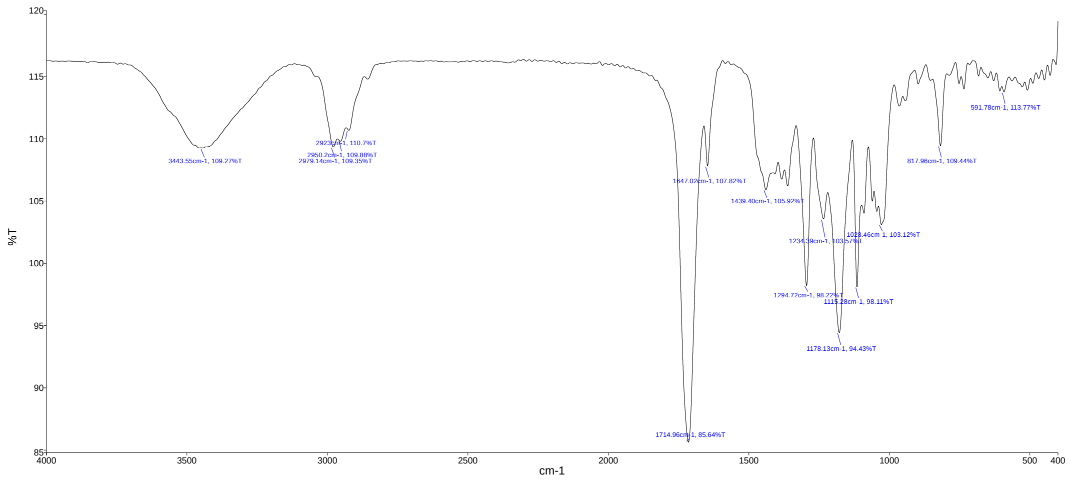

Figure S11. IR spectrum of **1**

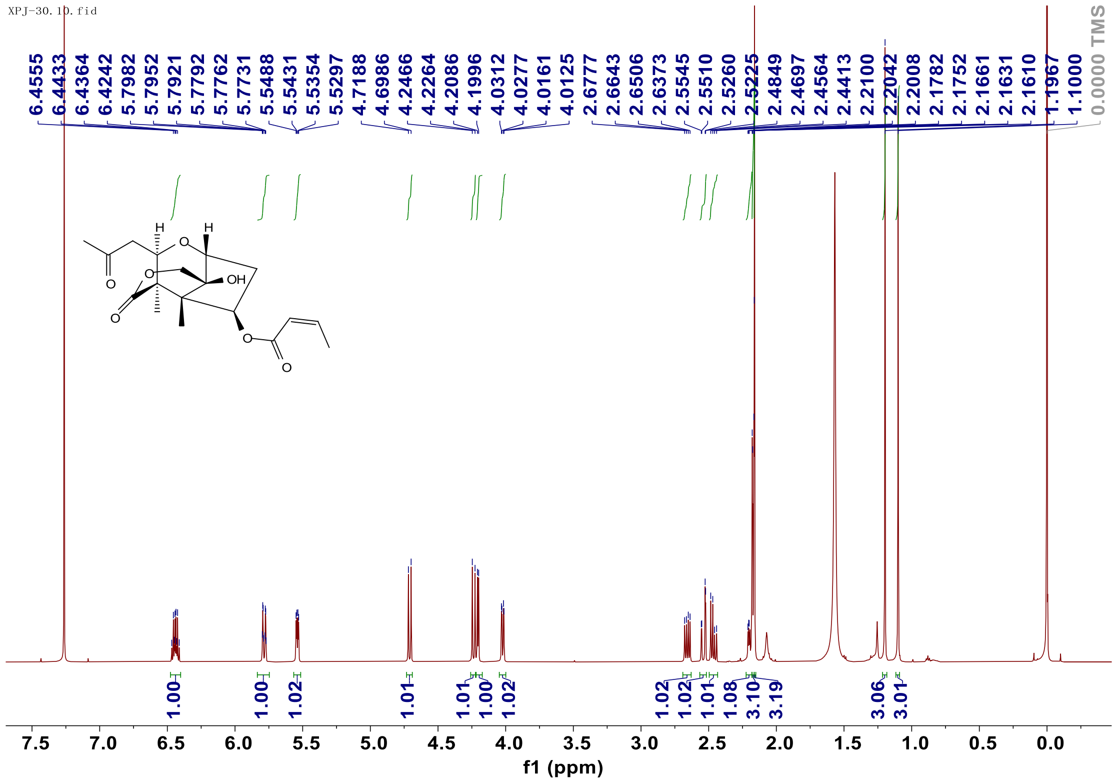

Figure S12. ^{1}H NMR spectrum of **2** (600 MHz, chloroform-*d*)

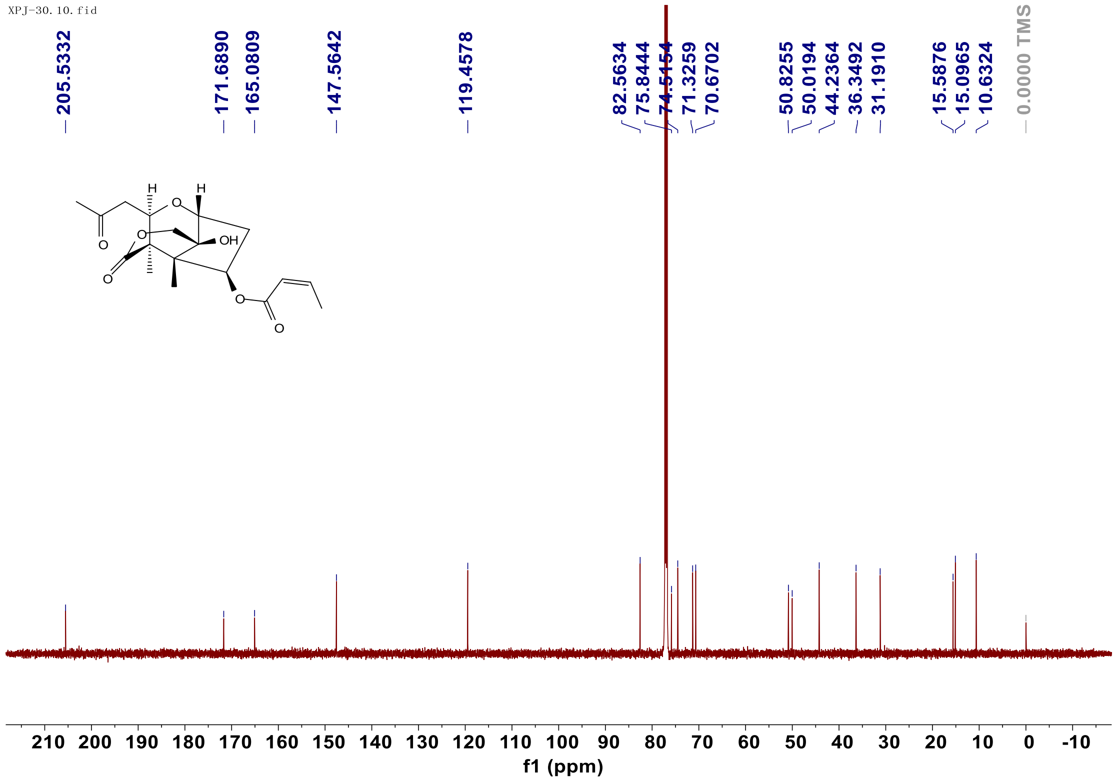

Figure S13. ^{13}C NMR spectrum of **2** (150 MHz, chloroform-*d*)

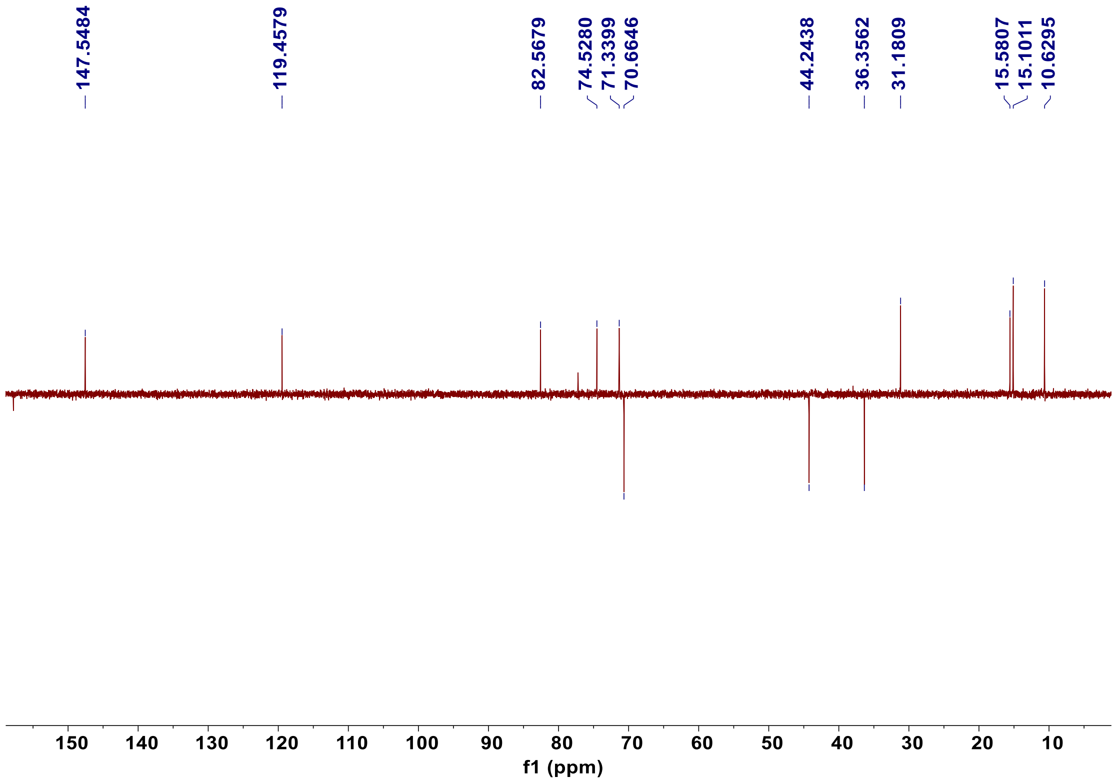

Figure S14. DEPT-135 spectrum of **2** (150 MHz, chloroform-*d*)

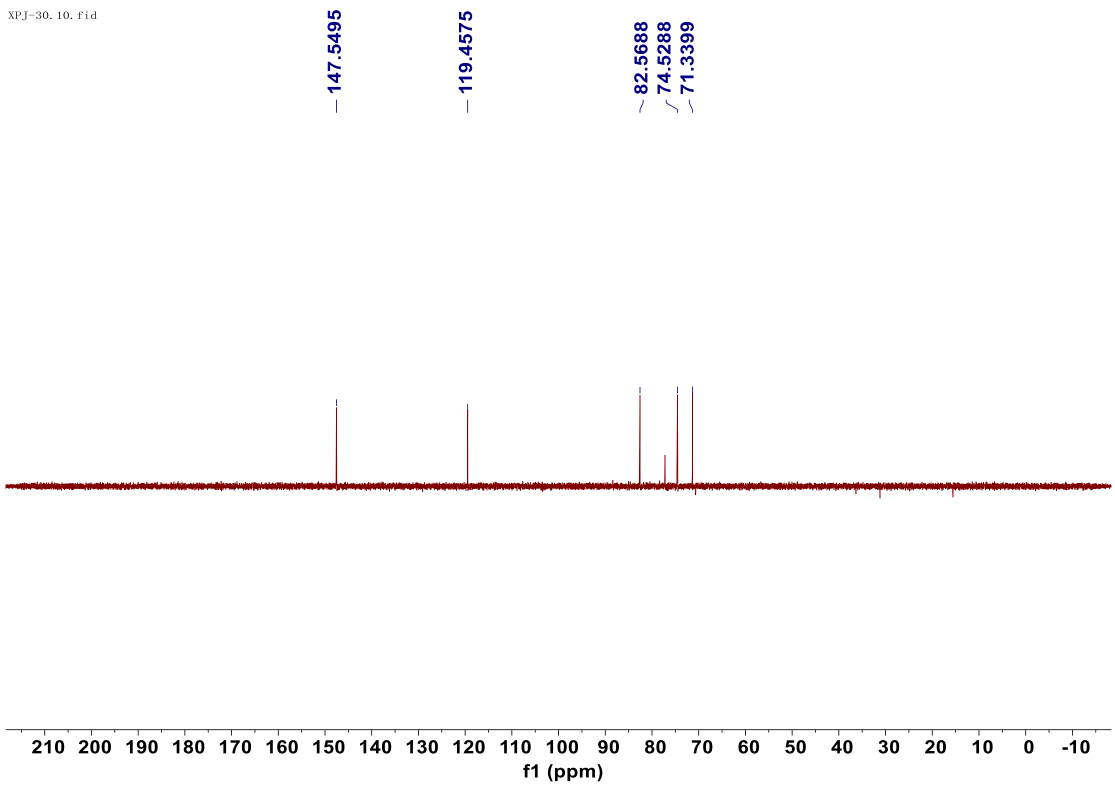

Figure S15. DEPT-90 spectrum of **2** (150 MHz, chloroform-*d*)

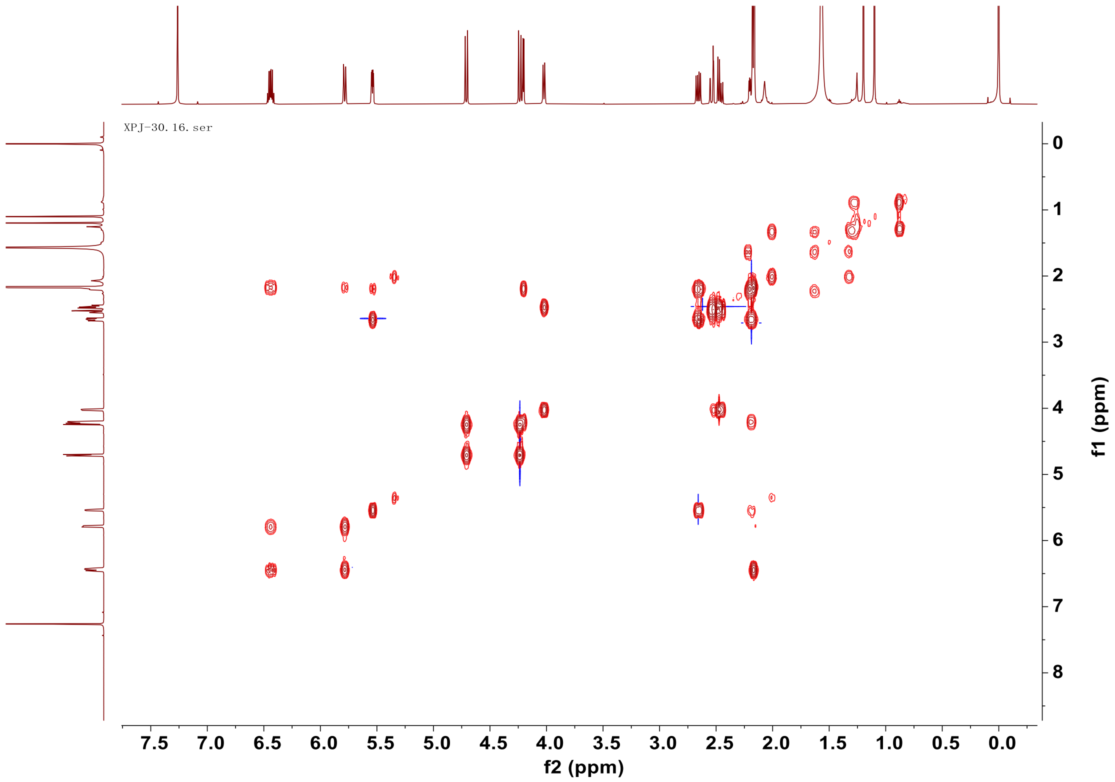

Figure S16. $^{1}H-^{1}H$ COSY spectrum of **2**

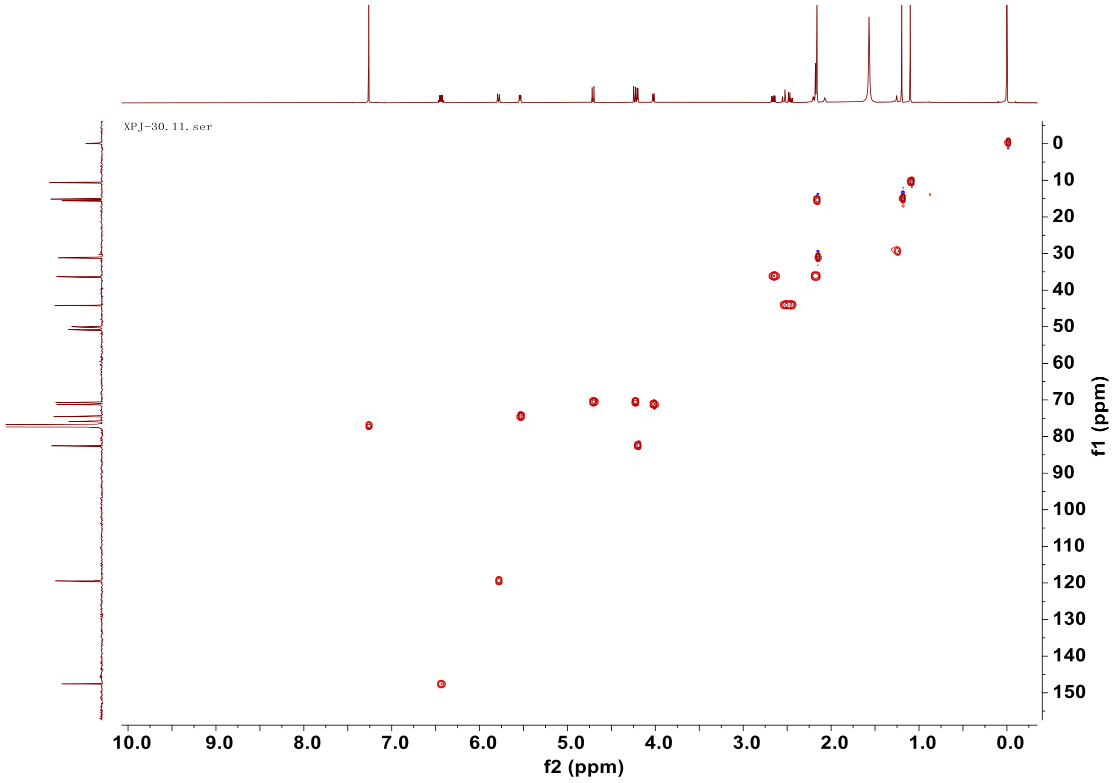

Figure S17. HSQC spectrum of **2**

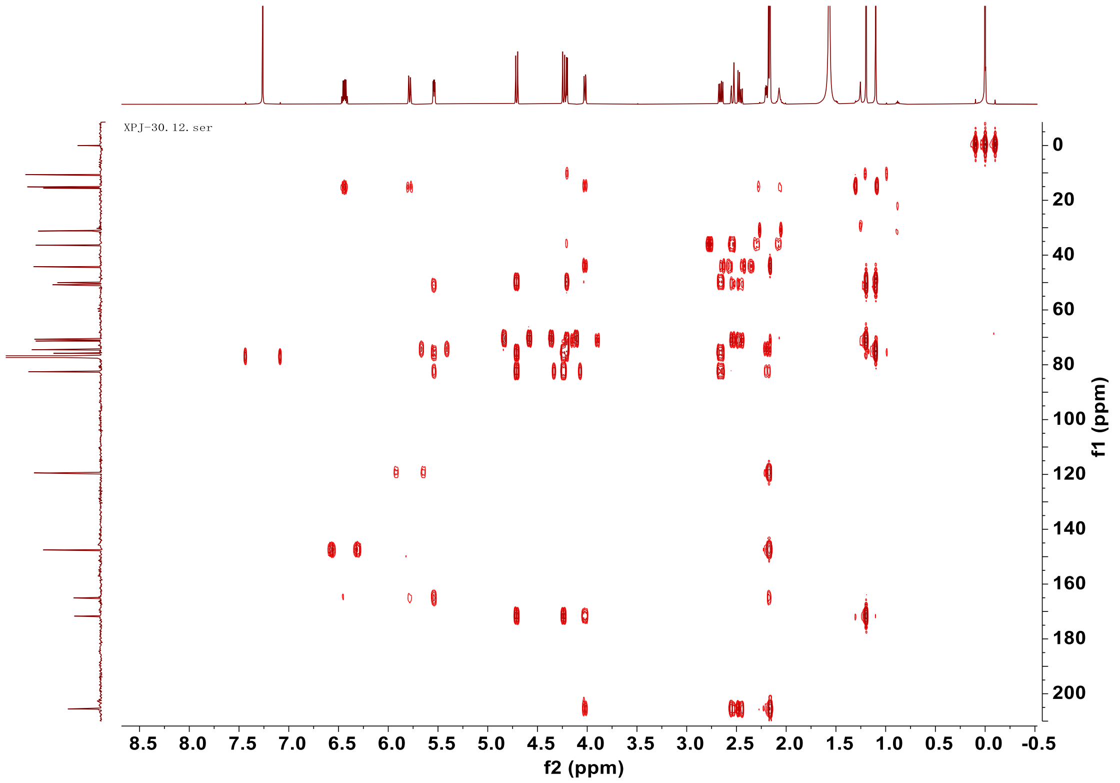

Figure S18. HMBC spectrum of **2**

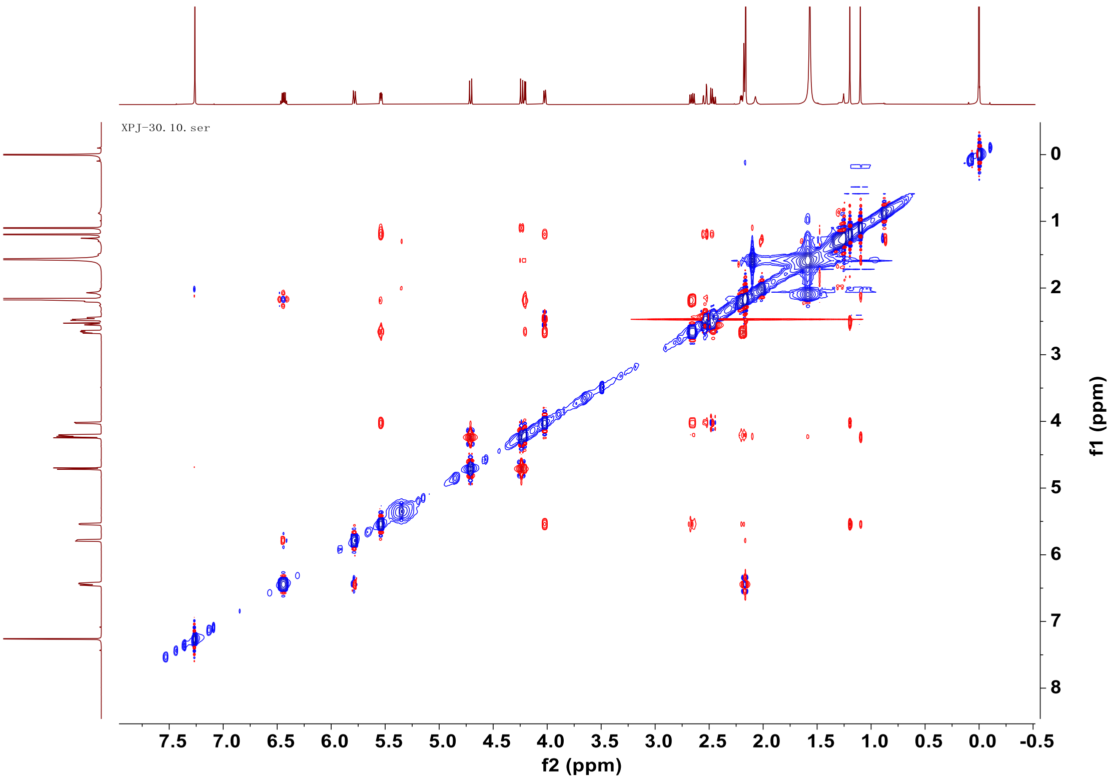

Figure S19. NOESY spectrum of **2**

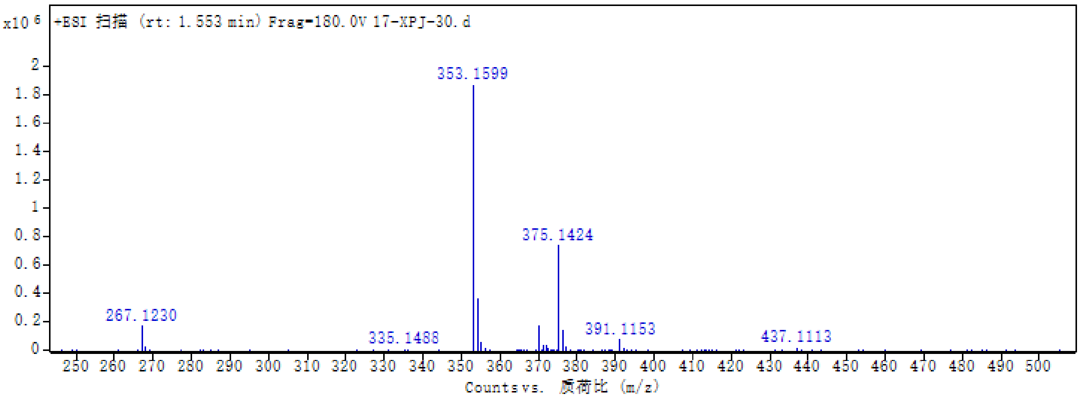

Figure S20. HRESIMS spectrum of **2**

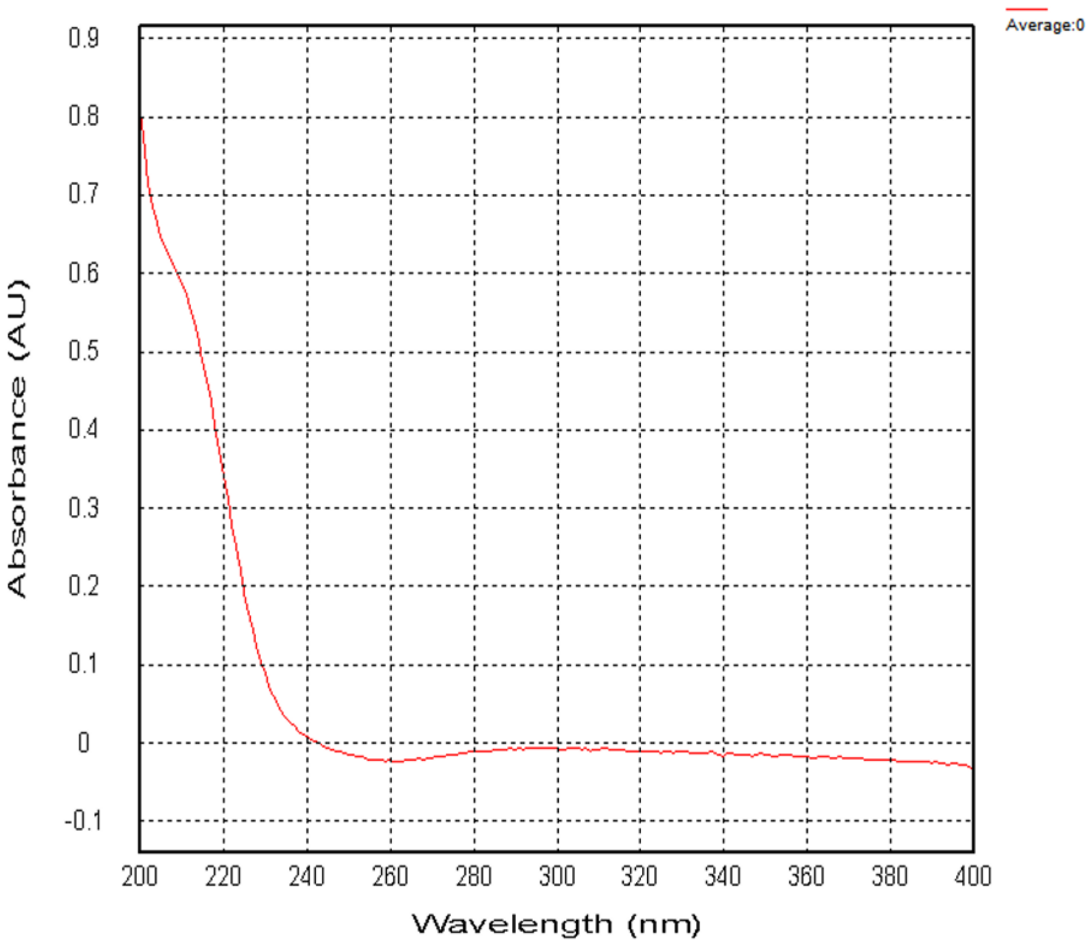

Figure S21. UV spectrum of **2**

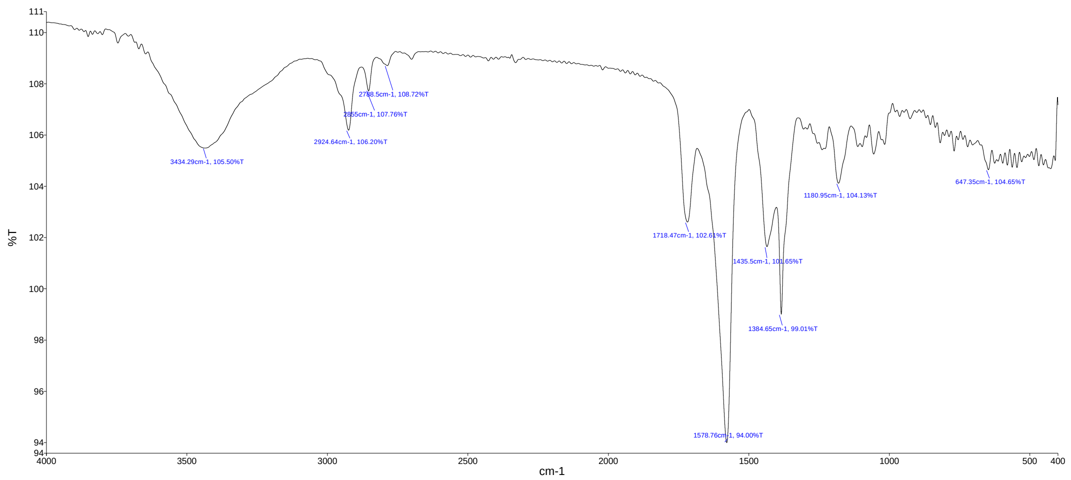

Figure S22. IR spectrum of **2**

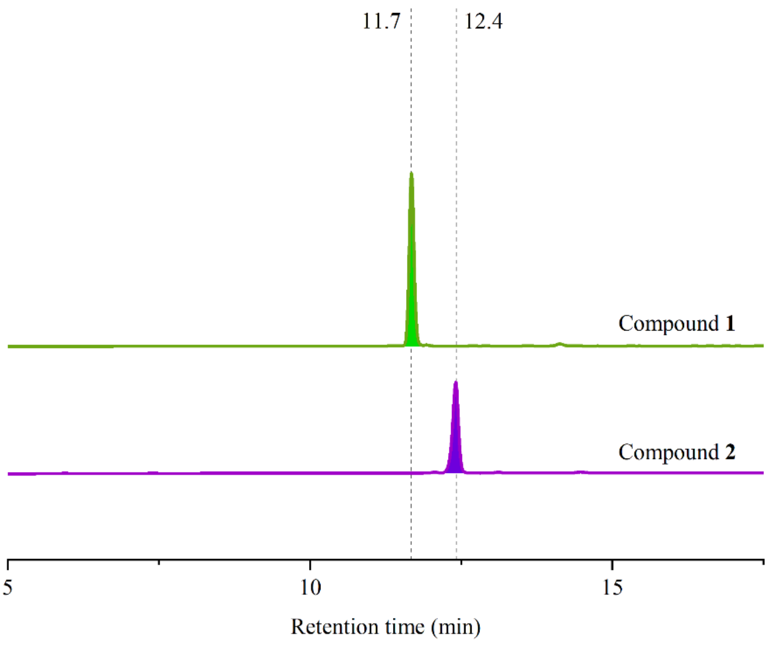

Figure S23. Agilent HPLC analysis of **1** and **2**

Analysis method: ZORBAX SB-C18 5μm 4.6 × 150mm, 5−95% CH_3CN-H_2O, 30 min, 210 nm

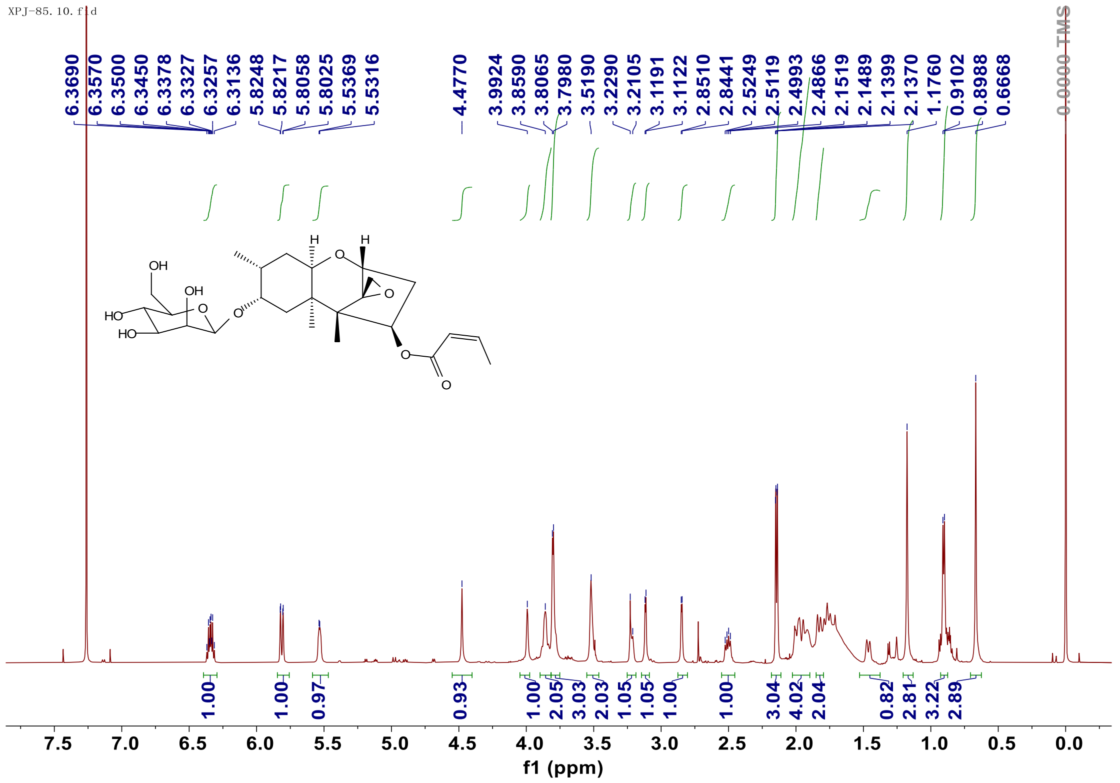

Figure S24. ^{1}H NMR spectrum of **3** (600 MHz, chloroform-*d*)

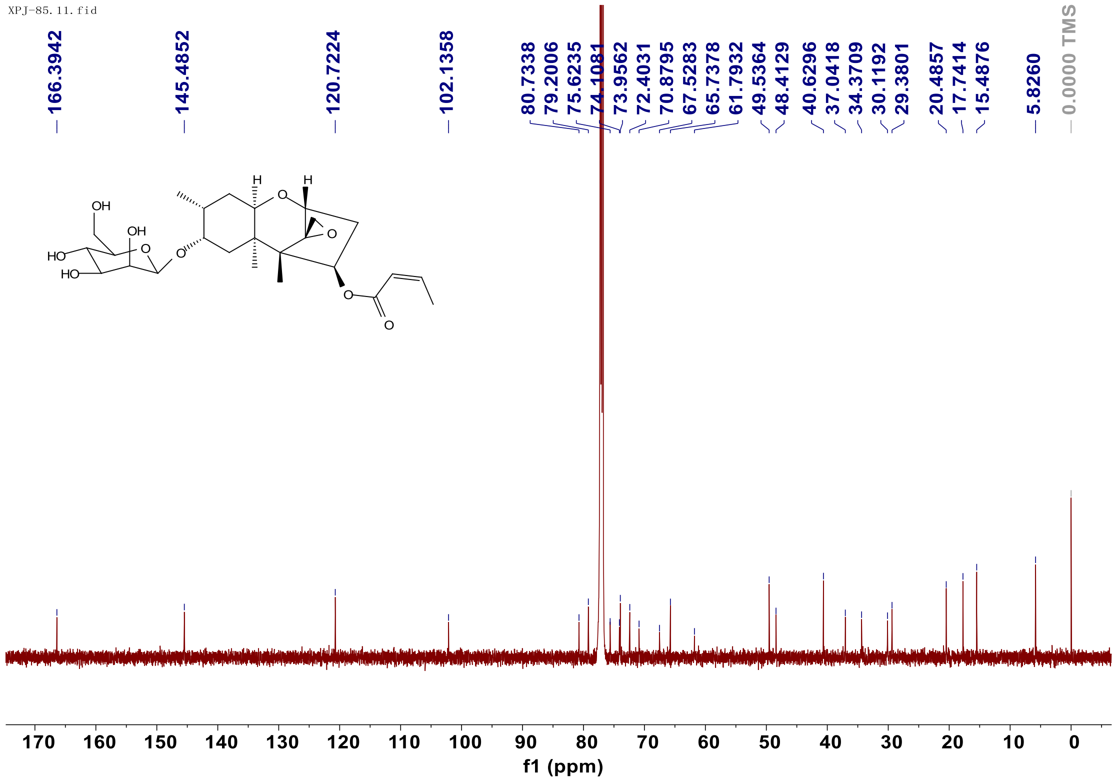

Figure S25. ^{13}C NMR spectrum of **3** (150 MHz, chloroform-*d*)

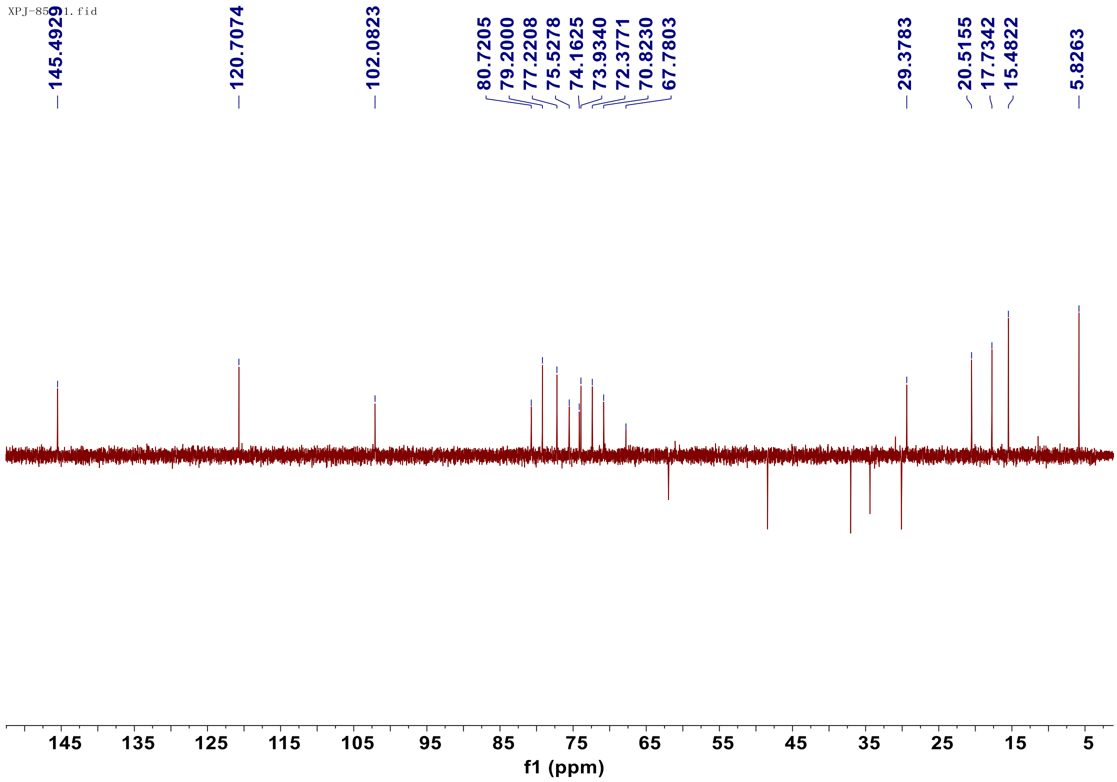

Figure S26. DEPT-135 spectrum of **3** (150 MHz, chloroform-*d*)

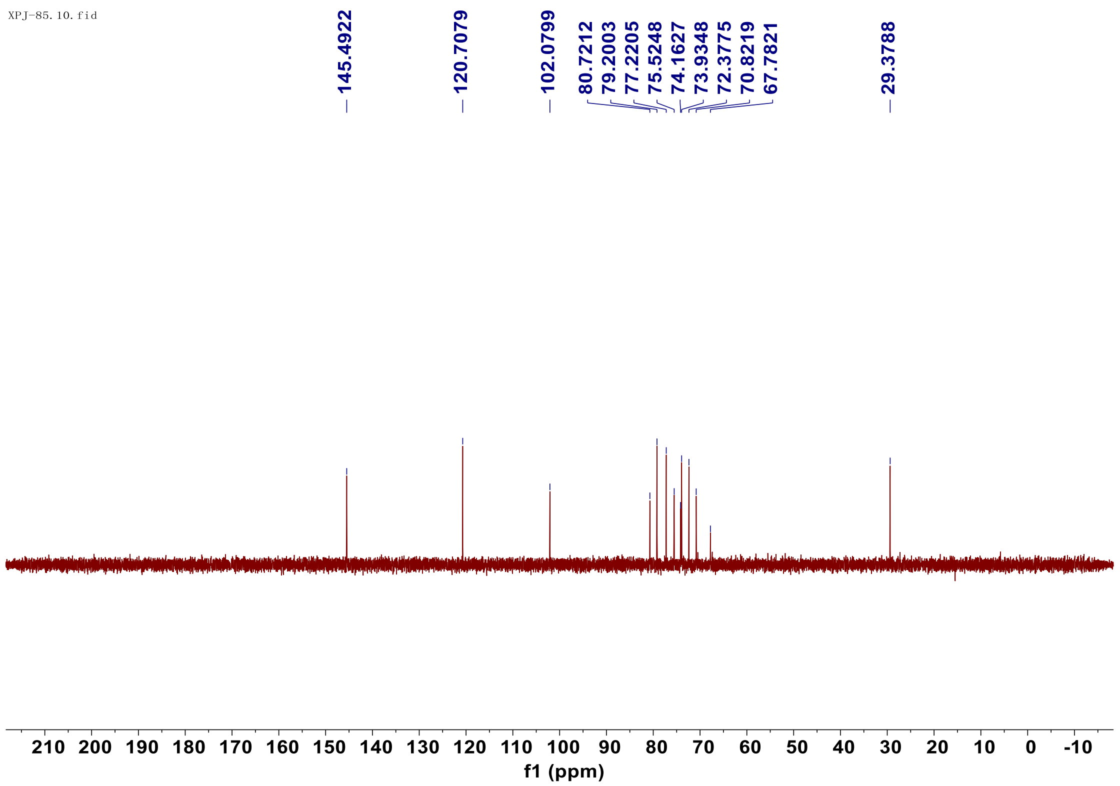

Figure S27. DEPT-90 spectrum of **3** (150 MHz, chloroform-*d*)

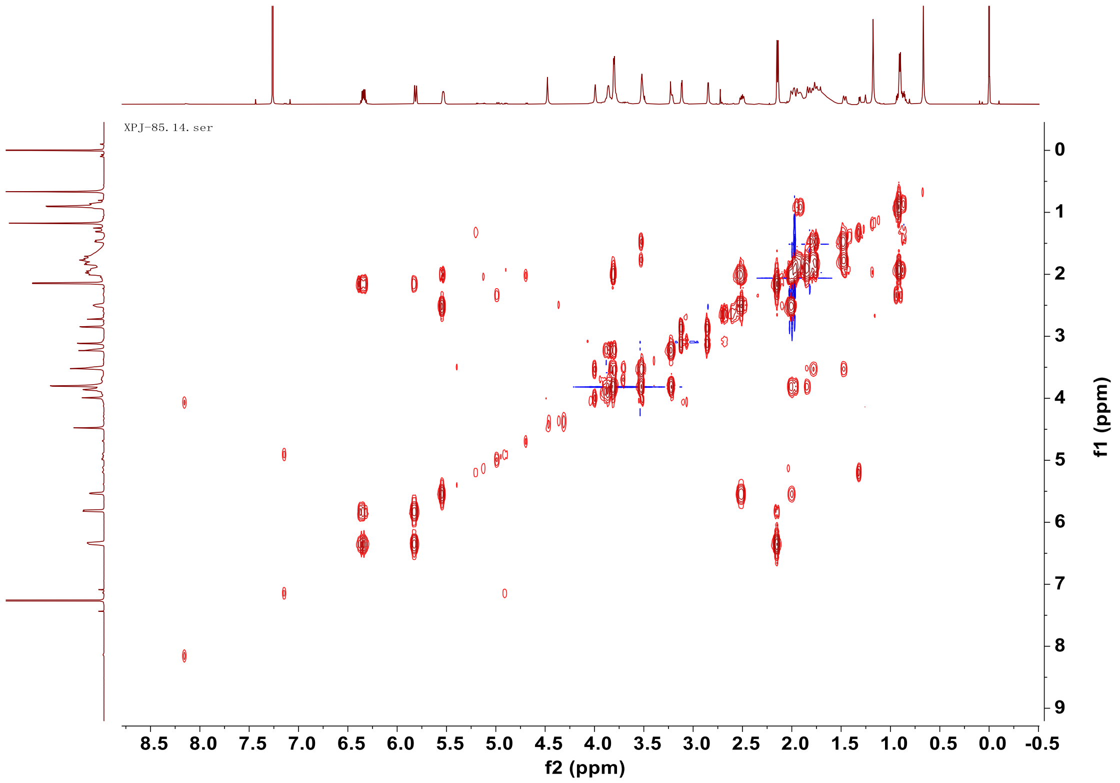

Figure S28. ^{1}H–^{1}H COSY spectrum of **3**

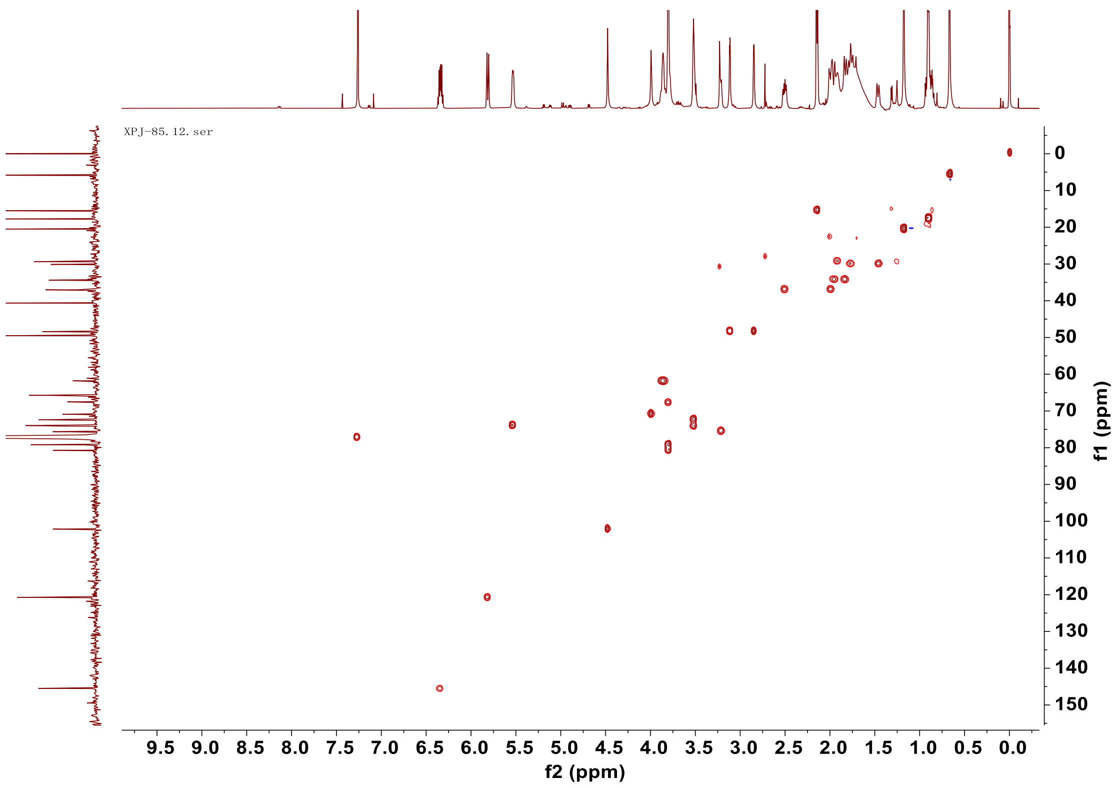

Figure S29. HSQC spectrum of **3**

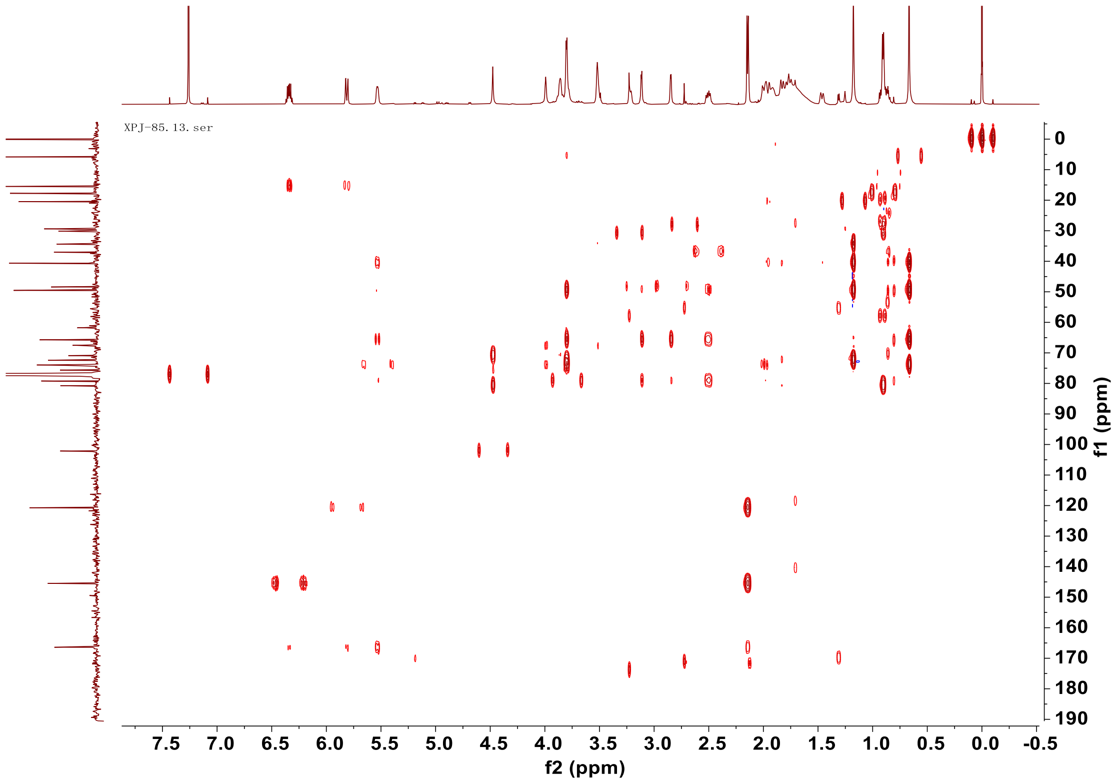

Figure S30. HMBC spectrum of **3**

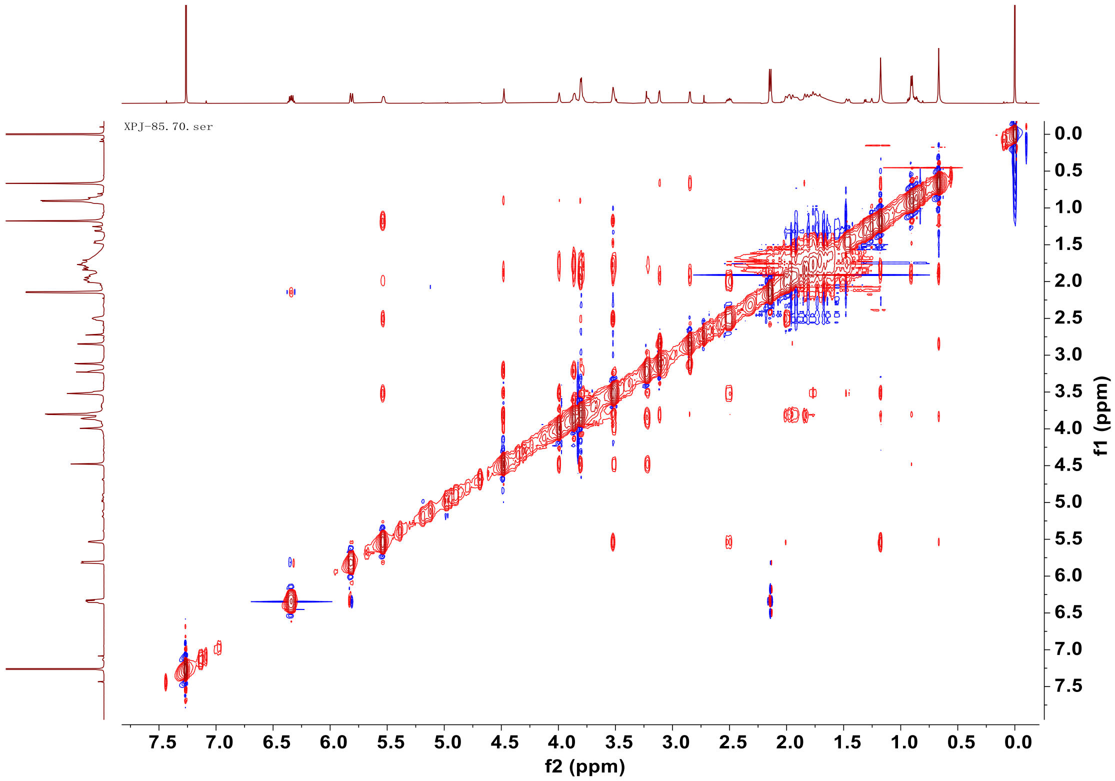

Figure S31. NOESY spectrum of **3**

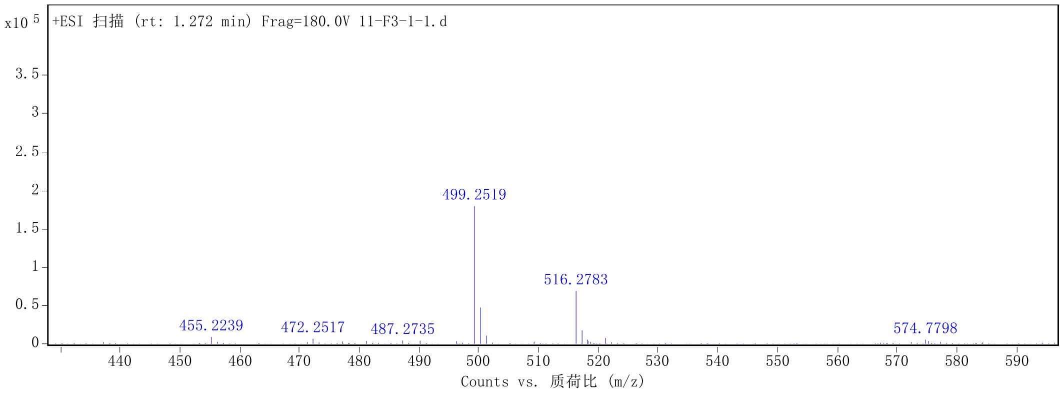

Figure S32. HRESIMS spectrum of **3**

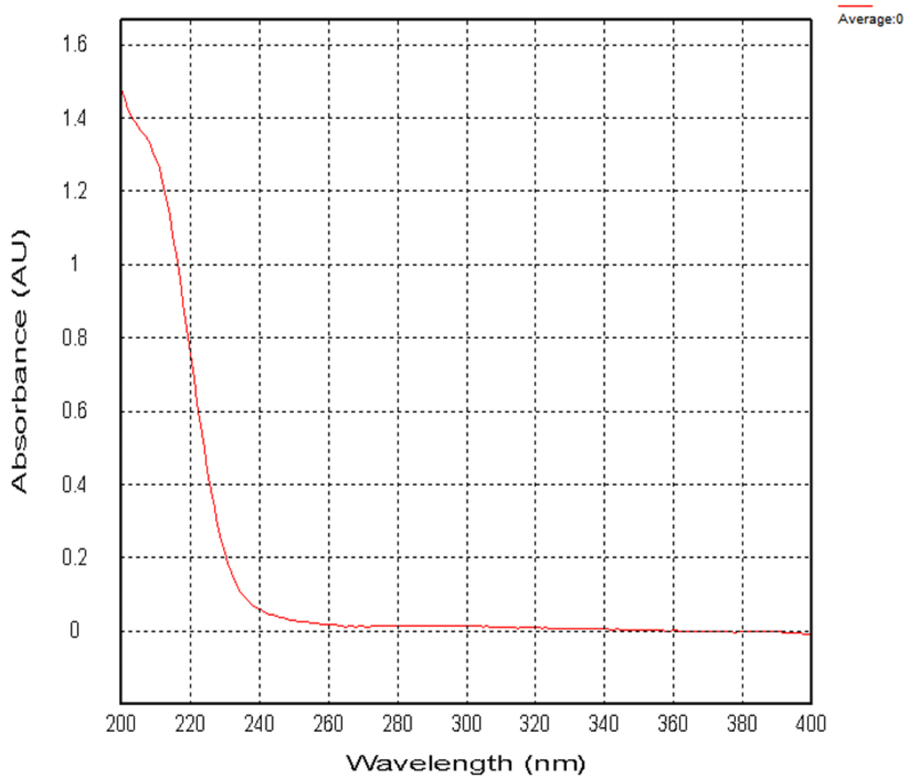

Figure S33. UV spectrum of **3**

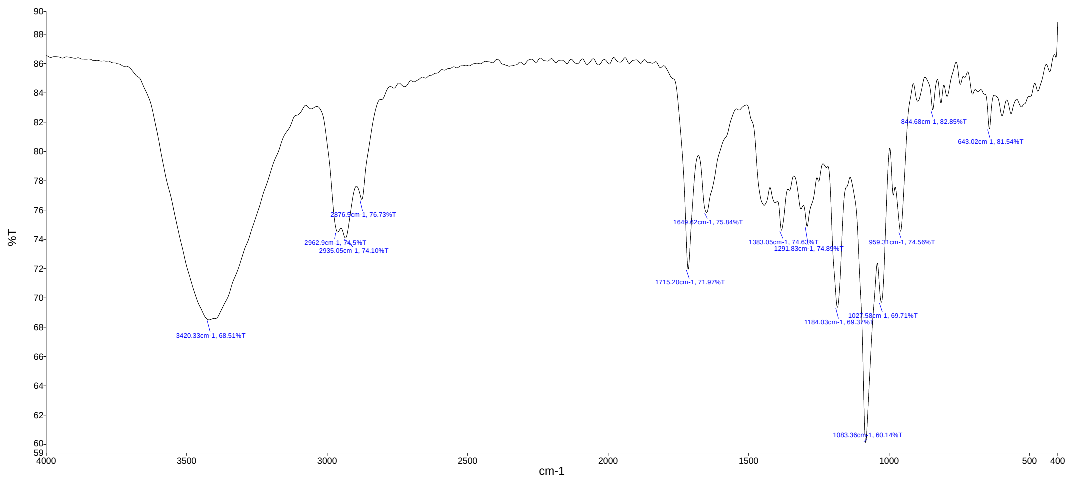

Figure S34. IR spectrum of **3**

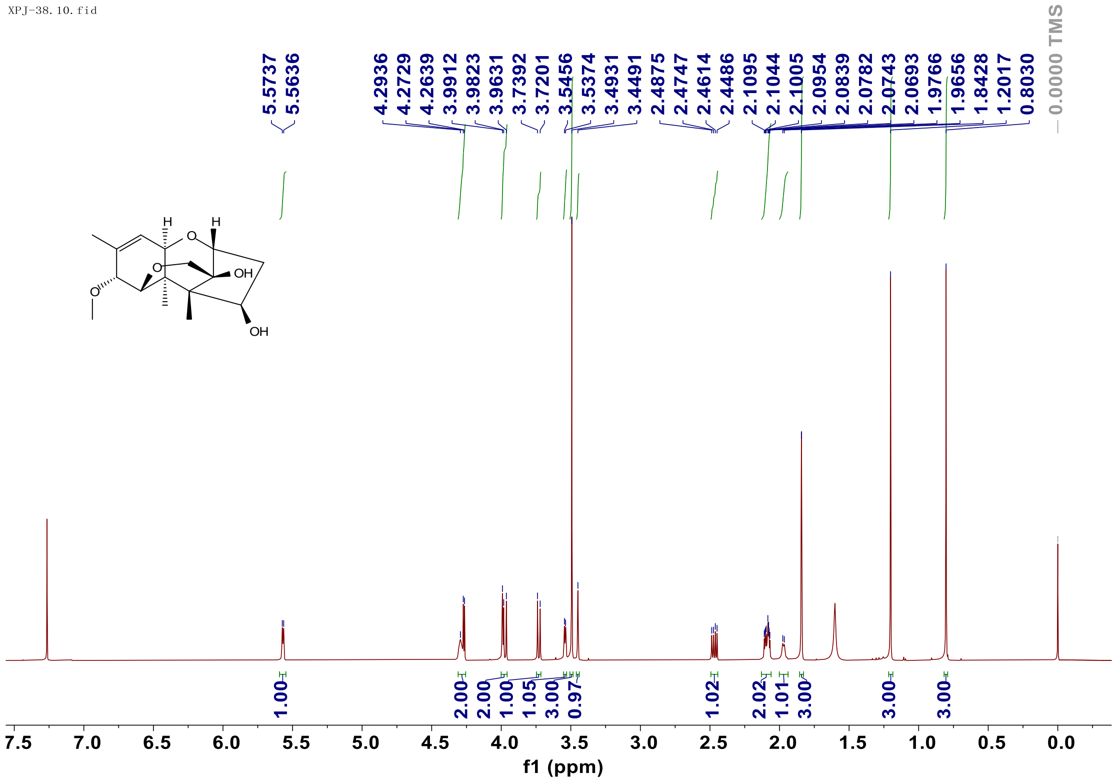

Figure S35. ^{1}H NMR spectrum of **4** (600 MHz, chloroform-*d*)

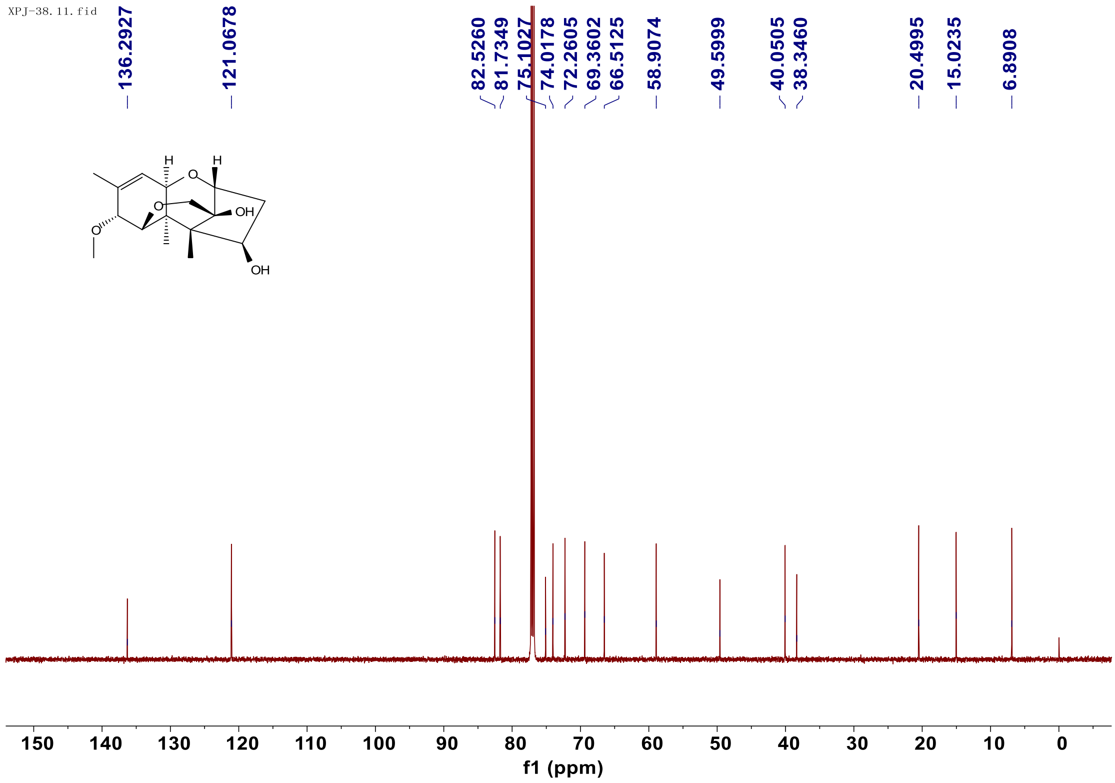

Figure S36. ^{13}C NMR spectrum of **4** (150 MHz, chloroform-*d*)

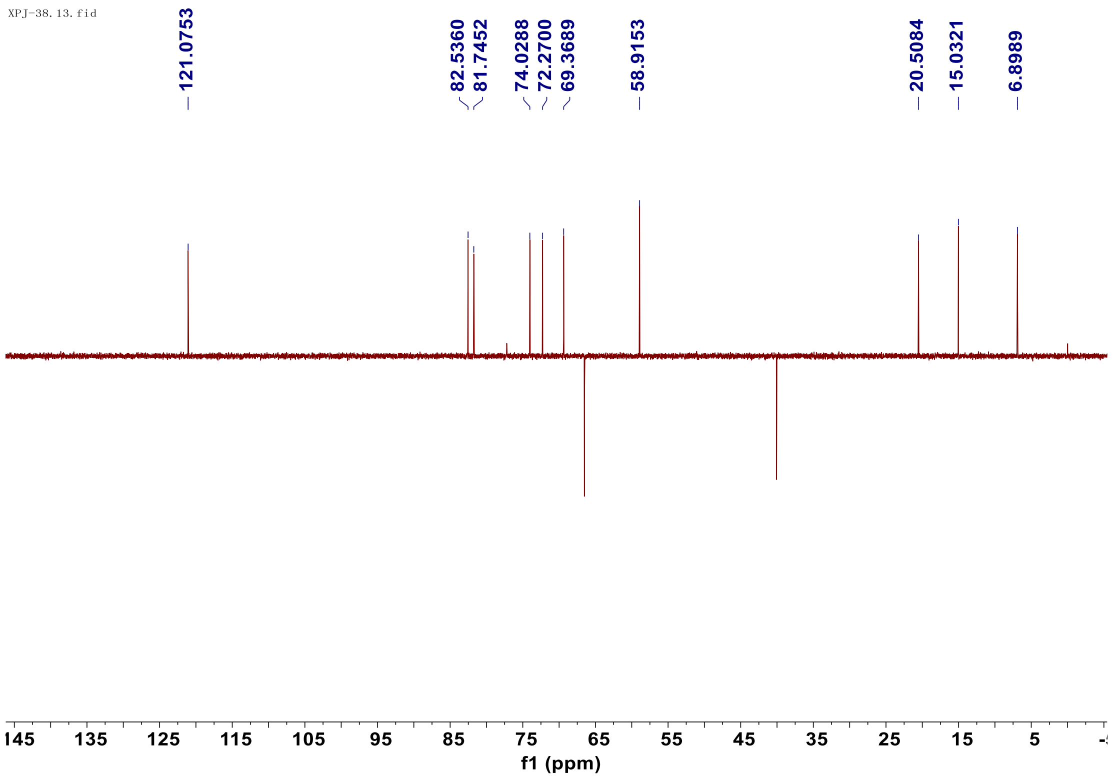

Figure S37. DEPT-135 spectrum of **4** (150 MHz, chloroform-*d*)

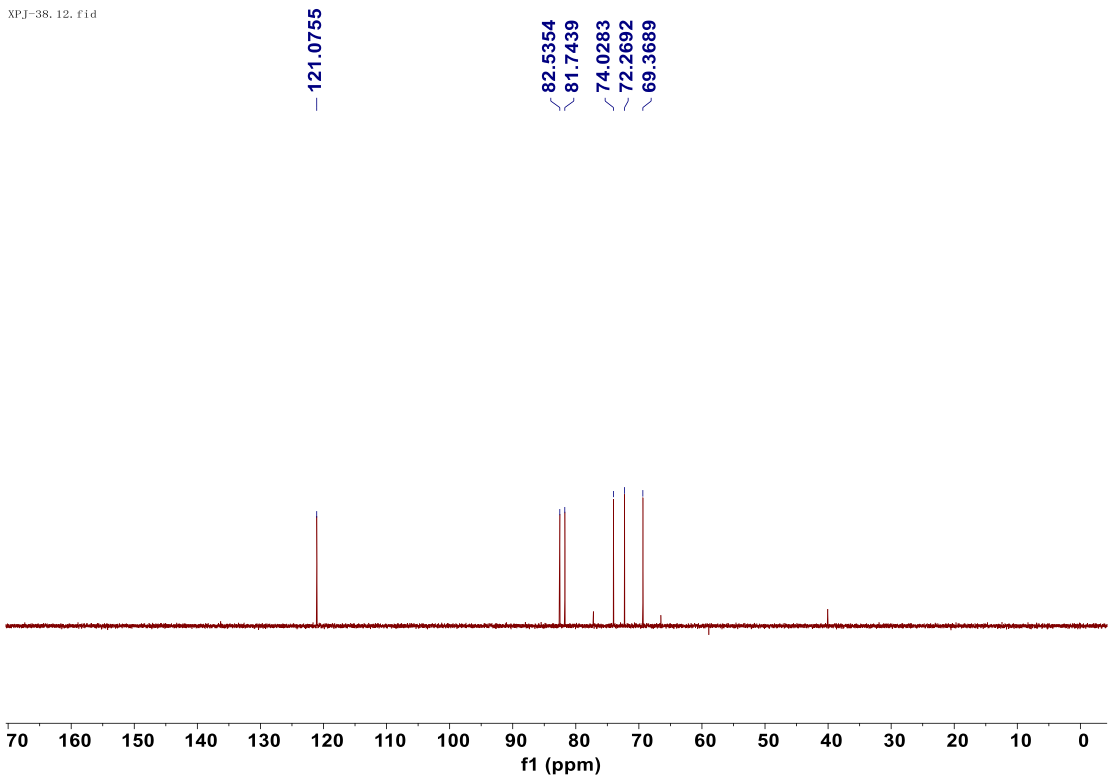

Figure S38. DEPT-90 spectrum of **4** (150 MHz, chloroform-*d*)

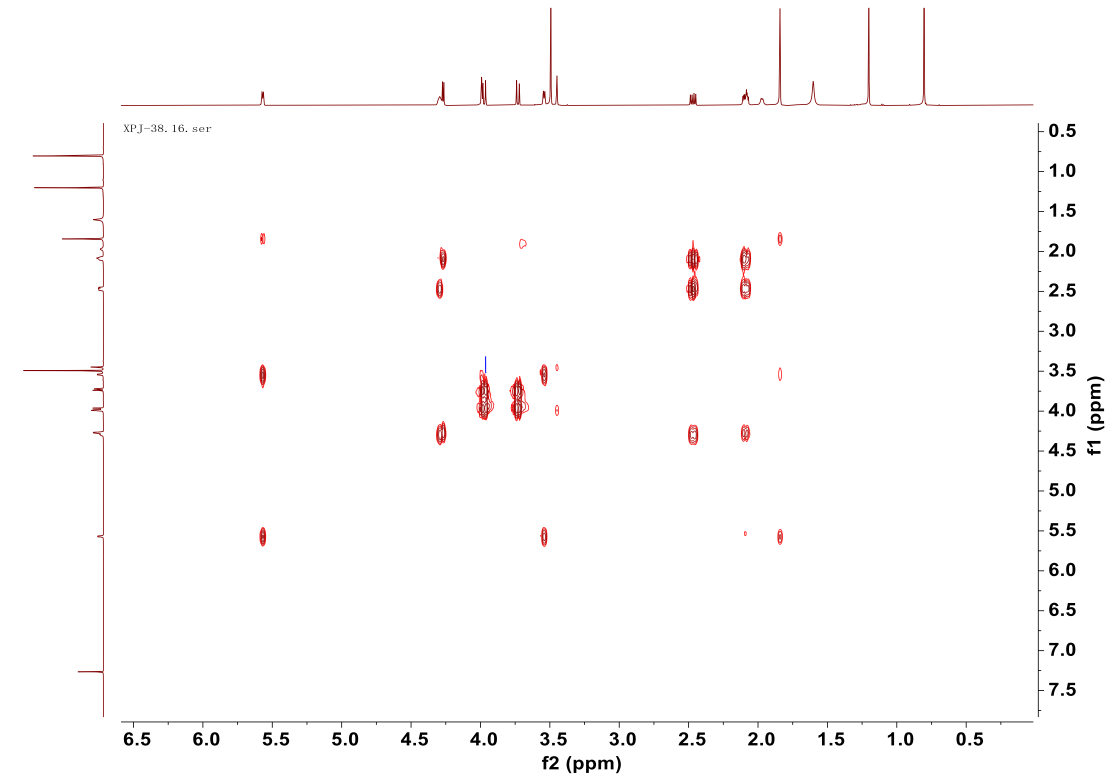

Figure S39. $^{1}H-^{1}H$ COSY spectrum of **4**

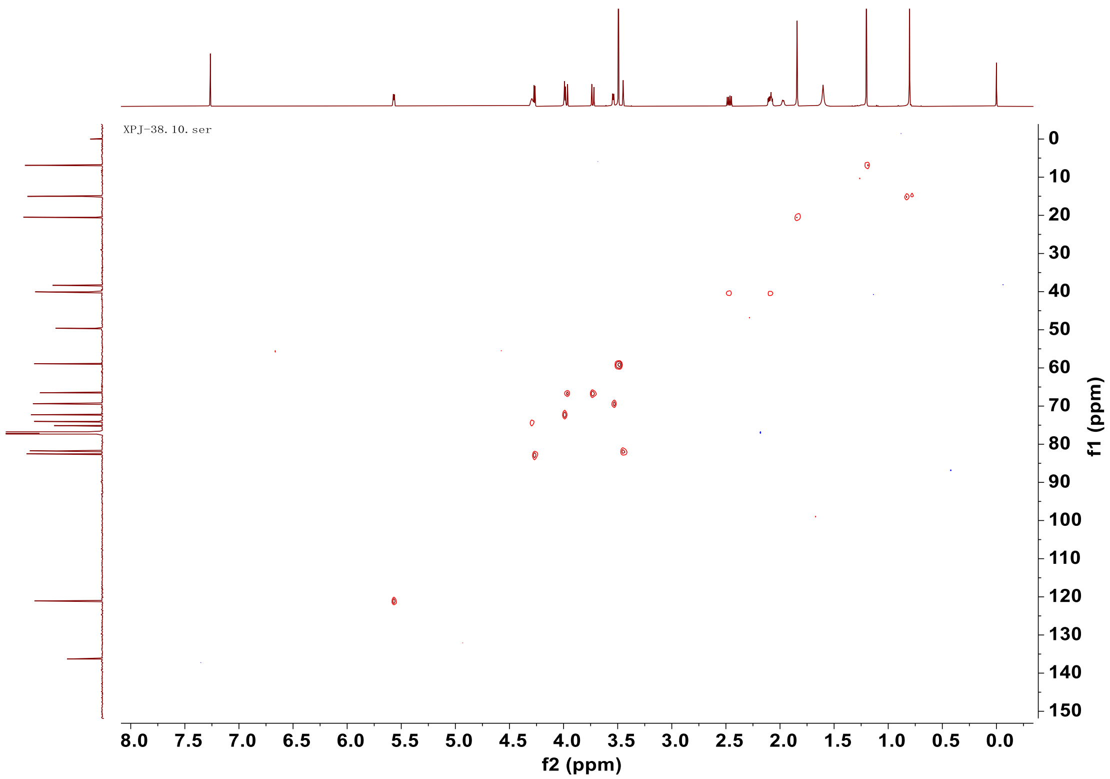

Figure S40. HSQC spectrum of **4**

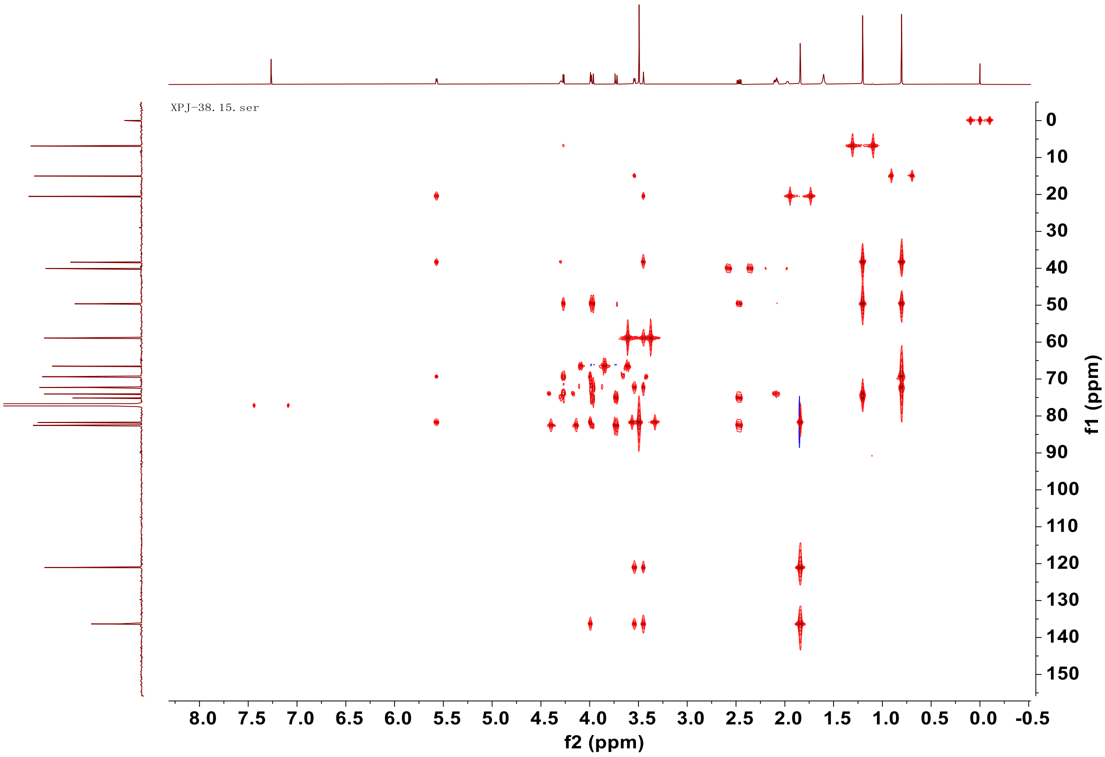

Figure S41. HMBC spectrum of **4**

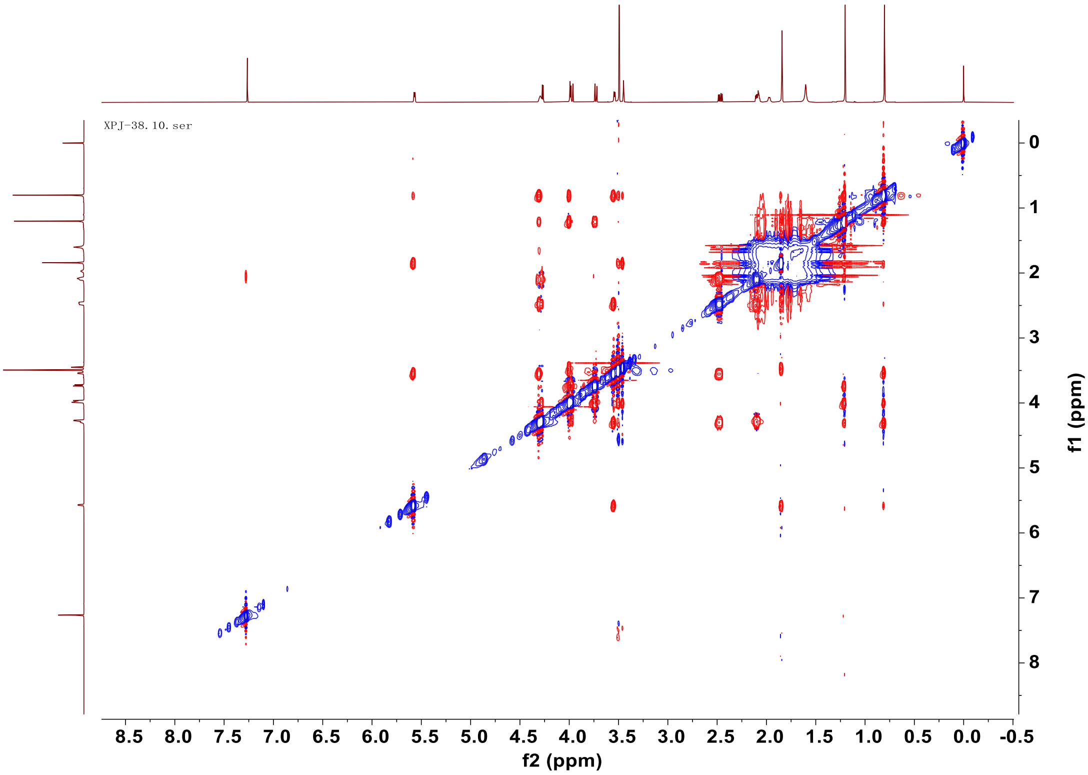

Figure S42. NOESY spectrum of **4**

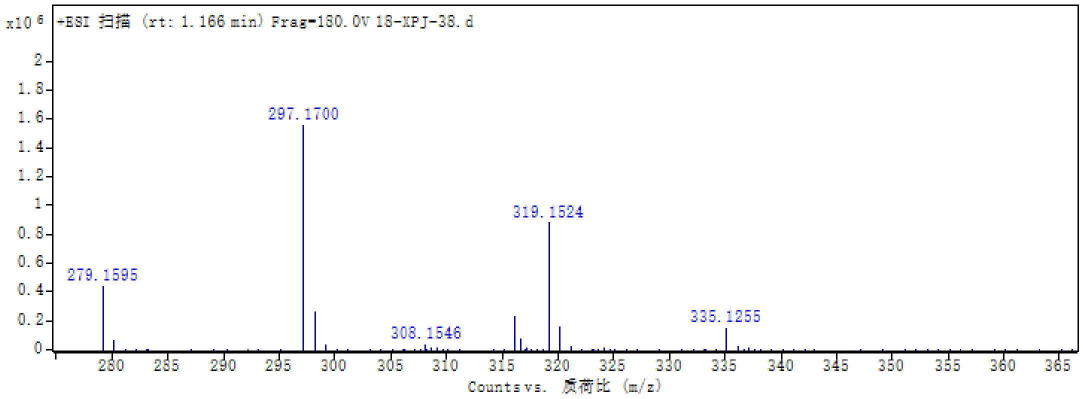

Figure S43. HRESIMS spectrum of **4**

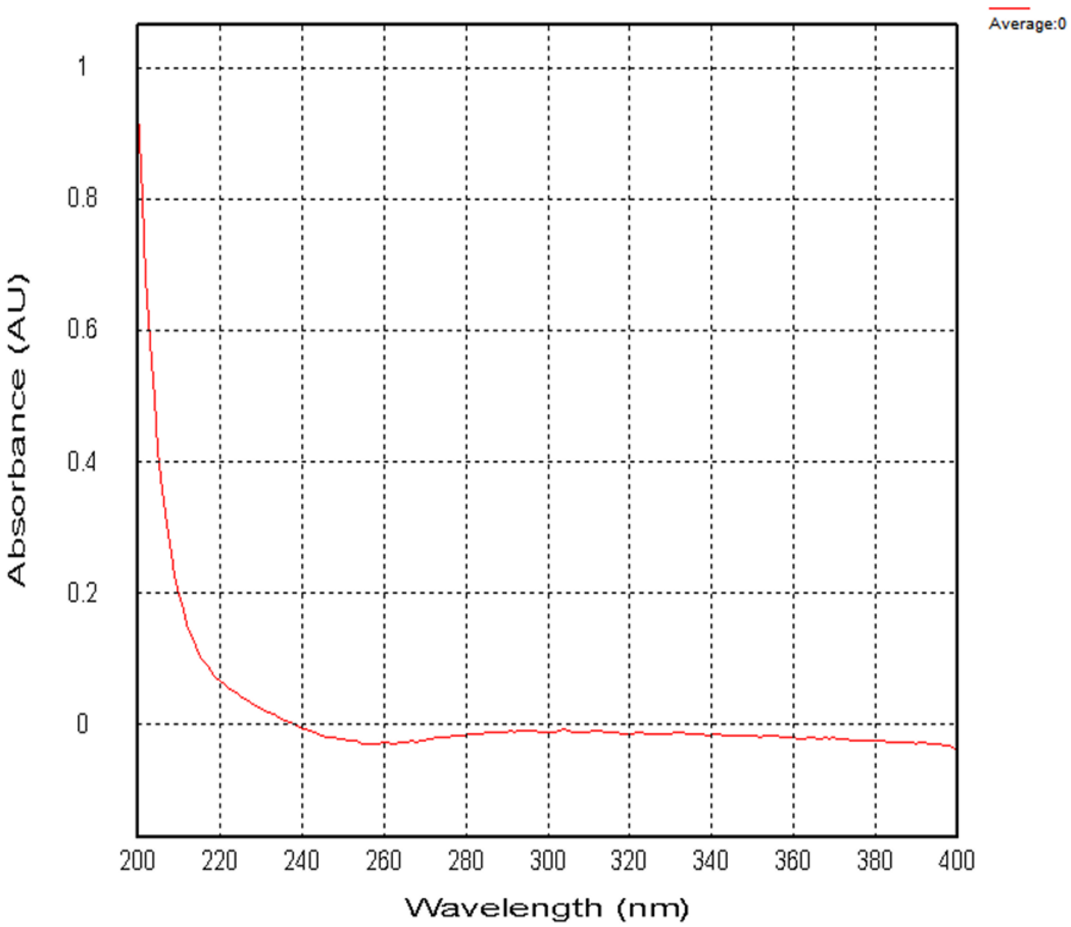

Figure S44. UV spectrum of **4**

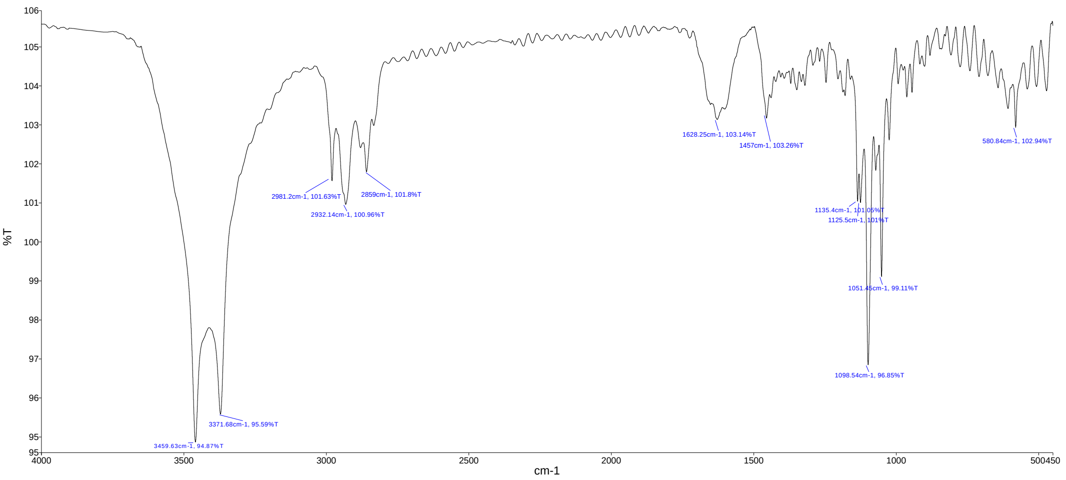

Figure S45. IR spectrum of **4**

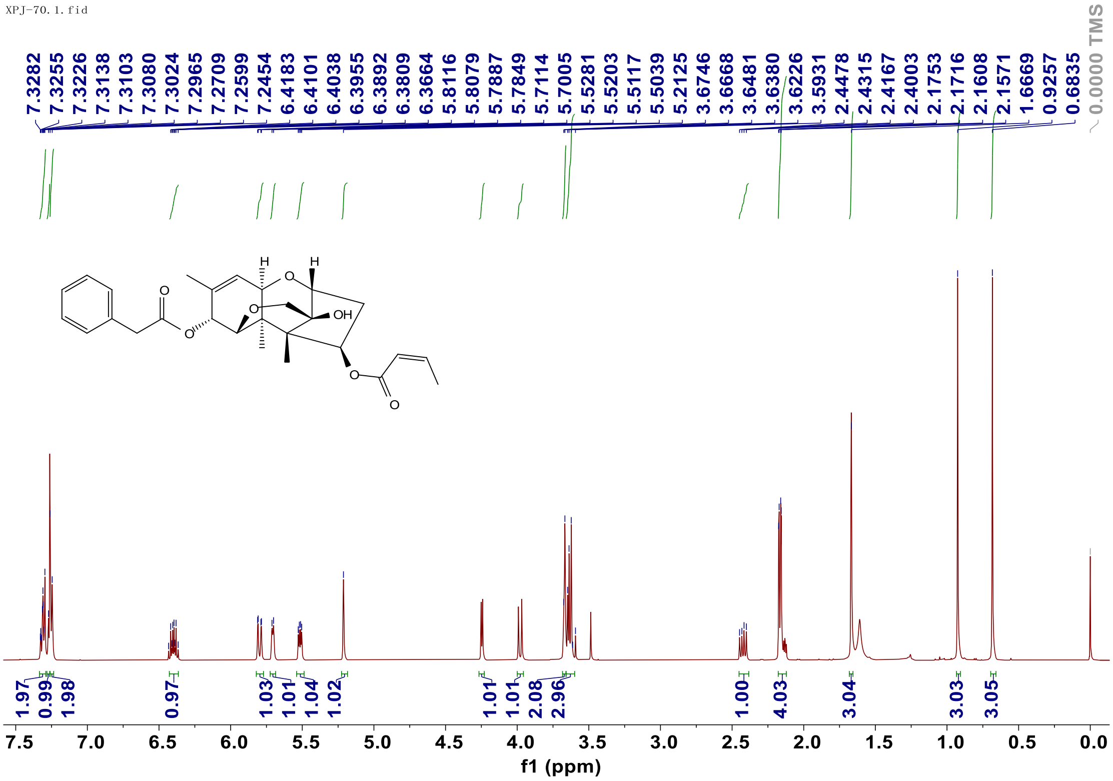

Figure S46. ^{1}H NMR spectrum of **5** (600 MHz, chloroform-*d*)

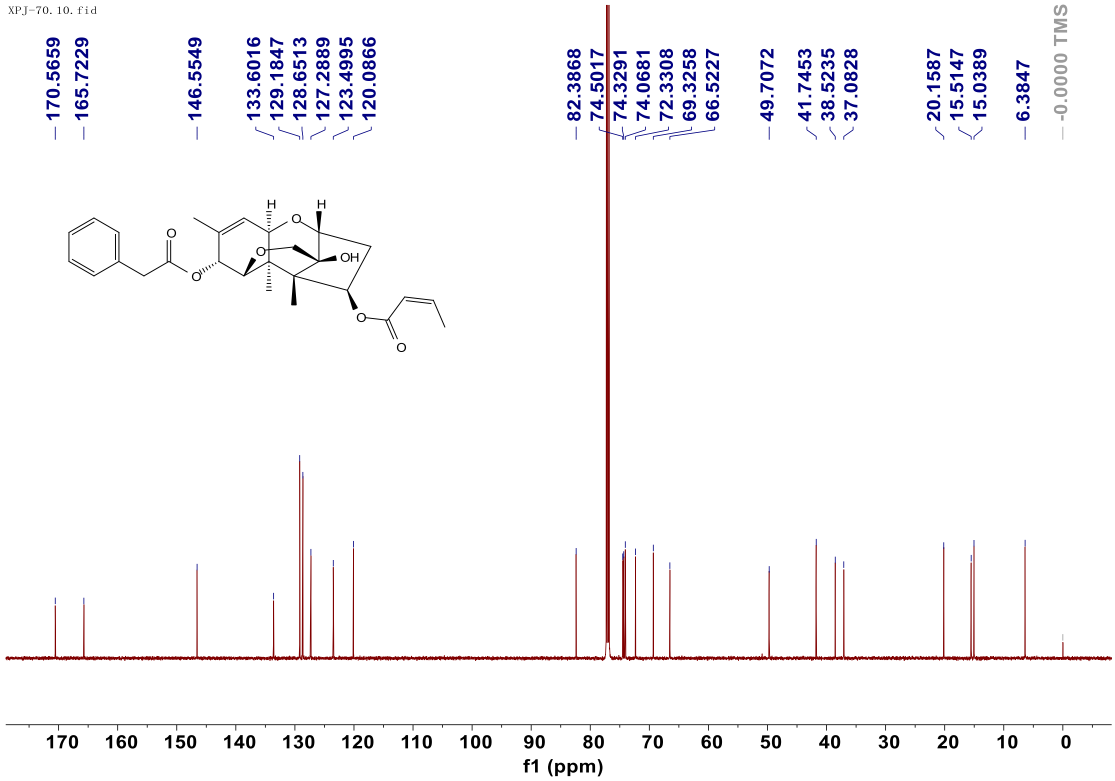

Figure S47. ^{13}C NMR spectrum of **5** (150 MHz, chloroform-*d*)

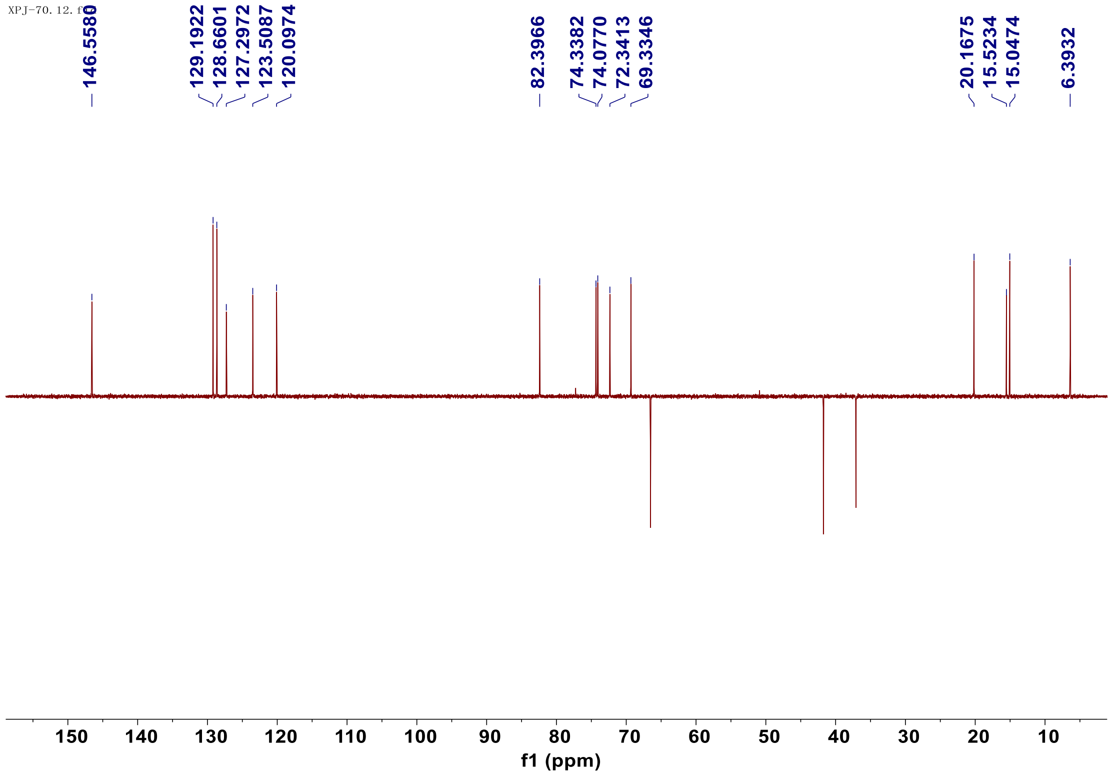

Figure S48. DEPT-135 spectrum of **5** (150 MHz, chloroform-*d*)

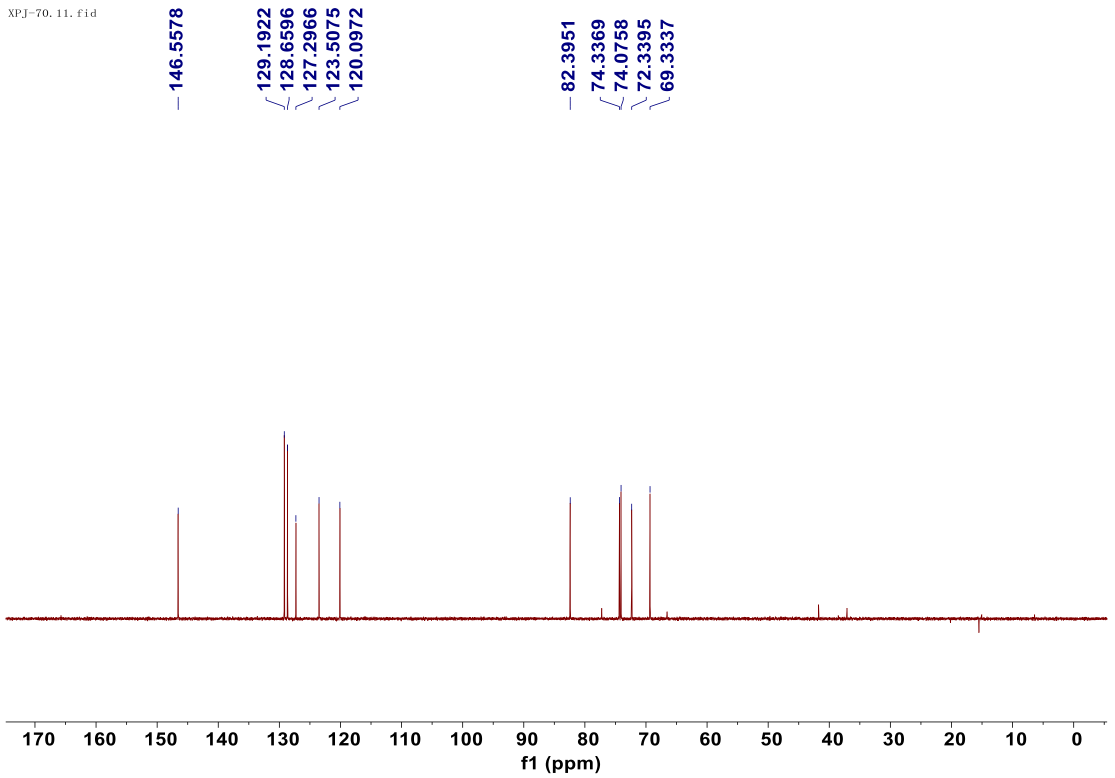

Figure S49. DEPT-90 spectrum of **5** (150 MHz, chloroform-*d*)

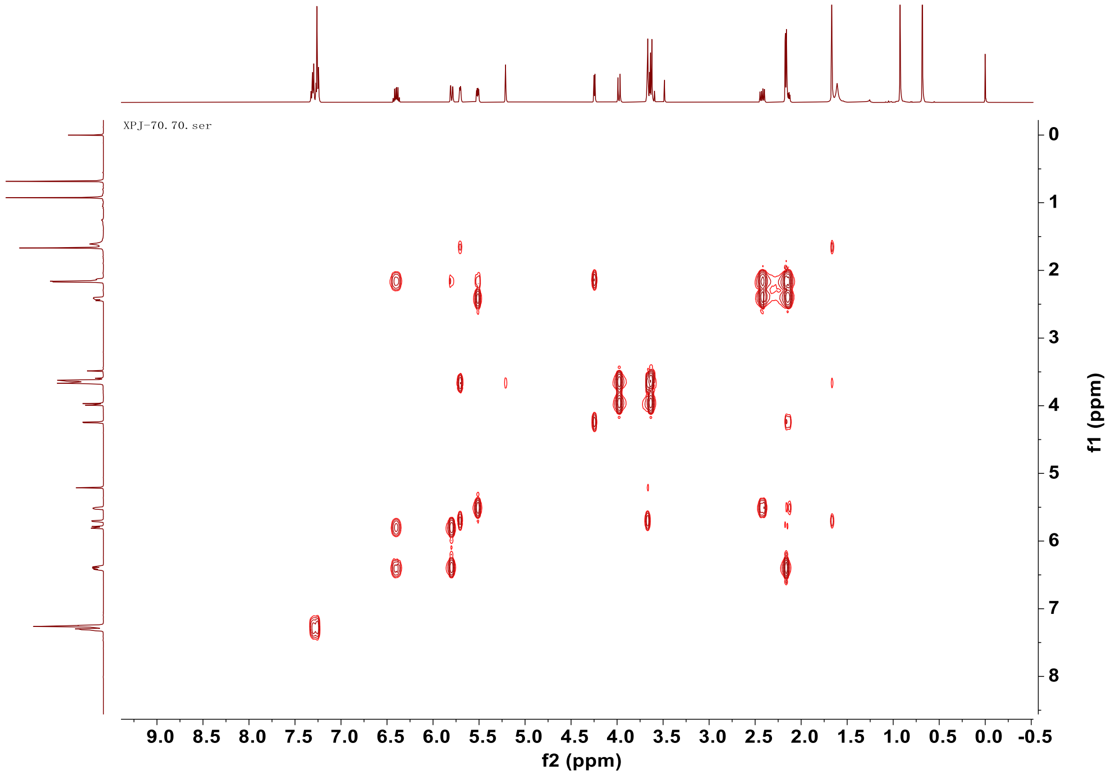

Figure S50. $^{1}H-^{1}H$ COSY spectrum of **5**

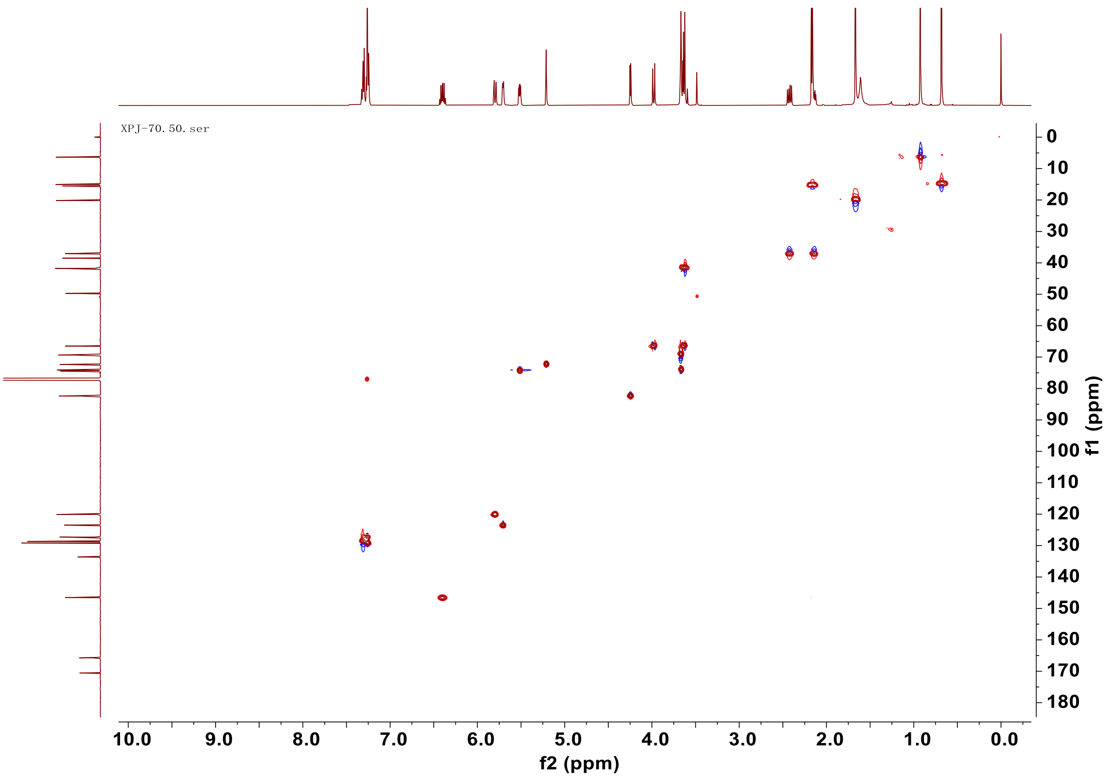

Figure S51. HSQC spectrum of **5**

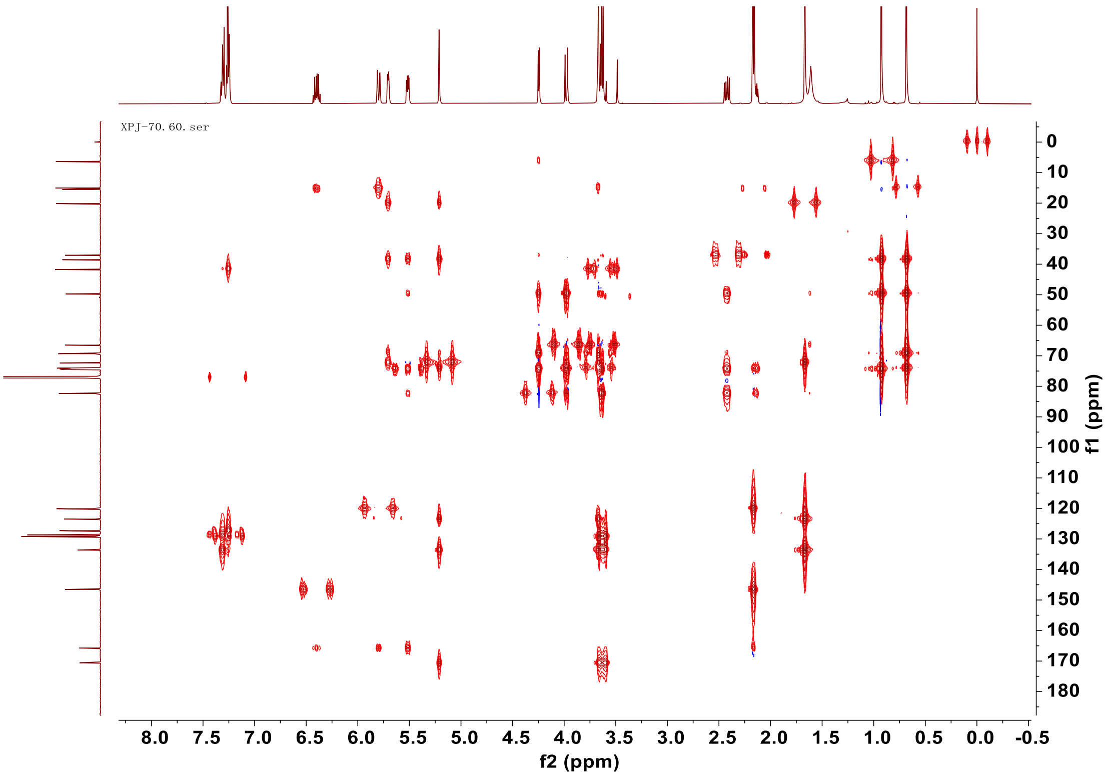

Figure S52. HMBC spectrum of **5**

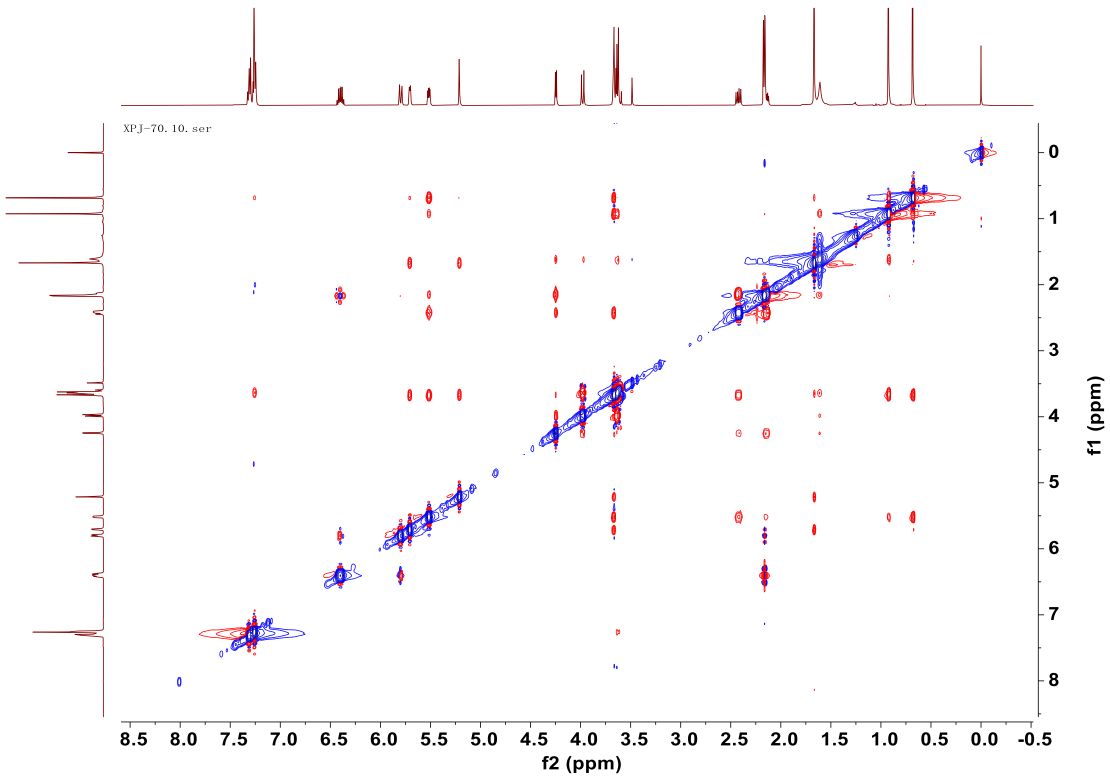

Figure S53. NOESY spectrum of **5**

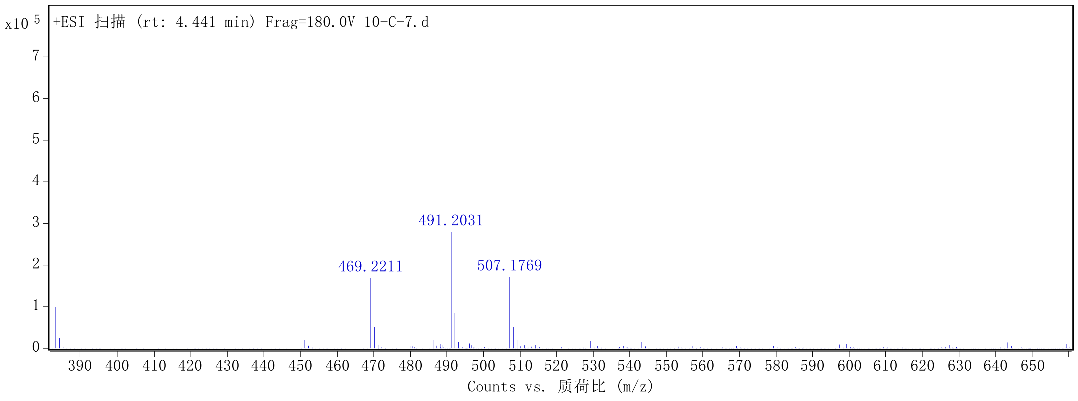

Figure S54. HRESIMS spectrum of **5**

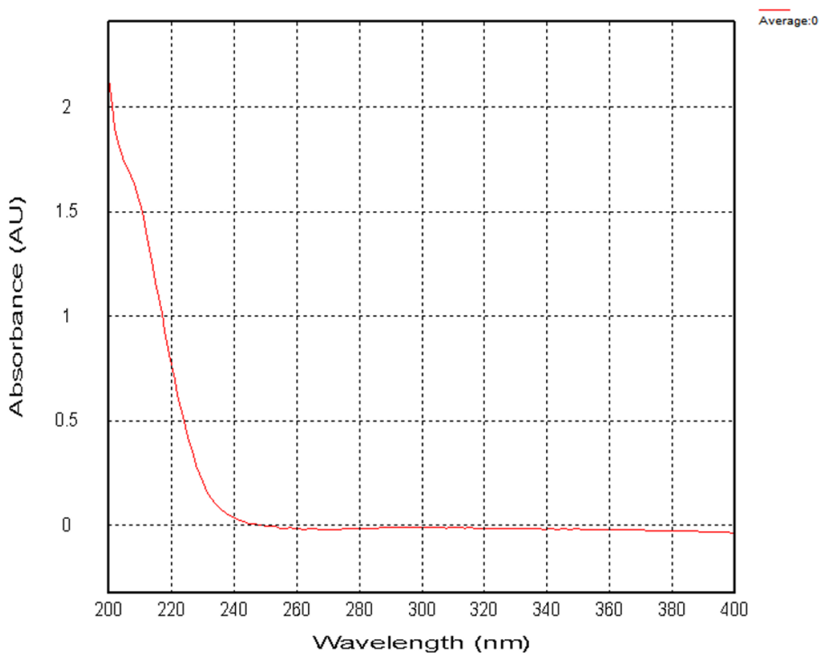

Figure S55. UV spectrum of **5**

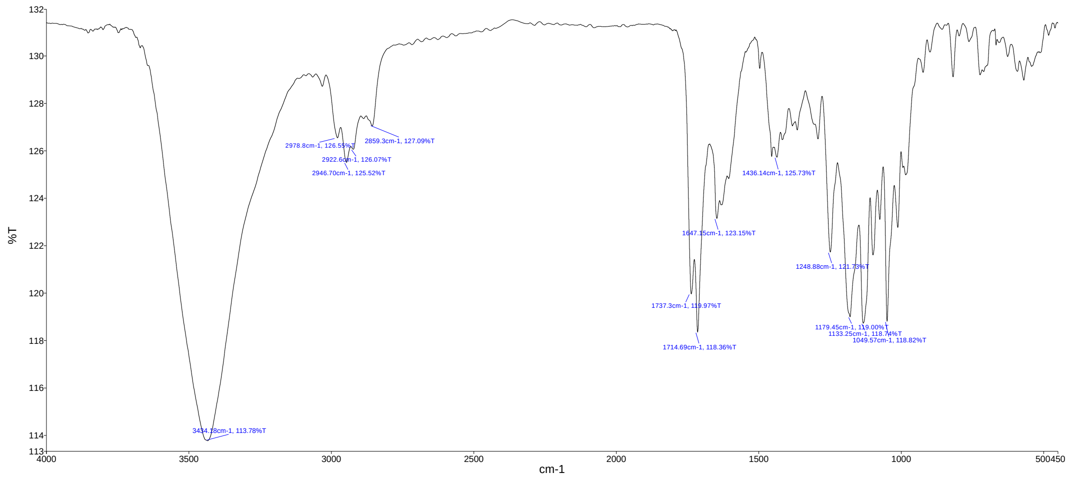

Figure S56. IR spectrum of **5**

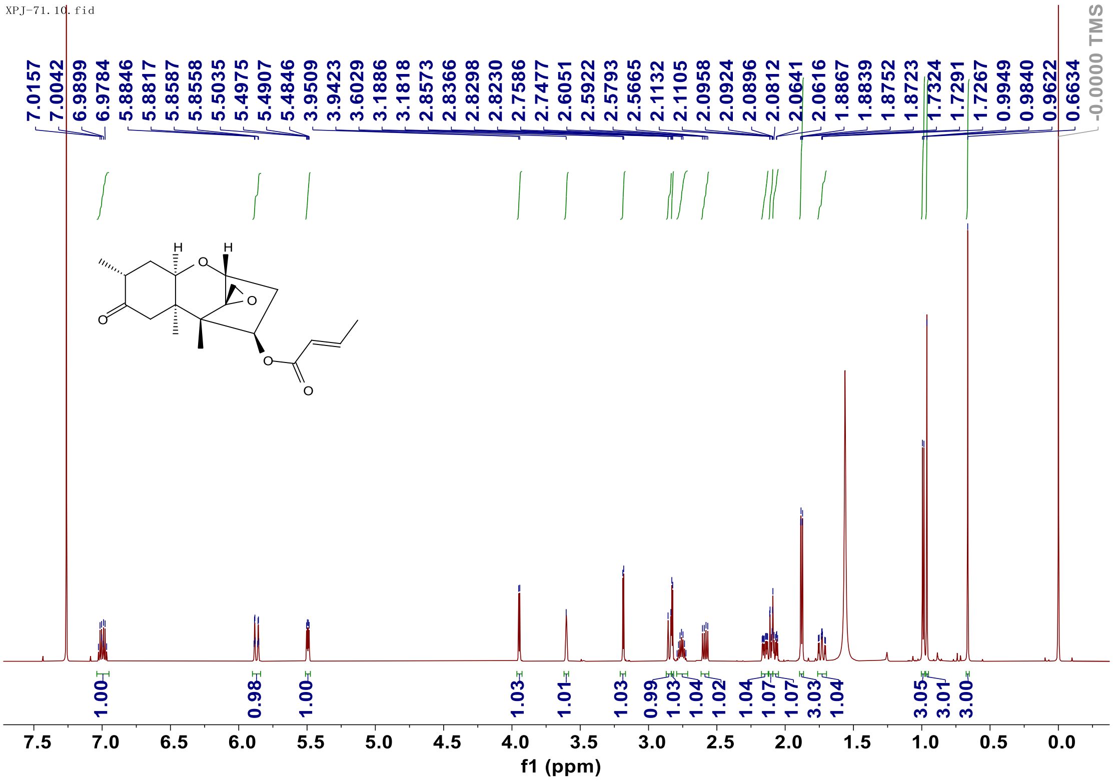

Figure S57. ^{1}H NMR spectrum of **6** (600 MHz, chloroform-*d*)

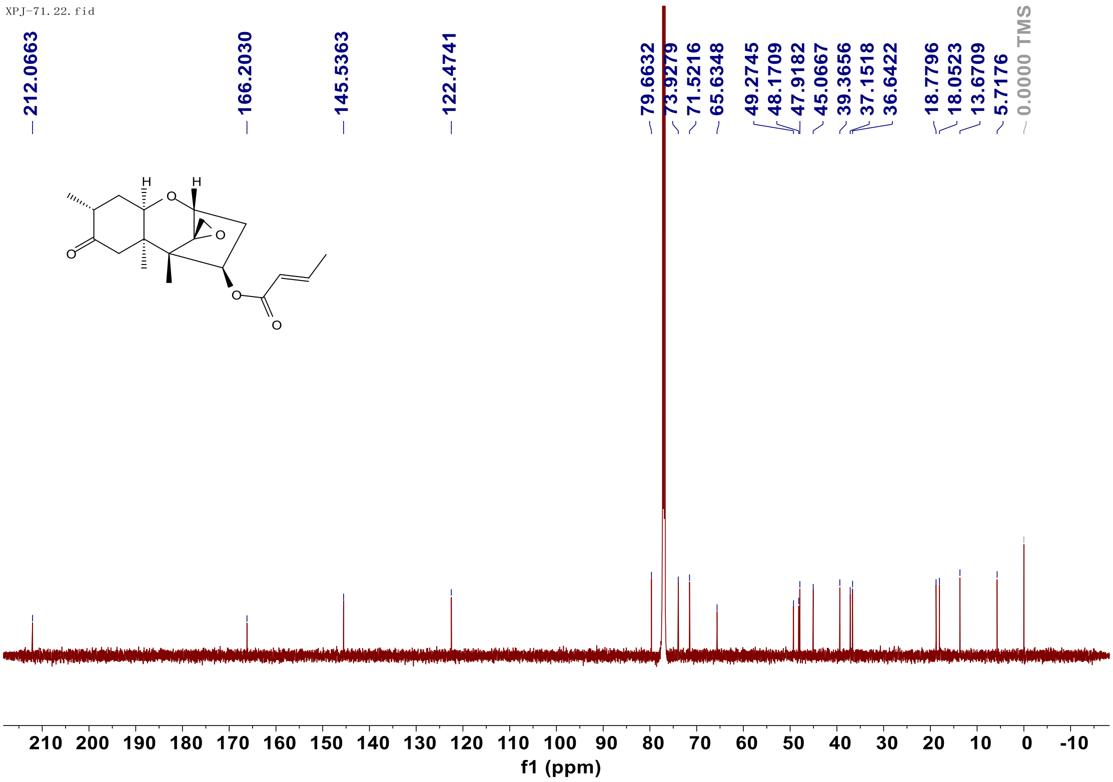

Figure S58. ^{13}C NMR spectrum of **6** (150 MHz, chloroform-*d*)

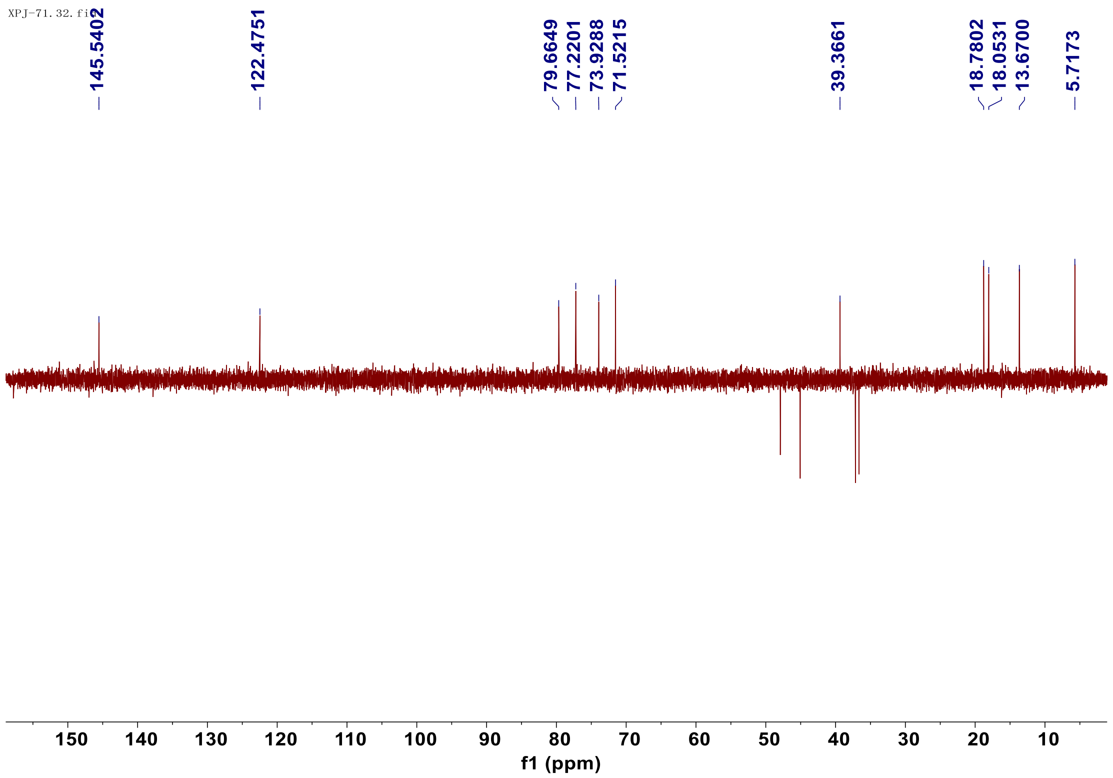

Figure S59. DEPT-135 spectrum of **6** (150 MHz, chloroform-*d*)

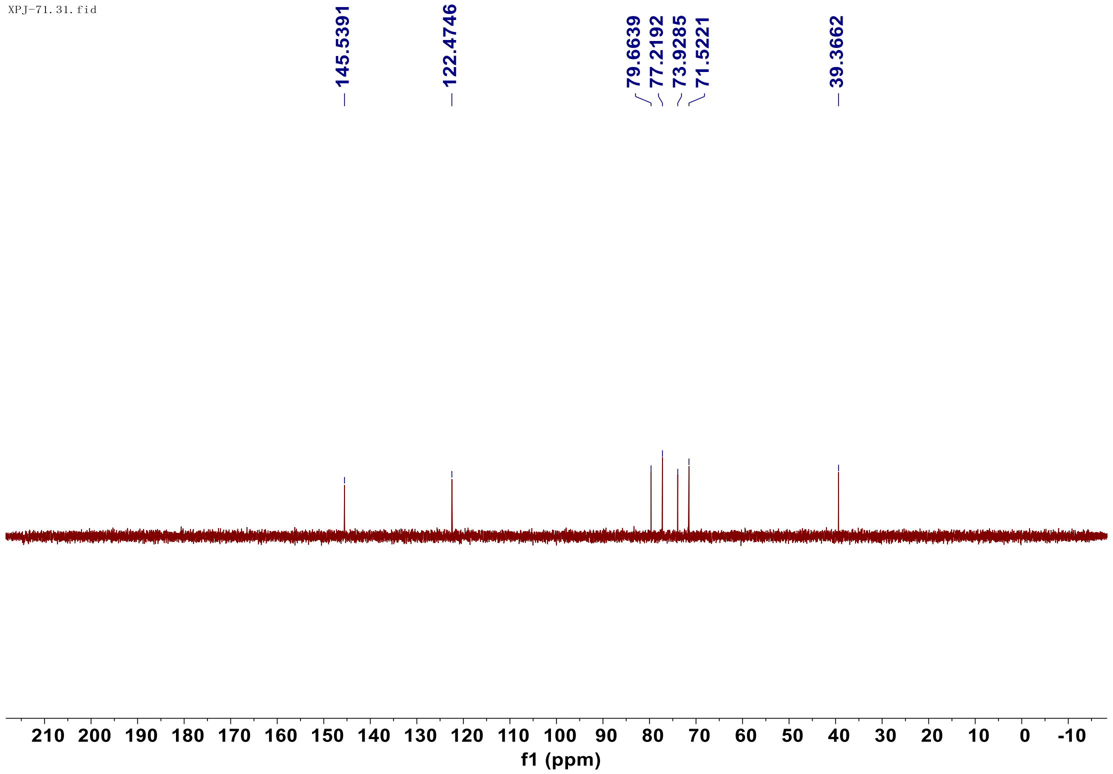

Figure S60. DEPT-90 spectrum of **6** (150 MHz, chloroform-*d*)

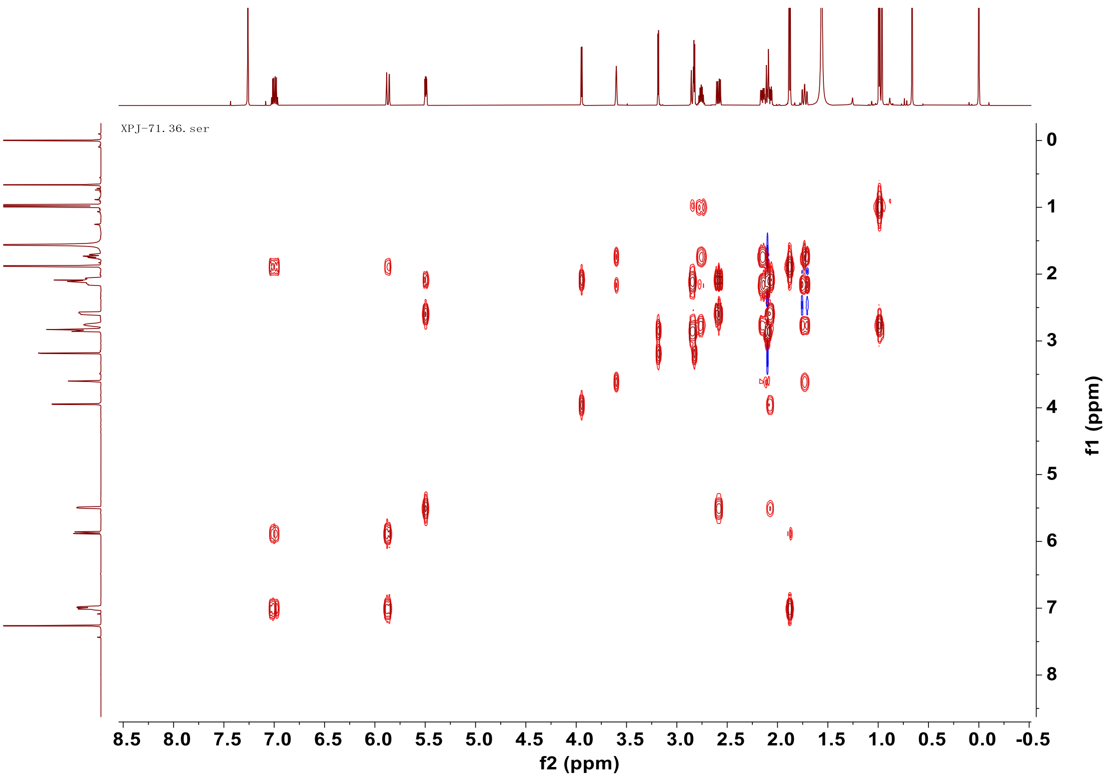

Figure S61. ^{1}H–^{1}H COSY spectrum of **6**

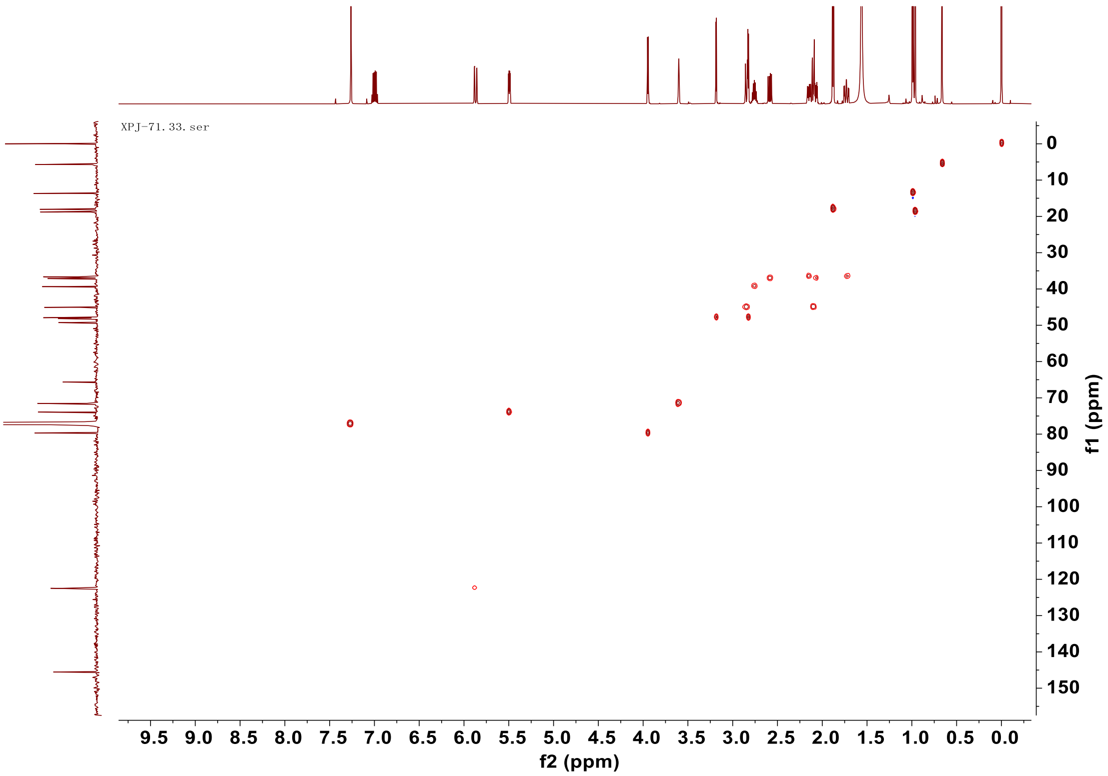

Figure S62. HSQC spectrum of **6**

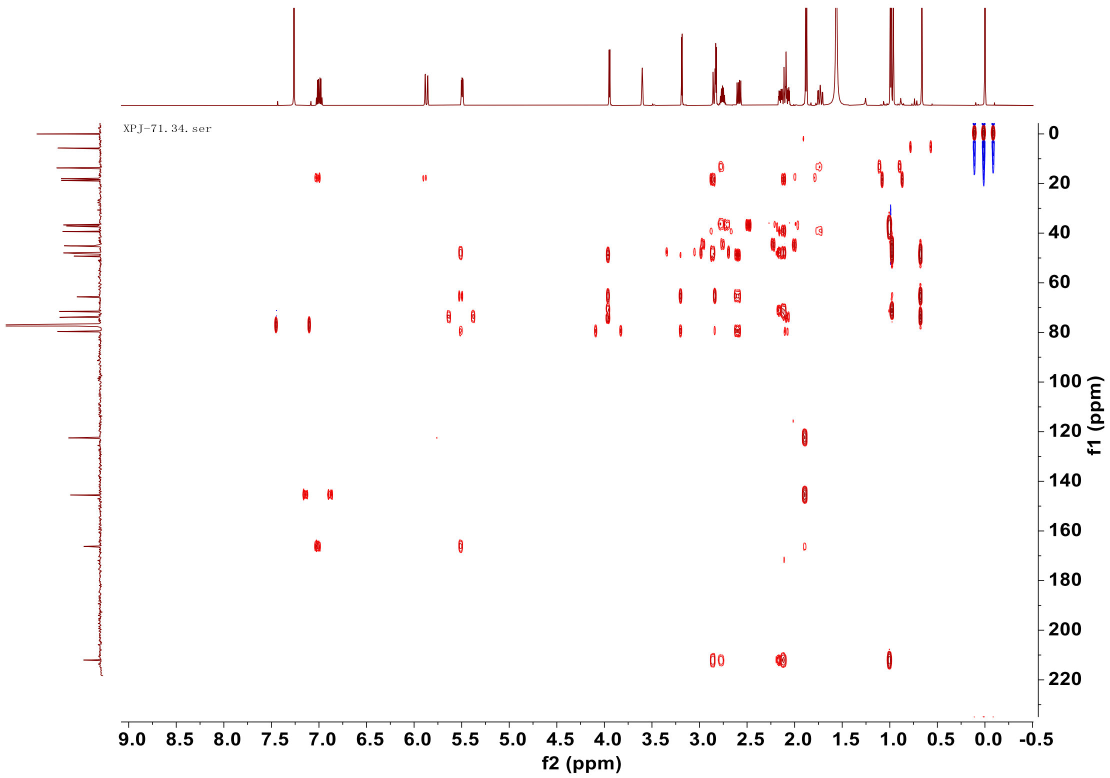

Figure S63. HMBC spectrum of **6**

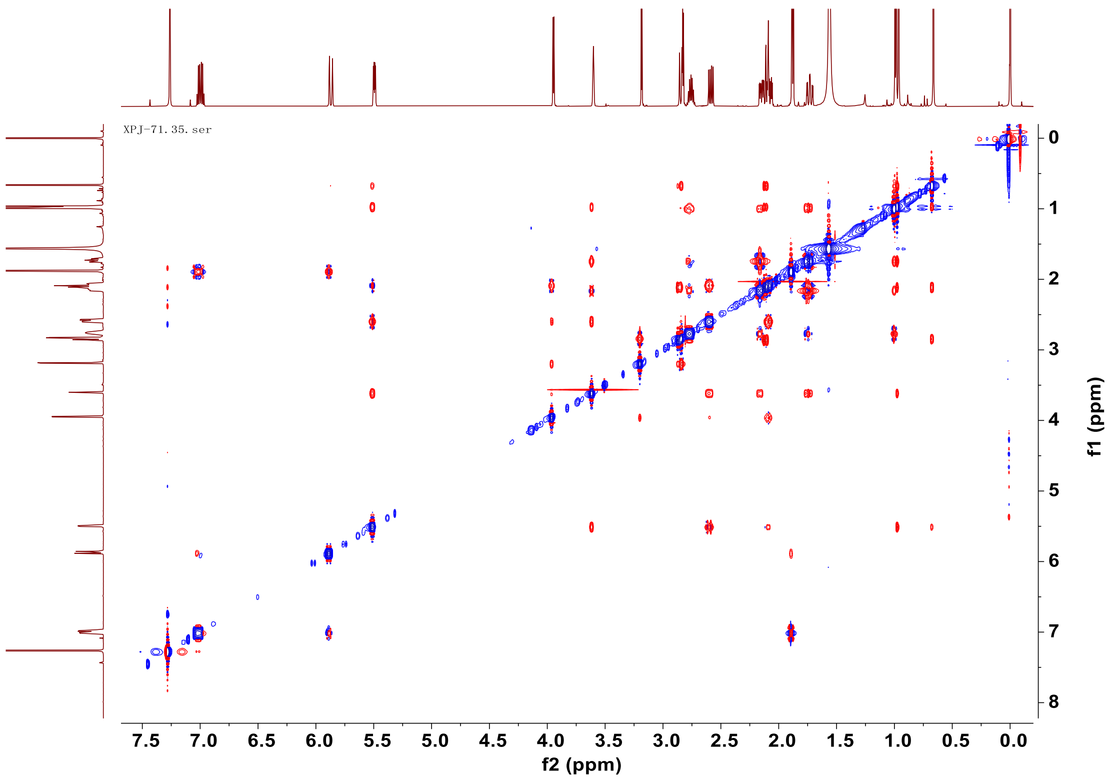

Figure S64. NOESY spectrum of **6**

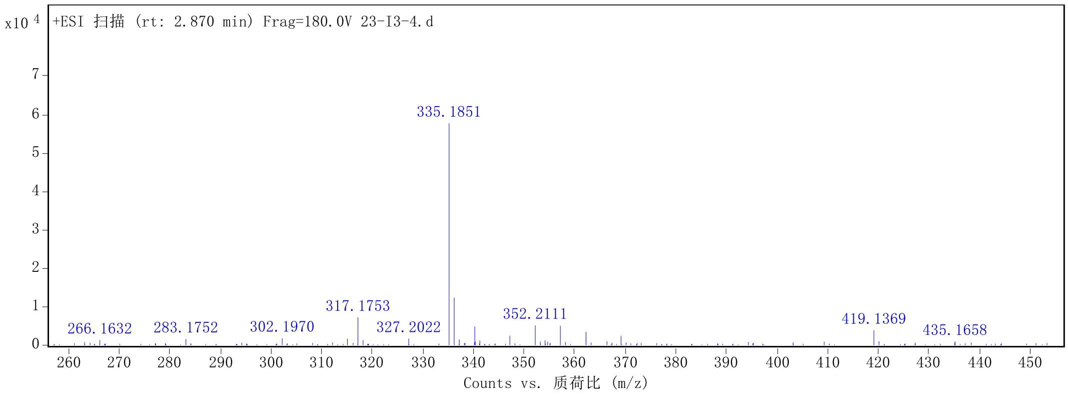

Figure S65. HRESIMS spectrum of **6**

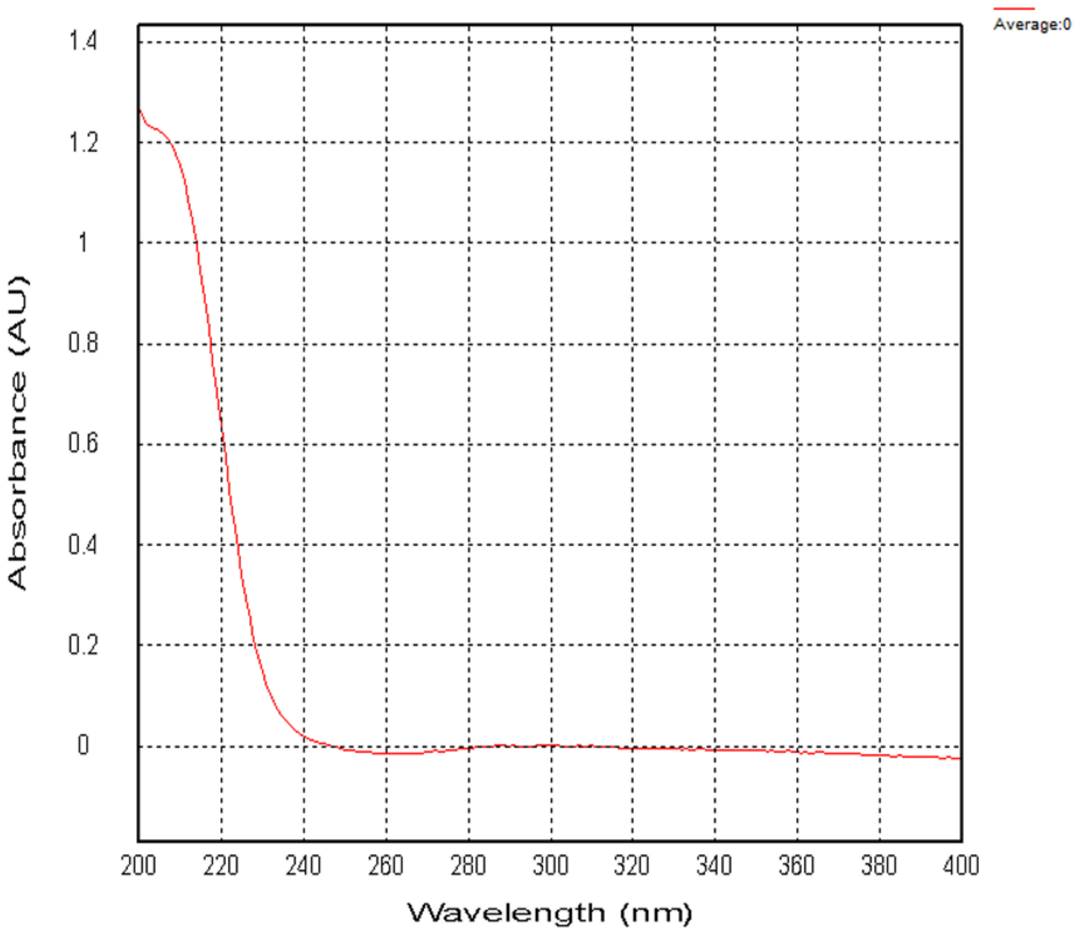

Figure S66. UV spectrum of **6**

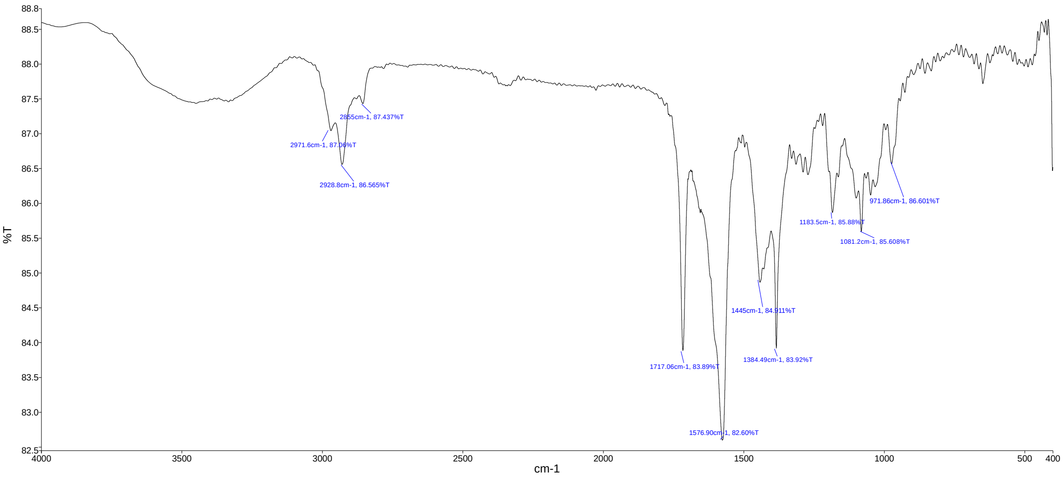

Figure S67. IR spectrum of **6**

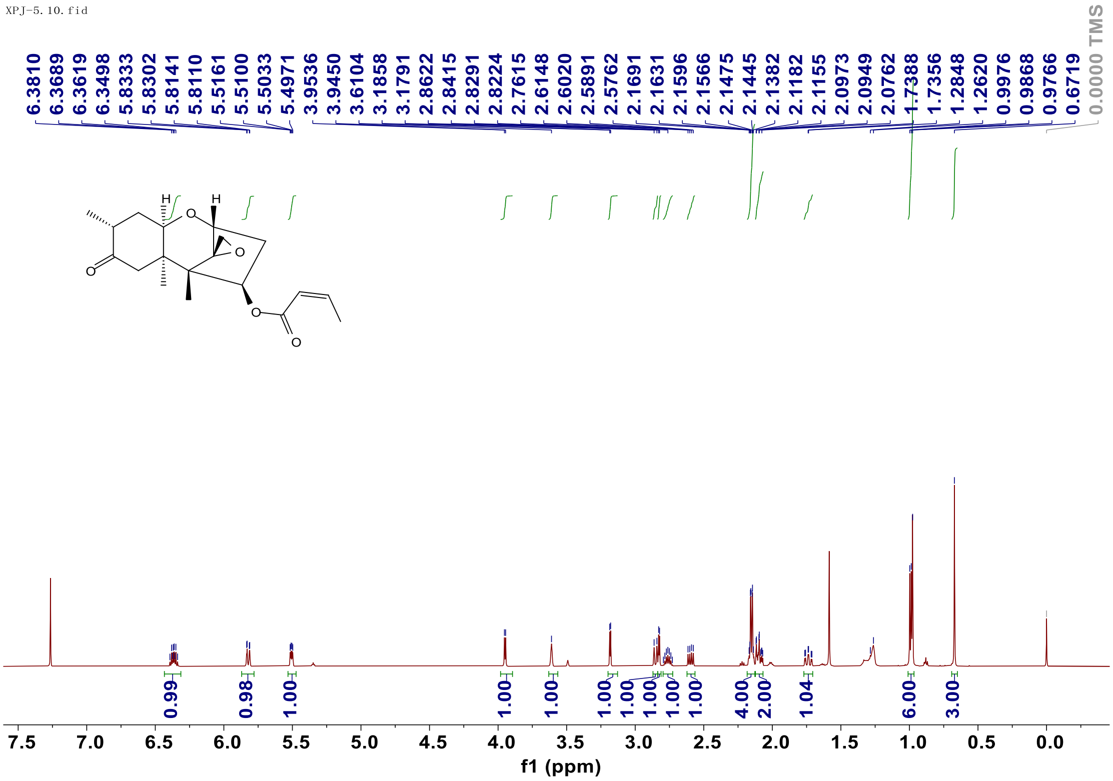

Figure S68. ^{1}H NMR spectrum of **7** (600 MHz, chloroform-*d*)

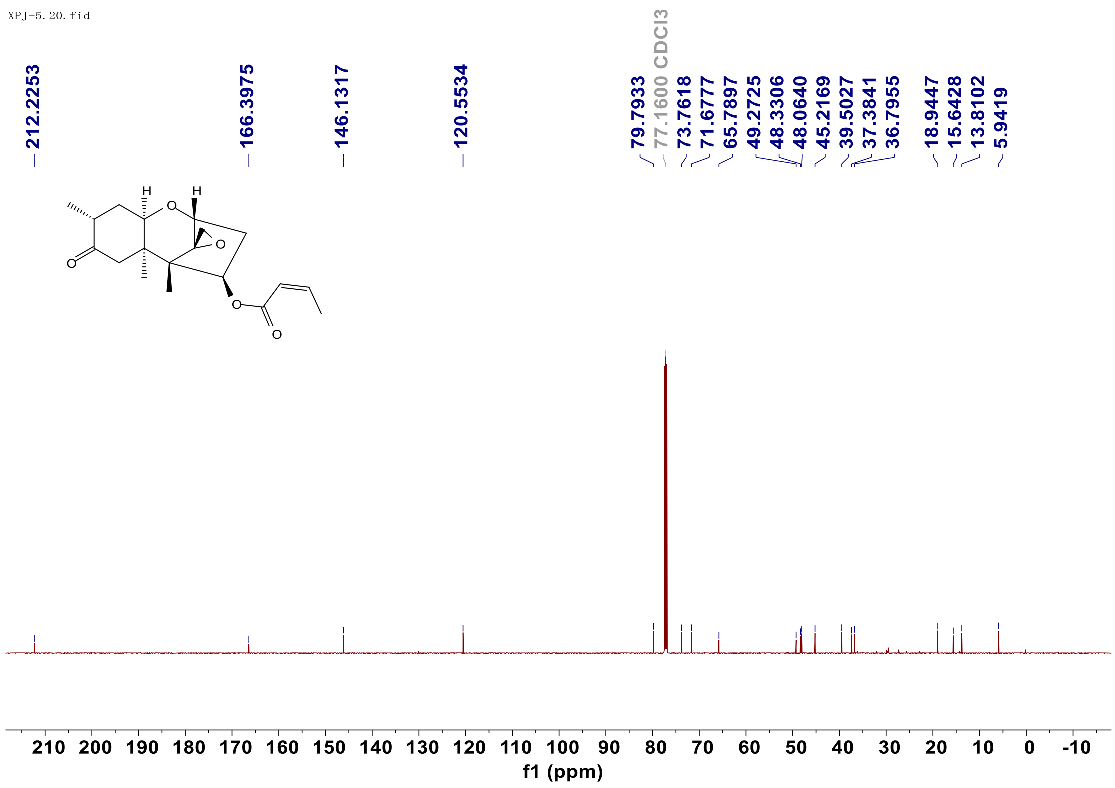

Figure S69. ^{13}C NMR spectrum of **7** (150 MHz, chloroform-*d*)

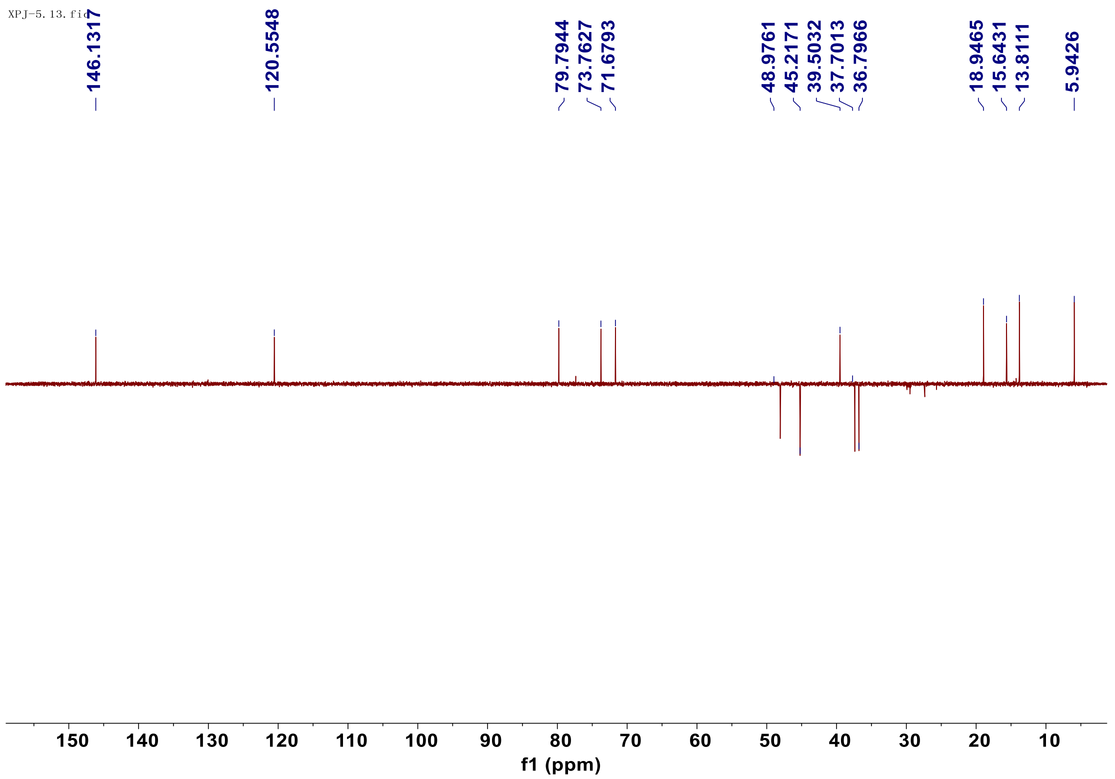

Figure S70. DEPT-135 spectrum of **7** (150 MHz, chloroform-*d*)

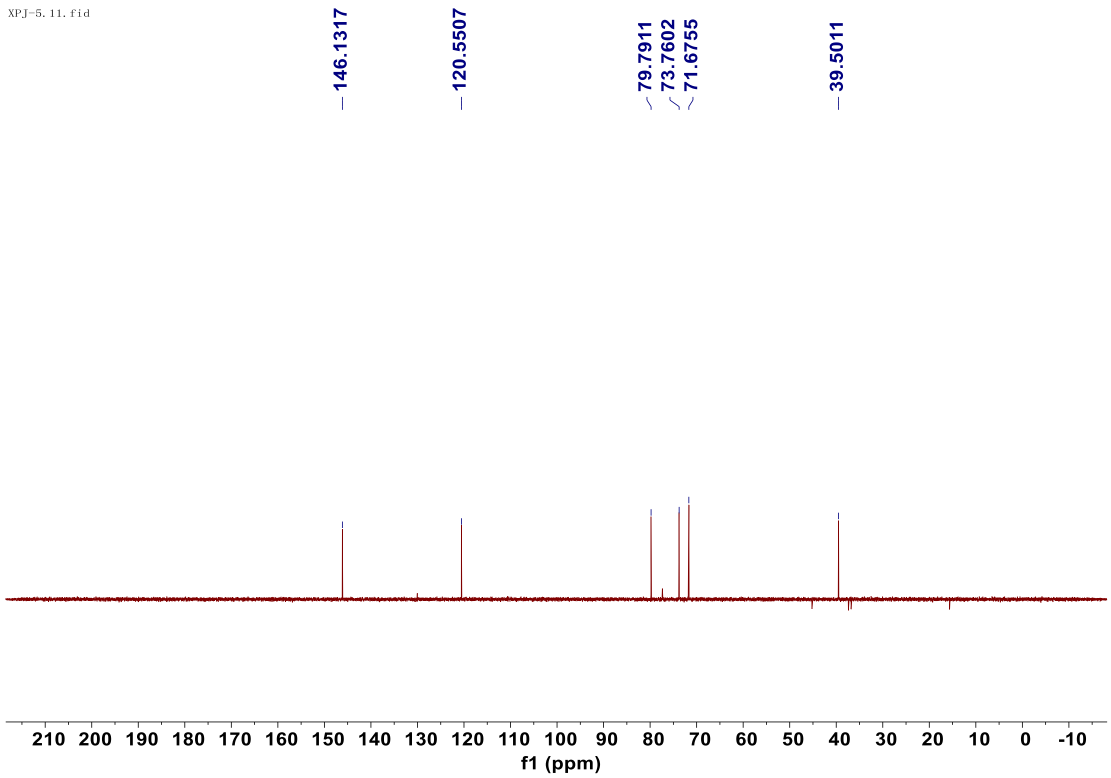

Figure S71. DEPT-90 spectrum of **7** (150 MHz, chloroform-*d*)

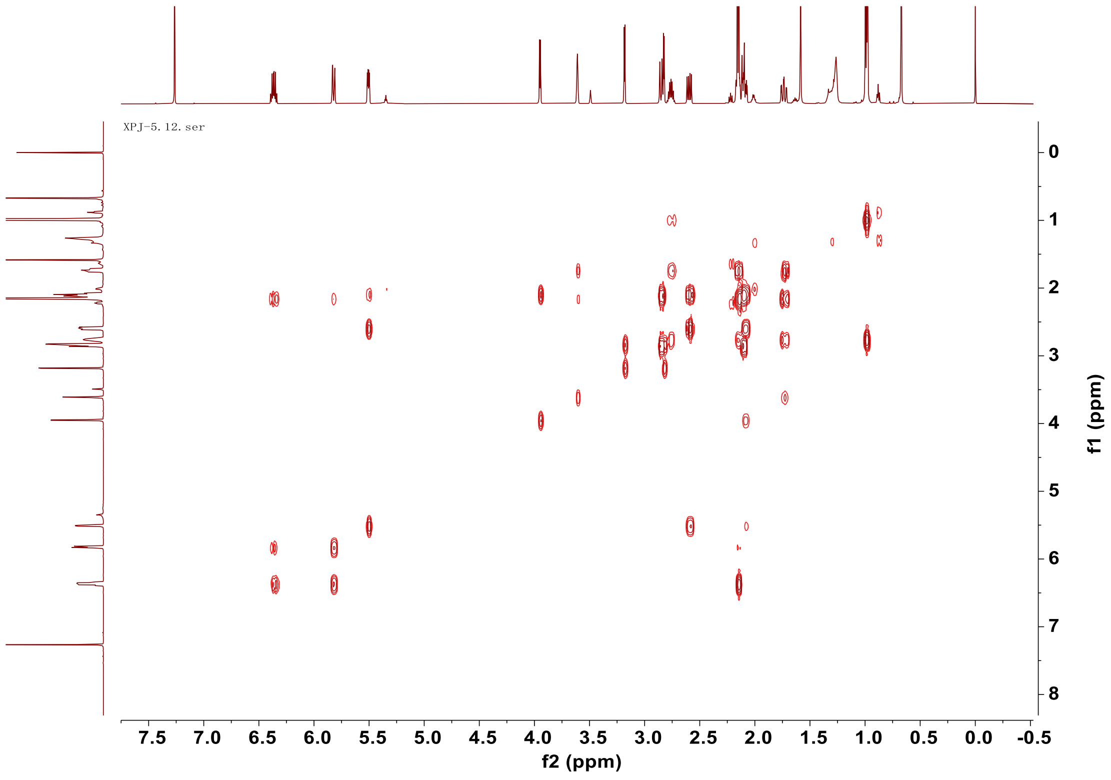

Figure S72. ^{1}H–^{1}H COSY spectrum of **7**

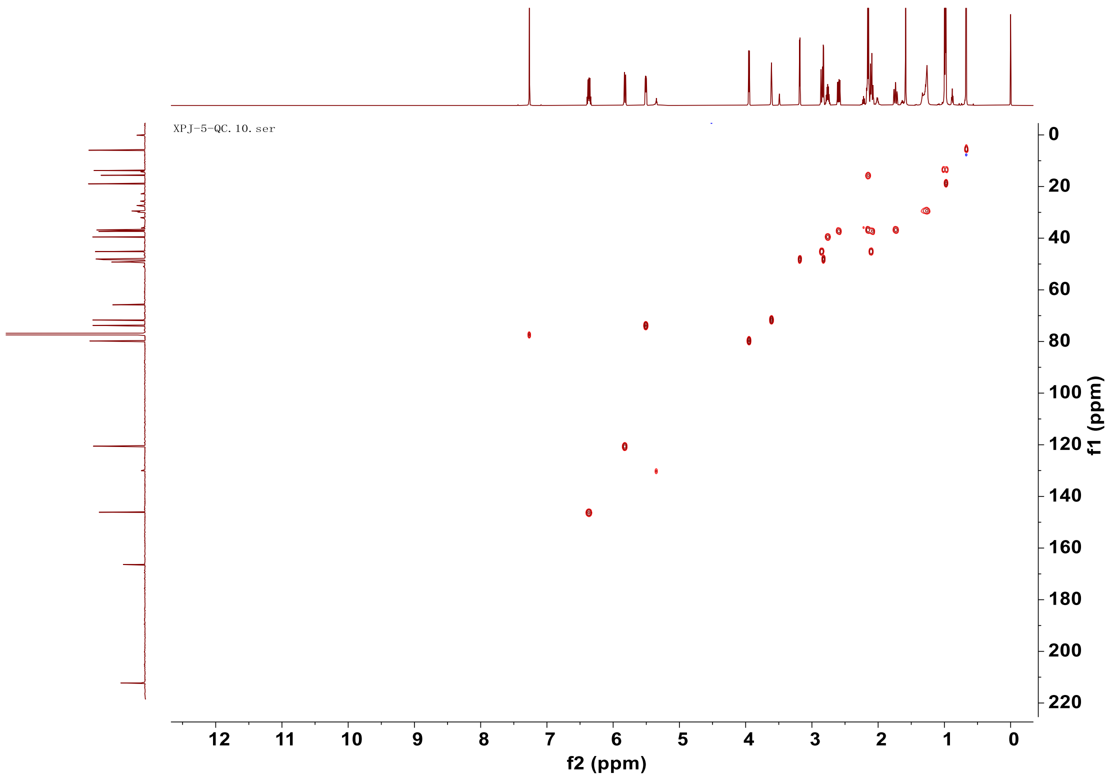

Figure S73. HSQC spectrum of **7**

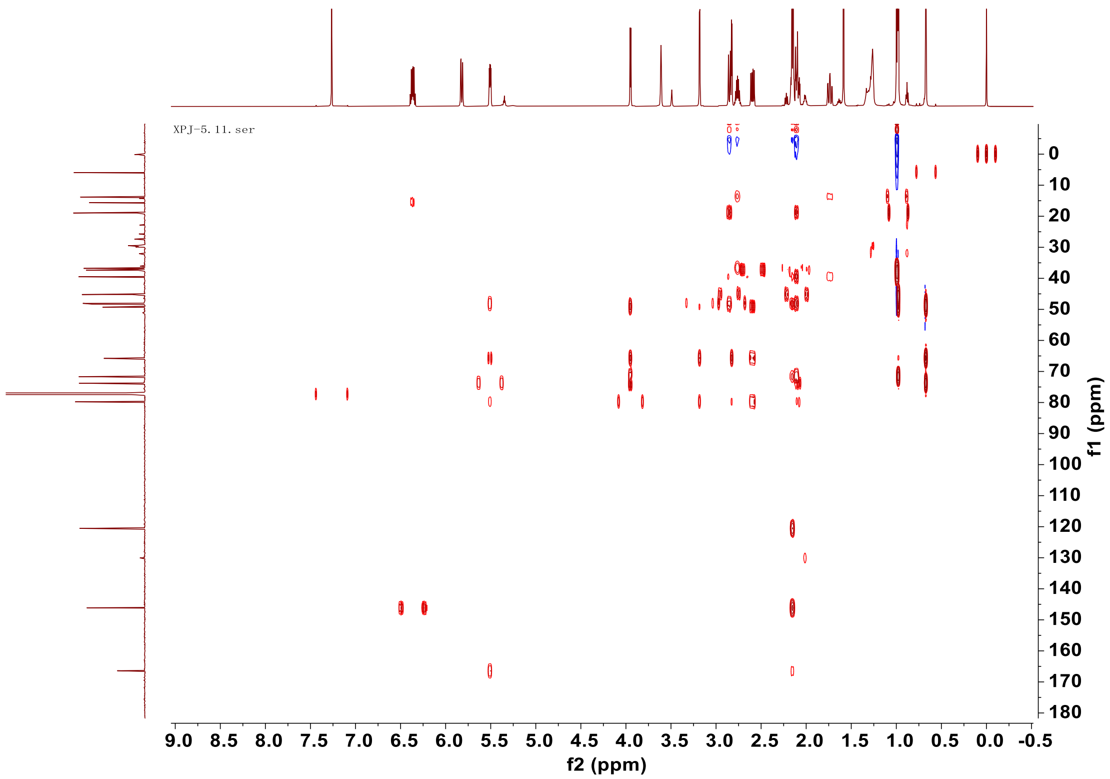

Figure S74. HMBC spectrum of **7**

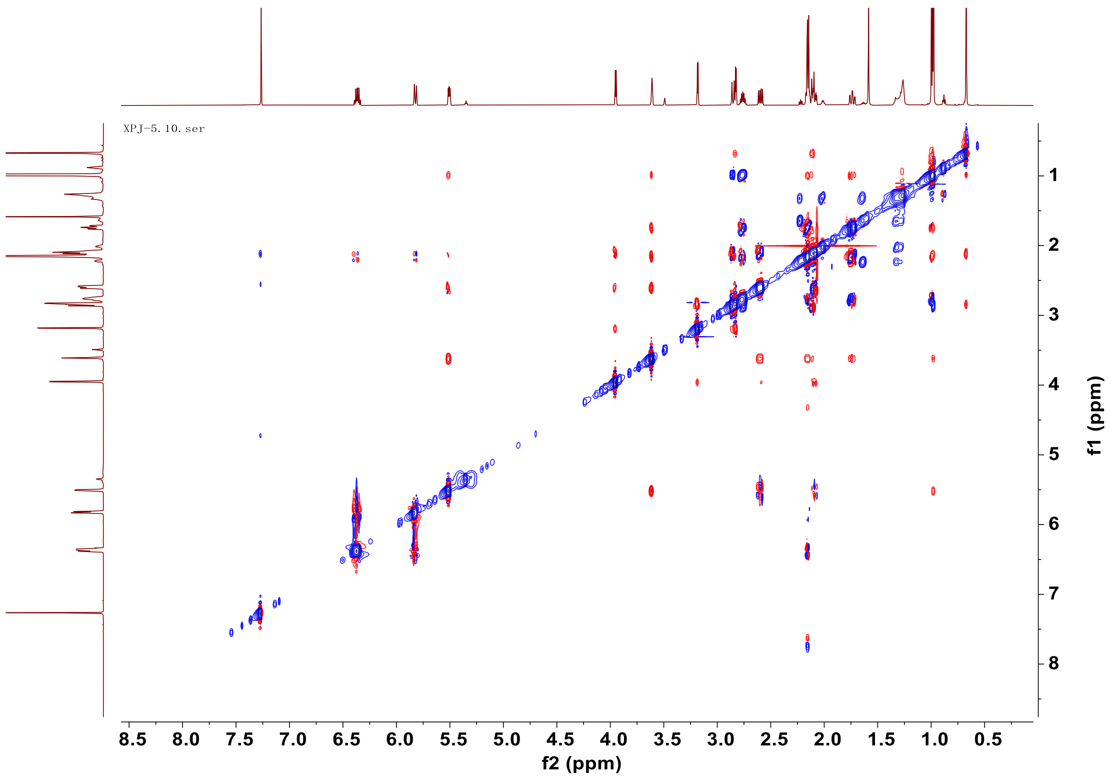

Figure S75. ROESY spectrum of **7**

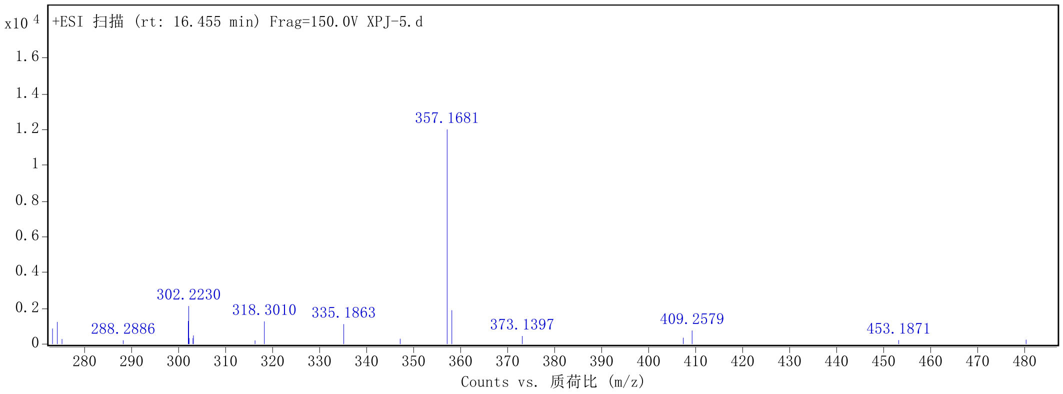

Figure S76. HRESIMS spectrum of **7**

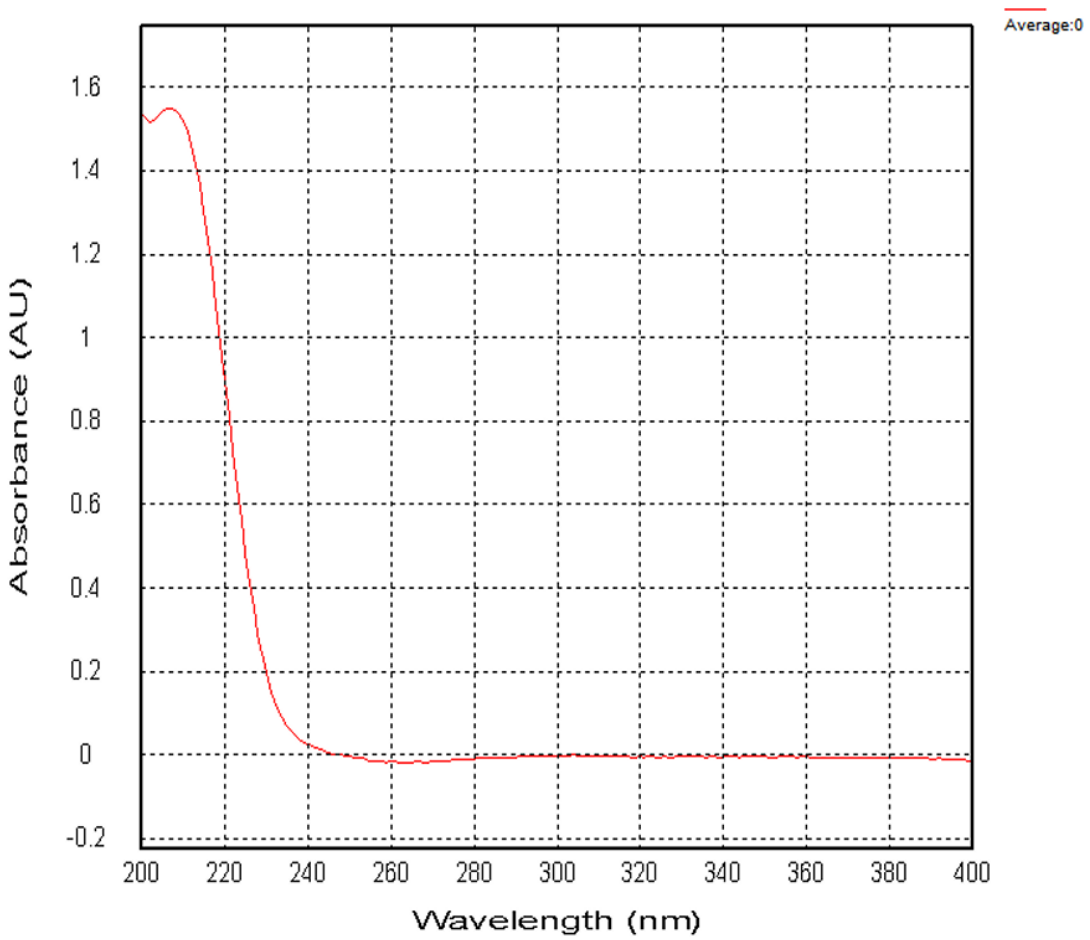

Figure S77. UV spectrum of **7**

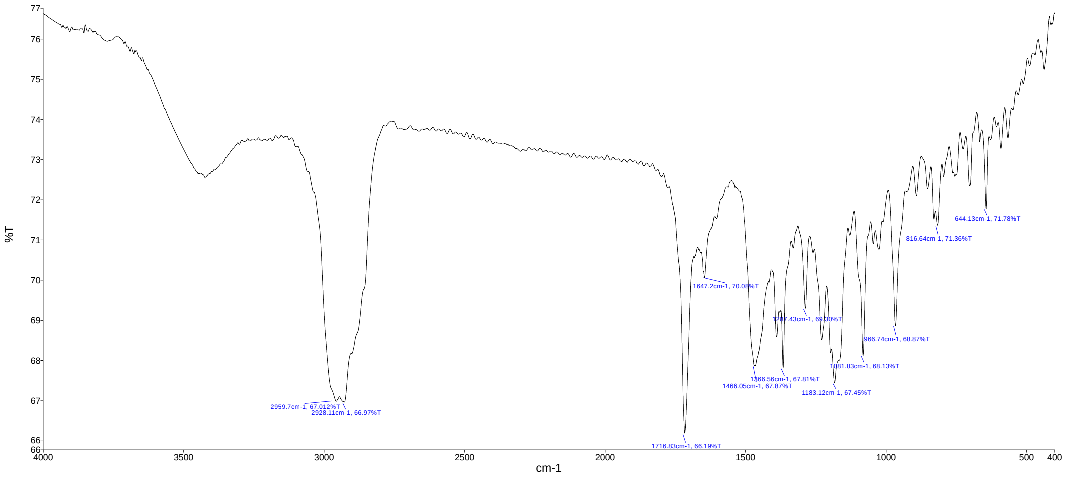

Figure S78. IR spectrum of **7**

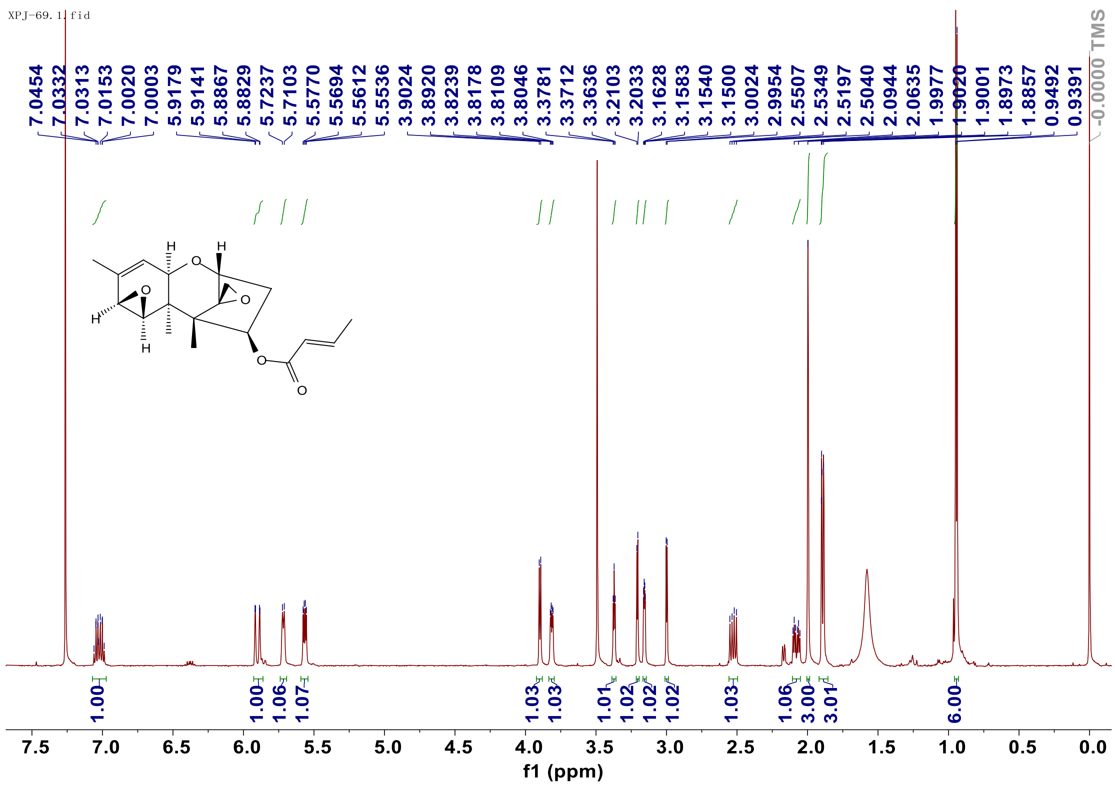

Figure S79. ^{1}H NMR spectrum of **8** (500 MHz, chloroform-*d*)

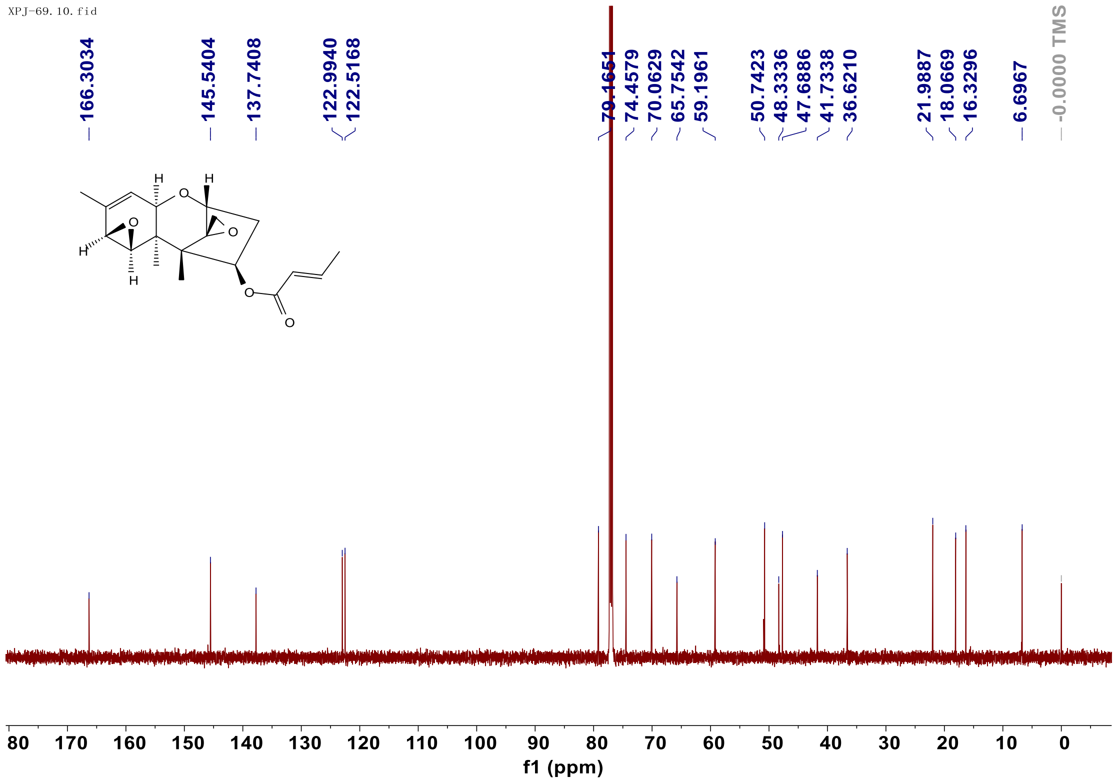

Figure S80. ^{13}C NMR spectrum of **8** (125 MHz, chloroform-*d*)

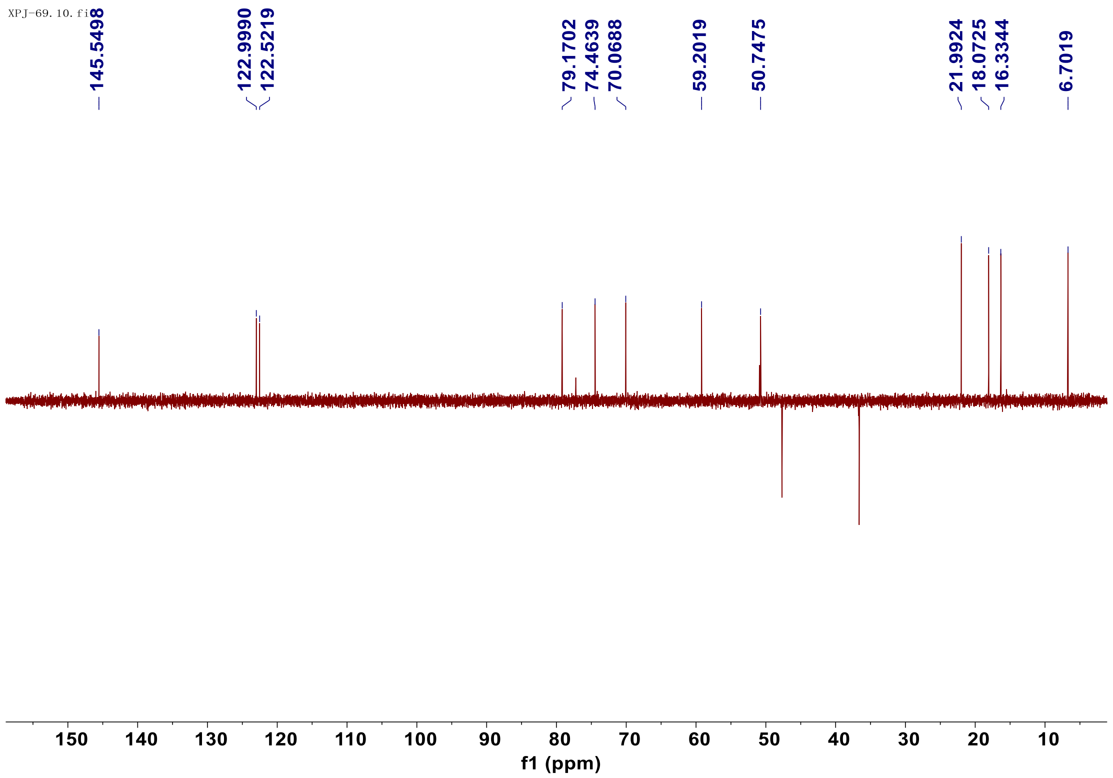

Figure S81. DEPT-135 spectrum of **8** (125 MHz, chloroform-*d*)

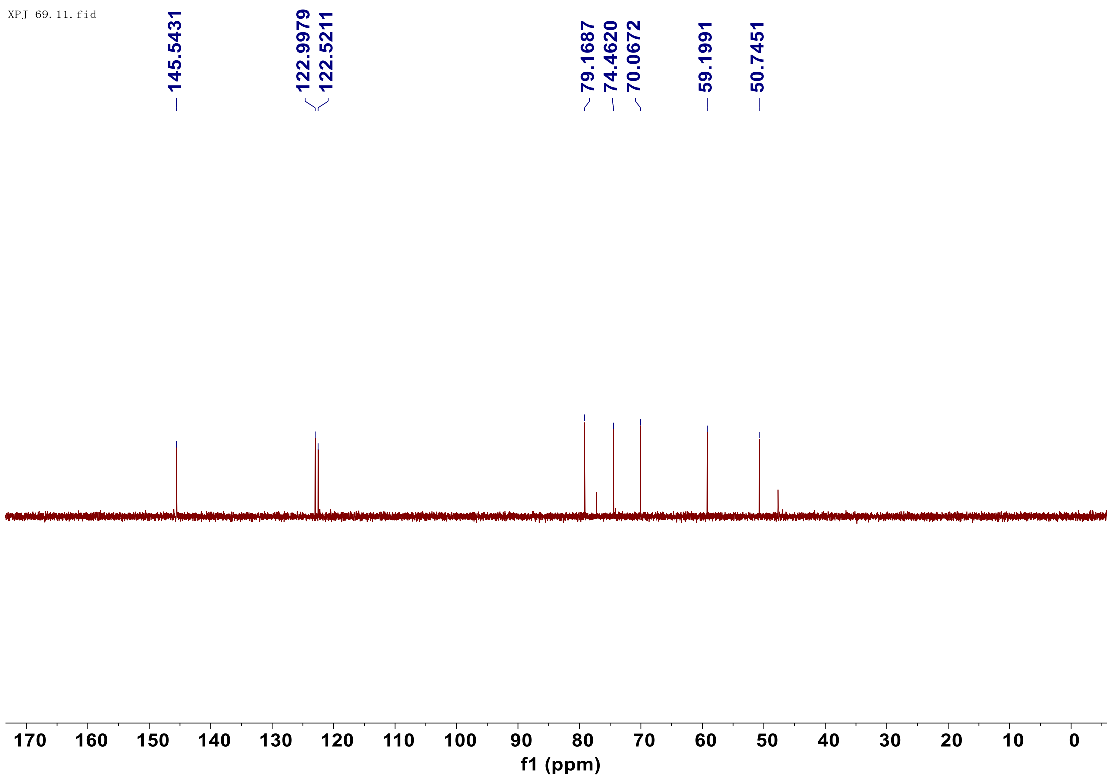

Figure S82. DEPT-90 spectrum of **8** (125 MHz, chloroform-*d*)

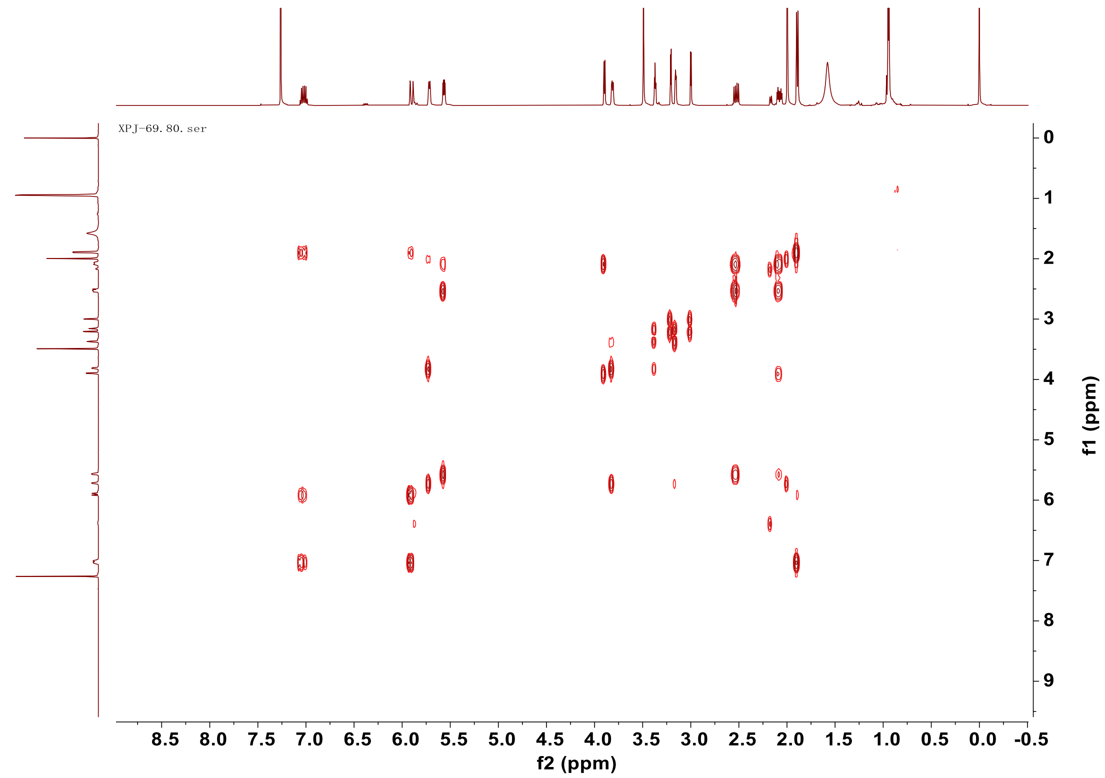

Figure S83. ^{1}H−^{1}H COSY spectrum of **8**

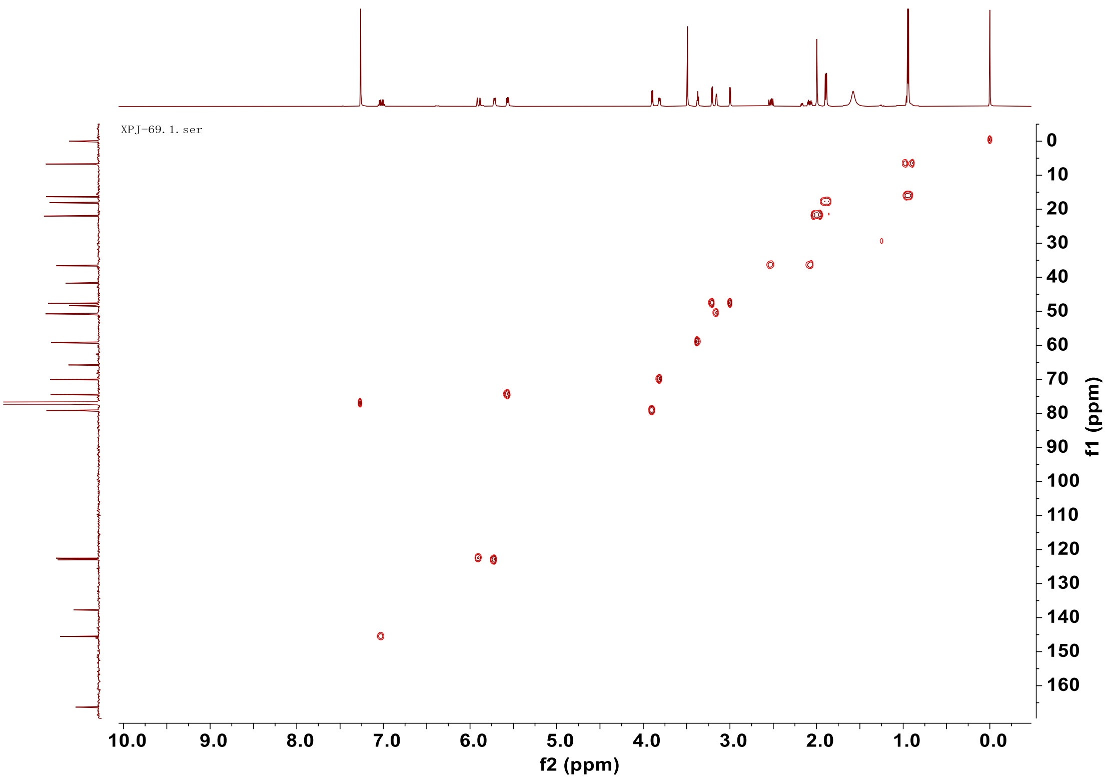

Figure S84. HSQC spectrum of **8**

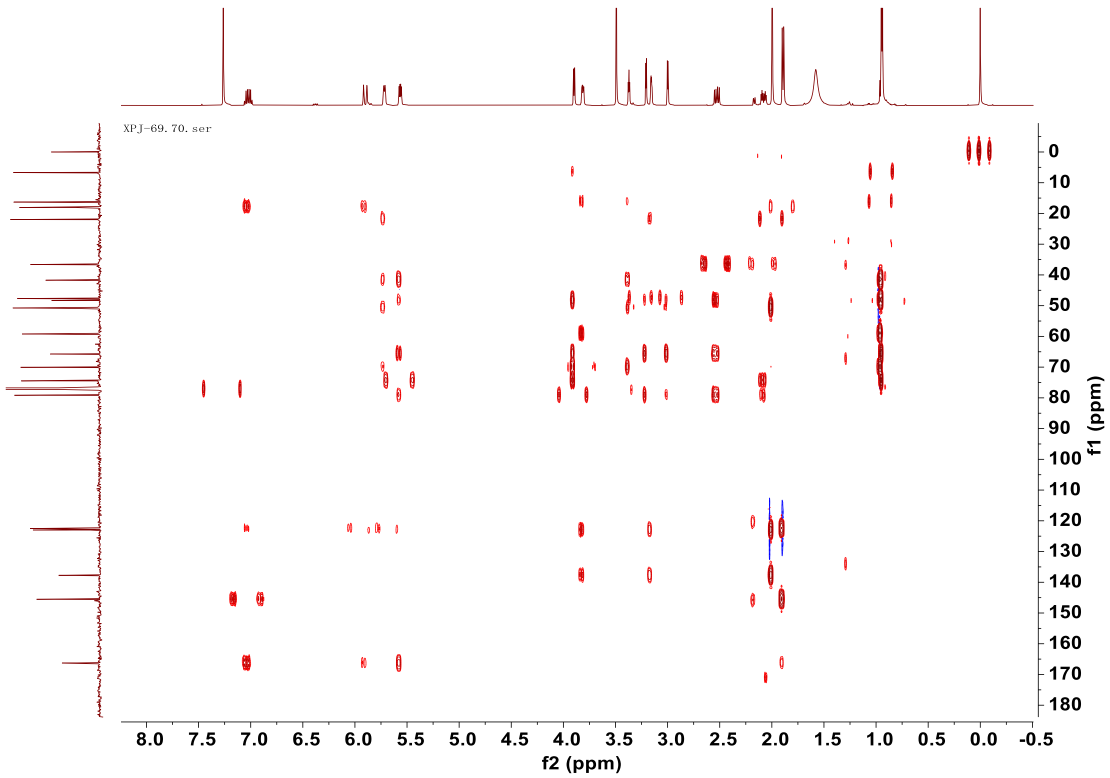

Figure S85. HMBC spectrum of **8**

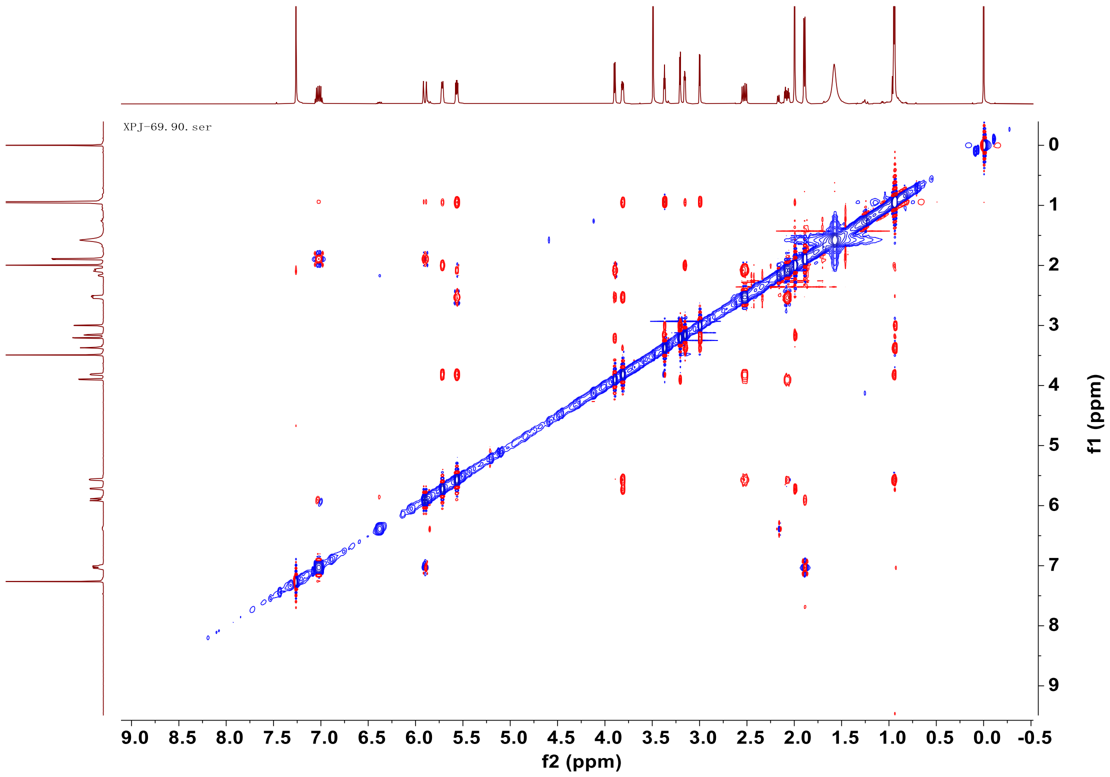

Figure S86. NOESY spectrum of **8**

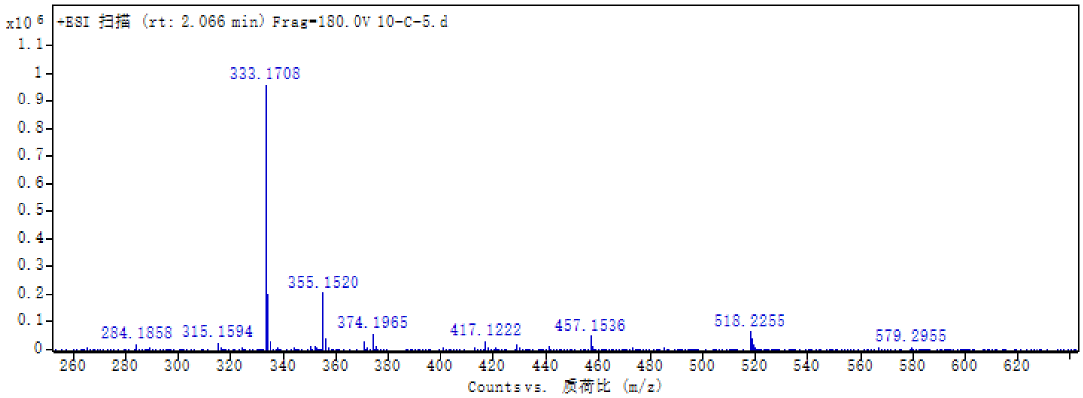

Figure S87. HRESIMS spectrum of **8**

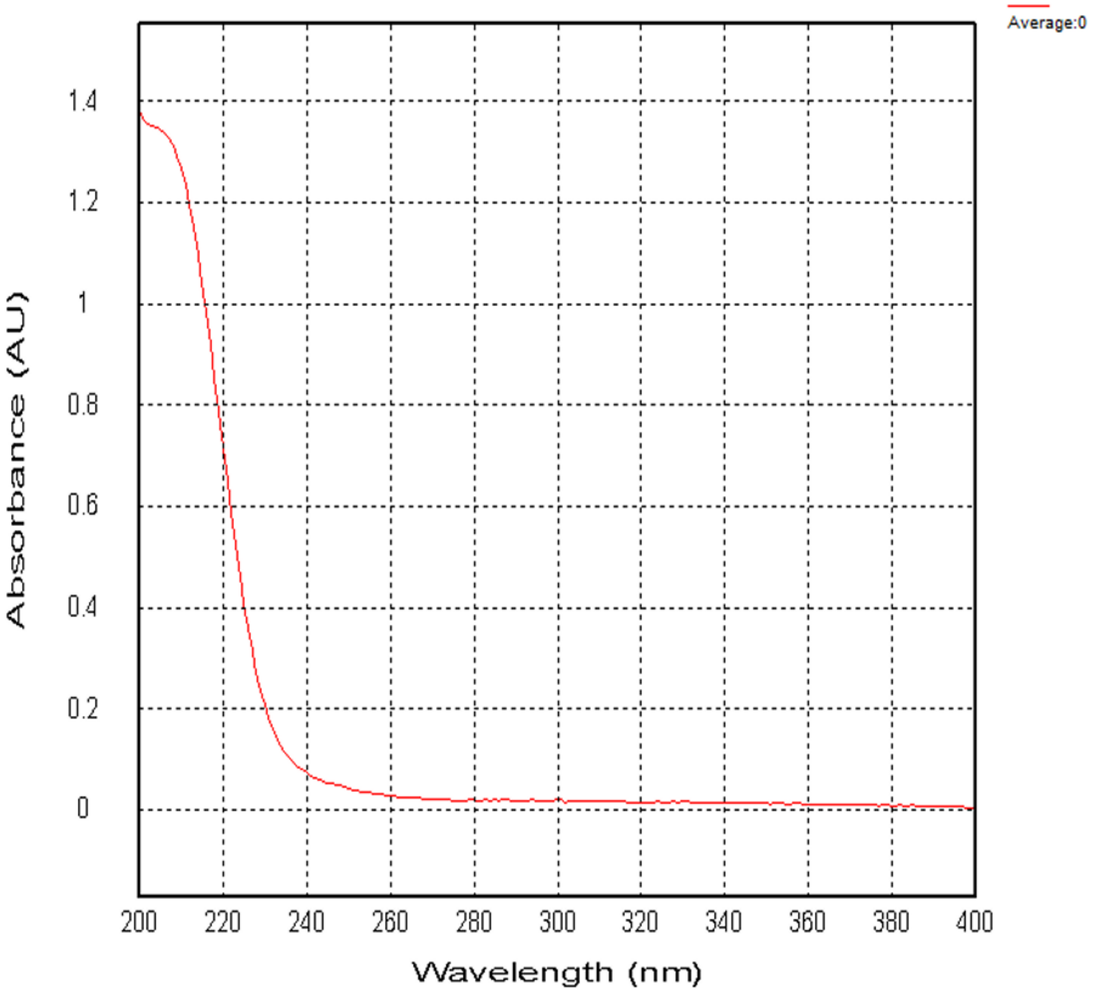

Figure S88. UV spectrum of **8**

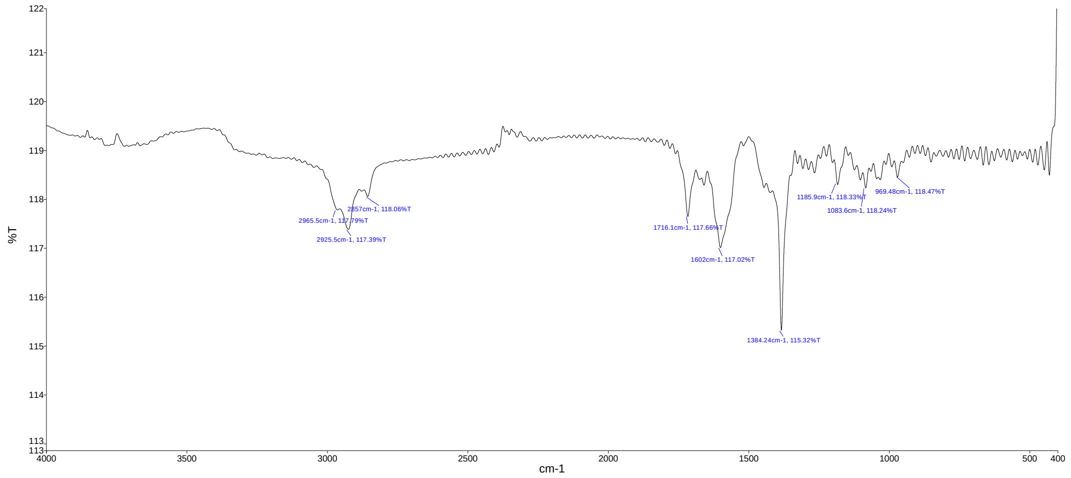

Figure S89. IR spectrum of **8**

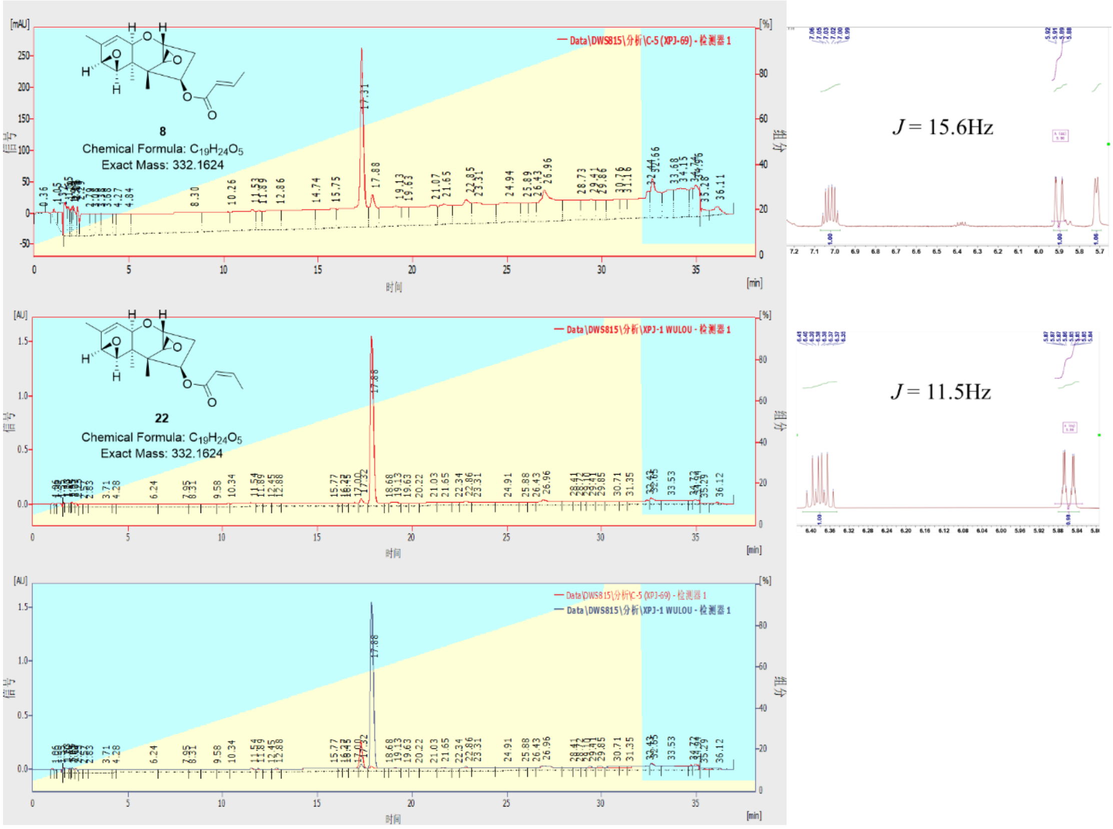

Figure S90. Elite HPLC comparative analysis of **8** and **22**

Analysis method: ZORBAX SB-C18 5μm 4.6 × 150mm, 5−95% CH_3CN−H_2O, 30 min, 210 nm

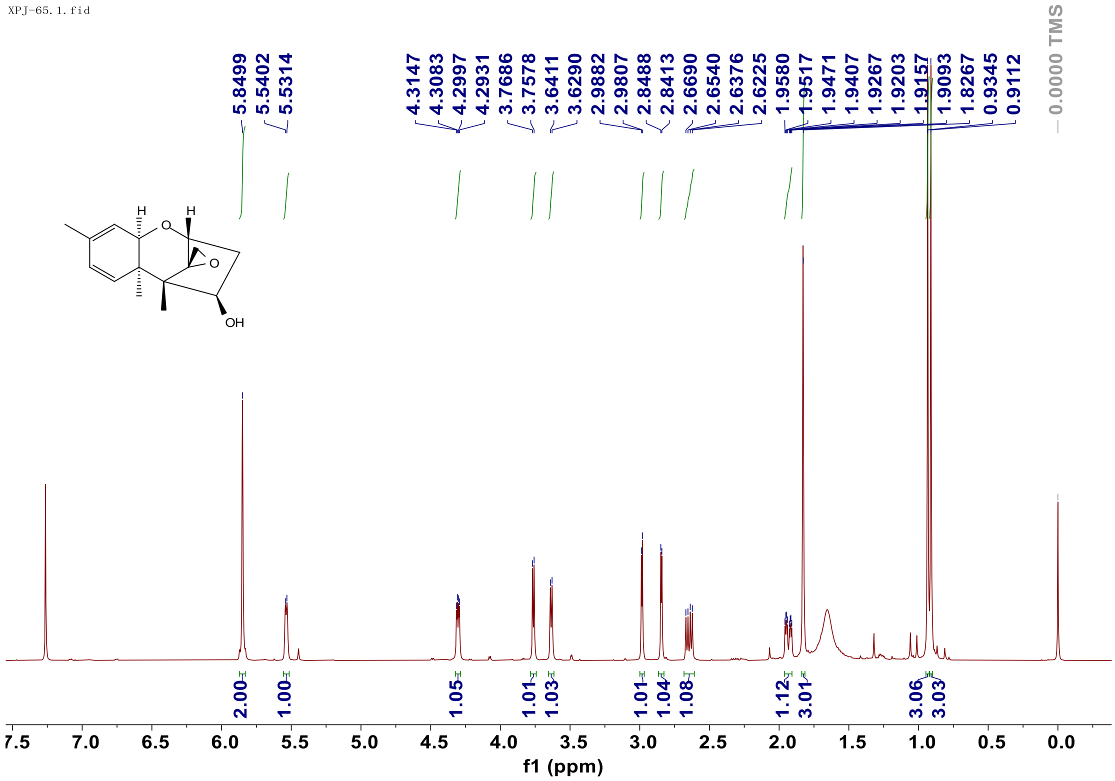

Figure S91. ^{1}H NMR spectrum of **9** (500 MHz, chloroform-*d*)

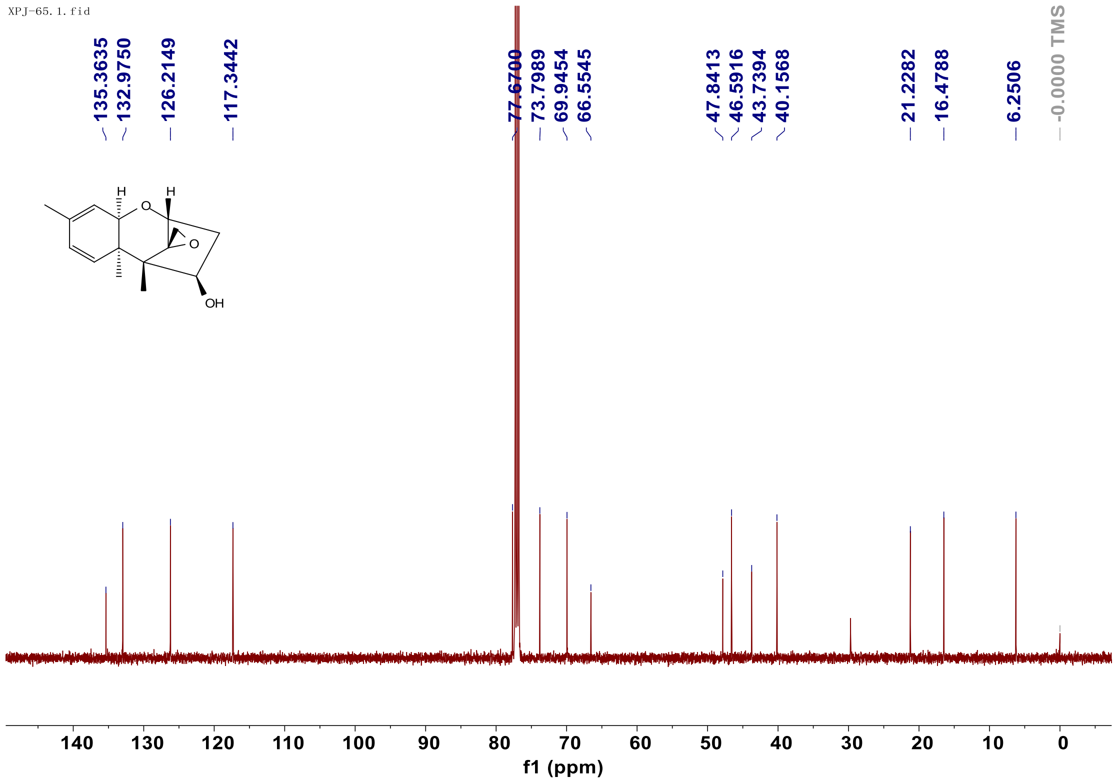

Figure S92. ^{13}C NMR spectrum of **9** (125 MHz, chloroform-*d*)

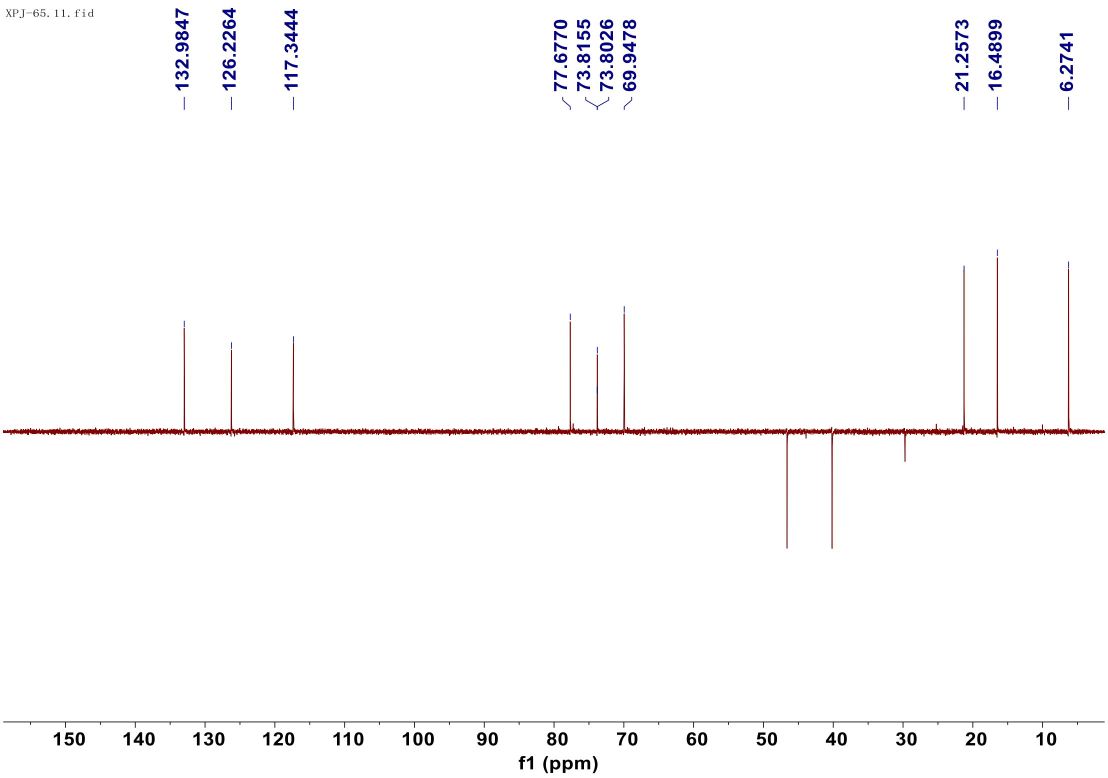

Figure S93. DEPT-135 spectrum of **9** (125 MHz, chloroform-*d*)

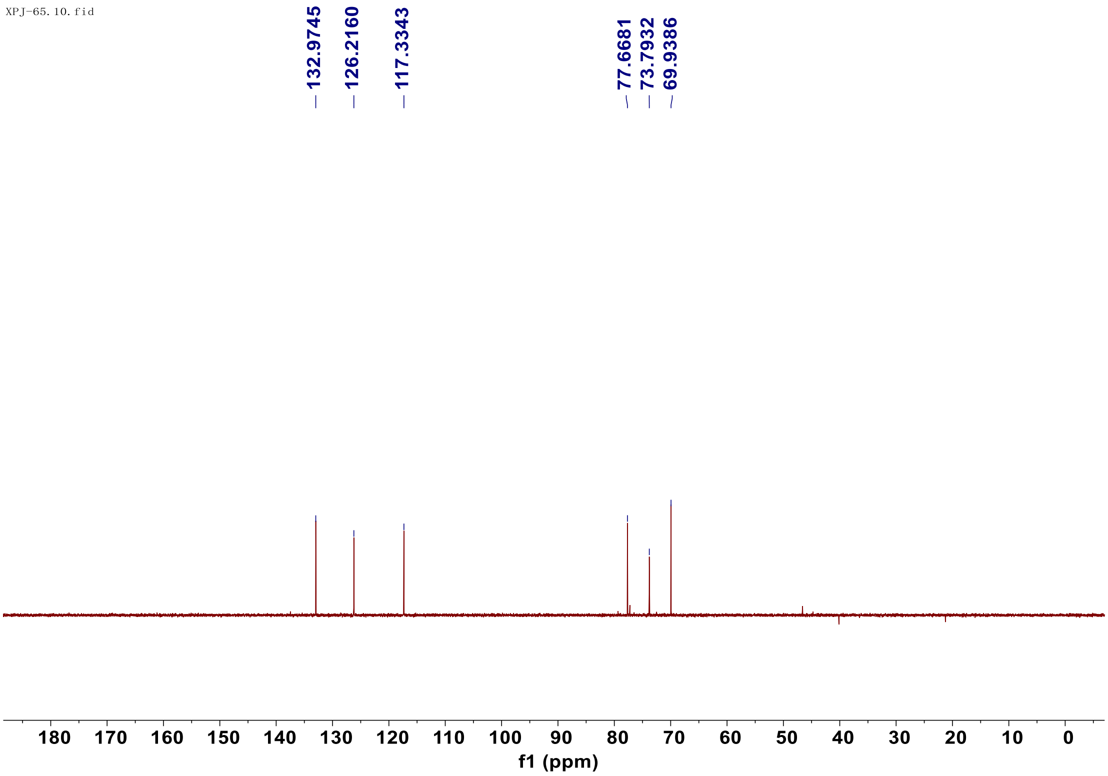

Figure S94. DEPT-90 spectrum of **9** (125 MHz, chloroform-*d*)

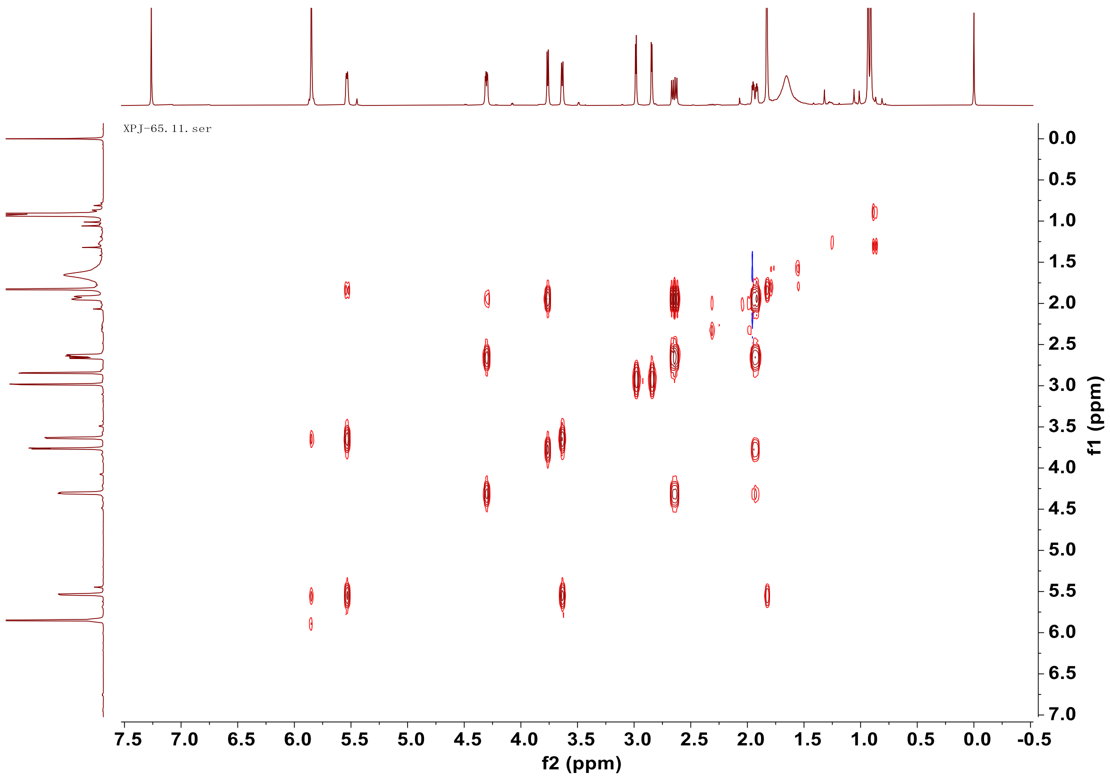

Figure S95. $^{1}H-^{1}H$ COSY spectrum of **9** (125 MHz, chloroform-*d*)

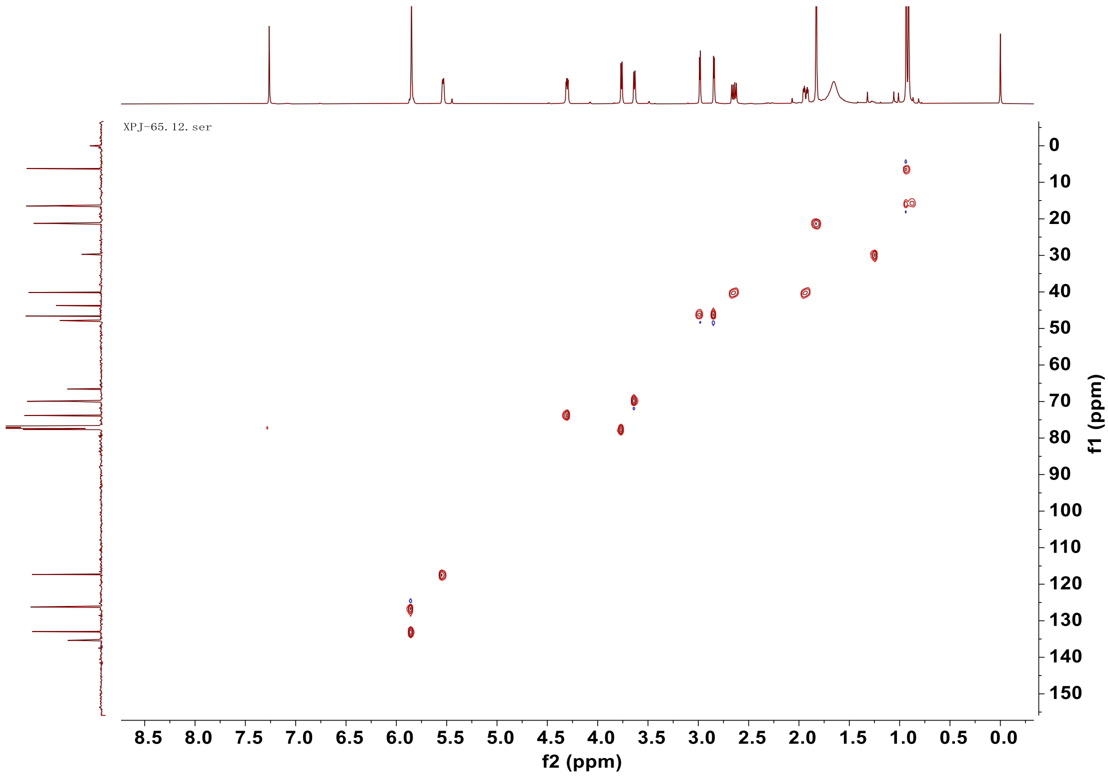

Figure S96. HSQC spectrum of **9**

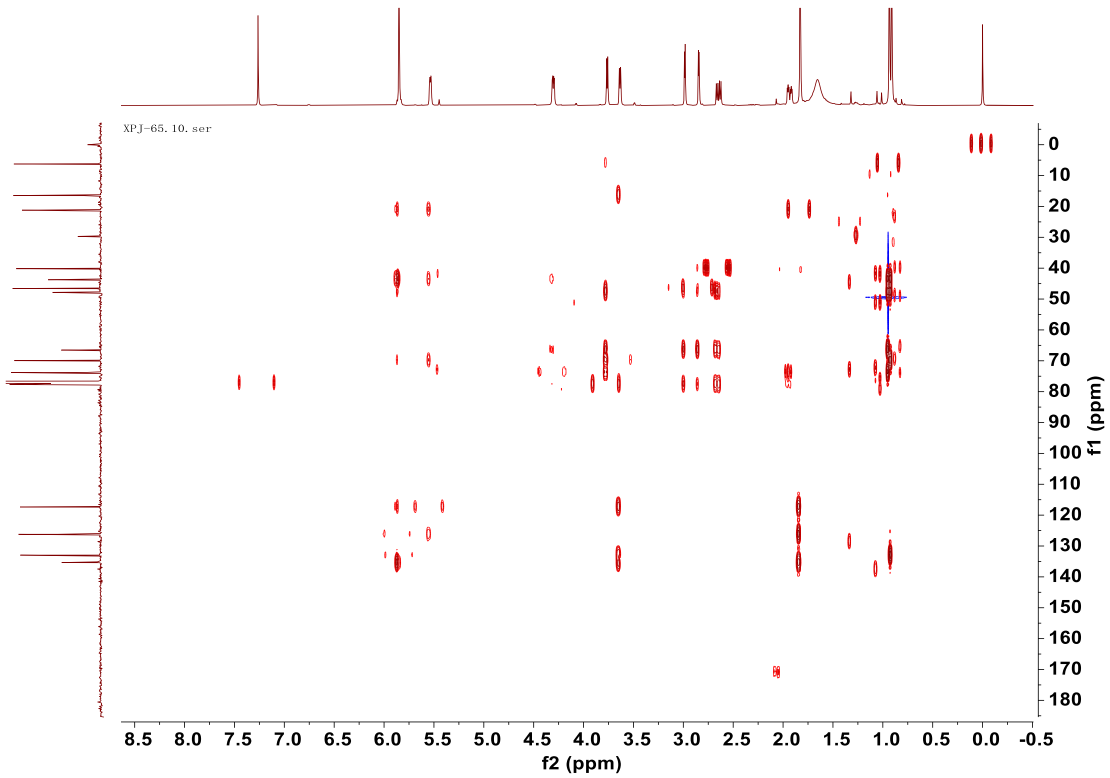

Figure S97. HMBC spectrum of **9**

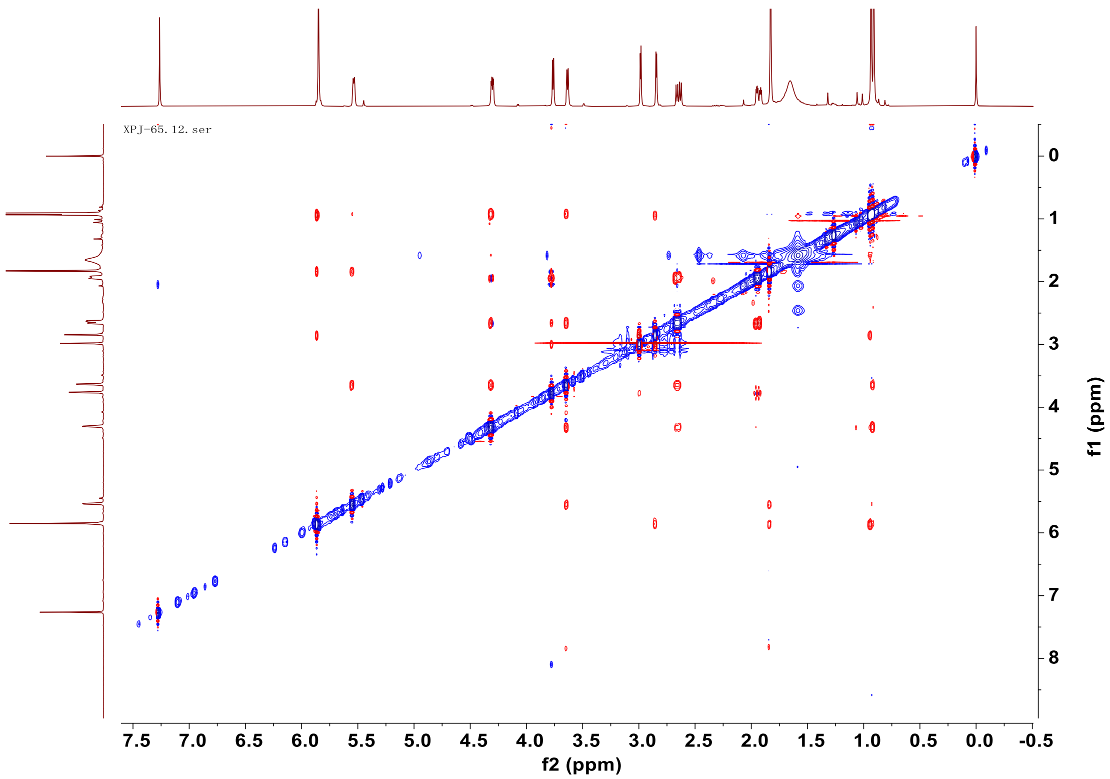

Figure S98. NOESY spectrum of **9**

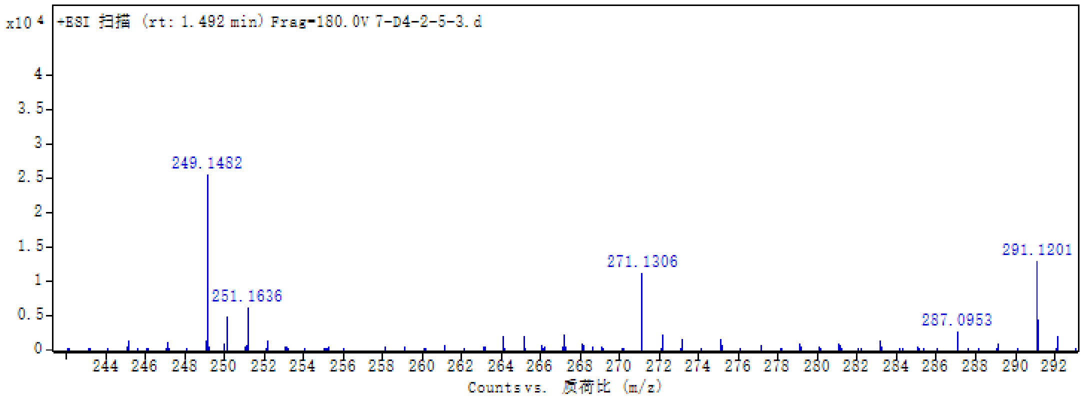

Figure S99. HRESIMS spectrum of **9**

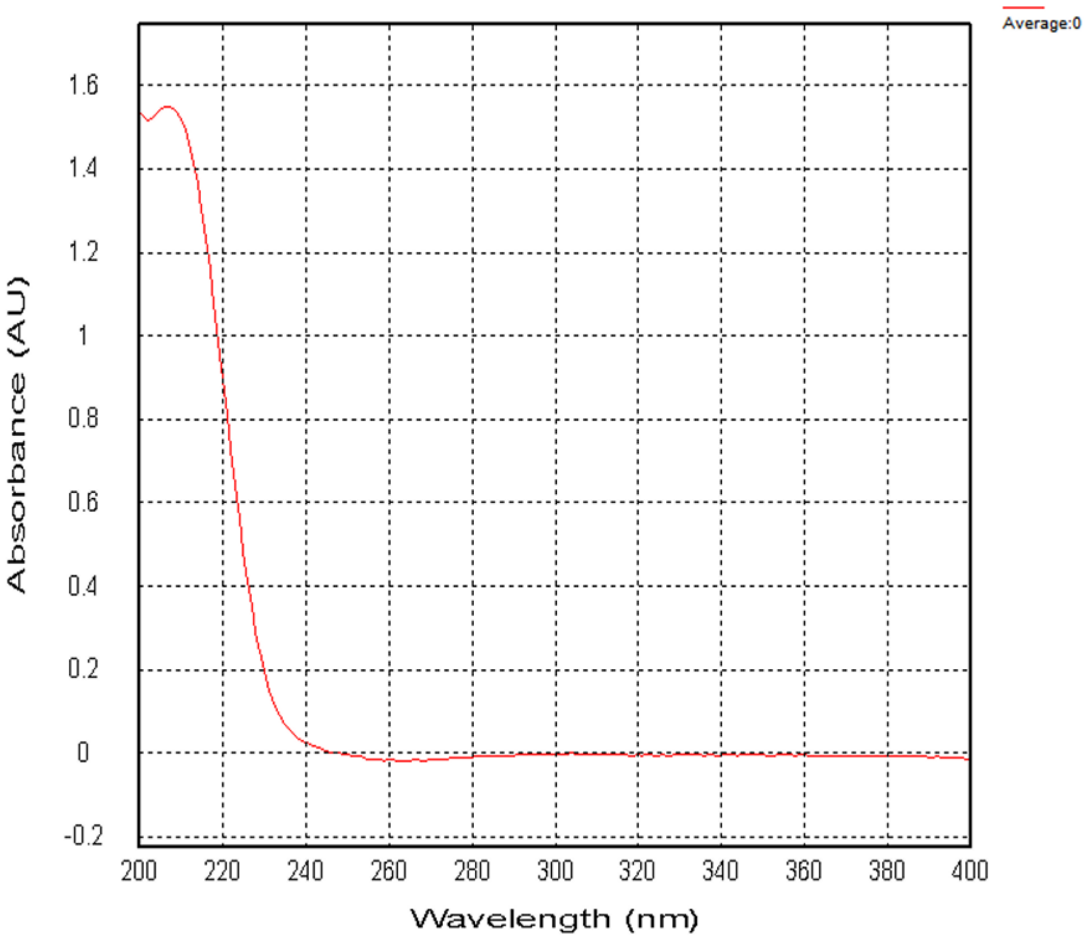

Figure S100. UV spectrum of **9**

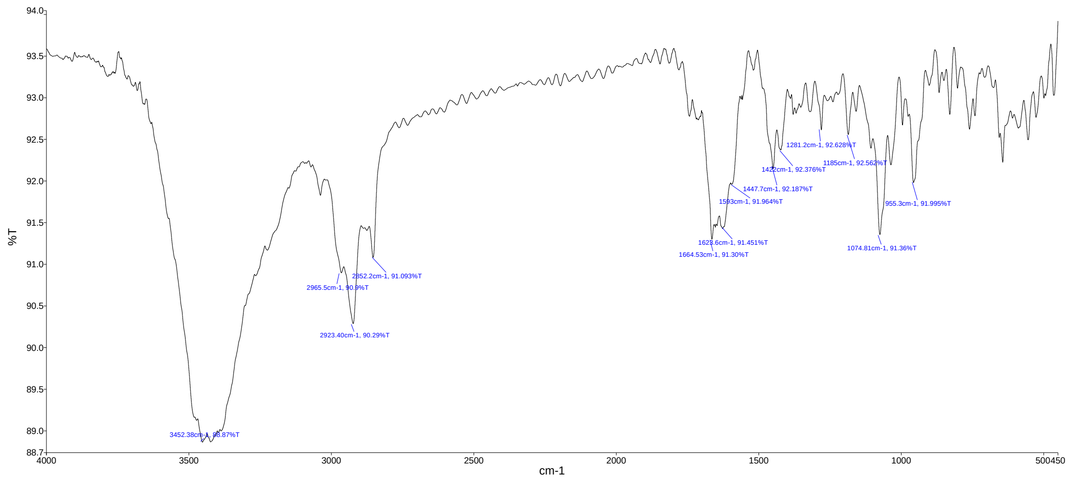

Figure S101. IR spectrum of **9**

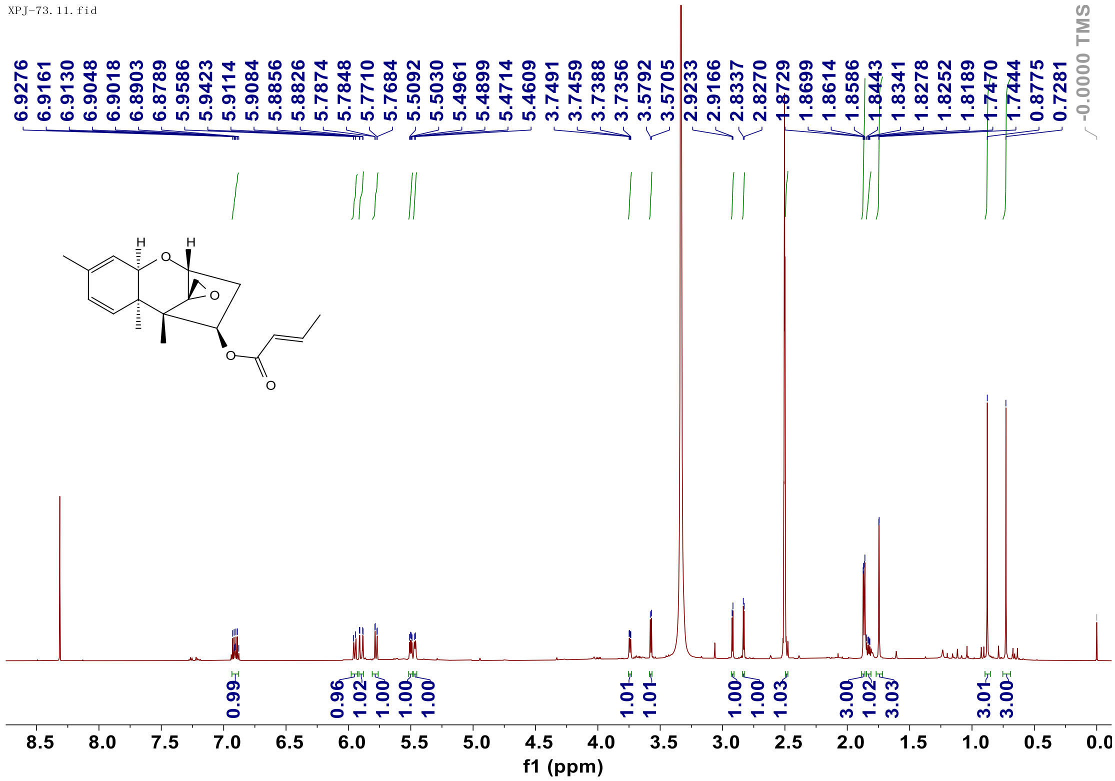

Figure S102. ^{1}H NMR spectrum of **10** (600 MHz, dimethyl sulfoxide-d_6)

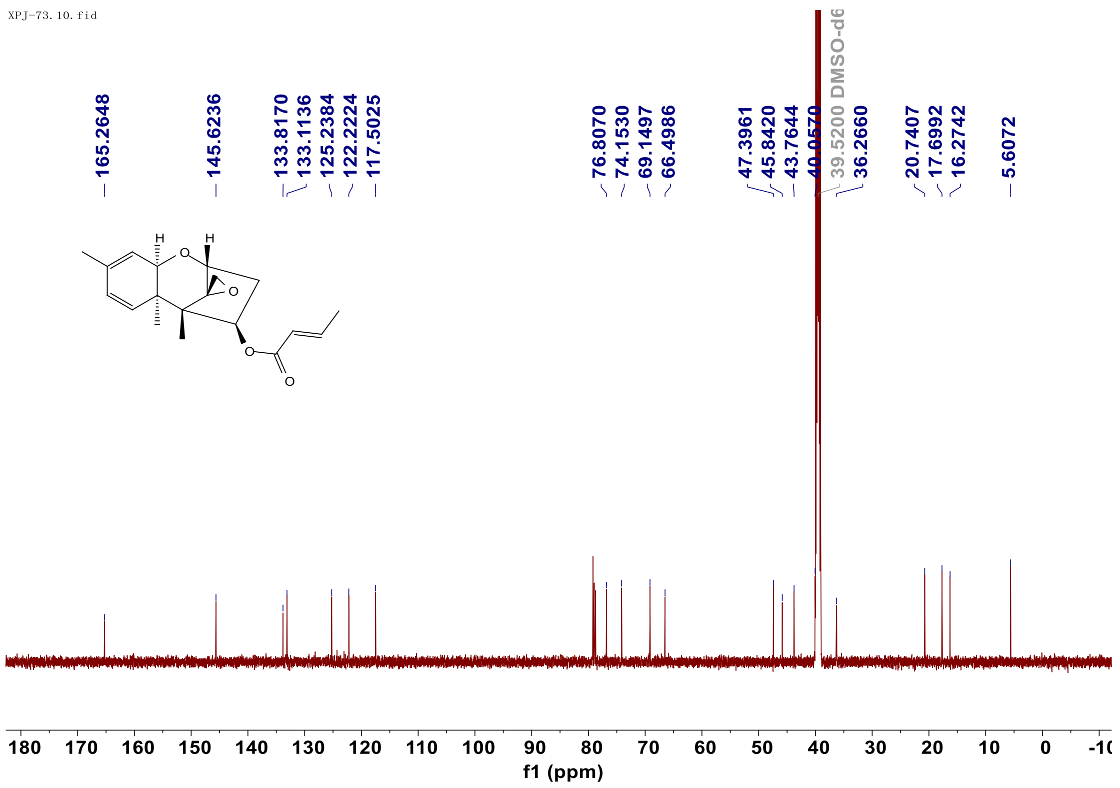

Figure S103. ^{13}C NMR spectrum of **10** (150 MHz, dimethyl sulfoxide-d_6)

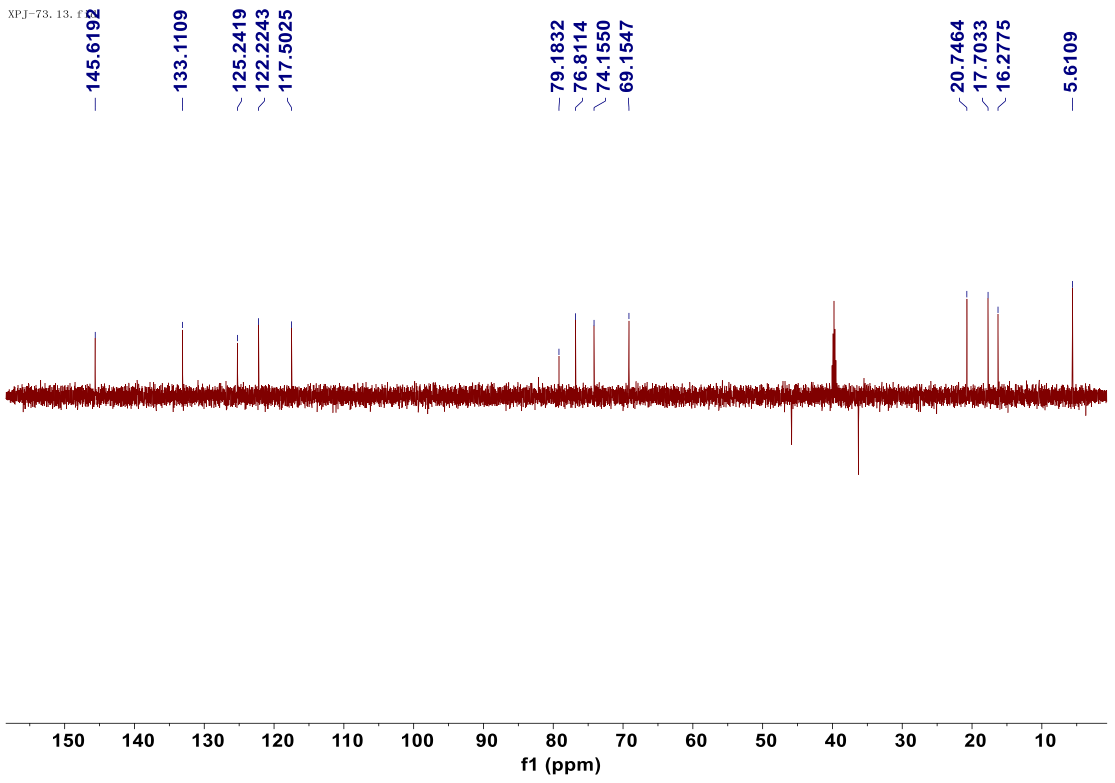

Figure S104. DEPT-135 spectrum of **10** (150 MHz, dimethyl sulfoxide-d_6)

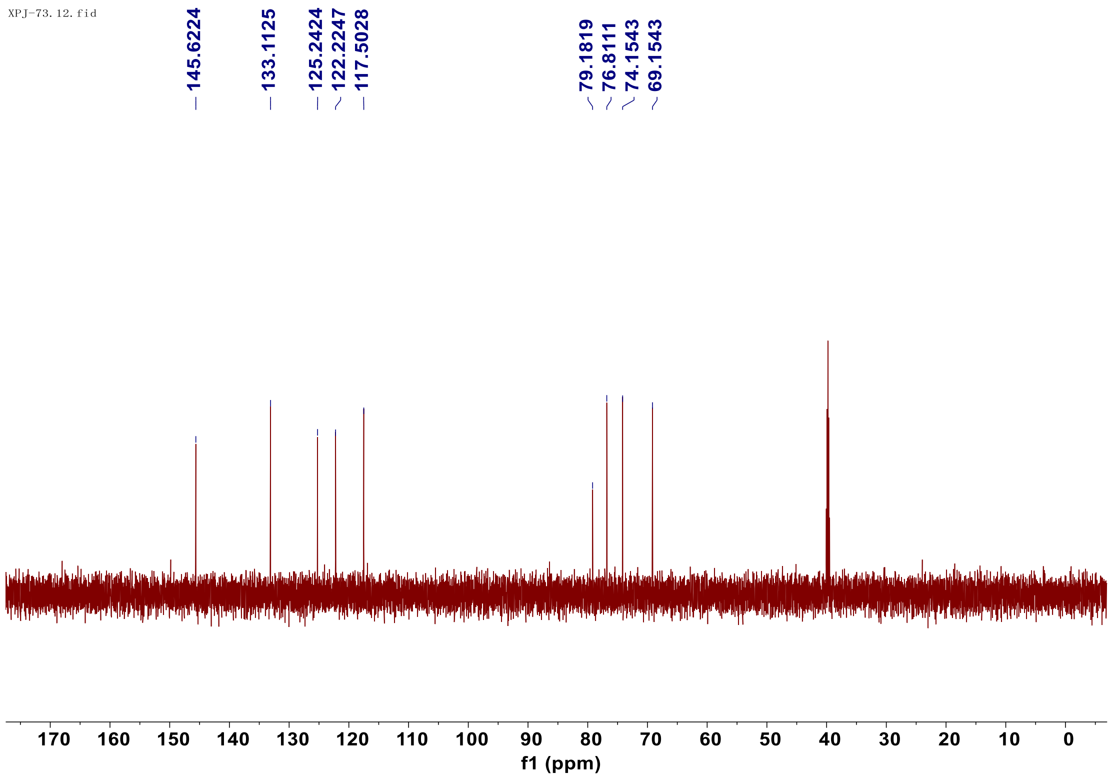

Figure S105. DEPT-90 spectrum of **10** (150 MHz, dimethyl sulfoxide-d_6)

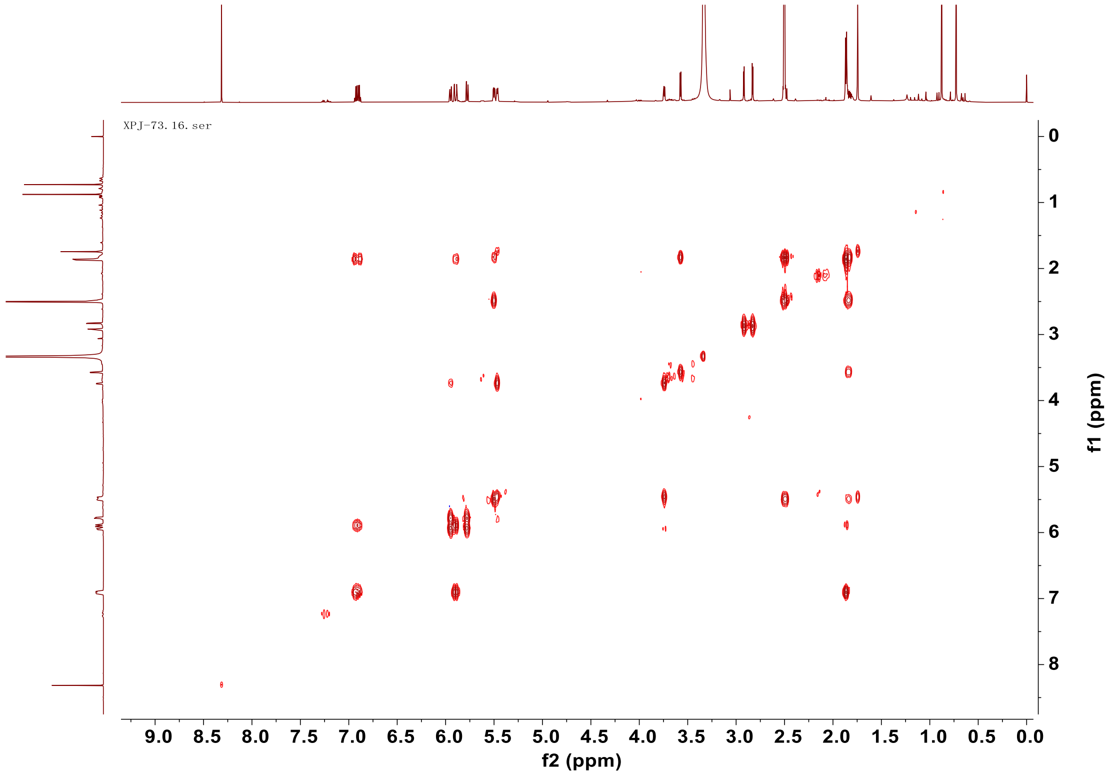

Figure S106. ^{1}H–^{1}H COSY spectrum of **10**

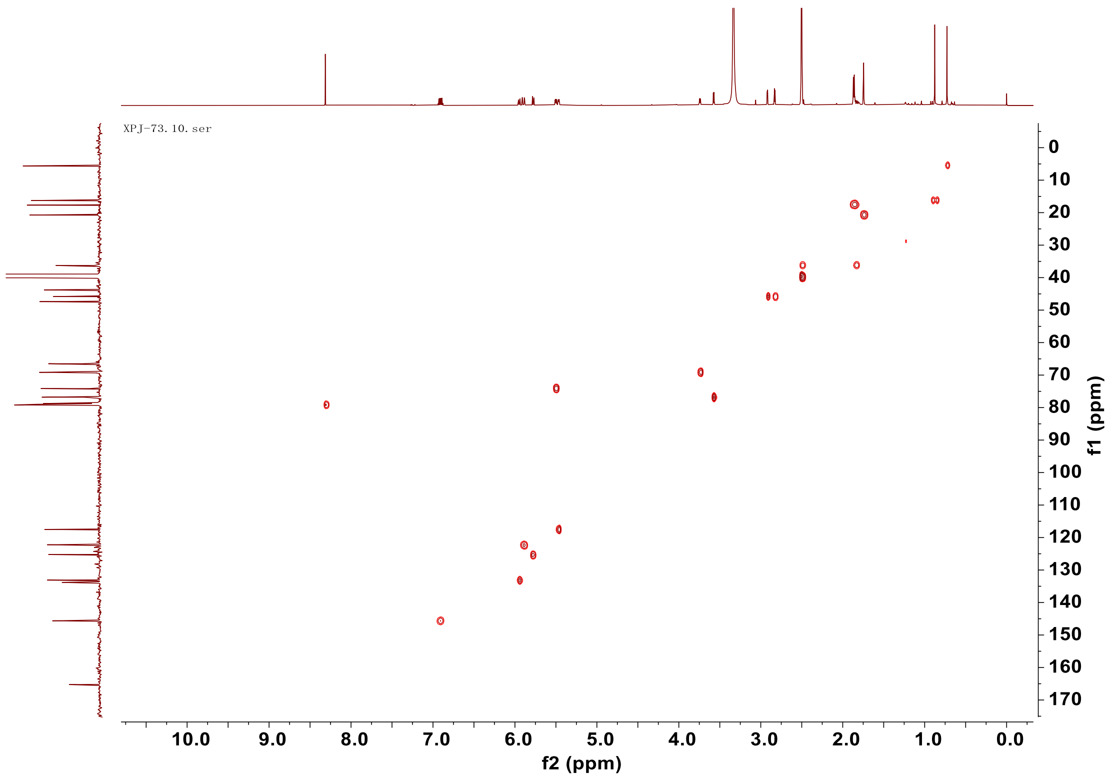

Figure S107. HSQC spectrum of **10**

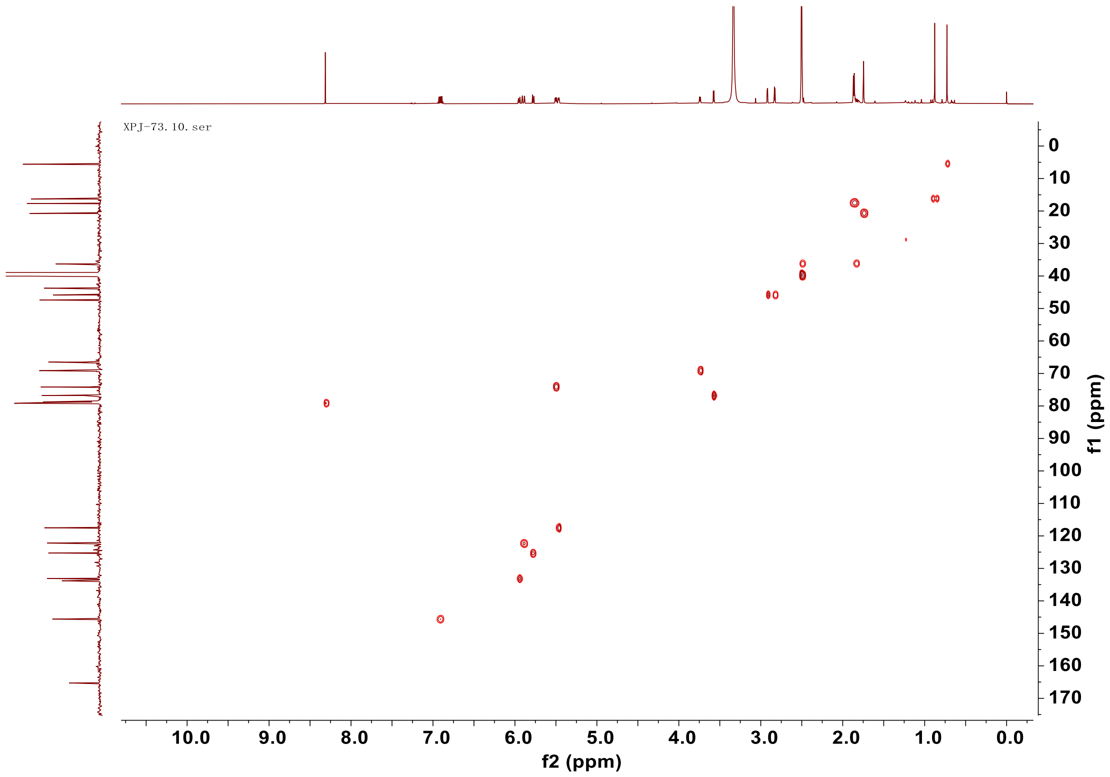

Figure S108. HMBC spectrum of **10**

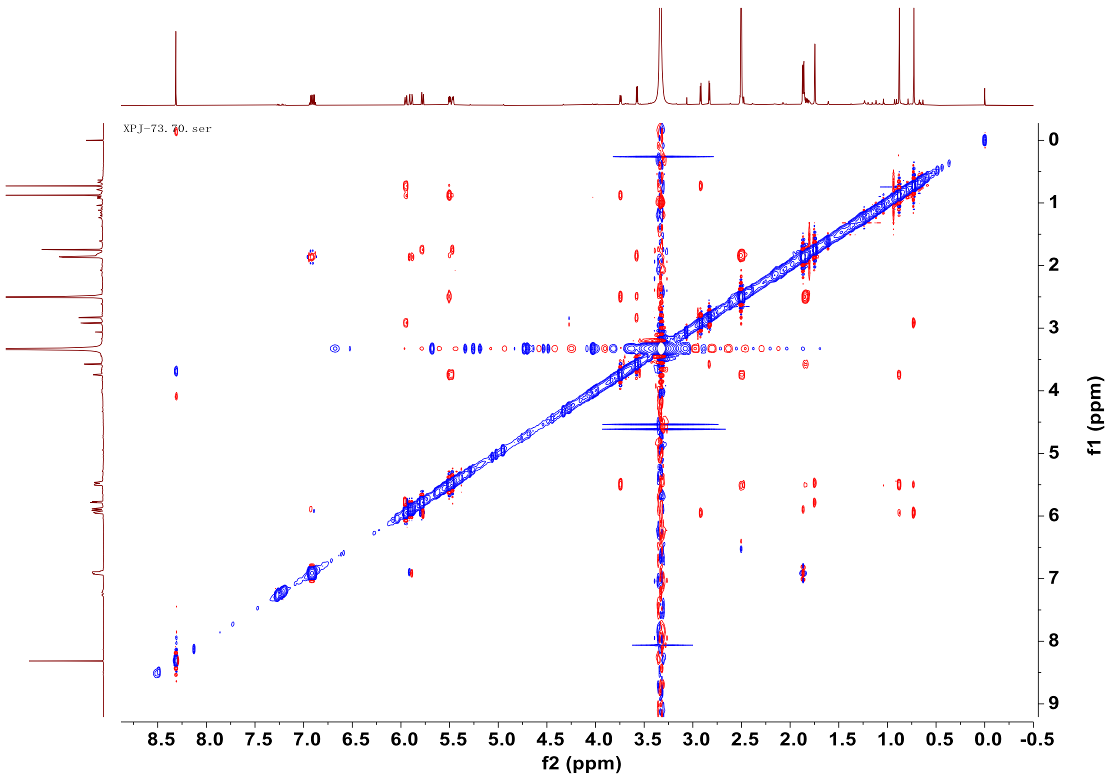

Figure S109. NOESY spectrum of **10**

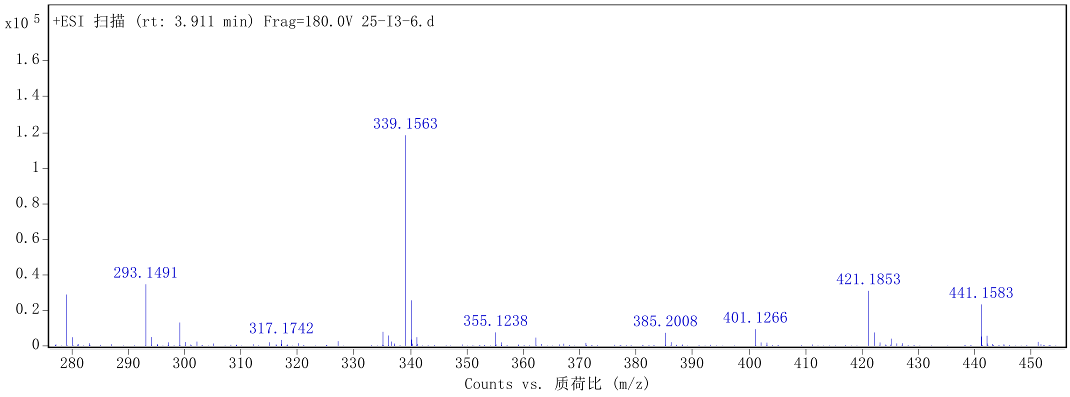

Figure S110. HRESIMS spectrum of **10**

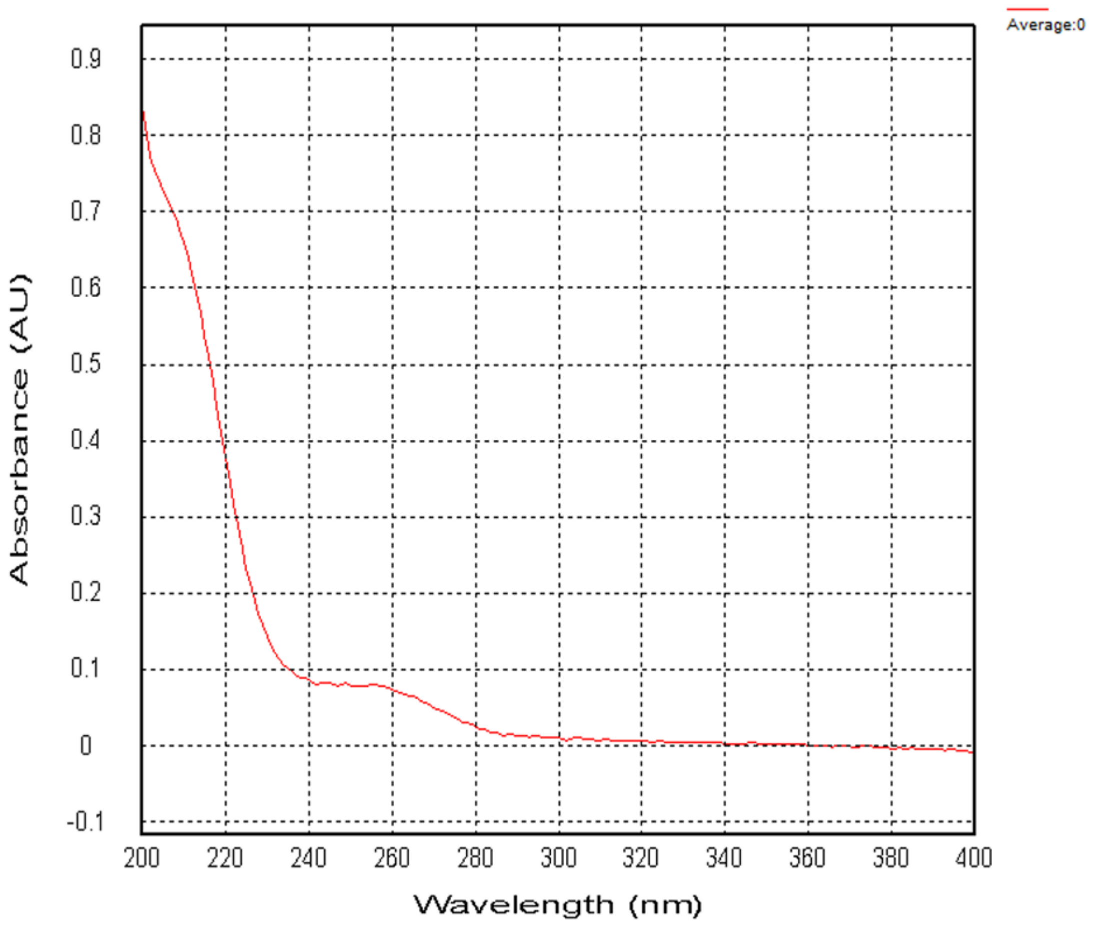

Figure S111. UV spectrum of **10**

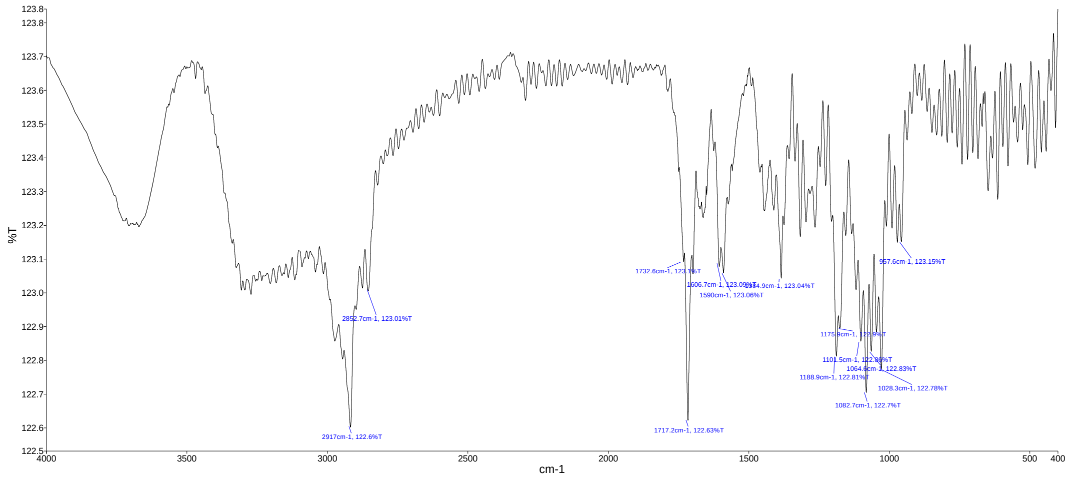

Figure S112. IR spectrum of **10**

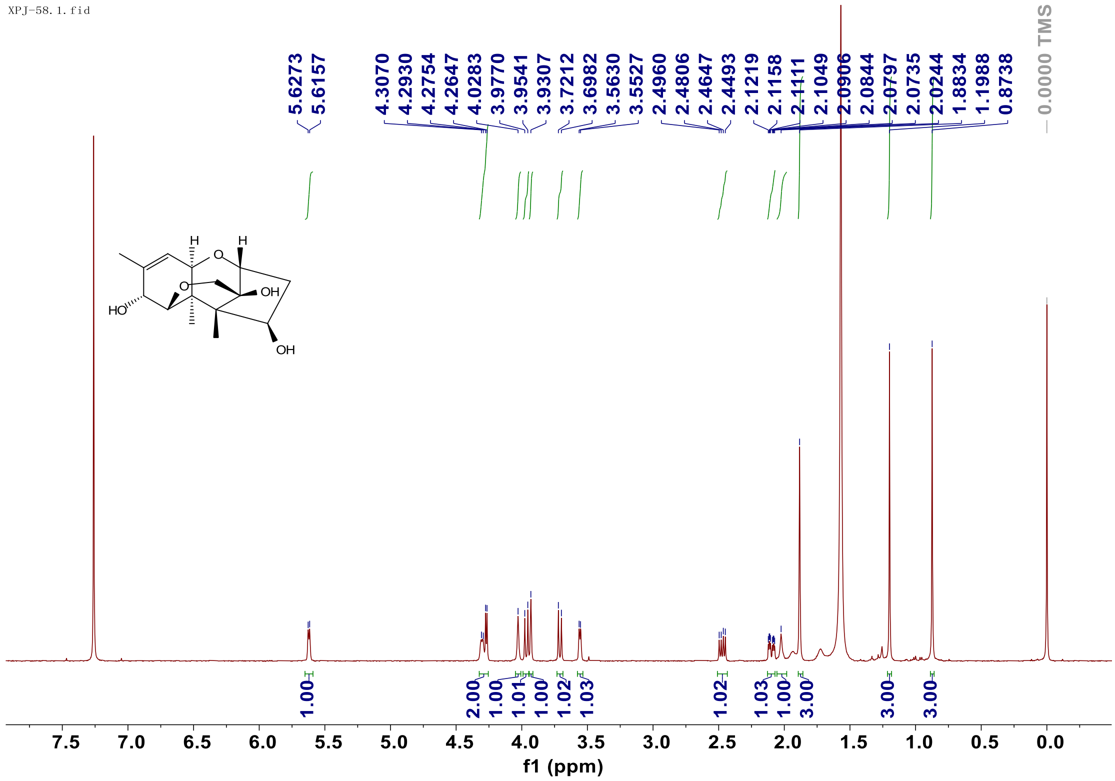

Figure S113. ^{1}H NMR spectrum of **11** (600 MHz, chloroform-*d*)

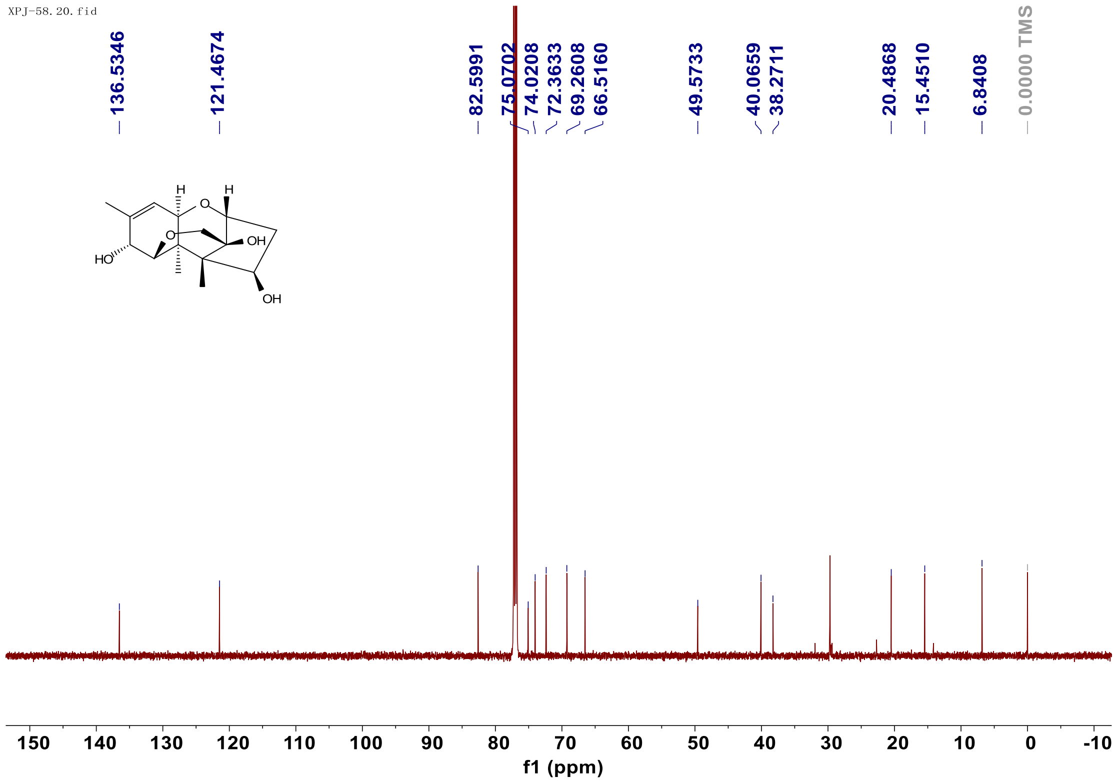

Figure S114. ^{13}C NMR spectrum of **11** (150 MHz, chloroform-*d*)

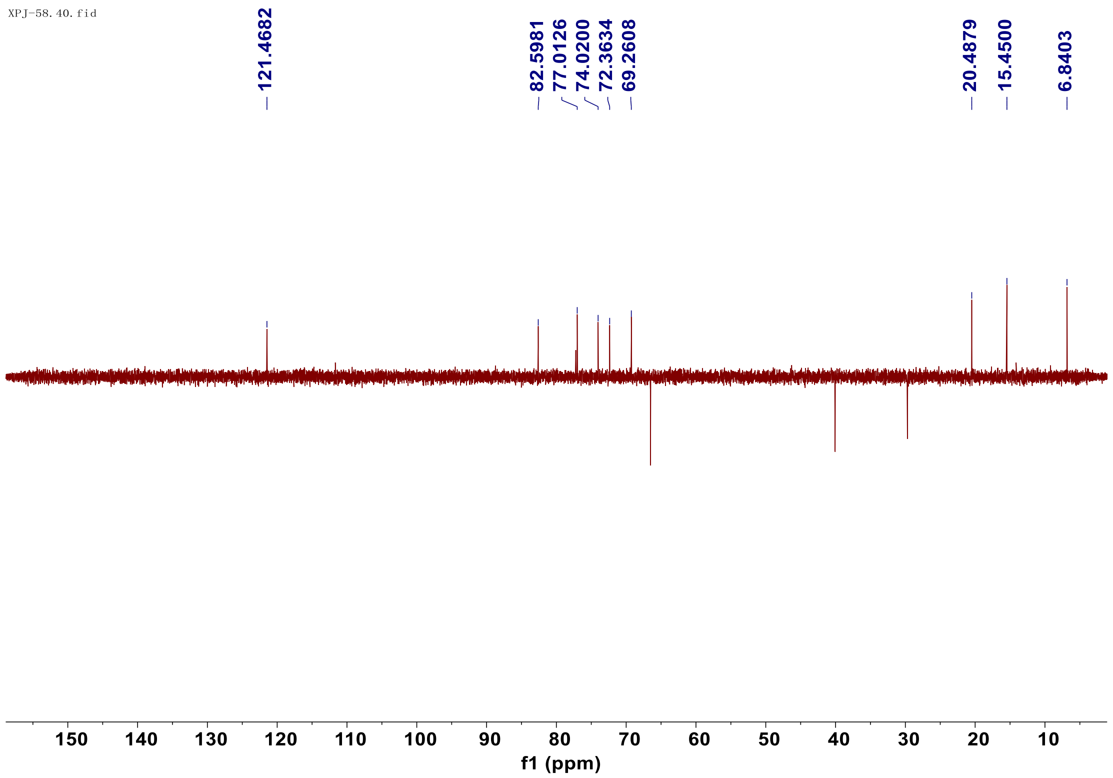

Figure S115. DEPT-135 spectrum of **11** (150 MHz, chloroform-*d*)

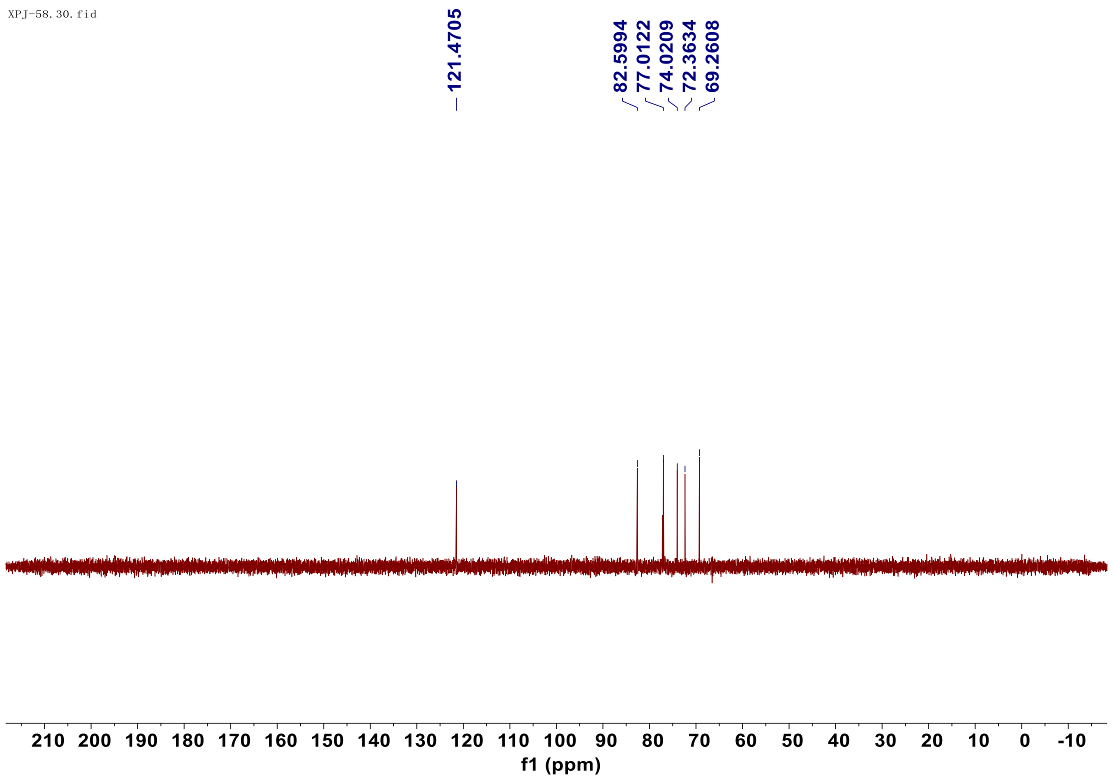

Figure S116. DEPT-90 spectrum of **11** (150 MHz, chloroform-*d*)

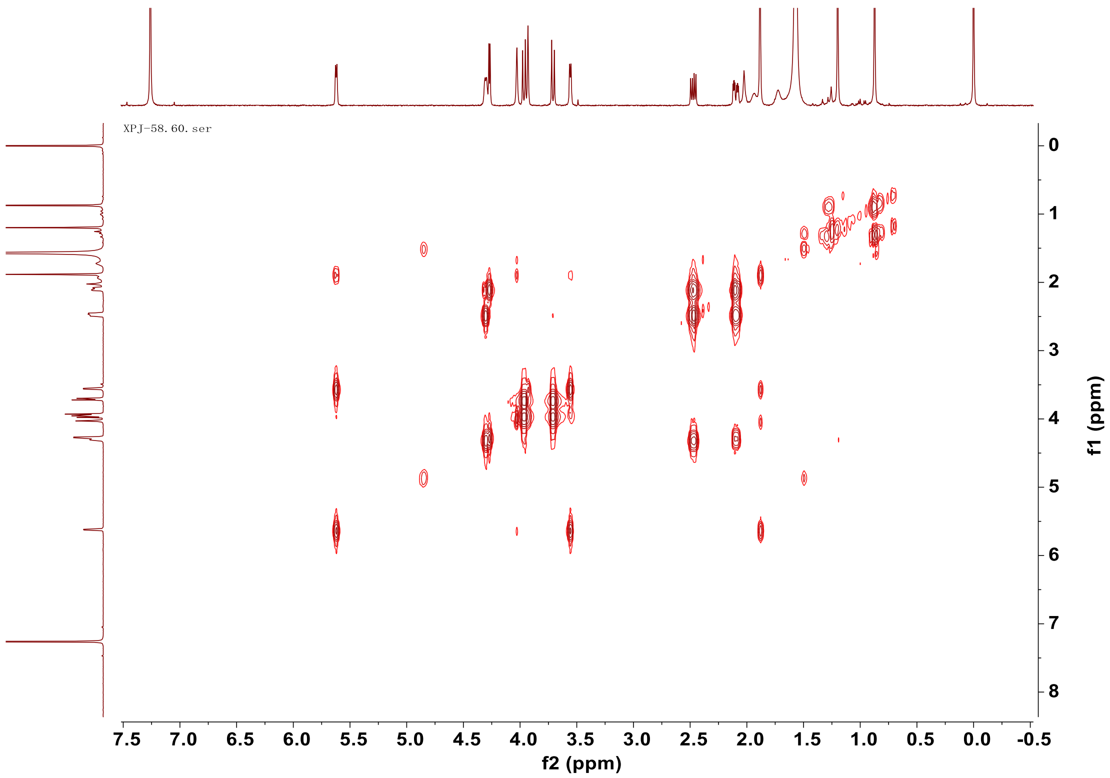

Figure S117. $^{1}H-^{1}H$ COSY spectrum of **11**

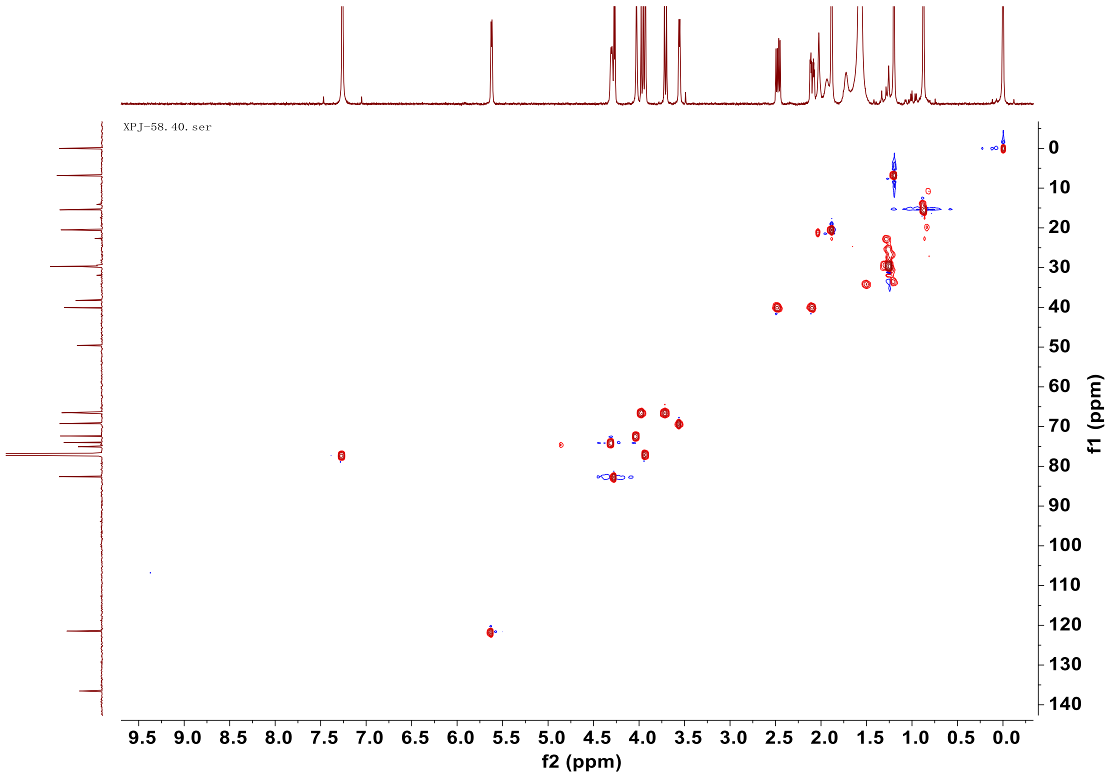

Figure S118. HSQC spectrum of **11**

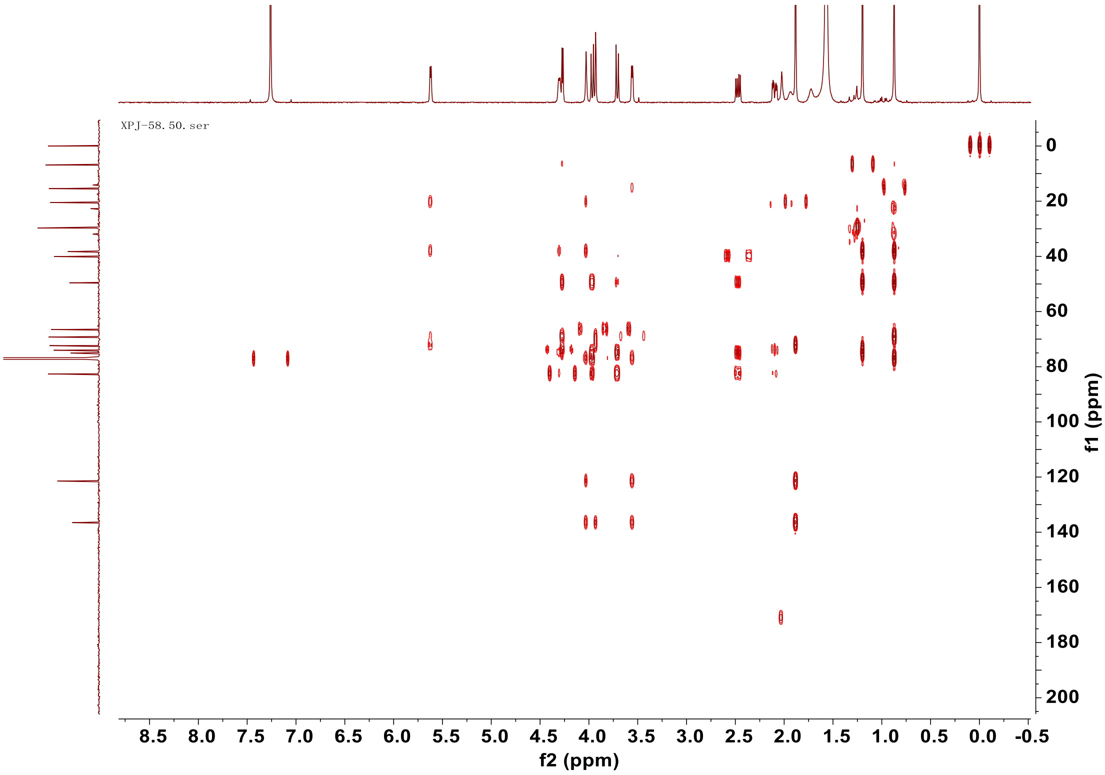

Figure S119. HMBC spectrum of **11**

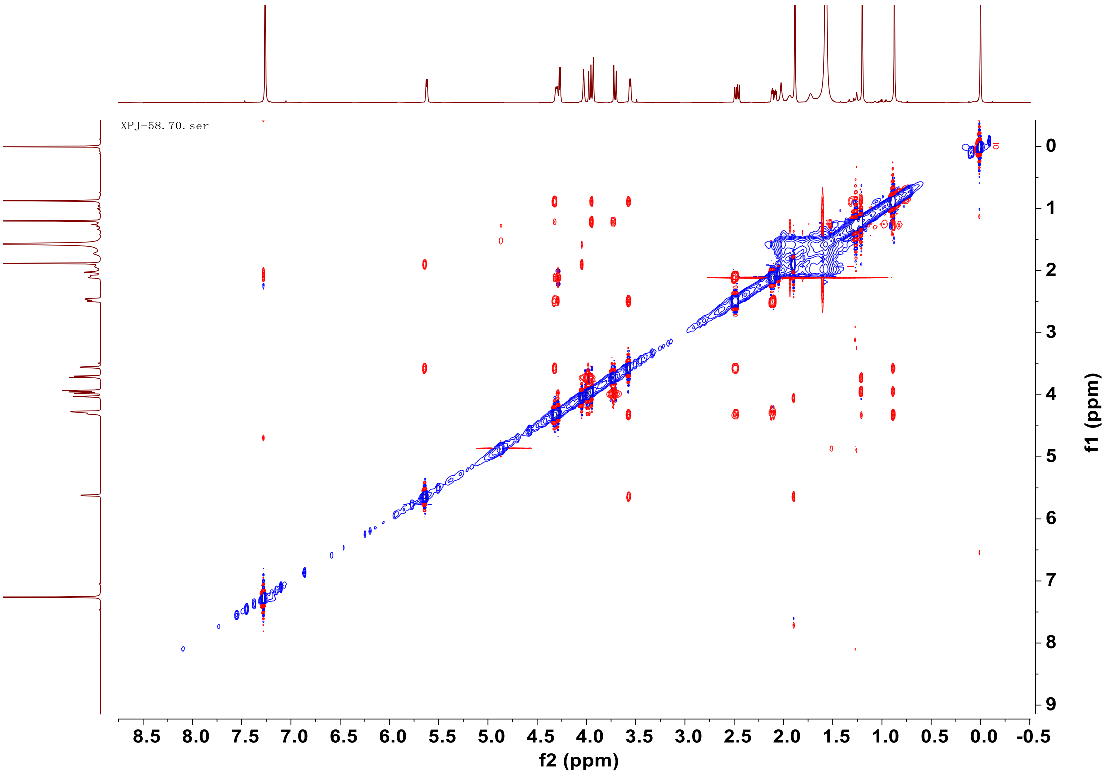

Figure S120. NOESY spectrum of **11**

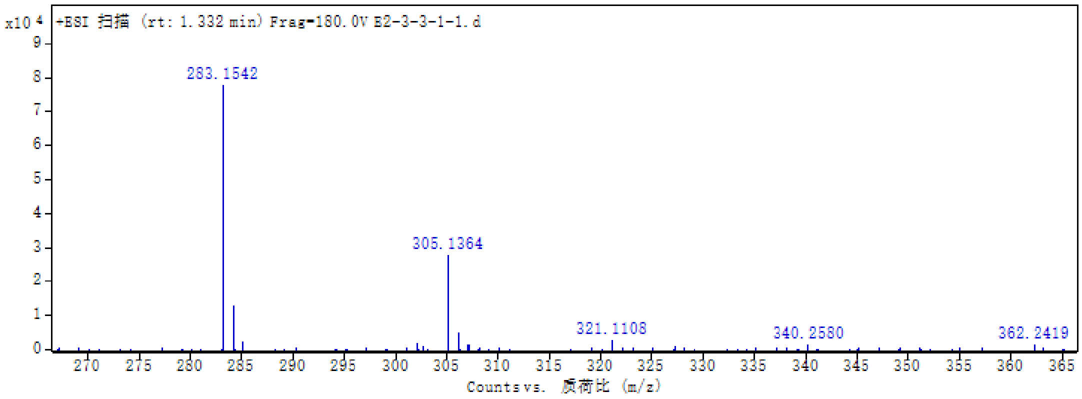

Figure S121. HRESIMS spectrum of **11**

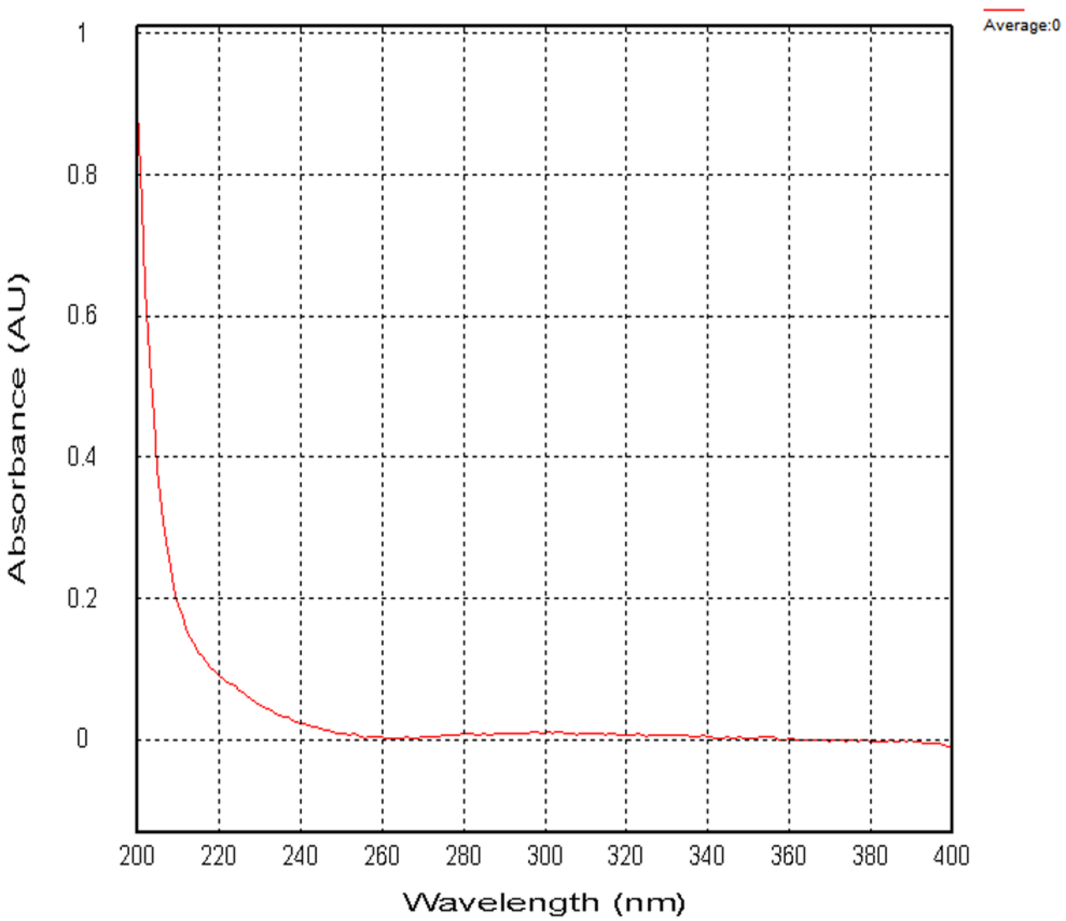

Figure S122. UV spectrum of **11**

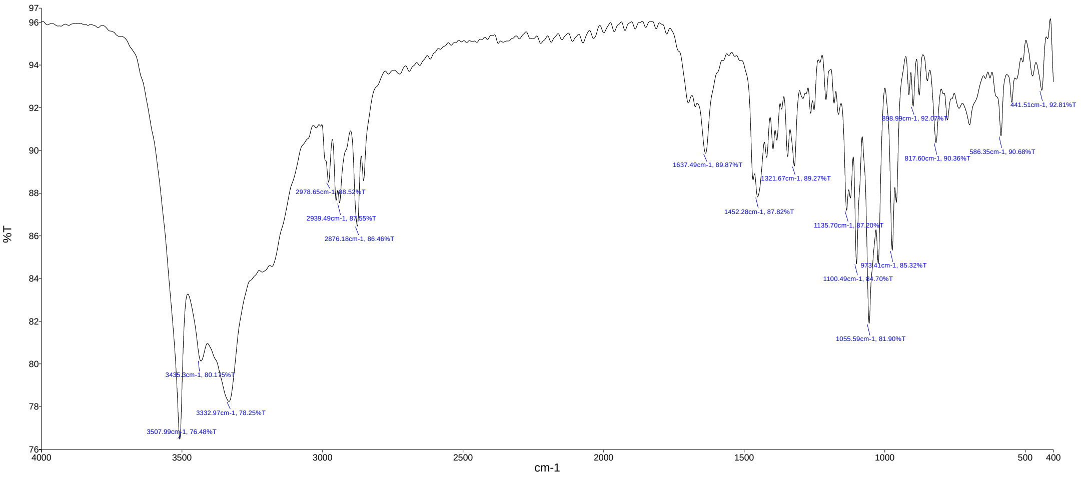

Figure S123. IR spectrum of **11**

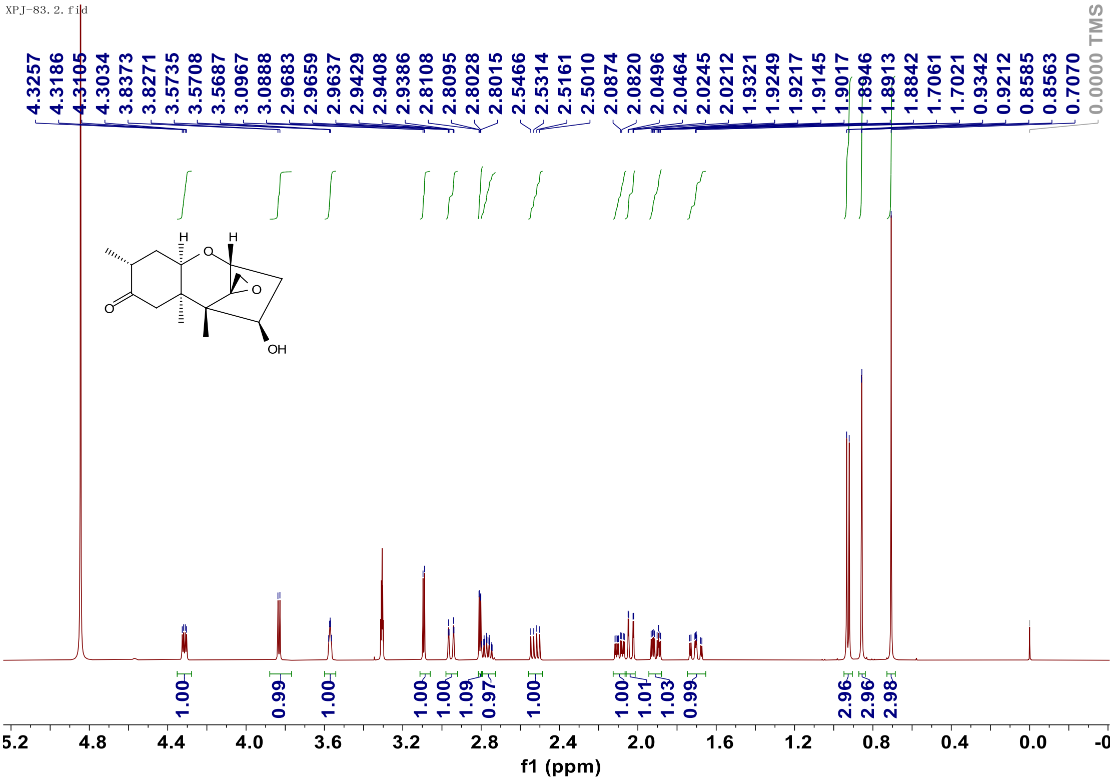

Figure S124. ^{1}H NMR spectrum of **12** (600 MHz, methanol-d_4)

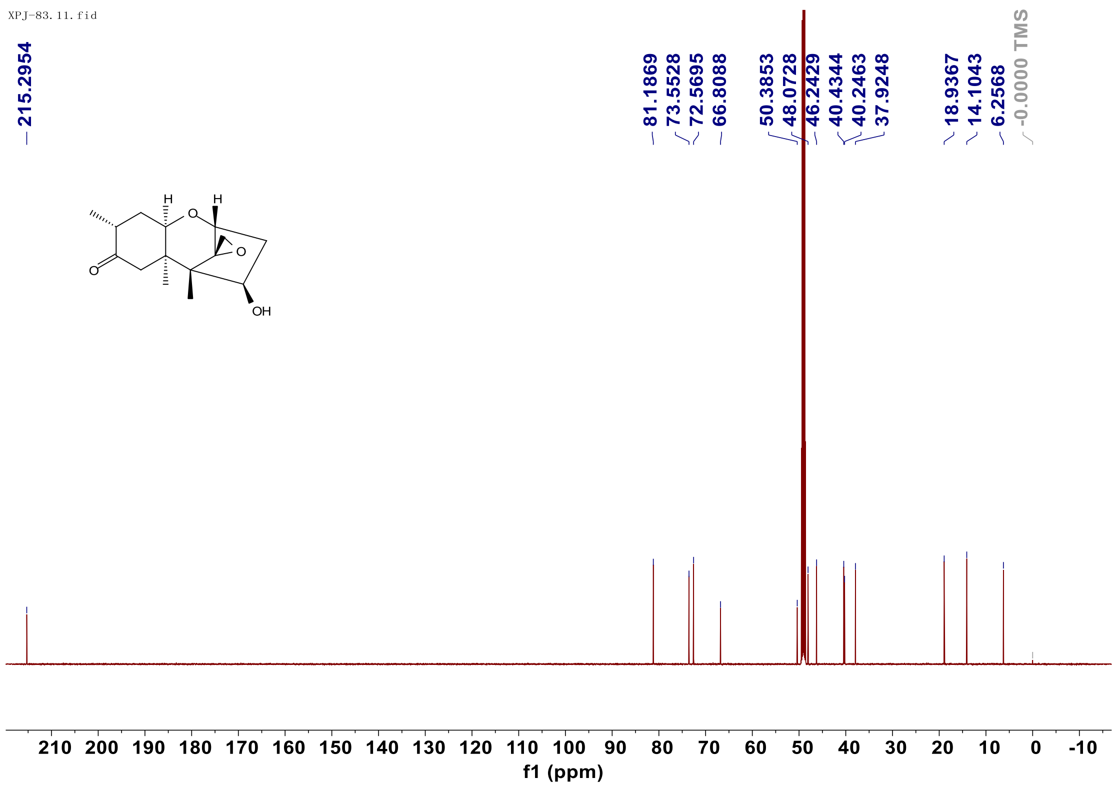

Figure S125. ^{13}C NMR spectrum of **12** (150 MHz, methanol-d_4)

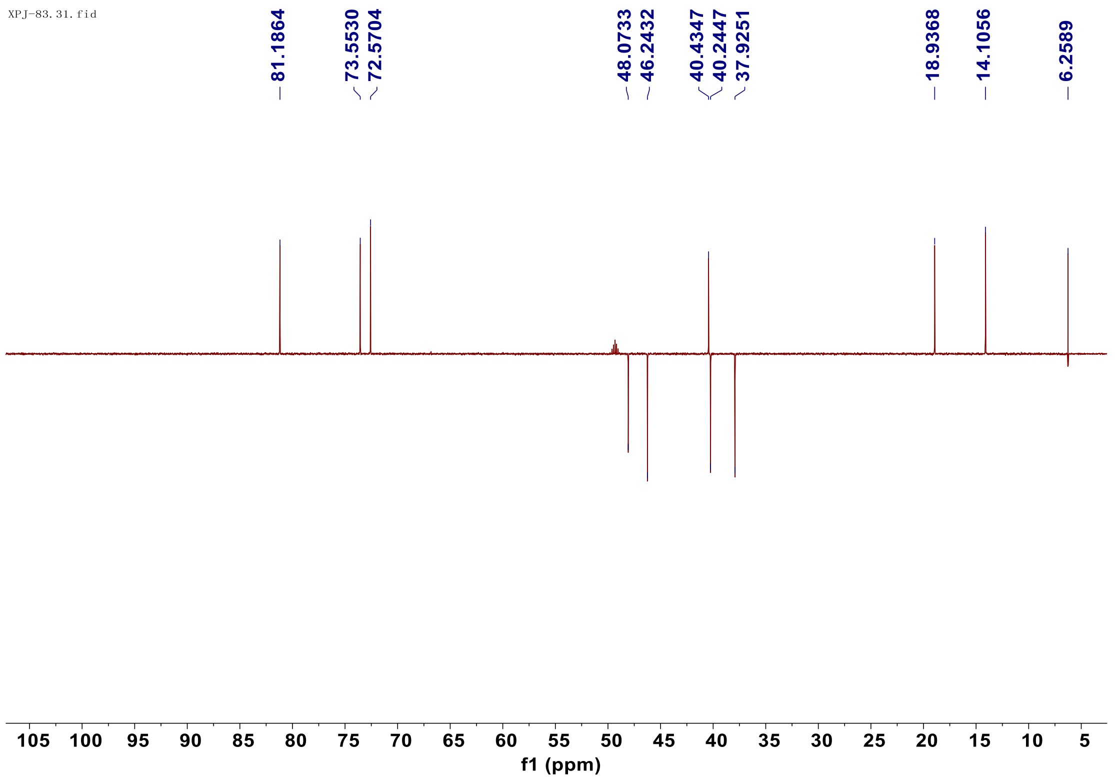

Figure S126. DEPT-135 spectrum of **12** (150 MHz, methanol-d_4)

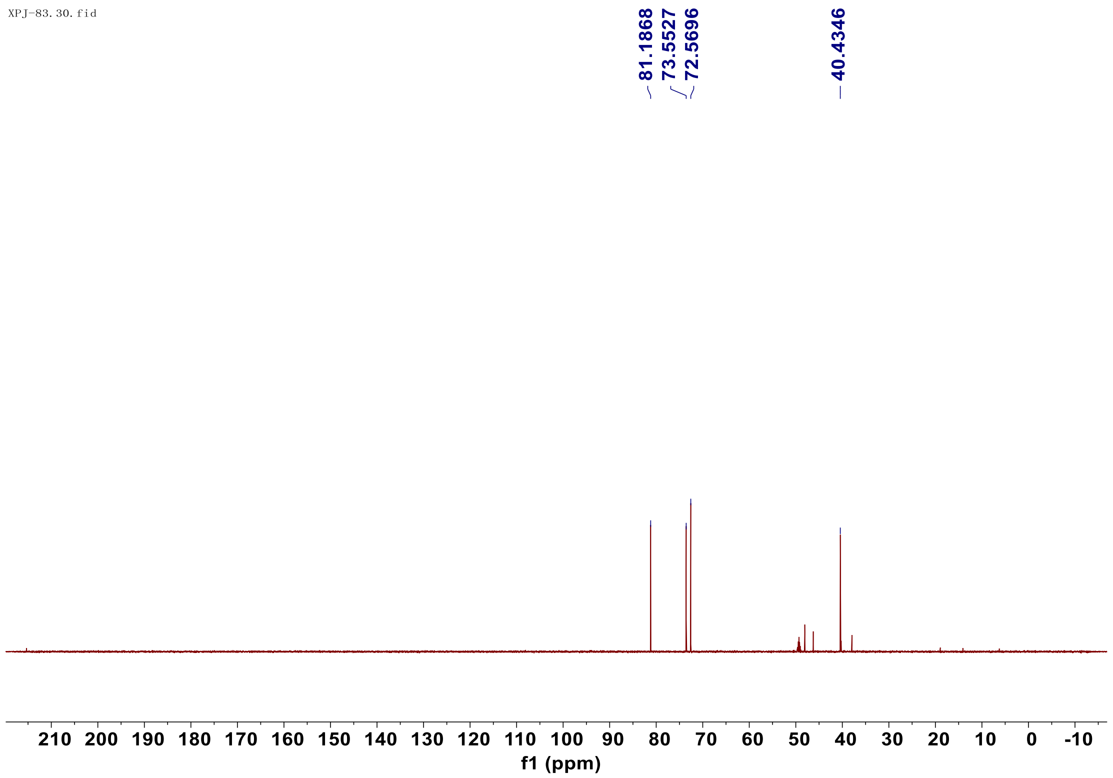

Figure S127. DEPT-90 spectrum of **12** (150 MHz, methanol-d_4)

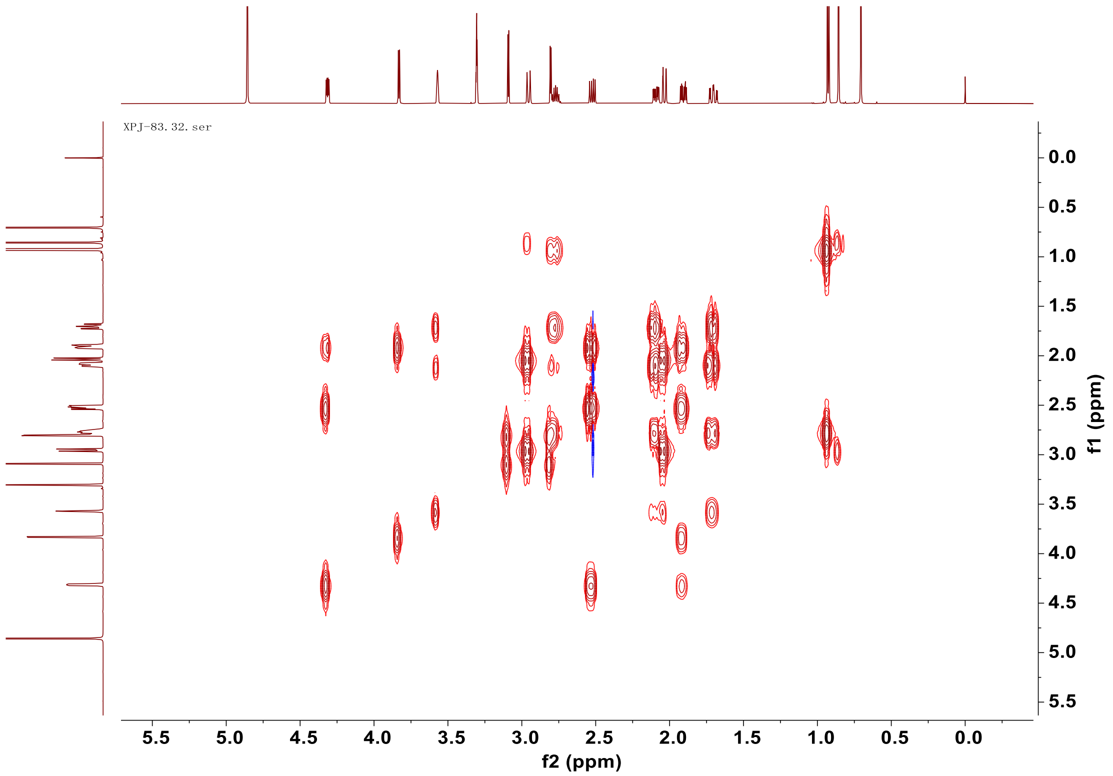

Figure S128. $^{1}H-^{1}H$ COSY spectrum of **12**

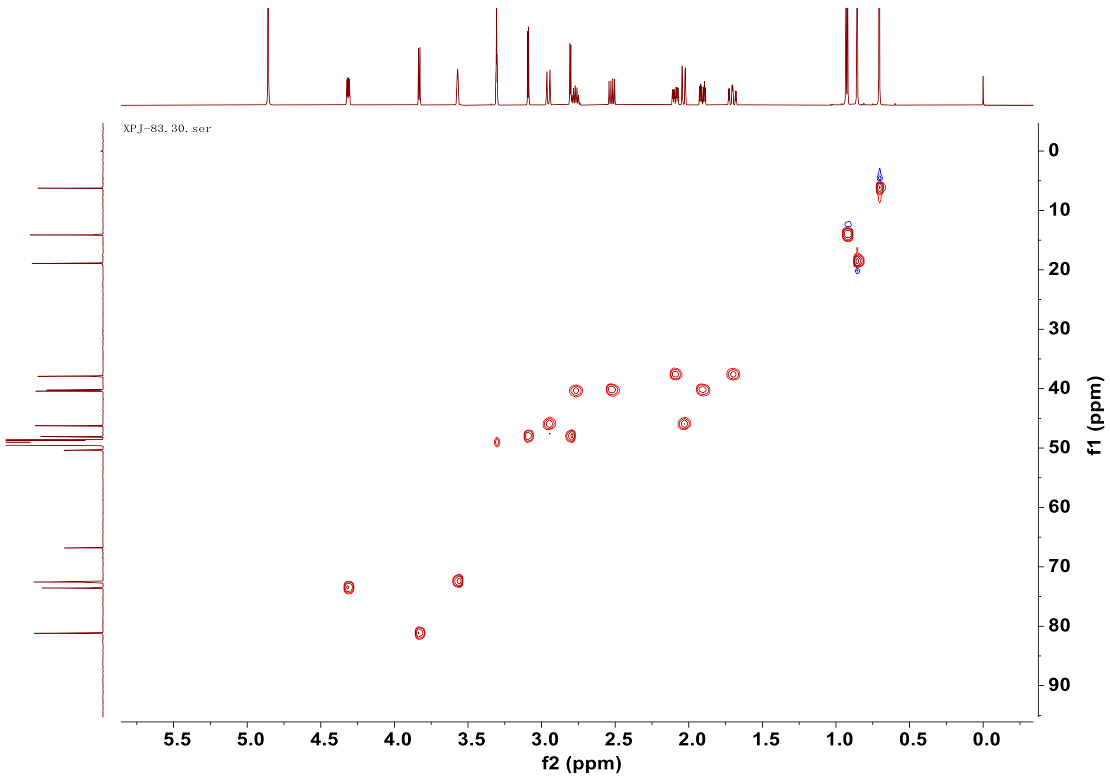

Figure S129. HSQC spectrum of **12**

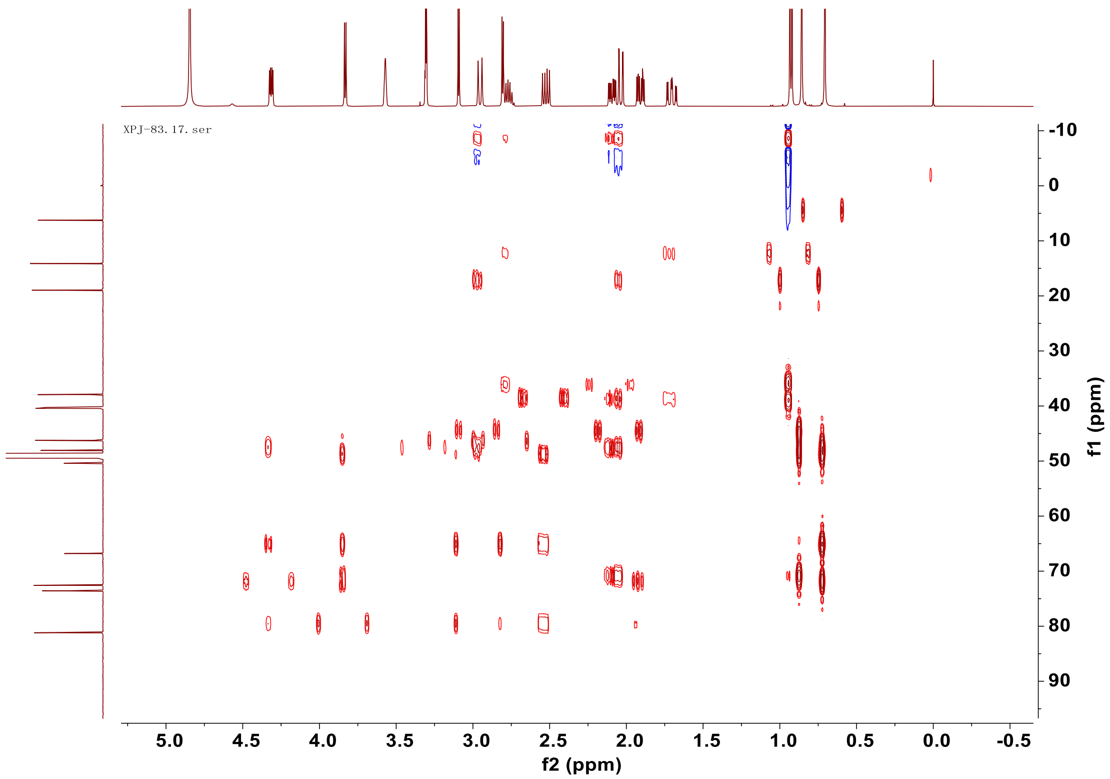

Figure S130. HMBC spectrum of **12**

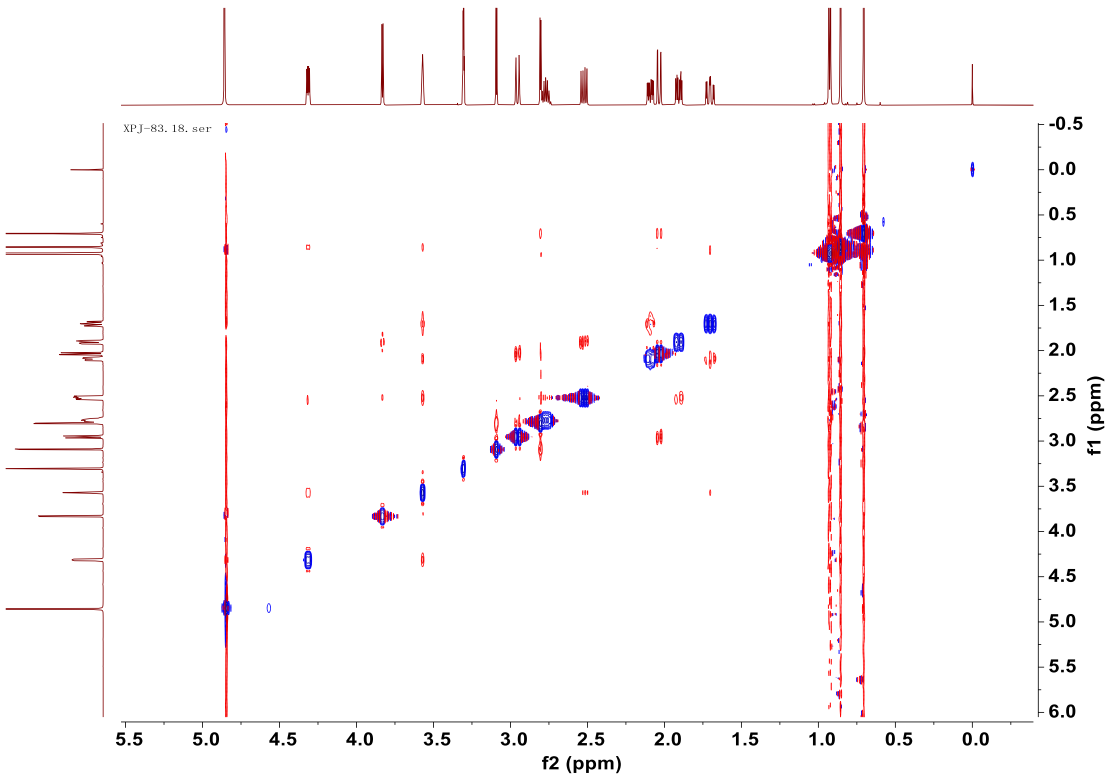

Figure S131. NOESY spectrum of **12**

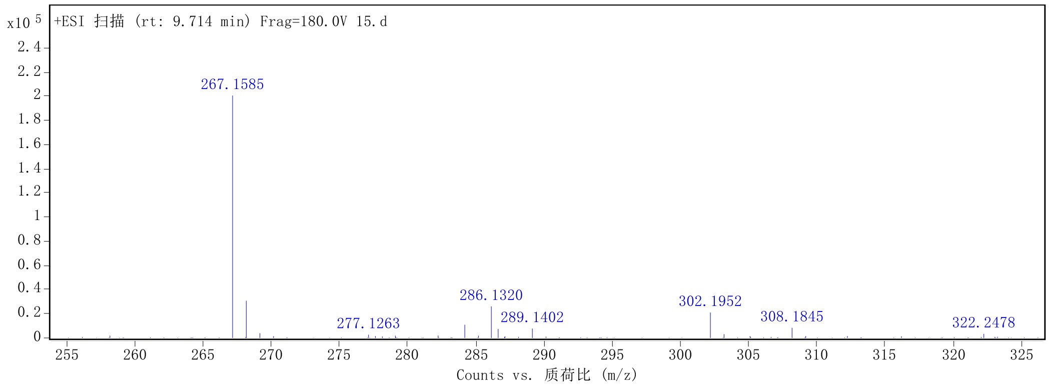

Figure S132. HRESIMS spectrum of **12**

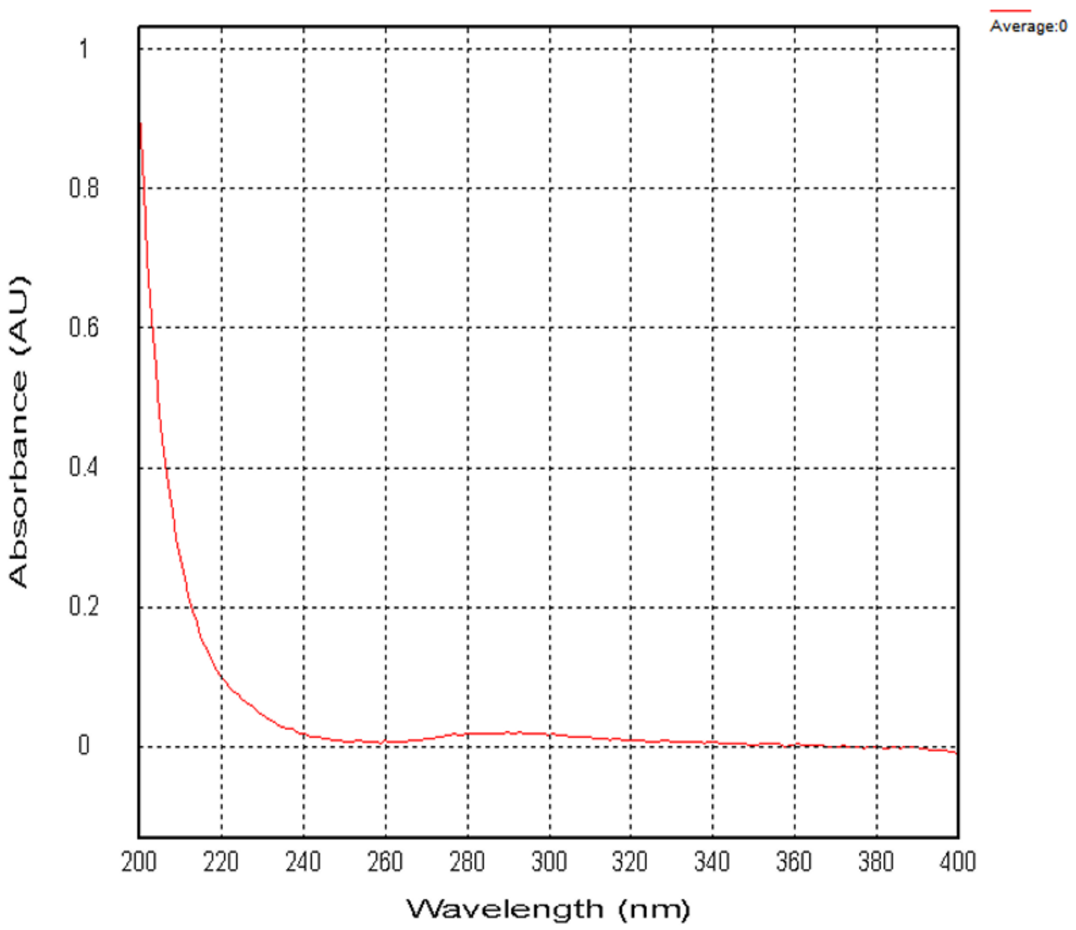

Figure S133. UV spectrum of **12**

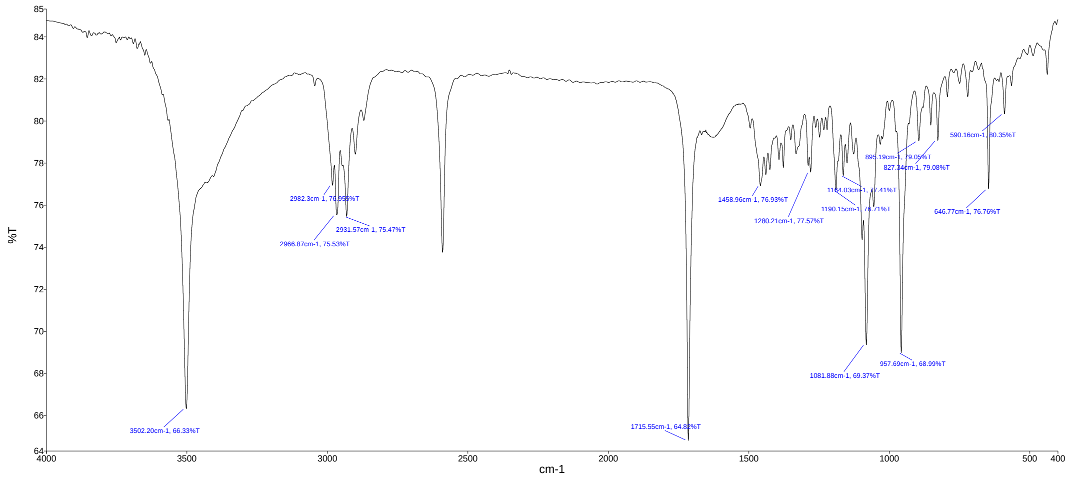

Figure S134. IR spectrum of **12**

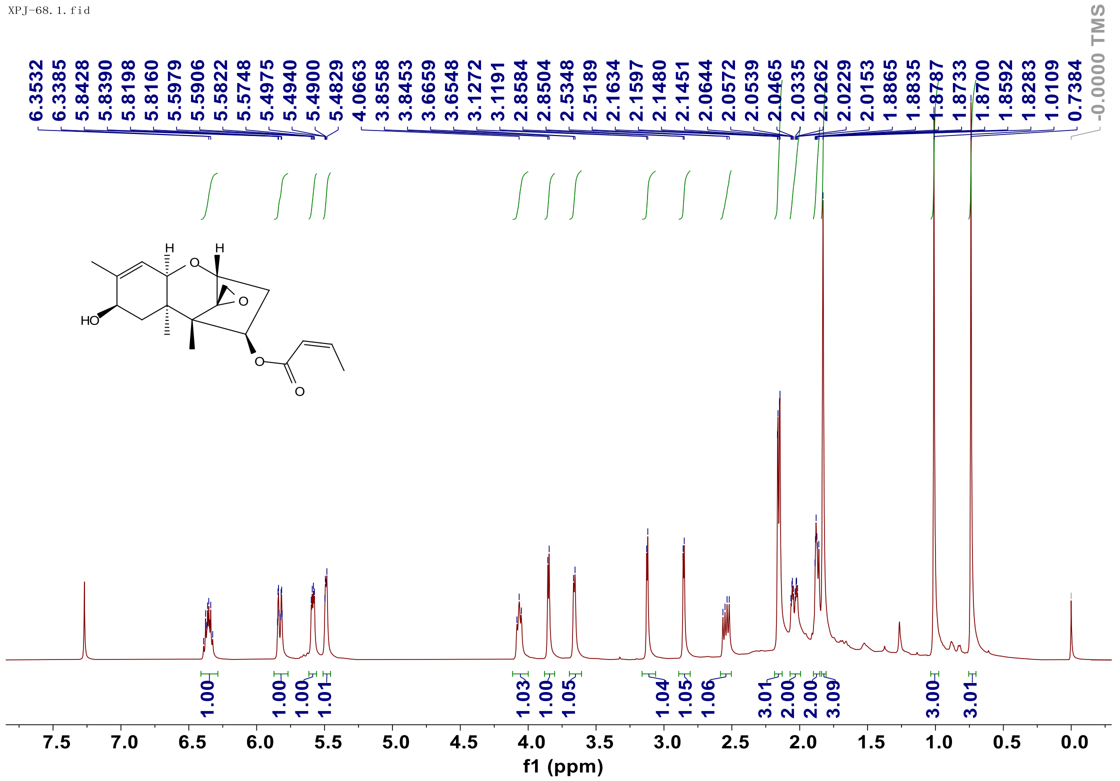

Figure S135. ^{1}H NMR spectrum of **13** (600 MHz, chloroform-*d*)

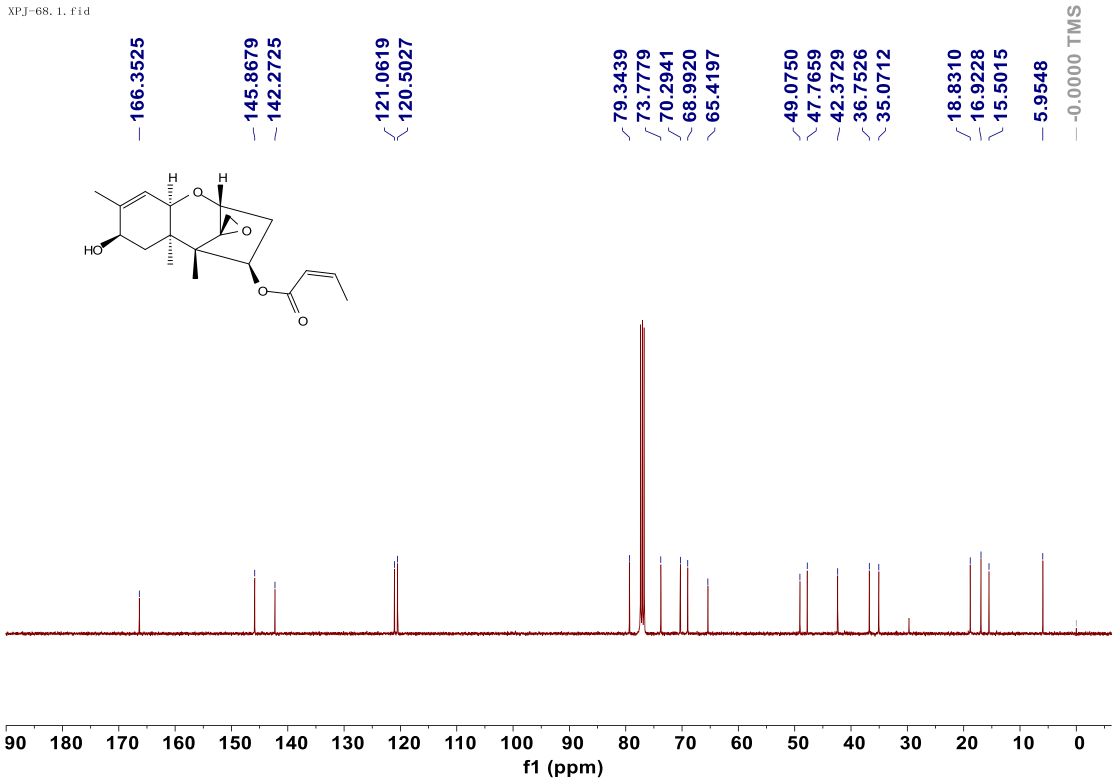

Figure S136. ^{13}C NMR spectrum of **13** (150 MHz, chloroform-*d*)

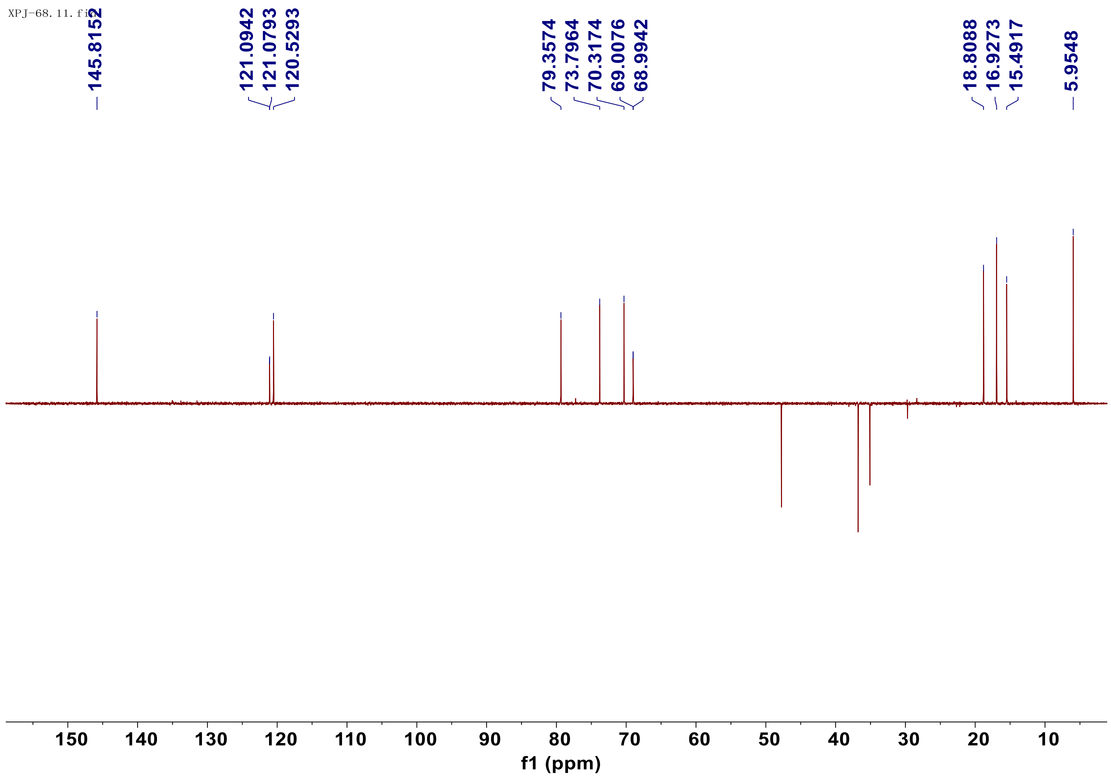

Figure S137. DEPT-135 spectrum of **13** (150 MHz, chloroform-*d*)

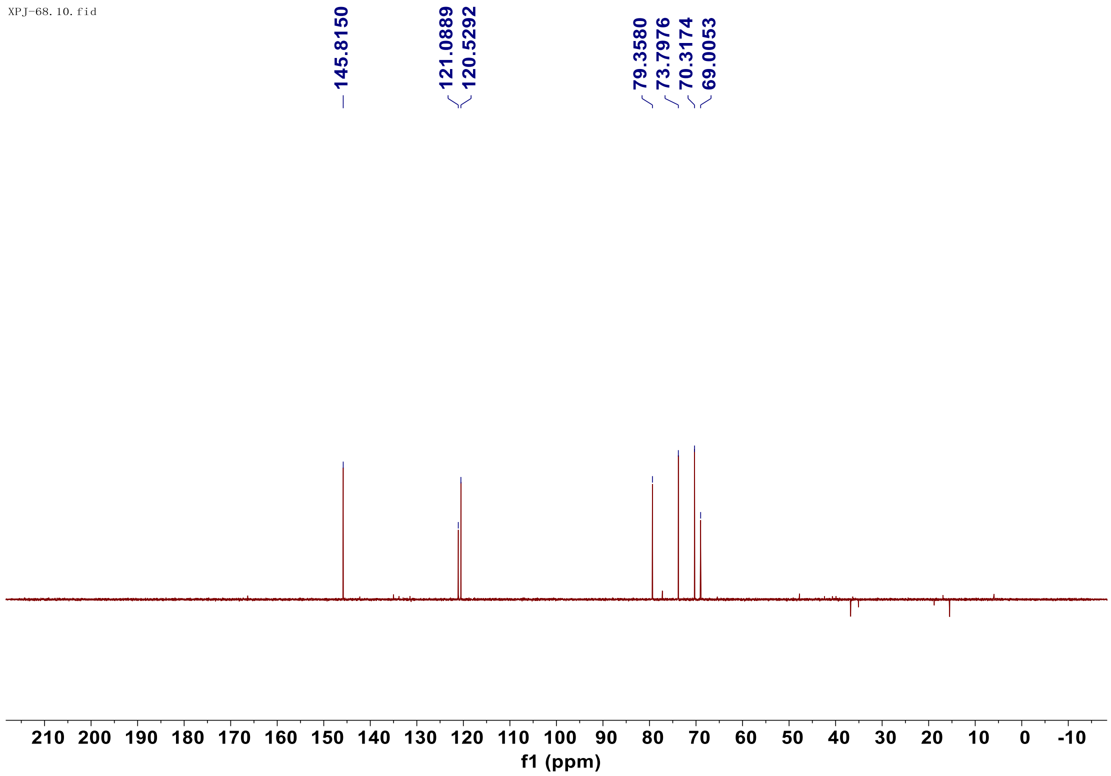

Figure S138. DEPT-90 spectrum of **13** (150 MHz, chloroform-*d*)

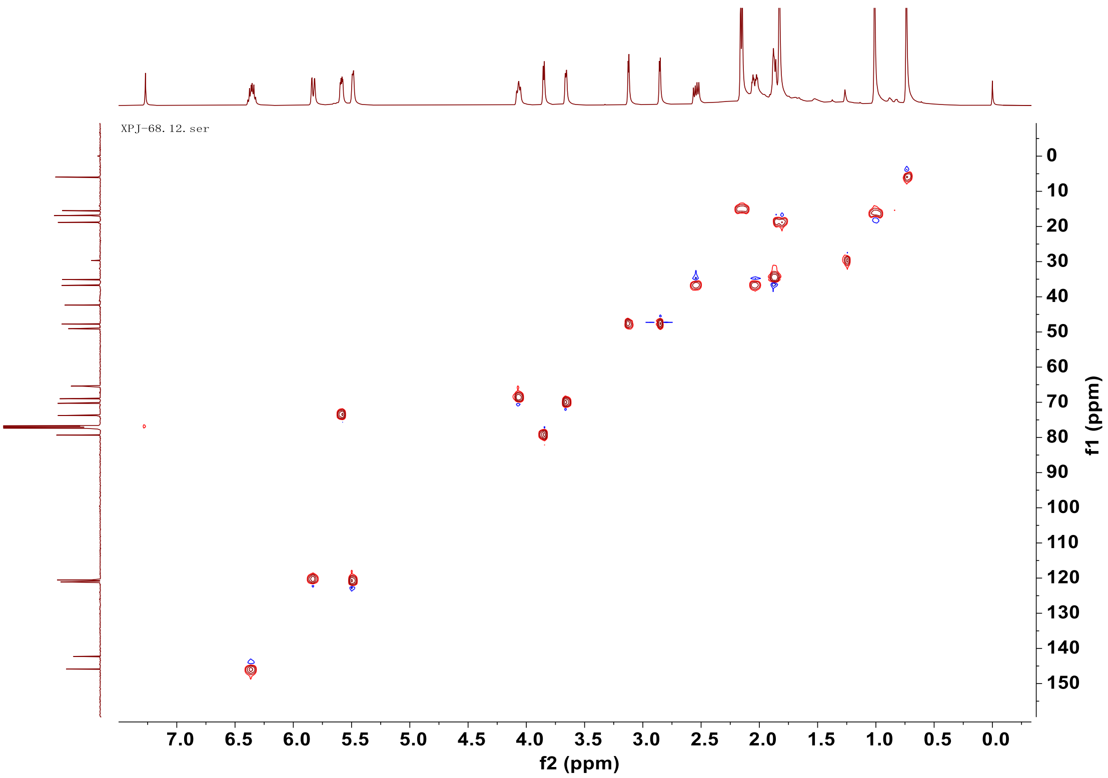

Figure S139. HSQC spectrum of **13**

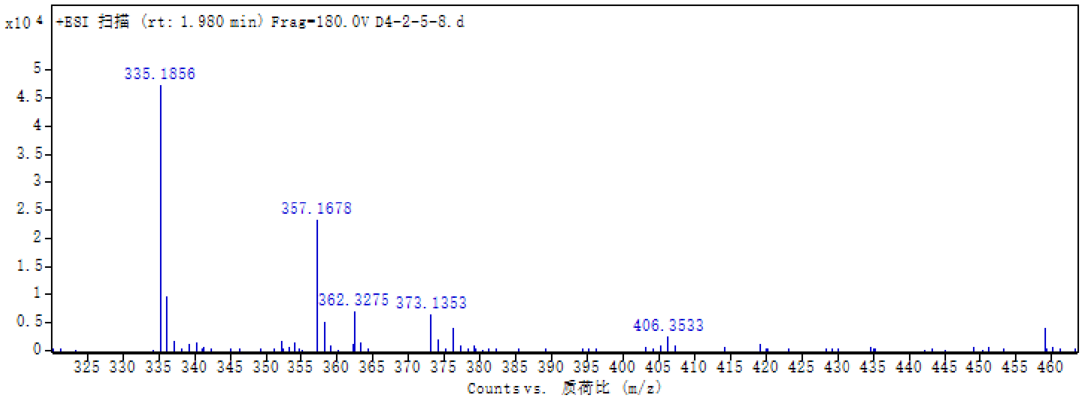

Figure S140. HRESIMS spectrum of **13**

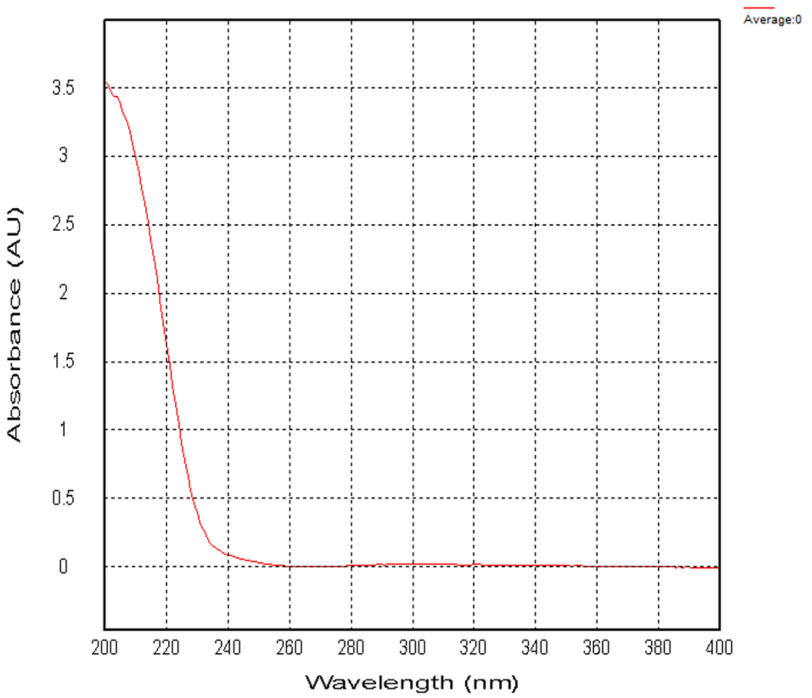

Figure S141. UV spectrum of **13**

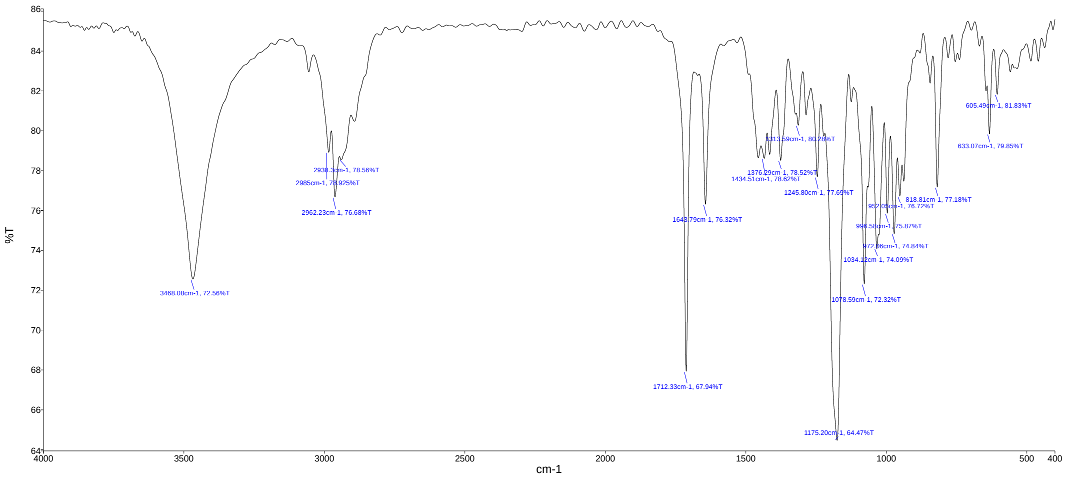

Figure S142. IR spectrum of **13**

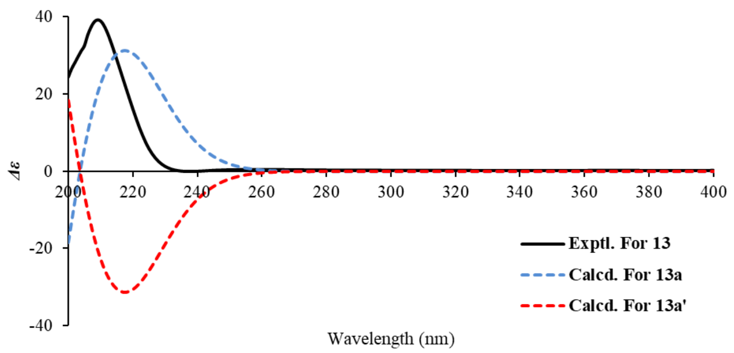

Figure S143. Calculated and experimental ECD spectra of **13**.

Table S1. Geometry data of conformers of structure **1a**

1a

1a-c1 , delta G = 0.0000 kcal/mol, population = 38.96 %

C 3.393729 -2.426034 0.444680
C 2.691561 1.839924 -0.816897
C 0.732417 0.070360 -1.029337
C 1.526446 -0.776420 -0.009021
C 2.836602 -1.370477 -0.492627
C -0.494340 0.722172 -0.274114
C 0.050124 1.668706 0.828526
C 0.715866 0.634542 1.762739
O 1.858652 0.027108 1.137349
C -0.418579 -0.381073 1.955197
C 4.854953 -2.733260 0.276569
C 0.225020 -0.816484 -2.176783
O -1.015316 2.321368 1.542421
C -1.181882 -0.378745 0.611861
O -2.558914 -0.002313 0.893618
C -1.473301 1.395259 -1.235892
O 2.281689 2.665167 0.160387
C 0.875751 2.876452 0.386329
O 2.699454 -3.000989 1.267347
O 3.890263 1.742557 -1.003184
H 1.077459 1.076086 2.695995
H 0.900915 -1.624250 0.304690
C -3.559560 -0.596194 0.178934
O -3.353578 -1.459152 -0.654998
C -4.850275 -0.040309 0.602584
C -6.038168 -0.294936 0.031112
C -6.356596 -1.161372 -1.134328
C 1.666644 1.134882 -1.678134
H 3.598913 -0.604939 -0.667250
H 2.696984 -1.882348 -1.454927
H -1.080943 -0.055263 2.761144
H -0.033842 -1.369464 2.220630
H 5.107709 -2.830889 -0.785087
H 5.432861 -1.882199 0.659599
H 5.130426 -3.636833 0.823084
H -0.507224 -1.553228 -1.834314
H -0.243442 -0.206144 -2.953451
H 1.053576 -1.354747 -2.645019
H -1.823065 1.789964 1.491514
H -1.201771 -1.353143 0.122897
H -2.182739 2.027121 -0.695996
H -0.957855 2.031138 -1.960196
H -2.046440 0.652351 -1.795836
H 0.421399 3.343057 -0.496814
H 0.852046 3.600273 1.203224
H -4.804981 0.644553 1.444618
H -6.895959 0.207958 0.479123
H -6.975055 -0.600476 -1.845494
H -5.471538 -1.549745 -1.634819
H -6.975495 -2.004669 -0.798551
H 1.062507 1.901993 -2.178131
H 2.246296 0.639480 -2.460932

1a-c9 , delta G = 0.1460 kcal/mol, population = 30.44 %

C 3.270435 -2.496668 0.417841
C 2.765386 1.810632 -0.819624
C 0.722427 0.135334 -1.021789
C 1.480029 -0.753214 -0.006547
C 2.767863 -1.392301 -0.493148
C -0.473855 0.831911 -0.258307
C 0.119119 1.741655 0.845953
C 0.720670 0.672168 1.779719
O 1.838083 0.019410 1.154997
C -0.467901 -0.280538 1.963791
C 4.711033 -2.882119 0.231487
C 0.175989 -0.722039 -2.174008
O -0.997180 2.367692 1.495257
C -1.203261 -0.256715 0.608061
O -2.592682 0.080476 0.840907
C -1.429866 1.559044 -1.201136
O 2.403921 2.641971 0.169957
C 1.008469 2.911897 0.415773
O 2.551813 -3.048256 1.235650
O 3.956694 1.661914 -1.021244
H 1.103236 1.086454 2.718998
H 0.822285 -1.580170 0.295796
C -3.537386 -0.556606 0.103102
O -3.278851 -1.394072 -0.746470
C -4.867374 -0.074302 0.506276
C -6.042584 -0.476419 -0.002590
C -6.319250 -1.477161 -1.067454
C 1.699896 1.159515 -1.672513

H 3.565871 -0.657440 -0.638600
H 2.616388 -1.867677 -1.472166
H -1.128146 0.095701 2.747665
H -0.136501 -1.283786 2.247651
H 4.955118 -2.960221 -0.833686
H 5.338253 -2.078973 0.639659
H 4.935736 -3.816754 0.748235
H 0.979386 -1.295139 -2.645066
H -0.590720 -1.425429 -1.837347
H -0.262876 -0.086048 -2.947760
H -0.677169 2.783961 2.308262
H -1.194001 -1.227165 0.110868
H -2.014058 0.846657 -1.789046
H -2.125941 2.176101 -0.630319
H -0.899470 2.210571 -1.901276
H 0.568420 3.416408 -0.453575
H 1.038190 3.628196 1.240551
H -4.864351 0.677310 1.290697
H -6.928634 -0.004929 0.424598
H -6.911812 -1.006229 -1.862356
H -5.416731 -1.912253 -1.491946
H -6.951887 -2.275371 -0.657158
H 1.126228 1.957052 -2.161190
H 2.248840 0.643830 -2.464403

1a-c10 , delta G = 0.6774 kcal/mol, population = 12.40 %
C 3.295379 -2.465920 0.461547
C 2.747875 1.810851 -0.840612
C 0.720149 0.114056 -1.024033
C 1.483610 -0.755068 0.002454
C 2.779950 -1.386255 -0.471813
C -0.482552 0.808225 -0.267191
C 0.101788 1.733190 0.836948
C 0.707916 0.676094 1.776824
O 1.830858 0.033792 1.156151
C -0.470780 -0.288614 1.962807
C 4.745036 -2.825210 0.295498
C 0.186765 -0.763502 -2.167496
O -0.949794 2.339753 1.602250
C -1.208549 -0.274489 0.608242
O -2.596056 0.072533 0.843019
C -1.438820 1.512433 -1.227436
O 2.383998 2.643243 0.146782
C 0.990041 2.905618 0.407885
O 2.579350 -3.018460 1.280918
O 3.939672 1.674462 -1.048368
H 1.081272 1.102483 2.712932
H 0.832769 -1.585026 0.311660
C -3.546647 -0.558505 0.106309
O -3.294166 -1.399512 -0.741303
C -4.872328 -0.064911 0.509099
C -6.049949 -0.448449 -0.008572
C -6.333633 -1.434767 -1.084875
C 1.686244 1.142535 -1.684682
H 3.568187 -0.643948 -0.631420
H 2.636158 -1.884198 -1.440712
H -1.132181 0.081540 2.748045
H -0.128236 -1.288224 2.246298
H 5.351218 -2.004643 0.700968
H 4.982543 -3.748781 0.826222
H 5.002814 -2.910673 -0.765852
H -0.245969 -0.142004 -2.956225
H 0.996875 -1.341041 -2.621265
H -0.580738 -1.464291 -1.827059
H -1.484136 2.894976 1.019131
H -1.203906 -1.248681 0.118060
H -2.190589 2.095767 -0.688618
H -0.916669 2.189650 -1.908514
H -1.984080 0.785236 -1.834091
H 0.546091 3.419963 -0.455568
H 1.017371 3.605568 1.244983
H -4.863909 0.679888 1.299945
H -6.932245 0.029285 0.419418
H -6.921169 -0.949817 -1.875016
H -5.434424 -1.873255 -1.512882
H -6.973558 -2.232113 -0.684266
H 1.104646 1.931341 -2.178174
H 2.238059 0.626060 -2.474050

1a-c2 , delta G = 0.7776 kcal/mol, population = 10.47 %
C 2.903750 -2.837318 -0.217076
C 2.834464 1.799595 -0.709318
C 0.919893 -0.003998 -0.815101
C 1.658594 -0.675413 0.365080
C 2.986744 -1.342560 0.028125
C -0.363271 0.713170 -0.243617

C 0.094550 1.827486 0.736975
C 0.718112 0.959809 1.852584
O 1.911047 0.295671 1.398451
C -0.410951 -0.042759 2.127657
C 4.100618 -3.443829 -0.893533
C 0.529037 -1.066804 -1.852199
O -1.027326 2.546907 1.279560
C -1.080531 -0.268253 0.754293
O -2.484432 0.087894 0.888353
C -1.292854 1.206202 -1.351158
O 2.355809 2.765917 0.093903
C 0.934762 2.981160 0.188542
O 1.943140 -3.508636 0.124615
O 4.041019 1.657414 -0.765413
H 1.012694 1.544586 2.728978
H 1.017679 -1.472650 0.763490
C -3.410121 -0.642760 0.199213
O -3.114055 -1.604538 -0.486292
C -4.745317 -0.083990 0.443736
C -5.895438 -0.537281 -0.080526
C -6.127029 -1.675537 -1.008182
C 1.866495 0.994363 -1.546158
H 3.655494 -1.232649 0.895469
H 3.521847 -0.872622 -0.802428
H -1.130619 0.391697 2.825788
H -0.034304 -0.967601 2.572794
H 4.082932 -4.532073 -0.814657
H 4.076915 -3.155805 -1.952795
H 5.029232 -3.038714 -0.477510
H 1.411714 -1.636869 -2.160073
H -0.211186 -1.772911 -1.464558
H 0.118592 -0.597078 -2.750417
H -1.822778 1.996488 1.243103
H -1.034588 -1.303668 0.415666
H -1.780857 0.368127 -1.854387
H -2.075003 1.856270 -0.951414
H -0.751978 1.779292 -2.108706
H 0.539436 3.321344 -0.776866
H 0.846511 3.806036 0.898473
H -4.771543 0.774627 1.108722
H -6.794547 0.006103 0.212640
H -5.211671 -2.187296 -1.298075
H -6.813546 -2.391009 -0.536654
H -6.646502 -1.311067 -1.903859
H 1.274061 1.687825 -2.154327
H 2.487792 0.431878 -2.246650

1a-c4 , delta G = 1.4806 kcal/mol, population = 3.19 %

C 3.395854 -2.382641 0.528910
C 2.595982 1.917885 -0.564423
C 0.804195 0.058654 -1.146355
C 1.508924 -0.809640 -0.078900
C 2.909212 -1.292642 -0.409436
C -0.552209 0.584901 -0.527715
C -0.222481 1.483725 0.693369
C 0.377503 0.428103 1.647425
O 1.630015 -0.071497 1.150864
C -0.705887 -0.658492 1.622861
C 4.884493 -2.578292 0.577578
C 0.512289 -0.778454 -2.400946
O -1.414806 2.024538 1.292158
C -1.275181 -0.613377 0.187616
O -2.703792 -0.340576 0.290666
C -1.437137 1.264101 -1.571953
O 2.007167 2.649603 0.395662
C 0.573062 2.763974 0.441011
O 2.628894 -3.065419 1.188695
O 3.812491 1.901635 -0.591363
H 0.582353 0.825612 2.645800
H 0.903848 -1.711808 0.090037
C -3.546795 -0.948421 -0.580171
O -3.171408 -1.734955 -1.434229
C -4.953210 -0.560376 -0.417389
C -5.534446 0.118820 0.584927
C -4.949162 0.669054 1.841413
C 1.741248 1.217454 -1.597495
H 3.635352 -0.474200 -0.418945
H 2.938424 -1.736529 -1.414387
H -1.491159 -0.418173 2.343617
H -0.300552 -1.639139 1.885893
H 5.141454 -3.500980 1.100681
H 5.307037 -2.575515 -0.433331
H 5.326742 -1.722738 1.104490
H 0.103867 -0.149840 -3.196634
H 1.429954 -1.231080 -2.786052
H -0.202380 -1.582023 -2.200034
H -2.174750 1.463277 1.081510

H -1.159401 -1.552132 -0.353493
H -2.257523 1.811360 -1.100957
H -0.875600 1.980570 -2.177532
H -1.875684 0.529004 -2.250957
H 0.206620 3.263056 -0.465022
H 0.394726 3.427671 1.289307
H -5.572730 -0.905386 -1.240839
H -6.603883 0.293549 0.464232
H -5.705243 0.674957 2.631747
H -4.069343 0.122246 2.181447
H -4.653152 1.716317 1.683171
H 1.156745 1.981879 -2.124158
H 2.449761 0.815117 -2.325945

1a-c8 , delta G = 1.5596 kcal/mol, population = 2.79 %

C 3.342041 -2.841504 0.763474
C 1.756590 2.368982 -1.528141
C 1.030479 -0.064213 -0.789027
C 1.658468 -1.008309 0.268952
C 2.983879 -1.647139 -0.103257
C -0.203455 0.651232 -0.115138
C 0.312812 1.493726 1.092191
C 0.752118 0.351071 2.039463
O 1.907875 -0.308516 1.501080
C -0.470202 -0.566198 2.051495
C 4.780275 -3.273411 0.707450
C 0.560474 -0.882059 -2.001430
O -0.740150 2.258375 1.695921
C -1.073374 -0.425062 0.641519
O -2.431839 0.068194 0.801220
C -1.064408 1.410434 -1.132382
O 1.399082 3.110090 -0.456533
C 1.440834 2.484507 0.851951
O 2.515302 -3.423828 1.445951
O 1.787609 2.912313 -2.615131
H 1.044107 0.713685 3.029950
H 0.950789 -1.830507 0.450266
C -3.423872 -0.465521 0.030717
O -3.229871 -1.361461 -0.770983
C -4.692652 0.198576 0.353136
C -5.864831 -0.002810 -0.269749
C -6.186890 -0.905554 -1.406053
C 2.136647 0.932272 -1.286199
H 3.807870 -0.925324 -0.060447
H 2.966157 -2.022125 -1.135773
H -1.188425 -0.223244 2.801101
H -0.202735 -1.597196 2.298661
H 5.135218 -3.295746 -0.328828
H 5.388336 -2.525435 1.232230
H 4.913101 -4.247732 1.180663
H 0.185453 -0.222161 -2.788453
H 1.391264 -1.453175 -2.425306
H -0.233134 -1.586824 -1.738206
H -1.579270 1.780588 1.626610
H -1.124820 -1.366501 0.093908
H -0.474118 1.926470 -1.889911
H -1.721716 0.716203 -1.662794
H -1.689222 2.155430 -0.634954
H 1.327936 3.311612 1.555499
H 2.418468 2.025417 1.010631
H -4.643293 0.919115 1.164581
H -6.705713 0.581608 0.105651
H -6.912382 -1.659956 -1.072852
H -6.692993 -0.331502 -2.192122
H -5.313354 -1.407760 -1.817213
H 2.548612 0.559329 -2.225950
H 2.960984 0.950983 -0.564911

1a-c6 , delta G = 1.8414 kcal/mol, population = 1.74 %

C 3.355036 -2.936466 0.622866
C 1.803908 2.348720 -1.519679
C 1.059196 -0.095454 -0.836019
C 1.687190 -1.072629 0.191668
C 3.001715 -1.716323 -0.209061
C -0.160560 0.614864 -0.132623
C 0.375749 1.421127 1.090723
C 0.811457 0.250590 2.004748
O 1.955286 -0.407043 1.438878
C -0.420364 -0.654044 2.005257
C 4.787233 -3.384019 0.538326
C 0.569914 -0.877681 -2.064338
O -0.663593 2.181454 1.722834
C -1.034381 -0.471571 0.604860
O -2.386876 0.029227 0.789246
C -1.023466 1.407965 -1.121631
O 1.465171 3.065717 -0.425832

C 1.512282 2.405722 0.865431
O 2.531458 -3.521479 1.306868
O 1.830308 2.919551 -2.592624
H 1.116346 0.584584 3.001398
H 0.972285 -1.891539 0.359271
C -3.388420 -0.469003 0.007150
O -3.206994 -1.339347 -0.825166
C -4.647169 0.201439 0.355168
C -5.837590 -0.005616 -0.230307
C -6.196796 -0.925798 -1.340941
C 2.171008 0.902357 -1.318756
H 3.834524 -1.005219 -0.156522
H 2.970392 -2.064640 -1.250511
H -1.128174 -0.322465 2.769757
H -0.161351 -1.693568 2.224065
H 5.406451 -2.666654 1.092310
H 4.910772 -4.378603 0.970198
H 5.137507 -3.366299 -0.499416
H 1.390258 -1.448798 -2.508268
H -0.231173 -1.578238 -1.812785
H 0.197956 -0.193782 -2.832046
H -1.507488 1.712221 1.653004
H -1.099460 -1.398314 0.034189
H -0.435992 1.942710 -1.868294
H -1.688651 0.733086 -1.666851
H -1.640619 2.141968 -0.598817
H 1.415179 3.215153 1.591509
H 2.486302 1.932100 1.002144
H -4.574382 0.929987 1.157651
H -6.665185 0.588096 0.159856
H -6.648719 -0.346771 -2.156725
H -5.349523 -1.493342 -1.720357
H -6.977687 -1.617530 -0.998742
H 2.569313 0.549505 -2.272047
H 3.002737 0.893085 -0.605770

Table S2. Geometry data of conformers of structure **2a**

2a

2a-c22 , delta G = 0.0000 kcal/mol, population = 82.09 %

C -3.840293 1.883336 1.052537
C -1.806872 -0.860631 -1.678943
C -0.916248 0.086729 -0.881012
C -1.783085 0.604237 0.316781
C -3.177410 1.077068 -0.050304
C 0.346485 -0.663522 -0.326835
C -0.198641 -1.825491 0.522158
C -0.781402 -1.061991 1.717424
O -1.985860 -0.406900 1.309574
C 0.345509 -0.088038 2.080889
C -5.336815 1.980689 0.976240
C -0.521039 1.265853 -1.775868
O 0.896756 -2.629006 0.961104
C 1.039972 0.239676 0.745467
O 2.448644 -0.071295 0.866982
C 3.329495 0.713425 0.190879
C 4.695185 0.223537 0.426369
O 2.992377 1.671299 -0.485440
C 1.265652 -1.120246 -1.456232
C 5.805406 0.668305 -0.183160
C 5.953813 1.722675 -1.221813
O -2.032636 -2.096600 -1.204139
C -1.166012 -2.731141 -0.227299
O -3.194902 2.432671 1.930092
O -2.413673 -0.517702 -2.674847
H -1.069022 -1.725151 2.540630
H -1.239380 1.454719 0.756019
H -3.820751 0.230273 -0.319775
H -3.155924 1.734648 -0.929603
H 1.052007 -0.583849 2.750512
H -0.036455 0.803348 2.587027
H -5.657551 2.188460 -0.050589
H -5.759777 1.004188 1.245719
H -5.714076 2.742572 1.660378
H 0.178663 1.926040 -1.258291
H -0.047609 0.909773 -2.693028
H -1.394533 1.850990 -2.067714
H 0.575360 -3.225002 1.652668
H 0.962047 1.296357 0.485919

H 4.778633 -0.580389 1.152261
H 2.048646 -1.774210 -1.067881
H 0.702671 -1.678100 -2.213972
H 1.741598 -0.270262 -1.949874
H 6.736068 0.184894 0.116586
H 6.554427 2.548417 -0.816870
H 6.527902 1.321532 -2.066202
H 5.001308 2.114866 -1.573050
H -0.578529 -3.471688 -0.780639
H -1.831492 -3.251489 0.469468

2a-c12 , delta G = 1.0364 kcal/mol, population = 14.25 %

C -4.014646 1.673244 0.978266
C -1.809022 -0.916242 -1.707625
C -0.954715 0.107447 -0.966498
C -1.833606 0.676339 0.195130
C -3.239970 1.110226 -0.200644
C 0.320958 -0.584469 -0.371533
C -0.194612 -1.707257 0.555039
C -0.806925 -0.881727 1.696059
O -2.025704 -0.279992 1.240547
C 0.283485 0.153403 1.995511
C -3.344210 2.727395 1.816181
C -0.589342 1.240755 -1.931061
O 0.841846 -2.546375 1.069716
C 1.008093 0.378745 0.651844
O 2.394773 -0.010583 0.834938
C 3.356175 0.684669 0.157934
C 4.670411 0.099671 0.448979
O 3.102001 1.641573 -0.550765
C 1.244364 -1.084762 -1.480158
C 5.835519 0.474122 -0.103549
C 6.104162 1.530321 -1.114520
O -1.994948 -2.128295 -1.159990
C -1.144981 -2.679857 -0.124742
O -5.149801 1.290722 1.212180
O -2.414457 -0.659412 -2.729059
H -1.079235 -1.500115 2.555904
H -1.291225 1.553935 0.580636
H -3.810512 0.275418 -0.618514
H -3.180759 1.904420 -0.955505
H 0.984277 -0.249998 2.732021
H -0.131040 1.075866 2.411926
H -2.580832 2.250082 2.443813
H -2.829092 3.460928 1.186529
H -4.075027 3.219293 2.460353
H 0.057861 1.972751 -1.441334
H -0.070116 0.848612 -2.807476
H -1.483051 1.756141 -2.286326
H 1.611559 -2.008395 1.304047
H 0.990095 1.417995 0.320965
H 4.665097 -0.712534 1.170282
H 2.024287 -1.732022 -1.072579
H 0.682302 -1.666931 -2.219820
H 1.727918 -0.255729 -2.001478
H 6.715741 -0.075522 0.232048
H 6.679502 1.100259 -1.943756
H 5.200037 2.001427 -1.495104
H 6.750775 2.298797 -0.669577
H -0.549536 -3.461762 -0.607076
H -1.818177 -3.142143 0.603559

2a-c18 , delta G = 1.8397 kcal/mol, population = 3.67 %

C -3.374875 2.299404 0.435333
C -2.017918 -0.922754 -1.430339
C -1.087411 -0.036895 -0.605345
C -1.826464 0.278406 0.742819
C -3.252306 0.795361 0.601054
C 0.259255 -0.783690 -0.296178
C -0.144554 -2.077471 0.433764
C -0.629698 -1.513964 1.774684
O -1.898224 -0.875452 1.587749
C 0.492645 -0.539890 2.154673
C -4.608704 2.775234 -0.273484
C -0.854692 1.265531 -1.377836
O 1.022771 -2.871345 0.649199
C 1.023402 0.006015 0.815186
O 2.449768 -0.230827 0.746550
C 3.219957 0.719410 0.151069
C 4.626599 0.293908 0.186565
O 2.767420 1.748766 -0.322043
C 1.071744 -1.029411 -1.563902
C 5.674305 0.995793 -0.273928
C 5.705057 2.338404 -0.913045
O -2.146487 -2.218739 -1.103469
C -1.146888 -2.929714 -0.329912

O -2.540203 3.067631 0.885531
O -2.735327 -0.491156 -2.312146
H -0.801514 -2.296497 2.521822
H -1.245091 1.066832 1.242921
H -3.771844 0.579779 1.547218
H -3.822023 0.279470 -0.178413
H 1.286004 -1.089074 2.666931
H 0.139873 0.250769 2.823299
H -4.716281 3.857946 -0.192153
H -4.533289 2.487577 -1.330509
H -5.494351 2.266660 0.124458
H -1.797641 1.784968 -1.557476
H -0.191394 1.935657 -0.825965
H -0.411864 1.058312 -2.354125
H 0.797825 -3.571841 1.278164
H 0.868989 1.081051 0.711510
H 4.801220 -0.680650 0.633663
H 1.478097 -0.096736 -1.962090
H 1.905233 -1.703279 -1.356993
H 0.450388 -1.486424 -2.343364
H 6.648775 0.518487 -0.162981
H 6.367967 2.997259 -0.337234
H 6.159317 2.255092 -1.909042
H 4.721095 2.794807 -0.998778
H -0.594098 -3.549367 -1.044450
H -1.702264 -3.581600 0.352514

Table S3. Geometry data of conformers of structure **3a**, **3b**, **3c**, **3d**, **3e**, **3f**, **3g** and **3h**

3a

3a-c9 , delta G = 0.0000 kcal/mol, population = 48.69 %

C -3.206388 -2.403070 0.651895
C -4.103355 -1.696219 -0.373900
C -3.466755 -0.302746 -0.572371
C -2.269954 -0.220434 0.445426
C -0.976475 -0.826342 -0.239876
C 0.128931 -1.042534 0.821440
C 1.325878 -1.876087 0.368584
C 0.888848 -3.219823 -0.211085
C -0.141138 -3.009270 -1.321236
C -1.348162 -2.193610 -0.870307
C -2.754548 -1.226772 1.485026
C -2.441614 -1.278100 2.906584
C -2.042191 1.179926 0.999790
C -0.484852 0.126631 -1.344107
C 2.071975 -4.058088 -0.687496
C 3.338589 -0.695809 -0.172205
C 4.108257 -0.155694 -1.379071
C 5.394218 0.509386 -0.898194
C 5.115138 1.533629 0.204653
C 4.322008 0.868060 1.326925
C 3.914270 1.836994 2.420491
C -4.469997 1.849952 -0.959207
C -5.539258 2.725688 -0.454735
C -5.768946 3.989704 -0.841945
C -5.036667 4.816954 -1.838737
O -2.066394 -3.034580 0.056391
H -1.982641 -2.013263 -1.754090
O -3.721114 -0.777877 2.458453
H -3.719994 -3.175761 1.230902
O 2.116850 -1.165990 -0.623471
O -4.477380 0.687205 -0.256389
O -3.687877 2.082496 -1.865202
O 3.103609 0.342148 0.783533
O 3.180616 1.183996 3.456340
O 6.336055 2.022446 0.755546
O 6.118525 1.107258 -1.972650
O 3.363639 0.832807 -2.086163

H 0.394597 -3.750951 0.614858
H 1.969496 -2.055236 1.243067
H 0.491692 -0.077093 1.187173
H -0.291494 -1.579669 1.678058
H 0.333655 -2.512497 -2.175865
H -0.502004 -3.979408 -1.684767
H -5.102286 -1.577707 0.055971
H -4.196916 -2.258904 -1.307182
H 2.800091 -4.208496 0.118161
H 2.585325 -3.569884 -1.523026
H 1.733601 -5.043080 -1.026577
H 0.026335 0.989637 -0.907871
H 0.229605 -0.371628 -2.001017
H -1.308690 0.497677 -1.960510
H -1.787462 -0.522254 3.342263
H -2.476074 -2.236331 3.426798
H -3.144763 -0.129662 -1.599732
H -1.847677 1.895454 0.197185
H -2.922242 1.515725 1.553104
H -1.189449 1.199718 1.683965
H -6.173560 2.284003 0.308605
H -6.607826 4.489570 -0.355875
H -5.714582 5.077675 -2.662534
H -4.744664 5.768646 -1.377356
H -4.158960 4.316962 -2.243559
H 3.930598 -1.497616 0.315808
H 4.921635 0.048996 1.764380
H 4.514292 2.361241 -0.209675
H 6.053297 -0.264599 -0.480993
H 4.357942 -1.002511 -2.039475
H 4.808719 2.263741 2.882076
H 3.326134 2.654417 1.972708
H 2.421929 0.758635 3.034983
H 6.887975 2.296382 0.009981
H 5.485139 1.639555 -2.475961
H 2.539524 0.424720 -2.382216

3a-c17 , delta G = 0.5515 kcal/mol, population = 19.17 %

C -3.221928 -2.452475 0.490061
C -4.056646 -1.597954 -0.474411
C -3.342076 -0.229377 -0.520332
C -2.166921 -0.314797 0.519681
C -0.892623 -0.921714 -0.202941
C 0.177850 -1.300164 0.848700
C 1.343483 -2.146379 0.334145
C 0.838401 -3.407687 -0.364038
C -0.146320 -3.032564 -1.473274
C -1.320048 -2.198558 -0.971477
C -2.725636 -1.391987 1.445008
C -2.442725 -1.601925 2.858047
C -1.877884 1.010475 1.212729
C -0.326640 0.109590 -1.194374
C 1.975015 -4.282256 -0.886515
C 3.296651 -0.810871 -0.059547
C 4.215234 -0.344567 -1.186174
C 5.405123 0.401615 -0.573614
C 4.934519 1.532101 0.341735
C 3.945166 0.996065 1.374942
C 3.307435 2.079750 2.228421
C -4.239638 1.994446 -0.729847
C -5.272855 2.872722 -0.158704
C -5.515469 4.142589 -0.517870
C -4.842377 4.976291 -1.549992
O -2.105555 -3.085423 -0.146492
H -1.922808 -1.899263 -1.845308
O -3.686032 -0.991537 2.445862
H -3.790315 -3.248316 0.979690
O 2.189926 -1.429997 -0.601357
O -4.303478 0.780490 -0.122946
O -3.438658 2.265359 -1.608176
O 2.865770 0.336428 0.695870
O 2.573359 3.025201 1.450511
O 6.031878 2.107883 1.047107
O 6.275278 0.907612 -1.585378
O 3.532960 0.486168 -2.127109
H 0.288031 -3.970300 0.402993
H 1.967588 -2.436846 1.195374
H 0.574074 -0.398947 1.323179
H -0.284859 -1.903611 1.636956
H 0.379438 -2.480518 -2.261376
H -0.548009 -3.940862 -1.939392
H -5.056942 -1.467798 -0.050992
H -4.159287 -2.058153 -1.461290
H 2.684010 -4.526304 -0.086643
H 2.525407 -3.773335 -1.684614
H 1.582145 -5.221751 -1.289831
H 0.162697 0.924643 -0.652163

H 0.427511 -0.337959 -1.842465
H -1.107989 0.544683 -1.824282
H -1.757897 -0.929120 3.375382
H -2.536343 -2.603827 3.279125
H -2.986758 0.026928 -1.518696
H -1.674253 1.799961 0.485057
H -2.735336 1.315286 1.817229
H -1.011867 0.928913 1.875532
H -5.874730 2.422497 0.625586
H -6.323202 4.640698 0.020139
H -4.448949 5.887509 -1.081032
H -4.037659 4.453371 -2.062898
H -5.585860 5.314209 -2.283689
H 3.850675 -1.487204 0.622765
H 4.468198 0.284443 2.039501
H 4.424799 2.300331 -0.264241
H 6.003599 -0.302278 0.022195
H 4.578942 -1.216040 -1.744783
H 2.660045 1.600711 2.978619
H 4.091264 2.636763 2.749706
H 1.876751 2.537516 0.991328
H 6.697188 2.340735 0.385201
H 5.708033 1.350510 -2.234922
H 2.914011 1.044944 -1.633668

3a-c20 , delta G = 0.9499 kcal/mol, population = 9.78 %

C -3.278366 -2.113922 0.511817
C -4.081329 -1.435320 -0.606992
C -3.359881 -0.093740 -0.861308
C -2.217005 -0.000354 0.214257
C -0.923212 -0.717227 -0.354293
C 0.112957 -0.917172 0.778431
C 1.280825 -1.847665 0.450400
C 0.793514 -3.201733 -0.061094
C -0.154346 -3.007177 -1.245604
C -1.336079 -2.102296 -0.915235
C -2.810754 -0.905503 1.288981
C -2.581975 -0.867236 2.726440
C -1.944124 1.423460 0.682624
C -0.323505 0.138350 -1.483446
C 1.945047 -4.143728 -0.401990
C 3.354910 -0.753305 -0.050082
C 4.257683 -0.434391 -1.243547
C 5.512499 0.277395 -0.749809
C 5.160798 1.477720 0.131069
C 4.241048 1.025467 1.263357
C 3.760654 2.176251 2.126916
C -4.231012 2.071711 -1.432576
C -5.227812 3.109124 -1.123229
C -6.002276 3.244836 -0.035125
C -6.106858 2.401806 1.188755
O -2.144801 -2.841046 0.024436
H -1.915406 -1.949696 -1.840964
O -3.808085 -0.341717 2.168009
H -3.862887 -2.817987 1.110786
O 2.168285 -1.268277 -0.543348
O -4.334115 0.965424 -0.664984
O -3.409021 2.201969 -2.326641
O 3.064257 0.430635 0.698965
O 2.909228 1.728013 3.181319
O 6.337514 2.033968 0.713889
O 6.360442 0.671536 -1.828022
O 3.620601 0.434413 -2.176830
H 0.215035 -3.638277 0.765068
H 1.866678 -2.001442 1.369815
H 0.514751 0.051023 1.091714
H -0.377814 -1.363818 1.649525
H 0.395809 -2.588357 -2.096541
H -0.548084 -3.977410 -1.572826
H -5.092441 -1.234650 -0.240590
H -4.159666 -2.055375 -1.504644
H 2.533723 -3.754438 -1.239441
H 1.563225 -5.130459 -0.685166
H 2.616312 -4.272303 0.455069
H 0.195183 1.007040 -1.067806
H 0.410330 -0.426024 -2.060984
H -1.090620 0.500601 -2.173845
H -1.916934 -0.112175 3.146705
H -2.695522 -1.781544 3.310197
H -2.975995 -0.006472 -1.877838
H -1.091392 1.458847 1.366033
H -1.727130 2.081012 -0.162582
H -2.813957 1.820797 1.211654
H -5.279803 3.869956 -1.897641
H -6.656403 4.118083 -0.039425
H -5.295047 1.685382 1.298846
H -6.155844 3.047690 2.072806

H -7.054659 1.845629 1.165430
H 3.867838 -1.476841 0.617014
H 4.769755 0.285872 1.892246
H 4.631614 2.232210 -0.475835
H 6.099038 -0.426824 -0.143595
H 4.540763 -1.384695 -1.725653
H 4.619813 2.663389 2.595667
H 3.246304 2.911522 1.487137
H 2.175634 1.254868 2.766698
H 6.967745 2.168335 -0.007378
H 5.798099 1.117661 -2.478085
H 2.817429 -0.004723 -2.485661

3a-c26 , delta G = 1.0442 kcal/mol, population = 8.34 %
C -3.310775 -2.150518 0.350697
C -4.045022 -1.359739 -0.741574
C -3.274862 -0.026405 -0.859050
C -2.170928 -0.058898 0.259777
C -0.880865 -0.774012 -0.319380
C 0.104382 -1.094001 0.829757
C 1.263606 -2.021652 0.463650
C 0.755286 -3.323082 -0.154155
C -0.147562 -3.016497 -1.350489
C -1.313695 -2.098744 -0.998262
C -2.834079 -1.023790 1.237515
C -2.656426 -1.106669 2.680394
C -1.866000 1.311469 0.851530
C -0.215591 0.145459 -1.358378
C 1.889572 -4.273459 -0.528421
C 3.324729 -0.843324 0.075139
C 4.280509 -0.524609 -1.076483
C 5.480537 0.244878 -0.538332
C 5.034853 1.461797 0.271211
C 4.067076 1.025929 1.371166
C 3.476749 2.185893 2.155596
C -4.053104 2.210256 -1.266838
C -5.025477 3.251535 -0.898274
C -5.829540 3.320124 0.174591
C -6.000875 2.379263 1.316979
O -2.182484 -2.877855 -0.149024
H -1.849767 -1.858744 -1.931714
O -3.843533 -0.495495 2.124933
H -3.941919 -2.877065 0.870203
O 2.186818 -1.409190 -0.473626
O -4.223963 1.043891 -0.608465
O -3.192649 2.389957 -2.115013
O 2.952484 0.342495 0.782914
O 2.747038 3.088469 1.325146
O 6.153881 2.080353 0.904356
O 6.377654 0.629296 -1.580692
O 3.665400 0.295183 -2.067277
H 0.139702 -3.799114 0.621769
H 1.826118 -2.253516 1.382128
H 0.508879 -0.167552 1.246085
H -0.430789 -1.608049 1.635379
H 0.444182 -2.557378 -2.151166
H -0.558061 -3.948605 -1.758113
H -5.065250 -1.156811 -0.402746
H -4.101091 -1.904389 -1.688358
H 1.488952 -5.231225 -0.877469
H 2.539271 -4.470113 0.332430
H 2.505325 -3.850597 -1.329120
H 0.351277 0.937462 -0.860099
H 0.489921 -0.405416 -1.982081
H -0.950015 0.619330 -2.016234
H -1.981911 -0.410814 3.180249
H -2.820753 -2.059173 3.185769
H -2.851907 0.131314 -1.851411
H -2.747656 1.708915 1.360383
H -1.056165 1.253969 1.583941
H -1.569091 2.020933 0.075357
H -5.027101 4.078256 -1.603887
H -6.453644 4.213496 0.227976
H -6.968938 1.867337 1.222554
H -5.220994 1.623206 1.384099
H -6.049029 2.947997 2.252680
H 3.821201 -1.535918 0.786416
H 4.598871 0.358275 2.074184
H 4.518739 2.174797 -0.390940
H 6.053690 -0.417580 0.125218
H 4.619723 -1.477848 -1.514526
H 2.835527 1.778058 2.952339
H 4.284433 2.759967 2.618449
H 2.079115 2.567937 0.858672
H 6.813993 2.232926 0.214481
H 5.841923 1.048941 -2.269639
H 2.900579 -0.184582 -2.410797

3a-c1 , delta G = 1.3835 kcal/mol, population = 4.70 %
C -3.267830 -2.462231 0.591221
C -4.156288 -1.752198 -0.439963
C -3.526595 -0.352839 -0.617903
C -2.343964 -0.271539 0.416269
C -1.037548 -0.863026 -0.256742
C 0.054771 -1.082122 0.817556
C 1.263681 -1.901346 0.372001
C 0.846035 -3.241922 -0.229003
C -0.173254 -3.028912 -1.348458
C -1.391687 -2.226475 -0.904586
C -2.835314 -1.290186 1.440495
C -2.540272 -1.351291 2.865303
C -2.132332 1.125442 0.985429
C -0.538011 0.102914 -1.346603
C 2.042322 -4.063441 -0.701630
C 3.287021 -0.721603 -0.140240
C 4.043735 -0.141848 -1.336927
C 5.334143 0.510335 -0.851304
C 5.052739 1.487644 0.289132
C 4.283231 0.781901 1.406646
C 3.894753 1.715845 2.549981
C -4.534458 1.799516 -0.993837
C -5.615633 2.664541 -0.496377
C -5.817078 3.945481 -0.841107
C -5.033683 4.803356 -1.771123
O -2.115728 -3.080807 0.005195
H -2.016356 -2.042429 -1.794616
O -3.817640 -0.856293 2.405119
H -3.783689 -3.243460 1.156573
O 2.059396 -1.168394 -0.600006
O -4.548145 0.627604 -0.306808
O -3.740421 2.047044 -1.885366
O 3.061613 0.276065 0.861867
O 5.008465 2.069948 3.364860
O 6.277506 1.985771 0.836376
O 6.037470 1.157138 -1.911449
O 3.289223 0.867778 -2.002239
H 0.347626 -3.787175 0.585209
H 1.897213 -2.084580 1.252442
H 0.405677 -0.118832 1.199331
H -0.373049 -1.631762 1.662428
H 0.307318 -2.520752 -2.193134
H -0.522335 -3.998390 -1.725069
H -5.161239 -1.644055 -0.021477
H -4.234498 -2.306856 -1.379423
H 2.760533 -4.220495 0.111616
H 2.563062 -3.558432 -1.522590
H 1.716926 -5.045849 -1.060404
H -0.028805 0.960318 -0.897109
H 0.178769 -0.388949 -2.006192
H -1.357695 0.481828 -1.963758
H -1.896590 -0.594770 3.315125
H -2.574546 -2.313921 3.377375
H -3.192317 -0.168669 -1.639453
H -1.930630 1.848575 0.191462
H -3.022084 1.451600 1.528982
H -1.289486 1.144244 1.681595
H -6.281591 2.200660 0.225756
H -6.669807 4.435504 -0.369431
H -4.128354 4.320356 -2.133982
H -5.661128 5.076158 -2.630462
H -4.781164 5.746549 -1.271257
H 3.880750 -1.543661 0.310612
H 4.898142 -0.043808 1.809868
H 4.433096 2.319316 -0.085503
H 6.005441 -0.272941 -0.473391
H 4.284893 -0.967300 -2.027064
H 3.396126 2.607555 2.135687
H 3.179548 1.194719 3.195633
H 5.688616 2.423841 2.773080
H 6.779730 2.373078 0.106294
H 5.390732 1.696882 -2.389174
H 2.441244 0.480262 -2.256401

3a-c15 , delta G = 1.4748 kcal/mol, population = 4.03 %
C -3.205872 -2.416038 0.637904
C -4.092103 -1.683817 -0.379562
C -3.429351 -0.301363 -0.568814
C -2.228380 -0.250265 0.445439
C -0.948907 -0.876914 -0.248170
C 0.153551 -1.121403 0.808889
C 1.336292 -1.971411 0.347281
C 0.871795 -3.302294 -0.241106
C -0.157402 -3.065809 -1.347195

C -1.348376 -2.231164 -0.888348
C -2.728976 -1.255025 1.478790
C -2.411528 -1.322896 2.898381
C -1.970626 1.140735 1.010212
C -0.440558 0.075751 -1.345048
C 2.035585 -4.162699 -0.725838
C 3.314118 -0.715099 -0.150242
C 4.146113 -0.246062 -1.344773
C 5.363806 0.516378 -0.834907
C 4.966737 1.615022 0.154034
C 4.109000 1.016012 1.270884
C 3.601824 2.072537 2.236397
C -4.390738 1.875061 -0.929795
C -5.437867 2.769232 -0.411201
C -5.595893 4.061201 -0.736421
C -4.787199 4.903091 -1.659579
O -2.080897 -3.065550 0.033656
H -1.981384 -2.032783 -1.769507
O -3.683829 -0.795843 2.459187
H -3.733046 -3.182831 1.212602
O 2.150263 -1.284742 -0.641413
O -4.420295 0.705017 -0.240726
O -3.610637 2.101191 -1.839158
O 2.965633 0.384266 0.691067
O 2.884547 1.515017 3.339693
O 6.130231 2.192270 0.744937
O 6.141275 1.056394 -1.903614
O 3.416319 0.642079 -2.187894
H 0.368802 -3.828044 0.582892
H 1.976208 -2.170996 1.221099
H 0.530844 -0.167852 1.187481
H -0.274050 -1.663877 1.658745
H 0.323770 -2.572129 -2.200085
H -0.536446 -4.027115 -1.715882
H -5.087011 -1.549213 0.054894
H -4.199620 -2.236982 -1.317005
H 2.770249 -4.319722 0.072647
H 2.547633 -3.688380 -1.569758
H 1.677012 -5.144182 -1.054364
H 0.265284 -0.429811 -2.005639
H -1.257854 0.466643 -1.958069
H 0.087196 0.925039 -0.901768
H -1.741610 -0.582668 3.336809
H -2.461848 -2.284199 3.411645
H -3.107418 -0.125477 -1.595782
H -2.840997 1.488340 1.571597
H -1.113572 1.138522 1.689340
H -1.767027 1.859410 0.212674
H -6.112991 2.319204 0.311218
H -6.427189 4.574789 -0.251781
H -3.890681 4.400000 -2.017347
H -5.401406 5.189320 -2.524238
H -4.517750 5.840045 -1.157268
H 3.901313 -1.446199 0.444660
H 4.713709 0.270965 1.823603
H 4.374872 2.383195 -0.372849
H 6.022131 -0.188464 -0.308086
H 4.478699 -1.133562 -1.908295
H 4.449767 2.680958 2.577180
H 2.896410 2.724041 1.707845
H 3.518421 1.051309 3.900632
H 6.727596 2.418099 0.018491
H 5.525754 1.516246 -2.493160
H 2.621475 0.179213 -2.483700

3a-c31 , delta G = 1.7056 kcal/mol, population = 2.73 %

C -3.304529 -2.163409 0.310335
C -4.019501 -1.332438 -0.764984
C -3.227455 -0.009117 -0.843065
C -2.128672 -0.090069 0.277961
C -0.848587 -0.813403 -0.313901
C 0.121761 -1.182076 0.833093
C 1.272739 -2.114340 0.449919
C 0.744991 -3.392162 -0.200887
C -0.145452 -3.040598 -1.394506
C -1.299473 -2.113553 -1.027493
C -2.812783 -1.068200 1.227946
C -2.642972 -1.191352 2.668793
C -1.802828 1.259509 0.905210
C -0.160577 0.119030 -1.325053
C 1.862258 -4.355414 -0.593491
C 3.273988 -0.833698 0.088802
C 4.331885 -0.580651 -0.982660
C 5.473868 0.233334 -0.366442
C 4.948229 1.509421 0.290884
C 3.831289 1.171144 1.276474
C 3.148780 2.392582 1.869204

C -3.969792 2.250236 -1.191293
C -4.919791 3.299941 -0.789020
C -5.710588 3.359658 0.294176
C -5.887939 2.396798 1.416953
O -2.186925 -2.898028 -0.202349
H -1.825461 -1.841971 -1.958059
O -3.816900 -0.545469 2.124983
H -3.951156 -2.891375 0.808456
O 2.213626 -1.512056 -0.473961
O -4.159585 1.069703 -0.564278
O -3.109185 2.437557 -2.037673
O 2.807364 0.432670 0.591854
O 2.547593 3.219771 0.873460
O 5.981942 2.173484 1.014289
O 6.471288 0.546085 -1.338234
O 3.792892 0.092629 -2.121685
H 0.117067 -3.874559 0.561001
H 1.826236 -2.377205 1.366781
H 0.530109 -0.276258 1.287542
H -0.428745 -1.718384 1.613540
H 0.458663 -2.569204 -2.178441
H -0.567298 -3.955574 -1.828579
H -5.038500 -1.122195 -0.426936
H -4.077838 -1.851124 -1.726036
H 1.443543 -5.299922 -0.957257
H 2.509910 -4.578017 0.262571
H 2.483385 -3.930265 -1.388458
H 0.379032 0.912700 -0.799995
H 0.574684 -0.417714 -1.926175
H -0.879125 0.589716 -2.002680
H -1.958643 -0.520790 3.189565
H -2.826046 -2.153512 3.148869
H -2.798753 0.169853 -1.829235
H -1.502921 1.986113 0.146221
H -2.676504 1.653780 1.430070
H -0.988922 1.173013 1.630420
H -4.912055 4.143122 -1.474887
H -6.315381 4.264142 0.375758
H -5.134984 1.611738 1.445382
H -5.891101 2.943331 2.367224
H -6.876810 1.924023 1.335819
H 3.720700 -1.399014 0.931774
H 4.248793 0.564961 2.101062
H 4.538362 2.175759 -0.487320
H 5.974190 -0.373250 0.401941
H 4.720610 -1.541183 -1.343355
H 2.401408 2.055318 2.603555
H 3.892365 3.005954 2.386223
H 1.884427 2.683546 0.418988
H 6.727415 2.273671 0.406542
H 5.998329 0.885447 -2.113442
H 3.147068 0.740963 -1.803634

3a-c30 , delta G = 1.7419 kcal/mol, population = 2.56 %

C -3.240456 -2.459962 0.413354
C -4.043529 -1.556285 -0.532930
C -3.297226 -0.203875 -0.526509
C -2.139326 -0.348506 0.526058
C -0.869403 -0.961967 -0.197391
C 0.172798 -1.404291 0.857504
C 1.330575 -2.255819 0.333453
C 0.810486 -3.478962 -0.419670
C -0.151373 -3.044952 -1.527727
C -1.313995 -2.201620 -1.014683
C -2.734921 -1.441165 1.408543
C -2.477140 -1.701002 2.818049
C -1.827108 0.948972 1.260680
C -0.263852 0.089766 -1.142585
C 1.934630 -4.360460 -0.957692
C 3.273408 -0.859782 0.046161
C 4.262879 -0.478874 -1.060248
C 5.347980 0.426625 -0.487475
C 4.732727 1.603679 0.267039
C 3.781319 1.074749 1.338995
C 3.101689 2.167283 2.150192
C -4.149391 2.041773 -0.688356
C -5.166761 2.927504 -0.100113
C -5.365958 4.216879 -0.413208
C -4.647934 5.070102 -1.398168
O -2.130855 -3.097716 -0.230890
H -1.896564 -1.859765 -1.886504
O -3.701722 -1.052396 2.407908
H -3.832475 -3.258322 0.869922
O 2.222018 -1.534545 -0.554817
O -4.239679 0.815932 -0.109659
O -3.342440 2.316120 -1.560058
O 2.761064 0.298881 0.706542

O 2.381044 3.108392 1.353669
O 5.742275 2.377296 0.913992
O 6.248428 0.883745 -1.496972
O 3.628886 0.242576 -2.113622
H 0.237201 -4.056018 0.319220
H 1.929523 -2.592854 1.195748
H 0.576965 -0.531301 1.374177
H -0.317779 -2.030156 1.610660
H 0.396906 -2.479881 -2.290972
H -0.566078 -3.928481 -2.028938
H -5.045669 -1.416412 -0.116969
H -4.144815 -1.982543 -1.535148
H 2.632988 -4.638603 -0.159741
H 2.501229 -3.841292 -1.737645
H 1.527366 -5.281141 -1.389180
H -1.028115 0.582564 -1.750992
H 0.264101 0.856870 -0.569688
H 0.466904 -0.361378 -1.814919
H -1.786190 -1.058926 3.365397
H -2.597539 -2.713497 3.205666
H -2.921427 0.074771 -1.511234
H -1.617007 1.758289 0.556903
H -2.675588 1.245709 1.881807
H -0.954866 0.834864 1.909961
H -5.793666 2.466006 0.657582
H -6.168623 4.717614 0.129936
H -4.255543 5.960178 -0.889769
H -3.836645 4.548794 -1.902311
H -5.362277 5.441751 -2.144609
H 3.787173 -1.499091 0.794675
H 4.356124 0.445506 2.045785
H 4.160082 2.223078 -0.443134
H 5.955500 -0.154118 0.220585
H 4.721739 -1.405126 -1.444305
H 2.366737 1.694590 2.811157
H 3.856145 2.666000 2.772899
H 3.016863 3.719692 0.962738
H 6.419462 2.562462 0.248548
H 5.705800 1.213379 -2.228277
H 2.909934 -0.306958 -2.452369

3b

3b-c13 , delta G = 0.0000 kcal/mol, population = 52.28 %

C -2.921874 -2.838389 -0.172129
C -3.971983 -2.001773 -0.915945
C -3.625830 -0.533902 -0.582579
C -2.437818 -0.587376 0.446613
C -1.071847 -0.645638 -0.355816
C 0.092331 -0.992520 0.604462
C 1.409969 -1.392041 -0.060657
C 1.212671 -2.505904 -1.083738
C 0.142125 -2.102421 -2.098521
C -1.186805 -1.746819 -1.442210
C -2.682679 -1.971648 1.041429
C -2.319892 -2.464193 2.363267
C -2.476214 0.538723 1.471709
C -0.814190 0.712519 -1.030555
C 2.518164 -2.913940 -1.761601
C 2.856836 0.511546 0.062342
C 3.123055 1.822311 -0.680650
C 4.166374 2.634644 0.079796
C 5.408723 1.797858 0.392994
C 4.990680 0.519034 1.115207
C 6.155282 -0.415749 1.382163
C -5.046427 1.376921 -0.230545
C -6.254933 1.789284 0.500318
C -6.765279 3.029509 0.542302
C -6.272596 4.274388 -0.106520
O -1.699518 -2.983629 -0.904471
H -1.870586 -1.389271 -2.230203
O -3.686496 -2.095810 2.070768
H -3.256609 -3.850110 0.073829
O 2.036261 -0.268694 -0.736329
O -4.800541 0.064435 0.021001
O -4.352174 2.061949 -0.962291
O 4.075936 -0.204441 0.281604
O 4.522105 3.829454 -0.615362
O 6.296458 2.518040 1.245642
O 3.636963 1.592591 -1.990090
O 5.738925 -1.596719 2.067026
H 0.830721 -3.364783 -0.514466
H 2.103083 -1.744749 0.717211
H 0.274762 -0.151393 1.283412
H -0.186109 -1.845032 1.232134
H 0.492911 -1.248811 -2.690695
H -0.034515 -2.924045 -2.803647
H -4.959348 -2.238306 -0.508708

H -3.986786 -2.203357 -1.990822
H 3.282323 -3.169195 -1.018415
H 2.910461 -2.101000 -2.380951
H 2.360147 -3.787639 -2.402977
H -1.698813 1.084349 -1.555454
H -0.530755 1.459328 -0.282724
H 0.005113 0.649134 -1.748180
H -1.813561 -1.801991 3.066284
H -2.149579 -3.531947 2.507636
H -3.375956 0.051595 -1.468132
H -3.375619 0.457127 2.086503
H -1.610341 0.496810 2.138462
H -2.482109 1.517013 0.985032
H -6.749521 0.996188 1.053928
H -7.669674 3.149531 1.140315
H -5.347135 4.129161 -0.660115
H -7.045044 4.660352 -0.784790
H -6.130221 5.049611 0.657237
H 2.393659 0.735012 1.044518
H 2.179243 2.390604 -0.727538
H 4.501143 0.780397 2.071348
H 5.910573 1.522823 -0.550550
H 3.727876 2.958315 1.034061
H 4.717209 3.574933 -1.529061
H 6.424950 3.388129 0.843231
H 3.002234 1.035314 -2.459515
H 6.884439 0.085624 2.024126
H 6.641808 -0.663121 0.424817
H 5.065197 -2.016591 1.516149

3b-c24 , delta G = 0.4680 kcal/mol, population = 23.71 %

C -2.963675 -2.836353 -0.073933
C -4.014475 -2.002844 -0.820411
C -3.652161 -0.532903 -0.514576
C -2.455110 -0.580078 0.503884
C -1.097107 -0.663915 -0.310800
C 0.072419 -1.009650 0.643260
C 1.380771 -1.429723 -0.028532
C 1.163392 -2.554897 -1.035174
C 0.085657 -2.156246 -2.044287
C -1.233023 -1.779696 -1.379177
C -2.705463 -1.952323 1.123238
C -2.335200 -2.426564 2.449811
C -2.474958 0.563698 1.509600
C -0.831408 0.681482 -1.006885
C 2.458722 -2.980992 -1.721836
C 2.821781 0.467601 0.061717
C 3.134667 1.773744 -0.662091
C 4.095147 2.594976 0.205067
C 5.333343 1.780949 0.583061
C 4.920635 0.448192 1.205619
C 6.087896 -0.484251 1.481656
C -5.062547 1.391446 -0.195915
C -6.258620 1.828150 0.541413
C -6.805647 3.052781 0.501890
C -6.377825 4.252965 -0.266198
O -1.750748 -3.003865 -0.816807
H -1.922324 -1.428589 -2.165115
O -3.701128 -2.052423 2.163638
H -3.304411 -3.841093 0.191776
O 2.011591 -0.323049 -0.727795
O -4.814871 0.086612 0.091047
O -4.377565 2.053445 -0.956912
O 4.052678 -0.244076 0.294096
O 4.473914 3.806998 -0.445924
O 6.129301 2.479596 1.538331
O 3.691398 1.551702 -1.958708
O 6.819747 -0.809855 0.300487
H 0.781569 -3.403823 -0.451036
H 2.076897 -1.780159 0.747850
H 0.266789 -0.162496 1.311336
H -0.208103 -1.852246 1.283221
H 0.437358 -1.314472 -2.652327
H -0.106064 -2.986865 -2.734821
H -4.999669 -2.224122 -0.399504
H -4.041819 -2.221940 -1.891631
H 3.229281 -3.231208 -0.983514
H 2.847077 -2.178874 -2.357532
H 2.287925 -3.862415 -2.349241
H -1.712562 1.048064 -1.541387
H -0.548665 1.438980 -0.269490
H -0.008793 0.603676 -1.718979
H -1.817327 -1.756833 3.137236
H -2.171253 -3.493120 2.609703
H -3.405422 0.035746 -1.411804
H -3.364258 0.495047 2.140606
H -1.598360 0.532373 2.162769

H -2.487413 1.533510 1.006124
H -6.714601 1.067107 1.168333
H -7.693572 3.194864 1.119446
H -7.198414 4.575907 -0.920185
H -6.202907 5.085146 0.427965
H -5.484302 4.081226 -0.862841
H 2.360954 0.691854 1.043783
H 2.206535 2.338846 -0.813955
H 4.392877 0.642501 2.157156
H 5.923454 1.574602 -0.326208
H 3.578686 2.891033 1.129405
H 4.721222 3.563286 -1.351037
H 6.287687 3.362564 1.177421
H 4.282246 0.786521 -1.898639
H 5.703510 -1.392628 1.969915
H 6.787210 0.007485 2.163883
H 6.207380 -1.251976 -0.301932

3b-c3 , delta G = 1.0137 kcal/mol, population = 9.43 %
C -2.995034 -2.865507 -0.097750
C -4.035538 -2.022716 -0.848231
C -3.656290 -0.556041 -0.548020
C -2.461389 -0.613142 0.472443
C -1.103327 -0.715535 -0.340282
C 0.061428 -1.069538 0.616280
C 1.366365 -1.504828 -0.052249
C 1.138315 -2.633594 -1.052645
C 0.065340 -2.229428 -2.064966
C -1.250322 -1.834094 -1.404379
C -2.727554 -1.980334 1.096471
C -2.362428 -2.454060 2.424626
C -2.469351 0.534375 1.473996
C -0.823278 0.624586 -1.041190
C 2.429480 -3.077815 -1.735690
C 2.800104 0.417786 0.016764
C 3.076968 1.682765 -0.798037
C 4.100448 2.547363 -0.069828
C 5.327372 1.726336 0.321162
C 4.902792 0.495701 1.124113
C 6.073715 -0.416144 1.482024
C -5.039761 1.388560 -0.237816
C -6.231305 1.844812 0.494705
C -6.746431 3.083565 0.468028
C -6.280039 4.283770 -0.277235
O -1.783333 -3.049723 -0.838700
H -1.933709 -1.478058 -2.193425
O -3.724263 -2.065608 2.136880
H -3.347441 -3.865363 0.171059
O 2.008178 -0.411468 -0.761130
O -4.812357 0.080724 0.052298
O -4.342419 2.038845 -0.997825
O 4.012704 -0.281720 0.319129
O 4.477748 3.689438 -0.838591
O 6.213186 2.504231 1.132916
O 3.620019 1.375850 -2.079910
O 6.894855 0.132143 2.509517
H 0.747241 -3.474992 -0.463892
H 2.060608 -1.854082 0.725764
H 0.262608 -0.223379 1.283311
H -0.227429 -1.908077 1.257849
H 0.426424 -1.395344 -2.678458
H -0.134641 -3.061935 -2.750825
H -5.023552 -2.231076 -0.427283
H -4.064781 -2.245673 -1.918630
H 3.197470 -3.330125 -0.995809
H 2.827894 -2.284874 -2.376690
H 2.249514 -3.961644 -2.357119
H -0.003341 0.534632 -1.755099
H -1.701110 1.000153 -1.574840
H -0.529711 1.380853 -0.306776
H -1.837342 -1.787542 3.109611
H -2.210594 -3.521854 2.587853
H -3.401328 0.005432 -1.447448
H -3.360138 0.478227 2.104146
H -1.594214 0.495509 2.128676
H -2.470102 1.502510 0.967163
H -6.711175 1.087566 1.108236
H -7.636165 3.239505 1.079590
H -6.105610 5.105170 0.429629
H -5.377592 4.101757 -0.857208
H -7.080008 4.628652 -0.945563
H 2.306290 0.696646 0.969117
H 2.131050 2.240224 -0.899782
H 4.401077 0.819215 2.054958
H 5.844071 1.381700 -0.590025
H 3.642094 2.936570 0.849767
H 4.673818 3.373495 -1.732781

H 6.436946 3.299913 0.630916
H 3.005662 0.773062 -2.519480
H 6.655467 -0.631049 0.570716
H 5.674016 -1.362163 1.862844
H 7.148308 1.021196 2.220335

3b-c55 , delta G = 1.1191 kcal/mol, population = 7.89 %
C -2.932014 -2.840133 -0.144181
C -3.973467 -2.005532 -0.902381
C -3.612200 -0.536050 -0.593819
C -2.426309 -0.584298 0.437968
C -1.059759 -0.669646 -0.361837
C 0.100326 -1.011766 0.604829
C 1.415870 -1.431387 -0.053035
C 1.208490 -2.562198 -1.055663
C 0.141563 -2.167817 -2.078116
C -1.184584 -1.787214 -1.430116
C -2.684744 -1.956113 1.055139
C -2.326586 -2.431259 2.384581
C -2.455138 0.558983 1.444080
C -0.788128 0.674944 -1.057941
C 2.510297 -2.993908 -1.726340
C 2.832683 0.503586 0.027892
C 3.128896 1.758403 -0.795419
C 4.141359 2.621627 -0.053063
C 5.368986 1.812431 0.368733
C 4.921492 0.583074 1.163340
C 6.088300 -0.308416 1.549157
C -5.014613 1.394934 -0.282896
C -6.218846 1.835121 0.438750
C -6.727558 3.076770 0.438595
C -6.238466 4.296508 -0.258960
O -1.711175 -3.009807 -0.873238
H -1.863592 -1.435509 -2.224921
O -3.689632 -2.054558 2.086562
H -3.277040 -3.844277 0.118187
O 2.049156 -0.329921 -0.756356
O -4.781278 0.086024 -0.002999
O -4.312660 2.057967 -1.027499
O 4.036466 -0.193458 0.351767
O 4.527591 3.766853 -0.813673
O 6.218033 2.602067 1.200342
O 3.693452 1.436263 -2.064048
O 5.702092 -1.388620 2.401701
H 0.819907 -3.407560 -0.470914
H 2.108234 -1.772981 0.729829
H 0.286238 -0.163212 1.273314
H -0.184750 -1.853716 1.243645
H 0.500281 -1.328942 -2.686420
H -0.042861 -3.001128 -2.767389
H -4.963452 -2.225091 -0.491935
H -3.989721 -2.225254 -1.973710
H 3.273572 -3.239170 -0.979240
H 2.907323 -2.197002 -2.363349
H 2.344424 -3.879056 -2.349889
H -1.668021 1.046822 -1.590702
H -0.499545 1.430662 -0.320986
H 0.032718 0.592586 -1.771735
H -1.813759 -1.762839 3.076909
H -2.166064 -3.498214 2.545060
H -3.354729 0.031113 -1.488975
H -3.356346 0.496911 2.058579
H -1.591079 0.519276 2.113296
H -2.450210 1.529282 0.941562
H -6.713013 1.062861 1.021444
H -7.628955 3.219251 1.036194
H -5.317787 4.129781 -0.814419
H -7.016196 4.658680 -0.944289
H -6.088551 5.098930 0.474677
H 2.322125 0.794288 0.968256
H 2.188761 2.321317 -0.920063
H 4.398398 0.921752 2.078583
H 5.910023 1.472485 -0.530974
H 3.666047 3.010851 0.858286
H 4.761544 3.450901 -1.698622
H 6.343990 3.445847 0.744324
H 3.095292 0.813075 -2.497720
H 6.868938 0.307739 2.014044
H 6.499928 -0.763333 0.640873
H 5.482081 -1.018708 3.265448

3b-c44 , delta G = 1.4337 kcal/mol, population = 4.64 %
C -3.114177 -2.520705 -0.131860
C -4.050269 -1.678705 -1.010383
C -3.592924 -0.218789 -0.800588
C -2.459359 -0.266840 0.288098

C -1.070965 -0.521939 -0.434046
C 0.016230 -0.861147 0.614035
C 1.324782 -1.424143 0.056651
C 1.075226 -2.624775 -0.851328
C 0.085320 -2.248432 -1.955494
C -1.234516 -1.715993 -1.409782
C -2.853205 -1.556941 1.001222
C -2.599726 -1.937655 2.384095
C -2.437317 0.957275 1.194191
C -0.667261 0.733129 -1.227160
C 2.368286 -3.202952 -1.421207
C 2.834147 0.426094 0.028555
C 3.236230 1.624639 -0.825510
C 4.133218 2.536936 0.016465
C 5.321896 1.763943 0.589078
C 4.844405 0.509255 1.320779
C 5.977268 -0.397868 1.770684
C -4.848890 1.824736 -0.672888
C -6.003332 2.516865 -0.078278
C -6.752952 2.166326 0.978723
C -6.670178 0.977587 1.872606
O -1.878107 -2.843185 -0.778945
H -1.849101 -1.386260 -2.263984
O -3.912929 -1.487534 1.980324
H -3.549386 -3.469593 0.194338
O 2.060154 -0.439595 -0.716901
O -4.739311 0.527957 -0.311718
O -4.082434 2.379719 -1.445074
O 4.026431 -0.273086 0.435603
O 4.585622 3.662436 -0.735403
O 6.045293 2.560745 1.524992
O 3.901035 1.229656 -2.026281
O 6.778610 -0.853704 0.680955
H 0.601275 -3.385210 -0.215398
H 1.959500 -1.739427 0.898347
H 0.229098 0.025267 1.222530
H -0.355078 -1.626891 1.303523
H 0.535825 -1.498589 -2.616044
H -0.132464 -3.126200 -2.576478
H -5.074095 -1.791819 -0.641616
H -4.029074 -1.985127 -2.060020
H 2.162805 -4.123020 -1.978927
H 3.079114 -3.440725 -0.621337
H 2.848619 -2.491970 -2.100967
H -1.491695 1.117737 -1.834278
H -0.355069 1.529067 -0.544065
H 0.177484 0.528314 -1.885660
H -2.069653 -1.250633 3.044509
H -2.533498 -2.994960 2.643957
H -3.259125 0.255372 -1.723494
H -1.609255 0.906269 1.906463
H -2.329426 1.876806 0.614354
H -3.365452 1.021206 1.767400
H -6.212180 3.461977 -0.572832
H -7.547438 2.868907 1.234958
H -7.522973 0.313972 1.671949
H -5.754249 0.401787 1.754551
H -6.775335 1.298682 2.915394
H 2.311704 0.780439 0.938477
H 2.337883 2.174292 -1.132285
H 4.260108 0.809893 2.209462
H 5.983441 1.453737 -0.237826
H 3.546374 2.942344 0.853022
H 4.907500 3.313938 -1.580678
H 6.248284 3.396376 1.082844
H 4.491961 0.495183 -1.802989
H 5.548416 -1.246211 2.325283
H 6.638689 0.157560 2.441818
H 6.199805 -1.355410 0.092140

3b-c7 , delta G = 1.9159 kcal/mol, population = 2.05 %

C -3.135014 -2.544535 -0.108055
C -4.063584 -1.727123 -1.017204
C -3.614800 -0.260760 -0.835541
C -2.493113 -0.278993 0.266254
C -1.095747 -0.545240 -0.433500
C -0.019237 -0.856110 0.634916
C 1.296526 -1.426604 0.104614
C 1.063890 -2.647632 -0.780347
C 0.083318 -2.302191 -1.902204
C -1.244165 -1.762325 -1.382886
C -2.891190 -1.553358 1.005123
C -2.653708 -1.900481 2.399622
C -2.484235 0.966480 1.143147
C -0.689538 0.693211 -1.251438
C 2.365458 -3.231310 -1.324014
C 2.854434 0.395433 0.054519

C 3.178398 1.602257 -0.828282
C 4.233160 2.466398 -0.146510
C 5.430158 1.623687 0.287042
C 4.963248 0.453780 1.155560
C 6.100035 -0.482453 1.557679
C -4.883210 1.778424 -0.766502
C -6.047478 2.475755 -0.197993
C -6.812943 2.136039 0.851325
C -6.741586 0.958180 1.760600
O -1.890006 -2.876937 -0.732933
H -1.850522 -1.456121 -2.251719
O -3.963267 -1.464274 1.968707
H -3.570135 -3.487385 0.235180
O 2.032338 -0.447482 -0.675314
O -4.769264 0.491016 -0.375135
O -4.112131 2.321085 -1.543057
O 4.039468 -0.332012 0.397717
O 4.649071 3.553539 -0.972952
O 6.344309 2.411486 1.057285
O 3.708193 1.205874 -2.091003
O 6.952533 0.090192 2.545521
H 0.586895 -3.396230 -0.132712
H 1.924953 -1.719616 0.958459
H 0.188027 0.046071 1.222048
H -0.397009 -1.605207 1.338977
H 0.537087 -1.567038 -2.577300
H -0.124240 -3.195397 -2.504363
H -5.091464 -1.836090 -0.658666
H -4.028386 -2.057427 -2.059202
H 2.168596 -4.159784 -1.870810
H 3.066983 -3.454698 -0.512285
H 2.854191 -2.529624 -2.007524
H -1.515647 1.071470 -1.860264
H -0.367284 1.499350 -0.585405
H 0.149156 0.471866 -1.912661
H -2.134398 -1.196249 3.050334
H -2.588094 -2.951169 2.685096
H -3.273745 0.194194 -1.765529
H -2.374757 1.872409 0.542423
H -3.417377 1.040091 1.707013
H -1.661444 0.935107 1.862362
H -6.251987 3.413283 -0.708490
H -7.613989 2.838950 1.085187
H -7.579460 0.281233 1.541980
H -5.815700 0.392675 1.674965
H -6.881261 1.288534 2.796280
H 2.370631 0.745928 0.988334
H 2.253930 2.188229 -0.960800
H 4.479382 0.845990 2.069330
H 5.933469 1.214371 -0.604637
H 3.790297 2.918635 0.751663
H 4.830718 3.185841 -1.850191
H 6.599605 3.168328 0.512175
H 3.063776 0.617320 -2.506000
H 6.664002 -0.773893 0.656347
H 5.666250 -1.387882 1.995668
H 7.233194 0.953063 2.206289

3c

3c-c36 , delta G = 0.0000 kcal/mol, population = 41.54 %

C -2.888434 -2.842044 -0.051901
C -3.828233 -1.882819 -0.794664
C -3.231631 -0.477683 -0.563755
C -2.017426 -0.673327 0.416205
C -0.723079 -0.992743 -0.438876
C 0.414632 -1.484913 0.485128
C 1.652564 -2.040748 -0.221733
C 1.299753 -3.153035 -1.212538
C 0.167361 -2.700137 -2.146666
C -1.061472 -2.116899 -1.452384
C -2.447592 -1.967860 1.099041
C -2.088260 -2.446620 2.426328
C -1.818582 0.498038 1.370875
C -0.293023 0.276056 -1.196576
C 1.016676 -4.470307 -0.483166
C 3.399596 -0.384433 -0.261745
C 4.230912 0.404869 -1.276084
C 5.264339 1.245002 -0.535501
C 4.605588 2.102252 0.544504
C 3.783047 1.213384 1.476907
C 2.993512 1.988914 2.518177
C -4.310362 1.655345 -0.287654
C -5.399995 2.303299 0.459124
C -5.719803 3.605711 0.414245
C -5.088784 4.704937 -0.364940
O -1.746545 -3.222074 -0.828093
H -1.712502 -1.696970 -2.238423

O -3.395553 -1.879069 2.183466
H -3.368300 -3.772013 0.266263
O 2.387141 -1.025494 -0.958242
O -4.259354 0.340125 0.049941
O -3.556784 2.170906 -1.095734
O 2.815857 0.485700 0.711042
O 2.064724 2.899565 1.931268
O 5.594875 2.757357 1.337260
O 6.032599 2.052856 -1.427999
O 3.426515 1.300687 -2.039193
H 2.191173 -3.311796 -1.835136
H 2.332201 -2.444423 0.546088
H 0.725234 -0.683873 1.159062
H 0.044581 -2.304131 1.110123
H 0.555563 -1.939410 -2.833429
H -0.160689 -3.541299 -2.769901
H -4.818288 -1.933864 -0.332026
H -3.931976 -2.132338 -1.854605
H 0.191145 -4.380612 0.226718
H 0.743925 -5.247708 -1.204612
H 1.909449 -4.805935 0.056901
H 0.425170 0.042976 -1.983468
H -1.144612 0.787917 -1.653401
H 0.198177 0.977452 -0.515943
H -1.442397 -1.839404 3.061294
H -2.079466 -3.519448 2.623579
H -2.933277 0.008167 -1.493002
H -0.919913 0.363698 1.978931
H -1.717678 1.438718 0.823778
H -2.673353 0.583806 2.045726
H -5.974103 1.640907 1.100776
H -6.561332 3.908717 1.038721
H -4.794202 5.511701 0.318275
H -4.225758 4.377411 -0.941084
H -5.833329 5.143030 -1.042841
H 4.051822 -1.112776 0.264008
H 4.463814 0.515225 1.998731
H 3.935871 2.837721 0.071581
H 5.980701 0.569801 -0.046944
H 4.745229 -0.314461 -1.934413
H 3.681722 2.582984 3.126205
H 2.478548 1.269484 3.173594
H 1.488723 2.384401 1.350297
H 6.187543 3.211161 0.722677
H 5.402750 2.514721 -2.000352
H 2.759269 0.774599 -2.498859

3c-c42 , delta G = 0.1096 kcal/mol, population = 34.51 %

C -2.889426 -2.807747 0.016462
C -3.863768 -1.908502 -0.755738
C -3.306491 -0.478346 -0.589745
C -2.081465 -0.596392 0.389338
C -0.783357 -0.909853 -0.462719
C 0.377761 -1.324033 0.470084
C 1.623648 -1.876463 -0.223136
C 1.297920 -3.038111 -1.164348
C 0.145800 -2.659500 -2.107377
C -1.094509 -2.084704 -1.426739
C -2.466748 -1.874666 1.127269
C -2.084996 -2.289996 2.469950
C -1.914124 0.616299 1.296067
C -0.398456 0.338166 -1.278376
C 1.059583 -4.331052 -0.377218
C 3.407232 -0.275488 -0.369931
C 4.149541 0.565077 -1.411566
C 5.274334 1.335190 -0.728203
C 4.760178 2.115171 0.483647
C 4.017415 1.167028 1.422471
C 3.386619 1.882824 2.601862
C -4.445703 1.632690 -0.405972
C -5.551044 2.282804 0.315460
C -5.932572 3.562496 0.183645
C -5.370577 4.628427 -0.689151
O -1.742674 -3.183896 -0.754251
H -1.763515 -1.714117 -2.222507
O -3.409661 -1.770356 2.214707
H -3.338035 -3.738213 0.376030
O 2.318142 -0.858842 -0.997011
O -4.354507 0.336693 -0.007840
O -3.710842 2.131640 -1.241231
O 2.942250 0.546544 0.704588
O 2.727084 0.974846 3.484171
O 5.845821 2.692228 1.206032
O 5.955441 2.201133 -1.635678
O 3.294897 1.525935 -2.026760
H 2.189804 -3.196195 -1.786584
H 2.318703 -2.229866 0.554688

H 0.674663 -0.478730 1.095906
H 0.039413 -2.117870 1.143692
H 0.505976 -1.918228 -2.829614
H -0.162027 -3.535766 -2.691190
H -4.847333 -1.968687 -0.280524
H -3.970072 -2.205083 -1.803227
H 0.237609 -4.235035 0.336189
H 0.803478 -5.146104 -1.062264
H 1.966143 -4.617195 0.168307
H 0.087308 1.078805 -0.636529
H 0.308904 0.088853 -2.070381
H -1.270413 0.809851 -1.739885
H -1.453600 -1.640302 3.076816
H -2.043916 -3.353482 2.709332
H -3.026665 -0.026442 -1.541550
H -1.023469 0.520098 1.922925
H -1.818124 1.534761 0.711712
H -2.779696 0.717083 1.954767
H -6.084072 1.642163 1.012272
H -6.776470 3.871523 0.801936
H -4.511123 4.295788 -1.267710
H -6.152516 4.988010 -1.371006
H -5.089920 5.492313 -0.073079
H 4.094163 -1.042092 0.045179
H 4.716046 0.394218 1.792363
H 4.059272 2.896162 0.142059
H 6.026028 0.615849 -0.374696
H 4.577413 -0.115677 -2.165740
H 2.687845 2.646819 2.224522
H 4.166963 2.381185 3.183160
H 2.054951 0.515461 2.964070
H 6.390516 3.163198 0.560282
H 5.275757 2.703235 -2.108568
H 2.564229 1.046621 -2.438750

3c-c77 , delta G = 1.0710 kcal/mol, population = 6.80 %
C -3.015158 -2.495117 -0.086575
C -3.866711 -1.593520 -0.990566
C -3.228532 -0.193035 -0.875336
C -2.079356 -0.320635 0.190446
C -0.753241 -0.781506 -0.546387
C 0.313000 -1.194809 0.494558
C 1.558778 -1.882667 -0.065907
C 1.209150 -3.093494 -0.933765
C 0.158210 -2.710268 -1.987039
C -1.081323 -2.003555 -1.443149
C -2.603801 -1.512183 0.984435
C -2.348689 -1.849422 2.377948
C -1.883709 0.940673 1.022001
C -0.233760 0.374873 -1.418689
C 0.817360 -4.294909 -0.066676
C 3.426571 -0.370124 -0.223656
C 4.333182 0.240948 -1.295649
C 5.428215 1.061890 -0.625082
C 4.841398 2.066488 0.367223
C 3.946141 1.332499 1.362973
C 3.244226 2.273402 2.323552
C -4.228545 1.991532 -0.849098
C -5.272482 2.851753 -0.269676
C -6.034915 2.649178 0.816632
C -6.079969 1.505145 1.769336
O -1.849114 -3.003815 -0.742510
H -1.667935 -1.649871 -2.308337
O -3.612534 -1.264424 1.987471
H -3.553475 -3.363677 0.303564
O 2.371509 -0.988591 -0.874393
O -4.260191 0.716038 -0.407222
O -3.428740 2.402326 -1.675954
O 2.912164 0.644819 0.644921
O 2.441575 1.567794 3.270170
O 5.881129 2.713299 1.098286
O 6.261778 1.720260 -1.578827
O 3.616286 1.119741 -2.159287
H 2.126338 -3.367752 -1.473387
H 2.178708 -2.215264 0.781793
H 0.629661 -0.322736 1.071474
H -0.124755 -1.905995 1.202315
H 0.621748 -2.051048 -2.729467
H -0.171282 -3.605379 -2.528776
H -4.885158 -1.557871 -0.592664
H -3.913954 -1.957462 -2.020837
H 1.659594 -4.597183 0.566252
H -0.041351 -4.078915 0.573075
H 0.546386 -5.144859 -0.701661
H 0.517852 0.031276 -2.130572
H -1.038816 0.854679 -1.982341
H 0.242011 1.136832 -0.795056

H -1.717704 -1.198535 2.983857
H -2.401036 -2.892113 2.693958
H -2.864216 0.184640 -1.830624
H -1.698207 1.808841 0.384949
H -2.775164 1.139523 1.621727
H -1.037232 0.837072 1.706676
H -5.375687 3.789200 -0.809933
H -6.730827 3.456720 1.049295
H -5.246387 0.813173 1.666312
H -6.119958 1.888295 2.795561
H -7.013794 0.945476 1.618431
H 4.007945 -1.093704 0.384492
H 4.552231 0.606109 1.934787
H 4.231309 2.805470 -0.179987
H 6.085728 0.379844 -0.068251
H 4.790713 -0.581494 -1.869664
H 2.637429 2.987826 1.744184
H 3.990326 2.832784 2.894270
H 1.797414 1.054459 2.765516
H 6.524921 3.024153 0.446657
H 5.673446 2.165816 -2.205880
H 2.911198 0.608283 -2.577103

3c-c48 , delta G = 1.2338 kcal/mol, population = 5.16 %

C -2.872725 -2.816142 -0.026244
C -3.820865 -1.873016 -0.779073
C -3.226805 -0.462297 -0.577375
C -2.005053 -0.636674 0.396913
C -0.715821 -0.967620 -0.461658
C 0.430253 -1.438013 0.463076
C 1.664358 -2.003631 -0.242444
C 1.308183 -3.133930 -1.211197
C 0.167271 -2.702464 -2.145052
C -1.058121 -2.110662 -1.452316
C -2.425888 -1.920222 1.105448
C -2.055198 -2.374414 2.438246
C -1.802540 0.551014 1.330042
C -0.293387 0.287908 -1.245607
C 1.035033 -4.439052 -0.456600
C 3.372743 -0.307435 -0.303718
C 4.252926 0.412549 -1.328702
C 5.251590 1.298396 -0.593674
C 4.554422 2.214236 0.414625
C 3.675418 1.377938 1.347578
C 2.873100 2.241759 2.304880
C -4.316095 1.669976 -0.334146
C -5.398482 2.327266 0.415635
C -5.743094 3.621232 0.330647
C -5.153972 4.700497 -0.507266
O -1.736115 -3.207172 -0.804572
H -1.715997 -1.706059 -2.240796
O -3.366540 -1.816063 2.195270
H -3.347821 -3.741417 0.312247
O 2.399528 -1.005951 -1.002327
O -4.252787 0.362817 0.029965
O -3.577146 2.173480 -1.163058
O 2.741344 0.632569 0.565684
O 2.110083 1.473363 3.237593
O 5.519766 2.903580 1.207626
O 6.055999 2.060131 -1.494514
O 3.488032 1.257494 -2.185029
H 2.195502 -3.301046 -1.837503
H 2.348483 -2.393670 0.528840
H 0.742395 -0.624392 1.121031
H 0.069101 -2.248164 1.105114
H 0.547526 -1.953201 -2.848655
H -0.162457 -3.556219 -2.750093
H -4.807262 -1.918569 -0.308121
H -3.931662 -2.141700 -1.833616
H 1.932434 -4.761656 0.083767
H 0.213740 -4.339698 0.256999
H 0.760209 -5.230148 -1.162231
H 0.429348 0.042272 -2.024662
H -1.147742 0.781922 -1.716891
H 0.191529 1.005743 -0.578758
H -1.406894 -1.753767 3.057545
H -2.040986 -3.443587 2.654358
H -2.937011 0.008227 -1.517116
H -1.704288 1.481751 0.765745
H -2.654436 0.648278 2.007000
H -0.900578 0.428408 1.935338
H -5.945316 1.679159 1.094629
H -6.573757 3.933095 0.965251
H -4.838848 5.530420 0.138222
H -4.311056 4.361956 -1.106393
H -5.929313 5.111582 -1.167132
H 3.999228 -0.992727 0.305080

H 4.322616 0.688685 1.924010
H 3.912178 2.929716 -0.127174
H 5.947341 0.653652 -0.038613
H 4.797349 -0.345839 -1.915209
H 2.150659 2.830439 1.727544
H 3.554457 2.932622 2.818438
H 2.726691 1.053398 3.849888
H 6.157962 3.291162 0.592691
H 5.451783 2.479793 -2.124249
H 2.821815 0.707882 -2.618278

3c-c5 , delta G = 1.2748 kcal/mol, population = 4.82 %
C -2.936811 -2.860115 -0.037282
C -3.895571 -1.942148 -0.807105
C -3.327049 -0.519321 -0.617678
C -2.110908 -0.661289 0.369173
C -0.808723 -0.976895 -0.475950
C 0.340882 -1.412379 0.461753
C 1.587404 -1.969014 -0.227970
C 1.257961 -3.116812 -1.184996
C 0.117653 -2.715774 -2.133071
C -1.122989 -2.137281 -1.456237
C -2.514494 -1.944705 1.088160
C -2.147241 -2.379451 2.428634
C -1.938833 0.538623 1.292087
C -0.406108 0.276859 -1.273972
C 1.000022 -4.416161 -0.414936
C 3.352200 -0.338938 -0.329826
C 4.137081 0.469129 -1.366640
C 5.214377 1.287282 -0.662632
C 4.618573 2.094990 0.488782
C 3.851290 1.172976 1.437115
C 3.150014 1.926133 2.565228
C -4.446418 1.600032 -0.407603
C -5.549968 2.249702 0.317112
C -5.897498 3.542654 0.227736
C -5.290473 4.627400 -0.590049
O -1.787010 -3.238251 -0.802414
H -1.781552 -1.751674 -2.253637
O -3.465144 -1.844736 2.169476
H -3.397597 -3.790363 0.307196
O 2.301530 -0.955651 -0.989173
O -4.372652 0.296477 -0.032017
O -3.698863 2.106205 -1.227191
O 2.824739 0.510784 0.694711
O 4.061551 2.398613 3.552954
O 5.654182 2.740220 1.237201
O 5.916082 2.137516 -1.569187
O 3.302349 1.389009 -2.066241
H 2.153228 -3.276947 -1.801773
H 2.273395 -2.337509 0.551294
H 0.640465 -0.579825 1.102374
H -0.010230 -2.213264 1.120527
H 0.491061 -1.969037 -2.842930
H -0.193225 -3.581941 -2.730261
H -4.884375 -1.999403 -0.342523
H -3.994177 -2.224546 -1.859267
H 0.739814 -5.220199 -1.111360
H 1.899493 -4.718568 0.133560
H 0.174181 -4.319444 0.293822
H 0.075461 1.008333 -0.618802
H 0.310827 0.031295 -2.058367
H -1.269141 0.757899 -1.742559
H -1.514443 -1.742350 3.047217
H -2.117499 -3.446068 2.655394
H -3.036238 -0.056692 -1.561006
H -1.834389 1.463629 0.719707
H -2.806460 0.636889 1.948550
H -1.051035 0.428231 1.920330
H -6.110465 1.597422 0.980794
H -6.745561 3.848991 0.841653
H -6.045315 5.027278 -1.280030
H -5.010558 5.462632 0.064570
H -4.422213 4.297782 -1.157093
H 4.025430 -1.083280 0.144444
H 4.548171 0.435217 1.876269
H 3.913970 2.841798 0.086195
H 5.964082 0.599795 -0.247350
H 4.611697 -0.237197 -2.067389
H 2.462888 1.236831 3.068055
H 2.556358 2.749743 2.135449
H 4.737202 2.913844 3.087904
H 6.157432 3.285127 0.616815
H 5.247168 2.589066 -2.104465
H 2.597510 0.881053 -2.488980

3c-c38 , delta G = 1.5339 kcal/mol, population =

3.11 %
C -1.881540 -2.172945 0.779699
C -3.372596 -1.899271 0.555906
C -3.427273 -0.514172 -0.120169
C -1.947910 0.016058 -0.164155
C -1.251288 -0.509407 -1.482646
C 0.277567 -0.205523 -1.489390
C 1.168910 -1.405486 -1.190142
C 0.976551 -2.523638 -2.215604
C -0.517725 -2.681712 -2.601566
C -1.472302 -2.041767 -1.603940
C -1.347756 -0.774705 0.992351
C -0.172328 -0.450095 1.788556
C -1.862089 1.529719 -0.008878
C -1.870187 0.182487 -2.708841
C 1.588847 -3.834664 -1.723409
C 3.154563 -0.594618 -0.080043
C 4.670268 -0.728992 -0.243163
C 5.362164 -0.136796 0.976716
C 4.875387 1.284548 1.258552
C 3.348122 1.299709 1.360254
C 2.813488 2.712760 1.522426
C -5.055078 1.247696 0.095254
C -5.771896 2.029092 1.115419
C -6.634935 3.029066 0.879715
C -7.086933 3.598556 -0.418358
O -1.248985 -2.751197 -0.366443
H -2.509518 -2.219606 -1.935523
O -1.503462 -0.259218 2.328687
H -1.675074 -2.847346 1.615759
O 2.568074 -1.018775 -1.264626
O -4.248827 0.344765 0.711798
O -5.144011 1.352267 -1.116328
O 2.784973 0.766824 0.157817
O 1.405591 2.754419 1.767845
O 5.406839 1.752408 2.497688
O 6.784665 -0.168481 0.854339
O 5.148970 -0.023468 -1.385732
H 1.523536 -2.198312 -3.112120
H 0.982522 -1.792141 -0.180654
H 0.560857 0.154005 -2.487538
H 0.523019 0.597314 -0.792920
H -0.708043 -2.245273 -3.587196
H -0.767436 -3.746029 -2.681972
H -3.875534 -1.847121 1.526160
H -3.853430 -2.681966 -0.037809
H 1.540020 -4.604645 -2.501045
H 2.640174 -3.697337 -1.447482
H 1.043486 -4.200750 -0.846617
H -2.956809 0.070040 -2.759793
H -1.640168 1.251732 -2.693543
H -1.445969 -0.226878 -3.630480
H 0.380102 0.469620 1.593248
H 0.392668 -1.260726 2.251839
H -3.881526 -0.544722 -1.110702
H -0.848624 1.895735 -0.189402
H -2.531373 2.033367 -0.711129
H -2.146853 1.821881 1.004476
H -5.555684 1.750345 2.142869
H -7.070758 3.496872 1.763578
H -8.175986 3.487292 -0.503495
H -6.894474 4.679067 -0.427474
H -6.608072 3.133235 -1.277532
H 2.823106 -1.213525 0.778541
H 3.041914 0.684269 2.228530
H 5.181530 1.945619 0.429487
H 5.119978 -0.756798 1.851241
H 4.914313 -1.801770 -0.318389
H 2.982135 3.258314 0.586748
H 3.370183 3.215472 2.323309
H 1.256892 2.503554 2.687861
H 6.358831 1.583312 2.471562
H 6.999000 0.191930 -0.018526
H 4.674623 -0.360746 -2.157014

3c-c84 , delta G = 1.6643 kcal/mol, population = 2.49 %
C -2.086150 -2.007588 0.846829
C -3.555764 -1.792167 0.468037
C -3.560369 -0.526620 -0.413156
C -2.082540 0.008605 -0.427559
C -1.290995 -0.705526 -1.594468
C 0.233773 -0.389620 -1.543274
C 1.106579 -1.514013 -0.996786
C 0.985803 -2.782524 -1.843511
C -0.476192 -3.019641 -2.303498
C -1.500458 -2.241176 -1.491112
C -1.575813 -0.585252 0.881269

C -0.479508 -0.112294 1.714861
C -2.008020 1.529161 -0.498334
C -1.820411 -0.218959 -2.954305
C 1.570433 -3.990687 -1.112939
C 3.039226 -0.568465 0.101343
C 4.562472 -0.651844 -0.014395
C 5.201256 0.077750 1.159693
C 4.655114 1.497970 1.284857
C 3.128771 1.467554 1.361116
C 2.503605 2.853337 1.361287
C -5.175122 1.248975 -0.567958
C -5.994550 2.222683 0.170704
C -5.906682 2.610853 1.452908
C -4.973160 2.187368 2.533598
O -1.365313 -2.744895 -0.144616
H -2.510534 -2.483050 -1.863238
O -1.853039 0.128825 2.103142
H -1.945660 -2.542801 1.790334
O 2.504236 -1.118780 -1.053406
O -4.442170 0.436548 0.223162
O -5.187618 1.176860 -1.787215
O 2.602003 0.791747 0.210854
O 2.836805 3.602577 0.194257
O 5.132114 2.115504 2.479612
O 6.626627 0.089343 1.067778
O 5.039637 -0.033918 -1.207284
H 1.593928 -2.593206 -2.739602
H 0.860972 -1.735751 0.049270
H 0.585091 -0.195567 -2.565488
H 0.432618 0.519953 -0.974780
H -0.596046 -2.750145 -3.357644
H -0.718658 -4.085980 -2.228230
H -4.135884 -1.600715 1.375675
H -3.985391 -2.662890 -0.035352
H 2.599208 -3.792595 -0.792194
H 0.970332 -4.228951 -0.227591
H 1.582362 -4.872392 -1.762885
H -1.332511 -0.763279 -3.768212
H -2.900841 -0.347384 -3.063600
H -1.590060 0.841658 -3.090519
H 0.075419 0.778034 1.420987
H 0.056854 -0.820964 2.347440
H -3.940105 -0.709994 -1.417928
H -0.981790 1.876076 -0.644554
H -2.611554 1.914690 -1.323727
H -2.380170 1.967853 0.430576
H -6.738190 2.695695 -0.465383
H -6.630814 3.368757 1.755787
H -4.611990 3.073095 3.069065
H -5.524239 1.590122 3.273554
H -4.127665 1.600222 2.180711
H 2.707355 -1.120383 1.004027
H 2.824110 0.943263 2.286382
H 4.952206 2.082302 0.399719
H 4.962827 -0.467620 2.083564
H 4.848417 -1.716354 0.013688
H 2.877641 3.417392 2.220378
H 1.411994 2.749292 1.462241
H 2.565082 3.075612 -0.569098
H 6.093454 2.011454 2.480381
H 6.846105 0.389426 0.173679
H 4.613596 -0.474089 -1.954462

3c-c11 , delta G = 1.9415 kcal/mol, population = 1.56 %

C -1.913891 -2.174525 0.774043
C -3.407416 -1.910860 0.552317
C -3.472133 -0.534040 -0.140045
C -1.995278 0.002639 -0.196049
C -1.301349 -0.536773 -1.510508
C 0.226705 -0.229926 -1.526806
C 1.121913 -1.425189 -1.219824
C 0.928087 -2.553755 -2.233072
C -0.568004 -2.720215 -2.608336
C -1.518037 -2.071165 -1.612280
C -1.390117 -0.769782 0.969943
C -0.226090 -0.420353 1.773226
C -1.915477 1.518321 -0.059158
C -1.926974 0.138807 -2.742333
C 1.547509 -3.857700 -1.731045
C 3.110205 -0.615438 -0.120690
C 4.626485 -0.738498 -0.286594
C 5.321635 -0.148446 0.935061
C 4.809261 1.260966 1.221952
C 3.284540 1.255637 1.344175
C 2.700782 2.654316 1.530386
C -5.108191 1.221617 0.060211
C -5.828967 2.007885 1.073580

C -6.708737 2.991071 0.829078
C -7.179630 3.532879 -0.474015
O -1.283642 -2.763432 -0.367482
H -2.556636 -2.256458 -1.935068
O -1.561061 -0.241990 2.300279
H -1.701297 -2.837581 1.617533
O 2.520268 -1.034247 -1.303432
O -4.294725 0.330323 0.684678
O -5.198309 1.313767 -1.152283
O 2.727464 0.743274 0.131020
O 2.927838 3.168645 2.839223
O 5.349559 1.751586 2.453134
O 6.742171 -0.153272 0.795722
O 5.098392 -0.023024 -1.425757
H 1.468830 -2.235615 -3.135919
H 0.940859 -1.801764 -0.205569
H 0.504892 0.121436 -2.529246
H 0.473333 0.579167 -0.838308
H -0.765271 -2.295765 -3.597778
H -0.815368 -3.786004 -2.674596
H -3.907584 -1.850148 1.523426
H -3.885594 -2.703139 -0.030656
H 2.600354 -3.714662 -1.463942
H 1.009178 -4.215818 -0.846673
H 1.495459 -4.636008 -2.500085
H -3.013541 0.023509 -2.786874
H -1.699309 1.208689 -2.741615
H -1.506107 -0.280903 -3.660831
H 0.313535 0.505408 1.573461
H 0.351674 -1.214176 2.248868
H -3.929381 -0.578319 -1.128629
H -2.587062 2.011634 -0.766412
H -2.200419 1.821159 0.951053
H -0.903328 1.885839 -0.247454
H -5.602309 1.747303 2.103534
H -7.145234 3.465620 1.708996
H -8.268263 3.409031 -0.545787
H -6.998750 4.615045 -0.504055
H -6.704336 3.057797 -1.329819
H 2.785461 -1.239369 0.736549
H 2.986737 0.624768 2.202976
H 5.087236 1.927793 0.388872
H 5.097704 -0.778811 1.806610
H 4.875822 -1.809394 -0.368927
H 1.614887 2.601726 1.392225
H 3.110757 3.322966 0.755802
H 3.880108 3.104172 3.005082
H 6.312824 1.710926 2.378528
H 6.938543 0.198007 -0.085159
H 4.636504 -0.368610 -2.200804

3d

3d-c47 , delta G = 0.0000 kcal/mol, population = 30.52 %

C -2.637132 -2.536631 1.481389
C -3.748228 -2.335530 0.442010
C -3.640364 -0.854178 0.021419
C -2.527495 -0.219605 0.933615
C -1.119289 -0.434543 0.237839
C 0.022250 -0.097366 1.227104
C 1.429750 -0.484789 0.779139
C 1.533470 -1.958117 0.369570
C 0.411568 -2.319989 -0.614210
C -0.995795 -1.923395 -0.177662
C -2.602007 -1.170168 2.124621
C -2.243601 -0.907186 3.511564
C -2.797430 1.237425 1.285134
C -1.028861 0.457854 -1.012508
C 1.602744 -2.860139 1.606464
C 3.108023 0.919793 -0.203258
C 3.277983 1.945078 -1.326302
C 4.713863 2.458624 -1.326981
C 5.718052 1.304099 -1.348848
C 5.419985 0.351213 -0.193015
C 6.306861 -0.879186 -0.195944
C -5.346910 0.729120 -0.589638
C -6.650063 1.244442 -0.141192
C -7.345416 2.234975 -0.720875
C -7.005674 3.047903 -1.919830
O -1.362844 -2.818788 0.891986
H -1.669565 -2.101289 -1.033013
O -3.631645 -0.942681 3.109325
H -2.836213 -3.346522 2.189000
O 1.838219 0.376369 -0.320625
O -4.925751 -0.232266 0.273173
O -4.714449 1.071490 -1.574298
O 4.074241 -0.127264 -0.321872
O 4.955923 3.361159 -2.405896

O 7.047950 1.792464 -1.188075
O 3.034722 1.372467 -2.608781
O 6.008909 -1.747856 0.896870
H 2.482563 -2.068504 -0.168912
H 2.116851 -0.297892 1.618604
H 0.017771 0.973398 1.458680
H -0.148692 -0.626709 2.169274
H 0.614633 -1.833793 -1.575407
H 0.416846 -3.400100 -0.805608
H -4.715176 -2.503373 0.925447
H -3.663029 -3.026114 -0.401969
H 1.647662 -3.912223 1.306166
H 2.503897 -2.634289 2.189264
H 0.730512 -2.740924 2.253799
H -0.882780 1.502550 -0.723225
H -0.181954 0.178622 -1.640927
H -1.935146 0.401253 -1.621939
H -1.880428 0.080614 3.796728
H -1.924889 -1.734471 4.147135
H -3.417033 -0.733485 -1.038766
H -2.933882 1.840996 0.384475
H -3.702435 1.317065 1.891960
H -1.969652 1.664716 1.857148
H -7.054459 0.764067 0.745290
H -8.295949 2.486434 -0.248438
H -7.043695 4.113266 -1.659241
H -6.030120 2.804528 -2.335728
H -7.775582 2.899580 -2.688709
H 3.247132 1.419189 0.777819
H 2.588160 2.784120 -1.137351
H 5.535951 0.889170 0.765593
H 5.616045 0.752279 -2.298875
H 4.880535 3.034201 -0.405974
H 4.614480 2.938101 -3.207293
H 7.160850 2.507947 -1.829074
H 2.137636 1.013365 -2.603360
H 7.351648 -0.576003 -0.087584
H 6.190356 -1.400317 -1.159906
H 5.072808 -1.977931 0.825377

3d-c117 , delta G = 0.1803 kcal/mol, population = 22.50 %

C -2.677880 -2.511175 1.553719
C -3.782049 -2.311812 0.506621
C -3.655960 -0.837468 0.066668
C -2.538924 -0.203448 0.973924
C -1.130418 -0.444096 0.286716
C 0.011183 -0.103261 1.275266
C 1.415973 -0.517331 0.840571
C 1.501273 -1.999293 0.459903
C 0.383237 -2.359219 -0.528900
C -1.021881 -1.940286 -0.105975
C -2.629197 -1.136332 2.177748
C -2.273989 -0.856813 3.562391
C -2.794241 1.261107 1.305090
C -1.025220 0.427877 -0.976373
C 1.542664 -2.880432 1.713308
C 3.090804 0.872209 -0.155671
C 3.308849 1.873253 -1.287030
C 4.725551 2.443264 -1.167912
C 5.766974 1.325268 -1.123480
C 5.414474 0.317519 -0.030458
C 6.318499 -0.903370 -0.013262
C -5.347466 0.751416 -0.572591
C -6.645400 1.287112 -0.132628
C -7.350349 2.247683 -0.749843
C -7.030862 3.000117 -1.993127
O -1.404812 -2.816760 0.973890
H -1.692955 -2.122556 -0.962522
O -3.660343 -0.883340 3.155148
H -2.889602 -3.308554 2.271858
O 1.835736 0.311468 -0.278899
O -4.935065 -0.198053 0.307308
O -4.712289 1.070285 -1.563327
O 4.080454 -0.169260 -0.253006
O 5.011045 3.350797 -2.231614
O 7.061969 1.846775 -0.833265
O 3.110347 1.284219 -2.573262
O 6.284553 -1.625849 -1.243626
H 2.454684 -2.133640 -0.065909
H 2.102754 -0.323020 1.678803
H 0.019796 0.971996 1.485034
H -0.170729 -0.611216 2.227113
H 0.597874 -1.884741 -1.493265
H 0.376623 -3.441514 -0.707729
H -4.753129 -2.462393 0.987411
H -3.700418 -3.014684 -0.327522
H 0.669892 -2.727373 2.352508

H 1.561951 -3.938688 1.432653
H 2.445917 -2.665880 2.296518
H -0.168606 0.144257 -1.588788
H -1.923429 0.359346 -1.596489
H -0.885958 1.477329 -0.701299
H -1.901149 0.131554 3.833006
H -1.966460 -1.677515 4.211875
H -3.428527 -0.732948 -0.994247
H -1.962327 1.688124 1.871289
H -2.925387 1.854047 0.396614
H -3.698266 1.357246 1.911015
H -7.038912 0.847948 0.779778
H -8.294359 2.521111 -0.276486
H -6.076298 2.713981 -2.429861
H -7.831509 2.843102 -2.727801
H -7.030022 4.076183 -1.776354
H 3.219699 1.378983 0.822105
H 2.578013 2.686766 -1.199764
H 5.475886 0.815505 0.954238
H 5.776184 0.798972 -2.093333
H 4.802033 3.025559 -0.238587
H 4.730864 2.909796 -3.048204
H 7.218672 2.566673 -1.459555
H 3.467520 0.384434 -2.542768
H 6.023188 -1.547053 0.829259
H 7.353098 -0.582807 0.139177
H 5.372322 -1.913207 -1.380061

3d-c37 , delta G = 0.2054 kcal/mol, population = 21.57 %
C -2.504950 -2.551331 1.496667
C -3.450722 -2.408010 0.297003
C -3.393939 -0.913010 -0.082613
C -2.483373 -0.220598 0.997328
C -0.973515 -0.316514 0.524508
C -0.030644 0.101739 1.679873
C 1.452919 -0.179434 1.463803
C 1.721019 -1.642669 1.097851
C 0.817900 -2.071373 -0.065761
C -0.669123 -1.785990 0.128619
C -2.670039 -1.200648 2.151746
C -2.552310 -0.945886 3.581012
C -2.914159 1.204138 1.322413
C -0.763804 0.596439 -0.696747
C 1.628023 -2.533829 2.342385
C 3.290180 0.766573 0.210255
C 3.692713 2.202520 -0.140119
C 5.146733 2.235715 -0.597736
C 5.412435 1.203482 -1.694404
C 4.948604 -0.171123 -1.218681
C 5.084194 -1.242146 -2.284654
C -5.101562 0.564528 -0.914354
C -6.487619 0.981473 -0.649878
C -7.156571 1.948007 -1.296930
C -6.703928 2.825071 -2.410304
O -1.137190 -2.728846 1.114543
H -1.174719 -1.997744 -0.829465
O -3.854372 -1.069608 2.965985
H -2.752206 -3.388307 2.155995
O 1.923085 0.752877 0.453969
O -4.746385 -0.393951 -0.019166
O -4.357402 0.976151 -1.788066
O 3.554530 -0.105152 -0.893426
O 5.542988 3.540421 -1.021136
O 6.806891 1.125015 -1.983404
O 2.900724 2.723480 -1.205239
O 4.646145 -2.516099 -1.813258
H 2.756916 -1.710811 0.740793
H 1.987616 0.058040 2.398670
H -0.149873 1.169364 1.893325
H -0.307793 -0.437169 2.589987
H 1.146239 -1.550289 -0.972285
H 0.936584 -3.145089 -0.257048
H -4.465922 -2.659259 0.617997
H -3.179797 -3.068459 -0.531714
H 1.791206 -3.580997 2.067233
H 2.398965 -2.249520 3.067993
H 0.651590 -2.471700 2.828269
H -1.563446 0.486785 -1.434713
H -0.739685 1.645024 -0.385496
H 0.185791 0.388156 -1.191329
H -2.312862 0.057671 3.934314
H -2.276672 -1.763703 4.248329
H -3.024986 -0.749846 -1.095169
H -2.937149 1.825936 0.423985
H -3.913624 1.202706 1.763420
H -2.233429 1.670383 2.039657
H -6.982321 0.446106 0.155590

H -8.179348 2.123098 -0.960377
H -5.668969 2.649826 -2.696918
H -7.353984 2.670375 -3.281593
H -6.835536 3.875658 -2.121207
H 3.867176 0.425275 1.093150
H 3.580620 2.821340 0.765464
H 5.528260 -0.466352 -0.324601
H 4.841235 1.476320 -2.598338
H 5.787817 1.989197 0.260247
H 4.860709 3.856287 -1.631429
H 7.112303 2.030498 -2.132481
H 1.976894 2.695737 -0.922437
H 6.136378 -1.348596 -2.561824
H 4.515931 -0.932524 -3.176643
H 3.726416 -2.410802 -1.535271

3d-c54 , delta G = 0.5989 kcal/mol, population = 11.09 %
C -2.549058 -2.503972 1.647021
C -3.479659 -2.418245 0.429804
C -3.400471 -0.947663 -0.033358
C -2.493433 -0.205857 1.016103
C -0.980072 -0.347384 0.567507
C -0.043794 0.115107 1.710736
C 1.438597 -0.194115 1.523309
C 1.695214 -1.675674 1.229975
C 0.791795 -2.155812 0.086866
C -0.692217 -1.840234 0.255513
C -2.705505 -1.116146 2.221978
C -2.601730 -0.778808 3.635122
C -2.908176 1.240851 1.254375
C -0.742685 0.494509 -0.698924
C 1.591795 -2.504894 2.515623
C 3.303411 0.700992 0.270140
C 3.698301 2.127101 -0.123908
C 5.161518 2.160712 -0.546239
C 5.454769 1.102501 -1.607565
C 5.002669 -0.267732 -1.105530
C 5.160874 -1.375001 -2.133928
C -5.080727 0.500214 -0.966821
C -6.465802 0.943778 -0.744127
C -7.116090 1.881609 -1.449706
C -6.640022 2.696453 -2.599982
O -1.179463 -2.718978 1.290517
H -1.194995 -2.098937 -0.692227
O -3.897760 -0.924421 3.012355
H -2.813627 -3.299095 2.349999
O 1.929329 0.681658 0.474321
O -4.747137 -0.410981 -0.015786
O -4.321079 0.856894 -1.851269
O 3.606755 -0.218974 -0.783175
O 5.557509 3.457472 -0.995673
O 6.856078 1.026674 -1.866834
O 2.925795 2.599304 -1.225315
O 4.429575 -1.117171 -3.331698
H 2.730743 -1.768487 0.877971
H 1.966447 0.082359 2.451271
H -0.151765 1.192905 1.872635
H -0.336077 -0.378268 2.641697
H 1.133413 -1.694934 -0.846721
H 0.895926 -3.240131 -0.042647
H -4.501477 -2.639972 0.751498
H -3.205964 -3.127305 -0.356682
H 0.614262 -2.413573 2.994881
H 1.748485 -3.565325 2.292042
H 2.362197 -2.191966 3.230076
H 0.207053 0.239484 -1.170251
H -1.538362 0.358768 -1.436876
H -0.700431 1.557944 -0.445579
H -2.354040 0.241541 3.929544
H -2.343698 -1.558057 4.353665
H -3.017334 -0.846965 -1.048902
H -2.231281 1.735820 1.955862
H -2.907043 1.810770 0.322029
H -3.914242 1.278771 1.678343
H -6.977196 0.455947 0.080853
H -8.141710 2.082917 -1.137298
H -7.277755 2.500811 -3.472158
H -6.768489 3.761463 -2.367892
H -5.602064 2.500293 -2.861257
H 3.856648 0.403712 1.183762
H 3.556107 2.775790 0.756332
H 5.593109 -0.535985 -0.209336
H 4.900960 1.342158 -2.529037
H 5.783921 1.944351 0.333486
H 4.896025 3.743460 -1.642364
H 7.153902 1.927942 -2.051047
H 1.996366 2.573004 -0.961208

H 4.843402 -2.324340 -1.675356
H 6.214190 -1.459418 -2.415869
H 3.504198 -0.999148 -3.079668

3d-c70 , delta G = 0.9064 kcal/mol, population = 6.60 %
C -1.556196 -1.289584 1.581605
C -3.073471 -1.503466 1.519331
C -3.513351 -0.855569 0.191038
C -2.235609 -0.164289 -0.408677
C -1.453497 -1.215333 -1.291354
C -0.052224 -0.690821 -1.731708
C 1.119046 -1.287985 -0.960898
C 1.199488 -2.802861 -1.137372
C -0.214765 -3.440354 -1.088085
C -1.270332 -2.528135 -0.480326
C -1.421388 0.035445 0.863496
C -0.390170 1.032312 1.122761
C -2.558293 1.122020 -1.159415
C -2.259707 -1.520642 -2.565494
C 2.153638 -3.428322 -0.120815
C 2.791082 0.408308 -0.813467
C 3.937282 1.024998 -1.616002
C 4.531679 2.188358 -0.827051
C 4.888156 1.772319 0.602252
C 3.663191 1.150953 1.269348
C 3.944473 0.618415 2.661477
C -5.538320 0.311590 -0.370791
C -6.480515 1.376338 0.008004
C -6.329284 2.368610 0.899548
C -5.183832 2.706886 1.789250
O -0.831476 -2.300881 0.876480
H -2.235162 -3.062413 -0.458454
O -1.715096 1.185437 1.683005
H -1.151937 -1.273783 2.597740
O 2.360383 -0.731120 -1.478708
O -4.527472 0.134932 0.507169
O -5.685414 -0.361484 -1.379399
O 3.234836 0.027074 0.490393
O 5.662606 2.755655 -1.486634
O 5.283284 2.906243 1.371196
O 4.988247 0.090608 -1.846920
O 2.776006 0.047767 3.251799
H 1.608549 -2.965120 -2.145161
H 1.059398 -1.046270 0.104614
H 0.102310 -0.947854 -2.787938
H -0.005205 0.400615 -1.673959
H -0.543103 -3.723787 -2.092945
H -0.183283 -4.365419 -0.501005
H -3.542678 -0.969981 2.351329
H -3.346800 -2.560099 1.589777
H 2.263973 -4.503244 -0.300814
H 3.145575 -2.968271 -0.178841
H 1.767709 -3.290834 0.895760
H -1.768676 -2.306298 -3.147162
H -3.282522 -1.845611 -2.354738
H -2.310938 -0.631308 -3.200405
H -0.143665 1.764371 0.352598
H 0.397374 0.817989 1.847375
H -3.955001 -1.570678 -0.502780
H -3.358666 0.965850 -1.886910
H -2.880233 1.896071 -0.458682
H -1.687384 1.501280 -1.700470
H -7.397796 1.335795 -0.573700
H -7.178815 3.048370 0.981737
H -4.272391 2.157815 1.561589
H -4.990634 3.784781 1.738358
H -5.462072 2.498437 2.831838
H 1.980185 1.157473 -0.706804
H 3.531103 1.399557 -2.570099
H 2.853143 1.902108 1.321155
H 5.694431 1.019807 0.571716
H 3.781246 2.988969 -0.767668
H 6.241598 2.019853 -1.733917
H 5.958684 3.370191 0.857326
H 4.613313 -0.662770 -2.321533
H 4.261497 1.439846 3.309434
H 4.760059 -0.120230 2.605013
H 2.482409 -0.661038 2.663511

3d-c78 , delta G = 1.0507 kcal/mol, population = 5.17 %
C -2.695397 -2.313474 1.193063
C -3.536345 -2.166540 -0.082166
C -3.380494 -0.688280 -0.496842
C -2.531660 -0.001469 0.635075
C -0.994659 -0.174726 0.291107
C -0.128887 0.235080 1.509262

C 1.353525 -0.116212 1.420776
C 1.586425 -1.597706 1.109234
C 0.758232 -2.020051 -0.111471
C -0.725081 -1.666169 -0.042600
C -2.854733 -0.937502 1.795613
C -2.848971 -0.643255 3.221925
C -2.918840 1.450860 0.885307
C -0.645180 0.693534 -0.930970
C 1.355161 -2.450830 2.362009
C 3.323095 0.727631 0.297641
C 3.804208 2.141329 -0.046362
C 5.285966 2.114406 -0.403540
C 5.585743 1.053522 -1.463347
C 5.045924 -0.294831 -0.992191
C 5.217833 -1.394313 -2.023233
C -4.944757 0.849600 -1.476102
C -6.301583 1.416274 -1.417837
C -7.205835 1.353119 -0.427700
C -7.134361 0.695094 0.906877
O -1.311199 -2.559602 0.926732
H -1.163698 -1.882925 -1.031787
O -4.098806 -0.734260 2.500198
H -3.030663 -3.119063 1.852576
O 1.941714 0.771658 0.433494
O -4.712916 -0.113646 -0.558402
O -4.121712 1.208509 -2.304100
O 3.635502 -0.176795 -0.766222
O 5.757971 3.396282 -0.818440
O 6.992047 0.924417 -1.661758
O 3.106251 2.671169 -1.170979
O 4.703154 -2.641114 -1.556394
H 2.643307 -1.718863 0.838110
H 1.821557 0.121320 2.390718
H -0.213176 1.313129 1.682968
H -0.503810 -0.261310 2.408515
H 1.180361 -1.543671 -1.003625
H 0.841829 -3.103308 -0.264035
H -4.584347 -2.362115 0.163556
H -3.232275 -2.863902 -0.867884
H 1.502887 -3.510109 2.127352
H 2.070227 -2.173978 3.145371
H 0.342742 -2.341424 2.756859
H -0.593462 1.747940 -0.644086
H 0.329254 0.423846 -1.339181
H -1.388253 0.600360 -1.727423
H -2.598636 0.360472 3.566784
H -2.666845 -1.450614 3.932496
H -2.924763 -0.573770 -1.480187
H -3.951983 1.513416 1.235427
H -2.280607 1.902878 1.649077
H -2.828717 2.046309 -0.026256
H -6.543216 1.992016 -2.307502
H -8.142909 1.876571 -0.623415
H -7.784868 -0.190848 0.909474
H -6.130975 0.381400 1.188587
H -7.540045 1.371180 1.668199
H 3.816061 0.383483 1.228763
H 3.653520 2.779758 0.839926
H 5.553272 -0.586680 -0.054182
H 5.081411 1.325450 -2.406360
H 5.858039 1.859766 0.499700
H 5.131011 3.726208 -1.478623
H 7.336596 1.817179 -1.802524
H 2.163575 2.667567 -0.956659
H 6.282276 -1.542720 -2.223622
H 4.723771 -1.087222 -2.959219
H 3.771023 -2.496985 -1.344936

3d-c9 , delta G = 1.4701 kcal/mol, population = 2.54 %
C -2.596909 -2.526027 1.588634
C -3.549324 -2.403679 0.391680
C -3.476265 -0.920408 -0.029486
C -2.547955 -0.210773 1.023441
C -1.043417 -0.341457 0.542804
C -0.085901 0.090784 1.680270
C 1.392119 -0.216052 1.459837
C 1.639591 -1.690173 1.124798
C 0.717625 -2.136734 -0.017154
C -0.763034 -1.825283 0.184531
C -2.739573 -1.155214 2.206368
C -2.608720 -0.860192 3.626589
C -2.957015 1.228542 1.310953
C -0.829293 0.535435 -0.703607
C 1.551391 -2.551243 2.390651
C 3.240043 0.706053 0.204374
C 3.631914 2.139132 -0.169871
C 5.090375 2.181945 -0.611580

C 5.368222 1.123313 -1.675895
C 4.926737 -0.251473 -1.173344
C 5.099149 -1.354469 -2.214584
C -5.174289 0.551894 -0.890570
C -6.553153 0.992314 -0.626334
C -7.219670 1.942521 -1.299505
C -6.771515 2.775570 -2.447919
O -1.234607 -2.732839 1.201882
H -1.281546 -2.056941 -0.761875
O -3.916621 -0.984898 3.024021
H -2.850701 -3.340627 2.273068
O 1.869270 0.685627 0.425677
O -4.821387 -0.382161 0.030793
O -4.433517 0.929258 -1.782458
O 3.530792 -0.196394 -0.869266
O 5.471478 3.478631 -1.071280
O 6.767909 1.062215 -1.969661
O 2.847747 2.630224 -1.254883
O 6.464060 -1.718282 -2.400970
H 2.669189 -1.777366 0.754805
H 1.935897 0.035964 2.385546
H -0.188935 1.164350 1.871077
H -0.362421 -0.425043 2.603894
H 1.044100 -1.647245 -0.941417
H 0.819340 -3.216675 -0.180262
H -4.565414 -2.633345 0.725734
H -3.291870 -3.089752 -0.420295
H 2.332643 -2.258579 3.101854
H 0.580654 -2.468394 2.884896
H 1.700990 -3.606143 2.138115
H 0.114922 0.300851 -1.196513
H -1.634419 0.415060 -1.433973
H -0.790487 1.591784 -0.421349
H -2.353872 0.150346 3.947687
H -2.339333 -1.661214 4.316436
H -3.113794 -0.789641 -1.049114
H -3.950865 1.252566 1.763861
H -2.262029 1.707436 2.005893
H -2.983945 1.823528 0.394637
H -7.044694 0.490160 0.202113
H -8.236955 2.139328 -0.958298
H -6.887646 3.836930 -2.193581
H -5.742265 2.578959 -2.741198
H -7.434348 2.598593 -3.305228
H 3.804650 0.392826 1.105625
H 3.500306 2.773487 0.722226
H 5.508128 -0.516992 -0.270711
H 4.794958 1.359528 -2.587862
H 5.727180 1.967413 0.257811
H 4.778390 3.773880 -1.679810
H 7.042356 1.948667 -2.241446
H 1.920556 2.594127 -0.984077
H 4.632232 -1.035973 -3.161109
H 4.575486 -2.249410 -1.860931
H 6.947376 -0.902725 -2.599720

3e

3e-c13 , delta G = 0.0000 kcal/mol, population = 67.89 %

C 1.663072 -1.688622 -1.974615
C 3.131234 -1.916776 -1.587869
C 3.371217 -1.010071 -0.360271
C 2.039649 -0.201527 -0.148235
C 1.071791 -1.059928 0.765174
C -0.344548 -0.440108 0.762358
C -1.398061 -1.326044 1.423323
C -1.491141 -2.696666 0.757133
C -0.104475 -3.347194 0.810917
C 0.998333 -2.499059 0.192197
C 1.486641 -0.244208 -1.571905
C 0.586181 0.697484 -2.223603
C 2.264525 1.210254 0.377433
C 1.604627 -1.104875 2.206424
C -2.532362 -3.593009 1.423990
C -3.178294 -0.095279 0.308589
C -4.700227 -0.254163 0.275853
C -5.268804 0.503249 -0.929186
C -4.784442 1.961172 -0.949912
C -3.256592 1.982602 -0.845768
C -2.679876 3.383052 -0.743959
C 5.304182 0.245875 0.325438
C 6.316972 1.174473 -0.200084
C 7.273681 1.784040 0.516555
C 7.561638 1.695249 1.973509
O 0.749674 -2.502446 -1.227170
H 1.958517 -3.005459 0.386423
O 2.002176 0.708059 -2.523081
H 1.450222 -1.875788 -3.030756

O -2.697154 -0.669291 1.481521
O 4.468272 -0.119373 -0.682073
O 5.197802 -0.166173 1.468005
O -2.829458 1.291051 0.337221
O -1.251937 3.361681 -0.677840
O -5.416283 2.727236 0.077742
O -6.685043 0.417352 -0.988025
O -5.294880 0.275847 1.463973
H -1.760885 -2.555037 -0.299860
H -1.162212 -1.470455 2.485746
H -0.327871 0.527572 1.274383
H -0.654719 -0.255618 -0.270136
H 0.154132 -3.563656 1.855450
H -0.127245 -4.311995 0.289823
H 3.764901 -1.578074 -2.412787
H 3.357909 -2.968627 -1.391588
H -2.554896 -4.577577 0.944489
H -3.534199 -3.158784 1.360596
H -2.291846 -3.738888 2.484657
H 1.033818 -1.803724 2.823037
H 2.653228 -1.411873 2.251858
H 1.523151 -0.118912 2.673235
H 0.227096 1.573735 -1.683792
H -0.052051 0.344576 -3.035428
H 3.653130 -1.572396 0.530506
H 1.314163 1.722455 0.552387
H 2.822872 1.203657 1.316682
H 2.828431 1.796544 -0.351660
H 6.250696 1.371047 -1.266418
H 7.936852 2.444642 -0.043601
H 6.859473 1.059384 2.508943
H 8.581260 1.314771 2.119527
H 7.553551 2.703602 2.406843
H -2.750750 -0.563821 -0.597251
H -2.834334 1.479450 -1.735893
H -5.084348 2.420615 -1.900686
H -4.889011 0.007236 -1.832844
H -4.937119 -1.325546 0.174676
H -2.949062 3.951584 -1.640193
H -3.105583 3.890495 0.132183
H -1.019161 2.856696 0.113032
H -5.229975 2.272820 0.916359
H -7.028255 1.011448 -0.304979
H -4.836458 -0.123039 2.216662

3e-c20 , delta G = 0.5111 kcal/mol, population = 28.62 %

C 1.852941 -1.541283 -1.839424
C 3.282777 -1.850142 -1.372855
C 3.446152 -1.087901 -0.039282
C 2.116866 -0.276330 0.165459
C 1.066438 -1.205511 0.900822
C -0.333159 -0.550190 0.862078
C -1.452307 -1.473814 1.336651
C -1.520654 -2.758716 0.514621
C -0.155298 -3.449886 0.600296
C 1.008719 -2.569894 0.165809
C 1.673715 -0.146171 -1.291051
C 0.847568 0.888658 -1.897738
C 2.323745 1.060459 0.866451
C 1.486269 -1.425259 2.363048
C -2.628713 -3.692345 0.995864
C -3.117794 -0.084517 0.231988
C -4.633933 -0.210032 0.066275
C -5.101898 0.683845 -1.086507
C -4.600512 2.125140 -0.912917
C -3.084733 2.105296 -0.693352
C -2.502106 3.476426 -0.404331
C 5.340531 0.052168 0.897154
C 6.421133 1.023857 0.667972
C 6.567294 1.914262 -0.325974
C 5.705857 2.198149 -1.507178
O 0.870736 -2.408935 -1.259301
H 1.941587 -3.120130 0.373475
O 2.282508 0.888764 -2.090311
H 1.718767 -1.604412 -2.922913
O -2.737627 -0.789866 1.369825
O 4.575077 -0.186770 -0.189848
O 5.179437 -0.502606 1.973447
O -2.756484 1.284087 0.437256
O -1.081613 3.425737 -0.248897
O -5.299539 2.790178 0.141188
O -6.511194 0.632653 -1.255727
O -5.312626 0.198093 1.257589
H -1.702991 -2.491066 -0.536482
H -1.303718 -1.742763 2.390506
H -0.339918 0.354471 1.478823
H -0.555705 -0.242742 -0.163551

H 0.016011 -3.787512 1.630598
H -0.155860 -4.350602 -0.025595
H 3.984535 -1.442812 -2.106670
H 3.473049 -2.922951 -1.277533
H -3.612730 -3.222829 0.909323
H -2.473128 -3.963324 2.047752
H -2.637126 -4.615816 0.406804
H 1.412210 -0.489064 2.923553
H 0.839064 -2.152622 2.859044
H 2.513502 -1.790077 2.449795
H 0.468341 1.711760 -1.291805
H 0.264906 0.647039 -2.788188
H 3.660816 -1.747856 0.801524
H 2.925787 1.724303 0.241544
H 1.369160 1.558744 1.057425
H 2.836481 0.933595 1.822923
H 7.158473 1.010543 1.466372
H 7.456148 2.543199 -0.255232
H 5.581303 3.282142 -1.613397
H 6.219984 1.862073 -2.418580
H 4.730205 1.717476 -1.466821
H -2.624517 -0.460790 -0.683398
H -2.601950 1.691132 -1.598618
H -4.821580 2.689353 -1.828232
H -4.664201 0.279868 -2.009583
H -4.872558 -1.259572 -0.169770
H -2.706700 4.140335 -1.250658
H -2.979642 3.896267 0.491279
H -0.905173 2.846318 0.504486
H -5.183384 2.246002 0.937859
H -6.896870 1.146130 -0.531268
H -4.913651 -0.285727 1.994214

3e-c27 , delta G = 1.7575 kcal/mol, population = 3.48 %
C 1.639959 -1.074517 -2.069402
C 3.100493 -1.456976 -1.784129
C 3.406921 -0.865000 -0.390028
C 2.131587 -0.044769 0.023507
C 1.118417 -1.022177 0.743964
C -0.276950 -0.367840 0.848632
C -1.343950 -1.317055 1.390273
C -1.513208 -2.555482 0.516404
C -0.141027 -3.226435 0.356634
C 0.976043 -2.305506 -0.112785
C 1.561419 0.257546 -1.358799
C 0.728064 1.383017 -1.757345
C 2.450255 1.197008 0.845515
C 1.643233 -1.378973 2.143515
C -2.502855 -3.558982 1.111367
C -3.490316 -0.392568 0.595977
C -4.722123 0.261672 1.232295
C -5.732107 0.632635 0.144705
C -5.062859 1.454882 -0.963240
C -3.828920 0.701746 -1.474193
C -3.082438 1.420005 -2.588813
C 5.430231 0.071590 0.513491
C 6.497827 1.029952 0.187029
C 7.519378 1.374304 0.985895
C 7.841698 0.904490 2.360312
O 0.699379 -1.991266 -1.491673
H 1.916546 -2.878389 -0.058306
O 2.136004 1.358558 -2.091740
H 1.398662 -1.014978 -3.134320
O -2.585577 -0.590281 1.633744
O 4.557241 0.006761 -0.525475
O 5.310993 -0.586575 1.533047
O -2.917462 0.464382 -0.393916
O -2.466721 2.641830 -2.194301
O -4.740708 2.778737 -0.505285
O -6.875077 1.283078 0.681337
O -4.356427 1.461941 1.923379
H -1.852257 -2.239494 -0.481194
H -1.073421 -1.645969 2.402743
H -0.234313 0.511842 1.500702
H -0.604282 -0.030283 -0.135504
H 0.154526 -3.669461 1.315786
H -0.224726 -4.057154 -0.354744
H 3.738164 -0.969826 -2.527659
H 3.272916 -2.535667 -1.838481
H -3.498462 -3.130844 1.259460
H -2.143975 -3.908008 2.087418
H -2.603578 -4.432171 0.457727
H 2.682399 -1.719903 2.119454
H 1.591419 -0.506292 2.801650
H 1.051269 -2.171412 2.607035
H 0.448022 2.133705 -1.018576
H 0.053086 1.279711 -2.606316

H 3.655611 -1.627573 0.349070
H 3.053832 0.950388 1.722516
H 3.006591 1.915174 0.238463
H 1.535068 1.685876 1.190247
H 6.418814 1.487818 -0.794916
H 8.216566 2.105635 0.574766
H 7.111646 0.198796 2.751446
H 8.836022 0.438750 2.360755
H 7.916122 1.769712 3.031585
H -3.790882 -1.338255 0.108611
H -4.175559 -0.263275 -1.891408
H -5.770812 1.583642 -1.791743
H -6.092595 -0.303064 -0.303058
H -5.179601 -0.456829 1.930580
H -2.282170 0.766725 -2.954934
H -3.794156 1.577012 -3.416472
H -3.139768 3.139689 -1.703396
H -4.254184 2.665793 0.331505
H -6.593491 2.162522 0.970106
H -3.576463 1.254239 2.458163

3f

3f-c67 , delta G = 0.0000 kcal/mol, population = 41.26 %

C 2.513796 -2.647005 -1.323960
C 3.843569 -2.323701 -0.626904
C 3.652281 -0.911028 -0.032107
C 2.248161 -0.413979 -0.533707
C 1.147859 -0.904277 0.491218
C -0.264515 -0.742484 -0.113251
C -1.362805 -1.351886 0.752084
C -1.141503 -2.844676 0.964120
C 0.249194 -3.058337 1.571047
C 1.382406 -2.407716 0.787829
C 2.103307 -1.268735 -1.788420
C 1.325127 -0.980283 -2.984174
C 2.196299 1.085922 -0.792807
C 1.247649 -0.088375 1.790356
C -2.218601 -3.464486 1.851719
C -3.480439 -0.225327 0.626137
C -4.847621 -0.376613 -0.040909
C -5.761493 0.746575 0.434236
C -5.105528 2.112000 0.234999
C -3.735155 2.133920 0.912225
C -2.956608 3.415092 0.666417
C 5.215908 0.888454 0.279817
C 6.250538 1.651558 -0.435144
C 6.918006 2.711629 0.046060
C 6.799771 3.376731 1.371666
O 1.524041 -3.169728 -0.427712
H 2.302522 -2.515311 1.385901
O 2.762438 -0.821242 -2.991067
H 2.606153 -3.371394 -2.137928
O -2.633731 -1.173799 0.073379
O 4.711391 -0.065651 -0.546451
O 4.845406 1.043150 1.431125
O -2.926868 1.071502 0.387632
O -7.039420 0.699239 -0.201498
O -5.894780 3.139717 0.832071
O -4.761748 -0.282588 -1.460393
O -2.722873 3.653624 -0.720816
H -1.170314 -3.320774 -0.027061
H -1.422365 -0.847090 1.728617
H -0.489189 0.316306 -0.273532
H -0.318318 -1.244713 -1.084413
H 0.259391 -2.667397 2.596619
H 0.458000 -4.132361 1.647628
H 4.633869 -2.290892 -1.382700
H 4.117903 -3.069609 0.124507
H -2.043832 -4.538123 1.980383
H -3.214958 -3.330542 1.420012
H -2.210950 -3.000379 2.846277
H 2.274411 -0.032587 2.163457
H 0.896133 0.934199 1.621720
H 0.632600 -0.516589 2.585619
H 0.765507 -0.046590 -3.049304
H 0.948742 -1.805607 -3.589863
H 3.721701 -0.899998 1.056314
H 2.845302 1.345034 -1.632730
H 1.181227 1.410857 -1.037055
H 2.529092 1.648645 0.082845
H 6.462447 1.307186 -1.443418
H 7.651944 3.155599 -0.627807
H 6.619574 4.449223 1.225681
H 6.014248 2.951003 1.992718
H 7.759082 3.302896 1.901155
H -3.588619 -0.366314 1.721171
H -5.271069 -1.348845 0.261294

H -3.870915 2.009388 2.002656
H -4.971383 2.299856 -0.841978
H -5.953456 0.610685 1.507711
H -6.876892 0.623934 -1.153127
H -6.793300 3.034317 0.490880
H -4.154993 -0.970706 -1.763249
H -2.007221 3.358653 1.221344
H -3.530342 4.266020 1.044495
H -2.268165 2.875237 -1.069299

3f-c88 , delta G = 0.3635 kcal/mol, population = 22.32 %

C 2.465682 -2.648515 -1.382363
C 3.793021 -2.313531 -0.685858
C 3.584733 -0.908528 -0.078544
C 2.171991 -0.426992 -0.570105
C 1.082101 -0.943678 0.452576
C -0.333177 -0.798285 -0.148578
C -1.419003 -1.419333 0.724775
C -1.183043 -2.911897 0.922835
C 0.215465 -3.117977 1.515593
C 1.338124 -2.445643 0.735260
C 2.034846 -1.271340 -1.832479
C 1.247932 -0.981465 -3.021905
C 2.096372 1.074936 -0.811965
C 1.173374 -0.135571 1.757497
C -2.244140 -3.550239 1.816771
C -3.493176 -0.224854 0.576408
C -4.880660 -0.347009 -0.054721
C -5.722872 0.849590 0.371934
C -5.001476 2.171619 0.099985
C -3.613909 2.144550 0.745915
C -2.805594 3.388354 0.420472
C 5.126223 0.910203 0.237942
C 6.140074 1.697148 -0.481414
C 6.796901 2.762804 0.001875
C 6.684702 3.414003 1.334976
O 1.486732 -3.194408 -0.488029
H 2.261060 -2.547219 1.330192
O 2.683416 -0.803945 -3.033386
H 2.565892 -3.363444 -2.203788
O -2.704810 -1.249814 0.072588
O 4.629120 -0.044164 -0.592034
O 4.765691 1.048393 1.394548
O -2.887266 1.022465 0.240712
O -7.011995 0.843643 -0.241729
O -5.734283 3.259509 0.661265
O -4.822377 -0.340390 -1.478665
O -1.534474 3.410492 1.073340
H -1.217036 -3.380973 -0.071663
H -1.469016 -0.922908 1.706582
H -0.569570 0.256526 -0.310612
H -0.384588 -1.305240 -1.117558
H 0.228697 -2.740669 2.546230
H 0.435703 -4.190764 1.576680
H 4.581074 -2.264256 -1.443127
H 4.078769 -3.062051 0.058718
H -2.232713 -3.091639 2.813870
H -2.053863 -4.622219 1.937951
H -3.246133 -3.426866 1.395575
H 0.577259 -0.584846 2.555593
H 2.202265 -0.057462 2.120981
H 0.794652 0.879142 1.599591
H 0.676164 -0.054406 -3.075137
H 0.879597 -1.805614 -3.634149
H 3.659221 -0.904937 1.009613
H 2.732278 1.352938 -1.655968
H 1.074094 1.388981 -1.039710
H 2.430373 1.632441 0.066593
H 6.344323 1.366551 -1.495869
H 7.514175 3.225651 -0.677292
H 6.453802 4.478525 1.200426
H 5.932917 2.952592 1.972080
H 7.659549 3.379912 1.839147
H -3.585491 -0.292701 1.680457
H -5.349247 -1.275364 0.312281
H -3.732978 2.055505 1.843106
H -4.880856 2.303140 -0.989080
H -5.903121 0.780422 1.453810
H -6.871636 0.704957 -1.189746
H -6.649339 3.159891 0.363922
H -4.229429 -1.053435 -1.750064
H -3.400176 4.276409 0.672094
H -2.598299 3.402452 -0.655743
H -1.688163 3.534066 2.018139

3f-c63 , delta G = 0.6796 kcal/mol, population = 13.08 %

C 2.498105 -2.616147 -1.398254
C 3.808904 -2.300059 -0.663005
C 3.598693 -0.897758 -0.049443
C 2.199235 -0.402666 -0.564697
C 1.082631 -0.924145 0.427045
C -0.317155 -0.751061 -0.201179
C -1.434109 -1.395349 0.613622
C -1.206746 -2.891006 0.791875
C 0.169263 -3.108108 1.431452
C 1.317645 -2.433326 0.691780
C 2.087147 -1.230833 -1.840944
C 1.332673 -0.922627 -3.047105
C 2.138709 1.102130 -0.794254
C 1.152540 -0.143110 1.748682
C -2.302299 -3.541042 1.634295
C -3.549450 -0.276315 0.457892
C -4.855294 -0.315669 -0.337664
C -5.776123 0.796934 0.154408
C -5.062387 2.151218 0.169284
C -3.766461 2.036552 0.968449
C -2.939016 3.307984 0.941652
C 5.142330 0.914916 0.296519
C 6.171916 1.697324 -0.404902
C 6.801461 2.779328 0.078362
C 6.637353 3.455328 1.393587
O 1.491467 -3.165403 -0.537436
H 2.224379 -2.550194 1.308392
O 2.768174 -0.752406 -3.019223
H 2.614284 -3.321256 -2.225975
O -2.683056 -1.202132 -0.103645
O 4.657265 -0.038537 -0.541654
O 4.760785 1.055637 1.446018
O -2.948471 1.017923 0.378093
O -6.979724 0.869349 -0.609107
O -5.878944 3.141396 0.790788
O -4.634419 -0.097892 -1.728667
O -1.728708 3.164594 1.684686
H -1.204992 -3.343050 -0.210906
H -1.530244 -0.919772 1.601740
H -0.545197 0.311226 -0.330435
H -0.346154 -1.218519 -1.190574
H 0.149764 -2.739322 2.465017
H 0.383608 -4.182168 1.490195
H 4.618273 -2.252635 -1.397434
H 4.066713 -3.057702 0.082519
H -2.323428 -3.103147 2.640468
H -2.123899 -4.616646 1.739381
H -3.287897 -3.401482 1.180071
H 0.490785 -0.570143 2.506231
H 2.163347 -0.128690 2.166717
H 0.841008 0.894516 1.593126
H 0.767151 0.008055 -3.104371
H 0.975879 -1.738015 -3.677680
H 3.654531 -0.900490 1.039947
H 1.124474 1.423924 -1.045870
H 2.455758 1.650991 0.095885
H 2.796330 1.382422 -1.620624
H 6.410948 1.350837 -1.406374
H 7.536517 3.236198 -0.585628
H 5.844027 3.021449 1.998990
H 7.583580 3.403577 1.948723
H 6.442394 4.522935 1.231957
H -3.755948 -0.505011 1.523602
H -5.337807 -1.292481 -0.167304
H -4.001671 1.769414 2.014871
H -4.814398 2.443203 -0.865597
H -6.083928 0.565779 1.183699
H -6.721151 0.872943 -1.542236
H -6.750168 3.085350 0.374318
H -4.000699 -0.761691 -2.031176
H -3.504534 4.121641 1.403583
H -2.730595 3.576095 -0.106783
H -1.260033 2.406323 1.310562

3f-c75 , delta G = 0.8504 kcal/mol, population = 9.81 %

C 2.334698 -2.318915 -1.610693
C 3.706912 -2.111500 -0.953165
C 3.553087 -0.839599 -0.089563
C 2.110417 -0.288218 -0.381331
C 1.093272 -0.994061 0.605135
C -0.361669 -0.733651 0.154100
C -1.391903 -1.531409 0.949919
C -1.151045 -3.029929 0.826031
C 0.278012 -3.335650 1.286060
C 1.352371 -2.521957 0.576352
C 1.883139 -0.885303 -1.766960
C 1.025041 -0.386421 -2.831546

C 2.023740 1.231985 -0.341421
C 1.291681 -0.457627 2.032252
C -2.159084 -3.849604 1.629985
C -3.244791 -0.010832 0.700873
C -4.750405 -0.141863 0.950807
C -5.378037 1.244111 1.050902
C -4.998345 2.114881 -0.147524
C -3.478305 2.142250 -0.287533
C -3.008221 2.907920 -1.509600
C 5.118257 0.916473 0.416901
C 6.079085 1.820248 -0.234240
C 6.787078 2.786081 0.371157
C 6.798193 3.191327 1.802653
O 1.411522 -3.025680 -0.772327
H 2.312181 -2.729999 1.077830
O 2.456794 -0.200322 -2.899789
H 2.375928 -2.867723 -2.555627
O -2.730901 -1.279332 0.456422
O 4.560994 0.107386 -0.522340
O 4.839546 0.859495 1.602493
O -2.997824 0.801194 -0.449936
O -6.795573 1.178772 1.205016
O -5.448898 3.454866 0.041105
O -5.404688 -0.828746 -0.113203
O -1.583757 2.910123 -1.609766
H -1.241582 -3.288641 -0.239118
H -1.371253 -1.248091 2.015457
H -0.586740 0.334402 0.240089
H -0.492823 -1.010351 -0.897107
H 0.356382 -3.160401 2.366740
H 0.495172 -4.398982 1.128589
H 4.443220 -1.922434 -1.739895
H 4.036197 -2.981677 -0.378207
H -2.112145 -3.580831 2.692966
H -1.938765 -4.919163 1.543490
H -3.181742 -3.682334 1.281066
H 0.732379 -1.044906 2.764530
H 2.342542 -0.470667 2.334966
H 0.935914 0.574798 2.102085
H 0.458199 0.532577 -2.679896
H 0.612861 -1.085785 -3.559886
H 3.709798 -1.028525 0.973169
H 2.379290 1.625412 0.614078
H 2.633537 1.664327 -1.138108
H 0.994720 1.573520 -0.484720
H 6.195930 1.672563 -1.304103
H 7.450550 3.362416 -0.275040
H 6.101034 2.620724 2.412930
H 7.813441 3.075028 2.204412
H 6.566888 4.261562 1.878672
H -2.766397 0.454672 1.585670
H -4.893291 -0.677139 1.904211
H -3.035297 2.591629 0.620444
H -5.432300 1.682634 -1.065404
H -4.996675 1.733493 1.958035
H -7.123814 0.573183 0.524410
H -6.383828 3.402954 0.283189
H -5.011331 -1.708856 -0.178850
H -3.326029 3.951070 -1.433262
H -3.467235 2.464776 -2.408096
H -1.306844 1.983858 -1.602907

3f-c11 , delta G = 1.2456 kcal/mol, population = 5.03 %

C 2.580509 -2.648331 -1.355207
C 3.895185 -2.324440 -0.630414
C 3.685738 -0.917465 -0.028136
C 2.289380 -0.422878 -0.552803
C 1.171165 -0.926398 0.446039
C -0.229741 -0.768829 -0.186161
C -1.341141 -1.389208 0.654019
C -1.115893 -2.881663 0.864598
C 0.264843 -3.092376 1.494669
C 1.408314 -2.430315 0.736680
C 2.172698 -1.268097 -1.816886
C 1.416070 -0.973022 -3.024700
C 2.235973 1.078977 -0.800278
C 1.240690 -0.119051 1.752367
C -2.205467 -3.510053 1.730881
C -3.465925 -0.279978 0.487149
C -4.826527 -0.464739 -0.187008
C -5.766851 0.646992 0.265019
C -5.118930 2.016046 0.070251
C -3.760855 2.063316 0.773361
C -3.014078 3.374248 0.540436
C 5.238433 0.883966 0.328010
C 6.283578 1.655858 -0.361816
C 6.942858 2.710562 0.141954

C 6.803513 3.360926 1.472899
O 1.576545 -3.182560 -0.481672
H 2.317850 -2.537323 1.350742
O 2.852749 -0.808322 -3.003184
H 2.691595 -3.365958 -2.172833
O -2.599672 -1.218337 -0.049963
O 4.750477 -0.063286 -0.515831
O 4.847152 1.027939 1.473888
O -2.933725 1.025831 0.238529
O -7.028434 0.583084 -0.399706
O -5.942440 3.045165 0.628237
O -4.733839 -0.386256 -1.607191
O -3.576992 4.458421 1.273977
H -1.125787 -3.353883 -0.128839
H -1.425361 -0.888599 1.630884
H -0.457770 0.289094 -0.346714
H -0.261173 -1.267259 -1.160288
H 0.254848 -2.708961 2.523026
H 0.477666 -4.165916 1.567197
H 4.699555 -2.281853 -1.370720
H 4.158919 -3.075119 0.120109
H -2.028533 -4.583612 1.857473
H -3.194953 -3.378112 1.282987
H -2.216297 -3.050724 2.727624
H 2.259617 -0.062031 2.146329
H 0.888656 0.903312 1.583726
H 0.611480 -0.554741 2.532379
H 0.853906 -0.041026 -3.092255
H 1.054534 -1.794942 -3.643931
H 3.734745 -0.914562 1.061467
H 2.554572 1.635269 0.084812
H 2.896194 1.347195 -1.628547
H 1.223334 1.402700 -1.055594
H 6.512165 1.322732 -1.370213
H 7.687523 3.161916 -0.515058
H 6.006985 2.929864 2.075991
H 7.753708 3.279374 2.017544
H 6.627696 4.435363 1.336127
H -3.580121 -0.411064 1.582698
H -5.233914 -1.440919 0.124132
H -3.905343 1.920318 1.860256
H -4.958365 2.194025 -1.006114
H -5.978422 0.516168 1.335114
H -6.843055 0.469415 -1.343431
H -6.811056 2.974773 0.209051
H -4.105431 -1.060124 -1.897876
H -2.987231 3.588056 -0.540708
H -1.982773 3.253464 0.889816
H -4.520106 4.485629 1.054656

3f-c12 , delta G = 1.2647 kcal/mol, population = 4.87 %

C 1.465934 -0.954243 -1.914697
C 2.961695 -1.253291 -1.733944
C 3.356246 -0.554233 -0.412926
C 2.081859 0.237192 0.056556
C 1.178237 -0.732260 0.923493
C -0.236242 -0.132156 1.085779
C -1.224109 -1.086710 1.747117
C -1.365203 -2.400281 0.973883
C 0.022348 -3.021673 0.798423
C 1.040018 -2.079228 0.171552
C 1.384376 0.419767 -1.287937
C 0.463916 1.476158 -1.685203
C 2.410887 1.542598 0.768556
C 1.822732 -0.954175 2.299344
C -2.314074 -3.373351 1.669319
C -3.007468 0.418810 1.002174
C -4.388324 0.887068 1.467437
C -5.014338 1.747797 0.372944
C -4.985028 1.047204 -0.989174
C -3.557689 0.603294 -1.300621
C -3.438683 -0.199514 -2.582216
C 5.408171 0.519843 0.239448
C 6.398534 1.494324 -0.244517
C 7.492743 1.899992 0.417906
C 7.987296 1.492114 1.760474
O 0.623787 -1.869574 -1.195146
H 2.014354 -2.593173 0.173248
O 1.837964 1.491364 -2.139379
H 1.130373 -0.979773 -2.955109
O -2.524082 -0.458641 1.967254
O 4.445962 0.355082 -0.706148
O 5.410596 -0.073100 1.304783
O -3.111551 -0.259536 -0.249161
O -6.344069 2.146752 0.705408
O -5.409867 1.937538 -2.018712
O -5.268142 -0.208565 1.702717

O -2.083193 -0.539244 -2.875547
H -1.762710 -2.164499 -0.018285
H -0.897533 -1.320780 2.769921
H -0.181829 0.793978 1.669783
H -0.626500 0.114909 0.094848
H 0.404696 -3.359980 1.769996
H -0.054264 -3.915766 0.167562
H 3.509826 -0.792100 -2.560762
H 3.182010 -2.324593 -1.733110
H -3.322736 -2.956701 1.746196
H -1.960342 -3.602571 2.682887
H -2.375780 -4.314679 1.112355
H 1.320832 -1.747131 2.858572
H 2.878899 -1.227384 2.216774
H 1.758397 -0.041302 2.899011
H 0.215848 2.262763 -0.971206
H -0.279783 1.269715 -2.454724
H 3.704477 -1.252909 0.349180
H 2.883382 2.241093 0.073671
H 1.507993 2.017657 1.162190
H 3.095053 1.375442 1.604091
H 6.194764 1.905583 -1.229118
H 8.114215 2.633208 -0.097998
H 7.338569 0.769905 2.251961
H 8.996393 1.070879 1.662175
H 8.094940 2.382208 2.393804
H -2.353052 1.302431 0.872448
H -4.264600 1.491629 2.381253
H -2.900935 1.491387 -1.363010
H -5.632660 0.154335 -0.953022
H -4.433325 2.676585 0.286423
H -6.817979 1.347968 0.979116
H -6.245577 2.325342 -1.724376
H -4.825698 -0.806861 2.319700
H -3.803491 0.398556 -3.421767
H -4.064452 -1.102299 -2.495819
H -1.723437 -1.019423 -2.115531

3f-c29 , delta G = 1.7849 kcal/mol, population = 2.02 %
C 1.433870 -0.947016 -1.903947
C 2.932251 -1.241200 -1.736397
C 3.335589 -0.543790 -0.417017
C 2.062530 0.242622 0.064461
C 1.169122 -0.731866 0.936131
C -0.248982 -0.141677 1.105906
C -1.223040 -1.101449 1.780804
C -1.359088 -2.421354 1.016439
C 0.031032 -3.029990 0.820742
C 1.033369 -2.078497 0.183632
C 1.354449 0.425907 -1.274141
C 0.432685 1.483899 -1.663867
C 2.392998 1.547405 0.776911
C 1.823460 -0.950261 2.307823
C -2.286256 -3.401225 1.731659
C -3.023348 0.376936 1.052392
C -4.412434 0.853110 1.473729
C -4.959879 1.774181 0.374352
C -4.911601 1.104343 -1.003488
C -3.504482 0.577888 -1.271019
C -3.386246 -0.227050 -2.550782
C 5.389933 0.533092 0.221612
C 6.374861 1.510558 -0.267296
C 7.471862 1.918398 0.389195
C 7.974964 1.510969 1.728703
O 0.601275 -1.867322 -1.178446
H 2.011075 -2.585525 0.172967
O 1.803049 1.498484 -2.127712
H 1.089496 -0.971875 -2.941357
O -2.529925 -0.491560 2.011011
O 4.420367 0.369283 -0.716623
O 5.402260 -0.063357 1.284940
O -3.148542 -0.312126 -0.203191
O -6.288489 2.199627 0.671590
O -5.249483 2.031759 -2.030955
O -5.310027 -0.232924 1.705563
O -2.039701 -0.619321 -2.815201
H -1.775000 -2.194291 0.029719
H -0.883922 -1.326370 2.801447
H -0.197623 0.788450 1.683987
H -0.646437 0.097188 0.115513
H 0.429145 -3.366469 1.786528
H -0.046290 -3.923871 0.189613
H 3.471662 -0.776168 -2.566777
H 3.156452 -2.311693 -1.740073
H -3.294735 -2.990310 1.834659
H -1.907677 -3.628516 2.736569
H -2.353934 -4.342711 1.175592

H 1.330956 -1.747171 2.869574
H 2.881119 -1.215403 2.218089
H 1.756044 -0.038387 2.908669
H 0.190139 2.269644 -0.946916
H -0.316364 1.278125 -2.428169
H 3.692083 -1.243209 0.340612
H 1.492030 2.017460 1.180865
H 3.086000 1.381058 1.605296
H 2.855930 2.249772 0.079475
H 6.164315 1.922487 -1.250191
H 8.088519 2.653612 -0.129638
H 8.085336 2.401152 2.361425
H 7.330141 0.787842 2.223946
H 8.983968 1.090944 1.624337
H -2.370385 1.256795 0.900687
H -4.344908 1.411854 2.415342
H -2.794955 1.425004 -1.304168
H -5.612579 0.249351 -1.012731
H -4.347008 2.685999 0.332485
H -6.775147 1.405062 0.939731
H -6.072327 2.461547 -1.759132
H -5.112489 -0.919457 1.051302
H -3.707814 0.387440 -3.396407
H -4.050410 -1.103844 -2.482594
H -1.705471 -1.103835 -2.045954

3f-c33 , delta G = 1.9195 kcal/mol, population = 1.61 %

C 2.762483 -2.382013 -1.157797
C 4.005055 -2.091066 -0.302994
C 3.713272 -0.738570 0.384849
C 2.339083 -0.243625 -0.191487
C 1.169406 -0.866459 0.674053
C -0.185989 -0.681237 -0.042667
C -1.340071 -1.414953 0.633035
C -1.076880 -2.912895 0.727632
C 0.255342 -3.140971 1.449356
C 1.430924 -2.384183 0.844874
C 2.345883 -0.977118 -1.528800
C 1.677931 -0.595396 -2.764067
C 2.245961 1.271996 -0.312820
C 1.119042 -0.189236 2.053070
C -2.210779 -3.649352 1.437392
C -3.482189 -0.357032 0.356161
C -4.733145 -0.464409 -0.518600
C -5.750381 0.581773 -0.077331
C -5.109486 1.966767 -0.010633
C -3.867586 1.930487 0.881239
C -3.122665 3.262428 0.923162
C 5.177631 1.090434 0.920389
C 6.216214 2.008983 0.427316
C 6.589309 2.269248 -0.835749
C 6.090101 1.716651 -2.125750
O 1.712239 -3.018290 -0.418544
H 2.295956 -2.522926 1.514470
O 3.103089 -0.396937 -2.612363
H 2.960564 -3.019803 -2.023713
O -2.532427 -1.216891 -0.172636
O 4.779323 0.174566 0.011482
O 4.743881 1.146535 2.060889
O -2.951614 0.972355 0.343503
O -6.902209 0.595985 -0.920091
O -6.026899 2.916509 0.541591
O -4.442428 -0.215191 -1.891548
O -3.812062 4.247268 1.687693
H -0.988763 -3.290649 -0.301646
H -1.535445 -1.010517 1.638018
H -0.438987 0.380873 -0.108722
H -0.128572 -1.071367 -1.063972
H 0.152076 -2.850258 2.502683
H 0.498382 -4.210550 1.446405
H 4.867207 -1.976493 -0.966559
H 4.224461 -2.892912 0.407637
H -2.005679 -4.724335 1.484255
H -3.162841 -3.506217 0.917409
H -2.323487 -3.282437 2.465630
H 0.407181 -0.685859 2.716838
H 2.092237 -0.198730 2.552642
H 0.799732 0.852504 1.954448
H 1.094189 0.325098 -2.797771
H 1.393356 -1.368164 -3.479228
H 3.702322 -0.807804 1.473139
H 1.242974 1.583847 -0.615944
H 2.478623 1.758955 0.637306
H 2.952264 1.634541 -1.063707
H 6.689723 2.562207 1.234211
H 7.379651 3.014274 -0.939762
H 5.166912 1.147903 -2.032529

H 5.952494 2.534561 -2.842487
H 6.857479 1.059919 -2.559210
H -3.738080 -0.623822 1.402096
H -5.160453 -1.472355 -0.387275
H -4.162010 1.649718 1.909324
H -4.800306 2.275988 -1.022950
H -6.110095 0.321937 0.927746
H -6.581488 0.598950 -1.833847
H -6.826058 2.890116 -0.002391
H -3.753935 -0.837203 -2.161404
H -2.939327 3.606189 -0.108217
H -2.152709 3.102523 1.406713
H -4.713206 4.295230 1.335462

3g

3g-c27 , delta G = 0.0000 kcal/mol, population = 45.69 %

C -2.615837 -2.524991 0.709205
C -3.870737 -2.321271 -0.151165
C -3.927515 -0.803205 -0.428343
C -2.745214 -0.165652 0.388698
C -1.436807 -0.204623 -0.500947
C -0.194752 0.140223 0.349480
C 1.121782 -0.037999 -0.399167
C 1.319053 -1.478000 -0.891338
C 0.091588 -1.869041 -1.733605
C -1.265691 -1.634708 -1.076982
C -2.584288 -1.224290 1.476283
C -2.048252 -1.057939 2.819762
C -3.062491 1.224086 0.925957
C -1.562494 0.805802 -1.653936
C 1.637863 -2.459286 0.240102
C 3.287642 0.956527 -0.011799
C 4.053541 1.615668 1.137660
C 5.388993 2.137930 0.623673
C 6.163093 1.042927 -0.107497
C 5.292063 0.445358 -1.212211
C 5.938173 -0.730467 -1.925416
C -5.795358 0.701945 -0.619151
C -7.048976 1.068206 0.057801
C -7.833907 2.111103 -0.253023
C -7.658820 3.137933 -1.315438
O -1.417192 -2.648484 -0.065046
H -2.035151 -1.791974 -1.852322
O -3.474604 -1.171174 2.609765
H -2.659997 -3.408728 1.351958
O 2.153474 0.372630 0.532849
O -5.204799 -0.318319 0.056336
O -5.327130 1.198264 -1.629732
O 4.076161 -0.056717 -0.641521
O 6.241979 -1.805463 -1.037595
O 7.331959 1.581441 -0.723939
O 6.177506 2.705544 1.670894
O 4.336664 0.689779 2.184053
H 2.179833 -1.477371 -1.571753
H 1.163892 0.643885 -1.262512
H -0.252358 1.178766 0.692275
H -0.156312 -0.493170 1.240608
H 0.109145 -1.304493 -2.673595
H 0.156330 -2.928382 -2.010262
H -4.747825 -2.612778 0.434221
H -3.856410 -2.921047 -1.065775
H 1.690395 -3.478120 -0.158580
H 2.604821 -2.216922 0.690836
H 0.875856 -2.453672 1.023148
H -2.507391 0.699009 -2.193138
H -1.511042 1.828542 -1.268731
H -0.756492 0.691619 -2.382906
H -1.716710 -0.072831 3.149128
H -1.585512 -1.909882 3.319548
H -3.865125 -0.566394 -1.490964
H -2.199766 1.654334 1.441509
H -3.351906 1.905114 0.122076
H -3.885645 1.169442 1.642168
H -7.327029 0.427937 0.890221
H -8.726067 2.233089 0.362608
H -6.719343 3.036452 -1.855170
H -8.491500 3.068991 -2.028300
H -7.727759 4.137946 -0.869335
H 3.025467 1.722618 -0.770010
H 5.069332 1.230340 -1.958638
H 6.429554 0.245229 0.603589
H 5.197668 2.955150 -0.085851
H 3.449416 2.457330 1.514698
H 5.265612 -1.063711 -2.730914
H 6.883111 -0.408246 -2.372036
H 5.417231 -2.049479 -0.596478
H 7.804392 2.074340 -0.039195

H 6.189375 2.061246 2.393696
H 3.494262 0.328119 2.489118

3g-c61 , delta G = 0.0428 kcal/mol, population = 42.50 %
C -2.797592 -2.841618 0.187024
C -4.015897 -2.223468 -0.512391
C -3.788409 -0.697884 -0.443774
C -2.483032 -0.488898 0.408017
C -1.233063 -0.555347 -0.558623
C 0.079314 -0.653211 0.252736
C 1.298007 -0.857866 -0.642311
C 1.217390 -2.158411 -1.439702
C -0.092251 -2.137869 -2.248211
C -1.356421 -1.818140 -1.451777
C -2.495709 -1.780667 1.219429
C -1.898558 -2.029562 2.523645
C -2.511421 0.776408 1.255114
C -1.193827 0.704317 -1.442771
C 1.392623 -3.413587 -0.579688
C 3.102830 0.340768 0.369210
C 4.116596 0.189185 1.503645
C 4.918760 1.478804 1.635155
C 5.513860 1.900617 0.292099
C 4.407902 1.987023 -0.759424
C 4.917780 2.303596 -2.154752
C -5.341616 1.119736 -0.179360
C -6.492262 1.539474 0.635587
C -7.112877 2.727258 0.567252
C -6.820613 3.894448 -0.307920
O -1.668958 -2.995416 -0.681858
H -2.165340 -1.649546 -2.183109
O -3.325836 -1.832197 2.397866
H -2.989721 -3.827591 0.619445
O 2.498173 -0.893940 0.173349
O -4.935862 -0.113585 0.222325
O -4.809850 1.738610 -1.085440
O 3.745706 0.718258 -0.850516
O 5.863412 1.343648 -2.623699
O 6.114245 3.190972 0.393333
O 5.936183 1.374663 2.631751
O 5.042906 -0.863543 1.249647
H 2.043968 -2.141459 -2.164711
H 1.398402 -0.023259 -1.348474
H 0.217839 0.262145 0.839274
H 0.036163 -1.487573 0.958391
H -0.003369 -1.395539 -3.050209
H -0.239855 -3.106737 -2.740417
H -4.913795 -2.479424 0.057881
H -4.144940 -2.589291 -1.535134
H 1.291327 -4.305337 -1.207497
H 2.386033 -3.431728 -0.121768
H 0.641423 -3.484004 0.210553
H -2.175029 0.944462 -1.861048
H -0.859869 1.567366 -0.858409
H -0.501631 0.588943 -2.279837
H -1.374297 -1.224863 3.039722
H -1.593648 -3.043371 2.786330
H -3.712447 -0.239775 -1.430744
H -1.572831 0.908239 1.800092
H -2.671737 1.662476 0.636037
H -3.318843 0.720028 1.988730
H -6.840006 0.802789 1.354182
H -7.950378 2.864557 1.252588
H -7.695185 4.103604 -0.938178
H -6.678857 4.787649 0.313723
H -5.948785 3.744055 -0.941363
H 2.366098 1.124587 0.637983
H 3.689206 2.773334 -0.462214
H 6.252399 1.151372 -0.033723
H 4.244177 2.276574 1.976211
H 3.562180 0.003921 2.438617
H 4.054044 2.370680 -2.834149
H 5.426542 3.271701 -2.142836
H 5.431698 0.479721 -2.593278
H 6.716791 3.159435 1.148981
H 6.429718 0.562533 2.446215
H 4.534924 -1.674406 1.114977

3g-c10 , delta G = 0.8009 kcal/mol, population = 11.81 %
C -2.796859 -2.894487 -0.002728
C -4.027270 -2.232324 -0.638018
C -3.796339 -0.714184 -0.475534
C -2.474055 -0.560516 0.361098
C -1.242111 -0.566398 -0.632209
C 0.084710 -0.715299 0.146985
C 1.289872 -0.876682 -0.775355

C 1.184510 -2.121464 -1.654785
C -0.137338 -2.039215 -2.438945
C -1.386017 -1.770002 -1.600904
C -2.472052 -1.901711 1.088399
C -1.846957 -2.232532 2.360751
C -2.483232 0.647968 1.287523
C -1.215035 0.744828 -1.437350
C 1.362123 -3.430191 -0.879060
C 3.114441 0.225808 0.311120
C 4.091979 -0.015173 1.462851
C 4.925117 1.240818 1.693611
C 5.546695 1.729300 0.386720
C 4.462272 1.914988 -0.675966
C 5.021731 2.323366 -2.036506
C -5.342667 1.081606 -0.060732
C -6.476191 1.445217 0.803741
C -7.095990 2.635065 0.829465
C -6.818963 3.858967 0.030208
O -1.686468 -2.993838 -0.902209
H -2.208433 -1.551704 -2.303319
O -3.276469 -2.028020 2.278924
H -2.982689 -3.905742 0.369985
O 2.495460 -0.983244 0.026297
O -4.929422 -0.175128 0.250771
O -4.827665 1.760284 -0.932875
O 3.797901 0.662632 -0.867860
O 5.463915 3.677898 -2.056872
O 6.197761 2.988957 0.582829
O 5.927178 1.040978 2.690109
O 4.999547 -1.075616 1.174072
H 1.999554 -2.065015 -2.390632
H 1.396203 0.001636 -1.425722
H 0.238830 0.166994 0.778660
H 0.049363 -1.585910 0.808027
H -0.058403 -1.243340 -3.189045
H -0.297733 -2.971495 -2.993719
H -4.915124 -2.524140 -0.069287
H -4.174906 -2.531673 -1.679667
H 1.227332 -4.280314 -1.556313
H 2.367923 -3.491119 -0.453155
H 0.633582 -3.536387 -0.071695
H -2.196154 0.996760 -1.849022
H -0.896306 1.574977 -0.799426
H -0.515117 0.688805 -2.274016
H -1.310438 -1.461775 2.914716
H -1.537973 -3.260806 2.553235
H -3.740110 -0.192949 -1.431964
H -2.659655 1.571085 0.730161
H -3.272063 0.544053 2.036203
H -1.531469 0.747468 1.816349
H -6.810614 0.661130 1.477092
H -7.919429 2.725381 1.539195
H -7.704953 4.110180 -0.567944
H -6.664449 4.708077 0.708063
H -5.959360 3.751878 -0.628379
H 2.385408 1.006667 0.607022
H 3.744857 2.685513 -0.337728
H 6.266316 0.978505 0.020125
H 4.268121 2.033559 2.077233
H 3.505663 -0.239924 2.369195
H 5.830758 1.629868 -2.318892
H 4.222939 2.236714 -2.780908
H 6.079312 3.781908 -1.316036
H 6.852410 2.866197 1.283870
H 6.373732 0.208652 2.476472
H 4.476024 -1.862262 0.971574

3h

3h-c54 , delta G = 0.0000 kcal/mol, population = 43.50 %

C 2.544399 -2.863027 -0.657096
C 3.825359 -2.334196 0.003047
C 3.563242 -0.831718 0.246313
C 2.157523 -0.525895 -0.387643
C 1.036816 -0.828035 0.687465
C -0.359561 -0.827035 0.025693
C -1.466014 -1.256614 0.984347
C -1.252924 -2.676382 1.512447
C 0.140929 -2.740925 2.162782
C 1.294693 -2.228307 1.302274
C 2.097105 -1.642026 -1.426265
C 1.349289 -1.664849 -2.674921
C 2.055063 0.874971 -0.977711
C 1.079390 0.239640 1.794110
C -1.489120 -3.757326 0.452919
C -3.535601 -0.103917 0.620288
C -4.913846 -0.324862 -0.005693
C -5.764358 0.924504 0.198497

C -5.031881 2.182127 -0.274138
C -3.665410 2.262628 0.402757
C -2.829444 3.426452 -0.096244
C 5.040252 1.065077 0.125051
C 6.060445 1.671505 -0.744230
C 6.644989 2.864674 -0.557311
C 6.431141 3.859531 0.528096
O 1.533776 -3.226249 0.291168
H 2.182988 -2.165261 1.954022
O 2.777644 -1.437226 -2.681310
H 2.703592 -3.741441 -1.288846
O -2.746307 -1.199283 0.302782
O 4.604462 -0.091594 -0.439536
O 4.627095 1.489475 1.190805
O -2.922840 1.075688 0.093622
O -7.037557 0.812302 -0.436717
O -5.768046 3.352253 0.075812
O -4.829647 -0.553378 -1.410043
O -1.555801 3.478309 0.546014
H -1.988733 -2.836945 2.314162
H -1.514957 -0.566161 1.838718
H -0.597031 0.172186 -0.349362
H -0.376255 -1.507820 -0.830352
H 0.131067 -2.155605 3.090084
H 0.367090 -3.774353 2.452247
H 4.654715 -2.444914 -0.701788
H 4.080763 -2.875810 0.918427
H -1.277057 -4.743735 0.879018
H -2.530593 -3.747688 0.118407
H -0.840035 -3.629688 -0.416314
H 0.734095 1.202347 1.405243
H 0.434671 -0.020947 2.636659
H 2.088068 0.384297 2.190564
H 0.751650 -0.798590 -2.960195
H 1.032385 -2.621887 -3.091433
H 3.606385 -0.561479 1.301970
H 1.047120 1.075584 -1.351310
H 2.298812 1.636485 -0.233021
H 2.748730 0.981242 -1.815010
H 6.332874 1.081332 -1.614659
H 7.379529 3.155348 -1.309556
H 6.190771 4.834187 0.084885
H 5.648191 3.568043 1.225389
H 7.370203 4.003633 1.078876
H -3.642707 0.013546 1.718263
H -5.391484 -1.180020 0.500256
H -3.800944 2.346393 1.496589
H -4.882843 2.126889 -1.366211
H -5.969704 1.035793 1.272256
H -6.874196 0.508509 -1.341625
H -6.675780 3.210599 -0.226719
H -4.266271 -1.326086 -1.547943
H -3.340468 4.365732 0.131468
H -2.718383 3.343538 -1.189466
H -1.120461 2.632597 0.374048

3h-c63 , delta G = 0.1087 kcal/mol, population = 36.21 %

C 2.572235 -2.877807 -0.570547
C 3.856834 -2.330047 0.066332
C 3.593447 -0.822197 0.271449
C 2.183969 -0.535064 -0.362920
C 1.070059 -0.807869 0.726690
C -0.330812 -0.828045 0.075300
C -1.427188 -1.230962 1.056214
C -1.211226 -2.637096 1.617705
C 0.187073 -2.682023 2.260674
C 1.334385 -2.189660 1.379620
C 2.118136 -1.678962 -1.369991
C 1.361948 -1.736946 -2.612434
C 2.076905 0.849558 -0.988473
C 1.114633 0.294935 1.799386
C -1.452946 -3.743958 0.586824
C -3.512420 -0.100656 0.717890
C -4.871046 -0.303768 0.046873
C -5.730095 0.934954 0.272252
C -4.991584 2.204714 -0.150374
C -3.639041 2.278357 0.557038
C -2.781205 3.451176 0.113360
C 5.069703 1.070893 0.095825
C 6.085710 1.655674 -0.793073
C 6.686252 2.844098 -0.627544
C 6.498796 3.853217 0.449570
O 1.568853 -3.215170 0.394997
H 2.226613 -2.106670 2.023744
O 2.789856 -1.507653 -2.634753
H 2.727627 -3.773437 -1.178614
O -2.711435 -1.182454 0.381465

O 4.630202 -0.098605 -0.438403
O 4.662395 1.520837 1.153435
O -2.883464 1.097356 0.256645
O -6.990670 0.837171 -0.391400
O -5.735736 3.363928 0.221499
O -4.745475 -0.482040 -1.361604
O -2.502498 3.425618 -1.285989
H -1.941632 -2.778206 2.428052
H -1.464255 -0.519754 1.893479
H -0.573890 0.160264 -0.324345
H -0.353669 -1.533845 -0.760030
H 0.181422 -2.072704 3.172483
H 0.417780 -3.706965 2.575620
H 4.681853 -2.458264 -0.640604
H 4.118594 -2.846983 0.994098
H -1.238590 -4.719844 1.035437
H -2.496247 -3.742002 0.257822
H -0.808374 -3.637432 -0.288540
H 0.504074 0.042133 2.669549
H 2.130044 0.481066 2.159828
H 0.727162 1.233194 1.390362
H 0.761121 -0.879631 -2.917316
H 1.043527 -2.705573 -2.999955
H 3.640966 -0.524580 1.319559
H 1.066550 1.039304 -1.361034
H 2.322461 1.629168 -0.263312
H 2.766366 0.936052 -1.831506
H 6.341912 1.051477 -1.658787
H 7.414638 3.117458 -1.392166
H 5.728317 3.573997 1.165357
H 7.449535 3.999717 0.979190
H 6.253967 4.823577 -0.000738
H -3.649317 -0.023911 1.816469
H -5.358073 -1.178304 0.509041
H -3.806936 2.359138 1.647192
H -4.821202 2.183959 -1.238432
H -5.958628 1.011705 1.344455
H -6.806818 0.580032 -1.306648
H -6.631650 3.240542 -0.120737
H -4.167847 -1.242353 -1.509498
H -1.849395 3.442734 0.699995
H -3.311827 4.385837 0.315673
H -2.081656 2.576662 -1.476369

3h-c76 , delta G = 0.6383 kcal/mol, population = 14.79 %

C 2.375193 -2.625187 -0.984292
C 3.733297 -2.240803 -0.379910
C 3.542046 -0.805970 0.157852
C 2.092917 -0.369621 -0.268270
C 1.073656 -0.843726 0.846274
C -0.381296 -0.711430 0.338331
C -1.398459 -1.295839 1.315728
C -1.169320 -2.786655 1.568430
C 0.278765 -2.975032 2.052514
C 1.352402 -2.332826 1.177281
C 1.893906 -1.279851 -1.475515
C 1.029953 -1.066545 -2.627448
C 1.983279 1.116593 -0.583351
C 1.253061 0.014751 2.110406
C -1.536889 -3.669329 0.371026
C -3.257472 0.142461 0.775151
C -4.760849 0.078502 1.062316
C -5.389153 1.448969 0.837893
C -5.015857 2.015840 -0.532657
C -3.496899 2.007062 -0.685436
C -3.037604 2.468097 -2.055605
C 5.071081 1.050446 0.252802
C 6.012920 1.805175 -0.588349
C 6.686103 2.907078 -0.223525
C 6.668145 3.637485 1.072514
O 1.455210 -3.137808 -0.013160
H 2.305028 -2.396154 1.730326
O 2.457343 -0.865128 -2.737000
H 2.440594 -3.378684 -1.774185
O -2.755133 -1.152857 0.827231
O 4.535146 0.037462 -0.477403
O 4.790177 1.257685 1.421077
O -3.020796 0.662936 -0.535863
O -6.806506 1.419478 1.006228
O -5.467713 3.362549 -0.659149
O -5.424032 -0.837312 0.194222
O -1.614270 2.448359 -2.167820
H -1.825638 -3.076544 2.401887
H -1.341442 -0.763676 2.277808
H -0.613803 0.344727 0.170906
H -0.503213 -1.225270 -0.619290
H 0.377173 -2.558302 3.062012

H 0.503461 -4.045068 2.136526
H 4.481401 -2.228533 -1.177966
H 4.064506 -2.943977 0.389619
H -2.608682 -3.609878 0.161566
H -0.988722 -3.389822 -0.531411
H -1.289903 -4.712720 0.594797
H 0.703723 -0.397353 2.960020
H 2.301594 0.095986 2.410152
H 0.874687 1.026700 1.936081
H 0.438460 -0.152733 -2.696601
H 0.640791 -1.927653 -3.172246
H 3.688547 -0.735952 1.236164
H 0.947835 1.407065 -0.780081
H 2.350853 1.723751 0.247619
H 2.573312 1.358294 -1.470496
H 6.144316 1.415601 -1.593868
H 7.339165 3.336649 -0.984402
H 7.681894 3.654011 1.493940
H 6.401119 4.686970 0.893543
H 5.982817 3.202735 1.797426
H -2.766825 0.797776 1.522336
H -4.896370 -0.217521 2.115773
H -3.045287 2.652948 0.089971
H -5.454249 1.381596 -1.322178
H -5.007952 2.138651 1.603938
H -7.134203 0.669187 0.488923
H -6.399523 3.367990 -0.399872
H -5.020844 -1.706110 0.322507
H -3.357382 3.500590 -2.219250
H -3.503812 1.829494 -2.823140
H -1.333755 1.544230 -1.971834

3h-c14 , delta G = 1.2241 kcal/mol, population = 5.50 %

C 2.626362 -2.907938 -0.551886
C 3.900816 -2.348121 0.095089
C 3.628349 -0.839423 0.281395
C 2.226082 -0.564961 -0.373981
C 1.098989 -0.831269 0.704021
C -0.293148 -0.868037 0.033844
C -1.400431 -1.265961 1.004471
C -1.182542 -2.662811 1.588545
C 0.207691 -2.691094 2.249384
C 1.362534 -2.203915 1.375764
C 2.177400 -1.719472 -1.369569
C 1.436701 -1.792333 -2.620447
C 2.122072 0.812874 -1.014851
C 1.123164 0.284356 1.763656
C -1.404546 -3.785064 0.569637
C -3.493059 -0.162296 0.616884
C -4.849531 -0.411187 -0.045406
C -5.732711 0.819559 0.131172
C -4.999907 2.083643 -0.314971
C -3.658881 2.200052 0.410749
C -2.830158 3.395744 -0.051740
C 5.102557 1.055618 0.107628
C 6.127201 1.635788 -0.774406
C 6.723301 2.826760 -0.611256
C 6.521685 3.843569 0.456071
O 1.612828 -3.239302 0.405193
H 2.247155 -2.109894 2.028676
O 2.864047 -1.558578 -2.627736
H 2.792805 -3.809291 -1.148460
O -2.675552 -1.239641 0.310966
O 4.670824 -0.118391 -0.422795
O 4.683743 1.512432 1.157760
O -2.884430 1.033586 0.118507
O -6.976438 0.685943 -0.555664
O -5.775253 3.246701 -0.006895
O -4.722105 -0.638075 -1.446805
O -3.348725 4.633875 0.425234
H -1.922254 -2.797680 2.391498
H -1.456329 -0.543163 1.830624
H -0.537184 0.113008 -0.382253
H -0.298823 -1.585758 -0.791415
H 0.187740 -2.069167 3.152454
H 0.439923 -3.710386 2.581096
H 4.735080 -2.479776 -0.600255
H 4.153111 -2.854539 1.031240
H -1.185721 -4.753188 1.032668
H -2.444626 -3.796613 0.230828
H -0.752665 -3.683721 -0.300884
H 0.494712 0.043051 2.624303
H 2.131319 0.474169 2.142241
H 0.744610 1.217429 1.335129
H 0.836747 -0.939786 -2.940095
H 1.126046 -2.765684 -3.002380
H 3.660505 -0.530373 1.326710

H 2.816782 0.890861 -1.854454

H 1.113819 0.998465 -1.394886

H 2.362934 1.600086 -0.296319

H 6.394482 1.025583 -1.632539

H 7.459957 3.095711 -1.369501

H 5.737497 3.572350 1.159997

H 7.463524 3.988181 1.001982

H 6.290076 4.812415 -0.004182

H -3.631053 -0.054907 1.712599

H -5.321658 -1.276600 0.448161

H -3.835899 2.284067 1.498999

H -4.803295 2.029590 -1.398742

H -5.981266 0.925580 1.196104

H -6.771127 0.368988 -1.447418

H -6.636406 3.136785 -0.433000

H -4.125918 -1.388612 -1.568935

H -2.760009 3.383136 -1.151905

H -1.817869 3.294738 0.355122

H -4.282754 4.663791 0.170147

Table S4. Experimental (Exptl.) of **3** and the calculated ^{13}C chemical shift values of possible isomers (**3a**, **3b**, **3c**, **3d**, **3e**, **3f**, **3g** and **3h**) with GIAO ^{13}C NMR calculations with STS protocol.

**Carbon no.**	**Exptl.**	**3a**	**3b**	**3c**	**3d**
1	79.2	78.71	79.33	78.52	78.74
2	37	35.07	35.68	34.80	34.96
3	74	72.99	73.45	72.88	73.01
4	49.5	51.00	51.61	51.16	51.24
5	40.5	41.10	41.62	41.29	41.79
6	34.4	35.63	31.51	31.06	30.29
7	80.7	80.67	75.30	82.77	81.77
8	29.4	29.62	29.69	32.91	32.37
9	30.1	29.89	30.44	27.83	27.72
10	72.4	70.78	71.64	71.00	71.43
11	65.7	66.03	66.48	66.00	66.10
12	48.4	47.99	48.82	47.69	48.00
13	5.8	6.70	7.24	6.74	7.12
14	20.5	21.69	21.66	22.01	21.17
15	17.7	17.50	18.46	20.11	19.81
16	102.1	102.11	96.99	101.96	100.85
17	70.9	70.90	72.14	71.10	71.68
18	74.1	74.13	75.10	73.98	74.46
19	67.5	67.97	69.25	67.38	68.13
20	75.6	74.80	75.17	74.69	74.89
21	61.8	62.18	63.06	61.96	62.16
22	166.4	166.47	167.16	166.13	166.02
23	120.7	118.93	119.37	118.67	118.79
24	145.5	147.75	149.07	148.01	148.01
25	15.5	14.80	15.15	14.75	14.91
	MAE	0.73	1.45	1.28	1.30
	RMS	0.98	2.00	1.63	1.60
	Pmean	47.79%	11.35%	19.04%	19.41%
	Prel	100.00%	0.00%	0.00%	0.00%

**Carbon no.**	**Exptl.**	**3e**	**3f**	**3g**	**3h**
1	79.2	79.57	78.86	79.39	78.94
2	37	35.13	34.75	35.61	35.11
3	74	73.27	72.49	73.59	72.91
4	49.5	51.08	50.40	51.72	51.03
5	40.5	43.60	43.15	44.49	44.20
6	34.4	34.49	36.33	30.72	33.01
7	80.7	78.73	81.54	76.42	79.12
8	29.4	29.07	31.79	33.24	32.60
9	30.1	33.53	32.99	31.80	31.54

10	72.4	70.42	70.00	70.99	70.49
11	65.7	66.14	65.63	66.47	66.03
12	48.4	48.42	47.99	48.62	48.31
13	5.8	6.84	6.41	7.54	7.25
14	20.5	18.58	18.21	19.74	19.47
15	17.7	18.02	17.33	15.46	14.78
16	102.1	94.57	101.18	99.27	101.51
17	70.9	75.24	70.77	72.06	71.47
18	74.1	69.36	73.99	74.88	74.30
19	67.5	70.17	67.35	67.22	68.00
20	75.6	76.85	74.69	74.93	74.84
21	61.8	61.93	61.86	61.24	62.39
22	166.4	167.33	166.36	166.62	166.02
23	120.7	119.57	118.57	118.97	118.50
24	145.5	148.62	148.48	148.97	148.44
25	15.5	14.86	14.28	15.44	15.16
	MAE	1.83	1.22	1.62	1.32
	RMS	2.53	1.58	2.09	1.66
	Pmean	3.83%	21.67%	8.83%	18.43%
	Prel	0.00%	0.00%	0.00%	0.00%

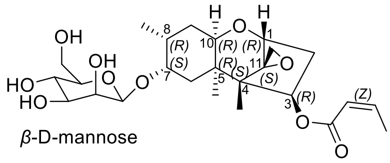

3a

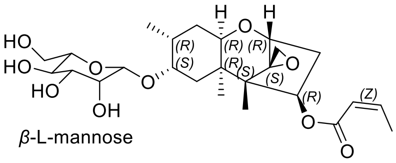

3b

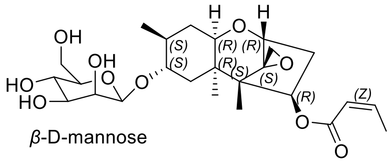

3c

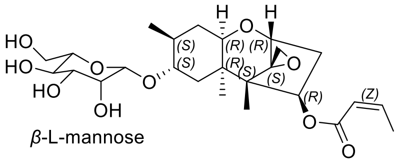

3d

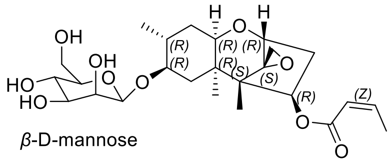

3e

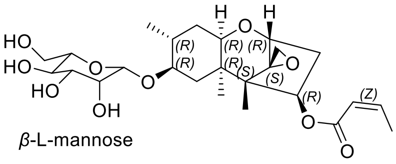

3f

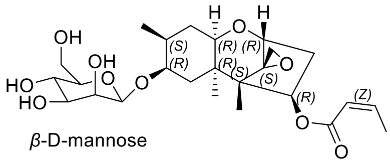

3g

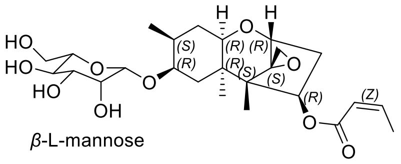

3h

Table S5. Geometry data of conformers of structure **3a** for ECD calculation.

3a

3a-c7 , delta G = 0.0000 kcal/mol, population = 34.77 %

C -3.229567 -2.464110 0.509776
C -4.070887 -1.645010 -0.479541
C -3.373492 -0.269110 -0.558618
C -2.193026 -0.317037 0.477850
C -0.915622 -0.933178 -0.231718
C 0.160970 -1.268108 0.827710
C 1.336655 -2.112679 0.335205
C 0.848684 -3.397241 -0.332334
C -0.150416 -3.070150 -1.443815
C -1.332091 -2.235407 -0.962001
C -2.739376 -1.373653 1.433639
C -2.448968 -1.543843 2.850185
C -1.909721 1.027314 1.135734
C -0.362002 0.070741 -1.258235
C 1.997065 -4.264543 -0.841208
C 3.292989 -0.792968 -0.074901
C 4.204974 -0.318417 -1.203112
C 5.402358 0.416037 -0.589386
C 4.943114 1.537101 0.343602
C 3.960785 0.989357 1.377614
C 3.338143 2.058247 2.259612
C -4.290188 1.949001 -0.779765
C -5.327824 2.822794 -0.211059
C -5.504202 4.123659 -0.490864
C -4.733018 5.005477 -1.407433
O -2.107626 -3.106030 -0.109138
H -1.940480 -1.968374 -1.842263
O -3.700775 -0.953323 2.427231
H -3.791169 -3.250045 1.022579
O 2.177149 -1.402262 -0.612023
O -4.348762 0.734997 -0.174822
O -3.491215 2.223283 -1.660274
O 2.872613 0.348766 0.694824
O 2.617703 3.037713 1.509124
O 6.048855 2.102952 1.046366
O 6.269035 0.930065 -1.601273
O 3.518598 0.522862 -2.132729
H 0.315410 -3.951371 0.452902
H 1.961078 -2.375812 1.204179
H 0.545264 -0.348566 1.275957
H -0.291864 -1.855269 1.633730
H 0.360693 -2.536867 -2.254261
H -0.542369 -3.998074 -1.878803
H -5.075262 -1.518039 -0.064900
H -4.162981 -2.131141 -1.454865
H 2.533819 -3.765637 -1.654932
H 1.617437 -5.219794 -1.219781
H 2.714829 -4.476884 -0.040192
H 0.380695 -0.398451 -1.904153
H -1.151866 0.485757 -1.891073
H 0.137961 0.900739 -0.749501
H -1.767733 -0.852000 3.346116
H -2.533939 -2.534719 3.297933
H -3.028418 -0.028982 -1.564572
H -1.676632 1.790291 0.389137
H -2.779720 1.361303 1.705922
H -1.064236 0.959267 1.825775
H -5.986739 2.345826 0.509113
H -6.326399 4.613215 0.032682
H -5.401835 5.390316 -2.188783
H -4.383887 5.886044 -0.853252
H -3.887107 4.503916 -1.873276
H 3.849673 -1.477843 0.595585
H 4.484559 0.261684 2.023726
H 4.431579 2.313888 -0.249397
H 6.000442 -0.299159 -0.007268
H 4.562786 -1.185374 -1.772249
H 2.682859 1.567083 2.994472
H 4.128697 2.588507 2.798352
H 1.914255 2.574365 1.034702
H 6.703654 2.354786 0.380634
H 5.704246 1.405460 -2.229754
H 2.923280 1.098517 -1.629141

3a-c3 , delta G = 0.2936 kcal/mol, population = 21.17 %

C -3.225276 -2.450776 0.441115
C -4.037824 -1.569082 -0.517706
C -3.312192 -0.205612 -0.523160
C -2.148969 -0.324856 0.526489
C -0.872533 -0.931239 -0.194286
C 0.183959 -1.336127 0.861816
C 1.346907 -2.189033 0.351147

C 0.837046 -3.430332 -0.378521
C -0.129261 -3.024574 -1.493256
C -1.302091 -2.189100 -0.991831
C -2.728421 -1.415355 1.423011
C -2.461303 -1.659135 2.833483
C -1.851554 0.982844 1.249162
C -0.287239 0.111447 -1.161280
C 1.969345 -4.310272 -0.901860
C 3.308717 -0.829998 0.026603
C 4.282045 -0.458544 -1.095112
C 5.407580 0.397231 -0.525351
C 4.853682 1.591070 0.250904
C 3.881530 1.100264 1.323470
C 3.186989 2.221598 2.078098
C -4.204227 2.028041 -0.686089
C -5.236777 2.895039 -0.098170
C -5.461635 4.180981 -0.410574
C -4.764150 5.051494 -1.394347
O -2.107568 -3.084152 -0.193695
H -1.891047 -1.868021 -1.867045
O -3.696665 -1.026600 2.422568
H -3.808510 -3.249464 0.908194
O 2.227716 -1.472779 -0.552795
O -4.270778 0.801576 -0.107356
O -3.402670 2.316533 -1.559690
O 2.837651 0.339806 0.704794
O 2.420739 3.065150 1.216005
O 5.906483 2.295267 0.910428
O 6.315701 0.828051 -1.540803
O 3.646238 0.303851 -2.119600
H 0.271422 -3.999999 0.371894
H 1.950117 -2.504295 1.217880
H 0.583938 -0.446004 1.353243
H -0.292186 -1.945449 1.637335
H 0.411781 -2.462562 -2.263975
H -0.532222 -3.919816 -1.983239
H -5.044359 -1.441451 -0.108428
H -4.128028 -2.006609 -1.516007
H 2.530304 -3.799185 -1.691274
H 1.570023 -5.241915 -1.317120
H 2.670314 -4.567599 -0.099231
H 0.485633 -0.328699 -1.792308
H -1.053252 0.543986 -1.811579
H 0.183419 0.924324 -0.600629
H -1.775975 -1.004314 3.372257
H -2.570085 -2.669111 3.230333
H -2.946507 0.072816 -1.511544
H -1.637916 1.785840 0.539270
H -2.708528 1.283487 1.856495
H -0.988448 0.879805 1.912484
H -5.855533 2.422761 0.659674
H -6.273716 4.664571 0.133946
H -4.388927 5.947341 -0.883030
H -3.944116 4.550662 -1.905024
H -5.489518 5.412890 -2.135158
H 3.822488 -1.481949 0.762124
H 4.433557 0.476649 2.050753
H 4.314809 2.259133 -0.439538
H 5.997655 -0.218422 0.167558
H 4.702228 -1.390172 -1.507674
H 2.549993 1.775564 2.856945
H 3.935753 2.855191 2.561974
H 1.790015 2.497154 0.752567
H 6.574479 2.490507 0.238750
H 5.781668 1.220225 -2.247274
H 2.953868 -0.247900 -2.506975

3a-c9 , delta G = 0.6136 kcal/mol, population = 12.33 %

C -3.282983 -2.115969 0.502454
C -4.081467 -1.432109 -0.616352
C -3.359647 -0.089131 -0.860756
C -2.219399 -0.001549 0.217552
C -0.924147 -0.716648 -0.351947
C 0.111902 -0.918271 0.780742
C 1.280479 -1.848037 0.451718
C 0.793170 -3.200634 -0.063830
C -0.152649 -3.002709 -1.249604
C -1.335514 -2.099545 -0.919332
C -2.814568 -0.912476 1.286626
C -2.583676 -0.884835 2.723749
C -1.946676 1.419485 0.694666
C -0.323018 0.141500 -1.478108
C 1.944553 -4.142836 -0.405058
C 3.353827 -0.750954 -0.046427
C 4.268950 -0.449979 -1.235426
C 5.522518 0.260976 -0.736180
C 5.169026 1.475896 0.123953

C 4.232977 1.044309 1.251563
C 3.748209 2.210199 2.092164
C -4.240753 2.078357 -1.420574
C -5.240655 3.110609 -1.105697
C -6.011687 3.241316 -0.014001
C -6.106605 2.397553 1.209482
O -2.148316 -2.842365 0.015440
H -1.912861 -1.943825 -1.845545
O -3.811648 -0.353186 2.170212
H -3.870229 -2.821710 1.096750
O 2.169613 -1.268412 -0.540518
O -4.335932 0.969515 -0.658989
O -3.421532 2.216659 -2.318164
O 3.060469 0.448011 0.681696
O 2.885311 1.781352 3.146827
O 6.343371 2.032907 0.713595
O 6.387743 0.632764 -1.809229
O 3.643713 0.410408 -2.185995
H 0.214579 -3.638825 0.761355
H 1.864772 -2.004571 1.371411
H 0.512149 0.049399 1.097624
H -0.377815 -1.366930 1.651287
H 0.398589 -2.580032 -2.097950
H -0.544452 -3.972096 -1.581992
H -5.094814 -1.235441 -0.253816
H -4.154974 -2.046830 -1.518033
H 2.532747 -3.753986 -1.243088
H 1.561978 -5.129625 -0.687426
H 2.616099 -4.269893 0.451964
H 0.170229 1.024140 -1.060548
H 0.432197 -0.411963 -2.038014
H -1.085902 0.481053 -2.184699
H -1.917623 -0.133292 3.148167
H -2.698072 -1.803285 3.300493
H -2.974890 0.004125 -1.876179
H -1.097998 1.449038 1.383291
H -1.723350 2.081508 -0.145490
H -2.818725 1.816122 1.220615
H -5.300855 3.872928 -1.878257
H -6.670190 4.111182 -0.014659
H -6.147693 3.043704 2.093800
H -7.056279 1.844191 1.193825
H -5.294309 1.680525 1.311669
H 3.858889 -1.463839 0.636835
H 4.750667 0.310674 1.896256
H 4.653485 2.227263 -0.498195
H 6.094657 -0.439835 -0.112840
H 4.552944 -1.406222 -1.703725
H 4.603884 2.702858 2.561681
H 3.240896 2.936438 1.436959
H 2.147650 1.312185 2.733854
H 6.976261 2.172284 -0.004623
H 5.841119 1.088448 -2.466305
H 2.885099 -0.060966 -2.554606

3a-c13 , delta G = 0.7923 kcal/mol, population = 9.12 %

C -3.316452 -2.152465 0.321842
C -4.040816 -1.345924 -0.765482
C -3.268750 -0.011949 -0.857717
C -2.170372 -0.062683 0.265524
C -0.878387 -0.773252 -0.316957
C 0.102596 -1.105059 0.832651
C 1.260994 -2.034358 0.466151
C 0.751079 -3.327301 -0.168142
C -0.141863 -3.004434 -1.367776
C -1.309217 -2.089195 -1.013871
C -2.840129 -1.040082 1.226516
C -2.666606 -1.146372 2.668206
C -1.865011 1.298461 0.877998
C -0.207353 0.155626 -1.343234
C 1.883128 -4.279734 -0.544436
C 3.319503 -0.840189 0.101027
C 4.314593 -0.575193 -1.030959
C 5.498310 0.217327 -0.489171
C 5.029359 1.470936 0.248205
C 4.029887 1.082507 1.337427
C 3.422472 2.271350 2.063188
C -4.054296 2.231307 -1.232001
C -5.027995 3.264692 -0.846403
C -5.825986 3.318533 0.232391
C -5.985802 2.365217 1.365436
O -2.186664 -2.877001 -0.179867
H -1.838617 -1.837548 -1.947886
O -3.851872 -0.522072 2.118750
H -3.953260 -2.883452 0.828025
O 2.196090 -1.422251 -0.459261
O -4.219232 1.056012 -0.592242

O -3.196279 2.427148 -2.081421
O 2.931330 0.380370 0.742778
O 2.719042 3.148759 1.181656
O 6.129589 2.123758 0.882019
O 6.431974 0.551511 -1.517814
O 3.733178 0.196525 -2.080722
H 0.129305 -3.808141 0.599804
H 1.814339 -2.277737 1.387030
H 0.507275 -0.182722 1.257659
H -0.435921 -1.623248 1.633223
H 0.456552 -2.534890 -2.157430
H -0.549499 -3.930633 -1.791718
H -5.064468 -1.148696 -0.433552
H -4.087992 -1.876346 -1.720721
H 2.502467 -3.854717 -1.341254
H 1.479270 -5.233933 -0.899797
H 2.529442 -4.482590 0.317472
H -0.935467 0.610056 -2.021661
H 0.329138 0.961503 -0.833454
H 0.525436 -0.382174 -1.946408
H -1.991809 -0.460299 3.180660
H -2.835762 -2.106177 3.157576
H -2.842658 0.161267 -1.845844
H -1.561499 2.017436 0.113164
H -2.748540 1.691954 1.386663
H -1.059648 1.228495 1.614128
H -5.038329 4.099879 -1.542141
H -6.453230 4.208662 0.299543
H -6.027041 2.924340 2.307242
H -6.954577 1.853996 1.273705
H -5.203752 1.610134 1.416762
H 3.787608 -1.501675 0.858190
H 4.538389 0.441123 2.080743
H 4.533187 2.150431 -0.462800
H 6.046363 -0.415400 0.222583
H 4.666456 -1.547416 -1.412395
H 2.756558 1.893793 2.853886
H 4.218076 2.858802 2.530145
H 2.041521 2.620796 0.737491
H 6.809446 2.243815 0.204573
H 5.924233 0.954801 -2.237289
H 3.013383 -0.323558 -2.461597

3a-c17 , delta G = 0.8244 kcal/mol, population = 8.63 %
C -3.280646 -2.119520 0.523446
C -4.091326 -1.450749 -0.595814
C -3.375393 -0.108853 -0.862556
C -2.225246 -0.005808 0.203919
C -0.933502 -0.724399 -0.369243
C 0.112901 -0.910614 0.756575
C 1.281218 -1.840963 0.427814
C 0.793109 -3.200731 -0.067476
C -0.163968 -3.018673 -1.246854
C -1.346405 -2.115182 -0.916270
C -2.808203 -0.905761 1.289052
C -2.565223 -0.860401 2.723820
C -1.953103 1.421683 0.661932
C -0.343430 0.120883 -1.510519
C 1.944257 -4.143293 -0.408776
C 3.341279 -0.750223 -0.104452
C 4.256828 -0.406607 -1.276689
C 5.537841 0.231883 -0.727280
C 5.222337 1.417793 0.187630
C 4.226389 0.992232 1.264568
C 3.754631 2.151375 2.121341
C -4.268078 2.049202 -1.440061
C -5.267230 3.082864 -1.127532
C -6.026601 3.225413 -0.029158
C -6.106443 2.395862 1.205150
O -2.148725 -2.848912 0.034566
H -1.932511 -1.972078 -1.838978
O -3.799195 -0.338823 2.174743
H -3.860666 -2.819555 1.131457
O 2.156187 -1.269823 -0.582262
O -4.352435 0.949761 -0.663707
O -3.458731 2.178241 -2.347923
O 3.055310 0.459364 0.624806
O 2.843398 1.727086 3.135877
O 6.402330 1.891403 0.833876
O 6.410893 0.633735 -1.782228
O 3.622865 0.462560 -2.217413
H 0.223361 -3.631142 0.767912
H 1.874405 -1.984697 1.344048
H 0.513805 0.061617 1.058186
H -0.368370 -1.349562 1.636685
H 0.378128 -2.602983 -2.104452
H -0.555696 -3.992828 -1.565105

H -5.101730 -1.252381 -0.226076
H -4.171557 -2.076163 -1.489515
H 2.522272 -3.762756 -1.257591
H 1.562379 -5.134874 -0.674799
H 2.624857 -4.257384 0.442929
H 0.127094 1.023195 -1.108505
H 0.429017 -0.427155 -2.051341
H -1.108696 0.428470 -2.229222
H -1.897561 -0.102072 3.133489
H -2.671880 -1.772260 3.312403
H -3.000307 -0.026355 -1.882512
H -1.092817 1.464431 1.335342
H -1.749125 2.076067 -0.188970
H -2.818920 1.818099 1.198281
H -5.337574 3.835390 -1.908788
H -6.687136 4.093708 -0.033284
H -7.051288 1.834028 1.202308
H -5.287749 1.686478 1.310035
H -6.146942 3.051966 2.082042
H 3.855270 -1.455072 0.578604
H 4.683867 0.217170 1.904969
H 4.765127 2.225159 -0.412623
H 6.087071 -0.518058 -0.140935
H 4.512732 -1.323871 -1.821356
H 4.612325 2.598686 2.631132
H 3.296152 2.913683 1.471229
H 2.091566 1.316350 2.688096
H 7.059038 2.044580 0.140269
H 5.867207 1.129347 -2.413729
H 3.097717 1.105432 -1.717365

3a-c1 , delta G = 1.1893 kcal/mol, population = 4.66 %

C -3.278437 -2.475462 0.547451
C -4.162565 -1.764386 -0.486876
C -3.553209 -0.352567 -0.634629
C -2.381397 -0.270312 0.410940
C -1.059435 -0.835658 -0.256637
C 0.028932 -1.045244 0.824262
C 1.249719 -1.853778 0.389743
C 0.850105 -3.194397 -0.223628
C -0.152324 -2.979002 -1.357824
C -1.386633 -2.197338 -0.922497
C -2.868326 -1.309953 1.416208
C -2.583195 -1.388452 2.842002
C -2.194868 1.121089 1.002776
C -0.562024 0.145893 -1.332007
C 2.058813 -4.005500 -0.682534
C 3.292922 -0.703653 -0.108333
C 4.104057 -0.216021 -1.309845
C 5.416917 0.387039 -0.820057
C 5.165171 1.429926 0.268097
C 4.327542 0.822055 1.394698
C 3.958479 1.832218 2.477944
C -4.604450 1.788475 -0.977650
C -5.704539 2.621462 -0.468483
C -5.931563 3.905576 -0.786393
C -5.163718 4.802950 -1.690987
O -2.111353 -3.072531 -0.031293
H -2.003141 -2.012416 -1.817653
O -3.865434 -0.902635 2.379259
H -3.791576 -3.269424 1.097319
O 2.054791 -1.113898 -0.568780
O -4.593878 0.605841 -0.311184
O -3.814481 2.067794 -1.864676
O 3.094537 0.353773 0.841669
O 5.073757 2.185684 3.294606
O 6.403527 1.879190 0.830079
O 6.180316 0.941576 -1.891190
O 3.418405 0.801708 -2.037553
H 0.343580 -3.747669 0.579986
H 1.871533 -2.036722 1.278449
H 0.366866 -0.078140 1.209096
H -0.397593 -1.597612 1.667858
H 0.335209 -2.452289 -2.187039
H -0.482905 -3.947202 -1.754190
H -5.176032 -1.680157 -0.083624
H -4.216716 -2.305186 -1.435963
H 2.768435 -4.156085 0.139368
H 2.585237 -3.495499 -1.496855
H 1.745191 -4.990054 -1.046102
H -0.134993 1.039618 -0.867631
H 0.223993 -0.305185 -1.939067
H -1.367145 0.462327 -2.001522
H -1.953359 -0.629870 3.307230
H -2.609523 -2.358662 3.339636
H -3.215344 -0.144418 -1.650091
H -1.355001 1.142833 1.702275

H -2.002855 1.860553 0.221594
H -3.091673 1.424597 1.547863
H -6.365094 2.129753 0.240219
H -6.796846 4.366292 -0.308198
H -5.796973 5.090115 -2.541331
H -4.929713 5.735749 -1.163246
H -4.248560 4.349589 -2.067218
H 3.838870 -1.527139 0.394938
H 4.885038 -0.014252 1.855349
H 4.608527 2.280821 -0.158209
H 6.031069 -0.415198 -0.388815
H 4.316774 -1.081480 -1.957805
H 3.507888 2.720672 2.006307
H 3.210457 1.378337 3.136976
H 5.780607 2.466021 2.693520
H 6.925662 2.258213 0.109689
H 5.576213 1.494463 -2.408670
H 2.620738 0.407913 -2.414565

3a-c19 , delta G = 1.2711 kcal/mol, population = 4.06 %

C -3.307221 -2.163579 0.306984
C -4.020040 -1.329757 -0.767573
C -3.225674 -0.007897 -0.842970
C -2.126446 -0.092786 0.276882
C -0.848138 -0.818594 -0.317239
C 0.123611 -1.188013 0.828362
C 1.273818 -2.121358 0.445136
C 0.744408 -3.397307 -0.207976
C -0.148603 -3.045257 -1.399637
C -1.301558 -2.117143 -1.031882
C -2.810640 -1.071731 1.225965
C -2.635789 -1.200390 2.665519
C -1.796091 1.254887 0.905838
C -0.160103 0.112079 -1.329723
C 1.859585 -4.362083 -0.603190
C 3.272735 -0.833789 0.093939
C 4.344064 -0.589703 -0.966449
C 5.475413 0.235026 -0.344154
C 4.937120 1.517086 0.290970
C 3.810934 1.183023 1.267950
C 3.118786 2.404730 1.847768
C -3.974957 2.252780 -1.187110
C -4.923406 3.301255 -0.779390
C -5.703230 3.363391 0.312127
C -5.864718 2.406011 1.441236
O -2.191546 -2.901199 -0.207365
H -1.827596 -1.844211 -1.961879
O -3.811941 -0.549348 2.127573
H -3.955738 -2.890143 0.804645
O 2.219619 -1.519778 -0.475390
O -4.156666 1.073128 -0.559947
O -3.121275 2.442400 -2.042275
O 2.797738 0.438052 0.575636
O 2.539399 3.236949 0.841214
O 5.960438 2.197263 1.016523
O 6.487104 0.537968 -1.305513
O 3.816886 0.066513 -2.121714
H 0.118801 -3.880048 0.555592
H 1.824315 -2.387042 1.362491
H 0.532247 -0.282570 1.282889
H -0.425300 -1.724205 1.609865
H 0.453489 -2.573939 -2.185238
H -0.570911 -3.960119 -1.833839
H -5.039471 -1.119908 -0.430440
H -4.077937 -1.845999 -1.729933
H 2.477575 -3.939522 -1.402123
H 1.438450 -5.307238 -0.962742
H 2.510770 -4.581803 0.250860
H -0.878410 0.574632 -2.013298
H 0.375789 0.909907 -0.807508
H 0.578103 -0.425777 -1.926148
H -1.948608 -0.532695 3.185794
H -2.819797 -2.163769 3.142386
H -2.798604 0.172109 -1.829405
H -1.489654 1.980151 0.148067
H -2.669273 1.653665 1.428093
H -0.984676 1.163888 1.633090
H -4.926727 4.142272 -1.468173
H -6.310051 4.266195 0.396155
H -5.853317 2.957771 2.388456
H -6.856070 1.935698 1.377177
H -5.111775 1.620672 1.461505
H 3.709278 -1.388720 0.948173
H 4.220402 0.582792 2.100663
H 4.533008 2.171591 -0.500010
H 5.964758 -0.362458 0.437968
H 4.741485 -1.553032 -1.309506

H 2.355181 2.066849 2.564427
H 3.851368 3.015710 2.383048
H 1.886965 2.702439 0.368725
H 6.709268 2.300751 0.413097
H 6.028778 0.892257 -2.082942
H 3.181621 0.733106 -1.819705

3a-c42 , delta G = 1.7062 kcal/mol, population = 1.95 %
C -3.313731 -2.155573 0.438452
C -4.090349 -1.426804 -0.667439
C -3.359380 -0.078828 -0.849803
C -2.233404 -0.039051 0.246001
C -0.934657 -0.741040 -0.331533
C 0.085277 -0.986038 0.806910
C 1.254207 -1.908755 0.460422
C 0.767706 -3.241241 -0.106324
C -0.162042 -2.998863 -1.296598
C -1.344924 -2.100658 -0.952665
C -2.849213 -0.984565 1.272470
C -2.638230 -1.011146 2.712781
C -1.956635 1.360514 0.780851
C -0.314699 0.152512 -1.419910
C 1.919720 -4.175460 -0.467018
C 3.345188 -0.816851 0.014720
C 4.264990 -0.490517 -1.164182
C 5.524364 0.195691 -0.646190
C 5.169897 1.384485 0.245926
C 4.246571 0.918153 1.371375
C 3.821175 2.030474 2.316690
C -4.220251 2.116690 -1.326804
C -5.218364 3.140120 -0.979177
C -6.003151 3.226244 0.107141
C -6.119427 2.328370 1.289758
O -2.175874 -2.871319 -0.057148
H -1.907227 -1.908948 -1.881473
O -3.854902 -0.451407 2.162728
H -3.914476 -2.878144 0.997956
O 2.154565 -1.298657 -0.502033
O -4.334285 0.974994 -0.618432
O -3.386778 2.289983 -2.205032
O 3.064886 0.352140 0.793217
O 3.202900 3.138280 1.657325
O 6.341613 1.942419 0.840556
O 6.389261 0.595924 -1.709843
O 3.649282 0.401854 -2.091616
H 0.177527 -3.705627 0.696068
H 1.827363 -2.098201 1.380866
H 0.485049 -0.031681 1.161317
H -0.417618 -1.462835 1.654806
H 0.403009 -2.551545 -2.122892
H -0.554191 -3.954516 -1.666300
H -5.109295 -1.239540 -0.315823
H -4.149953 -2.007246 -1.592512
H 2.517600 -3.759596 -1.284989
H 1.537091 -5.150585 -0.787371
H 2.581639 -4.334566 0.392151
H -1.069747 0.537919 -2.111218
H 0.196713 1.006492 -0.966032
H 0.429765 -0.391657 -2.002910
H -1.972930 -0.280074 3.172644
H -2.765485 -1.949345 3.253993
H -2.961423 0.052603 -1.855901
H -2.838640 1.751679 1.294043
H -1.130160 1.351913 1.496350
H -1.696486 2.049250 -0.026612
H -5.263886 3.936923 -1.717191
H -6.656589 4.099383 0.137297
H -6.176893 2.933864 2.201434
H -7.067952 1.775800 1.231912
H -5.308751 1.607161 1.374107
H 3.839994 -1.564928 0.667992
H 4.775391 0.154513 1.973010
H 4.642798 2.141525 -0.358034
H 6.094808 -0.525691 -0.045179
H 4.539407 -1.436347 -1.658715
H 3.075656 1.624788 3.009405
H 4.693430 2.352712 2.900002
H 3.899149 3.678113 1.263221
H 6.965859 2.110865 0.121048
H 5.841460 1.066783 -2.355149
H 2.884890 -0.050961 -2.471396

3a-c16 , delta G = 1.7205 kcal/mol, population = 1.90 %
C -3.286710 -2.132037 0.383709
C -4.018470 -1.335207 -0.706048
C -3.233814 -0.011381 -0.831253

C -2.121004 -0.055209 0.277420
C -0.844279 -0.788999 -0.310888
C 0.147689 -1.112048 0.831480
C 1.294054 -2.056427 0.464613
C 0.762218 -3.354617 -0.140350
C -0.144112 -3.044374 -1.333438
C -1.297628 -2.111582 -0.979911
C -2.786366 -1.010146 1.263459
C -2.592651 -1.095394 2.703795
C -1.793744 1.312123 0.864500
C -0.176660 0.115555 -1.360503
C 1.876295 -4.329553 -0.512998
C 3.290738 -0.769888 0.049834
C 4.353016 -0.643662 -1.044742
C 5.475600 0.260131 -0.551471
C 4.935863 1.591240 -0.026111
C 3.856824 1.328477 1.027460
C 3.207680 2.614169 1.509122
C -4.001308 2.233264 -1.234188
C -4.955542 3.285521 -0.850528
C -5.730533 3.371129 0.242837
C -5.878248 2.443841 1.398790
O -2.172354 -2.876947 -0.121838
H -1.837210 -1.869968 -1.910774
O -3.780256 -0.468443 2.162024
H -3.924124 -2.847687 0.910678
O 2.229598 -1.483009 -0.482718
O -4.169131 1.071480 -0.571109
O -3.155814 2.403921 -2.101410
O 2.825624 0.521887 0.453042
O 2.247574 2.396911 2.546168
O 5.984144 2.344201 0.584820
O 6.470402 0.477085 -1.553606
O 3.822042 -0.059197 -2.232828
H 0.145400 -3.813514 0.644719
H 1.855959 -2.290636 1.383425
H 0.562824 -0.187990 1.241200
H -0.384530 -1.618071 1.644092
H 0.447265 -2.595249 -2.140046
H -0.566729 -3.974250 -1.734122
H -5.034698 -1.121303 -0.361852
H -4.085925 -1.880612 -1.651609
H 1.454134 -5.285957 -0.840212
H 2.532910 -4.522064 0.343474
H 2.489315 -3.931942 -1.328518
H -0.907441 0.552765 -2.047400
H 0.362597 0.930584 -0.870393
H 0.556135 -0.435852 -1.951738
H -1.904190 -0.407865 3.195699
H -2.763510 -2.045741 3.210749
H -2.819283 0.140733 -1.827688
H -2.665953 1.721656 1.380077
H -0.978074 1.245664 1.589540
H -1.494304 2.016949 0.084983
H -4.969875 4.106716 -1.562656
H -6.345315 4.270341 0.304485
H -5.880526 3.022476 2.329782
H -6.860312 1.953063 1.345017
H -5.111297 1.673095 1.443926
H 3.725273 -1.285634 0.930409
H 4.316106 0.800491 1.885116
H 4.487191 2.158380 -0.859889
H 5.988245 -0.246256 0.278108
H 4.756478 -1.648124 -1.252892
H 3.992018 3.310551 1.832980
H 2.661755 3.068992 0.674230
H 2.724427 2.110427 3.335627
H 6.717217 2.360840 -0.046444
H 6.007218 0.762625 -2.354816
H 3.108756 -0.631850 -2.545698

3a-c2 , delta G = 1.8937 kcal/mol, population = 1.42 %

C -3.346194 -2.172828 0.439578
C -4.137761 -1.487419 -0.683227
C -3.432293 -0.131161 -0.901104
C -2.307193 -0.041295 0.192522
C -0.995119 -0.732401 -0.367517
C 0.029993 -0.931316 0.776100
C 1.213362 -1.843647 0.453491
C 0.751636 -3.196864 -0.083353
C -0.181052 -2.998272 -1.279242
C -1.380039 -2.114576 -0.954901
C -2.904384 -0.972176 1.243202
C -2.692978 -0.957793 2.683618
C -2.058582 1.377280 0.689806
C -0.391591 0.145099 -1.477760
C 1.920915 -4.118124 -0.421020

C 3.304913 -0.765837 -0.016069
C 4.215093 -0.441017 -1.202211
C 5.487849 0.228662 -0.694082
C 5.154835 1.408610 0.217104
C 4.227698 0.954406 1.346192
C 3.777628 2.101225 2.247304
C -4.337413 2.031987 -1.440140
C -5.356864 3.044220 -1.123120
C -6.144751 3.145407 -0.040374
C -6.243122 2.279861 1.167670
O -2.195011 -2.878561 -0.039417
H -1.946943 -1.956176 -1.887100
O -3.920265 -0.436100 2.119819
H -3.932130 -2.892734 1.017923
O 2.105385 -1.235860 -0.519760
O -4.426742 0.910166 -0.697080
O -3.508240 2.196108 -2.324183
O 3.039787 0.406449 0.768165
O 4.823446 2.567919 3.098685
O 6.345974 1.937458 0.811352
O 6.339423 0.635346 -1.764917
O 3.598495 0.464062 -2.116418
H 0.168626 -3.652848 0.729042
H 1.787146 -2.001911 1.379067
H 0.415569 0.038149 1.104968
H -0.464766 -1.392349 1.637230
H 0.375981 -2.558995 -2.115260
H -0.555436 -3.968924 -1.627609
H -5.159497 -1.310156 -0.334470
H -4.188335 -2.092369 -1.593055
H 2.515296 -3.708146 -1.244581
H 1.556555 -5.105752 -0.723785
H 2.581582 -4.249107 0.443815
H 0.374237 -0.394466 -2.036960
H -1.151016 0.485130 -2.187784
H 0.089190 1.027911 -1.046155
H -2.042123 -0.202437 3.124539
H -2.802306 -1.884237 3.248433
H -3.036731 -0.018407 -1.910358
H -2.943050 1.758434 1.206346
H -1.221424 1.407184 1.392102
H -1.828826 2.051548 -0.138798
H -5.418265 3.817762 -1.884352
H -6.816816 4.004794 -0.036294
H -6.319892 2.909856 2.061061
H -7.176750 1.701301 1.122586
H -5.414589 1.582423 1.275553
H 3.801013 -1.515886 0.632945
H 4.744598 0.193215 1.959443
H 4.638780 2.189996 -0.364767
H 6.059452 -0.504326 -0.108795
H 4.474137 -1.386382 -1.705602
H 3.374155 2.915041 1.623026
H 2.974190 1.738710 2.897728
H 5.580533 2.764903 2.526326
H 6.922592 2.228438 0.091628
H 5.782648 1.106488 -2.402324
H 2.829985 0.019388 -2.497564

Table S6. Geometry data of conformers of structure **4a** and **4b**

4a

4a-c1 , delta G = 0.0000 kcal/mol, population = 55.31 %

C -1.830173 -1.411487 0.699677
C -2.709334 -1.415058 -0.555031
C -2.413951 -0.072688 -1.267166
C -1.398939 0.679116 -0.331659
C 0.078014 0.222117 -0.616321
C 1.009342 0.710005 0.516227
C 2.464421 0.246183 0.346821
C 2.608189 -1.149928 -0.200632
C 1.553382 -1.847304 -0.625498
C 0.150464 -1.319839 -0.659903
C -1.733878 0.088401 1.047057
C -0.735471 0.485523 2.129315
C -1.560716 2.196031 -0.429721
C 0.568733 0.789818 -1.955744
C 4.000914 -1.705492 -0.201849
C 3.744699 2.256438 0.218125
O -0.536334 -1.957609 0.443332
O -3.035190 0.564171 1.432129
O -3.606760 0.675471 -1.516157
O 0.617649 0.204438 1.796456
O 3.218132 1.135459 -0.488345
H -2.239846 -2.004983 1.526716
H -0.317952 -1.653924 -1.602693
H 1.007722 1.812497 0.539192
H 1.688580 -2.874356 -0.962501
H -3.767396 -1.452835 -0.280493
H -2.497285 -2.289536 -1.178493
H 1.493012 0.301821 -2.270177
H -0.177319 0.646205 -2.742694
H 0.772333 1.860919 -1.871716
H -3.332972 0.045139 2.191866
H -1.980971 -0.231112 -2.259454
H -4.020852 0.826209 -0.654578
H -2.523276 2.510067 -0.022142
H -0.779652 2.729614 0.118163
H -1.523688 2.516894 -1.474201
H -0.868909 1.563487 2.327238
H -0.943729 -0.062186 3.057280
H 2.908897 0.248721 1.358392
H 4.421073 -1.691757 0.812071
H 4.019084 -2.732518 -0.575836
H 4.660229 -1.092011 -0.826399
H 4.443303 1.933898 1.004372
H 4.280066 2.868128 -0.512363
H 2.952164 2.865661 0.676689

4a-c3 , delta G = 0.2839 kcal/mol, population = 34.23 %

C -1.838360 -1.413023 0.695361
C -2.712937 -1.413526 -0.561506
C -2.406378 -0.076194 -1.277837
C -1.395578 0.676373 -0.338372
C 0.081456 0.220034 -0.620548
C 1.008618 0.710384 0.513089
C 2.464055 0.245320 0.348430
C 2.606956 -1.153555 -0.192281
C 1.552241 -1.851600 -0.616453
C 0.150299 -1.322057 -0.658707
C -1.737401 0.082768 1.043604
C -0.739146 0.461206 2.131681
C -1.555131 2.192269 -0.437405
C 0.578228 0.781678 -1.960721
C 3.998920 -1.710980 -0.187611
C 3.748701 2.252384 0.213846
O -0.542678 -1.954871 0.443202
O -3.056090 0.452142 1.482712
O -3.595096 0.673865 -1.542737
O 0.616114 0.204574 1.793283
O 3.219629 1.130537 -0.489321
H -2.251762 -2.000555 1.522119
H -0.314161 -1.658476 -1.602618
H 1.007262 1.812932 0.534502
H 1.686952 -2.880618 -0.947543
H -3.770641 -1.441474 -0.283708
H -2.509605 -2.291706 -1.182621
H 0.790885 1.851209 -1.878885
H 1.499155 0.285888 -2.273053
H -0.167762 0.641907 -2.748426
H -3.035020 1.376285 1.765955
H -1.965638 -0.240746 -2.265633
H -4.041643 0.793331 -0.692757
H -2.541765 2.510153 -0.090156
H -0.805298 2.727255 0.151751

H -1.467999 2.520062 -1.476748
H -0.875091 1.535522 2.363462
H -0.945581 -0.111421 3.043120
H 2.906792 0.251804 1.360705
H 4.015938 -2.741069 -0.553113
H 4.659568 -1.103701 -0.816864
H 4.418543 -1.689391 0.826375
H 2.957580 2.863216 0.672784
H 4.448581 1.930787 0.999310
H 4.283123 2.862151 -0.518953

4a-c5 , delta G = 0.9856 kcal/mol, population = 10.46 %
C -1.828996 -1.414719 0.679791
C -2.686519 -1.431953 -0.593220
C -2.396726 -0.083547 -1.295148
C -1.396631 0.673178 -0.364442
C 0.082718 0.212919 -0.631620
C 0.996177 0.706548 0.513338
C 2.454348 0.244553 0.365456
C 2.610906 -1.150319 -0.181303
C 1.564116 -1.851176 -0.619659
C 0.159592 -1.328536 -0.666348
C -1.755927 0.090151 1.019115
C -0.769331 0.497467 2.106025
C -1.548604 2.191894 -0.476991
C 0.590557 0.775454 -1.967154
C 4.005652 -1.700468 -0.165468
C 3.735263 2.255770 0.259515
O -0.532319 -1.960892 0.433825
O -3.036666 0.520071 1.495676
O -3.593986 0.720817 -1.386775
O 0.584733 0.206241 1.788177
O 3.218631 1.136691 -0.457397
H -2.249357 -1.999927 1.504250
H -0.300056 -1.667158 -1.612664
H 0.994994 1.809165 0.532023
H 1.706475 -2.877543 -0.955693
H -3.748542 -1.489329 -0.333131
H -2.448661 -2.301103 -1.214323
H -0.142857 0.621931 -2.764320
H 0.787409 1.848044 -1.887023
H 1.522081 0.290408 -2.264345
H -3.605676 0.682418 0.726570
H -1.985981 -0.224155 -2.303441
H -4.248366 0.205394 -1.875183
H -0.754338 2.720057 0.055794
H -1.520466 2.505427 -1.524078
H -2.501463 2.522253 -0.059702
H -0.899813 1.577992 2.289497
H -0.992754 -0.037847 3.035902
H 2.882752 0.247172 1.383864
H 4.032320 -2.727404 -0.539216
H 4.670436 -1.084391 -0.781596
H 4.413036 -1.685369 0.853622
H 4.278236 2.871149 -0.462226
H 2.936525 2.861944 0.711310
H 4.425113 1.931165 1.052585

4b

4b-c6 , delta G = 0.0000 kcal/mol, population = 70.77 %
C -1.623415 -0.263224 1.662378
C -2.676147 -1.194379 1.049183
C -2.586905 -0.966349 -0.471050
C -1.487358 0.134728 -0.672855
C -0.048208 -0.493306 -0.684714
C 1.001440 0.631575 -0.538846
C 2.426386 0.064296 -0.567890
C 2.604636 -1.138872 0.325553
C 1.553100 -1.832531 0.765800
C 0.123649 -1.454968 0.514898
C -1.575076 0.910352 0.659284
C -0.436888 1.909257 0.842757
C -1.758730 0.977095 -1.920248
C 0.200553 -1.264860 -1.989454
C 4.016306 -1.553575 0.612409
C 3.456706 2.154391 -1.075420
O -0.354726 -0.909963 1.764679
O -2.774879 1.682429 0.781966
O -3.889354 -0.504868 -0.888991
O 0.855553 1.338681 0.687490
O 3.411154 1.029920 -0.203399
H -1.870295 0.074060 2.674285
H -0.447742 -2.374508 0.291687
H 0.896577 1.336261 -1.381893
H 1.713826 -2.720481 1.375550
H -3.678405 -0.920587 1.392331

H -2.500087 -2.234140 1.339142
H 0.362577 -0.577786 -2.826042
H 1.079662 -1.909720 -1.902468
H -0.649745 -1.904974 -2.241810
H -3.484634 1.217345 0.311834
H -2.341968 -1.886460 -1.016788
H -3.885999 -0.401642 -1.849323
H -1.886397 0.339855 -2.802706
H -2.666114 1.573303 -1.796873
H -0.945691 1.673924 -2.136233
H -0.588083 2.728247 0.117751
H -0.470329 2.334484 1.852159
H 2.627801 -0.254776 -1.610012
H 4.532584 -0.792062 1.206111
H 4.041612 -2.502295 1.155034
H 4.586201 -1.662359 -0.319342
H 2.587773 2.812989 -0.939463
H 4.363111 2.710631 -0.823240
H 3.508497 1.839554 -2.129355

4b-c7 , delta G = 0.5755 kcal/mol, population = 26.76 %
C -1.621071 -0.255828 1.662404
C -2.671695 -1.194782 1.054673
C -2.581027 -0.970156 -0.472940
C -1.491683 0.130064 -0.678071
C -0.050580 -0.496458 -0.685838
C 0.997247 0.630851 -0.545844
C 2.422946 0.065114 -0.573028
C 2.604083 -1.134162 0.324941
C 1.553999 -1.827615 0.768845
C 0.124202 -1.451827 0.517857
C -1.578333 0.911272 0.651905
C -0.442465 1.913956 0.827985
C -1.760226 0.967375 -1.930318
C 0.199759 -1.273057 -1.987195
C 4.016590 -1.546070 0.611468
C 3.450795 2.153629 -1.091466
O -0.352531 -0.901082 1.766084
O -2.780957 1.678858 0.775662
O -3.824699 -0.451420 -0.994572
O 0.851037 1.344449 0.676933
O 3.406546 1.033721 -0.213430
H -1.866767 0.087313 2.672755
H -0.447444 -2.372681 0.299899
H 0.891243 1.331001 -1.392123
H 1.716382 -2.713261 1.381525
H -3.672269 -0.922669 1.406607
H -2.486506 -2.232332 1.349454
H -0.650303 -1.914248 -2.237491
H 0.360525 -0.588846 -2.826197
H 1.079555 -1.916548 -1.897103
H -3.484045 1.221026 0.288445
H -2.329305 -1.894626 -1.009155
H -4.515572 -1.087146 -0.768838
H -2.669170 1.559645 -1.813929
H -0.942349 1.659510 -2.143912
H -1.896390 0.323383 -2.803695
H -0.595487 2.727544 0.097506
H -0.476931 2.345834 1.834536
H 2.624071 -0.258153 -1.613869
H 4.043874 -2.492913 1.157289
H 4.585408 -1.657234 -0.320646
H 4.532687 -0.781849 1.201830
H 2.581115 2.811911 -0.958996
H 4.356636 2.712155 -0.842320
H 3.502780 1.833170 -2.143672

4b-c1 , delta G = 1.9852 kcal/mol, population = 2.47 %
C -1.559549 -0.085024 1.660051
C -2.626866 -1.077437 1.188128
C -2.610731 -0.995074 -0.356987
C -1.544214 0.105940 -0.704570
C -0.093202 -0.497505 -0.734971
C 0.950445 0.645758 -0.748445
C 2.373508 0.080393 -0.811294
C 2.618796 -1.046212 0.169032
C 1.600791 -1.690711 0.745057
C 0.155629 -1.342742 0.539735
C -1.571195 0.989737 0.552921
C -0.445045 2.018529 0.591913
C -1.892284 0.833691 -2.002905
C 0.097268 -1.386140 -1.973094
C 4.041103 -1.497494 0.326941
C 3.640340 1.741143 0.465657
O -0.273364 -0.694259 1.759436
O -2.829845 1.683071 0.584596

O -3.905805 -0.707187 -0.891066

O 0.848346 1.454728 0.419965

O 3.389157 1.085633 -0.785374

H -1.760619 0.338586 2.652097

H -0.410384 -2.285401 0.430744

H 0.807036 1.276816 -1.642220

H 1.802135 -2.536755 1.401242

H -3.614042 -0.783247 1.556229

H -2.419110 -2.083231 1.566966

H 0.215937 -0.778018 -2.875336

H 0.981277 -2.021809 -1.869629

H -0.762739 -2.044072 -2.125516

H -2.933063 2.081411 1.459895

H -2.338705 -1.954507 -0.807253

H -4.179505 0.137044 -0.505151

H -2.808011 1.415437 -1.885726

H -1.101419 1.520873 -2.313598

H -2.058146 0.116392 -2.811467

H -0.647077 2.772424 -0.189255

H -0.431617 2.527974 1.563946

H 2.489366 -0.347559 -1.819132

H 4.102187 -2.375815 0.974925

H 4.464790 -1.753832 -0.652974

H 4.678261 -0.710276 0.742415

H 4.715766 1.946507 0.502624

H 3.085106 2.682219 0.534268

H 3.362559 1.114331 1.320470

Table S7. Experimental (Exptl.) of **4** and the calculated ^{13}C chemical shift values of possible isomers (**4a** and **4b**) with GIAO ^{13}C NMR calculations with STS protocol.

Carbon no.	Exptl.	4a	4b
1	82.5	81.41	82.71
2	40.1	39.90	38.06
3	74.0	74.01	75.15
4	49.6	50.34	50.99
5	38.3	38.12	40.01
6	72.3	70.45	70.45
7	81.8	80.30	78.56
8	136.3	136.91	137.74
9	121.1	123.54	122.38
10	69.4	69.13	69.43
11	75.1	75.73	75.39
12	66.5	66.27	66.38
13	6.9	8.65	8.87
14	15.0	16.08	16.23
15	20.5	20.62	20.43
16	58.9	56.83	55.52
	MAE	0.92	1.34
	RMS	1.20	1.68
	Pmean	35.81%	16.47%
	Prel	100.00%	0.00%

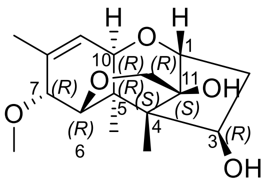

4a

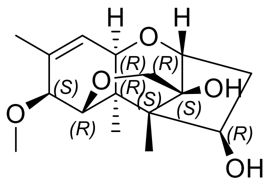

4b

Table S8. Geometry data of conformers of structure **5a** and **5b**

5a

5a-c8 , delta G = 0.0000 kcal/mol, population = 22.56 %

C -1.096098 -3.382499 0.193731
C -2.314095 -2.868252 -0.582674
C -2.218787 -1.336436 -0.500940
C -1.040406 -1.030620 0.485130
C 0.301423 -0.955204 -0.329612
C 1.500856 -0.980320 0.639573
C 2.852816 -0.986304 -0.081694
C 2.892658 -1.803418 -1.339491
C 1.787722 -2.367230 -1.830864
C 0.413148 -2.185306 -1.259456
C -0.937166 -2.335657 1.307636
C 0.336522 -2.421320 2.145013
C -1.280256 0.221297 1.326399
C 0.345976 0.326772 -1.172886
C 4.245981 -1.979758 -1.959416
C 3.765961 1.131001 0.561953
C 3.903572 2.572183 0.120088
C 2.522539 3.181316 -0.011894
C 1.701021 3.296332 1.113916
C 0.406799 3.793193 0.994833
C -0.081345 4.185046 -0.250537
C 0.735409 4.081945 -1.373304
C 2.029223 3.579085 -1.254495
C -3.894697 0.386500 -0.448990
C -5.170260 0.735055 0.195975
C -5.778778 1.928961 0.129397
C -5.344258 3.166849 -0.573764
O 0.080534 -3.436367 -0.612433
O -2.052366 -2.542565 2.178920
O -3.482176 -0.827716 -0.002410
O 1.536282 -2.170639 1.425727
O 3.245507 0.378406 -0.429337
O -3.278555 1.048906 -1.267080
O 4.031407 0.712355 1.668859
H -1.230281 -4.393379 0.592369
H -0.282889 -2.035695 -2.103730
H 1.468113 -0.094762 1.296518
H 1.861233 -3.013949 -2.704009
H -3.231188 -3.204497 -0.093095
H -2.317488 -3.236595 -1.612969
H 1.141869 0.282836 -1.919282
H -0.601650 0.488773 -1.693681
H 0.534201 1.200509 -0.543813
H -2.062921 -1.835997 2.837953
H -2.049203 -0.876141 -1.475767
H -1.409460 1.101457 0.691659
H -2.189124 0.118829 1.926457
H -0.448157 0.423483 2.006642
H 0.241410 -1.705733 2.982899
H 0.432444 -3.427844 2.566426
H 3.595112 -1.376811 0.626221
H 4.945093 -2.420393 -1.237514
H 4.197127 -2.623053 -2.841493
H 4.665215 -1.011135 -2.257181
H -5.623533 -0.057896 0.784253
H -6.721876 2.012394 0.670956
H -4.331214 3.098862 -0.966665
H -6.030828 3.372223 -1.406548
H -5.428903 4.022927 0.106173
H 4.498003 3.094930 0.874336
H 4.425660 2.611717 -0.840789
H 2.076025 2.986169 2.087065
H -0.223722 3.871839 1.876304
H -1.093277 4.569061 -0.344215
H 0.362920 4.385902 -2.347647
H 2.658297 3.485353 -2.136338

5a-c11 , delta G = 0.1429 kcal/mol, population = 17.72 %

C -1.087858 -3.383403 0.187731
C -2.312960 -2.868956 -0.578437
C -2.218615 -1.337477 -0.490078
C -1.039973 -1.035365 0.497831
C 0.299607 -0.954530 -0.322109
C 1.505107 -0.986587 0.640095
C 2.853646 -0.984795 -0.087131
C 2.889208 -1.790391 -1.352363
C 1.782539 -2.350182 -1.844265
C 0.410357 -2.175635 -1.264782
C -0.929246 -2.339240 1.309518
C 0.345701 -2.415650 2.146916
C -1.283529 0.209140 1.349010
C 0.338268 0.335553 -1.152634

C 4.239996 -1.959603 -1.979696
C 3.769455 1.127947 0.569026
C 3.905641 2.572320 0.136955
C 2.524861 3.181271 0.003185
C 1.701628 3.295075 1.127803
C 0.407411 3.791584 1.007184
C -0.078797 4.184492 -0.238581
C 0.739635 4.082483 -1.360229
C 2.033359 3.579793 -1.239889
C -3.897406 0.381741 -0.446309
C -5.172101 0.731263 0.199993
C -5.793798 1.917017 0.113243
C -5.378760 3.144340 -0.619612
O 0.082843 -3.434132 -0.629070
O -2.046735 -2.425431 2.199891
O -3.482031 -0.829020 0.006057
O 1.543016 -2.183081 1.417041
O 3.243216 0.383144 -0.425134
O -3.285007 1.040487 -1.270296
O 4.042050 0.700400 1.670745
H -1.216530 -4.400400 0.577413
H -0.289383 -2.019794 -2.104810
H 1.474181 -0.107123 1.305142
H 1.852653 -2.988160 -2.724135
H -3.227623 -3.206653 -0.085137
H -2.322002 -3.233390 -1.610047
H 0.521261 1.203483 -0.514101
H 1.134892 0.302281 -1.898637
H -0.609739 0.498668 -1.672341
H -2.044337 -3.308792 2.593494
H -2.046366 -0.874438 -1.463056
H -0.437392 0.421417 2.008181
H -1.442018 1.088481 0.719557
H -2.166680 0.070139 1.975459
H 0.247602 -1.684919 2.967709
H 0.445257 -3.415728 2.587046
H 3.599575 -1.380349 0.614177
H 4.657955 -0.987648 -2.268201
H 4.942094 -2.408211 -1.265605
H 4.187751 -2.592966 -2.868755
H -5.613130 -0.054009 0.807502
H -6.733790 2.001528 0.660039
H -5.463399 4.013159 0.044012
H -4.369566 3.077471 -1.022327
H -6.076871 3.326710 -1.448155
H 4.496950 3.090814 0.896540
H 4.431528 2.618133 -0.821660
H 2.075042 2.983776 2.101214
H -0.224805 3.868427 1.887561
H -1.090800 4.568005 -0.333484
H 0.368495 4.387054 -2.334908
H 2.663826 3.486868 -2.120842

5a-c1 , delta G = 0.1652 kcal/mol, population = 17.06 %

C -1.090141 -3.358052 0.147582
C -2.283475 -2.848097 -0.671206
C -2.215215 -1.314428 -0.561113
C -1.045092 -1.002275 0.432505
C 0.311739 -0.941262 -0.358788
C 1.489739 -0.977510 0.637883
C 2.857557 -0.998852 -0.052366
C 2.919271 -1.806759 -1.315099
C 1.820366 -2.354216 -1.836921
C 0.437606 -2.171340 -1.285877
C -0.970666 -2.300481 1.264491
C 0.275951 -2.373373 2.139648
C -1.289467 0.262231 1.255926
C 0.378859 0.341221 -1.198879
C 4.284796 -1.990128 -1.905413
C 3.786947 1.103843 0.618800
C 3.957012 2.543474 0.183156
C 2.590150 3.172595 0.007903
C 1.736180 3.300708 1.107761
C 0.454561 3.818639 0.949184
C 0.011947 4.219198 -0.310354
C 0.861075 4.102448 -1.407527
C 2.142074 3.578086 -1.249235
C -3.971467 0.340330 -0.428740
C -5.220886 0.635339 0.286405
C -5.902418 1.789719 0.224035
C -5.590009 3.025674 -0.542667
O 0.100985 -3.419117 -0.638530
O -2.059717 -2.458109 2.181813
O -3.482020 -0.860056 -0.006859
O 1.488705 -2.164166 1.430001
O 3.272687 0.363105 -0.384623
O -3.426501 1.017944 -1.281065

O 4.025707 0.677293 1.728605
H -1.235044 -4.365506 0.550239
H -0.248315 -2.022815 -2.139058
H 1.454297 -0.089792 1.291397
H 1.906174 -2.992758 -2.714860
H -3.221234 -3.209833 -0.239314
H -2.237654 -3.198302 -1.706306
H -0.552305 0.497699 -1.751052
H 0.542928 1.215744 -0.564023
H 1.196209 0.300304 -1.921949
H -2.856667 -2.057343 1.809059
H -2.080260 -0.820214 -1.524350
H -2.156727 0.141871 1.909894
H -0.433716 0.501361 1.892165
H -1.470602 1.122638 0.606795
H 0.165609 -1.627502 2.944756
H 0.352700 -3.365368 2.598263
H 3.578422 -1.401038 0.670998
H 4.962166 -2.445448 -1.171981
H 4.250462 -2.623777 -2.795138
H 4.720578 -1.022783 -2.182717
H -5.590383 -0.163175 0.923572
H -6.812554 1.836412 0.823366
H -6.363586 3.183669 -1.306516
H -5.646066 3.893178 0.126125
H -4.616920 2.991239 -1.029083
H 4.535000 3.057470 0.955933
H 4.509792 2.575779 -0.760768
H 2.075767 2.984242 2.091843
H -0.201649 3.907141 1.810723
H -0.989804 4.620648 -0.434936
H 0.524144 4.413203 -2.392635
H 2.796806 3.474611 -2.111115

5a-c6 , delta G = 0.4620 kcal/mol, population = 10.33 %
C -1.039286 -3.478180 -0.240595
C -2.307097 -3.187605 -1.054767
C -2.699559 -1.747228 -0.679142
C -1.698638 -1.302657 0.441477
C -0.398981 -0.711962 -0.215058
C 0.711725 -0.597204 0.850760
C 2.043485 -0.108606 0.275715
C 2.374063 -0.618456 -1.096653
C 1.494879 -1.340263 -1.793030
C 0.105517 -1.660262 -1.327065
C -1.268340 -2.656567 1.045461
C -0.082420 -2.530666 1.995038
C -2.329053 -0.337809 1.445338
C -0.682046 0.673265 -0.815231
C 3.750257 -0.293207 -1.593654
C 2.489592 2.013524 1.300282
C 2.614751 3.493828 1.007377
C 3.836053 3.727135 0.143090
C 5.106434 3.761327 0.723747
C 6.239575 3.924433 -0.068302
C 6.114754 4.054583 -1.449820
C 4.850664 4.021627 -2.034208
C 3.717569 3.856124 -1.241883
C -4.882698 -0.740456 -0.403308
C -6.172393 -0.967949 0.263409
C -7.199033 -0.104465 0.302359
C -7.309422 1.256115 -0.288151
O 0.135772 -3.049122 -0.929483
O -2.287872 -3.285191 1.830880
O -4.054104 -1.792800 -0.148447
O 1.035619 -1.859792 1.430965
O 2.071576 1.350575 0.203456
O -4.557658 0.209021 -1.092694
O 2.766841 1.483673 2.355013
H -0.890551 -4.541413 -0.027492
H -0.574088 -1.556424 -2.191962
H 0.404066 0.111884 1.637163
H 1.783245 -1.751813 -2.759040
H -3.107189 -3.876119 -0.767869
H -2.131426 -3.311924 -2.127101
H -1.621622 0.677715 -1.375251
H -0.756931 1.429233 -0.028268
H 0.118450 0.977843 -1.493032
H -3.156155 -3.075815 1.460560
H -2.699423 -1.064675 -1.530820
H -3.123910 -0.828669 2.012030
H -1.595307 0.028185 2.167159
H -2.762379 0.528094 0.938401
H -0.429919 -2.005866 2.900900
H 0.269234 -3.526096 2.287658
H 2.827325 -0.419638 0.978263
H 3.894172 0.792967 -1.653768

H 4.509106 -0.674736 -0.898817
H 3.928355 -0.723613 -2.582358
H -6.267952 -1.923893 0.770501
H -8.080991 -0.442307 0.848086
H -8.123139 1.265530 -1.025663
H -7.605266 1.967823 0.492822
H -6.390570 1.591315 -0.765236
H 1.712680 3.834244 0.489411
H 2.703761 4.015799 1.962979
H 5.206265 3.659572 1.802050
H 7.221858 3.952569 0.395135
H 6.998621 4.184427 -2.067907
H 4.745019 4.125904 -3.110590
H 2.732478 3.826287 -1.701184

5a-c29 , delta G = 0.6165 kcal/mol, population = 7.96 %
C -0.988329 -3.528821 -0.191130
C -2.257071 -3.229076 -0.997975
C -2.626958 -1.783335 -0.631631
C -1.653563 -1.369475 0.524721
C -0.349530 -0.752201 -0.098839
C 0.747312 -0.641689 0.980225
C 2.078043 -0.127155 0.426401
C 2.432878 -0.626148 -0.944346
C 1.571152 -1.352983 -1.657255
C 0.174568 -1.681241 -1.220021
C -1.213505 -2.733155 1.104594
C -0.023420 -2.629870 2.054316
C -2.306843 -0.439568 1.544104
C -0.633000 0.640626 -0.683213
C 3.810053 -0.276900 -1.422384
C 2.525692 2.003472 1.438586
C 2.622781 3.483955 1.130333
C 3.650161 3.720270 0.045246
C 3.263892 3.871239 -1.287751
C 4.221000 4.031750 -2.285971
C 5.575891 4.038970 -1.962114
C 5.968028 3.888998 -0.633665
C 5.010206 3.730891 0.364285
C -4.748530 -0.659539 -0.475783
C -6.101822 -0.828059 0.076669
C -7.046440 0.122768 0.138461
C -6.983301 1.540565 -0.309495
O 0.193760 -3.080759 -0.853175
O -2.264305 -3.420734 1.787807
O -4.010837 -1.765328 -0.198396
O 1.087680 -1.910098 1.537709
O 2.084972 1.330723 0.357952
O -4.319749 0.298126 -1.097455
O 2.827421 1.484450 2.491926
H -0.836652 -4.596183 -0.000241
H -0.488313 -1.560222 -2.094983
H 0.421517 0.048943 1.776503
H 1.877340 -1.754592 -2.622033
H -3.059173 -3.902288 -0.686130
H -2.091389 -3.364306 -2.071123
H -0.695578 1.389099 0.112046
H 0.162972 0.948731 -1.364928
H -1.578847 0.655378 -1.231583
H -2.511193 -2.908591 2.569089
H -2.544912 -1.101696 -1.480088
H -2.658223 0.479344 1.068218
H -3.176216 -0.915973 2.006581
H -1.613509 -0.160437 2.341852
H -0.373560 -2.160238 2.992498
H 0.343029 -3.633992 2.294011
H 2.858068 -0.428996 1.137085
H 4.008477 -0.703191 -2.408988
H 3.934059 0.812231 -1.480944
H 4.566708 -0.644600 -0.717818
H -6.316669 -1.813913 0.479422
H -7.994801 -0.178498 0.585248
H -7.665419 1.684029 -1.158729
H -7.354198 2.193756 0.489387
H -5.982960 1.846933 -0.610135
H 1.638028 3.836119 0.803976
H 2.896149 3.996702 2.055477
H 2.207282 3.858901 -1.544250
H 3.907155 4.151919 -3.319286
H 6.322772 4.164352 -2.740980
H 7.022629 3.897339 -0.372438
H 5.319152 3.613591 1.400741

5a-c27 , delta G = 0.8464 kcal/mol, population = 5.40 %
C -1.324619 -3.120255 0.036495
C -2.436877 -2.531257 -0.839941

C -2.239088 -1.009585 -0.757082
C -1.127660 -0.776728 0.321301
C 0.277799 -0.810795 -0.383151
C 1.393275 -0.915247 0.678141
C 2.794318 -1.028576 0.067549
C 2.873533 -1.857965 -1.180309
C 1.772258 -2.339889 -1.758892
C 0.374217 -2.053265 -1.298501
C -1.183981 -2.069380 1.154950
C 0.006278 -2.224056 2.099446
C -1.349807 0.499561 1.130335
C 0.485536 0.459254 -1.219011
C 4.255061 -2.139146 -1.689576
C 3.803980 1.027026 0.768830
C 4.099474 2.442893 0.320434
C 2.796570 3.148203 0.006093
C 2.474245 3.512742 -1.301024
C 1.245373 4.103418 -1.586621
C 0.323048 4.328617 -0.568213
C 0.640494 3.970189 0.740731
C 1.870536 3.385640 1.026478
C -3.805210 0.807386 -0.857218
C -5.076249 1.358654 -0.360070
C -5.811943 0.985794 0.699046
C -5.594973 -0.098600 1.696644
O -0.096439 -3.272325 -0.675689
O -2.377281 -2.056954 1.946335
O -3.502047 -0.410536 -0.362316
O 1.274565 -2.095820 1.471137
O 3.315686 0.298287 -0.255773
O -3.110855 1.404851 -1.666004
O 3.938075 0.609793 1.899699
H -1.562308 -4.117292 0.426528
H -0.241927 -1.862077 -2.194804
H 1.375363 -0.023559 1.327035
H 1.866424 -2.996067 -2.622964
H -3.413710 -2.793290 -0.426147
H -2.383001 -2.910456 -1.864788
H -0.397923 0.688758 -1.821303
H 0.680682 1.319170 -0.573198
H 1.337628 0.355291 -1.894053
H -2.475238 -2.930726 2.349402
H -1.960959 -0.569938 -1.715759
H -1.424182 1.370334 0.473599
H -2.275133 0.423932 1.705155
H -0.534596 0.683802 1.835081
H -0.104793 -1.472457 2.899675
H -0.007687 -3.220040 2.559480
H 3.446423 -1.465586 0.834817
H 4.229286 -2.786312 -2.569813
H 4.766337 -1.206655 -1.957242
H 4.860681 -2.621677 -0.912143
H -5.403648 2.216094 -0.942377
H -6.712645 1.578363 0.867114
H -4.613930 -0.565023 1.631382
H -5.751397 0.300638 2.705850
H -6.359957 -0.875324 1.557398
H 4.636881 2.941279 1.131636
H 4.737490 2.414712 -0.568530
H 3.185117 3.323037 -2.101587
H 1.005323 4.380352 -2.609471
H -0.639073 4.780393 -0.792536
H -0.073542 4.142614 1.541407
H 2.113007 3.102102 2.048637

5a-c34 , delta G = 0.8691 kcal/mol, population = 5.19 %

C -1.341955 -3.117763 0.021821
C -2.446347 -2.518012 -0.856251
C -2.240784 -0.997589 -0.768885
C -1.128026 -0.774078 0.309580
C 0.277410 -0.810513 -0.392374
C 1.387802 -0.917039 0.672944
C 2.790347 -1.034121 0.066172
C 2.869554 -1.866689 -1.179833
C 1.768403 -2.348701 -1.758811
C 0.369742 -2.055232 -1.304447
C -1.194254 -2.075114 1.141303
C -0.003860 -2.250980 2.081108
C -1.339672 0.499600 1.124582
C 0.493980 0.455456 -1.233299
C 4.251347 -2.151863 -1.686211
C 3.801131 1.019981 0.768582
C 4.096464 2.436248 0.321872
C 2.793098 3.141139 0.008306
C 2.471788 3.508916 -1.298208
C 1.242414 4.098604 -1.583669
C 0.318577 4.319807 -0.565748

C 0.635134 3.958422 0.742566
C 1.865531 3.374525 1.028241
C -3.790000 0.835643 -0.840054
C -5.058859 1.390552 -0.340879
C -5.817039 0.996564 0.694457
C -5.638326 -0.124227 1.658509
O -0.110320 -3.270620 -0.682237
O -2.390163 -2.185082 1.919002
O -3.502032 -0.396010 -0.370128
O 1.266607 -2.098994 1.463510
O 3.316245 0.290795 -0.257466
O -3.082087 1.444259 -1.628247
O 3.929100 0.603677 1.900525
H -1.588177 -4.110937 0.411451
H -0.241750 -1.861368 -2.203527
H 1.369313 -0.025333 1.321934
H 1.862887 -3.008485 -2.620072
H -3.424603 -2.777636 -0.444351
H -2.392700 -2.893761 -1.882411
H 1.341206 0.339770 -1.912677
H -0.390326 0.692686 -1.831295
H 0.703612 1.316090 -0.593037
H -2.391723 -1.479775 2.579839
H -1.962173 -0.555629 -1.726379
H -1.334909 1.383154 0.481384
H -2.306773 0.477573 1.636213
H -0.563454 0.635931 1.882828
H -0.112341 -1.523622 2.907234
H -0.020611 -3.258395 2.510953
H 3.439388 -1.472264 0.835363
H 4.857210 -2.627267 -0.904638
H 4.225900 -2.806421 -2.560964
H 4.762247 -1.221483 -1.961983
H -5.363102 2.272399 -0.898760
H -6.708375 1.600975 0.870226
H -6.445506 -0.855755 1.515384
H -4.684128 -0.638207 1.561344
H -5.755237 0.255427 2.680908
H 4.633192 2.933650 1.134141
H 4.734473 2.410001 -0.567103
H 3.183899 3.322444 -2.098406
H 1.003222 4.378080 -2.606015
H -0.643762 4.771226 -0.789855
H -0.079799 4.128501 1.542991
H 2.107397 3.088627 2.049894

5a-c30 , delta G = 1.0391 kcal/mol, population = 3.90 %

C -1.063952 -3.414920 -0.574101
C -2.327297 -2.927279 -1.291366
C -2.640980 -1.562925 -0.656792
C -1.644504 -1.405243 0.542622
C -0.323617 -0.727414 0.027524
C 0.785833 -0.857770 1.092087
C 2.131597 -0.297797 0.629552
C 2.455843 -0.538434 -0.818465
C 1.561710 -1.088459 -1.642102
C 0.158573 -1.448211 -1.254357
C -1.250242 -2.868639 0.850268
C -0.049376 -2.986176 1.784545
C -2.257692 -0.664472 1.728058
C -0.562042 0.757214 -0.288578
C 3.834751 -0.143713 -1.254893
C 3.210500 1.666891 1.524478
C 3.163109 3.179695 1.464890
C 3.657735 3.605398 0.097747
C 5.026221 3.758297 -0.135936
C 5.492364 4.078802 -1.408230
C 4.594790 4.247011 -2.460181
C 3.228354 4.096867 -2.232556
C 2.762403 3.776359 -0.960581
C -4.720367 -0.414259 -0.284130
C -6.077449 -0.632532 0.241116
C -7.021861 0.307819 0.397451
C -6.960504 1.764216 0.099285
O 0.128565 -2.891470 -1.157534
O -2.320050 -3.635637 1.408470
O -4.020124 -1.575518 -0.208365
O 1.082658 -2.220792 1.396131
O 2.150630 1.144309 0.874259
O -4.258619 0.621608 -0.732855
O 4.103416 1.020004 2.027623
H -0.949017 -4.503649 -0.587546
H -0.504407 -1.140938 -2.082647
H 0.494427 -0.318621 2.009599
H 1.844804 -1.305618 -2.671033
H -3.151138 -3.618487 -1.098033
H -2.175813 -2.865075 -2.373279

H -1.488526 0.894371 -0.852588
H -0.638829 1.345235 0.630338
H 0.259748 1.168668 -0.878762
H -2.535397 -3.276291 2.279149
H -2.542693 -0.740570 -1.367680
H -1.548909 -0.559105 2.553868
H -2.587100 0.336712 1.438875
H -3.136528 -1.196172 2.103549
H -0.375448 -2.689543 2.799112
H 0.282337 -4.029354 1.826179
H 2.908758 -0.751188 1.256341
H 4.595146 -0.639195 -0.638953
H 4.003739 -0.398919 -2.304043
H 3.985123 0.937780 -1.137515
H -6.299975 -1.656097 0.529268
H -7.972238 -0.038212 0.806169
H -7.725826 2.014644 -0.647238
H -7.225254 2.330807 1.001190
H -5.985698 2.083990 -0.263533
H 2.136043 3.521291 1.623140
H 3.808594 3.569985 2.254813
H 5.728499 3.621912 0.683122
H 6.558944 4.197950 -1.577494
H 4.958226 4.497331 -3.452864
H 2.522192 4.230662 -3.047404
H 1.696264 3.653247 -0.786258

5a-c32 , delta G = 1.0823 kcal/mol, population = 3.62 %

C -1.052391 -3.420702 -0.574160
C -2.313957 -2.937329 -1.298688
C -2.632059 -1.572648 -0.666917
C -1.642573 -1.413907 0.538748
C -0.318463 -0.738220 0.026401
C 0.790237 -0.868022 1.092674
C 2.136563 -0.307100 0.633376
C 2.463423 -0.543115 -0.814812
C 1.571434 -1.093159 -1.640682
C 0.168613 -1.456461 -1.255391
C -1.249284 -2.869569 0.851207
C -0.058091 -2.976454 1.800488
C -2.263191 -0.672392 1.719941
C -0.558534 0.746347 -0.287239
C 3.841357 -0.143205 -1.249476
C 3.215043 1.657368 1.529944
C 3.161332 3.170260 1.478813
C 3.638801 3.607101 0.109151
C 5.003497 3.772623 -0.137531
C 5.453931 4.105000 -1.412494
C 4.544312 4.272481 -2.454099
C 3.181526 4.109659 -2.213484
C 2.731323 3.777396 -0.938912
C -4.714934 -0.424197 -0.317586
C -6.072846 -0.638913 0.207038
C -7.005743 0.308989 0.384308
C -6.925937 1.770386 0.115370
O 0.141604 -2.900185 -1.160054
O -2.362612 -3.526389 1.465606
O -4.013285 -1.583055 -0.227747
O 1.083575 -2.231583 1.398602
O 2.153663 1.134245 0.882704
O -4.255445 0.607916 -0.777414
O 4.112755 1.011097 2.025279
H -0.934230 -4.510864 -0.589430
H -0.493238 -1.150272 -2.084939
H 0.496632 -0.329402 2.009655
H 1.855986 -1.306796 -2.669976
H -3.139688 -3.627569 -1.109658
H -2.156194 -2.876637 -2.379736
H 0.265743 1.161364 -0.871337
H -1.482216 0.883596 -0.855798
H -0.642166 1.330839 0.633320
H -2.158022 -4.469988 1.519373
H -2.528238 -0.751052 -1.377902
H -2.620157 0.315914 1.418936
H -3.110637 -1.236253 2.113714
H -1.545603 -0.537205 2.533320
H -0.397550 -2.650799 2.798723
H 0.269398 -4.021072 1.873290
H 2.913054 -0.761779 1.260119
H 4.012247 -0.397023 -2.298666
H 3.987507 0.938810 -1.131388
H 4.603128 -0.635951 -0.633095
H -6.303999 -1.664679 0.480113
H -7.959361 -0.033693 0.788145
H -5.931251 2.093786 -0.185392
H -7.641361 2.033657 -0.675472
H -7.246458 2.323223 1.007010

H 2.134703 3.506703 1.650522
H 3.813756 3.558522 2.264000
H 5.715291 3.637124 0.673426
H 6.517712 4.234089 -1.591824
H 4.895466 4.532213 -3.448783
H 2.465869 4.242907 -3.020102
H 1.668073 3.644766 -0.754325

5a-c4 , delta G = 1.1462 kcal/mol, population = 3.25 %

C -1.066299 -3.361114 -0.589468
C -2.296251 -2.862214 -1.358642
C -2.645090 -1.506909 -0.716908
C -1.659941 -1.331166 0.489177
C -0.319882 -0.681917 -0.013499
C 0.765739 -0.821554 1.075858
C 2.131243 -0.292137 0.635545
C 2.479247 -0.538841 -0.805872
C 1.591311 -1.073076 -1.646076
C 0.179943 -1.420525 -1.277273
C -1.299270 -2.794303 0.826324
C -0.135193 -2.906226 1.804325
C -2.278589 -0.553445 1.649646
C -0.532771 0.802140 -0.347108
C 3.872207 -0.166260 -1.216175
C 3.226958 1.655762 1.548020
C 3.208887 3.168890 1.482556
C 3.738636 3.580747 0.124388
C 5.114391 3.702480 -0.083440
C 5.611752 4.008889 -1.347341
C 4.738466 4.194034 -2.416732
C 3.364918 4.075241 -2.214892
C 2.867723 3.768808 -0.951299
C -4.792161 -0.476607 -0.295736
C -6.108169 -0.769197 0.289104
C -7.107050 0.111686 0.455124
C -7.156240 1.557348 0.109359
O 0.145326 -2.860024 -1.156066
O -2.362217 -3.518425 1.456513
O -4.014641 -1.595309 -0.234126
O 1.022524 -2.186738 1.405321
O 2.171794 1.150390 0.876562
O -4.408714 0.571632 -0.781925
O 4.096664 0.994042 2.071924
H -0.966428 -4.451067 -0.583676
H -0.470505 -1.122177 -2.119370
H 0.468385 -0.263767 1.979772
H 1.888012 -1.293773 -2.670336
H -3.130952 -3.558162 -1.233183
H -2.090996 -2.780997 -2.429853
H 0.312652 1.201923 -0.911247
H -1.436615 0.944043 -0.946355
H -0.636930 1.397086 0.564493
H -3.211782 -3.211422 1.112047
H -2.594337 -0.673838 -1.419739
H -2.653523 0.417424 1.315874
H -3.113990 -1.105250 2.086815
H -1.552580 -0.378327 2.446958
H -0.487708 -2.555266 2.789055
H 0.170409 -3.953718 1.902409
H 2.887225 -0.758614 1.278440
H 4.037626 0.912852 -1.097550
H 4.612302 -0.672936 -0.584827
H 4.057463 -0.426229 -2.261401
H -6.249944 -1.795478 0.615866
H -8.015077 -0.283176 0.912710
H -6.222215 1.929869 -0.306348
H -7.968429 1.730188 -0.609140
H -7.420889 2.135021 1.004227
H 2.185411 3.529950 1.619585
H 3.845580 3.549656 2.284226
H 5.797685 3.552663 0.749227
H 6.683764 4.103709 -1.496459
H 5.126379 4.433212 -3.402879
H 2.677585 4.222636 -3.043389
H 1.795933 3.670271 -0.797167

5a-c7 , delta G = 1.1936 kcal/mol, population = 3.00 %

C -1.355913 -3.044052 0.020673
C -2.448913 -2.429785 -0.864678
C -2.249245 -0.907820 -0.746009
C -1.111751 -0.701045 0.309006
C 0.285501 -0.769822 -0.407640
C 1.400333 -0.907199 0.651427
C 2.796149 -1.057899 0.036623
C 2.851551 -1.875759 -1.219926
C 1.736970 -2.322565 -1.800791

C 0.348982 -2.010876 -1.326099
C -1.201815 -1.996507 1.143163
C -0.016992 -2.174694 2.086648
C -1.284051 0.586496 1.114993
C 0.519999 0.497155 -1.240919
C 4.224382 -2.186555 -1.735351
C 3.866340 0.961111 0.754521
C 4.191610 2.374994 0.321869
C 2.902811 3.114872 0.029632
C 1.989901 3.355208 1.061082
C 0.772992 3.975584 0.795554
C 0.455916 4.367482 -0.503889
C 1.364986 4.138359 -1.533337
C 2.580413 3.511506 -1.268095
C -3.879748 0.874288 -0.699242
C -5.114812 1.380143 -0.083282
C -5.985275 0.747459 0.719279
C -5.986433 -0.658082 1.220558
O -0.134253 -3.219680 -0.697576
O -2.351141 -2.051950 1.997364
O -3.498101 -0.344178 -0.246760
O 1.247473 -2.082546 1.445859
O 3.352756 0.257472 -0.275550
O -3.252678 1.507667 -1.531931
O 3.995901 0.527361 1.879556
H -1.617068 -4.031383 0.414486
H -0.273903 -1.805496 -2.215361
H 1.412145 -0.015124 1.299724
H 1.810981 -2.971019 -2.672510
H -3.438456 -2.703371 -0.487417
H -2.376248 -2.785536 -1.896312
H 1.373655 0.377972 -1.911331
H -0.355563 0.740599 -1.850016
H 0.726773 1.353549 -0.594053
H -3.078430 -1.565159 1.586902
H -2.023469 -0.425649 -1.697601
H -2.178622 0.539980 1.741317
H -0.433609 0.766707 1.776836
H -1.380091 1.451505 0.453638
H -0.104095 -1.414095 2.880798
H -0.056420 -3.165078 2.553399
H 3.436652 -1.520126 0.798821
H 4.179889 -2.818094 -2.626102
H 4.761606 -1.264508 -1.987645
H 4.816280 -2.699206 -0.966639
H -5.310163 2.415099 -0.350590
H -6.823633 1.354975 1.061368
H -5.473681 -0.704686 2.192121
H -7.013609 -0.992275 1.392532
H -5.483795 -1.352912 0.547572
H 4.747700 2.849741 1.134664
H 4.820480 2.344166 -0.573466
H 2.232641 3.046732 2.075922
H 0.069293 4.150872 1.604730
H -0.495600 4.848464 -0.712245
H 1.125151 4.441127 -2.548902
H 3.280967 3.319762 -2.077221

5b

5b-c6 , delta G = 0.0000 kcal/mol, population = 25.77 %

C 1.825477 -0.720878 -2.608792
C 3.111222 -1.391825 -2.109816
C 3.283705 -0.895347 -0.664005
C 2.155901 0.168556 -0.431651
C 0.853171 -0.556979 0.060751
C -0.337426 0.421603 -0.028740
C -1.607674 -0.257725 0.471925
C -1.855595 -1.613291 -0.129964
C -0.852529 -2.296884 -0.684197
C 0.541274 -1.767650 -0.855871
C 1.835430 0.633111 -1.870274
C 0.553287 1.458999 -1.960165
C 2.592087 1.289918 0.509984
C 1.011320 -1.042948 1.509803
C -3.236417 -2.174295 0.029079
C -3.100235 1.504153 1.095326
C -4.368500 2.221059 0.680660
C -5.540898 1.265013 0.713155
C -6.004178 0.659782 -0.456274
C -7.054452 -0.253276 -0.409840
C -7.649875 -0.573850 0.807995
C -7.191853 0.026575 1.978976
C -6.143414 0.941198 1.931487
C 5.277254 -0.399170 0.605625
C 6.548788 0.331029 0.507185
C 7.447916 0.478187 1.492677
C 7.406690 -0.031251 2.889371

O 0.668005 -1.482036 -2.268095
O 2.843404 1.477418 -2.436886
O 4.596074 -0.276709 -0.569037
O -0.578165 0.834513 -1.369212
O -2.760271 0.579217 0.178848
O 4.854305 -1.034235 1.554524
O -2.486899 1.708121 2.122363
H 1.793112 -0.595220 -3.695758
H 1.252694 -2.573166 -0.598964
H -0.150241 1.303346 0.605982
H -1.035257 -3.299228 -1.068077
H 3.964845 -1.073303 -2.715651
H 3.044620 -2.481482 -2.176970
H 1.988829 -1.505119 1.672374
H 0.918571 -0.208445 2.212097
H 0.248400 -1.785964 1.759764
H 3.713144 1.199458 -2.119637
H 3.242014 -1.698925 0.072584
H 3.424317 1.853082 0.081393
H 1.784115 2.000689 0.699133
H 2.919472 0.887465 1.472068
H 0.748618 2.435337 -1.484805
H 0.297691 1.634136 -3.010969
H -1.545872 -0.353821 1.564423
H -3.553456 -2.130281 1.079468
H -3.965935 -1.587025 -0.539612
H -3.277685 -3.212463 -0.309365
H 6.744620 0.788203 -0.458601
H 8.337370 1.056807 1.239907
H 6.487349 -0.564533 3.122560
H 8.264787 -0.695354 3.058041
H 7.536450 0.807610 3.585029
H -4.517643 3.051943 1.374248
H -4.231317 2.617163 -0.330923
H -5.537136 0.904445 -1.407170
H -7.408622 -0.714681 -1.327639
H -8.469771 -1.285623 0.844177
H -7.654058 -0.215143 2.932123
H -5.788159 1.409670 2.846738

5b-c24 , delta G = 0.0612 kcal/mol, population = 23.23 %

C 1.734900 -0.406178 -2.615575
C 3.054444 -1.081631 -2.229226
C 3.248093 -0.748819 -0.741940
C 2.123445 0.280297 -0.378791
C 0.849483 -0.503786 0.097760
C -0.357681 0.456271 0.148745
C -1.595353 -0.290741 0.634048
C -1.850192 -1.582520 -0.093920
C -0.862606 -2.188510 -0.755569
C 0.519683 -1.627835 -0.918993
C 1.734710 0.867741 -1.756679
C 0.428031 1.661973 -1.733805
C 2.584157 1.322959 0.636858
C 1.068941 -1.123866 1.487085
C -3.219087 -2.175755 0.054197
C -3.101057 1.367126 1.480413
C -4.399188 2.090044 1.178845
C -5.486459 1.136155 0.744227
C -5.818525 0.986175 -0.602787
C -6.785851 0.063705 -0.993542
C -7.429780 -0.721529 -0.040137
C -7.104695 -0.575380 1.307018
C -6.139799 0.349223 1.696302
C 5.238458 -0.461907 0.577590
C 6.544928 0.214618 0.555332
C 7.466332 0.181742 1.530338
C 7.425758 -0.517041 2.843223
O 0.601229 -1.212294 -2.303370
O 2.750517 1.702620 -2.317580
O 4.572001 -0.182707 -0.572734
O -0.668189 0.982175 -1.137227
O -2.773614 0.543734 0.469562
O 4.787384 -1.172793 1.459860
O -2.446475 1.502971 2.493123
H 1.657671 -0.183812 -3.684938
H 1.244759 -2.446079 -0.760646
H -0.156245 1.280936 0.852537
H -1.050044 -3.147019 -1.236897
H 3.873794 -0.651153 -2.810095
H 3.023885 -2.158533 -2.420738
H 0.970783 -0.366703 2.271701
H 0.337722 -1.913642 1.683546
H 2.065953 -1.563510 1.573981
H 2.866688 2.472073 -1.744819
H 3.193412 -1.634347 -0.106192
H 2.896572 0.848931 1.570932

H 3.444151 1.880768 0.255754
H 1.794877 2.041620 0.873117
H 0.617808 2.614318 -1.203762
H 0.126214 1.898644 -2.759815
H -1.486928 -0.491785 1.708446
H -3.261267 -3.173726 -0.388823
H -3.498000 -2.245192 1.113908
H -3.975143 -1.543083 -0.425216
H 6.749953 0.787355 -0.344720
H 8.379228 0.747280 1.338696
H 7.579263 0.213157 3.648276
H 6.495623 -1.056204 3.010598
H 8.269975 -1.216026 2.907777
H -4.681837 2.636394 2.082331
H -4.192886 2.819952 0.386623
H -5.310499 1.590820 -1.350176
H -7.036075 -0.042223 -2.045604
H -8.183888 -1.441734 -0.344735
H -7.605401 -1.181282 2.057225
H -5.887395 0.460959 2.748828

5b-c23 , delta G = 0.2776 kcal/mol, population = 16.12 %
C 1.911856 -0.794892 -2.666584
C 3.211729 -1.396459 -2.122244
C 3.322626 -0.865042 -0.685102
C 2.173325 0.188595 -0.523836
C 0.878906 -0.543313 -0.017708
C -0.334148 0.400011 -0.160772
C -1.594350 -0.290919 0.351458
C -1.797886 -1.673454 -0.205013
C -0.771205 -2.349625 -0.724445
C 0.613807 -1.796559 -0.890908
C 1.858341 0.582515 -1.986941
C 0.549281 1.357199 -2.140098
C 2.571856 1.362113 0.367869
C 1.021743 -0.968503 1.452503
C -3.165069 -2.266521 -0.043512
C -3.101905 1.496031 0.854321
C -4.382135 2.168358 0.404607
C -5.560870 1.283739 0.751496
C -6.106294 0.417626 -0.198070
C -7.161211 -0.425712 0.140336
C -7.680354 -0.413071 1.433023
C -7.140300 0.449061 2.385004
C -6.085643 1.292235 2.046214
C 5.230732 -0.357788 0.689141
C 6.533004 0.325516 0.640709
C 7.373517 0.492978 1.673142
C 7.227875 0.056382 3.088036
O 0.767185 -1.566976 -2.312022
O 2.899545 1.346161 -2.600210
O 4.632652 -0.264409 -0.526772
O -0.575747 0.750258 -1.518967
O -2.764589 0.504299 0.009429
O 4.731827 -0.933654 1.641320
O -2.484843 1.778090 1.860632
H 1.893598 -0.717615 -3.758638
H 1.335569 -2.575481 -0.587028
H -0.173217 1.310704 0.439106
H -0.925561 -3.368741 -1.075272
H 4.059199 -1.036430 -2.710645
H 3.197280 -2.489585 -2.169517
H 0.278584 -1.727514 1.713846
H 2.011054 -1.387305 1.655182
H 0.881953 -0.111543 2.119218
H 2.964974 2.197460 -2.147665
H 3.237874 -1.656432 0.061704
H 3.456155 1.865869 -0.032429
H 1.772403 2.103202 0.451452
H 2.823168 1.022058 1.375699
H 0.704790 2.373730 -1.731741
H 0.303561 1.452441 -3.203294
H -1.547112 -0.344659 1.447515
H -3.171061 -3.318667 -0.338433
H -3.501010 -2.187657 0.998752
H -3.900759 -1.725851 -0.648827
H 6.805588 0.726514 -0.331466
H 8.297365 1.028488 1.450179
H 8.045860 -0.631088 3.341343
H 7.349311 0.923215 3.750028
H 6.275154 -0.429734 3.288186
H -4.450126 3.128760 0.921028
H -4.339157 2.336711 -0.675598
H -5.701979 0.405945 -1.207310
H -7.580239 -1.091958 -0.608769
H -8.505052 -1.069336 1.696368
H -7.542969 0.467975 3.393955

H -5.666152 1.965189 2.790559

5b-c9 , delta G = 0.3461 kcal/mol, population = 14.36 %

C 1.186691 -0.320922 -2.574624
C 2.683155 -0.561378 -2.350678
C 2.886259 -0.402289 -0.836390
C 1.525375 0.130637 -0.271421
C 0.622468 -1.094713 0.115400
C -0.820082 -0.611117 0.363379
C -1.705911 -1.787323 0.766290
C -1.592846 -2.969623 -0.155543
C -0.521989 -3.112417 -0.939174
C 0.586967 -2.108218 -1.058051
C 0.847288 0.744636 -1.520316
C -0.639571 1.042572 -1.321921
C 1.708794 1.111548 0.883138
C 1.153059 -1.793696 1.377104
C -2.677355 -4.000110 -0.064258
C -3.499511 -0.646905 1.851326
C -4.849413 -0.013811 1.596237
C -4.630780 1.206804 0.723821
C -4.039539 2.352828 1.262642
C -3.769725 3.453196 0.454355
C -4.087902 3.419797 -0.901958
C -4.680256 2.281485 -1.443270
C -4.948142 1.179753 -0.634651
C 4.780799 0.337019 0.447769
C 5.786127 1.409889 0.499777
C 6.720668 1.568287 1.449546
C 6.988915 0.741091 2.656892
O 0.410432 -1.497731 -2.359498
O 1.484613 1.941184 -1.973126
O 3.965456 0.540938 -0.619223
O -1.393657 -0.046764 -0.810026
O -3.096110 -1.354636 0.778823
O 4.664636 -0.605494 1.213055
O -2.837097 -0.489306 2.856048
H 0.947963 0.011631 -3.590182
H 1.548726 -2.651337 -1.060281
H -0.835987 0.128739 1.180385
H -0.431860 -3.999656 -1.563754
H 3.262013 0.200466 -2.878675
H 2.990125 -1.545290 -2.718131
H 0.741721 -2.803458 1.470640
H 2.242907 -1.878675 1.357627
H 0.880966 -1.231886 2.276493
H 1.358287 2.627590 -1.304838
H 3.172470 -1.337401 -0.351900
H 0.751114 1.474472 1.265182
H 2.248015 0.647693 1.713308
H 2.293918 1.978342 0.563136
H -0.730962 1.915756 -0.647675
H -1.092583 1.309642 -2.283002
H -1.448049 -2.095806 1.788286
H -2.826818 -4.313513 0.977080
H -3.633356 -3.592622 -0.410765
H -2.432295 -4.879009 -0.665527
H 5.735238 2.127627 -0.314099
H 7.381824 2.426926 1.324701
H 6.977526 1.382490 3.547255
H 6.279883 -0.075140 2.781322
H 8.005743 0.330798 2.596046
H -5.506132 -0.728070 1.091810
H -5.279853 0.263087 2.561991
H -3.786212 2.379023 2.320114
H -3.310951 4.339020 0.885084
H -3.876959 4.278289 -1.533485
H -4.932675 2.248543 -2.499564
H -5.398752 0.287910 -1.062843

5b-c12 , delta G = 0.6356 kcal/mol, population = 8.80 %

C 1.170770 -0.334528 -2.577264
C 2.669089 -0.574832 -2.361285
C 2.879705 -0.405187 -0.849239
C 1.522522 0.135739 -0.281812
C 0.618839 -1.088082 0.113509
C -0.824133 -0.604903 0.363044
C -1.708385 -1.778841 0.775385
C -1.597536 -2.968046 -0.137327
C -0.527663 -3.116973 -0.921021
C 0.579457 -2.112295 -1.051162
C 0.840772 0.741065 -1.524348
C -0.642340 1.050335 -1.309744
C 1.714317 1.123755 0.865386
C 1.153928 -1.775521 1.379232
C -2.681604 -3.998004 -0.035903

C -3.501217 -0.639222 1.862547
C -4.851491 -0.006350 1.608877
C -4.634047 1.214321 0.736213
C -4.958314 1.189405 -0.620620
C -4.692237 2.291607 -1.429303
C -4.094296 3.427837 -0.889790
C -3.768691 3.458828 0.464823
C -4.037209 2.358333 1.273216
C 4.787730 0.327429 0.416876
C 5.792124 1.401450 0.464233
C 6.738753 1.553957 1.402841
C 7.024776 0.717206 2.599599
O 0.396875 -1.512956 -2.357035
O 1.506290 1.961077 -1.864863
O 3.962541 0.534829 -0.641576
O -1.399388 -0.044192 -0.811443
O -3.098404 -1.345886 0.788949
O 4.680382 -0.618465 1.179424
O -2.838426 -0.482771 2.867081
H 0.927210 -0.011461 -3.596616
H 1.541852 -2.654195 -1.052957
H -0.837766 0.139201 1.176199
H -0.437695 -4.009432 -1.538188
H 3.248479 0.181588 -2.896443
H 2.971543 -1.562000 -2.723581
H 2.243415 -1.864004 1.355381
H 0.888401 -1.202998 2.273751
H 0.740208 -2.783025 1.485459
H 1.171987 2.252421 -2.724318
H 3.164094 -1.338269 -0.359843
H 2.260315 2.001966 0.516373
H 0.757614 1.465287 1.268236
H 2.281872 0.670770 1.682739
H -0.712262 1.911458 -0.622009
H -1.106839 1.336106 -2.261927
H -1.447582 -2.080026 1.798813
H -3.638272 -3.593358 -0.383933
H -2.437463 -4.881593 -0.630656
H -2.829235 -4.303132 1.008140
H 5.728415 2.126259 -0.342421
H 7.396130 2.415315 1.276775
H 8.043733 0.314594 2.525073
H 7.017477 1.350157 3.496076
H 6.322164 -0.104639 2.723401
H -5.508709 -0.720712 1.105243
H -5.280833 0.270641 2.575094
H -5.413355 0.299137 -1.047422
H -4.950757 2.260672 -2.484208
H -3.884635 4.286713 -1.521228
H -3.304622 4.342613 0.893976
H -3.777891 2.382597 2.329266

5b-c1 , delta G = 1.1379 kcal/mol, population = 3.77 %
C 1.264425 -0.555333 -2.593379
C 2.748638 -0.821518 -2.314709
C 2.925435 -0.546653 -0.811294
C 1.551454 0.012265 -0.305508
C 0.621413 -1.184524 0.106015
C -0.822331 -0.667782 0.276963
C -1.741226 -1.808389 0.704008
C -1.613111 -3.042098 -0.144874
C -0.514015 -3.244889 -0.874085
C 0.608011 -2.257750 -1.013013
C 0.926525 0.577285 -1.601569
C -0.554261 0.920866 -1.452017
C 1.711691 1.037454 0.815646
C 1.102061 -1.821576 1.418673
C -2.715542 -4.051532 -0.035910
C -3.573137 -0.641884 1.695873
C -4.921220 -0.025519 1.392519
C -4.675709 1.315921 0.730108
C -4.416378 2.446224 1.508979
C -4.130474 3.667607 0.905283
C -4.097305 3.770505 -0.483775
C -4.354879 2.646419 -1.265274
C -4.642002 1.425313 -0.661423
C 4.799213 0.375971 0.403608
C 5.732592 1.510925 0.384524
C 6.670057 1.771308 1.309180
C 7.007321 1.018170 2.546599
O 0.468623 -1.713548 -2.345688
O 1.533437 1.795406 -2.046620
O 3.963971 0.461504 -0.670779
O -1.338012 -0.148342 -0.942801
O -3.123576 -1.354093 0.644426
O 4.742703 -0.528163 1.217276
O -2.950368 -0.469371 2.723077

H 1.059788 -0.267688 -3.629598
H 1.565566 -2.806461 -0.958885
H -0.857541 0.109688 1.057405
H -0.411278 -4.168538 -1.441218
H 3.373404 -0.134646 -2.893489
H 3.030379 -1.840976 -2.593167
H 0.666966 -2.815916 1.558240
H 2.189936 -1.931943 1.432281
H 0.820255 -1.203377 2.276933
H 2.478247 1.778709 -1.843029
H 3.240628 -1.427673 -0.249449
H 0.744910 1.375584 1.195126
H 2.275171 0.617800 1.653071
H 2.246799 1.920433 0.458709
H -0.634437 1.805414 -0.796667
H -0.977444 1.179148 -2.429068
H -1.525320 -2.064804 1.749915
H -2.458593 -4.969247 -0.570531
H -2.913312 -4.297008 1.015523
H -3.650041 -3.654978 -0.447688
H 5.623510 2.184375 -0.460883
H 7.273155 2.663163 1.134173
H 6.364569 0.155535 2.709153
H 8.053590 0.688876 2.495989
H 6.947628 1.693371 3.409792
H -5.491361 -0.681544 0.729590
H -5.454892 0.101288 2.337725
H -4.438560 2.366514 2.593219
H -3.934862 4.540909 1.521396
H -3.874012 4.723372 -0.955398
H -4.332198 2.719331 -2.349150
H -4.834394 0.547623 -1.273334

5b-c26 , delta G = 1.2521 kcal/mol, population = 3.11 %
C 1.385634 -0.271204 -2.341608
C 2.847112 -0.644501 -2.070888
C 2.976683 -0.643317 -0.540426
C 1.623925 -0.073805 0.008324
C 0.619125 -1.264902 0.215206
C -0.801368 -0.706509 0.435957
C -1.788635 -1.847934 0.666685
C -1.694981 -2.943573 -0.358718
C -0.591907 -3.083183 -1.096665
C 0.584646 -2.152181 -1.055946
C 1.063643 0.708300 -1.195459
C -0.405451 1.101155 -1.029730
C 1.803874 0.768521 1.268547
C 1.025886 -2.122488 1.423555
C -2.844673 -3.902933 -0.425358
C -3.574865 -0.678951 1.738221
C -4.868490 0.054722 1.458314
C -4.537271 1.303697 0.666355
C -4.785495 1.366509 -0.705385
C -4.414652 2.490835 -1.438406
C -3.787400 3.562276 -0.807383
C -3.538212 3.506162 0.562581
C -3.911353 2.383325 1.295257
C 4.828758 -0.126891 0.898331
C 5.904284 0.827906 1.212057
C 6.073741 2.089033 0.784328
C 5.251147 2.917282 -0.140857
O 0.522049 -1.405226 -2.295098
O 1.830431 1.905768 -1.360312
O 4.099305 0.205918 -0.186897
O -1.262425 0.017031 -0.699866
O -3.145610 -1.320682 0.634928
O 4.642350 -1.138157 1.558196
O -2.971473 -0.647932 2.790708
H 1.229452 0.176483 -3.330523
H 1.505989 -2.761712 -1.066197
H -0.815203 -0.053375 1.324096
H -0.521119 -3.911526 -1.799675
H 3.509893 0.115696 -2.491694
H 3.102237 -1.610065 -2.517780
H 0.537578 -3.101027 1.391282
H 2.106014 -2.289956 1.449884
H 0.743658 -1.632522 2.360901
H 1.563635 2.316323 -2.194235
H 3.181741 -1.635769 -0.135912
H 2.268460 0.183914 2.066727
H 2.441085 1.630200 1.060628
H 0.848600 1.147040 1.640531
H -0.459876 1.884805 -0.254116
H -0.789543 1.521972 -1.967577
H -1.621393 -2.268231 1.667639
H -3.072345 -4.303517 0.570991
H -3.752493 -3.401419 -0.777992

H -2.620477 -4.735327 -1.096949
H 6.613750 0.423209 1.929196
H 6.950353 2.599570 1.186294
H 4.292812 2.469518 -0.396646
H 5.093547 3.907640 0.302706
H 5.814638 3.088616 -1.068744
H -5.542845 -0.589419 0.886620
H -5.326892 0.305183 2.418329
H -5.263365 0.526338 -1.202928
H -4.614590 2.528048 -2.505771
H -3.496219 4.438676 -1.379662
H -3.052478 4.339488 1.062794
H -3.711790 2.339791 2.363674

5b-c22 , delta G = 1.5137 kcal/mol, population = 2.00 %
C 1.968860 -0.118814 -2.263023
C 3.192047 -0.976543 -1.916136
C 3.272012 -0.951223 -0.379641
C 2.157577 0.040746 0.098937
C 0.800673 -0.733188 0.256124
C -0.350340 0.286034 0.397708
C -1.674779 -0.447393 0.578840
C -1.914998 -1.537416 -0.429540
C -0.892454 -2.070591 -1.100370
C 0.525403 -1.587071 -1.007757
C 1.963287 0.940738 -1.141835
C 0.717389 1.819963 -1.055297
C 2.548372 0.792837 1.370609
C 0.831316 -1.647753 1.490599
C -3.322828 -2.038471 -0.549723
C -3.170200 1.115232 1.604613
C -4.383927 1.983473 1.336703
C -5.465376 1.226913 0.602523
C -6.263826 0.312430 1.294482
C -7.225479 -0.436215 0.622102
C -7.401835 -0.276637 -0.750943
C -6.612724 0.637148 -1.445550
C -5.648966 1.383332 -0.771907
C 5.196878 -0.942962 1.076232
C 6.483941 -0.307821 1.392195
C 7.183947 0.593337 0.684917
C 6.893144 1.214105 -0.640814
O 0.767701 -0.887465 -2.250027
O 3.039623 1.861275 -1.355533
O 4.587095 -0.435449 -0.021879
O -0.474492 1.103944 -0.761091
O -2.780177 0.489918 0.480128
O 4.720732 -1.837991 1.754304
O -2.624000 0.992080 2.681090
H 2.023548 0.336691 -3.256906
H 1.190848 -2.468491 -0.967557
H -0.187661 0.918333 1.285814
H -1.074011 -2.893347 -1.789858
H 4.098157 -0.536174 -2.342625
H 3.095445 -1.990106 -2.315550
H 0.032335 -2.393496 1.444866
H 1.780716 -2.184812 1.568130
H 0.703125 -1.066195 2.408932
H 3.867226 1.463632 -1.053159
H 3.169055 -1.939259 0.070988
H 1.745947 1.449413 1.714002
H 2.784415 0.096015 2.178980
H 3.427800 1.418184 1.199492
H 0.902368 2.594031 -0.290953
H 0.549337 2.320422 -2.015302
H -1.701201 -0.876516 1.589900
H -3.730075 -2.291577 0.438008
H -3.978538 -1.265167 -0.966532
H -3.369441 -2.923340 -1.189262
H 6.884777 -0.648343 2.343048
H 8.109518 0.928970 1.153478
H 7.827707 1.489523 -1.137653
H 6.307955 0.568325 -1.295776
H 6.326277 2.145205 -0.497087
H -4.736646 2.353033 2.302980
H -4.048754 2.842166 0.742453
H -6.127156 0.185131 2.366424
H -7.840053 -1.144036 1.171595
H -8.153349 -0.859437 -1.276111
H -6.746256 0.769429 -2.515739
H -5.027168 2.088408 -1.318298

5b-c11 , delta G = 1.5775 kcal/mol, population = 1.79 %
C 1.464077 -0.305360 -2.225883
C 2.908279 -0.681917 -1.872601
C 2.971861 -0.617980 -0.336653

C 1.579385 -0.077510 0.135097
C 0.579030 -1.278124 0.295823
C -0.854103 -0.723612 0.429041
C -1.845644 -1.868686 0.618259
C -1.689780 -2.977527 -0.384603
C -0.544050 -3.123270 -1.053211
C 0.619514 -2.178793 -0.964114
C 1.085240 0.691948 -1.110828
C -0.390114 1.079103 -1.029293
C 1.675074 0.769439 1.403643
C 0.924774 -2.119461 1.534093
C -2.829183 -3.944085 -0.502096
C -3.692523 -0.712397 1.593517
C -4.967194 0.024526 1.245863
C -4.584977 1.290039 0.504012
C -4.741724 1.381204 -0.879516
C -4.323552 2.520093 -1.562952
C -3.739888 3.577612 -0.869779
C -3.582258 3.493193 0.512193
C -4.002531 2.355770 1.195275
C 4.763740 0.087349 1.118965
C 5.745085 1.138642 1.422932
C 6.121630 2.191750 0.680298
C 5.687442 2.616012 -0.682351
O 0.607473 -1.445598 -2.210348
O 1.773053 1.929170 -1.328659
O 4.020806 0.331292 0.013037
O -1.250407 -0.012927 -0.738319
O -3.201164 -1.348659 0.512374
O 4.625957 -0.905537 1.813644
O -3.148440 -0.686111 2.677998
H 1.359618 0.135394 -3.222388
H 1.548630 -2.775581 -0.918405
H -0.923031 -0.062347 1.308485
H -0.427562 -3.962100 -1.737344
H 3.605644 0.042870 -2.302739
H 3.170129 -1.668596 -2.265114
H 0.596381 -1.618097 2.449969
H 0.440013 -3.099288 1.490183
H 2.002245 -2.287192 1.616041
H 2.684840 1.848771 -1.017513
H 3.221821 -1.574395 0.124583
H 2.129537 0.201322 2.219397
H 2.285550 1.659601 1.234856
H 0.692074 1.110518 1.736194
H -0.494887 1.868412 -0.264913
H -0.722276 1.488853 -1.989731
H -1.728323 -2.276449 1.631529
H -3.111022 -4.331520 0.485575
H -3.717795 -3.452329 -0.912641
H -2.563302 -4.784517 -1.147905
H 6.209050 0.995121 2.395019
H 6.863743 2.842066 1.144526
H 4.880920 3.358673 -0.598683
H 6.514950 3.112582 -1.197888
H 5.318858 1.791457 -1.291981
H -5.601498 -0.606772 0.617149
H -5.487279 0.254998 2.179063
H -5.185215 0.551830 -1.424750
H -4.451488 2.579255 -2.640300
H -3.410520 4.464651 -1.403618
H -3.130667 4.315442 1.060421
H -3.874192 2.290145 2.273423

5b-c20 , delta G = 1.8902 kcal/mol, population = 1.06 %

C 2.077773 -0.545349 -2.279127
C 3.276663 -1.331035 -1.732921
C 3.312838 -1.007034 -0.228953
C 2.196651 0.064268 0.012718
C 0.824926 -0.655973 0.271021
C -0.317970 0.378375 0.173479
C -1.657359 -0.297582 0.447037
C -1.877105 -1.565538 -0.331303
C -0.840344 -2.222992 -0.854049
C 0.578264 -1.735265 -0.814292
C 2.051891 0.710283 -1.383097
C 0.816170 1.599547 -1.504675
C 2.557013 1.040540 1.131646
C 0.806531 -1.316477 1.657732
C -3.283028 -2.082730 -0.383781
C -3.170888 1.435053 1.104333
C -4.372411 2.231717 0.640492
C -5.570996 1.315032 0.518586
C -6.278293 0.933315 1.661449
C -7.350606 0.050862 1.565151
C -7.727484 -0.459484 0.324545
C -7.027243 -0.080805 -0.818477

C -5.953555 0.800443 -0.721331
C 5.201897 -0.722916 1.249064
C 6.488862 -0.046629 1.464980
C 7.223691 0.677488 0.605918
C 6.981955 1.007555 -0.829171
O 0.865584 -1.287905 -2.159385
O 3.145936 1.565458 -1.734951
O 4.622309 -0.435863 0.058950
O -0.392892 0.957369 -1.124633
O -2.742199 0.616550 0.125511
O 4.700001 -1.463359 2.078265
O -2.683623 1.489710 2.214299
H 2.170112 -0.289919 -3.339535
H 1.234462 -2.594634 -0.585967
H -0.178330 1.171956 0.926230
H -1.008645 -3.167275 -1.369102
H 4.201372 -0.990012 -2.207530
H 3.176536 -2.401805 -1.931683
H 0.668852 -0.568185 2.444647
H -0.006495 -2.044338 1.733049
H 1.742225 -1.844479 1.860934
H 3.959373 1.224174 -1.339705
H 3.185734 -1.884980 0.405474
H 3.425519 1.645410 0.859623
H 1.738791 1.730880 1.348676
H 2.797765 0.504994 2.053716
H 0.987145 2.498454 -0.888138
H 0.686582 1.915903 -2.545480
H -1.726346 -0.516974 1.521205
H -3.925768 -1.412424 -0.965057
H -3.316031 -3.079073 -0.831222
H -3.715872 -2.130387 0.624199
H 6.857784 -0.184837 2.477665
H 8.141735 1.094002 1.021499
H 7.936053 1.138922 -1.347640
H 6.385025 0.256591 -1.346811
H 6.449541 1.966768 -0.903118
H -4.551721 3.018745 1.376831
H -4.144378 2.686834 -0.328426
H -5.986098 1.331078 2.630809
H -7.894784 -0.236268 2.460668
H -8.565766 -1.146297 0.248757
H -7.317662 -0.471553 -1.789892
H -5.405040 1.090629 -1.614135

Table S9. Experimental (Exptl.) of **5** and the calculated ^{13}C chemical shift values of possible isomers (**5a** and **5b**) with GIAO ^{13}C NMR calculations with STS protocol.

Carbon no.	Exptl.	5a	5b
1	82.4	81.53	81.66
2	37.1	35.33	35.62
3	74.3	73.47	73.25
4	49.7	51.02	51.51
5	38.5	38.10	40.77
6	74.1	73.06	72.07
7	72.3	72.94	73.36
8	133.6	133.59	135.24
9	123.5	125.75	123.56
10	69.3	69.38	69.45
11	74.5	74.53	74.70
12	66.5	66.25	66.41
13	6.4	7.65	6.98
14	15	15.95	15.70
15	20.2	20.37	18.84
16	170.6	170.97	171.13
17	41.7	42.57	42.87
18	133.6	133.43	133.43
19	129.2	126.51	129.00
20	128.7	127.41	126.85
21	123.5	125.85	125.38
22	128.7	127.61	126.53
23	129.2	128.53	127.17
24	165.7	166.23	166.28
25	120.1	118.85	118.66
26	146.6	148.59	148.97
27	15.5	15.03	15.09
	MAE	0.95	1.11
	RMS	1.20	1.34
	Pmean	42.60%	34.28%
	Prel	99.72%	0.28%

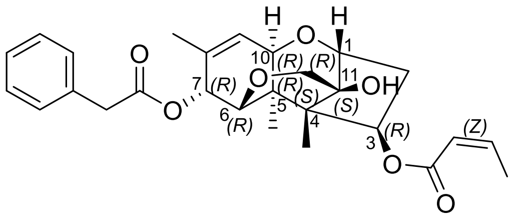

5a

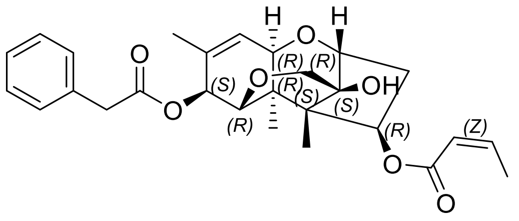

5b

Table S10. Geometry data of conformers of structure **6a** and **6b**

6a

6a-c1 , delta G = 0.0000 kcal/mol, population = 51.32 %

C 0.589060 -2.014211 -0.974092
C -0.610725 -1.413460 -1.721361
C -1.075366 -0.222648 -0.851667
C -0.186492 -0.258715 0.442785
C 1.141056 0.551432 0.160161
C 2.190592 0.276218 1.271080
C 3.535426 0.880501 0.943317
C 4.139673 0.403153 -0.359375
C 3.116988 0.636976 -1.488896
C 1.727941 0.088483 -1.196906
C 0.190660 -1.737659 0.457003
C 0.550892 -2.558938 1.604980
C -0.920754 0.204475 1.694372
C 0.863184 2.059790 0.121300
C 5.485210 1.042917 -0.668078
C -3.274388 0.648984 -0.428900
C -4.635109 0.239975 -0.049172
C -5.606759 1.143490 0.119173
C -7.005070 0.822412 0.506064
O 1.825768 -1.345689 -1.266384
O -0.760180 -2.670565 1.005997
O -2.469581 -0.439382 -0.518378
O -2.901380 1.789638 -0.641926
O 4.086612 1.682493 1.679751
H 0.755312 -3.074294 -1.183656
H 1.065915 0.442237 -2.003838
H 4.257414 -0.686415 -0.248406
H 1.855320 0.666617 2.235433
H 2.350512 -0.804507 1.364448
H 3.039700 1.713066 -1.685261
H 3.485541 0.173450 -2.411365
H -1.407508 -2.162268 -1.754696
H -0.369173 -1.125806 -2.748514
H 5.382466 2.128482 -0.770245
H 5.886468 0.645188 -1.605807
H 6.207754 0.848670 0.129355
H 0.484231 2.402366 1.087992
H 1.779388 2.623200 -0.081087
H 0.130568 2.325713 -0.646305
H 0.557282 -2.116949 2.601875
H 1.199296 -3.424070 1.461235
H -1.004480 0.736130 -1.367181
H -4.813157 -0.822885 0.094913
H -0.240805 0.284766 2.546920
H -1.389290 1.179713 1.542307
H -1.702952 -0.512826 1.953543
H -5.361398 2.194538 -0.036987
H -7.158892 -0.251413 0.639999
H -7.269191 1.340221 1.436820
H -7.703146 1.191368 -0.255956

6a-c2 , delta G = 0.0312 kcal/mol, population = 48.68 %

C 0.376249 -1.908317 -0.921627
C -0.750774 -1.215295 -1.701465
C -1.109786 0.036645 -0.868907
C -0.238693 -0.044980 0.434824
C 1.163721 0.624102 0.145010
C 2.170156 0.286408 1.278797
C 3.571473 0.743264 0.948612
C 4.137565 0.167307 -0.331019
C 3.155144 0.467206 -1.480483
C 1.716108 0.065899 -1.190030
C -0.006846 -1.552155 0.496064
C 0.260486 -2.372862 1.669501
C -0.935803 0.527136 1.661760
C 1.036314 2.150523 0.054264
C 5.544033 0.658809 -0.639293
C -3.229193 1.104208 -0.494201
C -4.630334 0.897866 -0.103970
C -5.155533 -0.280234 0.253857
C -6.572787 -0.477285 0.659182
O 1.675031 -1.372325 -1.217695
O -1.049858 -2.372846 1.058156
O -2.519316 -0.046235 -0.543173
O -2.762936 2.202408 -0.752927
O 4.193306 1.509290 1.666870
H 0.440148 -2.985289 -1.098748
H 1.098244 0.458514 -2.013755
H 4.142874 -0.924646 -0.186702
H 1.866120 0.741932 2.224800
H 2.222217 -0.800791 1.411891
H 3.187282 1.538801 -1.710415

H 3.484711 -0.059344 -2.383620
H -1.616015 -1.884230 -1.730413
H -0.469310 -0.978939 -2.731566
H 6.234678 0.415353 0.172793
H 5.552956 1.745892 -0.771794
H 5.912450 0.195805 -1.560316
H 0.699940 2.564047 1.008822
H 2.004183 2.609265 -0.170804
H 0.330932 2.461911 -0.721756
H 0.300369 -1.907629 2.654870
H 0.823360 -3.299946 1.554757
H -0.949068 0.969699 -1.410657
H -5.232146 1.803780 -0.110225
H -0.261958 0.560454 2.522063
H -1.295510 1.541849 1.474657
H -1.793730 -0.094440 1.929618
H -4.516616 -1.162538 0.255780
H -6.623234 -0.896057 1.672121
H -7.141920 0.455458 0.630083
H -7.055695 -1.211174 0.001574

6b

6b-c1 , delta G = 0.0000 kcal/mol, population = 41.92 %

C 0.702757 -1.981361 -0.849948
C -0.545573 -1.504733 -1.607438
C -1.051704 -0.274819 -0.820594
C -0.135158 -0.166193 0.451061
C 1.138328 0.688455 0.074311
C 2.225021 0.554080 1.171759
C 3.509740 1.248294 0.792034
C 4.123066 0.825905 -0.526879
C 3.060906 0.775314 -1.641460
C 1.720821 0.155715 -1.258934
C 0.324687 -1.616715 0.566919
C 0.762539 -2.325092 1.762218
C -0.868563 0.352048 1.681215
C 0.778523 2.173531 -0.066435
C 4.857366 -0.514247 -0.328829
C -3.284023 0.522525 -0.431761
C -4.621649 0.080268 -0.009887
C -5.637624 0.944979 0.083855
C -7.019093 0.588458 0.499093
O 1.894059 -1.273231 -1.225462
O -0.555691 -2.555252 1.214950
O -2.427579 -0.529527 -0.441669
O -2.968098 1.658882 -0.739395
O 4.048376 2.062654 1.525583
H 0.921325 -3.044317 -0.983352
H 1.017740 0.406704 -2.069721
H 4.870226 1.585573 -0.786254
H 1.880611 0.957133 2.127539
H 2.474607 -0.504634 1.312996
H 2.864736 1.795158 -1.989838
H 3.458235 0.223566 -2.501950
H -1.300908 -2.294679 -1.559875
H -0.344336 -1.286793 -2.660155
H 4.165796 -1.316236 -0.059806
H 5.351693 -0.795968 -1.264165
H 5.620392 -0.423442 0.451229
H 0.439565 2.575524 0.892381
H 1.650936 2.761633 -0.368985
H -0.010016 2.342378 -0.805697
H 0.770981 -1.807549 2.722004
H 1.455812 -3.161350 1.663793
H -1.038500 0.643138 -1.410128
H -4.746321 -0.974347 0.223336
H -0.182027 0.506558 2.518171
H -1.369346 1.301217 1.475334
H -1.624535 -0.370842 1.996442
H -5.445110 1.989849 -0.162136
H -7.312025 1.170838 1.381790
H -7.732241 0.854471 -0.291396
H -7.119233 -0.476191 0.725362

6b-c2 , delta G = 0.1742 kcal/mol, population = 31.23 %

C 0.484471 -1.881343 -0.810577
C -0.681168 -1.304262 -1.626790
C -1.090875 -0.009056 -0.889481
C -0.199193 0.055782 0.401959
C 1.167208 0.762182 0.036318
C 2.199734 0.560163 1.174349
C 3.562657 1.098966 0.812587
C 4.165226 0.562048 -0.468858
C 3.137078 0.586625 -1.615655
C 1.729552 0.122230 -1.256963
C 0.104842 -1.428936 0.580816

C 0.434692 -2.134848 1.811771
C -0.907327 0.693964 1.589493
C 0.967894 2.269248 -0.172491
C 4.739298 -0.842004 -0.198805
C -3.259156 0.973559 -0.559412
C -4.639408 0.725279 -0.121615
C -5.089471 -0.443867 0.350107
C -6.484761 -0.682971 0.805813
O 1.753931 -1.314261 -1.168042
O -0.885145 -2.248548 1.232156
O -2.489171 -0.137939 -0.532306
O -2.857987 2.070330 -0.915284
O 4.162631 1.881070 1.533439
H 0.594772 -2.965307 -0.901814
H 1.079166 0.414851 -2.097505
H 4.999835 1.224247 -0.728934
H 1.872398 1.036626 2.101864
H 2.329277 -0.512640 1.362071
H 3.060097 1.609580 -1.999662
H 3.499286 -0.031252 -2.446229
H -1.513871 -2.012919 -1.591458
H -0.420740 -1.136895 -2.675717
H 5.488602 -0.803299 0.598712
H 3.955842 -1.550231 0.081412
H 5.222090 -1.215483 -1.107659
H 0.233539 2.486520 -0.953787
H 0.632461 2.742403 0.754323
H 1.908735 2.751463 -0.456888
H 0.470340 -1.586467 2.753639
H 1.040651 -3.040282 1.761544
H -0.984656 0.886417 -1.503527
H -5.291928 1.592508 -0.192020
H -0.230170 0.814961 2.439350
H -1.303420 1.679205 1.331469
H -1.740929 0.064892 1.910720
H -4.401143 -1.285757 0.413908
H -7.108151 0.209491 0.707601
H -6.936399 -1.499799 0.228737
H -6.490899 -1.010385 1.853093

6b-c3 , delta G = 0.5890 kcal/mol, population = 15.49 %

C 0.843704 -1.725148 -0.672804
C -0.426189 -1.448045 -1.484079
C -1.047735 -0.192629 -0.836095
C -0.158085 0.138564 0.415832
C 1.033784 1.066083 -0.043407
C 2.113165 1.184173 1.079805
C 3.364495 0.365358 0.847095
C 4.097465 0.687782 -0.442226
C 3.088279 1.047153 -1.564037
C 1.712834 0.450449 -1.293149
C 0.436917 -1.236848 0.696179
C 0.962966 -1.752543 1.951516
C -0.957747 0.725237 1.572307
C 0.509285 2.470439 -0.375186
C 5.098598 -0.392647 -0.837559
C -3.350695 0.424541 -0.520708
C -4.629781 -0.085761 -0.004051
C -5.719540 0.688777 0.031594
C -7.046642 0.261989 0.546279
O 1.966641 -0.957192 -1.127298
O -0.337507 -2.172755 1.468872
O -2.395414 -0.533029 -0.423139
O -3.153994 1.538327 -0.975493
O 3.798102 -0.415065 1.679087
H 1.163415 -2.770560 -0.687267
H 1.065562 0.610766 -2.170457
H 4.658958 1.604094 -0.193696
H 2.456555 2.228643 1.101179
H 1.718288 0.953914 2.070043
H 2.993410 2.130681 -1.681535
H 3.458335 0.658461 -2.519029
H -1.113333 -2.291101 -1.364995
H -0.222100 -1.321583 -2.551133
H 4.579542 -1.327384 -1.069190
H 5.658803 -0.078399 -1.724134
H 5.809091 -0.584408 -0.029175
H 0.136349 2.959326 0.529292
H 1.313150 3.095430 -0.775025
H -0.300539 2.452030 -1.109862
H 0.933502 -1.133445 2.847459
H 1.734995 -2.520903 1.928686
H -1.114915 0.651609 -1.522783
H -4.646720 -1.111223 0.356836
H -1.583355 1.556468 1.237616
H -1.607867 -0.040190 2.002404
H -0.305583 1.098106 2.366837

H -5.634426 1.710291 -0.340771
H -7.039127 -0.773404 0.896421
H -7.361300 0.915297 1.369950
H -7.809012 0.369566 -0.235615

6b-c4 , delta G = 0.7725 kcal/mol, population = 11.36 %
C 0.651963 -1.659017 -0.645500
C -0.555094 -1.294919 -1.516499
C -1.084330 0.031261 -0.930960
C -0.212656 0.325832 0.342376
C 1.075823 1.123807 -0.100437
C 2.120929 1.184506 1.059688
C 3.297760 0.246043 0.903835
C 4.103756 0.453892 -0.365609
C 3.173065 0.865359 -1.536155
C 1.739737 0.404202 -1.300894
C 0.240652 -1.087527 0.690088
C 0.668766 -1.604772 1.981184
C -0.998341 1.027467 1.442788
C 0.695501 2.557702 -0.496852
C 5.014036 -0.726352 -0.687850
C -3.336715 0.844784 -0.722144
C -4.681776 0.544875 -0.213371
C -5.008130 -0.559478 0.469080
C -6.366040 -0.842273 1.005379
O 1.857333 -1.013160 -1.077569
O -0.646553 -1.920860 1.459553
O -2.468231 -0.178060 -0.554173
O -3.043232 1.907820 -1.246168
O 3.626268 -0.540773 1.776812
H 0.873618 -2.729174 -0.614218
H 1.142860 0.590311 -2.208291
H 4.737786 1.323375 -0.123442
H 2.557859 2.193621 1.059502
H 1.670151 1.028449 2.040723
H 3.180868 1.948678 -1.688069
H 3.540383 0.414536 -2.464428
H -1.320415 -2.068698 -1.403703
H -0.298025 -1.221517 -2.577089
H 4.419026 -1.616264 -0.912850
H 5.634132 -0.494035 -1.559642
H 5.672607 -0.955261 0.154129
H -0.093653 2.587245 -1.253354
H 0.345803 3.111549 0.378936
H 1.564519 3.090886 -0.893245
H 0.663078 -0.954873 2.855391
H 1.366510 -2.440809 2.013341
H -1.055461 0.853000 -1.647118
H -5.416322 1.322291 -0.410797
H -1.517890 1.906043 1.052208
H -1.743496 0.347638 1.862309
H -0.348087 1.358806 2.257111
H -4.239670 -1.308338 0.657564
H -6.747157 -1.785318 0.593457
H -6.322759 -0.980754 2.093033
H -7.073971 -0.041662 0.775850

Table S11. Experimental (Exptl.) of **6** and the calculated ^{13}C chemical shift values of possible isomers (**6a** and **6b**) with GIAO ^{13}C NMR calculations with STS protocol.

Carbon no.	Exptl.	6a	6b
1	79.7	79.68	79.21
2	37.2	35.35	34.94
3	73.9	73.32	73.20
4	49.3	50.91	50.82
5	48.2	48.46	47.75
6	45.1	44.75	42.03
7	212.1	211.36	213.01
8	39.4	39.02	41.65
9	36.6	35.78	32.15
10	71.5	70.96	71.02
11	65.6	66.29	65.89
12	47.9	47.99	47.81
13	5.7	6.60	6.63
14	18.8	18.66	19.49
15	13.7	14.27	19.36
16	166.2	166.48	166.00
17	122.5	121.68	121.18
18	145.5	147.26	146.74
19	18.1	18.17	18.14
	MAE	0.66	1.42
	RMS	0.85	2.06
	Pmean	54.03%	9.16%
	Prel	100.00%	0.00%

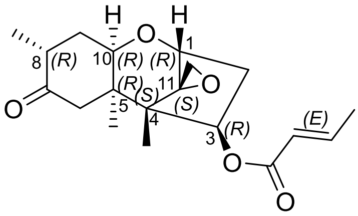

6a

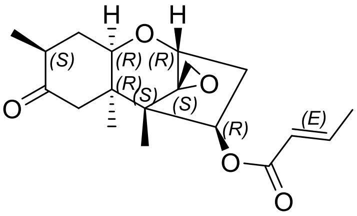

6b

Table S12. Geometry data of conformers of structure **7a** and **7b**

7a

7a-c1, delta G = 0.0000 kcal/mol, population = 72.94 %

C 0.633498 -2.052697 -0.982169
C -0.603436 -1.512569 -1.715495
C -1.120726 -0.349892 -0.837047
C -0.217177 -0.343395 0.448387
C 1.063571 0.534592 0.154503
C 2.136918 0.310181 1.254121
C 3.446663 0.981302 0.915092
C 4.060446 0.538311 -0.395475
C 3.015616 0.726500 -1.513196
C 1.659634 0.106448 -1.209911
C 0.235380 -1.801360 0.453791
C 0.647454 -2.607388 1.595213
C -0.960929 0.077645 1.709126
C 0.709994 2.027168 0.124547
C 5.370252 1.242768 -0.716003
C -3.358479 0.408501 -0.388282
C -4.686074 -0.093628 -0.003456
C -5.759708 0.660413 0.278338
C -5.895963 2.141974 0.280704
O 1.830821 -1.320495 -1.284440
O -0.661968 -2.782792 1.007905
O -2.497418 -0.639133 -0.491684
O -3.036058 1.564796 -0.600639
O 3.964943 1.807633 1.648647
H 0.851673 -3.102252 -1.197066
H 0.972232 0.427351 -2.009312
H 4.230996 -0.544762 -0.290406
H 1.791949 0.681163 2.222704
H 2.352392 -0.761168 1.343361
H 2.881808 1.798355 -1.702508
H 3.397826 0.287432 -2.442105
H -1.360009 -2.302157 -1.743293
H -0.387041 -1.209540 -2.743849
H 5.214509 2.322388 -0.814131
H 5.780163 0.866858 -1.658973
H 6.109677 1.081621 0.073301
H 1.597166 2.637026 -0.072400
H -0.033941 2.261379 -0.642616
H 0.312955 2.343643 1.092813
H 0.641212 -2.169338 2.593868
H 1.336935 -3.438557 1.442385
H -1.103461 0.612774 -1.350203
H -4.768265 -1.174837 0.062129
H -1.705874 -0.677453 1.970583
H -0.277972 0.185478 2.556224
H -1.476803 1.030083 1.566600
H -6.664481 0.114349 0.548605
H -6.624887 2.440137 -0.484913
H -6.318148 2.467166 1.239690
H -4.955196 2.656726 0.095783

7a-c2, delta G = 0.5869 kcal/mol, population = 27.06 %

C 0.314192 -1.870804 -0.946690
C -0.813546 -1.186416 -1.733132
C -1.185230 0.063964 -0.903973
C -0.320546 -0.009969 0.404606
C 1.078866 0.668194 0.120572
C 2.081593 0.338649 1.259714
C 3.482262 0.803109 0.936672
C 4.058220 0.231778 -0.340591
C 3.079310 0.522994 -1.494996
C 1.641708 0.112086 -1.211023
C -0.078888 -1.515458 0.468400
C 0.185416 -2.333456 1.644372
C -1.024870 0.558336 1.629235
C 0.941650 2.193622 0.027220
C 5.462388 0.734005 -0.642284
C -3.309868 1.115940 -0.519721
C -4.720844 0.923779 -0.150659
C -5.316572 -0.143196 0.405069
C -4.755350 -1.460174 0.816415
O 1.610720 -1.326462 -1.236590
O -1.121109 -2.341910 1.024456
O -2.598214 -0.031211 -0.587447
O -2.843203 2.216710 -0.768257
O 4.096232 1.571395 1.659191
H 0.385900 -2.947549 -1.122130
H 1.025214 0.499211 -2.038360
H 4.071530 -0.860029 -0.195115
H 1.770440 0.794012 2.203425
H 2.140108 -0.747973 1.395486
H 3.104846 1.594412 -1.726552

H 3.416744 -0.002605 -2.395772
H -1.674211 -1.861064 -1.765503
H -0.528497 -0.949281 -2.762008
H 5.838447 0.274322 -1.561879
H 6.151119 0.495396 0.172826
H 5.463460 1.821191 -0.774247
H 0.597754 2.606266 0.979472
H 1.907729 2.658296 -0.193284
H 0.238212 2.499279 -0.752791
H 0.216307 -1.867532 2.629702
H 0.754185 -3.257437 1.534100
H -1.029301 0.997745 -1.445468
H -5.316433 1.813550 -0.337357
H -1.879379 -0.068534 1.896233
H -0.353601 0.595296 2.491341
H -1.390313 1.570682 1.441513
H -6.384280 -0.027781 0.597691
H -5.106635 -2.236061 0.121717
H -3.667569 -1.486729 0.836265
H -5.152073 -1.733099 1.800971

7b

7b-c1 , delta G = 0.0000 kcal/mol, population = 67.73 %

C 0.751543 -1.998133 -0.885782
C -0.534495 -1.576174 -1.611520
C -1.093123 -0.391929 -0.790840
C -0.159014 -0.256411 0.465588
C 1.058079 0.674054 0.082819
C 2.172010 0.577827 1.157044
C 3.406475 1.353040 0.768756
C 4.019494 0.988208 -0.567535
C 2.941289 0.898655 -1.664388
C 1.644252 0.199283 -1.270835
C 0.381320 -1.681284 0.544739
C 0.881251 -2.385723 1.717729
C -0.896840 0.199257 1.717730
C 0.614450 2.138903 -0.022252
C 4.828139 -0.313623 -0.407372
C -3.360253 0.272729 -0.342847
C -4.648813 -0.272331 0.110575
C -5.777500 0.428610 0.299284
C -6.028371 1.882573 0.109064
O 1.895352 -1.218376 -1.267854
O -0.432978 -2.678529 1.191254
O -2.443167 -0.730590 -0.390647
O -3.107603 1.426561 -0.644506
O 3.908161 2.187406 1.506040
H 1.025662 -3.044667 -1.044120
H 0.914016 0.426590 -2.064344
H 4.719440 1.792003 -0.825529
H 1.824628 0.937981 2.128648
H 2.483765 -0.468035 1.266660
H 2.683499 1.912121 -1.990530
H 3.353284 0.385154 -2.541539
H -1.244916 -2.407057 -1.568831
H -0.364008 -1.326199 -2.662628
H 5.599913 -0.195722 0.360404
H 4.185475 -1.156202 -0.141134
H 5.319263 -0.551774 -1.356398
H 0.281604 2.506377 0.952477
H 1.444579 2.778348 -0.338970
H -0.203557 2.274634 -0.735827
H 0.880118 -1.885913 2.686906
H 1.617101 -3.180784 1.590801
H -1.141196 0.535985 -1.362704
H -4.652002 -1.340463 0.308350
H -1.450780 1.123728 1.537148
H -1.607628 -0.568843 2.030871
H -0.205380 0.375990 2.546022
H -6.639174 -0.145223 0.643057
H -6.404756 2.311634 1.046676
H -5.145203 2.429390 -0.214913
H -6.832366 2.018438 -0.626215

7b-c3 , delta G = 0.8955 kcal/mol, population = 14.91 %

C 0.430932 -1.838856 -0.854073
C -0.741226 -1.271684 -1.667948
C -1.170870 0.011039 -0.920626
C -0.284182 0.077186 0.374240
C 1.072854 0.806479 0.018237
C 2.105361 0.609582 1.156874
C 3.462088 1.169807 0.804117
C 4.074850 0.655622 -0.481657
C 3.049179 0.674445 -1.630572
C 1.647673 0.186584 -1.279327
C 0.040874 -1.404547 0.539994

C 0.375388 -2.117175 1.765609
C -1.001170 0.694139 1.567666
C 0.852014 2.312326 -0.177474
C 4.670354 -0.741851 -0.223311
C -3.349977 0.960424 -0.570590
C -4.739997 0.709845 -0.159000
C -5.256648 -0.345697 0.489997
C -4.605039 -1.587634 0.991767
O 1.692813 -1.250345 -1.203150
O -0.940907 -2.244127 1.179312
O -2.570673 -0.143705 -0.570687
O -2.956249 2.066119 -0.907528
O 4.050932 1.949786 1.536341
H 0.556905 -2.920279 -0.954649
H 0.995081 0.476942 -2.118930
H 4.899484 1.333302 -0.733456
H 1.769578 1.073791 2.087522
H 2.250516 -0.462662 1.336849
H 2.958170 1.699220 -2.006609
H 3.421965 0.068699 -2.465345
H -1.563752 -1.992439 -1.639859
H -0.481392 -1.091703 -2.714881
H 5.161662 -1.098780 -1.134220
H 5.416266 -0.699340 0.577166
H 3.896826 -1.464631 0.047365
H 0.114234 2.526150 -0.956461
H 0.510910 2.773069 0.753503
H 1.785832 2.810049 -0.458238
H 0.399344 -1.577989 2.713074
H 0.993688 -3.013791 1.709269
H -1.077214 0.913453 -1.526172
H -5.394711 1.542783 -0.401619
H -1.827402 0.052466 1.883451
H -0.325945 0.813135 2.419318
H -1.408425 1.677458 1.320700
H -6.326413 -0.283318 0.695343
H -4.963772 -1.802195 2.005048
H -4.920904 -2.438335 0.371651
H -3.517516 -1.546210 0.986594

7b-c2 , delta G = 1.0182 kcal/mol, population = 12.12 %
C 0.893288 -1.735624 -0.705275
C -0.409820 -1.517458 -1.481380
C -1.088110 -0.313666 -0.792706
C -0.191306 0.041336 0.447844
C 0.934835 1.046244 -0.015619
C 2.031935 1.199726 1.086133
C 3.321475 0.459400 0.804961
C 4.005142 0.858584 -0.490351
C 2.950823 1.181631 -1.581587
C 1.618870 0.499656 -1.294420
C 0.489101 -1.302505 0.683148
C 1.070271 -1.816053 1.914949
C -0.996518 0.554103 1.635046
C 0.326092 2.426479 -0.301001
C 5.062887 -0.145721 -0.935587
C -3.416193 0.165202 -0.409909
C -4.643570 -0.455050 0.111970
C -5.821024 0.161889 0.294768
C -6.194591 1.578062 0.033119
O 1.958068 -0.893619 -1.167124
O -0.213363 -2.299913 1.447378
O -2.404072 -0.740940 -0.361922
O -3.281222 1.300364 -0.833733
O 3.817775 -0.316149 1.605807
H 1.273110 -2.759843 -0.750316
H 0.943318 0.641522 -2.153452
H 4.512765 1.802281 -0.228800
H 2.315854 2.261305 1.127154
H 1.674275 0.923272 2.078678
H 2.791068 2.259937 -1.673764
H 3.320232 0.833870 -2.552467
H -1.042863 -2.401725 -1.362430
H -0.239892 -1.358639 -2.550101
H 5.805544 -0.309356 -0.150267
H 4.598693 -1.106450 -1.177053
H 5.578030 0.224748 -1.827694
H -0.493802 2.381891 -1.023473
H -0.057429 2.870012 0.622265
H 1.087392 3.105324 -0.696274
H 1.023456 -1.221448 2.826643
H 1.884851 -2.537247 1.857967
H -1.217287 0.540271 -1.458619
H -4.551429 -1.504318 0.377768
H -1.670122 1.360462 1.335092
H -1.597514 -0.256042 2.054538
H -0.348052 0.937003 2.427914

H -6.622488 -0.457645 0.699446
H -5.364047 2.177708 -0.333891
H -7.015116 1.608509 -0.695831
H -6.594871 2.023145 0.953108

7b-c4 , delta G = 1.5150 kcal/mol, population = 5.24 %
C 0.601178 -1.627338 -0.655682
C -0.614815 -1.288565 -1.524495
C -1.166075 0.030337 -0.942937
C -0.295546 0.346611 0.325914
C 0.977389 1.164991 -0.126425
C 2.025685 1.248555 1.029172
C 3.219953 0.332771 0.870189
C 4.016030 0.552063 -0.403470
C 3.071729 0.937110 -1.572195
C 1.648696 0.451210 -1.326271
C 0.183857 -1.056779 0.678306
C 0.622569 -1.560830 1.971084
C -1.085833 1.040324 1.428147
C 0.569730 2.589748 -0.528470
C 4.949929 -0.610281 -0.723369
C -3.428766 0.805947 -0.707359
C -4.785077 0.490792 -0.232743
C -5.194644 -0.499975 0.575420
C -4.428431 -1.592292 1.236970
O 1.793496 -0.962412 -1.094954
O -0.687994 -1.902297 1.452804
O -2.546768 -0.207215 -0.562893
O -3.145857 1.883089 -1.208691
O 3.566722 -0.445594 1.743692
H 0.841469 -2.693306 -0.620003
H 1.043908 0.620631 -2.231601
H 4.632173 1.436185 -0.168431
H 2.442573 2.266070 1.025910
H 1.582789 1.085044 2.012527
H 3.058420 2.019074 -1.733006
H 3.442367 0.485464 -2.498753
H -1.366098 -2.075307 -1.406947
H -0.361970 -1.214764 -2.586026
H 4.373067 -1.514320 -0.939337
H 5.560339 -0.370583 -1.599982
H 5.617449 -0.819803 0.116593
H 1.428235 3.137195 -0.928280
H -0.220410 2.600775 -1.284348
H 0.210704 3.141149 0.345035
H 0.606611 -0.906968 2.842196
H 1.334656 -2.384498 2.005869
H -1.155671 0.850007 -1.661555
H -5.517232 1.216568 -0.576918
H -0.435015 1.390653 2.233972
H -1.626703 1.905231 1.036714
H -1.812487 0.348724 1.861049
H -6.263190 -0.502894 0.796370
H -4.735366 -1.665795 2.286895
H -4.697425 -2.552948 0.775868
H -3.348627 -1.472497 1.176705

Table S13. Experimental (Exptl.) of **7** and the calculated ^{13}C chemical shift values of possible isomers (**7a** and **7b**) with GIAO ^{13}C NMR calculations with STS protocol.

Carbon no.	Exptl.	7a	7b
1	79.8	79.66	79.27
2	37.4	35.54	35.19
3	73.8	73.13	72.81
4	49.3	50.98	50.89
5	48.3	48.74	47.97
6	45.2	44.93	41.78
7	212.2	210.89	212.63
8	39.5	39.23	42.21
9	36.8	35.95	32.49
10	71.7	71.01	71.07
11	65.8	66.39	66.10
12	48.1	48.14	47.89
13	5.9	7.02	7.09
14	18.9	18.88	19.42
15	13.8	14.49	19.74
16	166.4	167.09	166.35
17	120.6	119.34	118.86
18	146.1	148.67	148.28
19	15.6	15.14	15.13
	MAE	0.82	1.57
	RMS	1.05	2.20
	Pmean	45.83%	7.00%
	Prel	100.00%	0.00%

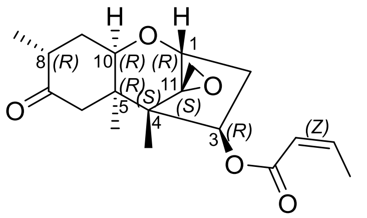

7a

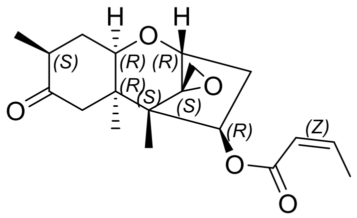

7b

Table S14. Geometry data of conformers of structure **8a**

8a

8a-c1 , delta G = 0.0000 kcal/mol, population = 64.43 %

C -4.023794 1.177545 0.078349
C -3.580190 0.615442 -1.223116
C -2.159669 0.288122 -1.424151
C -1.163549 0.653875 -0.336011
C -1.819265 0.558337 1.067675
C -3.197790 1.147593 1.129456
C 0.112676 -0.272406 -0.382653
C -0.371308 -1.675066 -0.033746
C -0.739782 -1.572631 1.429062
O -1.939055 -0.799405 1.559658
C 0.494215 -0.866262 2.005019
C -5.399795 1.767264 0.120005
C -0.801500 2.124816 -0.612128
O 0.467485 -2.793940 -0.385012
C -0.866721 -2.689099 -0.948497
C 1.008676 0.042231 0.869475
O 2.391322 -0.319140 0.617429
C 3.250485 0.663488 0.253060
C 4.592200 0.104387 0.032705
O 2.932870 1.835981 0.140030
C 0.869960 -0.183049 -1.701430
C 5.615696 0.881016 -0.340874
C 7.000313 0.399653 -0.581035
O -3.106989 -0.763033 -1.209671
H -0.950867 -2.540684 1.891965
H -1.176731 1.118733 1.767416
H -4.193507 0.853618 -2.092791
H -1.778023 0.336479 -2.445639
H -3.523437 1.541301 2.090371
H 1.255730 -1.617277 2.236052
H 0.270059 -0.314620 2.922468
H -6.148995 1.014193 -0.155278
H -5.638472 2.157474 1.112437
H -5.491765 2.582488 -0.609010
H -1.679988 2.757446 -0.453851
H -0.002492 2.481998 0.042840
H -0.474993 2.256057 -1.648017
H -0.891410 -2.483527 -2.018317
H -1.577404 -3.431619 -0.586120
H 0.986539 1.100915 1.129883
H 4.716289 -0.964969 0.185424
H 1.681300 -0.913793 -1.716783
H 0.217700 -0.395584 -2.552268
H 1.301344 0.810760 -1.844012
H 5.429187 1.946445 -0.480437
H 7.702755 0.928507 0.075626
H 7.097106 -0.676326 -0.415065
H 7.309628 0.633789 -1.607575

8a-c2 , delta G = 0.3517 kcal/mol, population = 35.57 %

C -4.082659 0.946575 0.125942
C -3.602334 0.460281 -1.192811
C -2.159074 0.280835 -1.415527
C -1.190413 0.717869 -0.329163
C -1.815213 0.527819 1.078996
C -3.244752 0.975083 1.167565
C 0.170352 -0.077862 -0.413966
C -0.170161 -1.527576 -0.090513
C -0.523876 -1.491728 1.379081
O -1.791713 -0.845090 1.542971
C 0.642669 -0.678790 1.954632
C -5.508668 1.399012 0.194593
C -0.976419 2.222603 -0.575273
O 0.764764 -2.554090 -0.479148
C -0.583418 -2.565074 -1.019302
C 1.051044 0.296297 0.831225
O 2.456099 0.069059 0.550479
C 3.216479 1.127516 0.195179
C 4.606128 0.763365 -0.109669
O 2.799968 2.275388 0.138645
C 0.894097 0.112329 -1.741182
C 5.082155 -0.488294 -0.116889
C 6.488373 -0.847908 -0.437778
O -2.994983 -0.864180 -1.217343
H -0.630517 -2.485495 1.823014
H -1.223261 1.135863 1.783644
H -4.247015 0.656617 -2.050190
H -1.796521 0.389474 -2.439287
H -3.595862 1.313843 2.140388
H 1.476625 -1.356531 2.161395
H 0.378225 -0.169779 2.885915
H -5.771891 1.743553 1.197671

H -5.686802 2.216558 -0.515585
H -6.185084 0.583104 -0.089877
H -0.224794 2.646309 0.095859
H -0.653548 2.403449 -1.604631
H -1.915844 2.760893 -0.417799
H -0.646392 -2.342021 -2.084000
H -1.214231 -3.378398 -0.661200
H 0.934474 1.342658 1.115074
H 5.246339 1.608530 -0.352299
H 1.230669 1.144424 -1.866505
H 1.769101 -0.539756 -1.786613
H 0.250123 -0.139378 -2.587628
H 4.409315 -1.310283 0.124623
H 6.517254 -1.535898 -1.292201
H 7.096729 0.031122 -0.665509
H 6.940366 -1.389098 0.403129

Table S15. Geometry data of conformers of structure **9a**

9a

9a-c3 , delta G = 0.0000 kcal/mol, population = 51.49 %

C 3.170026 -0.097472 -0.074089
C 2.508643 -0.367605 1.203623
C 1.236478 -0.010963 1.427148
C 0.439390 0.748830 0.389877
C 0.931316 0.353945 -1.023872
C 2.427314 0.280420 -1.127379
C -1.106203 0.457659 0.512944
C -1.262087 -1.032077 0.236577
C -0.948082 -1.152644 -1.237630
O 0.462292 -0.959455 -1.430238
C -1.791209 -0.016632 -1.826976
C 4.659122 -0.261469 -0.140149
C 0.745951 2.245597 0.618705
O -2.460903 -1.703546 0.671288
C -1.174605 -2.127965 1.191727
C -1.858812 1.067288 -0.728508
O -3.245278 1.326963 -0.471185
C -1.693094 0.925786 1.837055
H -1.178510 -2.139369 -1.649828
H 0.552725 1.101039 -1.739326
H 3.090644 -0.843405 1.990950
H 0.792714 -0.187367 2.402943
H 2.881930 0.462401 -2.098592
H -2.805423 -0.386390 -2.010382
H -1.388114 0.351383 -2.775130
H 5.042640 -0.045397 -1.140715
H 5.154068 0.404994 0.577244
H 4.944267 -1.285170 0.132651
H 0.502069 2.534652 1.644046
H 1.812947 2.427368 0.458599
H 0.185049 2.888859 -0.066991
H -1.024965 -1.912715 2.249186
H -0.842636 -3.108821 0.850366
H -1.378876 1.999757 -1.049370
H -3.304768 2.093197 0.113763
H -1.099215 0.590991 2.691584
H -1.745854 2.018582 1.879190
H -2.704321 0.531199 1.959289

9a-c1 , delta G = 0.0354 kcal/mol, population = 48.51 %

C 3.181201 -0.072726 -0.074445
C 2.517310 -0.350661 1.200431
C 1.242096 -0.002822 1.420366
C 0.444616 0.754740 0.380720
C 0.940602 0.354759 -1.028505
C 2.437599 0.296372 -1.130160
C -1.098727 0.459462 0.507172
C -1.255581 -1.030394 0.237769
C -0.924249 -1.166397 -1.231835
O 0.484684 -0.967535 -1.419812
C -1.770176 -0.039363 -1.836929
C 4.672100 -0.220762 -0.135110
C 0.741893 2.253644 0.602820
O -2.491264 -1.665970 0.637267
C -1.233091 -2.115922 1.206209
C -1.868458 1.049312 -0.733191
O -3.226091 1.389073 -0.436308
C -1.679761 0.940230 1.830649
H -1.148480 -2.158852 -1.633666
H 0.554710 1.091756 -1.750570
H 3.098919 -0.827212 1.987589
H 0.794111 -0.190354 2.392136
H 2.892459 0.483097 -2.100303
H -2.774647 -0.421668 -2.049428
H -1.351708 0.333454 -2.776832
H 4.967352 -1.241145 0.139355
H 5.056869 -0.001063 -1.134404
H 5.157104 0.451472 0.583683
H 1.808592 2.441549 0.448330
H 0.180892 2.888062 -0.091123
H 0.488834 2.547596 1.624420
H -1.112433 -1.889728 2.265263
H -0.919068 -3.110231 0.888316
H -1.420326 1.992799 -1.055824
H -3.684827 0.582478 -0.164362
H -1.790725 2.027630 1.830534
H -2.668101 0.504387 1.995380
H -1.048856 0.662545 2.679036

Table S16. Geometry data of conformers of structure **10a**

10a

10a-c1 , delta G = 0.0000 kcal/mol, population = 59.85 %

C -4.171330 1.031589 -0.029676
C -3.629494 0.524181 -1.291370
C -2.316694 0.318438 -1.462571
C -1.320457 0.663440 -0.378139
C -1.990759 0.497012 1.007928
C -3.383861 1.053912 1.058141
C -0.036738 -0.249472 -0.458458
C -0.526127 -1.671556 -0.207046
C -0.923757 -1.659304 1.251042
O -2.129195 -0.895523 1.399678
C 0.294619 -0.986515 1.895084
C -5.595752 1.499519 -0.018743
C -0.956306 2.152193 -0.571575
O 0.309708 -2.773085 -0.610063
C -1.013000 -2.627953 -1.190315
C 0.833141 -0.013106 0.826016
O 2.219759 -0.364668 0.576637
C 3.094401 0.635692 0.311198
C 4.435851 0.083260 0.072478
O 2.789865 1.816957 0.287441
C 0.747567 -0.068638 -1.751231
C 5.470276 0.877338 -0.226948
C 6.855204 0.404762 -0.482260
H -1.141145 -2.654343 1.649404
H -1.365896 1.012061 1.755097
H -4.323096 0.343959 -2.110700
H -1.946710 -0.017420 -2.426923
H -3.761535 1.397376 2.018451
H 1.052267 -1.749212 2.099663
H 0.049045 -0.489962 2.838216
H -5.896962 1.846184 0.973108
H -5.740185 2.317065 -0.736026
H -6.266606 0.688033 -0.327286
H -0.567668 2.324455 -1.578243
H -1.851885 2.767019 -0.441825
H -0.205913 2.485799 0.152319
H -1.013819 -2.365282 -2.247572
H -1.733719 -3.386251 -0.883661
H 0.809429 1.028337 1.148573

H 4.549709 -0.995571 0.145968
H 1.523861 -0.833151 -1.828994
H 0.105530 -0.156793 -2.631082
H 1.229560 0.911528 -1.783559
H 5.292818 1.951605 -0.289689
H 7.551394 0.879371 0.220847
H 6.943000 -0.681103 -0.393921
H 7.180607 0.710807 -1.484620

10a-c2 , delta G = 0.2363 kcal/mol, population = 40.15 %
C -4.213971 0.786282 -0.009343
C -3.629545 0.351972 -1.279181
C -2.303383 0.284653 -1.457613
C -1.343149 0.715605 -0.371334
C -1.986476 0.458598 1.012790
C -3.427841 0.873048 1.076098
C 0.027228 -0.058737 -0.470381
C -0.312427 -1.528078 -0.246646
C -0.707844 -1.583771 1.211618
O -1.983507 -0.948647 1.376134
C 0.437157 -0.802397 1.868359
C -5.677495 1.111539 0.011590
C -1.133232 2.235847 -0.544544
O 0.633901 -2.529308 -0.667581
C -0.696058 -2.510561 -1.249355
C 0.872472 0.239168 0.816822
O 2.284102 0.027151 0.553313
C 3.064237 1.105982 0.325978
C 4.455165 0.755850 0.010312
O 2.663917 2.259700 0.382119
C 0.786870 0.230628 -1.758326
C 4.913444 -0.494474 -0.130651
C 6.320410 -0.838396 -0.465009
H -0.822037 -2.603277 1.590604
H -1.412523 1.019070 1.768133
H -4.305142 0.111740 -2.098134
H -1.905189 0.000121 -2.427178
H -3.833237 1.164895 2.042152
H 1.268166 -1.488478 2.059116
H 0.143216 -0.350903 2.820253
H -6.008119 1.412284 1.009044
H -5.903539 1.921447 -0.693142
H -6.266495 0.242869 -0.308162
H -2.087867 2.754121 -0.414379
H -0.424088 2.636208 0.187306
H -0.758745 2.457394 -1.546915
H -0.721935 -2.228310 -2.301294
H -1.334568 -3.345509 -0.960293
H 0.748347 1.267364 1.158558
H 5.111893 1.612496 -0.123541
H 0.150213 0.121887 -2.639905
H 1.187047 1.247738 -1.757129
H 1.623187 -0.464299 -1.863368
H 4.223805 -1.327165 0.002601
H 6.945298 0.050762 -0.582010
H 6.748666 -1.477456 0.317618
H 6.356214 -1.426642 -1.390700

Table S17. Geometry data of conformers of structure **11a** and **11b**

11a

11a-c1 , delta G = 0.0000 kcal/mol, population = 23.74 %

C -1.462398 -1.595156 0.438663
C -2.329269 -1.498160 -0.820680
C -2.207982 -0.028467 -1.290710
C -1.304133 0.677840 -0.216057
C 0.220970 0.468530 -0.533725
C 1.077633 0.877304 0.686468
C 2.583326 0.640682 0.488340
C 2.910702 -0.628649 -0.256766
C 1.959295 -1.364786 -0.835864
C 0.498755 -1.022632 -0.829694
C -1.563994 -0.175881 1.034631
C -0.628949 0.156372 2.192526
C -1.656350 2.156799 -0.062772
C 0.630308 1.307525 -1.754598
C 4.360584 -1.012827 -0.288615
O -0.107204 -1.920448 0.128859
O -2.917561 0.058023 1.459533
O -3.486056 0.593474 -1.443526
O 0.749572 0.110851 1.849509
O 3.247943 1.780429 -0.085242
H -1.793918 -2.367491 1.143845
H 0.088358 -1.250028 -1.829621
H 0.943582 1.951228 0.896770
H 2.229117 -2.296062 -1.332720
H -3.374288 -1.715039 -0.581798
H -2.005019 -2.220544 -1.576540
H 1.560205 0.935119 -2.195571
H -0.130759 1.264263 -2.538296
H 0.767553 2.358538 -1.482563
H -3.147453 -0.611004 2.119110
H -1.753476 0.037197 -2.284033
H -3.921381 0.543127 -0.580595
H -0.964240 2.675192 0.605814
H -1.631123 2.657622 -1.034476
H -2.661958 2.276757 0.343780
H -0.902472 1.158763 2.565464
H -0.766102 -0.563347 3.009648
H 3.007772 0.560670 1.498991
H 4.510764 -1.954796 -0.822483
H 4.961378 -0.235063 -0.774069
H 4.749539 -1.118892 0.732122
H 3.091088 1.775243 -1.037495

11a-c2 , delta G = 0.0236 kcal/mol, population = 22.81 %

C -1.463286 -1.599015 0.435120
C -2.321296 -1.505453 -0.830825
C -2.199722 -0.036094 -1.301759
C -1.303934 0.673008 -0.222552
C 0.224711 0.466412 -0.530103
C 1.072083 0.874052 0.695893
C 2.579067 0.635518 0.506262
C 2.913218 -0.619531 -0.255489
C 1.964468 -1.365921 -0.823015
C 0.504853 -1.025055 -0.821497
C -1.571550 -0.178907 1.027511
C -0.646131 0.157958 2.191790
C -1.662285 2.151018 -0.074300
C 0.645181 1.307192 -1.743985
C 4.366835 -0.984470 -0.304523
O -0.105814 -1.922469 0.136117
O -2.928584 0.052751 1.443944
O -3.478906 0.582959 -1.461689
O 0.735627 0.114594 1.860892
O 3.229131 1.736572 -0.150031
H -1.799512 -2.370362 1.139354
H 0.098817 -1.254290 -1.822601
H 0.931245 1.949608 0.903087
H 2.237883 -2.298430 -1.315340
H -3.367610 -1.724219 -0.599313
H -1.990031 -2.228208 -1.583284
H 1.630532 1.009166 -2.106958
H -0.068437 1.195524 -2.565372
H 0.697189 2.367471 -1.482248
H -3.164101 -0.621469 2.096186
H -1.739626 0.031028 -2.292166
H -3.917480 0.533379 -0.600378
H -0.967415 2.676615 0.585931
H -1.646401 2.645994 -1.048975
H -2.665419 2.268497 0.339358
H -0.924848 1.160922 2.559735
H -0.788554 -0.559512 3.010015
H 2.996437 0.521455 1.521628

H 4.941006 -0.214427 -0.832183
H 4.782042 -1.049686 0.709269
H 4.518981 -1.941690 -0.810137
H 3.227092 2.483990 0.461075

11a-c6 , delta G = 0.2512 kcal/mol, population = 15.53 %
C -1.466622 -1.605479 0.405537
C -2.303109 -1.505822 -0.876332
C -2.183041 -0.038116 -1.328433
C -1.297171 0.669111 -0.243586
C 0.232937 0.459962 -0.535954
C 1.062136 0.857545 0.705507
C 2.571350 0.619596 0.533994
C 2.915807 -0.628003 -0.235310
C 1.974318 -1.370245 -0.820050
C 0.514274 -1.030604 -0.830934
C -1.592392 -0.187669 1.004466
C -0.677397 0.141717 2.177239
C -1.651138 2.149152 -0.097109
C 0.669964 1.306034 -1.740837
C 4.370240 -0.991249 -0.270943
O -0.105948 -1.930269 0.114671
O -2.921096 -0.009354 1.509422
O -3.525124 0.492883 -1.351711
O 0.706073 0.089283 1.857261
O 3.229663 1.727645 -0.102890
H -1.815898 -2.375561 1.100957
H 0.119592 -1.252770 -1.838674
H 0.920643 1.931933 0.919748
H 2.253868 -2.298423 -1.316994
H -3.353523 -1.729295 -0.667084
H -1.957415 -2.218186 -1.630848
H 1.660807 1.010935 -2.091101
H -0.030899 1.194613 -2.573542
H 0.717445 2.365690 -1.475385
H -3.507917 0.181822 0.761132
H -1.745639 0.054471 -2.330370
H -3.490269 1.399881 -1.682122
H -1.619268 2.655569 -1.068073
H -2.653623 2.273652 0.319879
H -0.957994 2.672066 0.565861
H -0.951681 1.145857 2.544655
H -0.835511 -0.575605 2.990657

H 2.974703 0.495621 1.553731
H 4.949748 -0.216380 -0.785552
H 4.774100 -1.064907 0.746830
H 4.528829 -1.944035 -0.782915
H 3.230622 2.464729 0.520614

11a-c3 , delta G = 0.3338 kcal/mol, population = 13.51 %
C -1.465183 -1.603609 0.407220
C -2.310715 -1.500379 -0.868198
C -2.191971 -0.031917 -1.318388
C -1.298395 0.672335 -0.237771
C 0.228574 0.461624 -0.540400
C 1.066723 0.860779 0.695627
C 2.575026 0.625772 0.516536
C 2.913403 -0.636964 -0.234498
C 1.970183 -1.368536 -0.832376
C 0.508924 -1.028347 -0.840158
C -1.585253 -0.187049 1.010628
C -0.660875 0.137964 2.177295
C -1.646973 2.153127 -0.085629
C 0.653945 1.306475 -1.752232
C 4.363832 -1.020280 -0.250052
O -0.106577 -1.929257 0.105743
O -2.910086 -0.008461 1.524709
O -3.532793 0.500937 -1.333524
O 0.719474 0.085057 1.845251
O 3.246162 1.770843 -0.039566
H -1.809336 -2.375271 1.103310
H 0.110802 -1.248831 -1.847076
H 0.931083 1.933360 0.913270
H 2.246957 -2.295547 -1.333277
H -3.359871 -1.722517 -0.651503
H -1.971639 -2.211685 -1.626690
H 1.591027 0.938063 -2.181018
H -0.094723 1.262665 -2.548073
H 0.785671 2.356895 -1.475013
H -3.501769 0.186870 0.781384
H -1.760424 0.060224 -2.323171
H -3.498726 1.408675 -1.662061
H -2.651598 2.279869 0.325377
H -0.956416 2.669087 0.585252
H -1.607388 2.665054 -1.053559
H -0.931180 1.140968 2.550260

H -0.813107 -0.582281 2.989213
H 2.984943 0.538129 1.532521
H 4.971379 -0.237772 -0.719237
H 4.738353 -1.136582 0.774947
H 4.521679 -1.956931 -0.791012
H 3.114194 1.764446 -0.995534

11a-c8 , delta G = 0.4894 kcal/mol, population = 10.38 %
C -1.474790 -1.591214 0.444346
C -2.335250 -1.494672 -0.818777
C -2.194751 -0.031605 -1.302825
C -1.287433 0.675661 -0.232119
C 0.236175 0.453710 -0.546364
C 1.089717 0.868101 0.672711
C 2.589164 0.619294 0.480689
C 2.908336 -0.657936 -0.259091
C 1.954181 -1.399326 -0.824158
C 0.497903 -1.043460 -0.823917
C -1.560618 -0.169874 1.028048
C -0.621433 0.148613 2.185555
C -1.629807 2.157325 -0.091727
C 0.661549 1.277475 -1.770429
C 4.357717 -1.043188 -0.299631
O -0.120859 -1.925278 0.142920
O -2.917239 -0.037100 1.486972
O -3.467008 0.601889 -1.466745
O 0.756459 0.113947 1.843356
O 3.158992 1.771632 -0.174553
H -1.817711 -2.349461 1.156415
H 0.087553 -1.277424 -1.822146
H 0.961201 1.944359 0.872630
H 2.218083 -2.337803 -1.310756
H -3.382638 -1.697676 -0.577200
H -2.019481 -2.228156 -1.567500
H -0.058030 1.169088 -2.586854
H 0.731351 2.339235 -1.518998
H 1.640553 0.961844 -2.136264
H -3.013695 0.830427 1.902696
H -1.736250 0.021186 -2.294796
H -3.923683 0.526154 -0.616970
H -1.584840 2.654175 -1.064539
H -2.647244 2.295510 0.283691
H -0.948153 2.676667 0.587110

H -0.890227 1.148483 2.577746
H -0.759336 -0.581366 2.991356
H 3.013828 0.538074 1.494589
H 4.949370 -0.298671 -0.847925
H 4.772504 -1.097369 0.715262
H 4.499062 -2.010618 -0.788450
H 4.108620 1.768504 -0.006509

11a-c10 , delta G = 0.8688 kcal/mol, population = 5.47 %
C -1.463828 -1.601374 0.412369
C -2.297008 -1.512240 -0.873881
C -2.178363 -0.039133 -1.331904
C -1.300178 0.671589 -0.253797
C 0.231433 0.459784 -0.542009
C 1.057750 0.865650 0.699005
C 2.567524 0.627242 0.532041
C 2.915322 -0.623022 -0.231336
C 1.975921 -1.369287 -0.814184
C 0.515522 -1.031554 -0.827372
C -1.595521 -0.179167 0.999423
C -0.684600 0.161074 2.171922
C -1.649921 2.154371 -0.116220
C 0.670708 1.299993 -1.750036
C 4.370495 -0.983510 -0.263935
O -0.103466 -1.926270 0.123991
O -2.927265 -0.004981 1.497671
O -3.469719 0.609979 -1.331237
O 0.699770 0.105784 1.855901
O 3.227664 1.732558 -0.107407
H -1.812578 -2.366870 1.113263
H 0.120954 -1.260526 -1.833856
H 0.915793 1.941225 0.905541
H 2.257617 -2.299026 -1.306994
H -3.345315 -1.746425 -0.661687
H -1.941521 -2.226829 -1.622724
H -0.029290 1.184773 -2.582898
H 0.716316 2.360782 -1.489303
H 1.662389 1.003686 -2.096905
H -3.504743 0.210308 0.748154
H -1.739315 0.045543 -2.334395
H -4.045609 0.103493 -1.917971
H -0.945566 2.676476 0.535701
H -1.635506 2.645360 -1.093043

H -2.648735 2.285359 0.303682
H -0.960312 1.168068 2.530006
H -0.844921 -0.549641 2.990718
H 2.967988 0.507455 1.553486
H 4.774023 -1.049981 0.754455
H 4.531429 -1.939088 -0.769974
H 4.948560 -0.210490 -0.782922
H 3.215693 2.476021 0.508408

11a-c9 , delta G = 0.9415 kcal/mol, population = 4.84 %
C -1.462560 -1.599876 0.415125
C -2.305033 -1.507618 -0.864464
C -2.187496 -0.034005 -1.321226
C -1.301773 0.674277 -0.247414
C 0.226671 0.461149 -0.545874
C 1.062104 0.868743 0.689580
C 2.570948 0.633515 0.514318
C 2.912633 -0.631471 -0.231495
C 1.971396 -1.367648 -0.826758
C 0.509814 -1.029490 -0.836190
C -1.588436 -0.178815 1.006403
C -0.667937 0.157074 2.172737
C -1.646481 2.157767 -0.104925
C 0.653990 1.300375 -1.760759
C 4.363931 -1.011293 -0.245157
O -0.104150 -1.925488 0.115803
O -2.916099 -0.003695 1.514233
O -3.477086 0.617220 -1.313286
O 0.713353 0.101300 1.844564
O 3.243453 1.776891 -0.043451
H -1.805684 -2.366873 1.117030
H 0.111312 -1.256825 -1.841652
H 0.925963 1.942482 0.899671
H 2.250287 -2.296155 -1.323710
H -3.352072 -1.740457 -0.644776
H -1.956309 -2.221579 -1.617085
H 1.592507 0.931249 -2.185915
H -0.093558 1.252315 -2.557367
H 0.782552 2.352294 -1.488045
H -3.499182 0.213465 0.769693
H -1.753966 0.049799 -2.326468
H -4.057701 0.111484 -1.896025
H -1.624872 2.653609 -1.079398

H -2.647294 2.290802 0.309221
H -0.943930 2.673900 0.553454
H -0.939694 1.163033 2.536256
H -0.822260 -0.556487 2.990165
H 2.978532 0.549485 1.531511
H 4.523942 -1.951122 -0.779972
H 4.969065 -0.230304 -0.719927
H 4.739460 -1.119654 0.780338
H 3.103180 1.774596 -0.998283

11a-c12 , delta G = 1.0988 kcal/mol, population = 3.71 %
C -1.466997 -1.591768 0.424932
C -2.308522 -1.501956 -0.855840
C -2.180283 -0.032462 -1.322525
C -1.288218 0.676342 -0.254510
C 0.238978 0.449562 -0.552240
C 1.076326 0.860098 0.680522
C 2.578393 0.613751 0.506005
C 2.911650 -0.655823 -0.240386
C 1.966800 -1.395472 -0.822737
C 0.508930 -1.045797 -0.830684
C -1.581287 -0.165399 1.005310
C -0.658100 0.173334 2.168716
C -1.624368 2.162796 -0.122670
C 0.677293 1.278080 -1.768419
C 4.362952 -1.034717 -0.268533
O -0.111729 -1.930909 0.128818
O -2.907654 0.022587 1.513007
O -3.465810 0.627972 -1.316401
O 0.723299 0.104427 1.842630
O 3.156033 1.771842 -0.132868
H -1.818007 -2.350112 1.132443
H 0.106570 -1.276867 -1.833570
H 0.946539 1.935884 0.882244
H 2.238995 -2.328427 -1.315318
H -3.357309 -1.725689 -0.635025
H -1.964535 -2.223556 -1.603352
H 0.731367 2.340363 -1.515703
H 1.665489 0.972288 -2.117413
H -0.026833 1.161675 -2.597453
H -3.488724 0.242092 0.767575
H -1.747799 0.042837 -2.328599
H -4.049664 0.124356 -1.897674

H -1.612368 2.648288 -1.102284
H -2.619357 2.304610 0.302799
H -0.911662 2.682944 0.521555
H -0.922390 1.184556 2.523246
H -0.817701 -0.531537 2.992654
H 2.988663 0.525191 1.525097
H 4.766639 -1.096734 0.750384
H 4.513771 -1.996989 -0.764684
H 4.957314 -0.282447 -0.803066
H 4.101047 1.775709 0.059159

11b

11b-c1 , delta G = 0.0000 kcal/mol, population = 55.05 %

C -1.362637 -0.371709 1.631275
C -2.355845 -1.357864 1.009895
C -2.336508 -1.058417 -0.507962
C -1.358694 0.159425 -0.680226
C 0.133630 -0.324544 -0.781740
C 1.079312 0.882551 -0.626349
C 2.550181 0.475003 -0.719907
C 2.877023 -0.751199 0.105428
C 1.916393 -1.565661 0.548333
C 0.448077 -1.323518 0.359278
C -1.450367 0.848463 0.692282
C -0.403532 1.942267 0.896645
C -1.760342 1.039760 -1.863205
C 0.400366 -1.001452 -2.133568
C 4.333626 -1.031300 0.322496
O -0.035852 -0.894391 1.654958
O -2.755420 1.438393 0.808031
O -3.646749 -0.804196 -1.021054
O 0.928863 1.507217 0.651086
O 3.392313 1.585966 -0.397705
H -1.593360 -0.112035 2.672222
H -0.037786 -2.283341 0.108032
H 0.884298 1.625789 -1.417451
H 2.184640 -2.458155 1.112703
H -3.362971 -1.181687 1.398538
H -2.083168 -2.390865 1.248105
H -0.389599 -1.714759 -2.383754
H 0.449067 -0.260409 -2.937445
H 1.346643 -1.549825 -2.117359
H -2.895920 1.682478 1.733285
H -1.982645 -1.920364 -1.081560
H -3.994167 -0.048441 -0.526507
H -1.864065 0.436649 -2.769418
H -2.720716 1.523667 -1.677862
H -1.027116 1.826105 -2.058378
H -0.661718 2.785429 0.232915
H -0.425752 2.299475 1.933734
H 2.776955 0.245725 -1.771299
H 4.479305 -1.991469 0.824187
H 4.870344 -1.045254 -0.634974
H 4.796998 -0.242787 0.925152
H 3.066939 1.936926 0.443879

11b-c2 , delta G = 0.3075 kcal/mol, population = 32.74 %

C -1.365608 -0.401290 1.622054
C -2.337643 -1.402076 0.986044
C -2.324933 -1.082078 -0.520188
C -1.354199 0.138051 -0.688630
C 0.141711 -0.333952 -0.780609
C 1.070602 0.884160 -0.608175
C 2.547424 0.495101 -0.692397
C 2.882730 -0.735959 0.122248
C 1.928532 -1.565269 0.550925
C 0.458667 -1.334863 0.357986
C -1.472499 0.825249 0.689735
C -0.436707 1.925172 0.904540
C -1.751032 1.015762 -1.876434
C 0.426473 -1.002025 -2.133446
C 4.341092 -1.002296 0.344681
O -0.033998 -0.915214 1.650625
O -2.738552 1.461470 0.894472
O -3.681605 -0.740215 -0.874963
O 0.899154 1.496350 0.671154
O 3.372202 1.612829 -0.350665
H -1.610647 -0.148085 2.658691
H -0.017509 -2.297787 0.098426
H 0.874779 1.631405 -1.395693
H 2.202854 -2.460425 1.108059
H -3.350263 -1.253516 1.372802
H -2.045166 -2.432221 1.208408
H 0.469377 -0.258390 -2.935323
H 1.380610 -1.536398 -2.113153
H -0.350929 -1.727364 -2.388984

H -3.413974 0.956481 0.415310
H -2.002897 -1.939210 -1.125223
H -3.723295 -0.595396 -1.829057
H -1.845958 0.417383 -2.789528
H -2.706956 1.513147 -1.695270
H -1.015276 1.798180 -2.073999
H -0.692283 2.767445 0.239014
H -0.474788 2.279947 1.940436
H 2.786898 0.280229 -1.744150
H 4.883340 -0.999827 -0.609803
H 4.792183 -0.215401 0.958629
H 4.494609 -1.966273 0.836674
H 3.033100 1.952282 0.490311

11b-c3 , delta G = 0.8915 kcal/mol, population = 12.21 %

C -1.362846 -0.395656 1.621158
C -2.330844 -1.405740 0.990555
C -2.321887 -1.085041 -0.522566
C -1.357788 0.130339 -0.695432
C 0.139862 -0.340202 -0.782943
C 1.065595 0.881539 -0.617522
C 2.543419 0.495740 -0.699631
C 2.882600 -0.730313 0.120818
C 1.930848 -1.560275 0.553551
C 0.460484 -1.333425 0.360543
C -1.476096 0.824078 0.680725
C -0.444382 1.929070 0.886885
C -1.750942 1.001974 -1.890482
C 0.426549 -1.014479 -2.132180
C 4.341746 -0.991291 0.344242
O -0.031146 -0.907234 1.651678
O -2.745381 1.453473 0.885776
O -3.629893 -0.663304 -0.969746
O 0.892855 1.501670 0.658064
O 3.365086 1.617387 -0.363185
H -1.607711 -0.136266 2.656417
H -0.014729 -2.298617 0.106825
H 0.868095 1.623373 -1.409223
H 2.207700 -2.452208 1.114595
H -3.340588 -1.262889 1.388980
H -2.025662 -2.432600 1.214395
H 0.466426 -0.274760 -2.937653
H 1.382143 -1.546139 -2.109180
H -0.349645 -1.742476 -2.383670
H -3.412976 0.957085 0.386202
H -2.001122 -1.945882 -1.123992
H -4.246728 -1.377616 -0.765494
H -1.003339 1.771020 -2.096424
H -1.866906 0.391248 -2.790207
H -2.701358 1.507429 -1.711860
H -0.702181 2.764613 0.213976
H -0.484294 2.292162 1.919823
H 2.783429 0.276275 -1.750289
H 4.884195 -0.989955 -0.610104
H 4.790094 -0.200932 0.955765
H 4.498391 -1.953148 0.839402
H 3.022687 1.961872 0.474423

Table S18. Experimental (Exptl.) of **11** and the calculated ^{13}C chemical shift values of possible isomers (**11a** and **11b**) with GIAO ^{13}C NMR calculations with STS protocol.

Carbon no.	Exptl.	11a	11b
1	82.6	81.64	82.09
2	40.1	38.82	39.04
3	74	74.02	74.08
4	49.6	50.04	50.60
5	38.3	37.67	40.06
6	77	76.16	74.21
7	72.4	72.08	69.30
8	136.5	136.80	138.94
9	121.5	123.31	121.60
10	69.3	68.75	69.10
11	75.1	75.10	75.42
12	66.5	65.87	66.52
13	6.8	8.44	8.89
14	15.5	16.25	15.44
15	20.5	20.76	20.42
	MAE	0.70	1.04
	RMS	0.87	1.49
	Pmean	50.97%	26.83%
	Prel	99.99%	0.01%

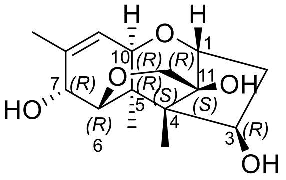

11a

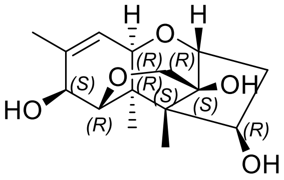

11b

Table S19. Geometry data of conformers of structure **12a** and **12b**

12a

12a-c1 , delta G = 0.0000 kcal/mol, population = 55.65 %

C -1.280863 -1.590261 0.552683
C -2.125979 -1.611089 -0.729151
C -2.114267 -0.148714 -1.263196
C -1.271454 0.671188 -0.212896
C 0.269189 0.542354 -0.537926
C 1.116617 1.057290 0.659565
C 2.588835 0.787542 0.469932
C 2.944561 -0.669007 0.271442
C 2.102302 -1.229039 -0.892874
C 0.611635 -0.947721 -0.773712
C -1.532806 -0.176376 1.026755
C -1.582160 0.257850 2.415611
C 0.640905 1.361730 -1.779947
C 4.432955 -0.904654 0.060706
O 0.121197 -1.772528 0.299423
O -2.808860 -0.000468 1.688317
O -3.436140 0.367912 -1.435727
C -1.730944 2.118895 -0.096064
O 3.422698 1.681181 0.486423
H -1.561778 -2.350664 1.286744
H 0.142837 -1.267772 -1.717663
H 2.608032 -1.178848 1.188004
H 0.970746 2.129498 0.814493
H 0.818684 0.526753 1.572173
H 2.471775 -0.811429 -1.836928
H 2.246932 -2.314088 -0.950937
H -3.153711 -1.883941 -0.467203
H -1.764541 -2.343142 -1.457593
H 4.631373 -1.974832 -0.055843
H 5.017120 -0.533714 0.907599
H 4.781208 -0.389819 -0.841183
H 1.708778 1.272265 -2.002982
H 0.090014 1.036233 -2.667451
H 0.434096 2.422737 -1.615716
H -1.411354 1.307947 2.654342
H -1.361975 -0.457542 3.208342
H -1.667798 -0.086242 -2.259494
H -3.888403 0.324656 -0.581780
H -1.045003 2.709609 0.517137
H -1.799356 2.586246 -1.081689
H -2.719923 2.168498 0.365650

12a-c3 , delta G = 0.1344 kcal/mol, population = 44.35 %

C -1.304356 -1.586208 0.540429
C -2.145634 -1.589305 -0.742993
C -2.099529 -0.137603 -1.277721
C -1.277848 0.677240 -0.206483
C 0.265700 0.548380 -0.525136
C 1.113070 1.054591 0.675642
C 2.584208 0.781077 0.486454
C 2.933540 -0.676945 0.287107
C 2.092846 -1.230440 -0.881578
C 0.602555 -0.942804 -0.770201
C -1.540660 -0.175491 1.030367
C -1.527688 0.238241 2.428007
C 0.646453 1.375701 -1.759879
C 4.421515 -0.919464 0.081248
O 0.098876 -1.775807 0.290659
O -2.781516 0.014859 1.741219
O -3.453298 0.312831 -1.405774
C -1.742432 2.121630 -0.082398
O 3.421797 1.671257 0.504527
H -1.592034 -2.353079 1.265013
H 0.139695 -1.252799 -1.720351
H 2.591223 -1.186744 1.201545
H 0.968327 2.126317 0.835839
H 0.814659 0.519412 1.584779
H 2.468881 -0.812612 -1.823008
H 2.232780 -2.316010 -0.941606
H -3.181208 -1.826053 -0.478948
H -1.802485 -2.331875 -1.469269
H 5.004465 -0.552728 0.930817
H 4.775416 -0.404905 -0.818604
H 4.615204 -1.990380 -0.036490
H 0.090268 1.069364 -2.650730
H 0.455443 2.437692 -1.582606
H 1.712711 1.274135 -1.985580
H -1.329105 1.281111 2.677138
H -1.287829 -0.493385 3.200475
H -1.614605 -0.083415 -2.260481
H -3.444863 1.168853 -1.852763
H -2.750324 2.154737 0.337707

H -1.084819 2.702897 0.569656
H -1.763599 2.615479 -1.059168

H -3.842873 0.519955 -0.226552
H -1.784399 2.813667 0.095380
H -2.601293 1.831302 1.312546
H -0.916863 2.303755 1.554144

12b

12b-c1 , delta G = 0.0000 kcal/mol, population = 45.47 %

C -1.190563 -1.654826 -0.129087
C -2.115914 -1.170647 -1.254870
C -2.122929 0.382378 -1.153145
C -1.208344 0.722541 0.085041
C 0.306187 0.762568 -0.358018
C 1.229794 0.764667 0.889519
C 2.688104 0.674151 0.520239
C 3.078714 -0.501639 -0.348843
C 2.100941 -0.666133 -1.528348
C 0.622607 -0.499906 -1.194270
C -1.397290 -0.557346 0.890685
C -1.347866 -0.720357 2.336655
C 0.604988 2.022403 -1.181222
C 3.181983 -1.760168 0.535193
O 0.191405 -1.696122 -0.518604
O -2.623632 -0.685001 1.649730
O -3.447538 0.903868 -1.021513
C -1.648006 1.992181 0.803312
O 3.514276 1.475225 0.935589
H -1.432524 -2.651348 0.250935
H 0.088433 -0.422631 -2.154761
H 4.077586 -0.286746 -0.747491
H 1.076893 1.658523 1.499096
H 1.007485 -0.115588 1.505321
H 2.350319 0.070869 -2.299218
H 2.240959 -1.653173 -1.985479
H -3.127505 -1.542045 -1.059838
H -1.810717 -1.541439 -2.238045
H 2.219203 -2.017250 0.983318
H 3.506300 -2.605889 -0.079742
H 3.917371 -1.610954 1.332751
H -0.015775 2.085318 -2.079961
H 0.435146 2.920219 -0.580446
H 1.652044 2.042128 -1.500377
H -1.150202 0.147317 2.966766
H -1.083131 -1.690498 2.758004
H -1.737390 0.848607 -2.064077

12b-c4 , delta G = 0.0557 kcal/mol, population = 41.38 %

C -1.215356 -1.646778 -0.136190
C -2.137445 -1.144633 -1.255727
C -2.106451 0.399458 -1.163079
C -1.213738 0.725096 0.095237
C 0.304034 0.762710 -0.342213
C 1.228218 0.757425 0.904232
C 2.685543 0.661604 0.533814
C 3.069318 -0.516552 -0.335094
C 2.092517 -0.672432 -1.516463
C 0.613974 -0.497686 -1.186684
C -1.405861 -0.558793 0.895837
C -1.297127 -0.742906 2.337589
C 0.611437 2.026119 -1.157997
C 3.161284 -1.776998 0.547316
O 0.168145 -1.696506 -0.524769
O -2.595574 -0.692421 1.700355
O -3.459086 0.849125 -1.022073
C -1.657018 1.987735 0.821559
O 3.515941 1.458954 0.948104
H -1.465417 -2.645591 0.232285
H 0.085702 -0.409102 -2.149349
H 4.070464 -0.308709 -0.731830
H 1.078167 1.650332 1.516229
H 1.004859 -0.123851 1.517445
H 2.348095 0.065219 -2.284783
H 2.227838 -1.658927 -1.976228
H -3.156589 -1.482372 -1.041910
H -1.853143 -1.527684 -2.240227
H 3.484433 -2.623213 -0.067574
H 3.893397 -1.633379 1.348925
H 2.194816 -2.030108 0.989835
H -0.008226 2.100568 -2.056433
H 0.448667 2.920801 -0.550467
H 1.658630 2.040361 -1.477108
H -1.072878 0.114514 2.973034
H -1.015504 -1.719247 2.733866
H -1.676954 0.849837 -2.066544

H -3.465044 1.811991 -1.097504
H -1.750124 2.832105 0.131024
H -2.629665 1.826600 1.292031
H -0.948333 2.275076 1.603012
H -3.800855 0.101107 -0.058529
H -1.040640 2.472329 1.327169
H -2.086708 2.691554 -0.084091
H -2.643823 1.730984 1.288067

12b-c3 , delta G = 0.7341 kcal/mol, population = 13.15 %

C -0.848525 -1.634387 -0.000669
C -1.911977 -1.389996 -1.076039
C -2.137463 0.147512 -1.092642
C -1.197021 0.721289 0.034707
C 0.256813 0.947554 -0.538916
C 1.272213 1.214922 0.620488
C 2.193016 0.059164 0.937040
C 3.031711 -0.420300 -0.232019
C 2.221139 -0.336407 -1.552613
C 0.720976 -0.321998 -1.291407
C -1.121174 -0.492714 0.950305
C -0.894566 -0.527223 2.387004
C 0.266491 2.149987 -1.493352
C 3.662948 -1.786702 0.011695
O 0.486430 -1.511547 -0.512793
O -2.229234 -0.725116 1.851273
O -3.512800 0.487708 -0.896862
C -1.770114 1.976967 0.679748
O 2.323335 -0.379460 2.070732
H -0.904315 -2.618596 0.473035
H 0.183606 -0.389479 -2.250536
H 3.837935 0.329999 -0.289370
H 1.937808 2.030143 0.301703
H 0.783207 1.537348 1.540626
H 2.494877 0.549671 -2.132092
H 2.458857 -1.205458 -2.175681
H -2.842646 -1.883350 -0.776030
H -1.619101 -1.796352 -2.048883
H 4.254258 -1.790592 0.931327
H 2.888270 -2.555255 0.094845
H 4.320955 -2.048530 -0.823075
H 1.252836 2.271217 -1.950113
H -0.466292 2.047264 -2.299050
H 0.046864 3.070623 -0.945579
H -0.763805 0.404773 2.936330
H -0.446196 -1.413715 2.834412
H -1.888752 0.579701 -2.065271

Table S20. Experimental (Exptl.) of **12** and the calculated ^{13}C chemical shift values of possible isomers (**12a** and **12b**) with GIAO ^{13}C NMR calculations with STS protocol.

Carbon no.	Exptl.	12a	12b
1	81.2	79.93	79.24
2	37.9	39.43	38.94
3	73.6	74.82	74.39
4	50.4	51.35	51.19
5	50	48.84	48.10
6	46.2	45.47	42.18
7	215.3	215.58	216.60
8	40.4	39.85	42.93
9	37.9	36.50	33.05
10	72.6	71.16	71.11
11	66.8	67.49	67.05
12	48.1	48.76	48.47
13	14.1	18.53	18.79
14	18.9	14.37	19.77
15	6.3	7.62	7.89
	MAE	1.48	1.90
	RMS	1.92	2.38
	Pmean	9.28%	3.97%
	Prel	100.00%	0.00%

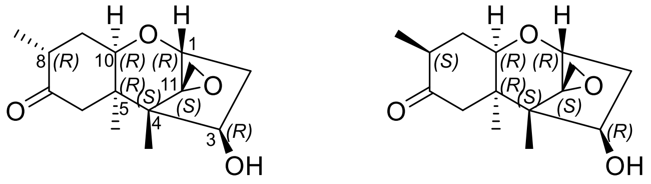

12a **12b**

Table S21. Geometry data of conformers of structure **13a** and **13b**

13a

13a-c2 , delta G = 0.0000 kcal/mol, population = 37.23 %

C 0.665069 -1.677831 -1.555940
C -0.598183 -0.995472 -2.095631
C -1.128498 -0.144003 -0.922385
C -0.206881 -0.472709 0.309329
C 1.031912 0.502873 0.280073
C 2.139240 0.042508 1.246295
C 3.334758 0.991649 1.249293
C 3.783827 1.363157 -0.145862
C 3.001055 1.138421 -1.205320
C 1.648925 0.491318 -1.138471
C 0.297976 -1.852238 -0.100282
C 0.787389 -2.915813 0.764109
C -0.949757 -0.449819 1.638871
C 0.591651 1.931970 0.638613
C 5.115815 2.042635 -0.262500
C -3.374671 0.402034 -0.259216
C -4.686174 -0.220008 -0.020397
C -5.787276 0.408084 0.419753
C -5.979793 1.840966 0.770923
O 1.835951 -0.853905 -1.648670
O -0.536199 -2.993192 0.184534
O -2.495596 -0.551224 -0.666380
O -3.077662 1.577736 -0.132526
O 4.412618 0.436072 2.023419
H 0.911105 -2.616740 -2.059937
H 0.976352 1.033002 -1.825549
H 3.081950 1.920558 1.782337
H 1.762307 -0.047818 2.270371
H 2.504452 -0.944758 0.937721
H 3.341939 1.429562 -2.198301
H -1.335217 -1.765785 -2.341870
H -0.405248 -0.406236 -2.996798
H 5.307630 2.360947 -1.290550
H 5.156526 2.921687 0.393713
H 5.926619 1.379768 0.058551
H 1.368680 2.655685 0.375885
H -0.322257 2.225084 0.114667
H 0.402245 2.016436 1.713222
H 0.800652 -2.768058 1.844227
H 1.505673 -3.636274 0.371009
H -1.138389 0.922610 -1.149212
H -4.732584 -1.286725 -0.220601
H -1.447242 0.510473 1.795548
H -1.707040 -1.236750 1.658955
H -0.268446 -0.617995 2.477071
H -6.672247 -0.217851 0.542266
H 4.686733 -0.383188 1.589379
H -6.337523 1.915439 1.806040
H -5.075419 2.434321 0.652838
H -6.778412 2.263321 0.147081

13a-c4 , delta G = 0.1617 kcal/mol, population = 28.33 %

C 0.315460 -1.475281 -1.515866
C -0.826384 -0.603824 -2.054028
C -1.213357 0.323473 -0.881860
C -0.331973 -0.123468 0.339197
C 1.047637 0.642725 0.275823
C 2.079869 0.023760 1.236626
C 3.412575 0.768185 1.213305
C 3.893892 1.052432 -0.191343
C 3.071858 0.940282 -1.238933
C 1.636539 0.513021 -1.148441
C -0.057777 -1.570250 -0.053755
C 0.262397 -2.688024 0.821259
C -1.034642 0.039067 1.680999
C 0.843581 2.127236 0.619252
C 5.312264 1.520011 -0.331233
C -3.360816 1.146004 -0.181075
C -4.755291 0.801107 0.138514
C -5.300508 -0.404017 0.368227
C -4.685620 -1.760902 0.368453
O 1.602216 -0.853371 -1.635136
O -1.062687 -2.559035 0.252609
O -2.620437 0.097881 -0.601639
O -2.932951 2.286676 -0.094732
O 4.399030 0.051154 1.976412
H 0.398743 -2.448582 -2.007619
H 1.049567 1.143434 -1.838319
H 3.321502 1.729188 1.741424
H 1.707666 0.007974 2.266208
H 2.278160 -1.013066 0.938616
H 3.440515 1.166113 -2.238974

H -1.676856 -1.248267 -2.296015
H -0.545492 -0.055897 -2.957966
H 5.535793 1.798864 -1.364300
H 5.494505 2.386761 0.317107
H 6.017385 0.743714 -0.015081
H -0.000617 2.563488 0.078697
H 0.653417 2.248041 1.690055
H 1.731840 2.713069 0.366334
H 0.309643 -2.531704 1.899254
H 0.851591 -3.519088 0.431973
H -1.087123 1.380737 -1.117167
H -5.384435 1.682631 0.230491
H -0.361571 -0.192346 2.510446
H -1.395671 1.061645 1.815650
H -1.890441 -0.637354 1.745571
H -6.365566 -0.391683 0.604879
H 4.529361 -0.804994 1.546565
H -5.039886 -2.320041 1.242194
H -5.037444 -2.318163 -0.511137
H -3.597502 -1.750333 0.353623

13a-c5 , delta G = 0.6473 kcal/mol, population = 12.47 %
C 0.664505 -1.677951 -1.568259
C -0.603113 -0.998159 -2.100728
C -1.127928 -0.147874 -0.924352
C -0.200497 -0.477464 0.303055
C 1.036304 0.500532 0.270271
C 2.144947 0.038958 1.233453
C 3.334686 0.987182 1.233686
C 3.784899 1.352497 -0.163512
C 2.998888 1.137851 -1.221528
C 1.646362 0.492421 -1.151362
C 0.305386 -1.855257 -0.111011
C 0.802314 -2.918500 0.749111
C -0.938395 -0.458464 1.635460
C 0.592829 1.928512 0.630615
C 5.125494 2.014391 -0.281630
C -3.371213 0.398748 -0.252143
C -4.682385 -0.222600 -0.009326
C -5.782452 0.406448 0.431944
C -5.973904 1.840158 0.780588
O 1.832344 -0.850826 -1.666249
O -0.524612 -2.998562 0.176663
O -2.494158 -0.554783 -0.662571
O -3.073006 1.574159 -0.125069
O 4.378674 0.320130 1.971031
H 0.910383 -2.615615 -2.074604
H 0.970215 1.035901 -1.833590
H 3.074861 1.922506 1.760369
H 1.768318 -0.051082 2.257500
H 2.517837 -0.944559 0.926993
H 3.339788 1.426770 -2.215188
H -1.340154 -1.769844 -2.342716
H -0.416383 -0.408299 -3.002790
H 5.928213 1.330897 0.020030
H 5.318811 2.338861 -1.307246
H 5.185940 2.890828 0.378153
H 1.365482 2.654873 0.362262
H -0.325751 2.217722 0.112685
H 0.410101 2.013080 1.706383
H 0.821638 -2.772259 1.829283
H 1.520656 -3.636030 0.351016
H -1.138688 0.918805 -1.150519
H -4.729329 -1.289568 -0.208074
H -1.433934 0.501936 1.797792
H -1.696645 -1.244424 1.655366
H -0.254321 -0.631123 2.470442
H -6.667415 -0.218965 0.557112
H 5.041678 0.975864 2.218349
H -5.069052 2.432544 0.661298
H -6.772119 2.261949 0.155815
H -6.331682 1.916881 1.815501

13a-c6 , delta G = 0.6915 kcal/mol, population = 11.57 %
C 0.313224 -1.471140 -1.530683
C -0.831916 -0.599435 -2.061301
C -1.213856 0.324575 -0.885037
C -0.326739 -0.125068 0.331098
C 1.051765 0.642820 0.264361
C 2.083230 0.022193 1.223817
C 3.411956 0.763071 1.197827
C 3.895611 1.037280 -0.208590
C 3.072116 0.938145 -1.255183
C 1.635474 0.516388 -1.162510
C -0.052718 -1.570507 -0.067098
C 0.273777 -2.690153 0.802837

C -1.024781 0.032561 1.675975
C 0.846159 2.127294 0.608294
C 5.322300 1.478311 -0.349506
C -3.358196 1.142743 -0.169435
C -4.750487 0.795224 0.156857
C -5.293373 -0.411457 0.383895
C -4.677573 -1.767915 0.372742
O 1.598366 -0.847671 -1.654479
O -1.054844 -2.561384 0.241778
O -2.619607 0.097336 -0.599109
O -2.930872 2.283487 -0.080240
O 4.342738 -0.060326 1.928873
H 0.395132 -2.443077 -2.025321
H 1.047108 1.150494 -1.847936
H 3.317460 1.732688 1.717970
H 1.710675 0.009410 2.253129
H 2.287478 -1.013115 0.929935
H 3.441964 1.159422 -2.255846
H -1.683049 -1.244012 -2.300617
H -0.555838 -0.048924 -2.965152
H 6.011588 0.677844 -0.055012
H 5.549296 1.764052 -1.379722
H 5.529491 2.336696 0.304080
H 1.732345 2.714338 0.350692
H -0.001544 2.562135 0.072111
H 0.661773 2.248243 1.680105
H 0.327239 -2.536949 1.880930
H 0.862528 -3.518796 0.407944
H -1.089344 1.382421 -1.118404
H -5.379598 1.675854 0.257275
H -1.381933 1.055605 1.817430
H -1.882623 -0.641335 1.739209
H -0.350456 -0.206331 2.502176
H -6.356896 -0.401194 0.627466
H 5.109676 0.478435 2.157665
H -5.026505 -2.332543 1.245078
H -5.033817 -2.320103 -0.508272
H -3.589552 -1.756536 0.351794

13a-c7 , delta G = 0.7553 kcal/mol, population = 10.39 %
C 0.312639 -1.474944 -1.521632
C -0.828461 -0.601659 -2.058170
C -1.215023 0.323047 -0.884090
C -0.334681 -0.127770 0.336553
C 1.044777 0.638509 0.276322
C 2.076942 0.017658 1.236795
C 3.406548 0.767401 1.214689
C 3.900236 1.023848 -0.185624
C 3.075295 0.928974 -1.232032
C 1.636931 0.510529 -1.146760
C -0.060568 -1.573544 -0.059804
C 0.261659 -2.692695 0.812182
C -1.038390 0.031918 1.678094
C 0.838619 2.123567 0.618143
C 5.327455 1.461106 -0.325971
C -3.361577 1.146099 -0.181122
C -4.757267 0.802347 0.134309
C -5.305395 -0.403131 0.355039
C -4.693102 -1.761253 0.346521
O 1.599359 -0.853797 -1.638735
O -1.064709 -2.563357 0.245706
O -2.622595 0.098607 -0.605253
O -2.932147 2.285897 -0.090234
O 4.422109 0.019801 1.910650
H 0.395441 -2.447101 -2.015724
H 1.053813 1.145752 -1.835569
H 3.297108 1.749729 1.709448
H 1.696460 0.002105 2.265073
H 2.285542 -1.015830 0.940284
H 3.448465 1.153572 -2.230370
H -1.679336 -1.244904 -2.301973
H -0.546727 -0.051576 -2.960515
H 6.011712 0.663716 -0.017542
H 5.553351 1.740522 -1.358405
H 5.533078 2.319049 0.326953
H -0.015370 2.553959 0.088404
H 0.662408 2.247413 1.691083
H 1.720481 2.712810 0.350956
H 0.310417 -2.539376 1.890521
H 0.851138 -3.522297 0.420382
H -1.087351 1.380633 -1.117070
H -5.384624 1.684690 0.230500
H -1.897904 -0.640135 1.738463
H -0.367931 -0.207748 2.507363
H -1.393837 1.055874 1.817422
H -6.370908 -0.390245 0.589542
H 4.147582 -0.047614 2.833548

H -5.057988 -2.330040 1.209469
H -5.035387 -2.307329 -0.543821
H -3.604816 -1.752485 0.343541

13b

13b-c1 , delta G = 0.0000 kcal/mol, population = 33.40 %
C -0.576966 -2.184294 0.955712
C 0.635978 -1.609303 1.697565
C 1.099385 -0.410167 0.842338
C 0.192483 -0.412428 -0.442949
C -1.113723 0.419964 -0.135971
C -2.186807 0.194003 -1.217504
C -3.508233 0.922864 -0.956788
C -3.926588 0.897538 0.493770
C -3.110747 0.434147 1.444672
C -1.717885 -0.068029 1.200671
C -0.203849 -1.884828 -0.477610
C -0.606387 -2.677510 -1.630115
C 0.915704 0.068742 -1.694096
C -0.763257 1.914613 -0.021328
C -5.304219 1.408836 0.793912
C 3.299490 0.453711 0.395082
C 4.644129 0.016875 -0.011240
C 5.690028 0.818681 -0.263565
C 5.775857 2.302856 -0.205260
O -1.805127 -1.513267 1.273966
O 0.720145 -2.819609 -1.070518
O 2.487920 -0.632221 0.490859
O 2.927919 1.591491 0.627826
O -3.542540 2.267741 -1.485320
H -0.750818 -3.246623 1.148606
H -1.081701 0.286765 2.029770
H -4.288249 0.407708 -1.535022
H -1.824863 0.493838 -2.206198
H -2.419902 -0.874955 -1.264755
H -3.456468 0.393384 2.477050
H 1.426725 -2.365215 1.712814
H 0.405762 -1.333372 2.730651
H -5.539770 1.322470 1.858029
H -5.401419 2.459624 0.497466
H -6.054159 0.850183 0.219538
H -0.648851 2.362277 -1.013408
H -1.543580 2.459636 0.520235
H 0.170411 2.079908 0.522988
H -0.635301 -2.216511 -2.617714
H -1.262738 -3.536419 -1.484838
H 1.041925 0.536025 1.381262
H 4.765151 -1.057356 -0.118418
H 1.726926 -0.616972 -1.949110
H 0.235947 0.117676 -2.548949
H 1.341394 1.064043 -1.543827
H 6.612090 0.315870 -0.558453
H -2.971099 2.819047 -0.936890
H 6.543969 2.593831 0.523353
H 6.122301 2.684310 -1.174363
H 4.831205 2.774318 0.058338

13b-c3 , delta G = 0.2059 kcal/mol, population = 23.59 %
C -0.572459 -2.178548 0.957966
C 0.633546 -1.590821 1.701648
C 1.095782 -0.396157 0.839113
C 0.188825 -0.406406 -0.445207
C -1.124115 0.420330 -0.143502
C -2.194617 0.180181 -1.223838
C -3.524108 0.894920 -0.963877
C -3.933450 0.894811 0.486158
C -3.121714 0.425499 1.435968
C -1.727476 -0.069646 1.192666
C -0.197821 -1.881638 -0.475503
C -0.593545 -2.680250 -1.626197
C 0.914579 0.072437 -1.696155
C -0.792368 1.916864 -0.037996
C -5.303457 1.425230 0.785738
C 3.305195 0.454848 0.402144
C 4.646391 0.008867 -0.006908
C 5.700758 0.802837 -0.248311
C 5.801094 2.285413 -0.172787
O -1.806285 -1.516133 1.269738
O 0.732480 -2.813834 -1.063618
O 2.483419 -0.624163 0.484872
O 2.945373 1.593757 0.646637
O -3.540887 2.266552 -1.415621
H -0.738002 -3.241685 1.154169
H -1.093853 0.290174 2.021306
H -4.307131 0.344009 -1.514915
H -1.830588 0.477772 -2.214131

H -2.416668 -0.891209 -1.272830
H -3.469488 0.383373 2.467473
H 1.428514 -2.341980 1.728059
H 0.395771 -1.307843 2.731099
H -5.553225 1.308708 1.843813
H -5.370149 2.488041 0.526233
H -6.059686 0.905367 0.183985
H -0.550633 2.324313 -1.023595
H -1.647893 2.476404 0.344680
H 0.059415 2.101901 0.622641
H -0.622386 -2.222348 -2.615389
H -1.245702 -3.542334 -1.480500
H 1.041540 0.554423 1.370119
H 4.756858 -1.065327 -0.125444
H 1.712291 -0.625924 -1.959578
H 0.232803 0.141106 -2.548233
H 1.358934 1.058511 -1.539974
H 6.618517 0.294161 -0.546579
H -3.425432 2.253889 -2.373839
H 6.562621 2.560356 0.568973
H 6.163757 2.674116 -1.132944
H 4.858123 2.763453 0.084977

13b-c4 , delta G = 0.2715 kcal/mol, population = 21.11 %
C -0.573332 -2.179932 0.942991
C 0.638696 -1.602829 1.685195
C 1.097110 -0.399116 0.833244
C 0.181050 -0.392575 -0.444871
C -1.127773 0.432687 -0.124025
C -2.203003 0.207975 -1.204299
C -3.527579 0.915441 -0.932157
C -3.933463 0.889405 0.523815
C -3.114301 0.422029 1.468138
C -1.722110 -0.071920 1.210459
C -0.208323 -1.866799 -0.489468
C -0.612420 -2.652153 -1.646471
C 0.899457 0.099692 -1.694879
C -0.793780 1.927366 -0.002599
C -5.307451 1.403996 0.837483
C 3.306540 0.451083 0.394075
C 4.643689 0.006092 -0.029223
C 5.697217 0.800252 -0.273448
C 5.799903 2.282185 -0.188790
O -1.803725 -1.519077 1.271463
O 0.716865 -2.793725 -1.093967
O 2.481579 -0.626122 0.466500
O 2.952480 1.587652 0.657113
O -3.454500 2.272492 -1.433253
H -0.739320 -3.245039 1.127853
H -1.082270 0.277264 2.038835
H -4.309716 0.384473 -1.500955
H -1.847204 0.524390 -2.189891
H -2.424307 -0.862084 -1.265467
H -3.453697 0.375945 2.502479
H 1.432532 -2.355505 1.697557
H 0.408274 -1.331412 2.719438
H -5.551155 1.273199 1.895144
H -5.391095 2.473333 0.603583
H -6.064006 0.884712 0.235071
H -1.647826 2.482009 0.390993
H 0.061070 2.104886 0.656117
H -0.558261 2.346003 -0.984813
H -0.647092 -2.183034 -2.630186
H -1.264917 -3.514871 -1.506026
H 1.048709 0.545591 1.375131
H 4.751355 -1.067315 -0.157113
H 1.695586 -0.595737 -1.970619
H 0.212788 0.178270 -2.542058
H 1.344864 1.083942 -1.530290
H 6.611835 0.292457 -0.582664
H -4.354662 2.612562 -1.503802
H 4.857740 2.760022 0.072325
H 6.561484 2.551365 0.555115
H 6.163575 2.676302 -1.146280

13b-c2 , delta G = 0.6724 kcal/mol, population = 10.72 %
C -0.257548 -1.990091 0.927336
C 0.838743 -1.277191 1.728999
C 1.160123 -0.000594 0.923166
C 0.300658 -0.081966 -0.390041
C -1.118535 0.549534 -0.102967
C -2.118235 0.211673 -1.225536
C -3.535615 0.740457 -0.987965
C -3.987019 0.613126 0.447294
C -3.141840 0.236909 1.411074
C -1.686631 -0.061174 1.197580

C 0.114684 -1.592483 -0.482681
C -0.134594 -2.393322 -1.671994
C 0.984512 0.542219 -1.599160
C -0.985086 2.073859 0.064134
C -5.430256 0.920543 0.715509
C 3.240102 1.144441 0.557068
C 4.656053 1.016812 0.177982
C 5.289691 -0.012908 -0.405343
C 4.778181 -1.341560 -0.843216
O -1.575755 -1.506598 1.223706
O 1.180129 -2.370239 -1.067298
O 2.577782 -0.031356 0.609892
O 2.729800 2.221617 0.824014
O -3.745037 2.081828 -1.483342
H -0.286835 -3.072317 1.081727
H -1.127622 0.348701 2.056181
H -4.218618 0.138172 -1.603609
H -1.773888 0.590346 -2.193261
H -2.198388 -0.877271 -1.311573
H -3.507233 0.116891 2.430428
H 1.726375 -1.916393 1.758693
H 0.536021 -1.066597 2.758741
H -5.682345 0.771691 1.768872
H -5.663589 1.955947 0.441808
H -6.078356 0.280653 0.103218
H -1.848799 2.484767 0.597240
H -0.097939 2.352703 0.639536
H -0.912466 2.563951 -0.911615
H -0.193827 -1.907647 -2.646232
H -0.669499 -3.338841 -1.575887
H 0.966139 0.912359 1.486975
H 5.217176 1.925220 0.381131
H 1.886693 -0.017898 -1.856740
H 0.326883 0.536686 -2.472168
H 1.270003 1.577672 -1.397265
H 6.350679 0.148925 -0.601766
H -3.290542 2.695064 -0.892911
H 5.168169 -2.118862 -0.171148
H 3.692241 -1.412656 -0.853248
H 5.175226 -1.574238 -1.837983

13b-c6 , delta G = 0.9960 kcal/mol, population = 6.21 %

C -0.257898 -1.987984 0.932269
C 0.829756 -1.266182 1.738552
C 1.153890 0.006842 0.927939
C 0.297518 -0.079348 -0.386220
C -1.127035 0.547454 -0.106085
C -2.119223 0.201834 -1.232384
C -3.540741 0.720775 -0.999637
C -3.992160 0.614963 0.434298
C -3.155085 0.225339 1.398231
C -1.699136 -0.067507 1.190605
C 0.118996 -1.591240 -0.476598
C -0.122578 -2.395257 -1.665190
C 0.987720 0.542824 -1.593147
C -1.009411 2.070139 0.058784
C -5.430267 0.946230 0.698376
C 3.242809 1.141575 0.570934
C 4.658321 1.007304 0.191143
C 5.286921 -0.022616 -0.397144
C 4.768907 -1.346609 -0.841412
O -1.580740 -1.513172 1.219346
O 1.189722 -2.366649 -1.055570
O 2.572289 -0.029778 0.614332
O 2.741207 2.220474 0.846598
O -3.729247 2.092140 -1.410194
H -0.280106 -3.070151 1.088825
H -1.146475 0.345166 2.051839
H -4.223959 0.088690 -1.594579
H -1.767527 0.578780 -2.199764
H -2.191759 -0.887564 -1.321955
H -3.526490 0.100801 2.414745
H 1.719227 -1.902196 1.779297
H 0.518128 -1.050781 2.764626
H -6.088934 0.345718 0.058199
H -5.695664 0.768042 1.743954
H -5.631982 1.996895 0.460304
H -0.770315 2.539926 -0.899343
H -1.953696 2.495236 0.403325
H -0.230158 2.345782 0.775183
H -0.179228 -1.911806 -2.640823
H -0.654299 -3.342757 -1.570252
H 0.962839 0.923735 1.485674
H 5.224249 1.911637 0.399160
H 1.297732 1.569198 -1.382132
H 1.875725 -0.034793 -1.861565
H 0.325803 0.562547 -2.462863

H 6.348769 0.134928 -0.592554
H -3.587774 2.127698 -2.364327
H 3.682673 -1.412671 -0.849914
H 5.163229 -1.575610 -1.838159
H 5.156702 -2.129229 -0.174258

13b-c7 , delta G = 1.1282 kcal/mol, population = 4.96 %

C -0.256917 -1.991647 0.916914
C 0.836661 -1.279648 1.723956
C 1.156857 0.001834 0.925074
C 0.291106 -0.068473 -0.383958
C -1.130442 0.556668 -0.086295
C -2.128083 0.227379 -1.213912
C -3.544466 0.739330 -0.969387
C -3.990932 0.606271 0.468727
C -3.147767 0.218434 1.427964
C -1.693303 -0.072630 1.207227
C 0.110236 -1.579130 -0.490075
C -0.140831 -2.369022 -1.686234
C 0.973550 0.566966 -1.588399
C -1.010286 2.077010 0.096238
C -5.432041 0.919250 0.744725
C 3.246019 1.136473 0.568841
C 4.658398 1.003653 0.176965
C 5.280317 -0.020385 -0.428448
C 4.756015 -1.337475 -0.885645
O -1.577220 -1.518703 1.218464
O 1.175758 -2.349004 -1.085683
O 2.572776 -0.033607 0.600754
O 2.749465 2.212709 0.863330
O -3.638782 2.110712 -1.424769
H -0.279047 -3.075494 1.061398
H -1.133559 0.329303 2.068851
H -4.228513 0.128880 -1.583193
H -1.787327 0.624911 -2.175180
H -2.199410 -0.859897 -1.318028
H -3.511720 0.089017 2.446791
H 1.725756 -1.916865 1.752407
H 0.531717 -1.075026 2.754248
H -6.089505 0.323634 0.098298
H -5.691791 0.720253 1.787824
H -5.652656 1.974925 0.539670
H -0.227111 2.344550 0.811364
H -0.778426 2.558104 -0.857892
H -1.952860 2.497267 0.451942
H -0.204058 -1.873575 -2.655415
H -0.672924 -3.317033 -1.598687
H 0.970979 0.913165 1.493445
H 5.228055 1.903953 0.392183
H 1.856856 -0.010753 -1.871574
H 0.304255 0.601619 -2.451887
H 1.289395 1.589236 -1.366326
H 6.341130 0.137096 -0.629500
H -4.574074 2.332408 -1.508710
H 5.149599 -2.129748 -0.233433
H 3.669687 -1.402011 -0.884444
H 5.140202 -1.553113 -1.889349

Table S22. Experimental (Exptl.) of **13** and the calculated ^{13}C chemical shift values of possible isomers (**13a** and **13b**) with GIAO ^{13}C NMR calculations with STS protocol.

Carbon no.	Exptl.	13a	13b
1	79.3	78.59	78.66
2	36.8	34.94	35.02
3	73.8	73.18	73.34
4	49.1	50.60	50.68
5	42.4	41.91	40.11
6	35.1	35.59	33.81
7	69	68.27	67.50
8	142.3	141.90	139.57
9	121.1	122.98	123.62
10	70.4	69.80	69.96

11	65.4	65.85	66.02
12	47.8	47.52	47.83
13	6	7.05	7.03
14	16.9	17.25	19.22
15	18.8	19.39	20.89
	166.4	166.29	166.91
	120.5	119.16	119.21
	145.9	146.93	148.21
	15.5	15.31	14.91
	MAE	0.77	1.37
	RMS	0.93	1.59
	Pmean	49.02%	22.62%
	Prel	100.00%	0.00%

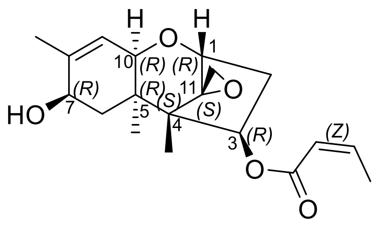

13a

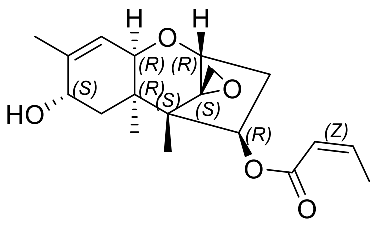

13b

Table S23. Geometry data of conformers of structure **13a** for ECD calculation.

This ECD calculation was calculated by time-dependent density functional theory (TDDFT) at the ωB97xd/6-311+G* level with the IEFPCM solvent model.

13a

13a-c2 , delta G = 0.0000 kcal/mol, population = 33.72 %

C -0.660175 -1.673634 1.555372
C 0.597633 -0.983282 2.097475
C 1.128384 -0.132802 0.923651
C 0.207386 -0.461752 -0.308095
C -1.036107 0.508672 -0.277130
C -2.138934 0.046392 -1.247972
C -3.338620 0.990097 -1.249542
C -3.794136 1.352318 0.145958
C -3.010745 1.131687 1.206180
C -1.654976 0.492092 1.140201
C -0.292601 -1.843685 0.099222
C -0.779241 -2.906772 -0.767086
C 0.948562 -0.432524 -1.638517
C -0.602544 1.941185 -0.629907
C -5.131159 2.021740 0.262784
C 3.381278 0.402273 0.262843
C 4.687545 -0.228263 0.021196
C 5.793243 0.390828 -0.421794
C 5.999335 1.820698 -0.775485
O -1.836514 -0.856308 1.647959
O 0.546693 -2.981836 -0.187483
O 2.495157 -0.544948 0.666783
O 3.092816 1.581569 0.141115
O -4.411216 0.434201 -2.031950
H -0.900955 -2.614638 2.057955
H -0.986084 1.034568 1.829775
H -3.087551 1.923715 -1.775046
H -1.757999 -0.036740 -2.271094
H -2.500623 -0.944137 -0.946015
H -3.354094 1.421362 2.198872
H 1.336895 -1.749126 2.350882
H 0.398552 -0.391442 2.995521
H -5.178716 2.899838 -0.394313
H -5.936674 1.351268 -0.056278
H -5.324692 2.339428 1.290738
H 0.302879 2.240138 -0.094542
H -0.403715 2.028740 -1.702477
H -1.386960 2.658897 -0.372991
H -0.792572 -2.756503 -1.846655
H -1.494348 -3.630491 -0.374705
H 1.140932 0.933621 1.150435
H 4.727673 -1.295328 0.221306
H 0.267993 -0.608133 -2.475653
H 1.436042 0.532688 -1.796536
H 1.714340 -1.211151 -1.660203
H 6.672389 -0.242906 -0.545508
H -4.684281 -0.389373 -1.604657
H 6.356327 1.888771 -1.811313
H 5.102434 2.425175 -0.656861
H 6.804798 2.234612 -0.154764

13a-c4 , delta G = 0.1798 kcal/mol, population = 24.88 %

C -0.311645 -1.471973 1.517779
C 0.827715 -0.597046 2.055244
C 1.214183 0.328381 0.881604
C 0.332840 -0.120030 -0.338609
C -1.048284 0.644630 -0.274808
C -2.078548 0.025344 -1.237839
C -3.412602 0.767170 -1.213712
C -3.896359 1.046673 0.191020
C -3.073722 0.938560 1.238912
C -1.637295 0.514917 1.149209
C 0.058955 -1.566807 0.055009
C -0.265985 -2.683430 -0.819504
C 1.034121 0.042497 -1.681181
C -0.846873 2.129897 -0.616478
C -5.316688 1.508032 0.331596
C 3.368527 1.144565 0.185277
C 4.762439 0.795874 -0.130617
C 5.303713 -0.410626 -0.364996
C 4.682753 -1.764112 -0.377476
O -1.600400 -0.852357 1.637387
O 1.062568 -2.557092 -0.253611
O 2.622369 0.100690 0.600561
O 2.946480 2.289270 0.101478
O -4.396785 0.050114 -1.981156
H -0.392465 -2.445167 2.010079

H -1.051839 1.145802 1.839452
H -3.322884 1.730505 -1.737705
H -1.704477 0.013416 -2.266746
H -2.275694 -1.012719 -0.943690
H -3.443118 1.165956 2.238465
H 1.678546 -1.239488 2.301417
H 0.544746 -0.046682 2.956994
H -6.018033 0.726188 0.020238
H -5.539691 1.789258 1.364171
H -5.504051 2.371704 -0.319479
H -0.647535 2.251337 -1.685499
H -1.739790 2.712120 -0.371988
H -0.010874 2.569414 -0.065899
H -0.315019 -2.526244 -1.897113
H -0.855073 -3.513555 -0.428501
H 1.089053 1.385622 1.116687
H 5.397244 1.674300 -0.214543
H 0.361784 -0.193997 -2.509637
H 1.390298 1.066465 -1.818688
H 1.893253 -0.629760 -1.744748
H 6.369888 -0.401709 -0.596283
H -4.526558 -0.808205 -1.554656
H 3.594630 -1.747627 -0.367547
H 5.039245 -2.318215 -1.253489
H 5.029285 -2.328898 0.499501

13a-c5 , delta G = 0.4721 kcal/mol, population = 15.18 %
C -0.659853 -1.677567 1.565681
C 0.601426 -0.989074 2.101903
C 1.126978 -0.138557 0.925851
C 0.201070 -0.468043 -0.302374
C -1.041186 0.503897 -0.267393
C -2.145703 0.039868 -1.234419
C -3.341586 0.980600 -1.232349
C -3.793767 1.343877 0.164698
C -3.007670 1.130283 1.223454
C -1.652958 0.489637 1.153062
C -0.298685 -1.849095 0.108131
C -0.789879 -2.912442 -0.755142
C 0.937791 -0.440984 -1.635327
C -0.605888 1.935531 -0.622493
C -5.133557 2.007485 0.281279
C 3.376852 0.399467 0.257924
C 4.683504 -0.228811 0.012650
C 5.787059 0.392470 -0.432581
C 5.989613 1.822985 -0.785862
O -1.834192 -0.858044 1.662967
O 0.538464 -2.988292 -0.181017
O 2.493181 -0.549324 0.663773
O 3.085989 1.578240 0.136804
O -4.384247 0.307310 -1.967931
H -0.899918 -2.617920 2.069823
H -0.979880 1.031863 1.838658
H -3.089867 1.917200 -1.759570
H -1.766042 -0.042809 -2.257916
H -2.511416 -0.948309 -0.933714
H -3.348392 1.422032 2.216490
H 1.341086 -1.756000 2.350938
H 0.407635 -0.397474 3.001252
H -5.188763 2.885269 -0.376889
H -5.937209 1.328124 -0.027109
H -5.329191 2.328668 1.307540
H -1.387871 2.654829 -0.362718
H 0.302789 2.232540 -0.091580
H -0.412198 2.022305 -1.696059
H -0.807904 -2.763277 -1.834790
H -1.504117 -3.635055 -0.359181
H 1.139495 0.927813 1.152611
H 4.725811 -1.295859 0.212402
H 1.423498 0.524485 -1.797307
H 1.704572 -1.218571 -1.657716
H 0.254827 -0.619429 -2.469883
H 6.667166 -0.239499 -0.558404
H -5.044932 0.962733 -2.224098
H 5.090921 2.424965 -0.668122
H 6.793052 2.238992 -0.163869
H 6.347938 1.892258 -1.821116

13a-c6 , delta G = 0.5080 kcal/mol, population = 14.29 %
C -0.309477 -1.472106 1.528980
C 0.832095 -0.596721 2.060910
C 1.213730 0.327430 0.884694
C 0.327261 -0.122125 -0.331614
C -1.053471 0.642959 -0.263349
C -2.083190 0.021587 -1.224471
C -3.414958 0.757278 -1.196244

C -3.897995 1.033193 0.210034
C -3.073692 0.935008 1.256673
C -1.636864 0.514387 1.163210
C 0.055149 -1.568545 0.064845
C -0.272874 -2.686585 -0.806659
C 1.024091 0.038394 -1.676831
C -0.852448 2.128416 -0.605165
C -5.322978 1.479759 0.350173
C 3.365350 1.141476 0.177349
C 4.757366 0.791298 -0.145104
C 5.296569 -0.415980 -0.380182
C 4.674635 -1.769094 -0.386296
O -1.597504 -0.852242 1.653049
O 1.057893 -2.558883 -0.246560
O 2.620625 0.098828 0.598123
O 2.943479 2.286238 0.093113
O -4.346236 -0.071439 -1.922701
H -0.388201 -2.444794 2.022626
H -1.049089 1.146595 1.850223
H -3.327242 1.725558 -1.718891
H -1.709926 0.013278 -2.253542
H -2.281791 -1.015900 -0.933930
H -3.442266 1.161815 2.256715
H 1.683901 -1.239032 2.304043
H 0.552996 -0.045354 2.963239
H -5.549829 1.764511 1.380746
H -5.523869 2.339403 -0.303440
H -6.016113 0.684232 0.051424
H -0.015649 2.568109 -0.055880
H -0.655480 2.250258 -1.674534
H -1.745179 2.710122 -0.358749
H -0.326908 -2.531165 -1.884286
H -0.860235 -3.516228 -0.412030
H 1.089785 1.384861 1.119312
H 5.392158 1.669228 -0.234179
H 1.377528 1.062760 -1.818299
H 1.884546 -0.632082 -1.740910
H 0.350216 -0.202295 -2.502758
H 6.361446 -0.408308 -0.617401
H -5.109705 0.468948 -2.161188
H 5.025151 -2.331866 0.490399
H 3.586577 -1.751871 -0.370836
H 5.026339 -2.325672 -1.262659

13a-c7 , delta G = 0.6149 kcal/mol, population = 11.93 %

C -0.309677 -1.475423 1.520089
C 0.828051 -0.598844 2.058079
C 1.214568 0.326004 0.884104
C 0.335332 -0.124827 -0.336902
C -1.046394 0.638572 -0.275845
C -2.076273 0.015976 -1.237711
C -3.409236 0.759734 -1.214025
C -3.902140 1.019453 0.185970
C -3.076630 0.927055 1.232655
C -1.638052 0.509353 1.146992
C 0.062486 -1.571385 0.057769
C -0.261303 -2.688626 -0.816056
C 1.038014 0.037786 -1.678661
C -0.844535 2.124314 -0.616528
C -5.327666 1.462936 0.326832
C 3.368714 1.145153 0.190742
C 4.764187 0.798688 -0.120536
C 5.308559 -0.407349 -0.350014
C 4.690066 -1.762061 -0.360194
O -1.599022 -0.857091 1.637746
O 1.067035 -2.561136 -0.249514
O 2.623407 0.100343 0.605099
O 2.945133 2.289236 0.105944
O -4.422249 0.004014 -1.906927
H -0.389948 -2.448239 2.013254
H -1.055760 1.143049 1.837274
H -3.306182 1.740242 -1.712808
H -1.697051 0.003903 -2.266123
H -2.279370 -1.019437 -0.943955
H -3.448424 1.158773 2.230058
H 1.679387 -1.240108 2.305598
H 0.543434 -0.047967 2.958984
H -5.529728 2.318914 -0.329815
H -6.017160 0.667708 0.023990
H -5.550648 1.748228 1.358340
H -0.657045 2.247905 -1.687462
H -1.732705 2.708800 -0.360286
H -0.000645 2.559726 -0.074747
H -0.310139 -2.532618 -1.893843
H -0.850058 -3.518932 -0.424942
H 1.087373 1.383214 1.118251
H 5.397383 1.678271 -0.204451

H 1.899511 -0.631607 -1.740213

H 0.367627 -0.202357 -2.507688

H 1.390720 1.062845 -1.817281

H 6.375468 -0.397028 -0.577775

H -4.149638 -0.059292 -2.831235

H 5.056918 -2.321642 -1.228306

H 5.027328 -2.320130 0.524696

H 3.601838 -1.747214 -0.362016

Table S24. ITS sequence of *Trichothecium* sp. DWS815

CGAGTTTACACTCCCAACCCTTTGTGAACCTTACCTCACCGTTGCTTCGG
CGGACCGCCCCGGGTGCTGCGCGCCCCGGACCCAGGCGCCCGCCGGGGAC
CAACCCAAACCCTGTTTTTTTTTTTAAATATGTGTATCATCTGAGCGAGC
CGAAAGGCAACGAAACAAAAACAATCAAAACTTTCAACAACGGATCTCTT
GGTTCTGGCATCGATGAAGAACGCAGCGAAATGCGATAAGTAATGTGAAT
TGCAGAATTCAGTGAATCATCGAATCTTTGAACGCACATTGCGCCCGCCA
GTATTCTGGCGGGCATGCCTGTCCGAGCGTCATTTCAACCCTCGGGCCCC
CGCCTCCCCGCGGGCGGCCCGGCGTTGGGGCTCAGGCGCCGTCTCTGCTC
GGCGCCTGCCCCCTAAATGCAG

Table S25. Maximum likelihood phylogenetic tree was constructed using ITS sequences in MEGA 12. Bootstrap support values of maximum likelihood above 50% are shown at the nodes [1,2].

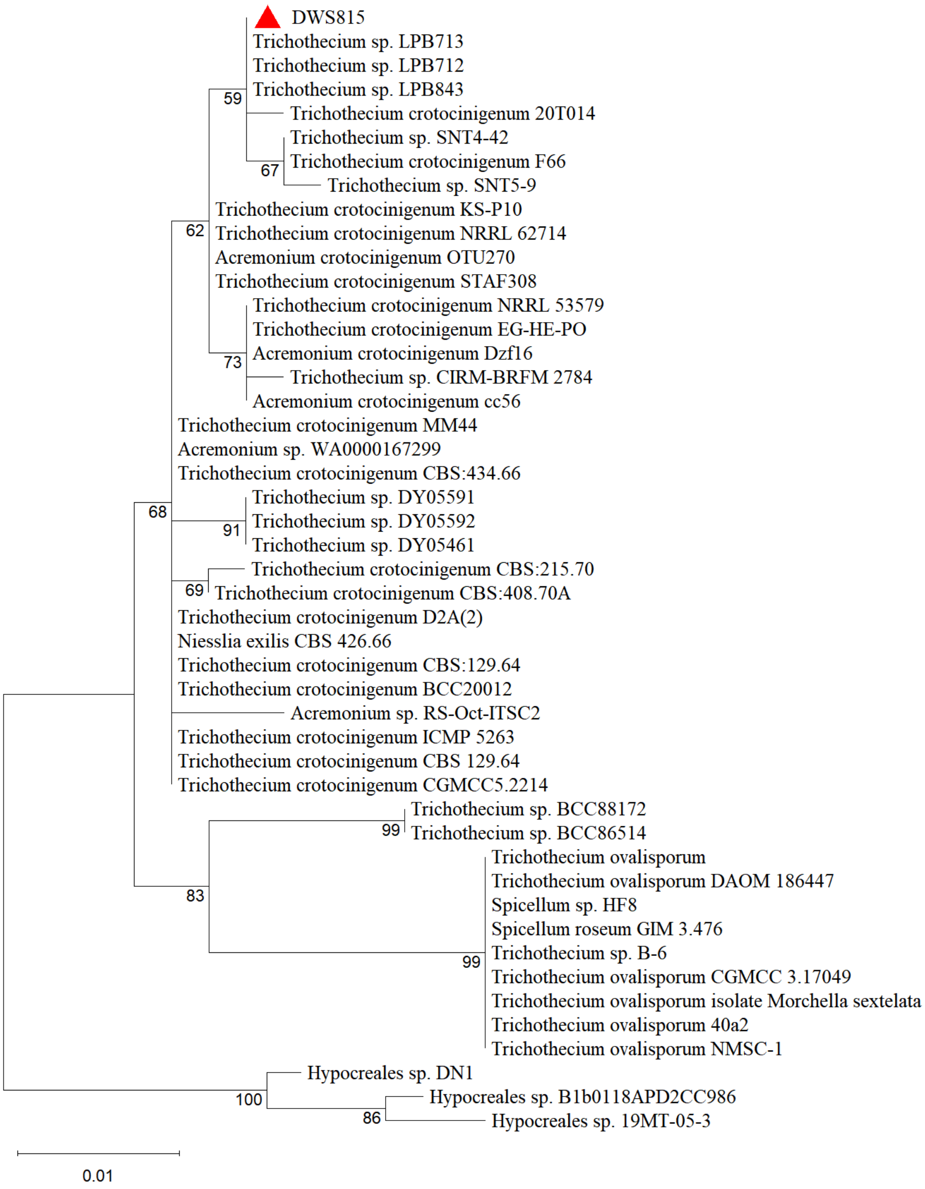

Reference:

1 Kumar S, Stecher G, Suleski M, Sanderford M, Sharma S, Tamura K. MEGA12: Molecular Evolutionary Genetic Analysis Version 12 for Adaptive and Green Computing. Molecular Biology and Evolution. 2024; 41. https://doi.org/10.1093/molbev/msae263.

2 https://blast.ncbi.nlm.nih.gov/blast/Blast.cgi

Table S26. The pairwise genetic distance matrix of ITS sequences based on the Kimura 2-parameter model (complete deletion)

	DWS815	Trichothecium sp. LPB843	Trichothecium sp. LPB712
DWS815		0	0
Trichothecium sp. LPB843			0
Trichothecium sp. LPB712			
Trichothecium crotocinigenum EG-HE-PO			
Trichothecium sp. CIRM-BRFM 2784			
Trichothecium crotocinigenum NRRL 53579			
Trichothecium crotocinigenum F66			
Trichothecium crotocinigenum ICMP 5263			
Niesslia exilis CBS 426.66			
Trichothecium crotocinigenum 20T014			
Acremonium crotocinigenum Dzf16			
Trichothecium crotocinigenum CBS 129.64			
Trichothecium crotocinigenum CBS:434.66			
Trichothecium crotocinigenum NRRL 62714			
Acremonium crotocinigenum OTU270			
Trichothecium crotocinigenum KS-P10			
Trichothecium sp. DY05591			
Trichothecium crotocinigenum BCC20012			
Trichothecium sp. DY05592			
Spicellum sp. HF8			
Acremonium sp. RS-Oct-ITSC2			
Trichothecium crotocinigenum CBS:215.70			
Trichothecium sp. DY05461			
Trichothecium ovalisporum			
Trichothecium crotocinigenum STAF308			
Trichothecium crotocinigenum D2A(2)			
Trichothecium sp. SNT4-42			
Acremonium sp. WA0000167299			
Trichothecium ovalisporum DAOM 186447			
Trichothecium ovalisporum 40a2			
Trichothecium ovalisporum CGMCC 3.17049			
Trichothecium crotocinigenum MM44			
Trichothecium crotocinigenum CBS:408.70A			
Trichothecium sp. B-6			
Trichothecium crotocinigenum CGMCC5.2214			
Trichothecium sp. LPB713			
Spicellum roseum GIM 3.476			
Trichothecium crotocinigenum CBS:129.64			
Acremonium crotocinigenum cc56			
Trichothecium sp. SNT5-9			
Trichothecium sp. BCC88172			
Hypocreales sp. DN1			
Trichothecium ovalisporum NMSC-1			
Trichothecium sp. BCC86514			
Hypocreales sp. B1b0118APD2CC986			
Hypocreales sp. 19MT-05-3			

Trichothecium crotocinigenum EG-HE-[illegible]	Trichothecium sp. CIRM-BRFM 2784	Trichothecium crotocinigenum NRRL 53[illegible]	Trichothecium crotocinigenum F66	Trichothecium crotocinigenum ICMP 52[illegible]
0.0047	0.0071	0.0047	0.0023	0.0047
0.0047	0.0071	0.0047	0.0023	0.0047
0.0047	0.0071	0.0047	0.0023	0.0047
	0.0023	0	0.0071	0.0047
		0.0023	0.0094	0.0071
			0.0071	0.0047
				0.0071

Niesslia exilis CBS 426.66	Trichothecium crotocinigenum 20T014	Acremonium crotocinigenum Dzf16	Trichothecium crotocinigenum CBS 129	Trichothecium crotocinigenum CBS:434
0.0047	0.0023	0.0047	0.0047	0.0047
0.0047	0.0023	0.0047	0.0047	0.0047
0.0047	0.0023	0.0047	0.0047	0.0047
0.0047	0.0071	0	0.0047	0.0047
0.0071	0.0094	0.0023	0.0071	0.0071
0.0047	0.0071	0	0.0047	0.0047
0.0071	0.0047	0.0071	0.0071	0.0071
0	0.0071	0.0047	0	0
	0.0071	0.0047	0	0
		0.0071	0.0071	0.0071
			0.0047	0.0047
				0

Trichothecium crotocinigenum NRRL 62	Acremonium crotocinigenum OTU270	Trichothecium crotocinigenum KS-P10	Trichothecium sp. DY05591	Trichothecium crotocinigenum BCC200
0.0023	0.0023	0.0023	0.0094	0.0047
0.0023	0.0023	0.0023	0.0094	0.0047
0.0023	0.0023	0.0023	0.0094	0.0047
0.0023	0.0023	0.0023	0.0094	0.0047
0.0047	0.0047	0.0047	0.0118	0.0071
0.0023	0.0023	0.0023	0.0094	0.0047
0.0047	0.0047	0.0047	0.0119	0.0071
0.0023	0.0023	0.0023	0.0047	0
0.0023	0.0023	0.0023	0.0047	0
0.0047	0.0047	0.0047	0.0119	0.0071
0.0023	0.0023	0.0023	0.0094	0.0047
0.0023	0.0023	0.0023	0.0047	0
0.0023	0.0023	0.0023	0.0047	0
	0	0	0.0071	0.0023
		0	0.0071	0.0023
			0.0071	0.0023
				0.0047

Trichothecium sp. DY05592	Spicellum sp. HF8	Acremonium sp. RS-Oct-ITSC2	Trichothecium crotocinigenum CBS:215	Trichothecium sp. DY05461
0.0094	0.024	0.0119	0.0094	0.0094
0.0094	0.024	0.0119	0.0094	0.0094
0.0094	0.024	0.0119	0.0094	0.0094
0.0094	0.0291	0.0119	0.0094	0.0094
0.0118	0.0316	0.0143	0.0118	0.0118
0.0094	0.0291	0.0119	0.0094	0.0094
0.0119	0.0265	0.0144	0.0118	0.0119
0.0047	0.024	0.0071	0.0047	0.0047
0.0047	0.024	0.0071	0.0047	0.0047
0.0119	0.0265	0.0144	0.0118	0.0119
0.0094	0.0291	0.0119	0.0094	0.0094
0.0047	0.024	0.0071	0.0047	0.0047
0.0047	0.024	0.0071	0.0047	0.0047
0.0071	0.0265	0.0095	0.007	0.0071
0.0071	0.0265	0.0095	0.007	0.0071
0.0071	0.0265	0.0095	0.007	0.0071
0	0.029	0.0119	0.0094	0
0.0047	0.024	0.0071	0.0047	0.0047
	0.029	0.0119	0.0094	0
		0.0316	0.029	0.029
			0.0118	0.0119
				0.0094

Trichothecium ovalisporum	Trichothecium crotocinigenum STAF308	Trichothecium crotocinigenum D2A(2)	Trichothecium sp. SNT4-42	Acremonium sp. WA0000167299
0.024	0.0023	0.0047	0.0023	0.0047
0.024	0.0023	0.0047	0.0023	0.0047
0.024	0.0023	0.0047	0.0023	0.0047
0.0291	0.0023	0.0047	0.0071	0.0047
0.0316	0.0047	0.0071	0.0094	0.0071
0.0291	0.0023	0.0047	0.0071	0.0047
0.0265	0.0047	0.0071	0	0.0071
0.024	0.0023	0	0.0071	0
0.024	0.0023	0	0.0071	0
0.0265	0.0047	0.0071	0.0047	0.0071
0.0291	0.0023	0.0047	0.0071	0.0047
0.024	0.0023	0	0.0071	0
0.024	0.0023	0	0.0071	0
0.0265	0	0.0023	0.0047	0.0023
0.0265	0	0.0023	0.0047	0.0023
0.0265	0	0.0023	0.0047	0.0023
0.029	0.0071	0.0047	0.0119	0.0047
0.024	0.0023	0	0.0071	0
0.029	0.0071	0.0047	0.0119	0.0047
0	0.0265	0.024	0.0265	0.024
0.0316	0.0095	0.0071	0.0144	0.0071
0.029	0.007	0.0047	0.0118	0.0047
0.029	0.0071	0.0047	0.0119	0.0047
	0.0265	0.024	0.0265	0.024
		0.0023	0.0047	0.0023
			0.0071	0
				0.0071

Trichothecium ovalisporum DAOM 186	Trichothecium ovalisporum 40a2	Trichothecium ovalisporum CGMCC 3.1	Trichothecium crotocinigenum MM44	Trichothecium crotocinigenum CBS:408
0.024	0.024	0.024	0.0047	0.0071
0.024	0.024	0.024	0.0047	0.0071
0.024	0.024	0.024	0.0047	0.0071
0.0291	0.0291	0.0291	0.0047	0.0071
0.0316	0.0316	0.0316	0.0071	0.0094
0.0291	0.0291	0.0291	0.0047	0.0071
0.0265	0.0265	0.0265	0.0071	0.0094
0.024	0.024	0.024	0	0.0023
0.024	0.024	0.024	0	0.0023
0.0265	0.0265	0.0265	0.0071	0.0094
0.0291	0.0291	0.0291	0.0047	0.0071
0.024	0.024	0.024	0	0.0023
0.024	0.024	0.024	0	0.0023
0.0265	0.0265	0.0265	0.0023	0.0047
0.0265	0.0265	0.0265	0.0023	0.0047
0.0265	0.0265	0.0265	0.0023	0.0047
0.029	0.029	0.029	0.0047	0.007
0.024	0.024	0.024	0	0.0023
0.029	0.029	0.029	0.0047	0.007
0	0	0	0.024	0.0265
0.0316	0.0316	0.0316	0.0071	0.0094
0.029	0.029	0.029	0.0047	0.0023
0.029	0.029	0.029	0.0047	0.007
0	0	0	0.024	0.0265
0.0265	0.0265	0.0265	0.0023	0.0047
0.024	0.024	0.024	0	0.0023
0.0265	0.0265	0.0265	0.0071	0.0094
0.024	0.024	0.024	0	0.0023
	0	0	0.024	0.0265
		0	0.024	0.0265
			0.024	0.0265
				0.0023

Trichothecium sp. B-6	Trichothecium ovalisporum	Trichothecium sp. LPB713	Spicellum roseum GIM 3.476	Trichothecium crotocinigenum CBS:129
0.024	0.024	0	0.024	0.0047
0.024	0.024	0	0.024	0.0047
0.024	0.024	0	0.024	0.0047
0.0291	0.0291	0.0047	0.0291	0.0047
0.0316	0.0316	0.0071	0.0316	0.0071
0.0291	0.0291	0.0047	0.0291	0.0047
0.0265	0.0265	0.0023	0.0265	0.0071
0.024	0.024	0.0047	0.024	0
0.024	0.024	0.0047	0.024	0
0.0265	0.0265	0.0023	0.0265	0.0071
0.0291	0.0291	0.0047	0.0291	0.0047
0.024	0.024	0.0047	0.024	0
0.024	0.024	0.0047	0.024	0
0.0265	0.0265	0.0023	0.0265	0.0023
0.0265	0.0265	0.0023	0.0265	0.0023
0.0265	0.0265	0.0023	0.0265	0.0023
0.029	0.029	0.0094	0.029	0.0047
0.024	0.024	0.0047	0.024	0
0.029	0.029	0.0094	0.029	0.0047
0	0	0.024	0	0.024
0.0316	0.0316	0.0119	0.0316	0.0071
0.029	0.029	0.0094	0.029	0.0047
0.029	0.029	0.0094	0.029	0.0047
0	0	0.024	0	0.024
0.0265	0.0265	0.0023	0.0265	0.0023
0.024	0.024	0.0047	0.024	0
0.0265	0.0265	0.0023	0.0265	0.0071
0.024	0.024	0.0047	0.024	0
0	0	0.024	0	0.024
0	0	0.024	0	0.024
0	0	0.024	0	0.024
0.024	0.024	0.0047	0.024	0
0.0265	0.0265	0.0071	0.0265	0.0023
	0	0.024	0	0.024
		0.0047	0.024	0
			0.024	0.0047
				0.024

Acremonium crotocinigenum cc56	Trichothecium sp. SNT5-9	Trichothecium sp. BCC88172	Hypocreales sp. DN1	Trichothecium ovalisporum NMSC-1
0.0047	0.0047	0.024	0.0341	0.024
0.0047	0.0047	0.024	0.0341	0.024
0.0047	0.0047	0.024	0.0341	0.024
0	0.0094	0.0215	0.0341	0.0291
0.0023	0.0118	0.024	0.0366	0.0316
0	0.0094	0.0215	0.0341	0.0291
0.0071	0.0023	0.0265	0.0366	0.0265
0.0047	0.0094	0.0191	0.029	0.024
0.0047	0.0094	0.0191	0.029	0.024
0.0071	0.0071	0.0265	0.0315	0.0265
0	0.0094	0.0215	0.0341	0.0291
0.0047	0.0094	0.0191	0.029	0.024
0.0047	0.0094	0.0191	0.029	0.024
0.0023	0.0071	0.0215	0.0315	0.0265
0.0023	0.0071	0.0215	0.0315	0.0265
0.0023	0.0071	0.0215	0.0315	0.0265
0.0094	0.0143	0.024	0.034	0.029
0.0047	0.0094	0.0191	0.029	0.024
0.0094	0.0143	0.024	0.034	0.029
0.0291	0.029	0.029	0.047	0
0.0119	0.0168	0.0265	0.0366	0.0316
0.0094	0.0142	0.024	0.034	0.029
0.0094	0.0143	0.024	0.034	0.029
0.0291	0.029	0.029	0.047	0
0.0023	0.0071	0.0215	0.0315	0.0265
0.0047	0.0094	0.0191	0.029	0.024
0.0071	0.0023	0.0265	0.0366	0.0265
0.0047	0.0094	0.0191	0.029	0.024
0.0291	0.029	0.029	0.047	0
0.0291	0.029	0.029	0.047	0
0.0291	0.029	0.029	0.047	0
0.0047	0.0094	0.0191	0.029	0.024
0.0071	0.0118	0.0215	0.0315	0.0265
0.0291	0.029	0.029	0.047	0
0.0047	0.0094	0.0191	0.029	0.024
0.0047	0.0047	0.024	0.0341	0.024
0.0291	0.029	0.029	0.047	0
0.0047	0.0094	0.0191	0.029	0.024
	0.0094	0.0215	0.0341	0.0291
		0.029	0.0392	0.029
			0.0417	0.029
				0.047

Trichothecium sp. BCC86514	Hypocreales sp. B1b0118APD2CC986	Hypocreales sp. 19MT-05-3
0.024	0.0366	0.0417
0.024	0.0366	0.0417
0.024	0.0366	0.0417
0.0215	0.0366	0.0417
0.024	0.0392	0.0443
0.0215	0.0366	0.0417
0.0265	0.0392	0.0443
0.0191	0.0366	0.0366
0.0191	0.0366	0.0366
0.0265	0.0392	0.0443
0.0215	0.0366	0.0417
0.0191	0.0366	0.0366
0.0191	0.0366	0.0366
0.0215	0.0341	0.0392
0.0215	0.0341	0.0392
0.0215	0.0341	0.0392
0.024	0.0417	0.0417
0.0191	0.0366	0.0366
0.024	0.0417	0.0417
0.029	0.0549	0.0549
0.0265	0.0443	0.0443
0.024	0.0418	0.0418
0.024	0.0417	0.0417
0.029	0.0549	0.0549
0.0215	0.0341	0.0392
0.0191	0.0366	0.0366
0.0265	0.0392	0.0443
0.0191	0.0366	0.0366
0.029	0.0549	0.0549
0.029	0.0549	0.0549
0.029	0.0549	0.0549
0.0191	0.0366	0.0366
0.0215	0.0392	0.0392
0.029	0.0549	0.0549
0.0191	0.0366	0.0366
0.024	0.0366	0.0417
0.029	0.0549	0.0549
0.0191	0.0366	0.0366
0.0215	0.0366	0.0417
0.029	0.0417	0.047
0	0.0496	0.0496
0.0417	0.0118	0.0166
0.029	0.0549	0.0549
	0.0496	0.0496
		0.0094
